# Lysine Targeting Group-Transfer Chimeras for Proximity Induction

**DOI:** 10.1002/anie.202512131

**Published:** 2026-02-11

**Authors:** Sameek Singh, Kien Tran, Endri Karaj, Basudeb Mondal, Wenzhi Tian, Surached Siriwongsup, Shaimaa H. Sindi, Uttam Dhawa, Kaushik Pal, Veronika M. Shoba, Sunny Shi, Anna Lian, Jody Mou, Myeonghoon Han, Prashant Singh, Nicholas F. Polizzi, Amit Choudhary

**Affiliations:** Chemical Biology and Therapeutics Science, Broad Institute of MIT and Harvard, Cambridge, MA 02142, USA; Chemical Biology and Therapeutics Science, Broad Institute of MIT and Harvard, Cambridge, MA 02142, USA; Divisions of Renal Medicine and Engineering, Brigham and Women’s Hospital, Boston, MA 02115, USA; Chemical Biology and Therapeutics Science, Broad Institute of MIT and Harvard, Cambridge, MA 02142, USA; Chemical Biology and Therapeutics Science, Broad Institute of MIT and Harvard, Cambridge, MA 02142, USA; Chemical Biology and Therapeutics Science, Broad Institute of MIT and Harvard, Cambridge, MA 02142, USA; Chemical Biology and Therapeutics Science, Broad Institute of MIT and Harvard, Cambridge, MA 02142, USA; Divisions of Renal Medicine and Engineering, Brigham and Women’s Hospital, Boston, MA 02115, USA; Chemical Biology and Therapeutics Science, Broad Institute of MIT and Harvard, Cambridge, MA 02142, USA; Chemical Biology and Therapeutics Science, Broad Institute of MIT and Harvard, Cambridge, MA 02142, USA; Chemical Biology and Therapeutics Science, Broad Institute of MIT and Harvard, Cambridge, MA 02142, USA; Department of Chemistry, Indian Institute of Technology Tirupati, Yerpedu-Venkatagiri Road, Yerpedu, AP 517619, India; Chemical Biology and Therapeutics Science, Broad Institute of MIT and Harvard, Cambridge, MA 02142, USA; Chemical Biology and Therapeutics Science, Broad Institute of MIT and Harvard, Cambridge, MA 02142, USA; Department of Cancer Biology, Dana-Farber Cancer Institute, Boston, MA 02215, USA; Department of Biological Chemistry and Molecular Pharmacology, Harvard Medical School, Boston, MA 02215, USA; Department of Cancer Biology, Dana-Farber Cancer Institute, Boston, MA 02215, USA; Department of Biological Chemistry and Molecular Pharmacology, Harvard Medical School, Boston, MA 02215, USA; Chemical Biology and Therapeutics Science, Broad Institute of MIT and Harvard, Cambridge, MA 02142, USA; Chemical Biology and Therapeutics Science, Broad Institute of MIT and Harvard, Cambridge, MA 02142, USA; Department of Cancer Biology, Dana-Farber Cancer Institute, Boston, MA 02215, USA; Department of Biological Chemistry and Molecular Pharmacology, Harvard Medical School, Boston, MA 02215, USA; Chemical Biology and Therapeutics Science, Broad Institute of MIT and Harvard, Cambridge, MA 02142, USA; Department of Medicine, Harvard Medical School, Boston, MA 02115, USA; Divisions of Renal Medicine and Engineering, Brigham and Women’s Hospital, Boston, MA 02115, USA

**Keywords:** Group transfer, Immunotherapy, Lysine, Protein modifications, Proteomics

## Abstract

Nature employs post-translational modifications (PTMs) to induce proximity between proteins by engendering new interactions. Furthermore, we find that protein ligands are invariably proximal to a lysine. Inspired by these two observations, we developed group-transfer chimeras (GRCs) that append a moiety-of-interest to the lysine side chain. GRCs employ a protein’s ligand and a handle with a *transferase*-type reactivity to modify the proximal lysine. Contemporary lysine-targeting group-transfer handles were incompatible with GRCs due to their hydrolytic instability, large size, high reactivity, and synthetic incompatibility with diverse ligands. Accordingly, we developed an *N*-(sulfonyl)-*N*-(trifluoroethyl)-ethanamide (SuFA) handle that is stable, small, and exhibits tunable reactivity and synthetic compatibility with diverse ligands and proteins. Using GRCs that group-transfer binders of tags (e.g., HaloTag, FKBP) onto proteins overexpressed in cancer cells, we displayed these binders on the surface of the cancer cell. With a universal T cell engager (UniTE) that binds to the displayed ligands and T cells, these GRCs induced proximity between cancer cells and cytotoxic T cells, leading to the latter’s activation. We envision the GRC platform to find utility in basic research and biomedicine.

## Introduction

**Figure 1.**
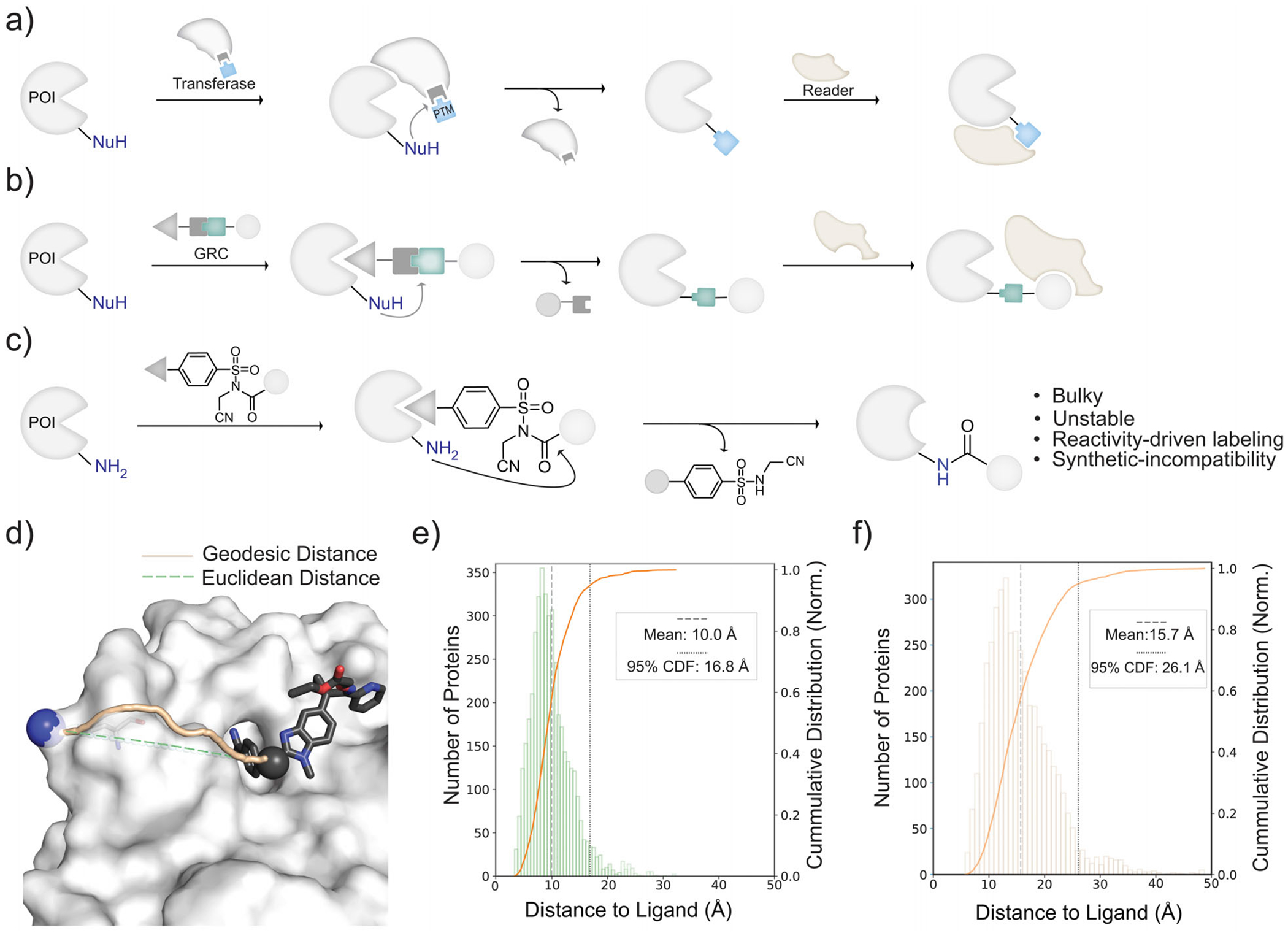
a) Schematic of a group-transfer reaction catalyzed by a *transferase* to append a post-translational modification (PTM) on a protein-of-interest (POI). b) GRC mediated group-transfer reaction to tag a protein binder to a proximal nucleophile (e.g., cysteine). c) Previously reported lysine-reactive *N*-acyl-*N*-alkyl sulfonamide (NASA) handle. d) Cartoon representation of geodesic or Euclidean distances from the ligand to lysine. e) and f) Distributions of the distances between the ligand and lysine: Euclidean distance (e) or geodesic distance (f).

Post-translational modifications (PTMs) are often appended to the protein-of-interest (POI) via a group-transfer reaction catalyzed by a *transferase* (Figure 1a).^[1]^ For example, kinases facilitate the transfer of a phosphoryl group from ATP to the POI.^[2]^ These modifications are recognized by readers (e.g., SH2 domains for phosphorylation) to engender new protein-protein interactions. We recently reported group-transfer chimeras (GRCs) whose binding to POI triggers a group-transfer reaction with a proximal cysteine, resulting in the tagging of POI’s cysteine with the binder of another protein, enabling new protein-protein interaction (Figure 1b).^[3–5]^ However, these cysteine-based GRCs are limited for multiple reasons, including the limited availability of cysteines proximal to the ligand-binding site and their near absence among extracellular proteins or in oxidative environments. In contrast, lysines are considerably more abundant in the human proteome (5.8% versus 1.9% for cysteine) and present on both intracellular and extracellular proteins.^[6,7]^

Notwithstanding these advantages, contemporary lysine group-transfer handles are incompatible with GRCs for multiple reasons (Figure 1c). First, contemporary handles are too reactive, leading to indiscriminate labeling of both proximal and distal lysines from the ligand’s binding pocket (vide infra). Such non-specific labeling will engender a confounding number of protein–protein interfaces and biological activities. Second, contemporary handles are hydrolytically unstable, incompatible with serum, exhibit poor shelf life,^[8,9]^ and are synthetically challenging to incorporate for many ligands with basic/nucleophilic groups. These attributes not only make them difficult to work with but also hinder the GRC’s ability to facilitate protein-protein interactions, as the hydrolyzed components compete with both the POI and the target protein for binding. Third, the large molecular footprint of contemporary group-transfer moieties exacerbates the large size of GRCs, preventing access to lysines in deep or constrained pockets. Finally, the pKa and reactivity of lysine vary widely across POIs,^[10,11]^ requiring tunable group-transfer handles.

Herein, we report GRCs for lysine that engender new protein–protein interactions with downstream biological activities, including the induction of proximity between cancer and T cells. Using structural bioinformatics, we identify > 4000 POI-ligand complexes (Table S1) with a proximal lysine. We optimized group-transfer handles for lysine that are miniature and have tunable reactivity and stability, yielding *N*-(sulfonyl)-*N*-(trifluoroethyl) ethanamide (SuFA), which was used for GRC generation and validation. We developed a robust synthetic platform for incorporating SuFA in diverse ligands, including peptides and small molecules, and confirmed that SuFA enabled proximity-guided labeling of diverse POIs. Finally, GRCs built from SuFA engender new protein–protein interactions that induce proximity between cancer cells and T cells, providing a new avenue for immunotherapy.

## Results and Discussion

### Most Ligand-Binding Pockets Possess a Proximal Lysine

The success of proteolysis targeting chimeras (PROTACs), which leverage the high abundance of surface-exposed lysine on the target protein for ubiquitination,^[12]^ led us to hypothesize that most binding pockets will have a proximal lysine. We tested this hypothesis by analyzing structures of protein-ligand complexes in PDBbind database, a curated collection of high-resolution (<3 Å) protein-ligand co-crystal structures with experimentally determined binding affinities.^[13]^ We calculated the geodesic distance (i.e., surface path, Figure 1d,f) and Euclidean distance (Figure 1d,e) between lysines and the bound ligand. We surmised that the Euclidean distance may be important for proteins with high dynamics. Notably, 95% of protein-ligand complexes analyzed contained a lysine within 17 Å (versus 21 Å for cysteine) by Euclidean distance and within 27 Å (versus 38 Å for cysteine) by geodesic distance (Figures 1e,f and S1b,c). We acknowledge that both the surface and Euclidean distances, while offering initial design guidance based on static crystal structures, may not fully represent the complete range of distances sampled by the dynamic protein ensemble. These observations motivated us to deploy the reported *N*-acyl-*N*-alkyl sulfonamide (NASA)^[14]^ as a lysine-targeting reactive group for developing GRCs.

### NASA Labels both Proximal and Distal Lysine for Diverse Ligands

**Figure 2.**
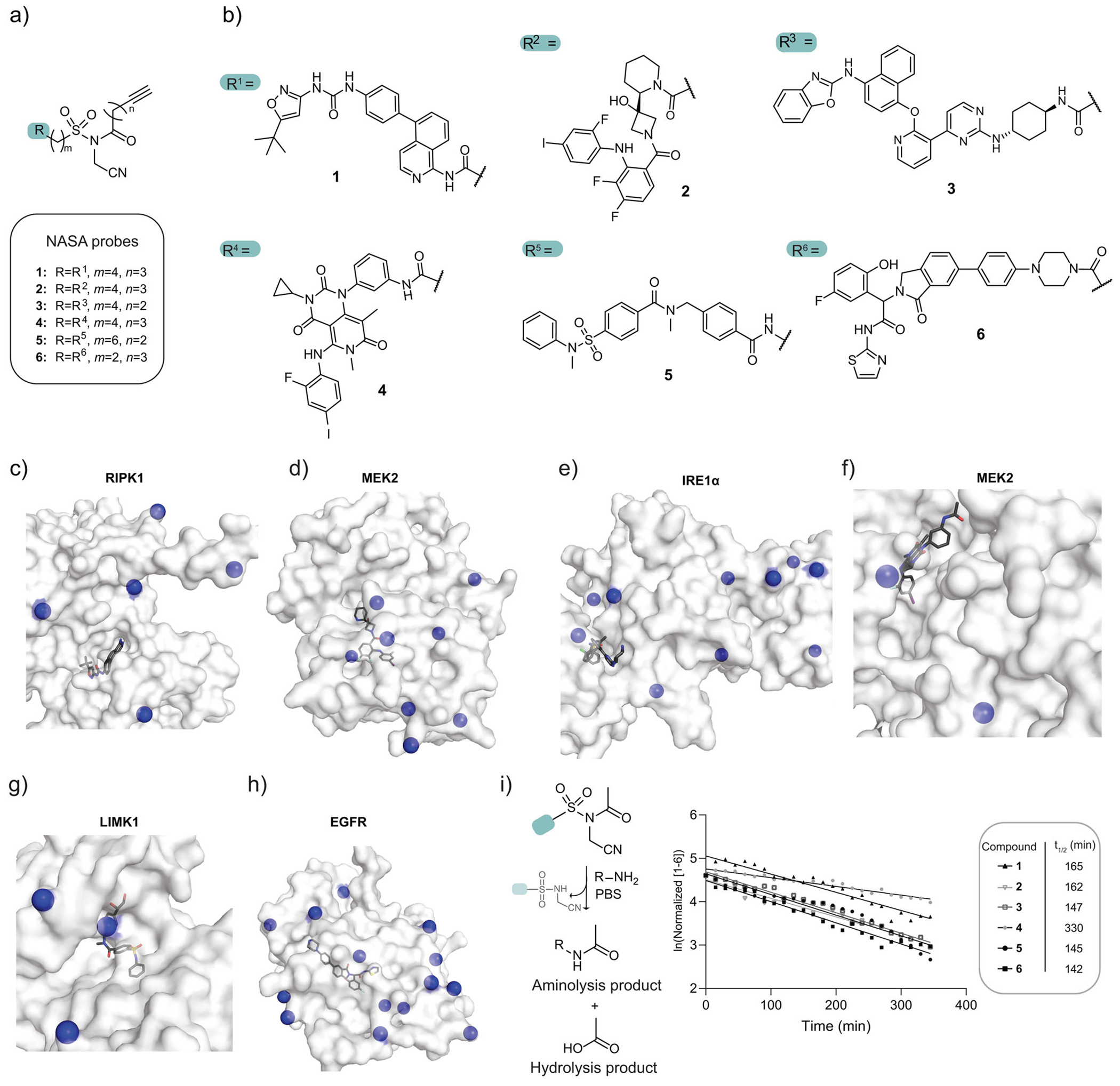
a) and b) Structure of NASA probes utilizing diverse allosteric inhibitors. **c)–h)** Lysine labeling for: (c) RIPK1 by **1** (d) MEK2 by **2**, (e) IRE1*α* by **3**, (f) MEK2 by **4**, (g) LIMK1 by **5**, and (h) EGFR by **6**; lysines labeled by NASA probes appear as dark blue spheres. i) Reactivity assessment of NASA probes with Boc-Lys-Coumarin in acetonitrile-PBS, pH 7.4 (1:4).

The GRC design strategy requires that the *transferase*-type reactivity operate via an induced-proximity mechanism where GRC binding to POI enhances the effective molarity, triggering labeling of proximal but not distal lysine(s). In contrast, group transfer by a reactivity-driven mechanism will result in labeling of lysines distal from the binding pocket. To confirm that NASA operates by an induced-proximity mechanism, we selected six allosteric inhibitors targeting RIPK1,^[15]^ MEK2,^[16]^ IRE1*α*,^[17]^ MEK2,^[18]^ LIMK1,^[19]^ or EGFR^[20]^ (Figure 2a). The choice of allosteric binding sites (versus active sites) ensures diversity since active-site pockets are highly conserved. Furthermore, we selected high-affinity inhibitors (affinity < 200 nM; Figure S2A)^[15–18]^ and, as controls, a LIMK1 inhibitor (affinity > 3 μM, Figure S2A) and a mutant-selective EGFR inhibitor (0.6% wild-type EGFR binding in KINOMEscan assay, Figure S2A). We appended NASA with an alkyne handle to the solvent-exposed site on the inhibitors (Figure 2a,b) and used an in-gel fluorescence experiment to determine target protein labeling (Figure S2C). Here, the target protein is incubated with the NASA-alkyne probe, followed by a click reaction with a fluorophoreazide. We observed significant target protein labeling for all probes, prompting us to identify labeled lysine(s) using mass spectrometry. Unfortunately, the mass spectrometry studies indicated widespread labeling of both proximal and distal lysines, regardless of inhibitor affinity or probe concentration, suggesting that group transfer by NASA was not primarily induced-proximity driven (Figures 2a–h and S3–S8). Notably, concentration-dependent studies with RIPK1 (Figure S3) and MEK2 (Figure S6) revealed both proximal and distal lysine labeling, whereas, for LIMK1 (Figure S7), only the distal lysine was labeled at the lowest concentration. Furthermore, the NASA probes were hydrolytically unstable and had a poor shelf life. Indeed, the probes rapidly reacted with lysine-coumarin in PBS, exhibiting a short half-life (*t*_1/2_ ~ 2.5 h, Figure 2i).^[8,9]^ Since many protein ligands possess basic groups, the observed enhanced reactivity is a potential concern. These studies prompted us to develop a group-transfer handle with tunable reactivity and enhanced stability.

### Development of Group-Transfer Handles with Tunable Reactivity and Stability

**Figure 3.**
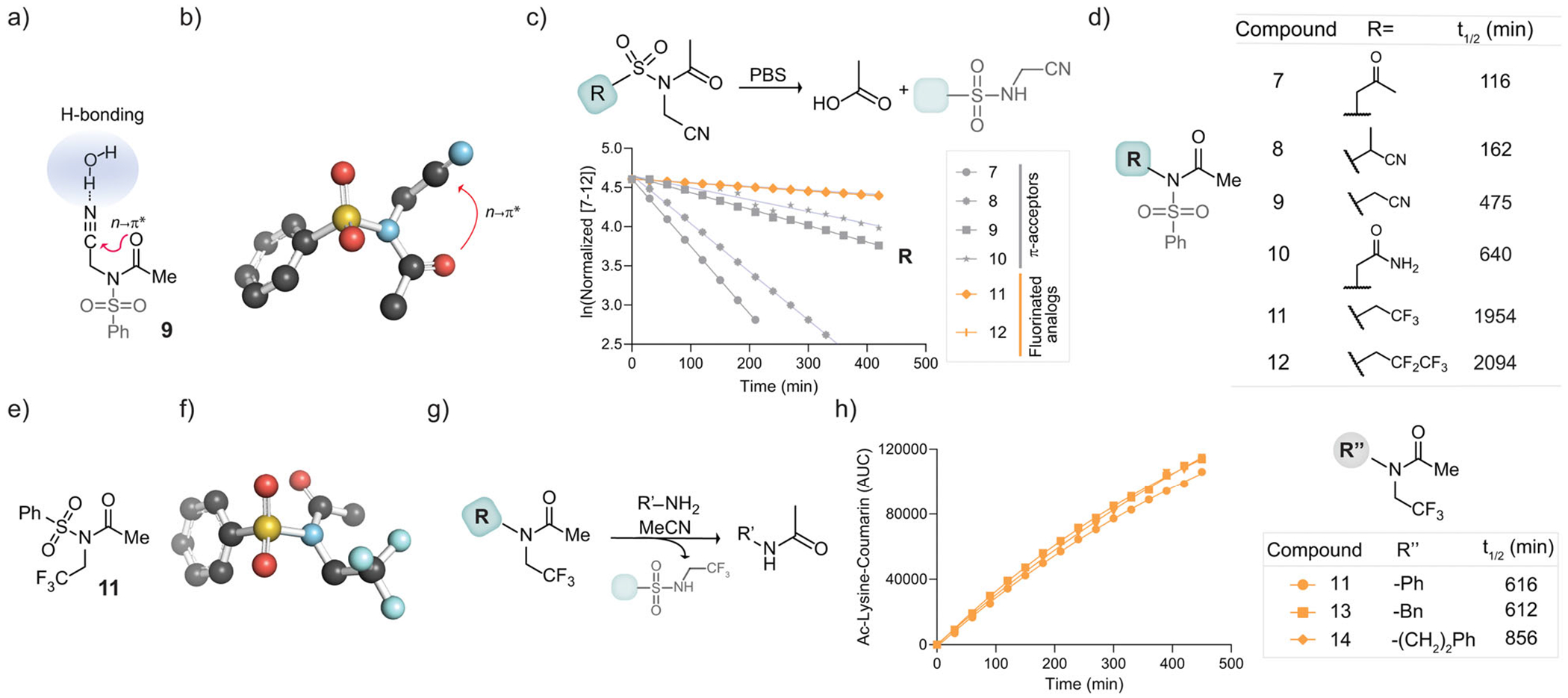
a) The nitrile group in NASA can hydrogen bond to yield a hydration sphere or withdraw electron density from the carbonyl via *n*→π* electron interaction. b) Crystal structure of NASA compound **9** (CDCC number 2450818). c) and d) Hydrolysis kinetics of *π*-acceptors (**7**–**10**) and –CF_3_/–CF_2_CF_3_ analogs in PBS, pH 7.4. e) and f) Crystal structure of –CF_3_ compound **11** (CDCC number 2450817). g) and h) Reactivity of SuFA analogs with Boc-Lys-Coumarin in acetonitrile.

We began our efforts to optimize group-transfer handles by examining the factors contributing to NASA’s high reactivity. We hypothesized that the nitrile group in NASA might be a potential source of instability, either by acting as a hydrogen bond acceptor or a *π*-acceptor (Figure 3a). Studies on carboxypeptidases^[21–23]^ and activated amides^[24,25]^ have demonstrated that hydrogen bond acceptors can facilitate amide hydrolysis. Furthermore, the nitrile group can withdraw electron density from the carbonyl group via *n*→π* electron donation^[26]^ from carbonyl oxygen into the π* orbital of the nitrile group; such interactions have been reported to impact amide’s hydrolytic stability.^[27–29]^ We obtained a crystal structure of **9** (Figure 3b) and observed a short contact of 3.0 Å (distance shorter than the sum of van der Waal’s radii) between the donor carbonyl oxygen and the acceptor nitrile carbon, indicative of*n*→π* electron donation (Figure 3b). Furthermore, we synthesized NASA analogs in which the nitrile group is replaced with a carbonyl or amide group (good H-bond and *π*-acceptor) or a −CF_3_/−CF_2_CF_3_ group (poor H-bond and *π*-acceptor) (Figure 3c,d).^[30]^ While nitrile, carbonyl, and amide analogs exhibited poor hydrolytic stability (compound **7**–**9**, *t*_1/2_ of 2–10 h), the −CF_3_/−CF_2_CF_3_ analogs (**11, 12**) were ~3 times more hydrolytically stable than nitrile analogs (Figure 3d). Interestingly, the crystal structure of **11** revealed a conformation in which the carbonyl and −CF_3_ groups are *trans* compared to the *cis* conformation for nitrile analog **9** (Figure 3e,f), suggesting a potential role of*n*→π* interaction in NASA’s conformational stability.

Nearly all reports of the NASA group employ bulky aryl sulfonamide moiety,^[14,31]^ which is particularly undesirable as GRCs already contain ligands for target binding and effector function. We hypothesize that the aryl sulfonamide in reported NASA can be replaced with alkyl sulfonamide without significantly impacting its reactivity or hydrolytic profile because, in contrast to benzamides, phenylsulfonamides exhibit weak resonance.^[32–34]^ Indeed, such replacements for −CF_3_, −CF_2_CF_3_, or −CN did not significantly perturb hydrolytic stability or aminolysis (Figures 3d,h and S2E). Since lysine pKa (and reactivity) can vary considerably in proteins, we generated a library of NASA analogs by modifying the *N*-alkylation or the sulfonyl groups. For example, we tested *N*-heterocyclic substitutions, including pyridyl, pyrimidyl, and piperidyl,^[35]^ some of which were also independently reported by Hamachi and co-workers.^[8]^ An analysis of the hydrolytic stability, lysine reactivity, size, and ease of synthesis of all the handles suggested that −CF_3_ group (compound **11**) exhibits a balanced profile, and we prioritized this handle for GRC platform development. Per the nomenclature of International Union for Pure and Applied Chemistry (IUPAC),^[36]^ the reactive moiety should be called *N-*((*λ*^3^-methyl)sulfonyl*)-N*-(2,2,2-trifluoroethyl)-2*λ*^3^-ethanamide, which we abbreviate as SuFA.

### SuFA Displays Proximity-Driven Group Transfer at Diverse Binding Sites

We selected five protein targets (GLP1R, FPR2, PSMA, VEGFR2, and BTK) for GRC development as they allow the assessment of multiple factors, including target location (membrane versus cytosol), binding site (intracellular versus extracellular), ligand nature (peptidic versus small molecule), and diverse downstream biology. Here, GLP1R, FPR2, PSMA, and VEGFR2 are membrane-bound, while BTK is cytosolic. We selected peptidic ligands for FPR2^[37]^ and GLP1R^[38]^ and small molecule ligands for PSMA,^[39]^ VEGFR2,^[40]^ and BTK.^[41]^ The ligands target intracellular binding sites for BTK and VEGFR2, and extracellular sites for the other targets. Finally, the selected proteins are high-value therapeutic targets for metabolic disorders (GLP1R), inflammatory diseases (FPR2), and cancers (PSMA, VEGFR2, BTK).^[42–46]^

**Figure 4.**
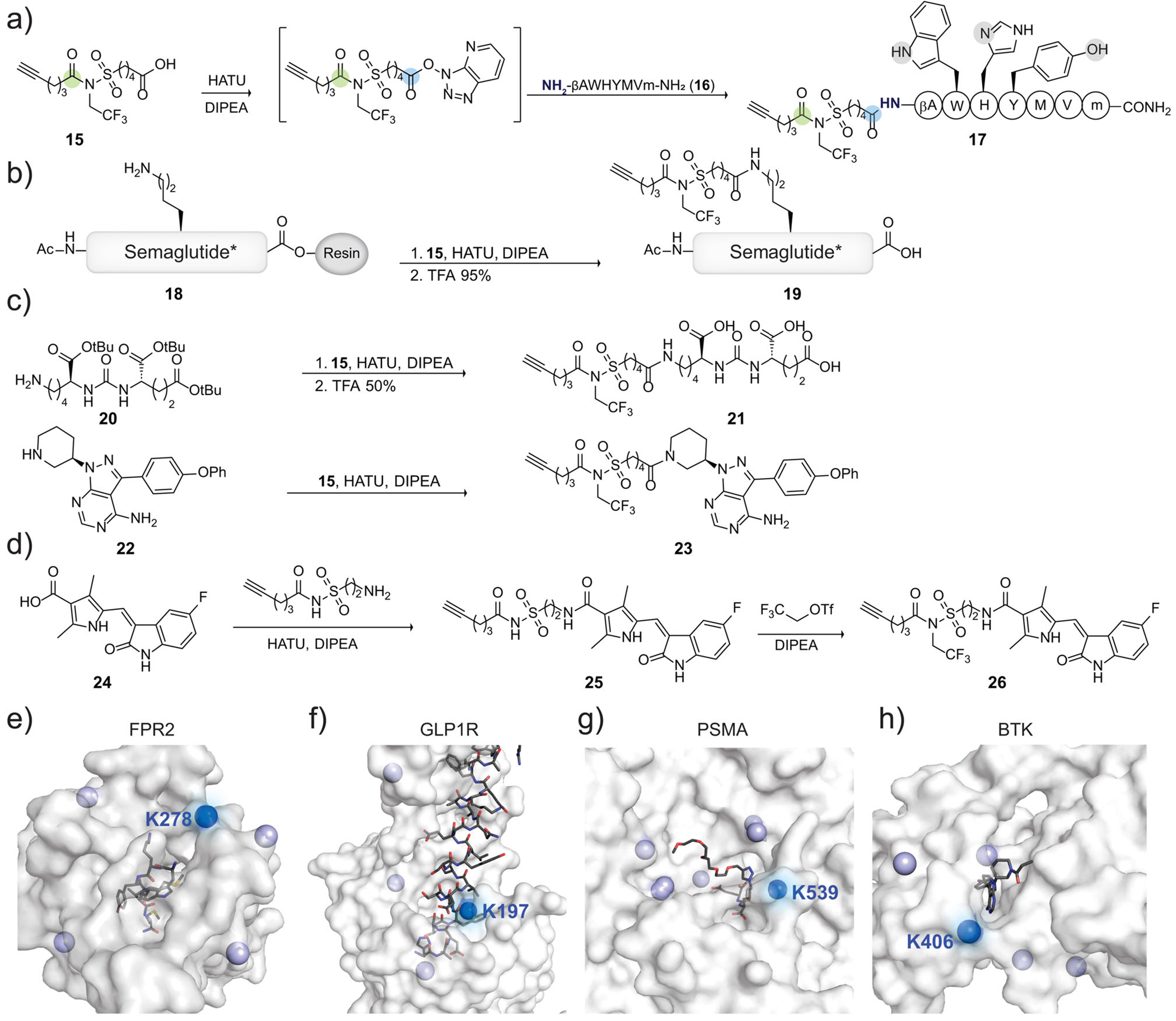
a) Synthesis of FPR2 SuFA probe. b) Solid-phase peptide synthesis of a semaglutide-based SuFA probe. c) Synthesis of small molecule-based SuFA probe. d) Synthesis of small molecule-based SuFA probe using SuFA amine handle. e)–h) SuFA probes mediated binding site mapping for: e) FPR2, f) GLP1R, g) PSMA, and h) BTK. Lysine amines within 10 Å of the ligand are shown as light blue spheres, lysines labeled by SuFA probes appear as dark blue spheres with annotated residue numbers.

Next, we developed a synthesis platform to append SuFA to the target ligands. We designed handle **15** bearing SuFA, alkyne and carboxyl group for ligand conjugation. We successfully coupled **15** (Figure 4a) to FPR2 ligand **16** with unprotected nucleophilic residues (Trp, His, Tyr) to yield **17**. Since NASA was originally developed for use on the solid support as Kenner’s safety-catch handle,^[47,48]^ we explored the compatibility of SuFA **15** with solid-phase peptide synthesis using semaglutide analog (Figure 4b). Here, the handle-bearing alkyne and SuFA were conjugated to the lysine side chain, and gratifyingly, the SuFA group was compatible with the acidic conditions used for peptide cleavage (95% trifluoroacetic acid). The relative ease of synthesis allowed the rapid generation of several semaglutide analogs bearing SuFA (**19** and **S1**-**S5**). A similar approach was used for conjugating linker **15** to PSMA and BTK ligands to yield **21** and **23**, respectively (Figure 4c). Finally, the VEGFR2 ligand (sunitinib), which has a free carboxyl group, required an assembly of **26** directly (Figure 4d). Overall, SuFA is compatible with both solution- and solid-phase synthesis and can be readily appended in a modular fashion to both peptidic and small-molecule ligands. Importantly, the tempered reactivity of SuFA allows for synthetic procedures under both basic conditions (amide coupling) and harsh acidic conditions (peptide cleavage using 95% TFA), which are potentially not suitable for NASA reactive groups. It also ensures the resulting probes are stable enough to be isolated at reasonable yield and remain intact for long-term storage, even for molecules bearing multiple functional groups (phenol, indoles, aromatic amine) such as compounds **17**, **19**, **21**, **23**.

With SuFA probes in hand, we confirmed proximity-driven labeling using immunoprecipitation–mass spectrometry (IP-MS) on HEK293T cells transiently expressing BTK, FPR2, PSMA, or GLP1R. SuFA probes were compatible with serum-containing media, similar to the latest generation ArNASA, unlike alkyl NASA probes that exhibit poor stability.^[8,14,49]^ Furthermore, we detected only one labeled site per target protein near the binding pocket (Figures 4e–h and S9–S16), unlike NASA probes that labeled multiple lysines at distant sites (Figures S3–S8). Under biochemical conditions, we observed that only one lysine (K-838) was labeled dose-dependently by VEGFR2-targeting **26**, consistent with proximity-driven labeling (Figure S11). Furthermore, while there are seven lysines within 10 Å of the binding site in PSMA, only one lysine was labeled (Figures 4g and S12). The in-gel fluorescence analysis of the LNCaP whole cell lysate also demonstrated the selectivity of probe **21** for PSMA (Figure S21).

For GLP1R, peptidic ligands with SuFA placed near the *N*-terminus (**19**, Figures S10, and **S1**–**S3**, Figures S13–S15) showed lysine labeling, while those near C-terminus (**S4** and **S5**) showed no labeling. Finally, the small size of SuFA (versus NASA) enabled labeling of a deeply seated lysine (by **17** in FPR2) that will be difficult to access using a bulkier probe (Figure 4e). These studies provide an opportunity to develop covalent ligands for GLP1R, which is being targeted for obesity and other metabolic disorders. Furthermore, the higher specificity of SuFA may facilitate the facile identification of the binding site compared to diazirine-based methods,^[50,51]^ which yield complex mixtures of labeled products that are difficult to identify using mass spectrometry.^[52,53]^

To determine if SuFA probes exhibited proximity-driven and reduced heterogeneous labeling exhibited by NASA probes, we performed a series of competition, kinetics, and mass spectrometry experiments using a matched pair of SuFA and NASA probes for two targets—RIPK1 and VEGFR (**S6**, Figure S22). In a competition-based in-gel fluorescence experiment, RIPK1 was pre-incubated with its inhibitor, followed by treatment with NASA or SuFA probes containing an alkyne handle to which a fluorescent dye can be “clicked.” The labeling by the NASA probe was unabolished, even with a 10 × excess of competitor (>40% labeling remaining, Figure S22A–C), suggesting significant reactivity-driven, non-specific labeling. In contrast, labeling by the SuFA probe S6 was significantly blocked (>90% competition) under the same conditions (Figure S22B). Using mass spectrometry, we confirmed that while the NASA probe labeled 4–5 lysines of RIPK1 (Figures 2c, S3A, and S3H), the SuFA probe **S6** mostly labeled one lysine (Figures S3H and S22D). We also assessed the reaction kinetics of matched SuFA and NASA probes of VEGFR2 (Figure S23).^[54]^ The observed rate constant (*k*_obs_) for the SuFA probe **26** (1.1 × 10^−3^ s^−1^) was lower than that of the NASA probe **S7** (3.4 × 10^−3^ s^−1^). However, the competition experiment again confirmed significant off-target labeling by the NASA probe, with only a 67% reduction in labeling in the presence of an excess of competitor, while more than 90% reduction was observed for the corresponding SuFA probe. Overall, the SuFA probes exhibited less heterogeneous labeling of the lysines than NASA probes, which is critical for GRCs function.

### SuFA-Based GRCs Enable the Recruitment of T cells to Cancer Cells

Bispecific T-cell engagers (BiTEs) are an emergent class of therapeutics that simultaneously bind to cancer cell surface antigen and cytotoxic T cells, triggering proximity-driven death of cancer cells.^[55,56]^ The constancy of the transferred moiety by GRC, regardless of the ligand used, enables the recognition of diverse cancer antigens with a single T-cell engager. This modularity provides an advantage over traditional modalities, allowing a single, established BiTE to be rapidly repurposed against multiple or newly emergent tumor antigens simply by switching the guiding small-molecule ligand. Leveraging this simplifying feature of GRCs, we developed Universal T-cell Engagers (UniTEs) that bind T cells and a specific moiety displayed on cancer cells. We initiated our investigation using PSMA, a prostate cancer antigen for which we had previously validated lysine labeling using SuFA. For the displayed moiety, we employed chloroalkane (which binds to HaloTag)^[57]^ or AP1867 (which binds to FKBP^F36V^)^[ 58,59]^ and generated corresponding UniTEs by fusing HaloTag or FKBP^F36V^ with the anti-CD3 scFv.^[60,61]^

**Figure 5.**
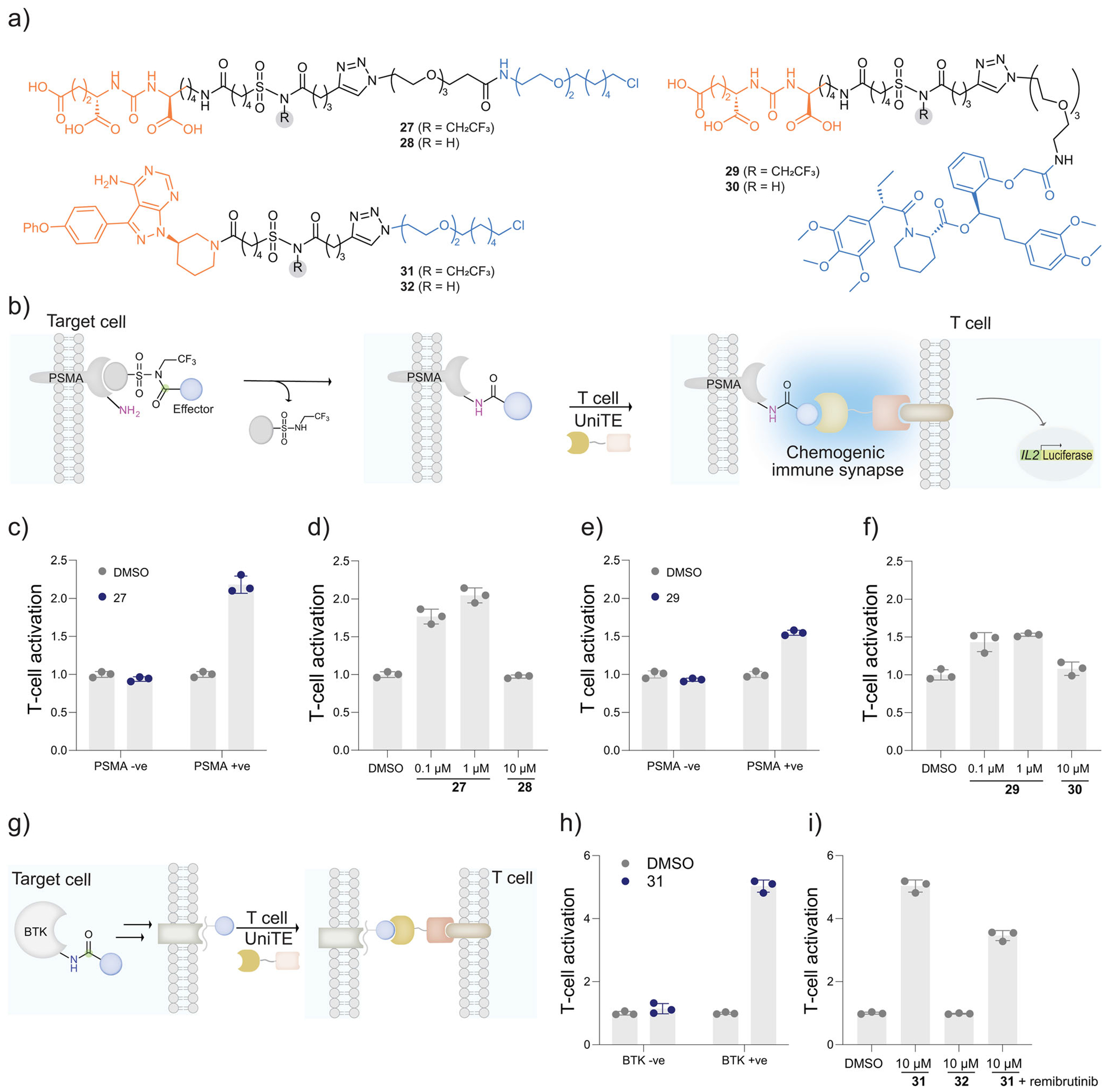
a) Structure of GRC targeting PSMA, BTK, and their non-covalent analogs. b) Schematic of the GRC-mediated modification of PSMA and reporter assay for quantifying T-cell activation. c)–f) T-cells activation assay using covalent Halo-PSMA-GRC **27**, FKBP-PSMA-GRC **29,** and their non-covalent analogs **28** and **30**, respectively. g) Schematic of the GRC-mediated modification of an intracellular protein BTK and reporter assay for quantifying the T-cell activation. h)–i) T-cells activation assay using covalent BTK GRC **31**, its non-covalent analog **32**, and BTK selective inhibitor (remibrutinib). Data is from at least three individual experiments performed in triplicate.

We used the previously described T-cell activation reporter assay to assess proximity induction between cancer and T cells.^[62–64]^ Here, as a model for cytotoxic T cells, we used a Jurkat cell line with an *IL2*-promoter-driven *luciferase* (Figure 5b). Upon formation of chemogenic synapse between the cancer cell and the Jurkat cell mediated by UniTE, the signal transduction in Jurkat cells results in expression of the luciferase, whose bioluminescence serves as a proxy for T-cell activation. PSMA-expressing (LNCaP) and non-expressing (DU145) cells^[65]^ were treated with GRC **27**, its non-covalent analog **28**, or vehicle, followed by HaloTag UniTE and IL-2 Jurkat reporter cells co-culture (Figure 5 c,d).^[66]^ We observed significantly increased T-cell activation by **27** in PSMA-expressing LNCaP cells compared to DU145 (Figure 5c). Importantly, the covalent GRC **27** showed dose-dependent T-cell activation and significantly outperformed its non-covalent analog **28**, demonstrating efficacy even at a 100-fold lower concentration (Figure 5d). Similar results were obtained using FKBP^F36V^-based UniTE and FKBP-based GRC **29** or the non-covalent analog **30** (Figure 5e,f). These results suggest that covalency can lead to an improved T-cell response, potentially arising from the increased residence time of T cells on cancer cells, as reported recently.^[67]^

Recent studies have shown that cysteine-targeting covalent inhibitors can be displayed via MHC-I pathway, triggering T-cell activation using BiTEs that recognize the covalent inhibitors and T cells. ^[66,68–70]^ We investigated whether GRCs could similarly install moieties on intracellular proteins for subsequent MHC-I display that can be recognized by UniTEs (Figure 5g). Here, we used BTK as a model protein because prior work has demonstrated that an ibrutinib-labeled BTK peptide is displayed via MHC-I and elicits T-cell activation.^[68]^ We have previously validated BTK lysine-labeling with GRC **31** (Figure 4h). We treated BTK-expressing cell line (Raji)^[ 71]^ and non-expressing cell line (HEK293T)^[72]^ with BTK-targeting GRC **31**, its non-covalent analog **32**, or vehicle, then co-cultured them with the IL-2 Jurkat reporter cells in the presence of a HaloTag UniTE (Figure 5h). We observed significantly increased T-cell activation in the case of BTK-expressing Raji compared to non-expressing HEK293T cells. No T-cell activation was observed in the presence of non-covalent analog **32** and the activation was significantly diminished in the presence of competitor, remibrutinib,^[73]^ a BTK selective inhibitor (Figure 5i). These studies confirm that GRCs can recruit T cells to cancer cells by targeting intra/extracellular oncoproteins via installing a universal chemical epitope.

### Conclusion

In this study, we mined the PDB and found that lysine residues are abundantly present near ligand-binding pockets. We developed a hydrolytically stable SuFA handle for targeting lysines, enabling a robust group-transfer for both small molecules and peptide ligands. We demonstrated proximity-driven labeling of SuFA probes for FPR2, PSMA, BTK, and GLP1R, and identified SuFA-modified lysine in live cells using mass spectrometry. Compared to traditional diazirine-based methods, this chemoproteomic strategy holds the potential to simplify binding-site mapping and downstream computational analysis, which will be investigated in our future studies. The results can be combined with machine learning approaches such as DiffDock^[74]^ to accelerate ligand discovery for new targets. Finally, we developed a universal T cell recruitment system based on GRCs by installing chemical epitopes on either extracellular (PSMA) or intracellular (BTK) oncogenic proteins. Overall, we envision the lysine-targeting GRC platform will provide versatile tools for interrogating and enhancing protein function.

## Supplementary Material

Supplementary material

Additional supporting information can be found online in the Supporting Information section

## Data Availability

The data that support the findings of this study are available in the Supporting Information of this article.

## References

[1] Walsh C, Post-translational modification of proteins: expanding nature’s inventory, Roberts and Company Publishers, Greenwood Village, CO 2006.

[2] Manning G, Whyte DB, Martinez R, Hunter T, Sudarsanam S, Science 2002, 298, 1912–1934, 10.1126/science.1075762. 12471243

[3] Pergu R, Shoba VM, Chaudhary SK, Munkanatta Godage DNP, Deb A, Singha S, Dhawa U, Singh P, Anokhina V, Singh S, Siriwardena SU, Choudhary A, ACS Cent. Sci 2023, 9, 1558–1566, 10.1021/acscentsci.3c00200. 37637727 PMC10450875

[4] Reddi RN, Rogel A, Gabizon R, Rawale DG, Harish B, Marom S, Tivon B, Arbel YS, Gurwicz N, Oren R, David K, Liu J, Duberstein S, Itkin M, Malitsky S, Barr H, Katz BZ, Herishanu Y, Shachar I, Shulman Z, London N, J. Am. Chem. Soc 2023, 145, 3346–3360, 10.1021/jacs.2c08853. 36738297 PMC9936582

[5] London N, Chem. Rev 2025, 125, 326–368, 10.1021/acs.chemrev.4c00570. 39692621 PMC11719315

[6] Jones A, Zhang X, Lei X, Cell Chem. Biol 2017, 24, 537–539, 10.1016/j.chembiol.2017.05.003. 28525765

[7] Bairoch A, Apweiler R, Nucleic Acids Res. 2000, 28, 45–48, 10.1093/nar/28.1.45. 10592178 PMC102476

[8] Kawano M, Murakawa S, Higashiguchi K, Matsuda K, Tamura T, Hamachi I, J. Am. Chem. Soc 2023, 145, 26202–26212, 10.1021/jacs.3c08740. 37987622

[9] Tamura T, Hamachi I, Acc. Chem. Res 2025, 58, 87–100, 10.1021/acs.accounts.4c00628. 39661110

[10] Isom DG, Castañeda CA, Cannon BR, García-Moreno B, Proc. Natl. Acad. Sci. USA 2011, 108, 5260–5265, 10.1073/pnas.1010750108. 21389271 PMC3069169

[11] Platzer G, Okon M, McIntosh LP, Biomol J. NMR 2014, 60, 109–129, 10.1007/s10858-014-9862-y. 25239571

[12] Békés M, Langley DR, Crews CM, Nat. Rev. Drug Discov 2022, 21, 181–200. 35042991 10.1038/s41573-021-00371-6 PMC8765495

[13] Wang R, Fang X, Lu Y, Wang S, J. Med. Chem 2004, 47, 2977–2980, 10.1021/jm030580l. 15163179

[14] Tamura T, Ueda T, Goto T, Tsukidate T, Shapira Y, Nishikawa Y, Fujisawa A, Hamachi I, Nat. Commun. 2018, 9, 1870, 10.1038/s41467-018-04343-0. 29760386 PMC5951806

[15] Harris PA, Bandyopadhyay D, Berger SB, Campobasso N, Capriotti CA, Cox JA, Dare L, Finger JN, Hoffman SJ, Kahler KM, Lehr R, Lich JD, Nagilla R, Nolte RT, Ouellette MT, Pao CS, Schaeffer MC, Smallwood A, Sun HH, Swift BA, Totoritis RD, Ward P, Marquis RW, Bertin J, Gough PJ, ACS Med. Chem. Lett 2013, 4, 1238–1243, 10.1021/ml400382p. 24900635 PMC4027519

[16] Heinzerling L, Eigentler TK, Fluck M, Hassel JC, Heller-Schenck D, Leipe J, Pauschinger M, Vogel A, Zimmer L, Gutzmer R, ESMO Open 2019, 4, e000491. 31231568 10.1136/esmoopen-2019-000491 PMC6555610

[17] Harrington PE, Biswas K, Malwitz D, Tasker AS, Mohr C, Andrews KL, Dellamaggiore K, Kendall R, Beckmann H, Jaeckel P, Materna-Reichelt S, Allen JR, Lipford JR, ACS Med. Chem. Lett 2015, 6, 68–72, 10.1021/ml500315b. 25589933 PMC4291719

[18] Pratt EC, Isaac E, Stater EP, Yang G, Ouerfelli O, Pillarsetty N, Grimm J, J. Nucl. Med 2020, 61, 1845–1850, 10.2967/jnumed.120.241901. 32444378 PMC9364901

[19] Goodwin NC, Cianchetta G, Burgoon HA, Healy J, Mabon R, Strobel ED, Allen J, Wang S, Hamman BD, Rawlins DB, ACS Med. Chem. Lett 2015, 6, 53–57, 10.1021/ml500242y. 25589930 PMC4291701

[20] To C, Jang J, Chen T, Park E, Mushajiang M, De Clercq DJH, Xu M, Wang S, Cameron MD, Heppner DE, Shin BH, Gero TW, Yang A, Dahlberg SE, Wong KK, Eck MJ, Gray NS, Jänne PA, Cancer Discov. 2019, 9, 926–943, 10.1158/2159-8290.CD-18-0903. 31092401 PMC6664433

[21] Kumar P, Reithofer V, Reisinger M, Wallner S, Pavkov-Keller T, Macheroux P, Gruber K, Sci. Rep 2016, 6, 23787, 10.1038/srep23787. 27025154 PMC4824452

[22] Wu S, Zhang C, Cao R, Xu D, Guo H, J. Phys. Chem. B 2011, 115, 10360–10367, 10.1021/jp2046504. 21732684 PMC3162075

[23] Christianson DW, David PR, Lipscomb WN, Proc. Natl. Acad. Sci. USA 1987, 84, 1512–1515, 10.1073/pnas.84.6.1512. 3470737 PMC304464

[24] Kirby AJ, McDonald RS, Smith CR, J. Chem. Soc. Perkin Trans 2, 1495, 10.1039/p29740001495.

[25] I VK, Yu Ishchenko A, Hovtvianitsa A, Stepanenko V, Kharchenko S, A DB, A JK, Molecules 2019, 24. 30764512 10.3390/molecules24030572 PMC6384577

[26] Choudhary A, Gandla D, Krow GR, Raines RT, J. Am. Chem. Soc 2009, 131, 7244–7246, 10.1021/ja901188y. 19469574 PMC2811422

[27] Pollock SB, Kent SB, Chem. Commun 2011, 47, 2342–2344, 10.1039/C0CC04120C. 21173985

[28] Yang J, Kojasoy V, Porter GJ, Raines RT, ACS Cent. Sci 2024, 10, 1829–1834, 10.1021/acscentsci.4c00971. 39463835 PMC11503490

[29] Choudhary A, Fry CG, Kamer KJ, Raines RT, Chem. Commun 2013, 49, 8166, 10.1039/c3cc44573a. PMC3840071 23928794

[30] Dunitz JD, Taylor R, Chem. Eur. J 1997, 3, 89–98, 10.1002/chem.19970030115.

[31] Teng M, Jiang J, Ficarro SB, Seo HS, Bae JH, Donovan KA, Fischer ES, Zhang T, Dhe-Paganon S, Marto JA, Gray NS, ACS Med. Chem. Lett 2021, 12, 1302–1307, 10.1021/acsmedchemlett.1c00285. 34413960 PMC8366001

[32] Allen OKFH, Watson DG, Brammer L, Orpen AG, Taylor R, J. Chem. Soc., Perkin Trans 1987, 2, S1–S19, 10.1039/p298700000s1.

[33] Petrov V, Petrova V, Girichev GV, Oberhammer H, Giricheva NI, Ivanov S, J. Org. Chem 2006, 71, 2952–2956, 10.1021/jo0524270. 16599587

[34] Petrov VM, Girichev GV, Oberhammer H, Petrova VN, Giricheva NI, Bardina AV, Ivanov SN, J. Phys. Chem. A 2008, 112, 2969–2976, 10.1021/jp710532z. 18302350

[35] Sindi S, Choudhary S, Tefera M, Fung J, Karaj E, Chaudhary A, PCT/US2024/044175. U.S. Provisional Application 63/535,016, 2023.

[36] Favre HA, Powell WH, Applied PC, R Soc Chem Cambridge, England, 2014.

[37] Le Y, Gong W, Li B, Dunlop NM, Shen W, Su SB, Ye RD, Wang JM, J. Immunol 1999, 163, 6777–6784, 10.4049/jimmunol.163.12.6777. 10586077

[38] Lau J, Bloch P, Schäffer L, Pettersson I, Spetzler J, Kofoed J, Madsen K, Knudsen LB, McGuire J, Steensgaard DB, Strauss HM, Gram DX, Knudsen SM, Nielsen FS, Thygesen P, Reedtz-Runge S, Kruse T, J. Med. Chem 2015, 58, 7370–7380, 10.1021/acs.jmedchem.5b00726. 26308095

[39] Kozikowski AP, Nan F, Conti P, Zhang J, Ramadan E, Bzdega T, Wroblewska B, Neale JH, Pshenichkin S, Wroblewski JT, J. Med. Chem 2001, 44, 298–301, 10.1021/jm000406m. 11462970

[40] Mendel DB, Laird AD, Xin X, Louie SG, Christensen JG, Li G, Schreck RE, Abrams TJ, Ngai TJ, Lee LB, Murray LJ, Carver J, Chan E, Moss KG, Haznedar JO, Sukbuntherng J, Blake RA, Sun L, Tang C, Miller T, Shirazian S, McMahon G, Cherrington JM, Clin. Cancer Res 2003, 9, 327–337. 12538485

[41] Pan Z, Scheerens H, Li SJ, Schultz BE, Sprengeler PA, Burrill LC, Mendonca RV, Sweeney MD, Scott KC, Grothaus PG, Jeffery DA, Spoerke JM, Honigberg LA, Young PR, Dalrymple SA, Palmer JT, ChemMedChem 2007, 2, 58–61, 10.1002/cmdc.200600221. 17154430

[42] Wright SC, Motso A, Koutsilieri S, Beusch CM, Sabatier P, Berghella A, Blondel-Tepaz É, Mangenot K, Pittarokoilis I, Sismanoglou DC, Le Gouill C, Olsen JV, Zubarev RA, Lambert NA, Hauser AS, Bouvier M, Lauschke VM, Nat. Commun 2023, 14, 6243, 10.1038/s41467-023-41893-4. 37813859 PMC10562414

[43] Lee C, Han J, Jung Y, Exp. Mol. Med 2023, 55, 325–332, 10.1038/s12276-023-00941-1. 36750693 PMC9981720

[44] Shilleh AH, Viloria K, Broichhagen J, Campbell JE, Hodson DJ, Peptides 2024, 175, 171179, 10.1016/j.peptides.2024.171179. 38360354 PMC7618508

[45] Gabery S, Salinas CG, Paulsen SJ, Ahnfelt-Rønne J, Alanentalo T, Baquero AF, Buckley ST, Farkas E, Fekete C, Frederiksen KS, Helms HCC, Jeppesen JF, John LM, Pyke C, Nøhr J, Lu TT, Polex-Wolf J, Prevot V, Raun K, Simonsen L, Sun G, Szilvásy-Szabó A, Willenbrock H, Secher A, Knudsen LB, Hogendorf WFJ, JCI Insight 2020, 5, e133429. 32213703 10.1172/jci.insight.133429 PMC7213778

[46] Ast J, Arvaniti A, Fine NHF, Nasteska D, Ashford FB, Stamataki Z, Koszegi Z, Bacon A, Jones BJ, Lucey MA, Sasaki S, Brierley DI, Hastoy B, Tomas A, D’Agostino G, Reimann F, Lynn FC, Reissaus CA, Linnemann AK, D’Este E, Calebiro D, Trapp S, Johnsson K, Podewin T, Broichhagen J, Hodson DJ, Nat. Commun 2020, 11, 467, 10.1038/s41467-020-14309-w. 31980626 PMC6981144

[47] Kenner G, McDermott J, Sheppard R, J. Chem. Soc. D 1971, 636, 10.1039/c29710000636.

[48] Backes BJ, Virgilio AA, Ellman JA, J. Am. Chem. Soc 1996, 118, 3055–3056, 10.1021/ja9535165.

[49] Ueda T, Tamura T, Kawano M, Shiono K, Hobor F, Wilson AJ, Hamachi I, J. Am. Chem. Soc 2021, 143, 4766–4774, 10.1021/jacs.1c00703. 33733756

[50] Dubinsky L, Krom BP, Meijler MM, Bioorg. Med. Chem 2012, 20, 554–570, 10.1016/j.bmc.2011.06.066. 21778062

[51] Homan JDLRA, Woo CM, Niessen S, Jones LH, Parker CG, Nat. Rev. Methods Primers 2024, 4, 30, 10.1038/s43586-024-00308-4.

[52] West AV, Muncipinto G, Wu HY, Huang AC, Labenski MT, Jones LH, Woo CM, J. Am. Chem. Soc 2021, 143, 6691–6700, 10.1021/jacs.1c02509. 33876925 PMC11647638

[53] Tzakoniati F, Xu H, Li T, Garcia N, Kugel C, Payandeh J, Koth CM, Tate EW, Cell Chem. Biol 2020, 27, 306–313.e4.e304, 10.1016/j.chembiol.2019.10.011. 31732432 PMC7083225

[54] Frankovich TV, McCann HM, Hoffman KS, Rullo AF, Acs Central Sci. 2025, 11, 2006–2017, 10.1021/acscentsci.5c00699. PMC12550623 41142344

[55] Singh S, Tian W, Severance ZC, Chaudhary SK, Anokhina V, Mondal B, Pergu R, Singh P, Dhawa U, Singha S, Choudhary A, Chem. Soc. Rev 2023, 52, 5485–5515, 10.1039/D2CS00943A. 37477631

[56] Palecki J, Bhasin A, Bernstein A, Mille PJ, Tester WJ, Kelly WK, Zarrabi KK, Cancer Biol. Ther 2024, 25, 2356820, 10.1080/15384047.2024.2356820. 38801069 PMC11135853

[57] Los GV, Encell LP, McDougall MG, Hartzell DD, Karassina N, Zimprich C, Wood MG, Learish R, Ohana RF, Urh M, Simpson D, Mendez J, Zimmerman K, Otto P, Vidugiris G, Zhu J, Darzins A, Klaubert DH, Bulleit RF, Wood KV, ACS Chem. Biol 2008, 3, 373–382, 10.1021/cb800025k. 18533659

[58] Clackson T, Yang W, Rozamus LW, Hatada M, Amara JF, Rollins CT, Stevenson LF, Magari SR, Wood SA, Courage NL, Lu X, Cerasoli F Jr., Gilman M, Holt DA, Proc. Natl. Acad. Sci. USA 1998, 95, 10437–10442, 10.1073/pnas.95.18.10437. 9724721 PMC27912

[59] Nabet B, Roberts JM, Buckley DL, Paulk J, Dastjerdi S, Yang A, Leggett AL, Erb MA, Lawlor MA, Souza A, Scott TG, Vittori S, Perry JA, Qi J, Winter GE, Wong KK, Gray NS, Bradner JE, Nat. Chem. Biol 2018, 14, 431–441, 10.1038/s41589-018-0021-8. 29581585 PMC6295913

[60] Kipriyanov SM, Moldenhauer G, Martin AC, Kupriyanova OA, Little M, Protein Eng. 1997, 10, 445–453, 10.1093/protein/10.4.445. 9194170

[61] e Gall FL, Reusch U, Moldenhauer G, Little M, Kipriyanov SM, Immunol J. Methods 2004, 285, 111–127, 10.1016/j.jim.2003.11.007. 14871540

[62] Weaver JR, Good K, Walters RD, Kugel JF, Goodrich JA, Mol. Immunol 2007, 44, 2813–2819, 10.1016/j.molimm.2007.01.027. 17337059 PMC1924494

[63] Deng D, Saqcena C, Liu T, Paraiso K, Diehl P, Chenchik A, Immunother Cancer J 2024, 12, e009983, A1-A1683, 10.1136/jitc-2024-009983.

[64] Bhatt RS, Berjis A, Konge JC, Mahoney KM, Klee AN, Freeman SS, Chen C-H, Jegede OA, Catalano PJ, Pignon J-C, Cancer Immunol. Res 2021, 9, 156–169, 10.1158/2326-6066.CIR-20-0315. 33229411 PMC8284010

[65] Bates D, Abraham S, Campbell M, Zehbe I, Curiel L, PLoS One 2014, 9, e97220, 10.1371/journal.pone.0097220. 24819929 PMC4018298

[66] Zhang Z, Rohweder PJ, Ongpipattanakul C, Basu K, Bohn MF, Dugan EJ, Steri V, Hann B, Shokat KM, Craik CS, Cancer Cell 2022, 40, 1060–1069.e7.e1067, 10.1016/j.ccell.2022.07.005. 36099883 PMC10393267

[67] Serniuck NJ, Kapcan E, Moogk D, Moore AE, Lake BPM, Denisova G, Hammill JA, Bramson JL, Rullo AF, Mol. Ther. Oncol 2024, 32, 200842, 10.1016/j.omton.2024.200842. 39045028 PMC11264187

[68] Hattori T, Maso L, Araki KY, Koide A, Hayman J, Akkapeddi P, Bang I, Neel BG, Koide S, Cancer Discov. 2023, 13, 132–145, 10.1158/2159-8290.CD-22-1074. 36250888 PMC9827112

[69] Maso L, Rajak E, Bang I, Koide A, Hattori T, Neel BG, Koide S, Proc. Natl. Acad. Sci. USA 2024, 121, e2319029121, 10.1073/pnas.2319029121. 38781214 PMC11145297

[70] Pandey A, Rohweder PJ, Chan LM, Ongpipattanakul C, Chung DH, Paolella B, Quimby FM, Nguyen N, Verba KA, Evans MJ, Craik CS, Cancer Res. 2025, 85, 329–341, 10.1158/0008-5472.CAN-24-2450. 39656104 PMC11733532

[71] Chu Y, Lee S, Shah T, Yin C, Barth M, Miles RR, Ayello J, Morris E, Harrison L, Van de Ven C, Galardy P, Goldman SC, Lim MS, Hermiston M, McAllister-Lucas LM, Giulino-Roth L, Perkins SL, Cairo MS, Oncoimmunology 2019, 8, e1512455. 30546948 10.1080/2162402X.2018.1512455 PMC6287791

[72] Wang S, Mondal S, Zhao C, Berishaj M, Ghanakota P, Batlevi CL, Dogan A, Seshan VE, Abel R, Green MR, Younes A, Wendel HG, JCI Insight 2019, 4, e127566. 31217352 10.1172/jci.insight.127566 PMC6629124

[73] Angst D, Gessier F, Janser P, Vulpetti A, Wälchli R, Beerli C, Littlewood-Evans A, Dawson J, Nuesslein-Hildesheim B, Wieczorek G, Gutmann S, Scheufler C, Hinniger A, Zimmerlin A, Funhoff EG, Pulz R, Cenni B, J. Med. Chem 2020, 63, 5102–5118, 10.1021/acs.jmedchem.9b01916. 32083858

[74] Corso G, Stärk H, Jing B, Barzilay R, Jaakkola T, arXiv preprint arXiv:2210.01776 2022.

