# Supplementary material for: Lysine Targeting Group-Transfer Chimeras for Proximity Induction
Source: Angew Chem Int Ed Engl. Author manuscript; Available in PMC 2026 Jul 28. (PMC13410498; doi:10.1002/anie.202512131)
Supplement: Supplementary material [file NIHMS2190473-supplement-Supplementary_material.pdf]

Lysine Targeting Group-Transfer Chimeras for Proximity Induction

Sameek Singh,[1,7] Kien Tran,[1,3,7] Endri Karaj,[1,7] Basudeb Mondal,[1,7] Wenzhi Tian,[1,7] Surached Siriwongsup,[1,3] Shaimaa H. Sindi,[1] Uttam Dhawa,[1] Kaushik Pal,[1,6] Veronika M. Shoba,[1] Sunny Shi,[1] Anna Lian,[4,5] Jody Mou,[4,5] Myeonghoon Han,[1] Prashant Singh,[1] Nicholas F. Polizzi,[4,5] Amit Choudhary*,[1,2,3]

[1]Chemical Biology and Therapeutics Science, Broad Institute of MIT and Harvard, Cambridge, MA 02142, USA
[2]Department of Medicine, Harvard Medical School, Boston, MA 02115, USA
[3]Divisions of Renal Medicine and Engineering, Brigham and Women's Hospital, Boston, MA 02115, USA
[4]Department of Cancer Biology, Dana-Farber Cancer Institute, Boston, MA 02215, USA.
[5]Department of Biological Chemistry and Molecular Pharmacology, Harvard Medical School, Boston, MA 02215, USA.
[6]Department of Chemistry, Indian Institute of Technology Tirupati, Yerpedu-Venkatagiri Road, Yerpedu, AP-517619, India

[7]These authors contributed equally to this work. They will put their name first in their curriculum vitae citations or elsewhere.

*Correspondence: achoudhary@bwh.harvard.edu (A.C.)

*To whom correspondence should be addressed:

Amit Choudhary
Chemical Biology and Therapeutics Science
Broad Institute of MIT and Harvard
415 Main Street, Rm 3012
Cambridge, MA 02142
Phone: (617) 714-7445
Fax: (617) 715-8969
Email: achoudhary@bwh.harvard.edu

Table of Contents

1 Materials and methods. **5**

1.1 Materials and general procedures **5**

1.1.1 Plasmids 5

1.1.2 Proteins 5

1.1.3 Reagents and Cells 6

1.1.4 Chemistry 6

1.2 Bioinformatic analysis **7**

1.2.1 Calculation of geodesic or Euclidean distances 7

1.3 Reactivity and the stability assay **7**

1.3.1 Hydrolytic Stability of GRC Fragments 7

1.3.2 Lysine Reactivity of GRC Fragments 8

1.3.3 Stability of NASA probes 8

1.4 Labeling and mass spectrometry **8**

1.5 T-cell activation assay **12**

1.6 X-ray crystallography **12**

2 Supporting Figures **13**

3 Synthesis and Characterization **62**

3.1 Synthesis of the NASA probes **62**

3.1.1 Compound **1** (RIPK1 NASA probe) 62

3.1.2 Compound **2** (MEK2 NASA probe) 63

3.1.3 Compound **3** (IRE1α NASA probe) 64

3.1.4 Compound **4** (MEK2 NASA probe) 70

3.1.5 Compound **5** (LIMK1 NASA probe) 73

3.1.6 Compound **6** (EGFR NASA probe) 78

3.2 Synthesis of the *N*-substituted *N*-acyl sulfonamide library **80**

3.2.1 *N*-(2-oxopropyl)-*N*-(phenylsulfonyl)acetamide (compound **7**) 80

3.2.2 *N*-(1-cyanoethyl)-*N*-(phenylsulfonyl)acetamide (compound **8**) 81

3.2.3 *N*-(Cyanomethyl)-*N*-(phenylsulfonyl)acetamide (compound **9**) 81

3.2.4 *N*-(2-amino-2-oxoethyl)-*N*-(phenylsulfonyl)acetamide (compound **10**) 82

3.2.5 *N*-(Phenylsulfonyl)-*N*-(2,2,2-trifluoroethyl)acetamide (compound **11**) 82

3.2.6 *N*-(2,2,3,3,3-pentafluoropropyl)-*N*-(phenylsulfonyl)acetamide (compound **12**) 82

3.2.7 *N*-(benzylsulfonyl)-*N*-(2,2,2-trifluoroethyl)acetamide (compound **13**) 83

3.2.8 *N*-(phenethylsulfonyl)-*N*-(2,2,2-trifluoroethyl)acetamide (compound **14**) 83

3.2.9 Methyl *N*-acetyl-*N*-(phenylsulfonyl)glycinate (**4S1**) 83

3.2.10 *N*-(2,2,3,3,3-pentafluoropropyl)-*N*-(phenethylsulfonyl)acetamide (**4S3**) 84

3.3 Synthesis of the SuFA linker S15 **84**

3.4 Synthesis of compound 17 (FPR SuFA probe) **86**

3.5 Synthesis of compound 19 (GLP1R SuFA probe) **89**

3.6 **Synthesis of compound 21 (PSMA SuFA probe)** 92
3.7 **Synthesis of compound 23 (BTK SuFA probe)** 94
3.8 **Synthesis of compound 26 (VEGFR2 SuFA probe)** 95
3.9 **Synthesis of compound 27 (Halo-PSMA-GRC)** 97
3.10 **Synthesis of compound 28 (Halo-PSMA-iGRC)** 99
3.11 **Synthesis of compound 29 (FKBP-PSMA-GRC)** 99
3.12 **Synthesis of compound 30 (FKBP-PSMA-iGRC)** 100
3.13 **Synthesis of compound 31 (Halo-BTK-GRC)** 102
3.14 **Synthesis of compound 32 (Halo-BTK-iGRC)** 103
3.15 **Synthesis of compound S1, S2 and S3 (GLP1R SuFA probe)** 104
3.16 **Synthesis of compound S4 and S5 (GLP1R SuFA probe)** 106
3.17 **Synthesis of compound S6 (RIPK1 SuFA probe)** 106
3.18 **Synthesis of compound S7 (VEGFR2 NASA probe)** 107
3.19 **X-ray data for compound 9 and 11** 107
3.20 **LC-MS traces of ligands in manuscript** 113
3.20.1 Compound **1** (RIPK1 NASA probe) 113
3.20.2 Compound **2** (MEK2 NASA probe) 116
3.20.3 Compound **3** (IRE1α NASA probe) 119
3.20.4 Compound **4** (MEK2 NASA probe) 120
3.20.5 Compound **6** (EGFR NASA probe) 123
3.20.6 Compound **17** (FPR SuFA probe) 125
3.20.7 Compound **19** (GLP1R SuFA probe) 126
3.20.8 Compound **23** (BTK SuFA probe) 127
3.20.9 Compound **26** (VEGFR2 SuFA probe) 128
3.20.10 Compound **27** (Halo-PSMA-GRC) 129
3.20.11 Compound **28** (Halo-PSMA-iGRC) 130
3.20.12 Compound **29** (FKBP-PSMA-GRC) 131
3.20.13 Compound **30** (FKBP-PSMA-iGRC) 132
3.20.14 Compound **31** (Halo-BTK-GRC) 133
3.20.15 Compound **32** (Halo-BTK-iGRC) 134
3.20.16 Compound **S1** (GLP1R SuFA probe) 135
3.20.17 Compound **S2** (GLP1R SuFA probe) 136
3.20.18 Compound **S3** (GLP1R SuFA probe) 137
3.20.19 Compound **S4** (GLP1R SuFA probe) 138
3.20.20 Compound **S5** (GLP1R SuFA probe) 139
3.20.21 Compound **S6** (RIPK1 SuFA probe) 140
3.20.22 Compound **S7** (VEGFR2 NASA probe) 141
3.21 **HRMS spectra of ligands in manuscript** 142
3.21.1 Compound **17** (FPR SuFA probe) 142
3.21.2 Compound **19** (GLP1R SuFA probe) 143

3.21.3 Compound **21** (PSMA SuFA probe) 144
3.21.4 Compound **23** (BTK SuFA probe) 145
3.21.5 Compound **26** (VEGFR2 SuFA probe) 146
3.21.6 Compound **27** (Halo-PSMA-GRC) 147
3.21.7 Compound **28** (Halo-PSMA-iGRC) 148
3.21.8 Compound **29** (FKBP-PSMA-GRC) 149
3.21.9 Compound **30** (FKBP-PSMA-iGRC) 150
3.21.10 Compound **31** (Halo-BTK-GRC) 151
3.21.11 Compound **32** (Halo-BTK-iGRC) 152
3.21.12 Compound **S6** (RIPK1 SuFA probe) 153
3.21.13 Compound **S7** (VEGFR2 NASA probe) 154

3.22 NMR spectra 155
3.22.1 Compound **1** (RIPK1 NASA probe) 155
3.22.2 Compound **2** (MEK2 NASA probe) 156
3.22.3 Compound **3** (IRE1α NASA probe) 157
3.22.4 Compound **4** (MEK2 NASA probe) 158
3.22.5 Compound **5** (LIMK1 NASA probe) 160
3.22.6 Compound **6** (EGFR NASA probe) 162
3.22.7 *N*-substituted *N*-acyl sulfonamide library **7-14** 166
3.22.8 SuFA linker **S15** 181
3.22.9 Compound **17** (FPR2 SuFA probe) 185
3.22.10 Compound **21** (PSMA SuFA probe) 194
3.22.11 Compound **23** (BTK SuFA probe) 195
3.22.12 Compound **26** (VEGFR2 SuFA probe) 197
3.22.13 Compound **27** (Halo-PSMA-GRC) 201
3.22.14 Compound **28** (Halo-PSMA-iGRC) 203
3.22.15 Compound **29** (FKBP-PSMA-GRC) 204
3.22.16 Compound **30** (FKBP-PSMA-iGRC) 210
3.22.17 Compound **31** (Halo-BTK-GRC) 213
3.22.18 Compound **32** (Halo-BTK-iGRC) 216
3.22.19 Compound **S6** (RIPK1 SuFA probe) 217
3.22.20 Compound **S7** (VEGFR2 NASA probe) 219

4 References 220

1 Materials and methods.

1.1 Materials and general procedures.

1.1.1 Plasmids

The plasmids - Halo-3_G4S_antiCD3-scFv-His_pcDNA3.1 and FKBPF36V-3_G4S_antiCD3-scFv-His_pcDNA3.1(+) were generated by GenScript. N-3HA-tagged FPR2 was purchased from the cDNA Resource Center (www.cdna.org). N-HA-tagged PSMA (HG15877-NY), N-FLAG-tagged VEGFR2 (HG10012-NF), and C-FLAG-tagged BTK (HG10578-CF) plasmid were purchased from Sino Biological Inc.

The N-3HA-GLP1R plasmid was constructed by inserting a synthetic DNA fragment (GLP1R-gBlock, sequence below) into the Addgene vector-66295. The vector was linearized by restriction digestion with NheI and PaqCI, and the insertion was performed via NEBuilder HiFi DNA Assembly from New England Biolabs (NEB) following the manufacturer's protocol.
GLP1R gBlock sequence -
TATAGGGAGACCCAAGCTGGCTAGCGTTTAAACTTAAGCTTGGTACCGAGCTCGGATCCACTAGTCCAGTG
TGGTGGAATTCTGCAGATATCCAGCACAGTGGCGGCCGCGCCACCATGAAGACGATCATCGCCCTGAGCT
ACATCTTCTGCCTGGTATTCGCCTATCCTTACGATGTGCCAGATTATGCTTACCCGTACGACGTTCCGGACT
ACGCTTACCCATACGATGTACCAGACTATGCCATGGCAGGTGCCC

1.1.2 Proteins

Following Universal T cell Engager (UniTE) proteins were produced by GenScript-
Halo-3(G4S)antiCD3-scFv-His
Sequence:
MEGWSCIILFLVATATGVHSEIGTGFPFDPHYVEVLGERMHYVDVGPRDGTPVLFLHGNPTSSYVWRNIIPHVA
PTHRCIAPDLIGMGKSDKPDLGYFFDDHVRFMDAFIEALGLEEVVLVIHDWGSALGFHWAKRNPERVKGIAFME
FIRPIPTWDEWPEFARETFQAFRTTDVGRKLIIDQNVFIEGTLPMGVVRPLTEVEMDHYREPFLNPVDREPLWR
FPNELPIAGEPANIVALVEEYMDWLHQSPVPKLLFWGTPGVLIPPAEAARLAKSLPNCKAVDIGPGLNLLQEDNP
DLIGSEIARWLSTLEISGGGGGSGGGGSGGGGSDIKLQQSGAELARPGASVKMSCKTSGYTFTRYTMHWVKQ
RPGQGLEWIGYINPSRGYTNYNQKFKDKATLTTDKSSSTAYMQLSSLTSEDSAVYYCARYYDDHYCLDYWGQ
GTTLTVSSVEGGSGGSGGSGGSGGVDDIQLTQSPAIMSASPGEKVTMTCRASSSVSYMNWYQQKSGTSPKT
WIYDTSKVASGVPYRFSGSGSGTSYSLTISSMEAEDAATYYCQQWSSNPLTFGAGTKLELKGSGSGHHHHHH

FKBPF36V-3(G4S)antiCD3-scFv-His
Sequence:

MEGWSCIILFLVATATGVHSGVQVETISPGDGRTFPKRGQTCVVHYTGMLEDGKKVDSSRDRNKPFKFMLGK
QEVIRGWEEGVAQMSVGQRAKLTISPDYAYGATGHPGIIPPHATLVFDVELLKLEGGGGSGGGGSGGGGSDI
KLQQSGAELARPGASVKMSCKTSGYTFTRYTMHWVKQRPGQGLEWIGYINPSRGYTNYNQKFKDKATLTTDK
SSSTAYMQLSSLTSEDSAVYYCARYYDDHYCLDYWGQGTTLTVSSVEGGSGGSGGSGGSGGVDDIQLTQSP
AIMSASPGEKVTMTCRASSSVSYMNWYQQKSGTSPKTWIYDTSKVASGVPYRFSGSGSGTSYSLTISSMEAE
DAATYYCQQWSSNPLTFGAGTKLELKGSGSGHHHHHH

Protein production was carried out in a mammalian expression system, and purification was performed using a HisTrap™ FF Crude column followed by size-exclusion chromatography on a HiLoad™ 26/600 Superdex 200 pg column (320 mL). The purity of the proteins was assessed by SEC-HPLC and was ≥98%. RIP1 kinase protein (ab271732) and LIM kinase 1 protein (ab174066) were purchased from Abcam. MEK2 protein (10678-H09B), IRE1 protein (11905-H20B), EGFR protein (10001-H20B2), and VEGFR2 protein (10012-H20B1) were purchased from Sino Biological Inc.

1.1.3 Reagents and Cells

TransIT®-LT1 transfection reagent was purchased from Mirus (MIR 2304). cOmplete™, Mini, EDTA-free Protease Inhibitor Cocktail (Cat#04693159001) and Inhibitor, Phosphatase, PhosSTOP (Cat#4906837001) were purchased from Millipore Sigma. M-PER™ Mammalian Protein Extraction Reagent (Cat#78501), Pierce™ BCA Protein Assay Kit (Cat#23225), Pierce™ Anti-HA Magnetic Beads (88837) were purchased from Invitrogen. Opti-MEM I (cat# 11058-021) was purchased from Life Technologies. ChromoTek DYKDDDDK Fab-Trap® Agarose was purchased from Proteintech. Polymerases, restriction enzymes, and NEBuilder HiFi DNA Assembly mix were purchased from New England Biolabs. Primers and Synthetic DNA fragments (gBlocks) were purchased from GENEWIZ and Integrated DNA Technologies respectively.

TCEP Solution (77720), TEAB buffer (90114), and Pierce™ GPCR Extraction and Stabilization Reagent (A43436) were purchased from ThermoFisher. Iodoacetamide (I0044) was purchased from Tokyo Chemical Industry Co., Ltd. S-Trap micro columns (CO2-micro-80) were purchased from Protifi. Trypsin (VA9000), Chymotrypsin (V1062), Trypsin/Lys-C Mix (V5071), and Bright-Glo™ Luciferase Assay System (E2610) were purchased from Proemga. Jurkat cells (IL2-Luciferase reporter cell line, 60481) were purchased from BPS Bioscience. Raji, HEK293T, DU145, and LNCaP cells were purchased from ATCC.

1.1.4 Chemistry

Rink Amide (loading 0.3 – 0.6 mmol/g) was purchased from Chem-Impex (Wood Dale, IL), Fmoc-Gly-Wang ProTide resin (loading 0.22 mmol/g) were obtained from CEM (Matthews, NC). Piperidine, 4-(Dimethylamino)pyridine (DMAP) were acquired from Sigma-Aldrich (St. Louis, MO), trifluoroacetic acid and triisoproylsilane from Oakwood Chemical (Estill, SC). Amino acids, HATU, diisopropylethylamine (DIPEA), and other chemicals were purchased from Combi-Blocks (San Diego, CA). *N*, *N*-dimethylformamide (DMF),

dichloromethane (DCM), ethyl acetate (EtOAc) and other solvents were purchased from Fischer Scientific (Waltham, MA), and NMR solvents were purchased from Cambridge Isotope Laboratories, Inc (Tewksbury, MA). All chemicals were used as received. Purification of the building blocks was performed via silica gel column chromatography on Teledyne Isco CombiFlash Rf system (Lincoln, NE). Analytical HPLC-MS was performed on an Acquity UPLC system (Water, Milford, MA) equipped with Acquity BEH C18 column 2.1 × 50 mm, particle size 1.7 µm, pore 130 Å. Purification of final compounds was carried out using the preparative HPLC ACCQPrep® HP150 system equipped with XBridge Prep C18 column, 19 × 250 mm, 5 µm particle size, pore 130 Å. High resolution mass spectrometry (HRMS) was acquired with Agilent 6210/6220 ESI-TOF. ^{1}H and ^{13}C NMR spectra (298 K) were recorded at 400 MHz and 100 MHz respectively on Bruker Ascend 400 (Billerica, MA). Chemical shifts are reported as parts per million (ppm) and residual solvent signals were used as reference.

1.2 Bioinformatic analysis

1.2.1 Calculation of geodesic or Euclidean distances

The protein structures analyzed in this study were obtained from the refined set of the PDBbind database.[1] Solvent-excluded surfaces were generated for each protein structure using Pymol and downloaded as STL surface representations. The edges and vertices of the triangles in the STL mesh were processed using PyMeshFix[2] and read using Potpourri3d.[3] Surface path distances or geodesic distance were computed from the nearest solvent-exposed ligand atom, which had a solvent-accessible surface area greater than 10 Å², to the target residue atom. To calculate the shortest surface path distance over the surface graph, we took the mesh vertices closest to the ligand atom and the target atom using the Potpourri3d compute_distance method and adjusted for accuracy by adding mesh to atom distance. Distances were evaluated to all lysine CB, lysine NZ, cysteine CB, and cysteine SG atoms present in the protein structures. The straight-line distance, or Euclidean distance between the starting ligand atom coordinates and the target residue atom coordinates, was also calculated. The shortest paths depicted in Figure 1 were identified using the find_geodesic_path method from Potpourri3d and visualized in Pymol. All shown paths correspond to the nearest lysine or cysteine residues based on surface path distance, excluding cysteine residues involved in disulfide bonding.

1.3 Reactivity and the stability assay

1.3.1 Hydrolytic stability of GRC fragments

To determine the hydrolytic stability of GRC fragments, the compounds were diluted to 1 mM (from 10 mM DMSO stock) in PBS (0.5 mL, pH 7.4) and incubated at 37 °C. N-(4-methoxyphenyl)acetamide (100 µM) was included as an internal standard. The mixtures were immediately analyzed by LC-MS, and the disappearance of the starting material was monitored by integration of the corresponding AUC. Half-lives ($t_{1/2}$) were calculated assuming first-order kinetics.

1.3.2 Lysine reactivity of GRC fragments

The intrinsic lysine reactivity of the fragments was assessed by diluting GRC fragments to 100 µM (from a 10 mM DMSO stock) in anhydrous MeCN (0.5 mL) at room temperature. N-(4-methoxyphenyl)acetamide (100 µM) was added as an internal standard. N-Boc-Lysine-Coumarin (100 µM, 1 equiv.) was then added, and the reactions were incubated at 37 °C. Reaction mixtures were analyzed at regular intervals by LC-MS. Formation of the acylated product was monitored by integration of the AUC. Half-lives ($t_{1/2}$) and reaction rate constants were calculated assuming second-order kinetics. Note: Reactions were performed in anhydrous MeCN to avoid competing hydrolysis.

1.3.3 Stability of NASA probes

The stability of NASA-GRCs in the presence of a lysine nucleophile was determined by diluting the compounds to 100 µM (from 10 mM stock) in MeCN/PBS (20% MeCN, 0.5 mL, pH 7.4) at room temperature. N-(4-methoxyphenyl)acetamide (100 µM) was included as an internal standard. An initial LC-MS measurement was taken (t_0) to serve as reference. N-Boc-Lysine-Coumarin (1 mM, 10 equiv.) was added, and the samples were incubated at 37 °C. At defined time points, samples were analyzed by LC-MS, and the AUC of the starting material was normalized to the internal standard. Ratios were expressed as percentages relative to t_0 (100% = starting material; 0% = fully consumed). Half-lives ($t_{1/2}$) were calculated assuming pseudo-first-order kinetics. Note: Disappearance of the starting material results from both hydrolysis and acyl-transfer reactions.

1.4 Labeling and mass spectrometry

1.4.1 In-gel fluorescence

In-gel fluorescence: Click-chemistry labeling was performed in a 30 µL reaction by adding 1.5 µL each of 800 µM sulfoCy5.5-azide (BroadPharm, BP-22483), 60 mM THPTA (Lumiprobe, H4050), 60 mM $CuSO_4$, and 74 mM sodium ascorbate to a mixture containing 1 µM protein and 5 µM NASA probe in PBS. After a 30 min incubation at room temperature, reactions were quenched with 10 µL of 4× Laemmli reducing buffer (bioWorld, 10570020) and heated to 100 °C for 5 min. Following cooling, 20 µL of each sample was resolved on 4–12% Bis-Tris gels (ThermoFisher, NP0321BOX) in MOPS buffer (ThermoFisher, NP0001) at 150 V for 1 h, and imaged on an Azure c600 system at 700 nm (NIR).

1.4.2 Enzymatic digestion of NASA labeled proteins

Proteins (2 µM) were incubated with probes (at the indicated concentrations) in PBS at ambient temperature for 1h. The protein digestion protocol was adapted from Protifi using S-Trap™ micro columns. The labeled protein was denatured by adding 10 µL of 9% SDS in 100 mM TEAB buffer, followed by reduction with TCEP and alkylation with iodoacetamide to prevent disulfide bond formation. After acidifying with 27.5% phosphoric acid, 165 µL of 100 mM TEAB in methanol was added, and the mixture was loaded onto the S-Trap and spun at 4,000 g for 30 s. The column was washed five times with 100 mM TEAB in methanol, then digested overnight at 37 °C

with 25 μL of trypsin (1:10–1:15 w/w). Peptides were eluted by sequentially adding 40 μL of three different buffers—50 mM TEAB, 0.2% formic acid in 50% acetonitrile, and 95% acetonitrile, with centrifugation at 4,000 g for 1 minute after each buffer. The eluted peptides were dried in a speed vacuum concentrator and stored at -80°C for further analysis.

1.4.3 Enzymatic digestion of NASA labeled proteins expressed in HEK293T cells

HEK293T cells (25.0×10^6 cells) were seeded on a 15 cm dish and cultured in DMEM supplemented with 10% FBS, penicillin (100 units/mL) and streptomycin (100 μg/mL) for 12 hours at 37 ºC in a 5% CO2 humidified atmosphere. Respective plasmids - N-3HA-FPR2, N-3HA-GLP1R, N-HA-PSMA or C-FLAG-tagged BTK were transfected into HEK293T cells using TransIT®-LT1 transfection reagent and OPTI-MEM (25 μg of each DNA in 3000 μL OPTI-MEM with 90 μL of trans-IT per dish), and cells were maintained for another 24 hours. Then cells were treated with 1 μM concentration of respective GRCs for 4 h at 37 ºC in DMEM media with the serum. After 5 hours of incubation, cells were washed with cold PBS and lysed on ice using the cold lysis buffer (Pierce™ GPCR Extraction and Stabilization Reagent, EDTA-free Protease Inhibitor Cocktail (cOmplete) and PhosSTOP) following manufacturer's protocol. Pierce BCA Protein Assay Kit was used to determine the protein concentration of cell lysates, and immunoprecipitation (IP) was performed using the volume of cell lysate that contains 2-3 milligram of protein for each IP sample. Anti-HA magnetic beads or ChromoTek DYKDDDDK Fab-Trap® Agarose were used, and the immunoprecipitation protocol from Thermo Scientific Pierce or Proteintech was followed. Approximately, 50-150 μL of beads were used for each sample, and beads were washed with lysis buffer 2× times before incubation with cell lysates. A volume of cell lysate containing approximately 2-3 milligram of proteins was incubated with the beads at 4 ºC overnight on a rotating shaker. The next day, beads were washed 3× times with TBST (0.05% Tween-20) buffer and one time with water before elution of proteins. For eluting the bound proteins from the beads 30 μL 5% SDS was used and samples heated at 95 °C for 10 minutes. Subsequently, the eluted proteins were cleaned and digested with protocol adapted from Protifi using S-Trap micro columns (cat. CO2-micro-80). Trypsin, Chymotrypsin or Trypsin/Lys-C Mix at a 1:10-15 weight to weight ratio was added to the S-trap. The digestion proceeded at 37 °C overnight. The digested peptides were eluted using sequential addition of 40 μL of following buffers to the top of the trap and centrifugation at 4,000g for 1 min each: 50 mM TEAB buffer, 0.2% formic acid in 50% ACN in water, and finally 95% ACN. The eluted peptides were dried in a speed vacuum concentrator and stored at -80°C for further analysis.

1.4.4 LC-MS/MS analysis of digested proteins

Prior to LC-MS/MS analysis, dried samples were reconstituted in minimal 2% ACN, 0.2% formic acid solution, sonicated for 3 minutes, and centrifuged at 20,000g for 1 minute. The supernatant was then transferred to LCMS-vials (Thermo Fisher, cat. 6PK1655), which were subsequently loaded onto a Vanquish Neo UHPLC system (ThermoFisher) coupled to an Orbitrap Eclipse mass spectrometer (ThermoFisher). Peptides were separated on a 150 mm column (Waters, cat. 186009259) using a 130-minute gradient (3.5-123.5 min, 2%-35% ACN, 123.5-

124 min 35%-60% ACN, 124-127 min 60% ACN, 127-130 min wash) at a flow rate of 4 μL/min or some minor variations of this method. MS1 data were acquired in Orbitrap mode with a resolution of 120,000, standard AGC target, and auto maximum injection time. Charge states from 2+ to 7+ were included, and a dynamic exclusion time of 30 seconds was used. MS2 scans were isolated with the quadrupole and fragmented using HCD with a fixed collision energy of 30% and a 1.6 m/z isolation window. The normalized AGC target for MS2 was set to 250%, and fragment ions were detected in the Orbitrap at a resolution of 15,000 with a defined first mass of m/z 100. Minor variations were applied across both MS1 and MS2 parameters based on specific experiments.

1.4.5 LC-MS/MS data processing and analysis

Proteome Discoverer 2.5 (Thermo Fisher) was used to process the raw data, utilizing Sequest HT for identification against the human EGFR (Uniprot ID: P00533), human FPR2 (Uniprot ID: P25090), human GLP1R (Uniprot ID: P43220), human IRE1 (Uniprot ID: O75460), human LIMK1 (Uniprot ID: P53667) , human MEK2 (Uniprot ID: P36507), human PSMA (Uniprot ID: Q04609), human RIPK1 (Uniprot ID: Q13546), and human VEGFR2 (Uniprot ID: P35968) proteins. The search criteria permitted up to two missed cleavages and included peptides with at least 6 amino acids. Precursor mass tolerance was set at 10 ppm, and fragment mass tolerance was fixed at 0.01 Da. The dynamic modifications accounted for methionine oxidation and the addition of the NASA warhead (94.042 Da), with N-terminal glutamate cyclization considered only for peptides. Acetylation at protein termini was also allowed, while cysteine alkylation was treated as a static modification. Peptide-spectrum match (PSM) validation was performed using the Target Decoy PSM Validator with a strict false discovery rate (FDR) of 0.01. The labeling percentage was determined by dividing the abundance of the modified peptide by the combined abundance of both modified and unmodified peptides.

1.4.6. Competition studies

Purified protein (8 μL, 800 nM) was first pre-incubated with inhibitor (1 μL, 1 μM final concentration) in PBS, pH 7.4 at room temperature for 30 min. Subsequently, the corresponding SuFA or NASA probe (1 μL, 100 nM final concentration) was added, and the reaction was further incubated for 1 h at room temperature. Then, the copper-catalyzed click reaction was performed by the addition of $CuSO_4$ (1 μL, 1 mM final concentration), THPTA (1 μL, 5 mM final concentration), sulfo-Cy5-azide (Lumiprobe, cat# B3330; 0.5 μL, 40 μM final concentration), and sodium ascorbate (1 μL, 5 mM final concentration). The reaction mixtures were incubated at room temperature for 1 h in the dark after which 3 μL of 6× Laemmli SDS reducing buffer (Thermofisher) was added to the samples and boiled (95 °C, 10 min). After cooling to room temperature, 10 μL of the sample was resolved on 4–12% Bis-Tris gels (ThermoFisher, cat# NP0321BOX) in 1× MOPS buffer (ThermoFisher, cat# NP0001) at 170 V for 1 h. In-gel fluorescence was measured using the LiCor Odyssey CLx Imaging System. After imaging, gels were stained in InstantBlue® Coomassie Protein Stain (abcam, cat#ab119211) or silver stain (Thermofisher, cat# 24612).

1.4.7. In-gel fluorescence of cells labeled by PSMA SuFA probe **21**

LnCAP cells (ATCC) were plated at a density of 1 × 10^6 cells per well in media (2 mL, RPMI 1640 media supplemented with 10% fetal bovine serum, penicillin (100 units/mL), and streptomycin (0.1 mg/mL) in a 6-well plate. Then, cells were treated with either DMSO vehicle or PSMA SuFA probe **21** (100 nM final concentration). For competition, cells were first pre-treated with PSMA ligand **21** (1 μM final concentration) for 30 min, before addition of SuFA probe **21** (100 nM). After 5 h incubation at 37 °C, the cells were pelleted, washed once with PBS, pH 7.4, and lysed in cold M-PER lysis buffer containing Halt™ protease/phosphatase inhibitor cocktail (Thermofisher, cat# 78440). The lysates were centrifuged (17000×g, 10 min, 4 °C) and the protein concentration of the supernatant was determined by BCA assay following the manufacturer's protocol. The copper-catalyzed click reaction was performed by taking lysate (45 μL, at 2 mg/mL) and adding $CuSO_4$ (1 μL, 50 mM in water; 1 mM final concentration), THPTA (2.5 μL, 100 mM in water; 5 mM final concentration), sulfo-Cy5-azide (0.5 μL, 4 mM in DMSO; 40 μM final concentration), and lastly, sodium ascorbate (1 μL, 250 mM in water; 5 mM final concentration). The reaction mixtures were incubated at room temperature for 1 h in the dark after which 10 μL of 6× Laemmli SDS reducing buffer was added to the samples and boiled (95 °C, 10 min). After cooling to room temperature, 10 μL of the sample was resolved on 4–12% Bis-Tris gels in 1× MOPS buffer at 170 V for 1 h. In-gel fluorescence was measured using the LiCor Odyssey CLx Imaging System. After imaging, the gel was stained in InstantBlue® Coomassie Protein Stain (abcam, cat#ab119211).

1.4.8. Time-course and kinetic studies

Purified VEGFR2 protein (8 μL, 500 nM) was incubated the corresponding SuFA **26** or NASA probe **S7** (2 μL, 10 μM final concentration). After each desired time point, the reaction was quenched by adding 16.6% (wt% in H_2O) hydroxylamine solution (1 μL) followed by sunitinib (0.5 μL, 500 μM final concentration). After 5 min, the reaction tubes were then snap-frozen in liquid nitrogen and stored at –80 °C until completion of the last time point. After all time points have been completed, all samples were brought to room temperature. Then, copper-catalyzed click chemistry was performed by the addition of $CuSO_4$ (1 μL, 1 mM final concentration), THTPA (1 μL, 5 mM final concentration), sulfo-Cy5-azide (0.5 μL, 40 μM final concentration), and sodium ascorbate (1 μL, 5 mM final concentration). The reaction mixtures were incubated at room temperature for 1 h in the dark after which 3 μL of 6× Laemmli SDS reducing buffer was added to the samples and boiled (95 °C, 10 min). After cooling to room temperature, 10 μL of the sample was resolved on 4–12% Bis-Tris gels in 1× MOPS buffer at 170 V for 1 h. In-gel fluorescence was measured using the LiCor Odyssey CLx Imaging System.

Band intensity from the in-gel fluorescence was measured using ImageJ. The data were then used to generate reaction curves which were analyzed using a first-order association fit (GraphPad Prism). The extracted k_{obs} was taken to be equivalent to k_{inact}.

1.5 T-cell activation assay

For this experiment, reporter Jurkat cells were cultured in RPMI 1640 media containing 10% FBS, penicillin (100 units/mL) and streptomycin (100 µg/mL) and 1 mg/ml of Geneticin. HEK293T cells were grown in DMEM media supplemented with 10% FBS, penicillin (100 units/mL) and streptomycin (100 µg/mL). DU145 cells were cultured in EMEM media supplemented with 10% FBS, penicillin (100 units/mL) and streptomycin (100 µg/mL). Raji and LNCaP cells were cultured in RPMI 1640 media supplemented with 10% FBS, penicillin (100 units/mL) and streptomycin (100 µg/mL).

Adherent or suspension target cells (0.2×10^6 cells/well or 0.4×10^6 cells/well, respectively) were seeded in 100 µL of RPMI or DMEM media in a 96-well plate and incubated for 24 or 12 h respectively. On the following day, cells were treated with 0.1–10 µM of the indicated compounds or DMSO as a control in 100 µL for 5 h. After treatment, the cells were gently washed/centrifuged three times with 100 µL of complete media and cocultured with 0.06×10^6 or 0.1×10^6 Jurkat IL2-Luciferase reporter cells in the presence of UniTEs (2 µM) for 6-12 h, using either "Halo-3G4S-aCD3-ScFV" for HaloTag-based GRCs or "FKBP-3G4S-aCD3-ScFV" for FKBP-based GRCs. Bright-Glo luciferase assay reagent was then added to each well, incubated for 5 min, and luminescence was measured. Data were normalized to control wells and the fold changes in relative luminescence units (RLUs) were reported as T cell activation.

1.6 X-ray crystallography

A crystal mounted on a diffractometer was collected data at 100 K. The intensities of the reflections were collected by means of a Bruker D8 Venture diffractometer ($Mo_{K\alpha}$ radiation, λ=0.71073 Å), and equipped with an Oxford Cryosystems nitrogen flow apparatus. The collection method involved 0.5° scans in ω at 11° in 2θ. Data integration down to 0.77 Å resolution was carried out using SAINT V8.40A[1] with reflection spot size optimization. Absorption corrections were made with the program SADABS[4]. The structure was solved by the Intrinsic Phasing methods and refined by least-squares methods again F^2 using SHELXT-2018[5] and SHELXL-2018[6] with OLEX 2[7] interface. Non-hydrogen atoms were refined anisotropically, and hydrogen atoms were allowed to ride on the respective atoms. Crystal data as well as details of data collection and refinement are summarized in Table S1 and S3, and geometric parameters are shown in Table S2 and S4. The Ortep plots produced with SHELXL-2018 program, and the three-dimensional supramolecular architecture drawing was produced with Accelrys DS Visualizer 2.06[8]. We thank the support to the X-ray facility from the Major Research Instrumentation (MRI) Program of the National Science Foundation (NSF) under Award Numbers 2216066.

2 Supporting Figures

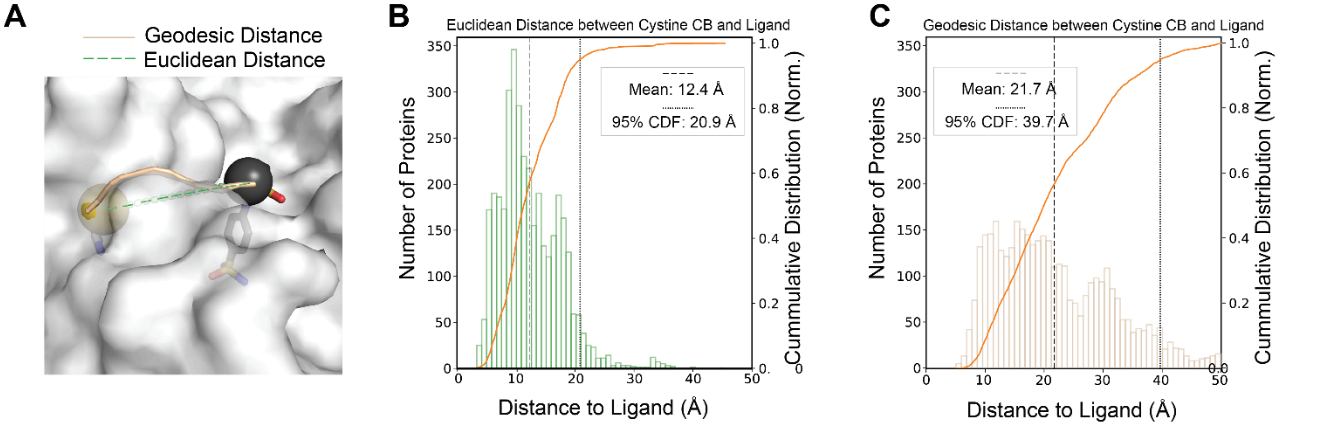

Figure S1. (A) Cartoon representation of geodesic or Euclidean distances from the ligand to Cysteine. **(B)** Distribution of Euclidean distance from the ligand to cysteine. **(C)** Distribution of the geodesic distance from the ligand to cysteine.

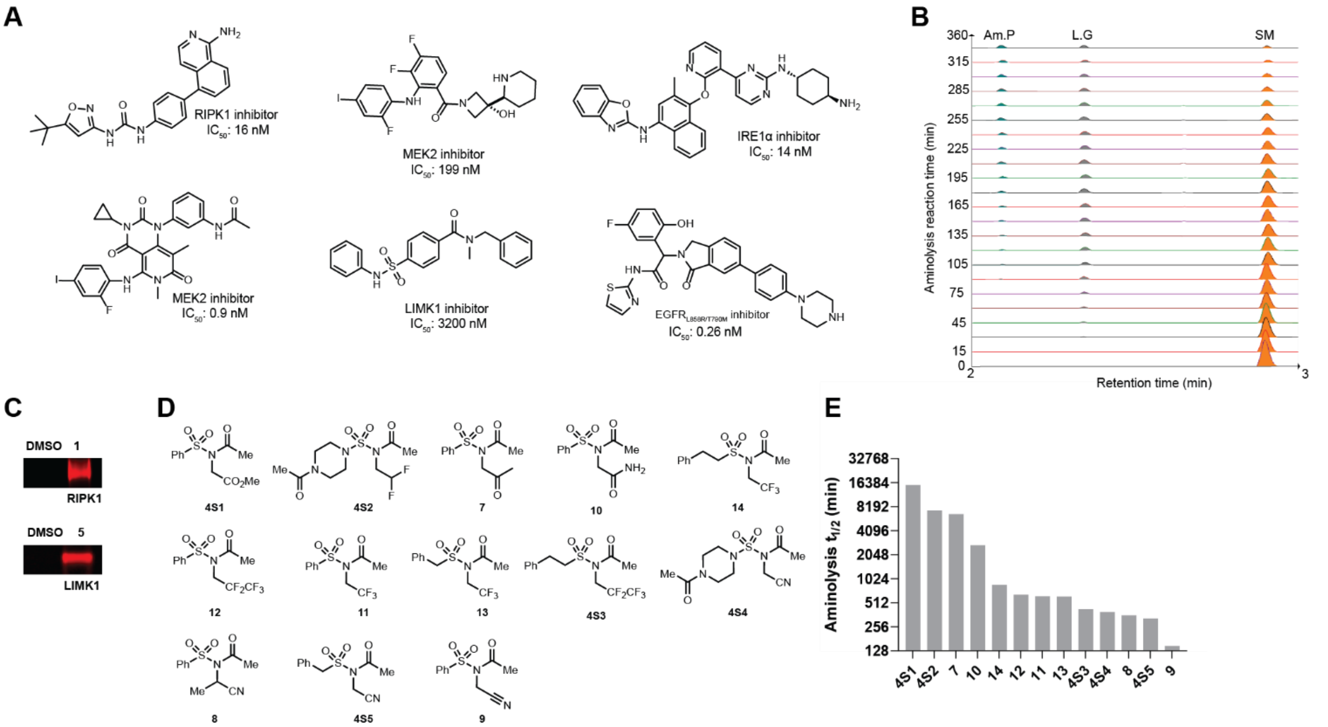

Figure S2. (A) Structure of allosteric kinase inhibitors derivatized for preparing kinase-NASA probes. **(B)** A representative HPLC chromatogram showing aminolysis of **5**. Am. P (aminolysis product), L.G. (leaving group), S.M. (starting material) **(C)** Representative blots of in-gel fluorescence studies using NASA probes demonstrating labeling of purified proteins. **(D-E)** Aminolytic half-lives of diverse NASA analogues. Compounds (0.1 mM) were incubated with Boc-Lys-coumarin (1 mM, Cat.No. 2C48530, 1clickchemistry) in acetonitrile at room temperature and the reaction was monitored by LC-MS. Full size blots of Figure S2C can be found at Figure S24.

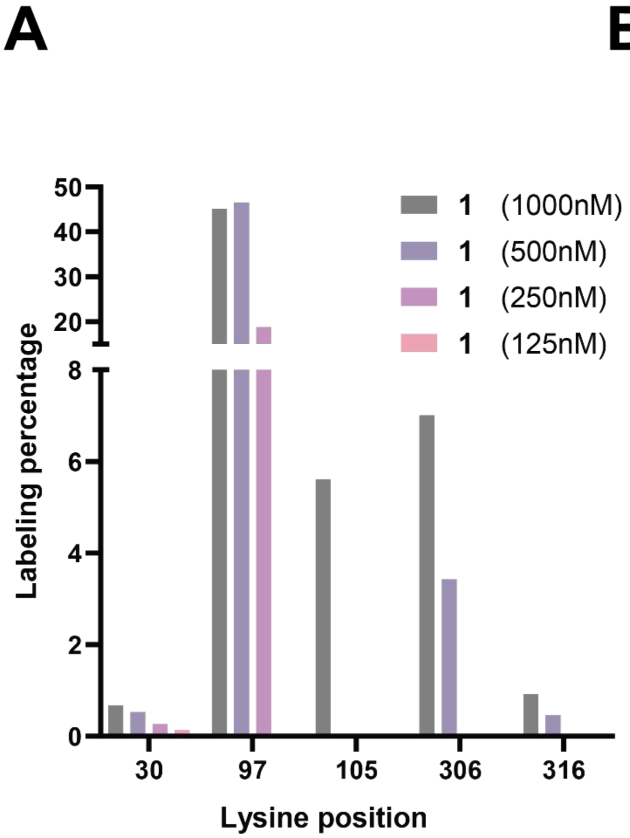

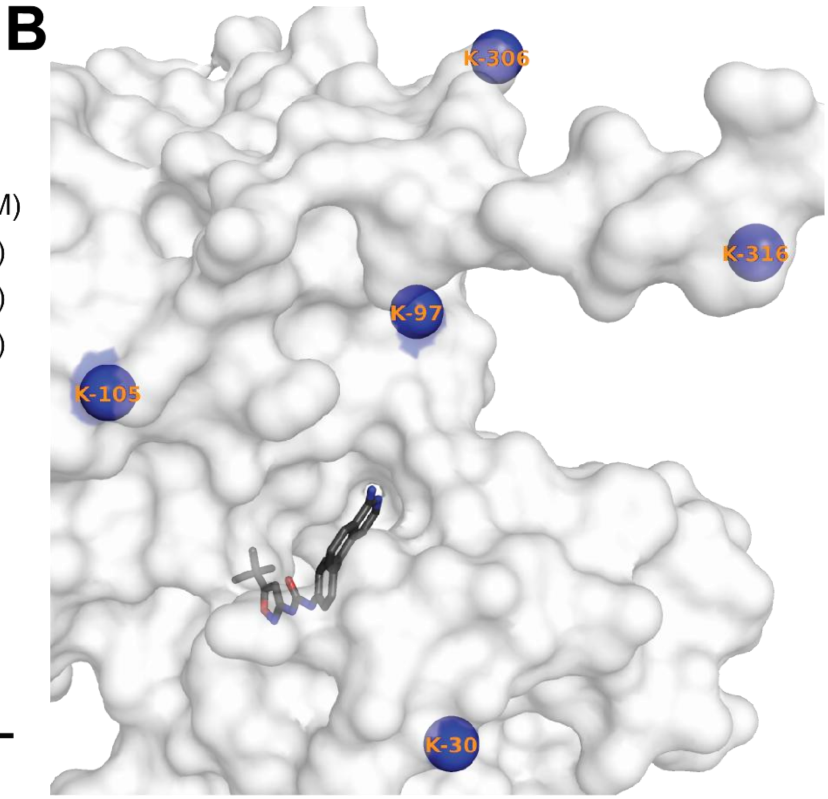

PDB (4NEU)

C

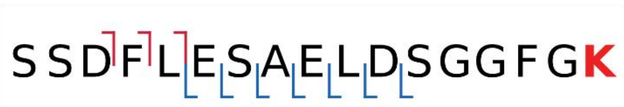

K-30

#1	b⁺	b²⁺	Seq.	y⁺	y²⁺	#2
1	88.03930	44.52329	S			17
2	175.07133	88.03930	S	1752.79628	876.90178	16
3	290.09828	145.55278	D	1665.76425	833.38576	15
4	437.16669	219.08698	F	1550.73731	775.87229	14
5	550.25075	275.62902	L	1403.66890	702.33809	13
6	679.29335	340.15031	E	1290.58483	645.79605	12
7	766.32538	383.66633	S	1161.54224	581.27476	11
8	837.36249	419.18488	A	1074.51021	537.75874	10
9	966.40508	483.70618	E	1003.47310	502.24019	9
10	1079.48915	540.24821	L	874.43050	437.71889	8
11	1194.51609	597.76168	D	761.34644	381.17686	7
12	1281.54812	641.27770	S	646.31950	323.66339	6
13	1338.56958	669.78843	G	559.28747	280.14737	5
14	1395.59104	698.29916	G	502.26601	251.63664	4
15	1542.65946	771.83337	F	445.24454	223.12591	3
16	1599.68092	800.34410	G	298.17613	149.59170	2
17			K-NASA_6...	241.15466	121.08097	1

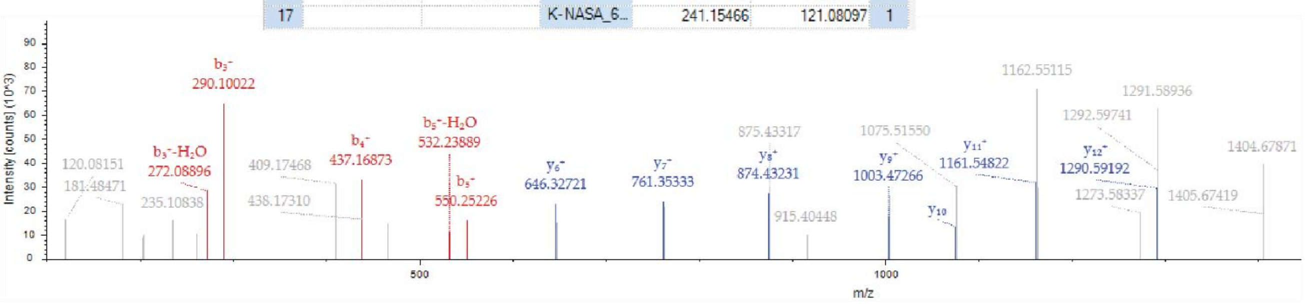

D

Y S L V M E Y M E **K** G N L M H V L K

K-97

#1	b⁺	b²⁺	b³⁺	Seq.	y⁺	y²⁺	y³⁺	#2
1	164.07061	82.53894	55.36172	Y				18
2	251.10263	126.05496	84.37240	S	2116.06395	1058.53561	706.02617	17
3	364.18670	182.59699	122.06708	L	2029.03192	1015.01960	677.01549	16
4	463.25511	232.13119	155.08989	V	1915.94785	958.47757	639.32080	15
5	594.29560	297.65144	198.77005	M	1816.87944	908.94336	606.29800	14
6	723.33819	362.17273	241.78425	E	1685.83896	843.42312	562.61784	13
7	886.40152	443.70440	296.13869	Y	1556.79636	778.90182	519.60364	12
8	1017.44200	509.22464	339.81885	M	1393.73303	697.37016	465.24920	11
9	1146.48459	573.74594	382.83305	E	1262.69255	631.84991	421.56903	10
10	1368.62142	684.81435	456.87866	K-NASA_6...	1133.64996	567.32862	378.55484	9
11	1425.64288	713.32508	475.88581	G	911.51313	456.26021	304.50923	8
12	1539.68581	770.34654	513.90012	N	854.49167	427.74947	285.50207	7
13	1652.76987	826.88857	551.59481	L	740.44874	370.72801	247.48777	6
14	1783.81036	892.40882	595.27497	M	627.36468	314.18598	209.79308	5
15	1920.86927	960.93827	640.96127	H	496.32419	248.66574	166.11292	4
16	2019.93768	1010.47248	673.98408	V	359.26528	180.13628	120.42661	3
17	2133.02175	1067.01451	711.67877	L	260.19687	130.60207	87.40381	2
18				K	147.11280	74.06004	49.70912	1

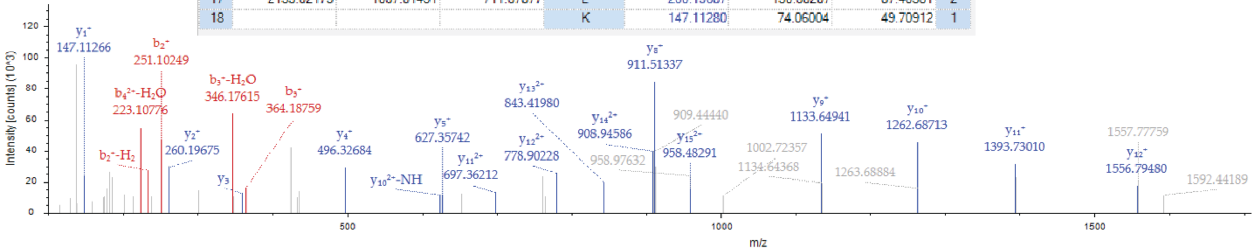

E

G N L M H V L **K** A E M S T P L S V K

K-105

#1	b⁺	b²⁺	b³⁺	Seq.	y⁺	y²⁺	y³⁺	#2
1	58.02874	29.51801	20.01443	G				18
2	172.07167	86.53947	58.02874	N	1992.06566	996.53647	664.69340	17
3	285.15573	143.08150	95.72343	L	1878.02273	939.51501	626.67910	16
4	416.19622	208.60175	139.40359	M	1764.93867	882.97297	588.98441	15
5	553.25513	277.13120	185.08989	H	1633.89818	817.45273	545.30425	14
6	652.32354	326.66541	218.11270	V	1496.83927	748.92327	499.61794	13
7	765.40761	383.20744	255.80739	L	1397.77086	699.38907	466.59514	12
8	987.54443	494.27585	329.85299	K-NASA_6...	1284.68680	642.84704	428.90045	11
9	1058.58154	529.79441	353.53203	A	1062.54997	531.77862	354.85484	10
10	1187.62414	594.31571	396.54623	E	991.51286	496.26007	331.17580	9
11	1318.66462	659.83595	440.22639	M	862.47027	431.73877	288.16161	8
12	1405.69665	703.35196	469.23707	S	731.42978	366.21853	244.48144	7
13	1506.74433	753.87580	502.91963	T	644.39775	322.70251	215.47077	6
14	1603.79709	802.40218	535.27055	P	543.35007	272.17868	181.78821	5
15	1716.88115	858.94422	572.96524	L	446.29731	223.65229	149.43729	4
16	1803.91318	902.46023	601.97591	S	333.21325	167.11026	111.74260	3
17	1902.98160	951.99444	634.99872	V	246.18122	123.59425	82.73192	2
18				K	147.11280	74.06004	49.70912	1

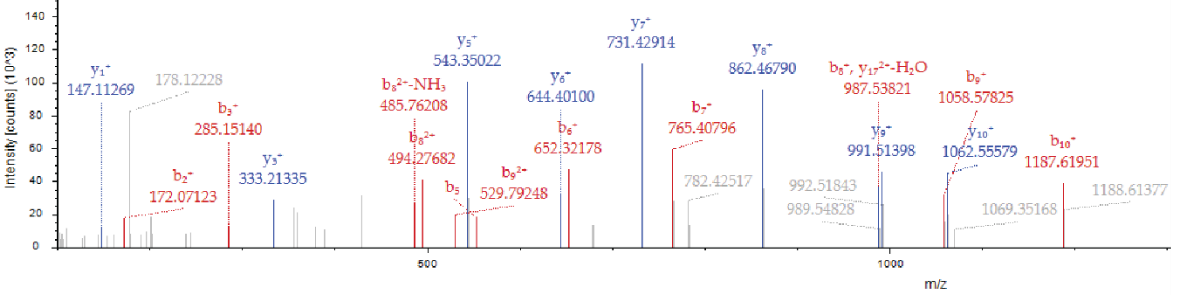

F

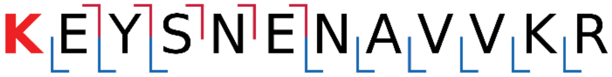

K-306

#1	b⁺	b²⁺	b³⁺	Seq.	y⁺	y²⁺	y³⁺	#2
1	223.14410	112.07569	75.05288	K-NASA_6...				12
2	352.18669	176.59698	118.06708	E	1308.65425	654.83077	436.88960	11
3	515.25002	258.12865	172.42152	Y	1179.61166	590.30947	393.87540	10
4	602.28205	301.64466	201.43220	S	1016.54833	508.77780	339.52096	9
5	716.32498	358.66613	239.44651	N	929.51630	465.26179	310.51029	8
6	845.36757	423.18742	282.46071	E	815.47338	408.24033	272.49598	7
7	959.41050	480.20889	320.47502	N	686.43078	343.71903	229.48178	6
8	1030.44761	515.72744	344.15405	A	572.38786	286.69757	191.46747	5
9	1129.51602	565.26165	377.17686	V	501.35074	251.17901	167.78843	4
10	1228.58444	614.79586	410.19966	V	402.28233	201.64480	134.76563	3
11	1356.67940	678.84334	452.89798	K	303.21392	152.11060	101.74282	2
12				R	175.11895	88.06311	59.04450	1

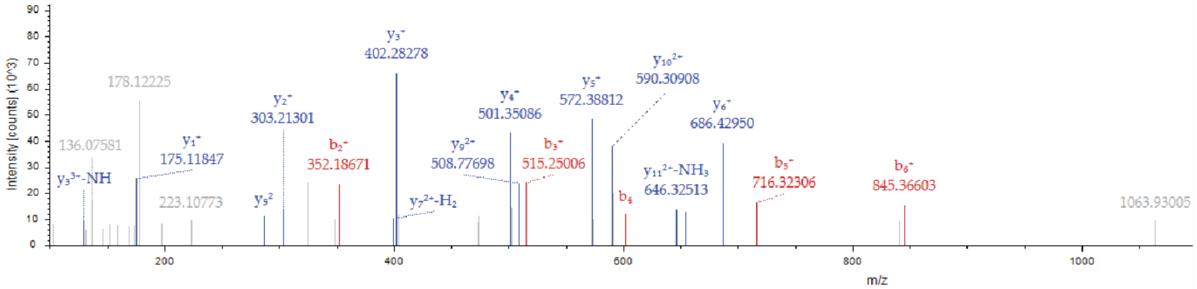

G

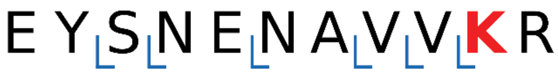

K-316

#1	b⁺	b²⁺	Seq.	y⁺	y²⁺	#2
1	130.04987	65.52857	E			11
2	293.11320	147.06024	Y	1273.65352	637.33040	10
3	380.14523	190.57625	S	1110.59019	555.79873	9
4	494.18815	247.59772	N	1023.55816	512.28272	8
5	623.23075	312.11901	E	909.51524	455.26126	7
6	737.27367	369.14048	N	780.47264	390.73996	6
7	808.31079	404.65903	A	666.42972	333.71850	5
8	907.37920	454.19324	V	595.39260	298.19994	4
9	1006.44762	503.72745	V	496.32419	248.66573	3
10	1228.58444	614.79586	K-NASA_6...	397.25578	199.13153	2
11			R	175.11895	88.06311	1

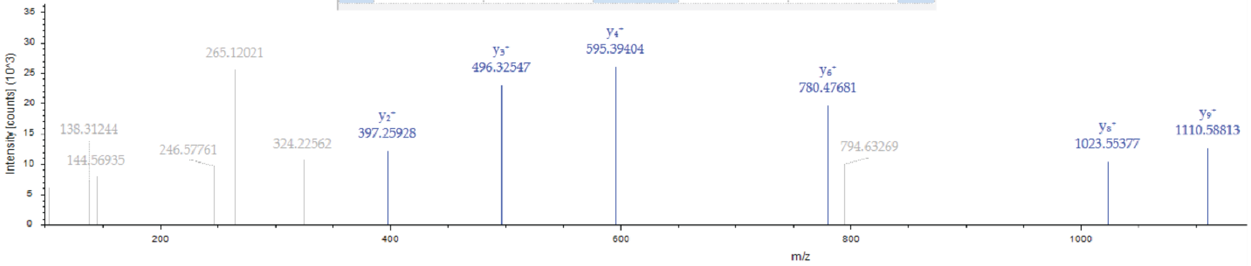

H

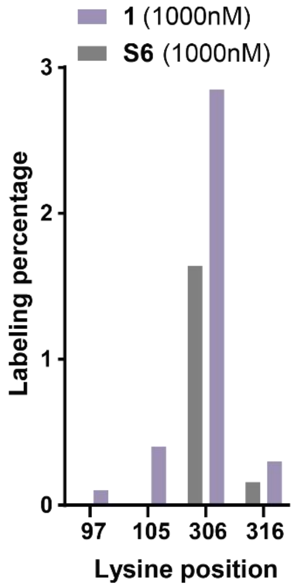

I

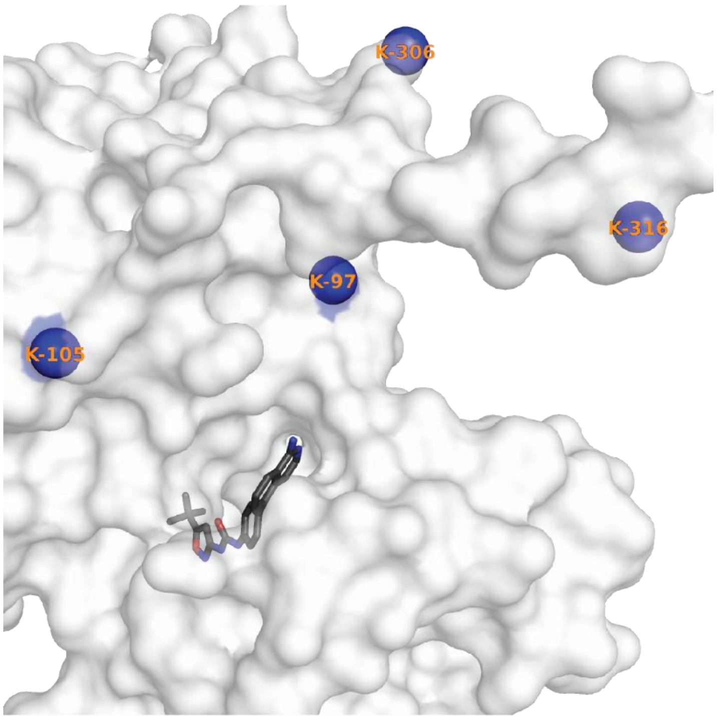

PDB (4NEU)

J

YSLVMEYMEKGNLMHVLK

K-97

#1	b⁺	b²⁺	Seq.	y⁺	y²⁺	#2
1	164.07061	82.53894	Y			18
2	251.10263	126.05496	S	2116.06395	1058.53561	17
3	364.18670	182.59699	L	2029.03192	1015.01960	16
4	463.25511	232.13119	V	1915.94785	958.47757	15
5	594.29560	297.65144	M	1816.87944	908.94336	14
6	723.33819	362.17273	E	1685.83896	843.42312	13
7	886.40152	443.70440	Y	1556.79636	778.90182	12
8	1017.44200	509.22464	M	1393.73303	697.37016	11
9	1146.48459	573.74594	E	1262.69255	631.84991	10
10	1368.62142	684.81435	K-NASA_6...	1133.64996	567.32862	9
11	1425.64288	713.32508	G	911.51313	456.26021	8
12	1539.68581	770.34654	N	854.49167	427.74947	7
13	1652.76987	826.88857	L	740.44874	370.72801	6
14	1783.81036	892.40882	M	627.36468	314.18598	5
15	1920.86927	960.93827	H	496.32419	248.66574	4
16	2019.93768	1010.47248	V	359.26528	180.13628	3
17	2133.02175	1067.01451	L	260.19687	130.60207	2
18			K	147.11280	74.06004	1

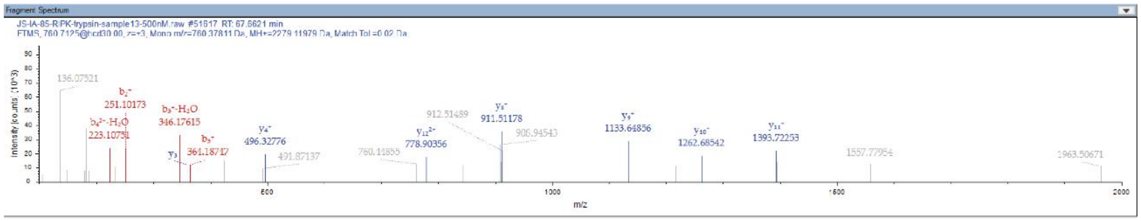

K

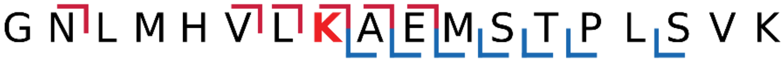

K-105

#1	b⁺	b²⁺	Seq.	y⁺	y²⁺	#2
1	58.02874	29.51801	G			18
2	172.07167	86.53947	N	1992.06566	996.53647	17
3	285.15573	143.08150	L	1878.02273	939.51501	16
4	416.19622	208.60175	M	1764.93867	882.97297	15
5	553.25513	277.13120	H	1633.89818	817.45273	14
6	652.32354	326.66541	V	1496.83927	748.92327	13
7	765.40761	383.20744	L	1397.77086	699.38907	12
8	987.54443	494.27585	K-NASA_6...	1284.68680	642.84704	11
9	1058.58154	529.79441	A	1062.54997	531.77862	10
10	1187.62414	594.31571	E	991.51286	496.26007	9
11	1318.66462	659.83595	M	862.47027	431.73877	8
12	1405.69665	703.35196	S	731.42978	366.21853	7
13	1506.74433	753.87580	T	644.39775	322.70251	6
14	1603.79709	802.40218	P	543.35007	272.17868	5
15	1716.88115	858.94422	L	446.29731	223.65229	4
16	1803.91318	902.46023	S	333.21325	167.11026	3
17	1902.98160	951.99444	V	246.18122	123.59425	2
18			K	147.11280	74.06004	1

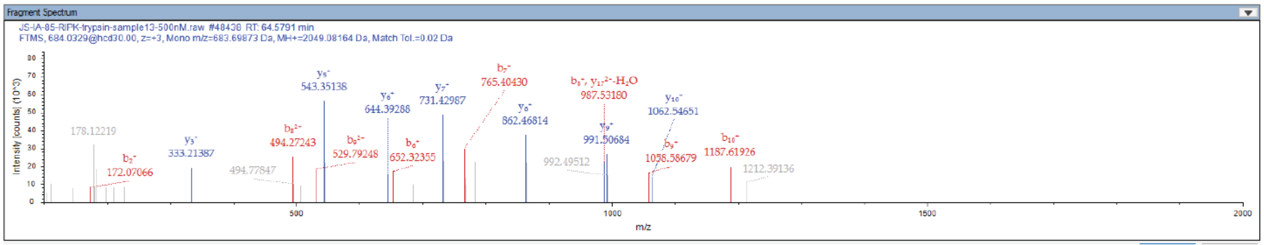

L

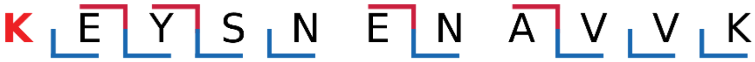

K-306

#1	b⁺	b²⁺	Seq.	y⁺	y²⁺	#2
1	223.14410	112.07569	K-NASA_6...			11
2	352.18669	176.59698	E	1152.55314	576.78021	10
3	515.25002	258.12865	Y	1023.51055	512.25891	9
4	602.28205	301.64466	S	860.44722	430.72725	8
5	716.32498	358.66613	N	773.41519	387.21124	7
6	845.36757	423.18742	E	659.37227	330.18977	6
7	959.41050	480.20889	N	530.32967	265.66847	5
8	1030.44761	515.72744	A	416.28675	208.64701	4
9	1129.51602	565.26165	V	345.24963	173.12845	3
10	1228.58444	614.79586	V	246.18122	123.59425	2
11			K	147.11280	74.06004	1

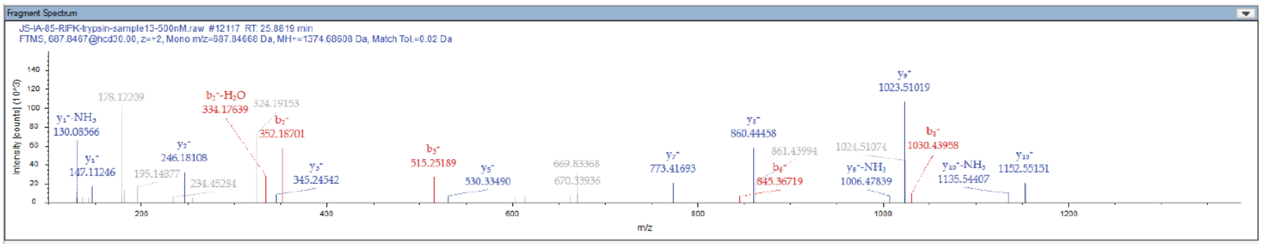

M

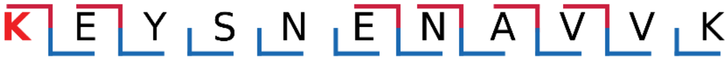

K-306

#1	b⁺	b²⁺	Seq.	y⁺	y²⁺	#2
1	223.14410	112.07569	K-NASA_6...			11
2	352.18669	176.59698	E	1152.55314	576.78021	10
3	515.25002	258.12865	Y	1023.51055	512.25891	9
4	602.28205	301.64466	S	860.44722	430.72725	8
5	716.32498	358.66613	N	773.41519	387.21124	7
6	845.36757	423.18742	E	659.37227	330.18977	6
7	959.41050	480.20889	N	530.32967	265.66847	5
8	1030.44761	515.72744	A	416.28675	208.64701	4
9	1129.51602	565.26165	V	345.24963	173.12845	3
10	1228.58444	614.79586	V	246.18122	123.59425	2
11			K	147.11280	74.06004	1

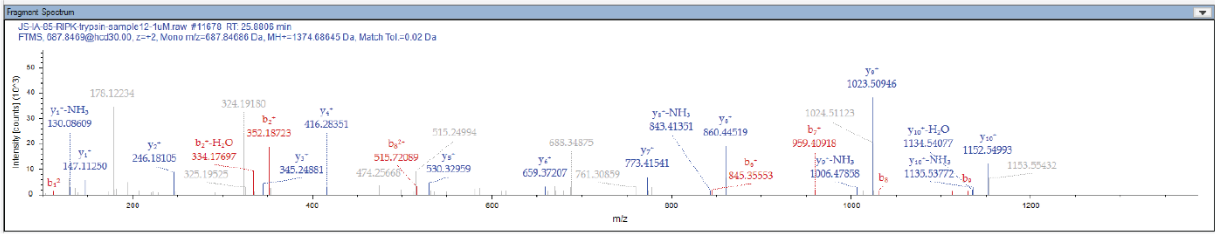

N

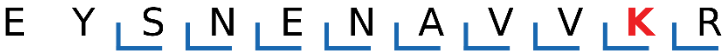

K-316

#1	b⁺	b²⁺	Seq.	y⁺	y²⁺	#2
1	130.04987	65.52857	E			11
2	293.11320	147.06024	Y	1273.65352	637.33040	10
3	380.14523	190.57625	S	1110.59019	555.79873	9
4	494.18815	247.59772	N	1023.55816	512.28272	8
5	623.23075	312.11901	E	909.51524	455.26126	7
6	737.27367	369.14048	N	780.47264	390.73996	6
7	808.31079	404.65903	A	666.42972	333.71850	5
8	907.37920	454.19324	V	595.39260	298.19994	4
9	1006.44762	503.72745	V	496.32419	248.66573	3
10	1228.58444	614.79586	K-NASA_6...	397.25578	199.13153	2
11			R	175.11895	88.06311	1

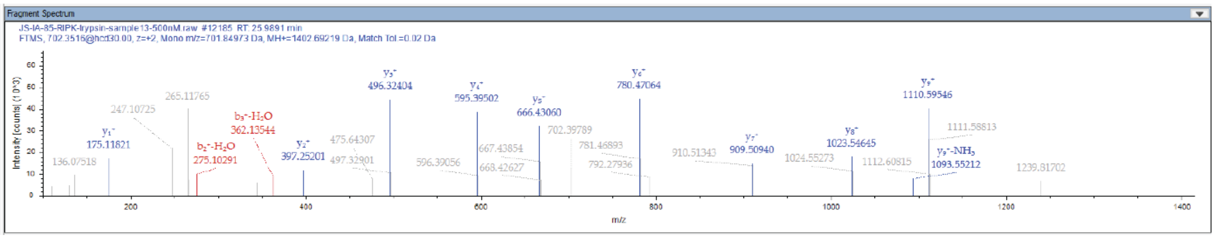

O

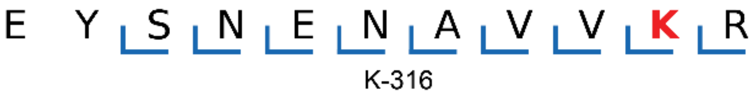

#1	b⁺	b²⁺	Seq.	y⁺	y²⁺	#2
1	130.04987	65.52857	E			11
2	293.11320	147.06024	Y	1273.65352	637.33040	10
3	380.14523	190.57625	S	1110.59019	555.79873	9
4	494.18815	247.59772	N	1023.55816	512.28272	8
5	623.23075	312.11901	E	909.51524	455.26126	7
6	737.27367	369.14048	N	780.47264	390.73996	6
7	808.31079	404.65903	A	666.42972	333.71850	5
8	907.37920	454.19324	V	595.39260	298.19994	4
9	1006.44762	503.72745	V	496.32419	248.66573	3
10	1228.58444	614.79586	K-NASA_6...	397.25578	199.13153	2
11			R	175.11895	88.06311	1

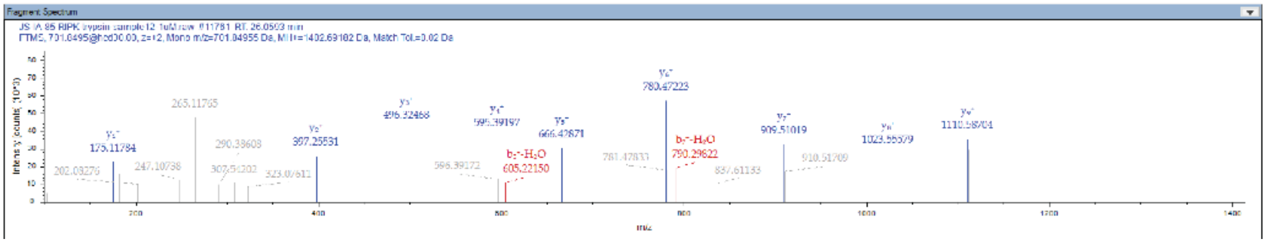

Figure S3. (A) RIPK1-nitrile NASA probe **1** labels multiple lysines near the binding pocket. **(B)** Crystal structure of RIPK1 showing multiple lysines (blue sphere) labelled by **1**, RIPK1 (PDB: 4NEU), ligand shown as sticks, missing residues were modelled with ChimeraX-MODELLER. **(C-G)** MS/MS spectrum of RIPK1 labeled peptides with **1**. **(H-I)** Lysine labeling by RIPK1-nitrile NASA probe **1** (another replicate) and SuFA probe **S6** near the binding pocket. **(J-L** and **N)** MS/MS spectrum of RIPK1 labeled peptides with **1. (M** and **O)** MS/MS spectrum of RIPK1 labeled peptides with SuFA probe **S6**.

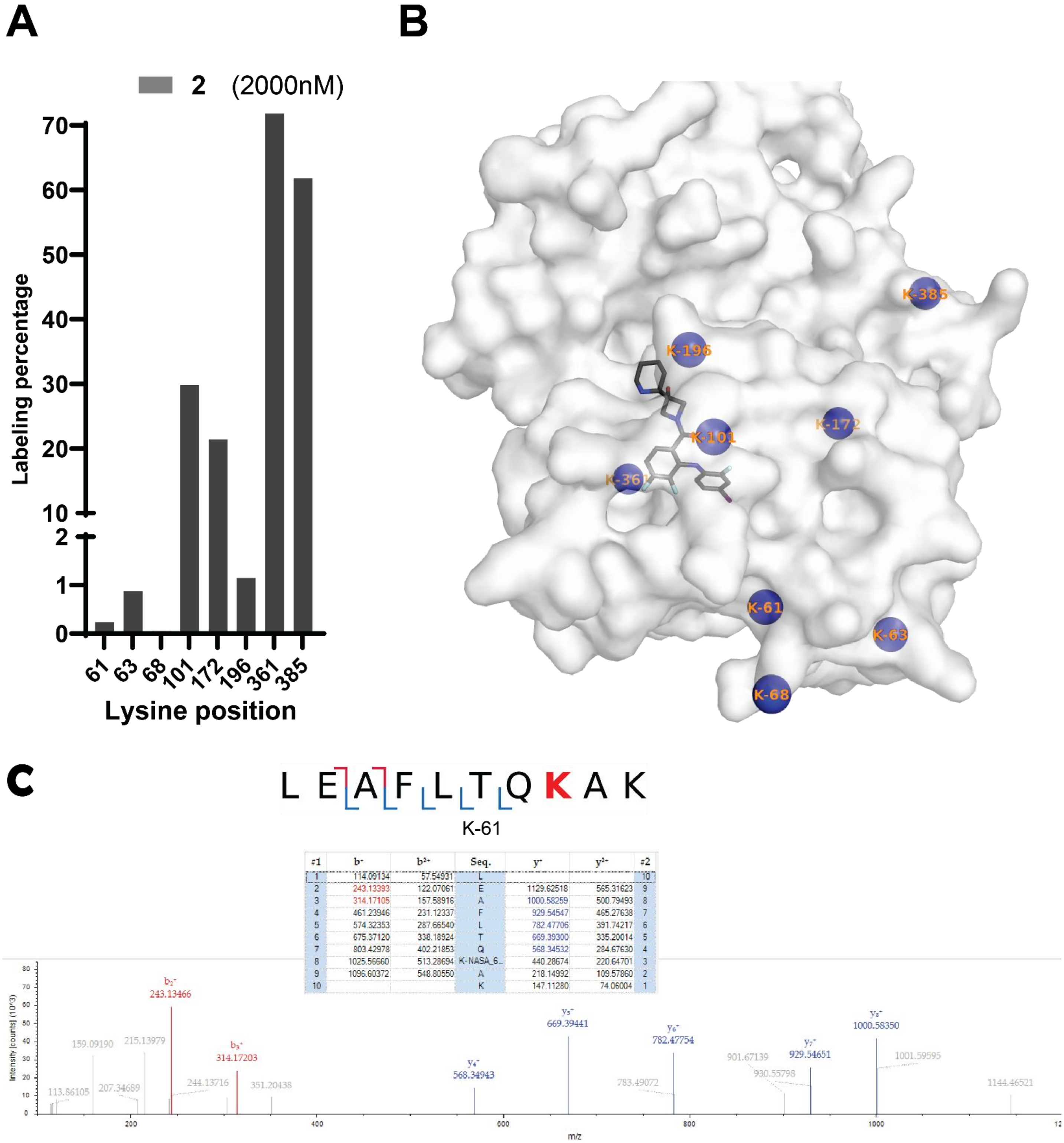

#1	b⁺	b²⁺	Seq.	y⁺	y²⁺	#2
1	114.09134	57.54931	L			10
2	243.13393	122.07061	E	1129.62518	565.31623	9
3	314.17105	157.58916	A	1000.58259	500.79493	8
4	461.23946	231.12337	F	929.54547	465.27638	7
5	574.32353	287.66540	L	782.47706	391.74217	6
6	675.37120	338.18924	T	669.39300	335.20014	5
7	803.42978	402.21853	Q	568.34532	284.67630	4
8	1025.56660	513.28694	K-NASA_6...	440.28674	220.64701	3
9	1096.60372	548.80550	A	218.14992	109.57860	2
10			K	147.11280	74.06004	1

D

A K V G E L K D D D F E R

K-63

#1	b⁺	b²⁺	b³⁺	Seq.	y⁺	y²⁺	y³⁺	#2
1	72.04439	36.52583	24.68631	A				13
2	294.18121	147.59424	98.73192	K-NASA_6...	1544.75911	772.88319	515.59122	12
3	393.24963	197.12845	131.75473	V	1322.62229	661.81478	441.54561	11
4	450.27109	225.63918	150.76188	G	1223.55387	612.28057	408.52281	10
5	579.31368	290.16048	193.77608	E	1166.53241	583.76984	389.51565	9
6	692.39775	346.70251	231.47077	L	1037.48981	519.24855	346.50146	8
7	820.49271	410.74999	274.16909	K	924.40575	462.70651	308.80677	7
8	935.51965	468.26347	312.51140	D	796.31079	398.65903	266.10845	6
9	1050.54660	525.77694	350.85372	D	681.28385	341.14556	227.76613	5
10	1165.57354	583.29041	389.19603	D	566.25690	283.63209	189.42382	4
11	1312.64195	656.82462	438.21884	F	451.22996	226.11862	151.08150	3
12	1441.68455	721.34591	481.23303	E	304.16155	152.58441	102.05870	2
13				R	175.11895	88.06311	59.04450	1

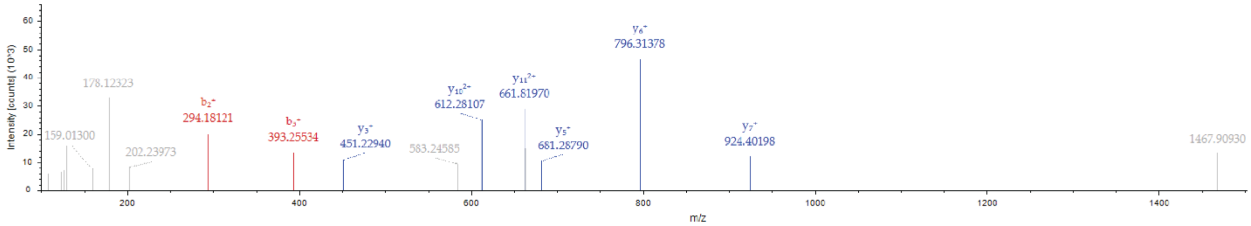

E

V G E L K D D D F E R

K-68

#1	b⁺	b²⁺	Seq.	y⁺	y²⁺	#2
1	100.07569	50.54148	V			11
2	157.09715	79.05222	G	1317.59573	659.30150	10
3	286.13975	143.57351	E	1260.57427	630.79077	9
4	399.22381	200.11554	L	1131.53167	566.26948	8
5	621.36063	311.18396	K-NASA_6...	1018.44761	509.72744	7
6	736.38758	368.69743	D	796.31079	398.65903	6
7	851.41452	426.21090	D	681.28385	341.14556	5
8	966.44146	483.72437	D	566.25690	283.63209	4
9	1113.50988	557.25858	F	451.22996	226.11862	3
10	1242.55247	621.77987	E	304.16155	152.58441	2
11			R	175.11895	88.06311	1

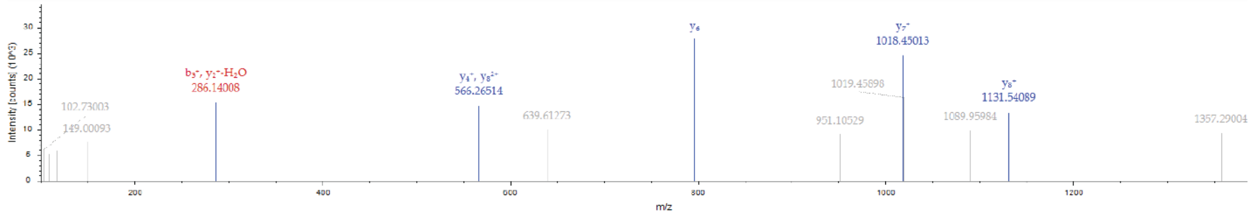

F

K L I H L E I **K** P A I R

K-101

1	129.10224	65.05476	43.70560	K				12
2	242.18630	121.59679	81.40029	L	1396.86748	698.93738	466.29401	11
3	355.27037	178.13882	119.09497	I	1283.78341	642.39534	428.59932	10
4	492.32928	246.66828	164.78128	H	1170.69935	585.85331	390.90463	9
5	605.41334	303.21031	202.47597	L	1033.64044	517.32386	345.21833	8
6	734.45594	367.73161	245.49016	E	920.55637	460.78183	307.52364	7
7	847.54000	424.27364	283.18485	I	791.51378	396.26053	264.50944	6
8	1069.67682	535.34205	357.23046	K-NASA_6...	678.42972	339.71850	226.81476	5
9	1166.72959	583.86843	389.58138	P	456.29289	228.65009	152.76915	4
10	1237.76670	619.38699	413.26042	A	359.24013	180.12370	120.41823	3
11	1350.85076	675.92902	450.95511	I	288.20302	144.60515	96.73919	2
12				R	175.11895	88.06311	59.04450	1

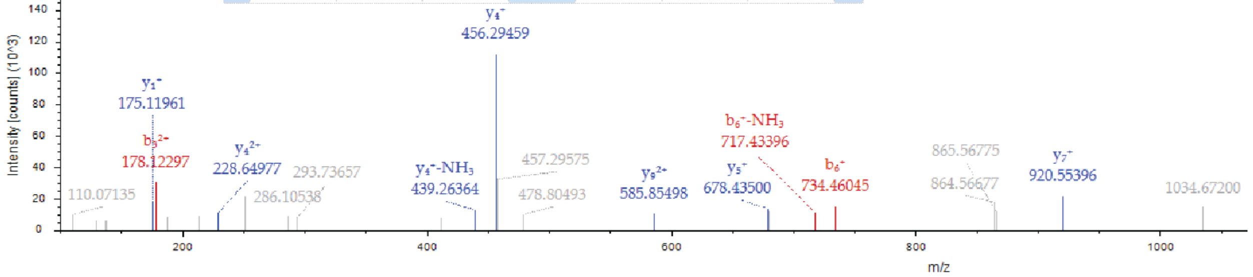

G

E A **K** R I P E E I L G K

K-163

#1	b^+	b^{2+}	b^{3+}	Seq.	y^+	y^{2+}	y^{3+}	#2
1	130.04987	65.52857	44.02147	E				12
2	201.08698	101.04713	67.70051	A	1347.79946	674.40337	449.93800	11
3	423.22381	212.11554	141.74612	K-NASA_6...	1276.76234	638.88481	426.25897	10
4	579.32492	290.16610	193.77982	R	1054.62552	527.81640	352.21336	9
5	692.40898	346.70813	231.47451	I	898.52441	449.76584	300.17965	8
6	789.46175	395.23451	263.82543	P	785.44035	393.22381	262.48497	7
7	918.50434	459.75581	306.83963	E	688.38758	344.69743	230.13405	6
8	1047.54693	524.27710	349.85383	E	559.34499	280.17613	187.11985	5
9	1160.63100	580.81914	387.54852	I	430.30240	215.65484	144.10565	4
10	1273.71506	637.36117	425.24320	L	317.21833	159.11280	106.41096	3
11	1330.73652	665.87190	444.25036	G	204.13427	102.57077	68.71627	2
12				K	147.11280	74.06004	49.70912	1

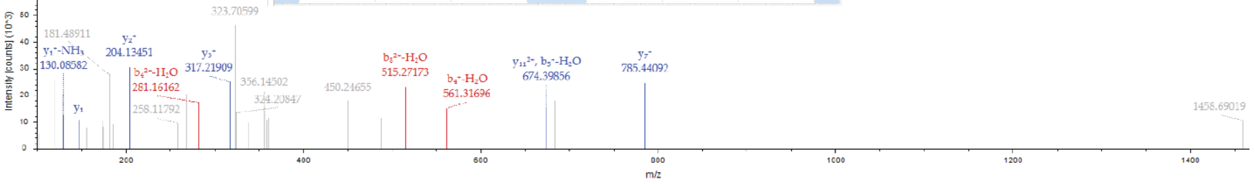

H

R I P E E I L G **K** V S I A V L R

K-172

#1	b⁺	b²⁺	b³⁺	Seq.	y⁺	y²⁺	y³⁺	#2
1	157.10839	79.05783	53.04098	R				16
2	270.19245	135.59986	90.73567	I	1731.04148	866.02438	577.68534	15
3	367.24522	184.12625	123.08659	P	1617.95741	809.48235	539.99066	14
4	496.28781	248.64754	166.10079	E	1520.90465	760.95596	507.63973	13
5	625.33040	313.16884	209.11498	E	1391.86206	696.43467	464.62554	12
6	738.41447	369.71087	246.80967	I	1262.81946	631.91337	421.61134	11
7	851.49853	426.25290	284.50436	L	1149.73540	575.37134	383.91665	10
8	908.51999	454.76363	303.51152	G	1036.65134	518.82931	346.22196	9
9	1130.65682	565.83205	377.55712	K-NASA_6...	979.62987	490.31857	327.21481	8
10	1229.72523	615.36625	410.57993	V	757.49305	379.25016	253.16920	7
11	1316.75726	658.88227	439.59060	S	658.42464	329.71596	220.14640	6
12	1429.84132	715.42430	477.28529	I	571.39261	286.19994	191.13572	5
13	1500.87844	750.94286	500.96433	A	458.30854	229.65791	153.44103	4
14	1599.94685	800.47706	533.98713	V	387.27143	194.13935	129.76199	3
15	1713.03091	857.01910	571.68182	L	288.20302	144.60515	96.73919	2
16				R	175.11895	88.06311	59.04450	1

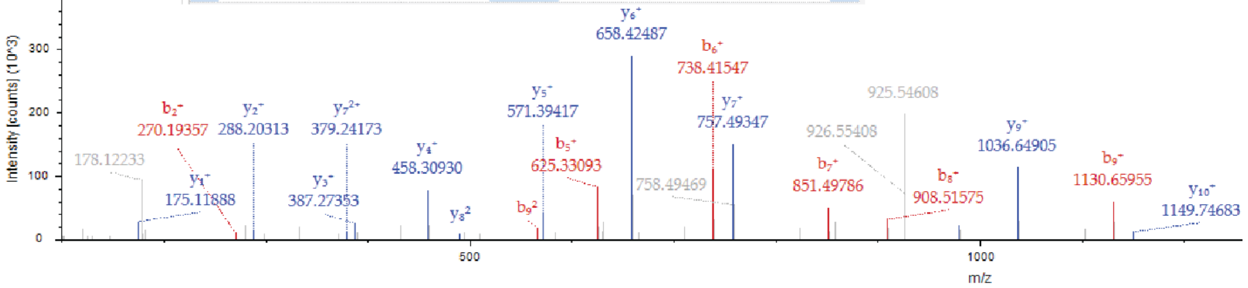

I

D V **K** P S N I L V N S R

K-196

#1	b⁺	b²⁺	Seq.	y⁺	y²⁺	#2
1	116.03422	58.52075	D			12
2	215.10263	108.05496	V	1320.76341	660.88534	11
3	437.23946	219.12337	K-NASA_6...	1221.69499	611.35113	10
4	534.29222	267.64975	P	999.55817	500.28272	9
5	621.32425	311.16576	S	902.50541	451.75634	8
6	735.36718	368.18723	N	815.47338	408.24033	7
7	848.45124	424.72926	I	701.43045	351.21886	6
8	961.53530	481.27129	L	588.34639	294.67683	5
9	1060.60372	530.80550	V	475.26232	238.13480	4
10	1174.64665	587.82696	N	376.19391	188.60059	3
11	1261.67867	631.34298	S	262.15098	131.57913	2
12			R	175.11895	88.06311	1

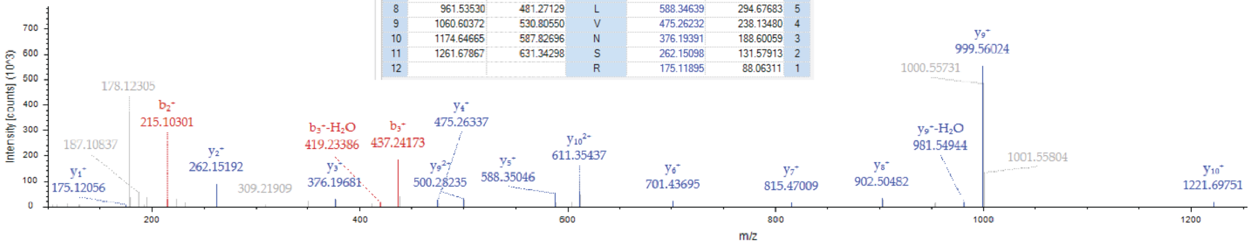

J

A D L **K** M L T N H T F I K

K-361

#1	b⁺	b²⁺	b³⁺	Seq.	y⁺	y²⁺	y³⁺	#2
1	72.04439	36.52583	24.68631	A				13
2	187.07133	94.03930	63.02863	D	1554.83486	777.92107	518.94980	12
3	300.15540	150.58134	100.72332	L	1439.80791	720.40760	480.60749	11
4	522.29222	261.64975	174.76892	K-NASA_6...	1326.72385	663.86556	442.91280	10
5	653.33270	327.16999	218.44909	M	1104.58703	552.79715	368.86719	9
6	766.41677	383.71202	256.14377	L	973.54654	487.27691	325.18703	8
7	867.46445	434.23586	289.82633	T	860.46248	430.73488	287.49234	7
8	981.50737	491.25733	327.84064	N	759.41480	380.21104	253.80978	6
9	1118.56629	559.78678	373.52695	H	645.37187	323.18957	215.79548	5
10	1219.61396	610.31062	407.20951	T	508.31296	254.66012	170.10917	4
11	1366.68238	683.84483	456.23231	F	407.26528	204.13628	136.42661	3
12	1479.76644	740.38686	493.92700	I	260.19687	130.60207	87.40381	2
13				K	147.11280	74.06004	49.70912	1

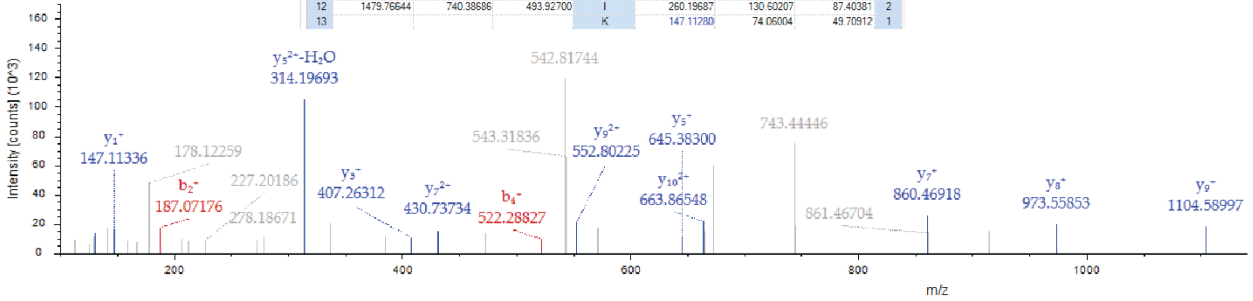

K

R S E V E E V D F A G W L C **K** T L R

K-385

#1	b⁺	b²⁺	b³⁺	Seq.	y⁺	y²⁺	y³⁺	#2
1	157.10839	79.05783	53.04098	R				18
2	244.14042	122.57385	82.05166	S	2133.03211	1067.01969	711.68222	17
3	373.18301	187.09514	125.06585	E	2046.00008	1023.50368	682.67155	16
4	472.25142	236.62935	158.08866	V	1916.95749	958.98238	639.65735	15
5	601.29402	301.15065	201.10286	E	1817.88908	909.44818	606.63454	14
6	730.33661	365.67194	244.11705	E	1688.84648	844.92688	563.62035	13
7	829.40502	415.20615	277.13986	V	1559.80389	780.40558	520.60615	12
8	944.43197	472.71962	315.48217	D	1460.73548	730.87138	487.58334	11
9	1091.50038	546.25383	364.50498	F	1345.70853	673.35791	449.24103	10
10	1162.53749	581.77238	388.18402	A	1198.64012	599.82370	400.21822	9
11	1219.55896	610.28312	407.19117	G	1127.60301	564.30514	376.53919	8
12	1405.63827	703.32277	469.21761	W	1070.58154	535.79441	357.53203	7
13	1518.72233	759.86481	506.91230	L	884.50223	442.75475	295.50559	6
14	1678.75298	839.88013	560.25585	C-Carbami...	771.41817	386.21272	257.81091	5
15	1900.88981	950.94854	634.30145	K-NASA_6...	611.38752	306.19740	204.46736	4
16	2001.93748	1001.47238	667.98401	T	389.25069	195.12899	130.42175	3
17	2115.02155	1058.01441	705.67870	L	288.20302	144.60515	96.73919	2
18				R	175.11895	88.06311	59.04450	1

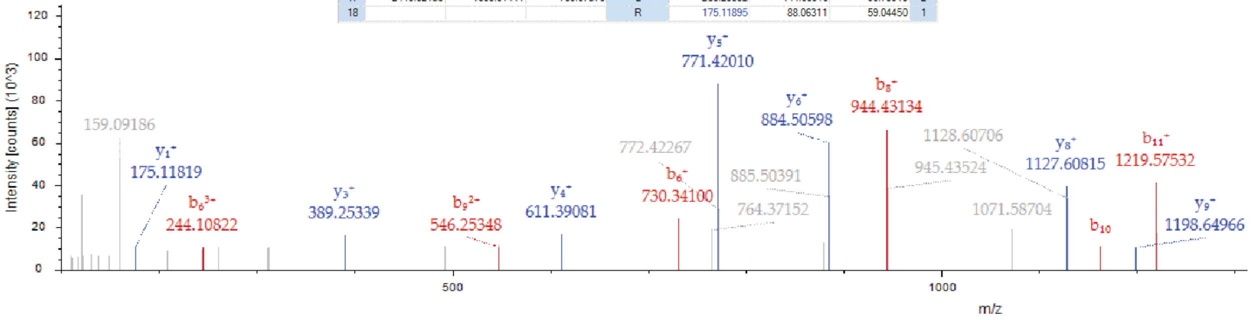

Figure S4. (A) MEK2-nitrile NASA probe **2** labels multiple lysines near the binding pocket. **(B)** Crystal structure of MEK2 showing multiple lysines (blue sphere) labelled by **2**; MEK2 (PDB 1S9I) aligned with MEK1 (PDB 7JUS), ligand VKD shown as sticks. **(C-K)** MS/MS spectrum of MEK2 labeled peptides with **2**.

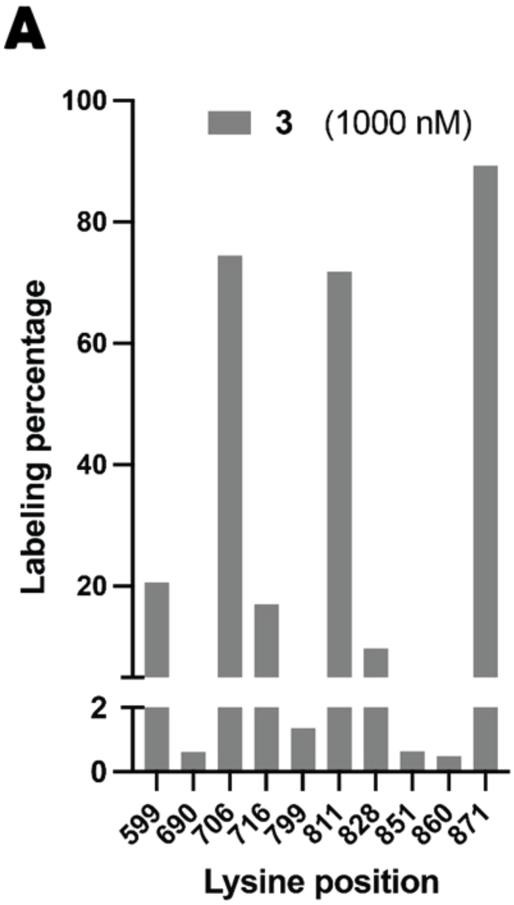

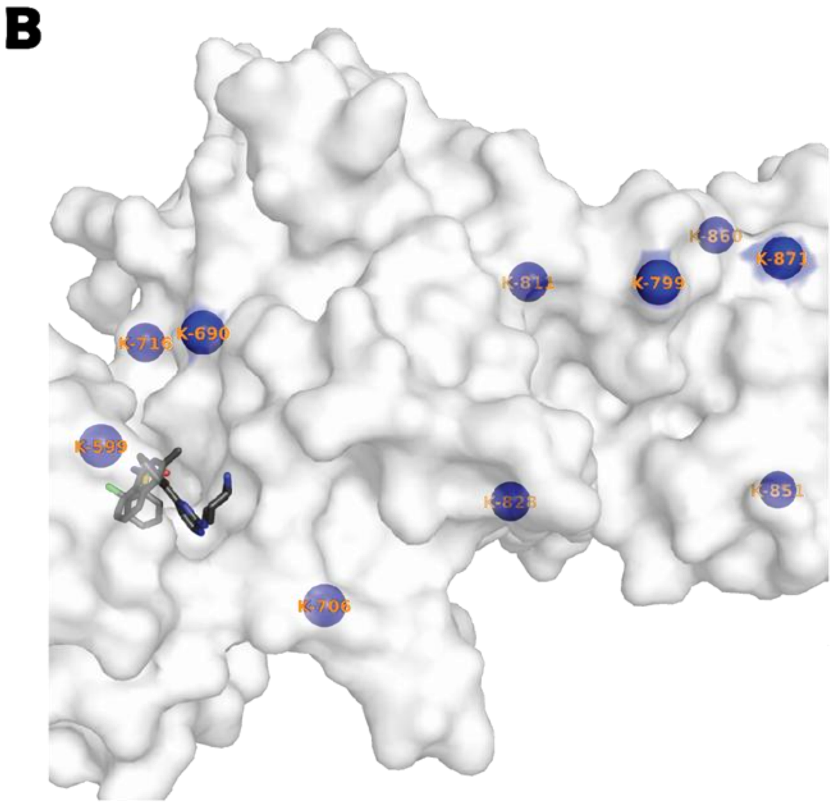

PDB (4U6R)

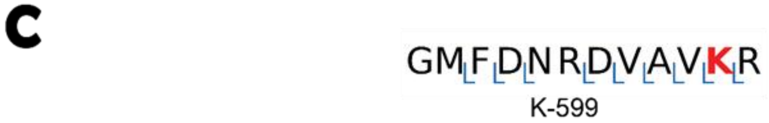

#1	b+	b2+	b3+	Seq.	y+	y2+	y3+	#2
1	58.02874	29.51801	20.01443	G				12
2	189.06922	95.03825	63.69459	M	1430.72089	715.86408	477.57848	11
3	336.13764	168.57246	112.71740	F	1299.68040	650.34384	433.89832	10
4	451.16458	226.08593	151.05971	D	1152.61199	576.80963	384.87551	9
5	565.20751	283.10739	189.07402	N	1037.58505	519.29616	346.53320	8
6	721.30862	361.15795	241.10772	R	923.54212	462.27470	308.51889	7
7	836.33556	418.67142	279.45004	D	767.44101	384.22414	256.48519	6
8	935.40398	468.20563	312.47284	V	652.41407	326.71067	218.14287	5
9	1006.44109	503.72418	336.15188	A	553.34565	277.17646	185.12007	4
10	1105.50950	553.25839	369.17469	V	482.30854	241.65791	161.44103	3
11	1313.63068	657.31898	438.54841	K-SAS_NA...	383.24013	192.12370	128.41823	2
12				R	175.11895	88.06311	59.04450	1

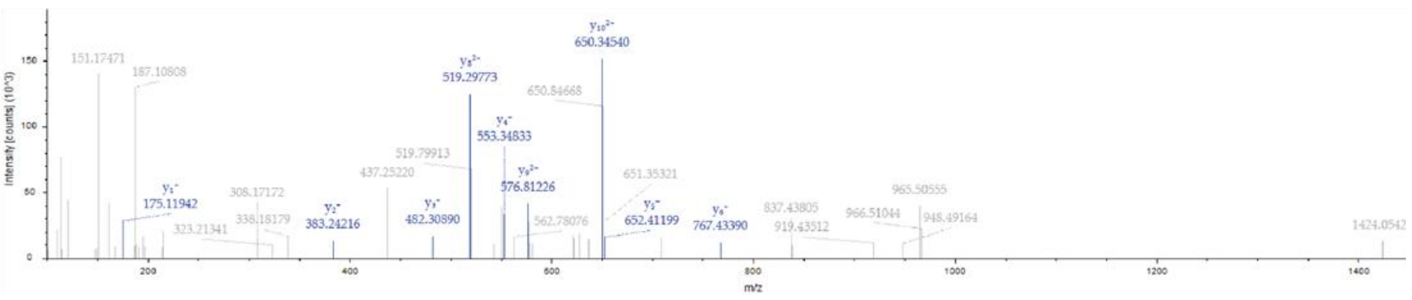

D

D L K P H N I L I S M P N A H G K

K-690

#1	b⁺	b²⁺	b³⁺	Seq.	y⁺	y²⁺	y³⁺	#2
1	116.03422	58.52075	39.34959	D				17
2	229.11828	115.06278	77.04428	L	1850.01053	925.50890	617.34169	16
3	437.23946	219.12337	146.41800	K-SAS_NA...	1736.92647	868.96687	579.64701	15
4	534.29222	267.64975	178.76892	P	1528.80529	764.90628	510.27328	14
5	671.35113	336.17920	224.45523	H	1431.75253	716.37990	477.92236	13
6	785.39406	393.20067	262.46954	N	1294.69362	647.85045	432.23606	12
7	898.47812	449.74270	300.16423	I	1180.65069	590.82898	394.22175	11
8	1011.56219	506.28473	337.85891	L	1067.56663	534.28695	356.52706	10
9	1124.64625	562.82676	375.55360	I	954.48256	477.74492	318.83237	9
10	1211.67828	606.34278	404.56428	S	841.39850	421.20289	281.13768	8
11	1342.71876	671.86302	448.24444	M	754.36647	377.68687	252.12701	7
12	1439.77153	720.38940	480.59536	P	623.32598	312.16663	208.44685	6
13	1553.81446	777.41087	518.60967	N	526.27322	263.64025	176.09592	5
14	1624.85157	812.92942	542.28871	A	412.23029	206.61879	138.08162	4
15	1761.91048	881.45888	587.97501	H	341.19318	171.10023	114.40258	3
16	1818.93194	909.96961	606.98217	G	204.13427	102.57077	68.71627	2
17				K	147.11280	74.06004	49.70912	1

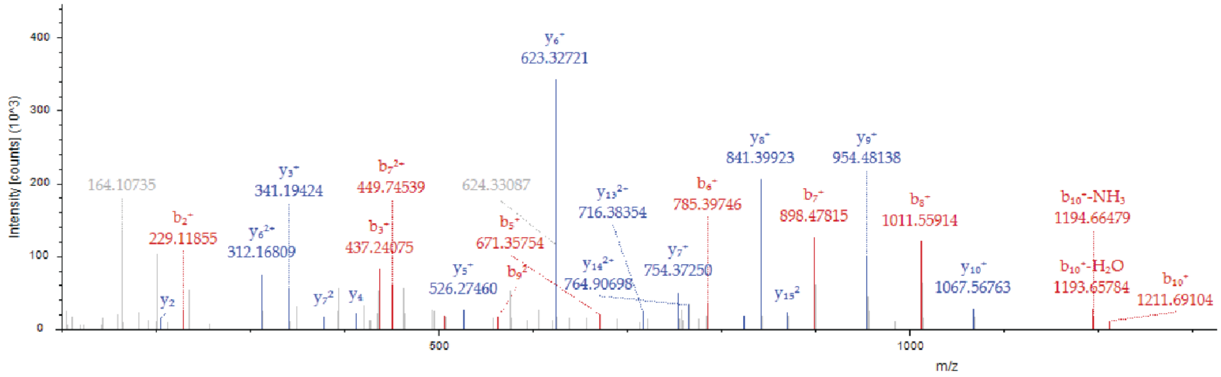

E

I K A M I S D F G L C K

K-706

#1	b⁺	b²⁺	Seq.	y⁺	y²⁺	#2
1	114.09134	57.54931	I			12
2	322.21251	161.60990	K-SAS_NA...	1349.65920	675.33324	11
3	393.24963	197.12845	A	1141.53803	571.27265	10
4	524.29011	262.64869	M	1070.50091	535.75410	9
5	637.37418	319.19073	I	939.46043	470.23385	8
6	724.40620	362.70674	S	826.37637	413.69182	7
7	839.43315	420.22021	D	739.34434	370.17581	6
8	986.50156	493.75442	F	624.31739	312.66234	5
9	1043.52302	522.26515	G	477.24898	239.12813	4
10	1156.60709	578.80718	L	420.22752	210.61740	3
11	1316.63774	658.82251	C-Carbami...	307.14345	154.07536	2
12			K	147.11280	74.06004	1

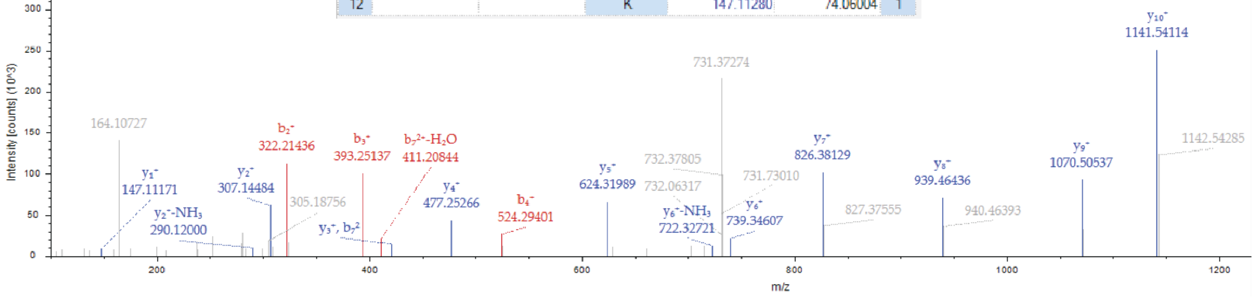

F

A M I S D F G L C **K** K

K-716

#1	b+	b2+	Seq.	y+	y2+	#2
1	72.04439	36.52583	A			11
2	203.08487	102.04608	M	1278.62209	639.81468	10
3	316.16894	158.58811	I	1147.58160	574.29444	9
4	403.20097	202.10412	S	1034.49754	517.75241	8
5	518.22791	259.61759	D	947.46551	474.23639	7
6	665.29632	333.15180	F	832.43857	416.72292	6
7	722.31779	361.66253	G	685.37015	343.18871	5
8	835.40185	418.20456	L	628.34869	314.67798	4
9	995.43250	498.21989	C-Carbami...	515.26463	258.13595	3
10	1203.55367	602.28047	K-SAS_NA...	355.23398	178.12063	2
11			K	147.11280	74.06004	1

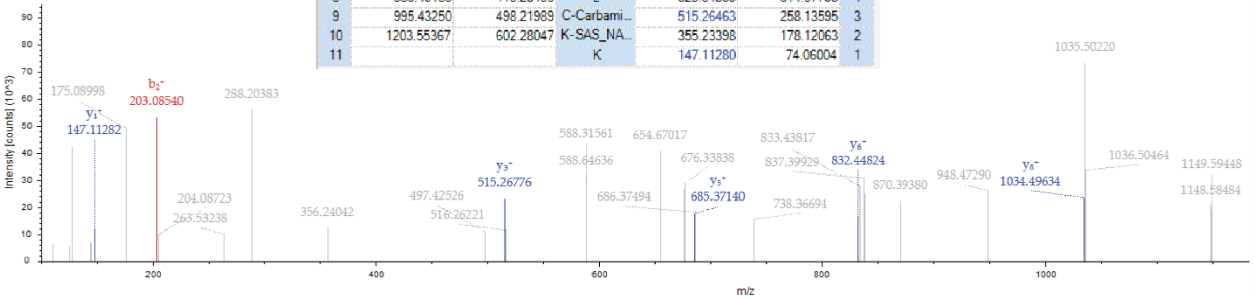

G

Q A N I L L G A C S L D C L H P E **K** H E D V I A R

K-799

#1	b+	b2+	b3+	b4+	Seq.	y+	y2+	y3+	y4+	#2
1	129.06585	65.03657	43.69347	33.02192	Q					25
2	200.10297	100.55512	67.37251	50.78120	A	2811.39164	1406.19946	937.80206	703.60337	24
3	314.14590	157.57659	105.38682	79.29193	N	2740.35453	1370.68090	914.12303	685.84409	23
4	427.22996	214.11862	143.08150	107.56295	I	2626.31160	1313.65944	876.10872	657.33336	22
5	540.31402	270.66065	180.77619	135.83396	L	2513.22753	1257.11741	838.41403	629.06234	21
6	653.39809	327.20268	218.47088	164.10498	L	2400.14347	1200.57537	800.71934	600.79133	20
7	710.41955	355.71341	237.47803	178.36035	G	2287.05941	1144.03334	763.02465	572.52031	19
8	781.45666	391.23197	261.15707	196.11962	A	2230.03794	1115.52261	744.01750	558.26494	18
9	941.48731	471.24729	314.50062	236.12729	C-Carbami...	2159.00083	1080.00405	720.33846	540.50566	17
10	1028.51934	514.76331	343.51130	257.88529	S	1998.97018	999.98873	666.99491	500.49800	16
11	1141.60341	571.30534	381.20599	286.15631	L	1911.93815	956.47271	637.98424	478.74000	15
12	1256.63035	628.81881	419.54830	314.91304	D	1798.85409	899.93068	600.28955	450.46898	14
13	1416.66100	708.83414	472.89185	354.92071	C-Carbami...	1683.82715	842.41721	561.94723	421.71224	13
14	1529.74506	765.37617	510.58654	383.19172	L	1523.79650	762.40189	508.60368	381.70458	12
15	1666.80397	833.90562	556.27284	417.45645	H	1410.71243	705.85985	470.90900	353.43357	11
16	1763.85674	882.43201	588.62376	441.71964	P	1273.65352	637.33040	425.22269	319.16884	10
17	1892.89933	946.95330	631.63796	473.98029	E	1176.60076	588.80402	392.87177	294.90565	9
18	2101.02050	1051.01389	701.01169	526.01058	K-SAS_NA...	1047.55816	524.28272	349.85757	262.64500	8
19	2238.07941	1119.54335	746.69799	560.27531	H	839.43699	420.22213	280.48385	210.61471	7
20	2367.12201	1184.06464	789.71219	592.53596	E	702.37808	351.69268	234.79754	176.34998	6
21	2482.14895	1241.57811	828.05450	621.29269	D	573.33549	287.17138	191.78335	144.08933	5
22	2581.21736	1291.11232	861.07731	646.05980	V	458.30854	229.65791	153.44103	115.33259	4
23	2694.30143	1347.65435	898.77199	674.33081	I	359.24013	180.12370	120.41823	90.56549	3
24	2765.33854	1383.17291	922.45103	692.09009	A	246.15607	123.58167	82.72354	62.29447	2
25					R	175.11895	88.06311	59.04450	44.53520	1

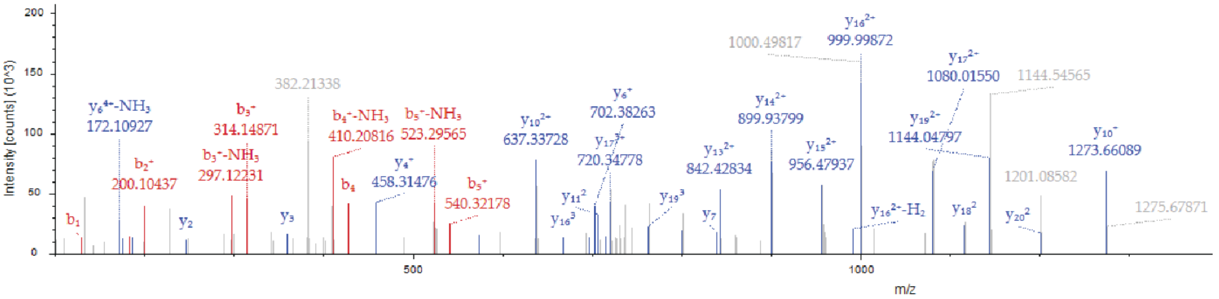

H

E L I E **K** M I A M D P Q K

K-811

#1	b⁺	b²⁺	Seq.	y⁺	y²⁺	#2
1	130.04987	65.52857	E			13
2	243.13393	122.07061	L	1496.78513	748.89620	12
3	356.21800	178.61264	I	1383.70107	692.35417	11
4	485.26059	243.13393	E	1270.61700	635.81214	10
5	693.38176	347.19452	K-SAS_NA...	1141.57441	571.29084	9
6	824.42225	412.71476	M	933.45324	467.23026	8
7	937.50631	469.25679	I	802.41275	401.71001	7
8	1008.54343	504.77535	A	689.32869	345.16798	6
9	1139.58391	570.29559	M	618.29157	309.64942	5
10	1254.61085	627.80906	D	487.25109	244.12918	4
11	1351.66362	676.33545	P	372.22415	186.61571	3
12	1479.72219	740.36474	Q	275.17138	138.08933	2
13			K	147.11280	74.06004	1

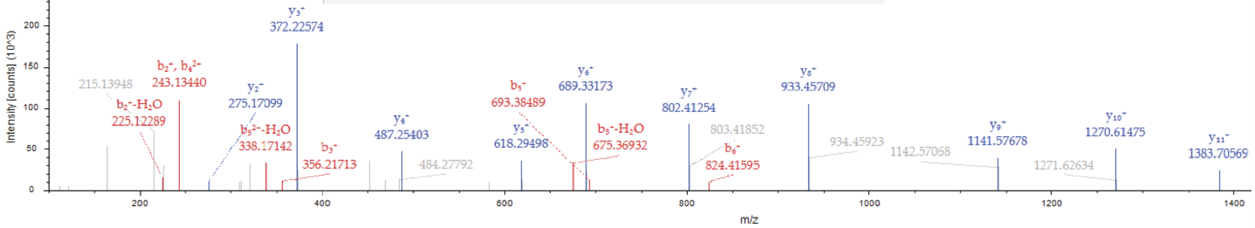

I

H V L **K** H P F F W S L E K

K-828

#1	b⁺	b²⁺	b³⁺	Seq.	y⁺	y²⁺	y³⁺	#2
1	138.06619	69.53673	46.69358	H				13
2	237.13460	119.07094	79.71639	V	1610.87296	805.94012	537.62917	12
3	350.21867	175.61297	117.41107	L	1511.80454	756.40591	504.60637	11
4	558.33984	279.67356	186.78480	K-SAS_NA...	1398.72048	699.86388	466.91168	10
5	695.39875	348.20301	232.47110	H	1190.59931	595.80329	397.53795	9
6	792.45151	396.72940	264.82202	P	1053.54039	527.27384	351.85165	8
7	939.51993	470.26360	313.84483	F	956.48763	478.74745	319.50073	7
8	1086.58834	543.79781	362.86763	F	809.41922	405.21325	270.47792	6
9	1272.66766	636.83747	424.89407	W	662.35080	331.67904	221.45512	5
10	1359.69968	680.35348	453.90475	S	476.27149	238.63938	159.42868	4
11	1472.78375	736.89551	491.59943	L	389.23946	195.12337	130.41800	3
12	1601.82634	801.41681	534.61363	E	276.15540	138.58134	92.72332	2
13				K	147.11280	74.06004	49.70912	1

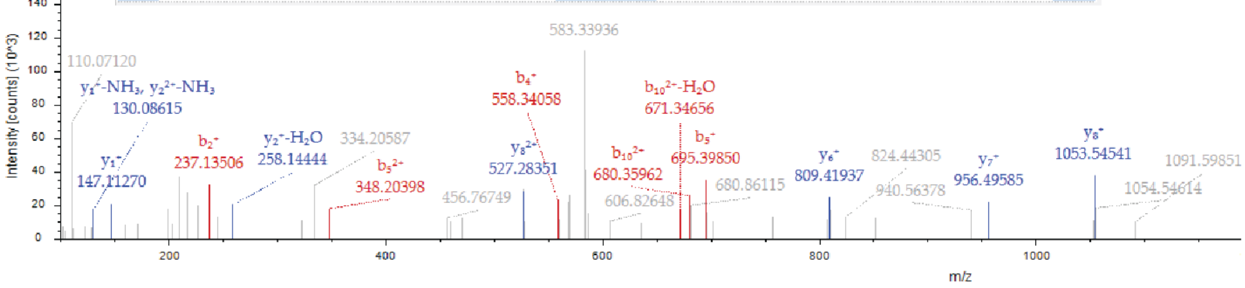

J

I E K E S L D G P I V K

K-851

#1	b⁺	b²⁺	Seq.	y⁺	y²⁺	#2
1	114.09134	57.54931	I			12
2	243.13393	122.07061	E	1294.68890	647.84809	11
3	451.25511	226.13119	K-SAS_NA...	1165.64631	583.32679	10
4	580.29770	290.65249	E	957.52514	479.26621	9
5	667.32973	334.16850	S	828.48254	414.74491	8
6	780.41379	390.71053	L	741.45052	371.22890	7
7	895.44073	448.22401	D	628.36645	314.68686	6
8	952.46220	476.73474	G	513.33951	257.17339	5
9	1049.51496	525.26112	P	456.31805	228.66266	4
10	1162.59903	581.80315	I	359.26528	180.13628	3
11	1261.66744	631.33736	V	246.18122	123.59425	2
12			K	147.11280	74.06004	1

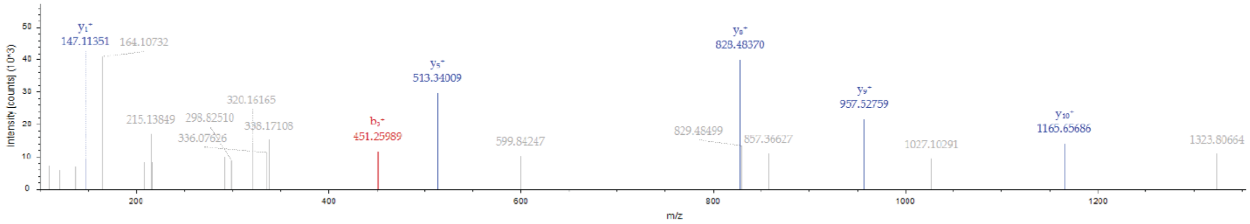

K

E S L D G P I V K Q L E R

K-860

#1	b⁺	b²⁺	Seq.	y⁺	y²⁺	#2
1	130.04987	65.52857	E			13
2	217.08190	109.04459	S	1434.78510	717.90119	12
3	330.16596	165.58662	L	1347.76307	674.38517	11
4	445.19290	223.10009	D	1234.67901	617.84314	10
5	502.21437	251.61082	G	1119.65206	560.32967	9
6	599.26713	300.13720	P	1062.63060	531.81894	8
7	712.35120	356.67924	I	965.57784	483.29256	7
8	811.41961	406.21344	V	852.49377	426.75053	6
9	1019.54078	510.27403	K-SAS_NA...	753.42536	377.21632	5
10	1147.59936	574.30332	Q	545.30419	273.15573	4
11	1260.68342	630.84535	L	417.24561	209.12644	3
12	1389.72602	695.36665	E	304.16155	152.58441	2
13			R	175.11895	88.06311	1

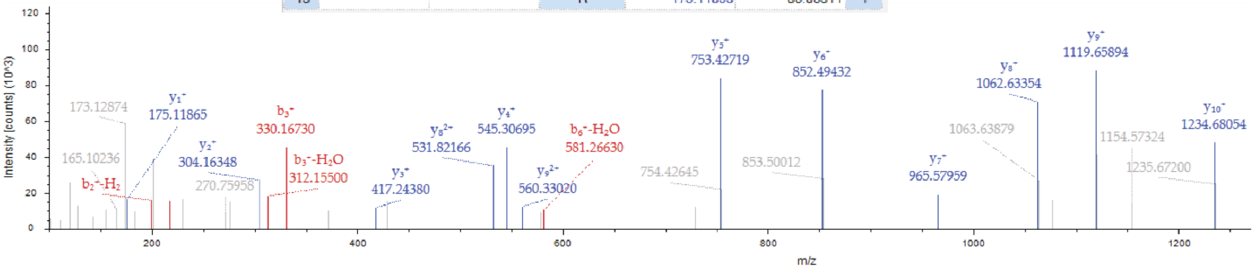

L

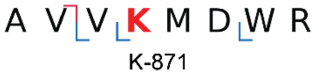

#1	b⁺	b²⁺	Seq.	y⁺	y²⁺	#2
1	72.04439	36.52583	A			8
2	171.11280	86.06004	V	1029.51861	515.26294	7
3	270.18122	135.59425	V	930.45019	465.72874	6
4	478.30239	239.65483	K-SAS_NA...	831.38178	416.19453	5
5	625.33779	313.17253	M-Oxidation	623.26061	312.13394	4
6	740.36473	370.68601	D	476.22521	238.61624	3
7	926.44405	463.72566	W	361.19827	181.10277	2
8			R	175.11895	88.06311	1

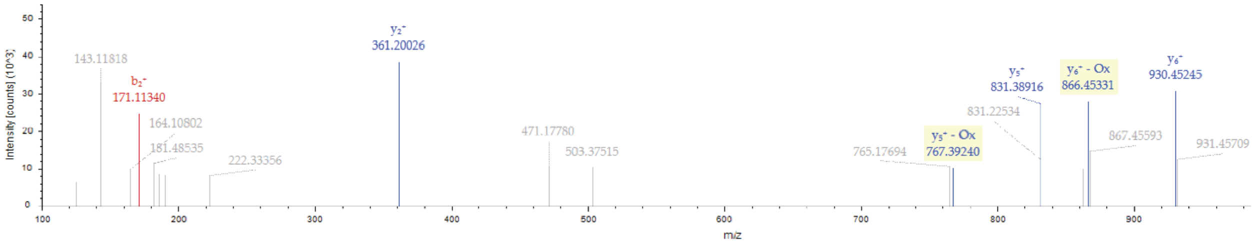

Figure S5. (A) IRE1α-nitrile NASA probe **3** labels multiple lysines near the binding pocket. **(B)** Crystal structure of IRE1α showing multiple lysines (blue sphere) labelled by **3**, IRE1α (PDB:4U6R). **(C-L)** MS/MS spectrum of IRE1α labeled peptides with **3**.

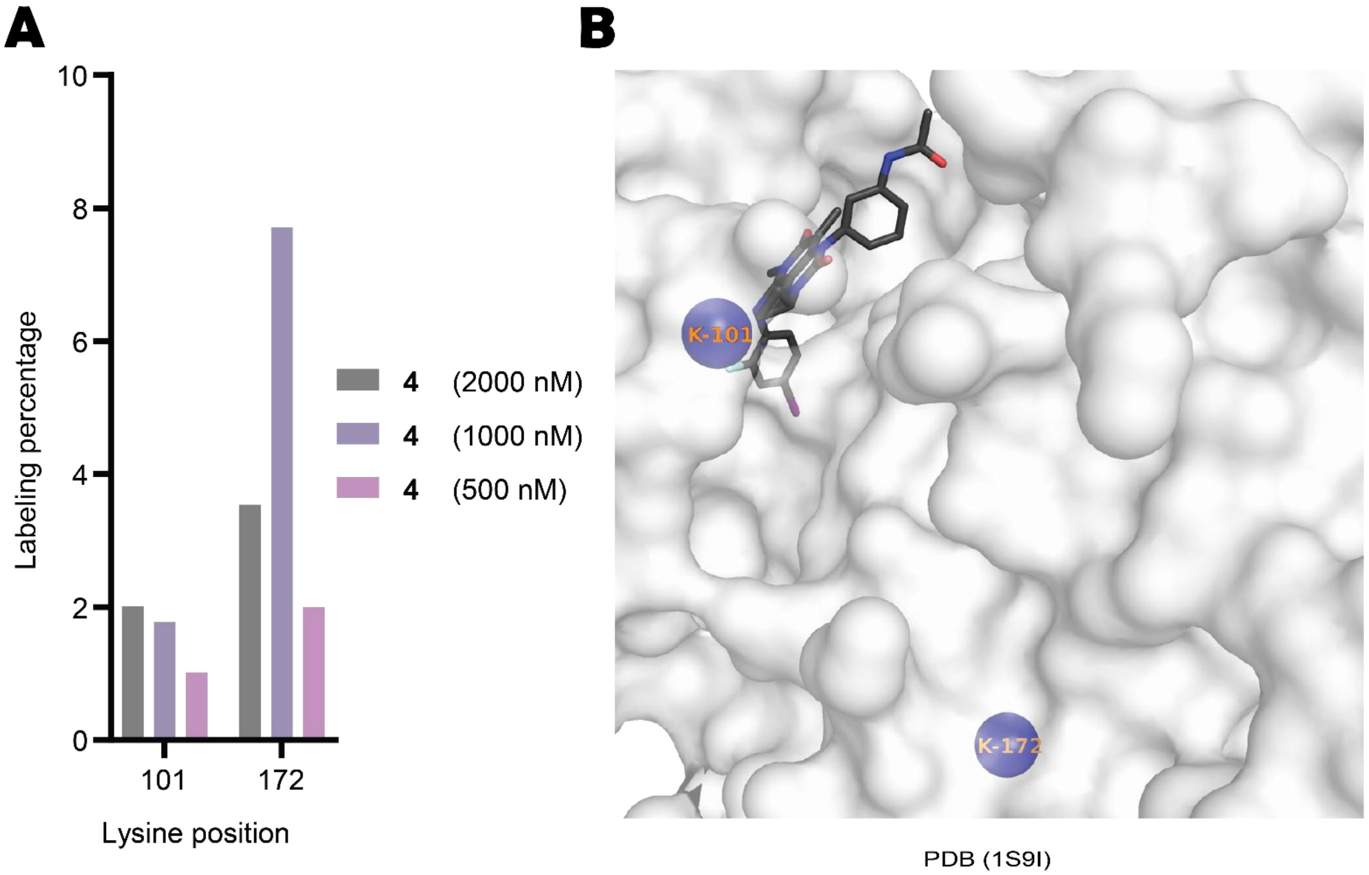

PDB (1S9I)

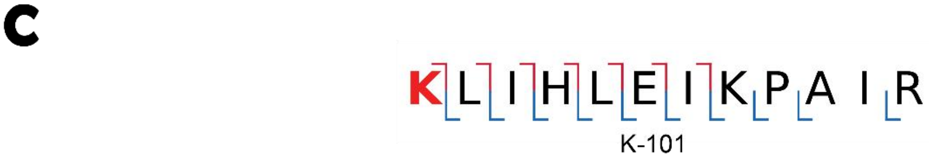

#1	b⁺	b²⁺	b³⁺	Seq.	y⁺	y²⁺	y³⁺	#2
1	223.14410	112.07569	75.05288	K-NASA_6...				12
2	336.22816	168.61772	112.74757	L	1302.82562	651.91645	434.94672	11
3	449.31223	225.15975	150.44226	I	1189.74155	595.37441	397.25204	10
4	586.37114	293.68921	196.12856	H	1076.65749	538.83238	359.55735	9
5	699.45520	350.23124	233.82325	L	939.59858	470.30293	313.87104	8
6	828.49780	414.75254	276.83745	E	826.51451	413.76090	276.17636	7
7	941.58186	471.29457	314.53214	I	697.47192	349.23960	233.16216	6
8	1069.67682	535.34205	357.23046	K	584.38786	292.69757	195.46747	5
9	1166.72959	583.86843	389.58138	P	456.29289	228.65009	152.76915	4
10	1237.76670	619.38699	413.26042	A	359.24013	180.12370	120.41823	3
11	1350.85076	675.92902	450.95511	I	288.20302	144.60515	96.73919	2
12				R	175.11895	88.06311	59.04450	1

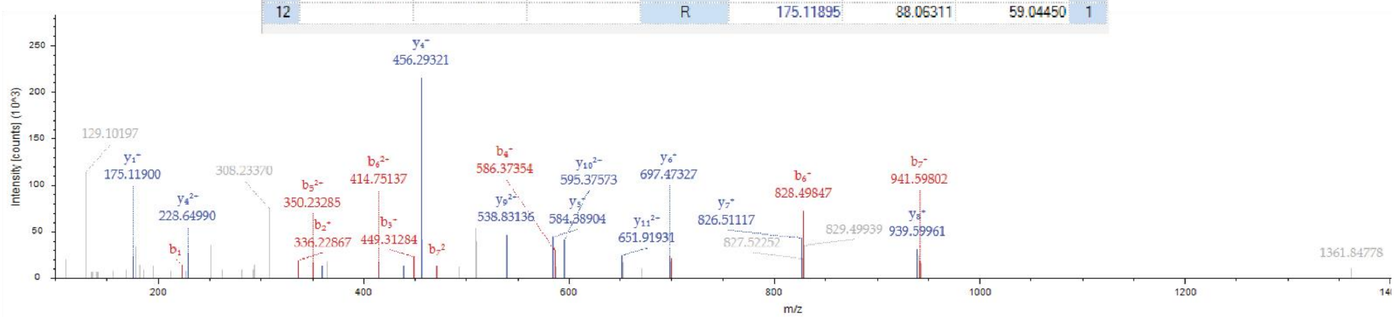

D

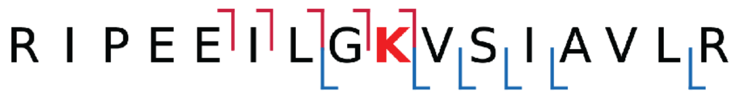

K-172

#1	b⁺	b²⁺	b³⁺	Seq.	y⁺	y²⁺	y³⁺	#2
1	157.10839	79.05783	53.04098	R				16
2	270.19245	135.59986	90.73567	I	1731.04148	866.02438	577.68534	15
3	367.24522	184.12625	123.08659	P	1617.95741	809.48235	539.99066	14
4	496.28781	248.64754	166.10079	E	1520.90465	760.95596	507.63973	13
5	625.33040	313.16884	209.11498	E	1391.86206	696.43467	464.62554	12
6	738.41447	369.71087	246.80967	I	1262.81946	631.91337	421.61134	11
7	851.49853	426.25290	284.50436	L	1149.73540	575.37134	383.91665	10
8	908.51999	454.76363	303.51152	G	1036.65134	518.82931	346.22196	9
9	1130.65682	565.83205	377.55712	K-NASA_6...	979.62987	490.31857	327.21481	8
10	1229.72523	615.36625	410.57993	V	757.49305	379.25016	253.16920	7
11	1316.75726	658.88227	439.59060	S	658.42464	329.71596	220.14640	6
12	1429.84132	715.42430	477.28529	I	571.39261	286.19994	191.13572	5
13	1500.87844	750.94286	500.96433	A	458.30854	229.65791	153.44103	4
14	1599.94685	800.47706	533.98713	V	387.27143	194.13935	129.76199	3
15	1713.03091	857.01910	571.68182	L	288.20302	144.60515	96.73919	2
16				R	175.11895	88.06311	59.04450	1

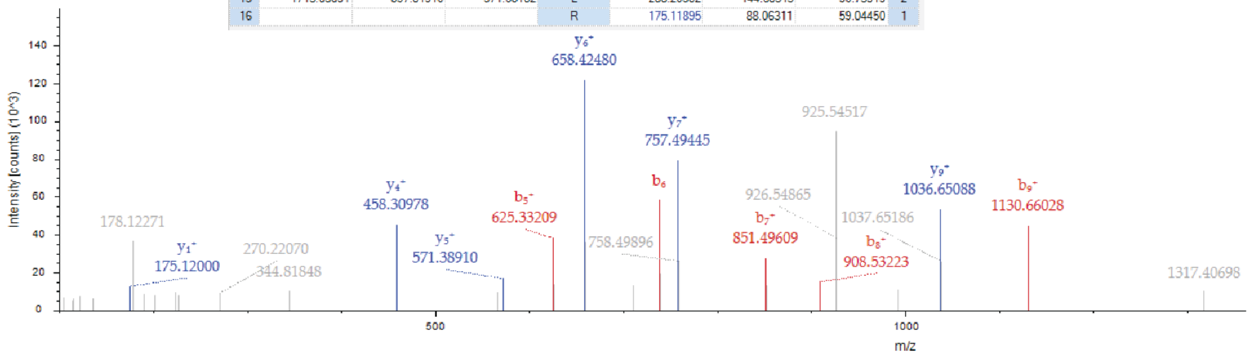

Figure S6. (A) Nitrile NASA probe **4** labels multiple lysines in MEK2 protein. **(B)** Crystal structure of MEK2 showing multiple lysines (blue sphere) labelled by **4**; MEK2 (PDB 1S9I) aligned with MEK1 (PDB 7JUR), ligand QOM shown as sticks. **(C-D)** MS/MS spectrum of MEK2 labeled peptides with **4**.

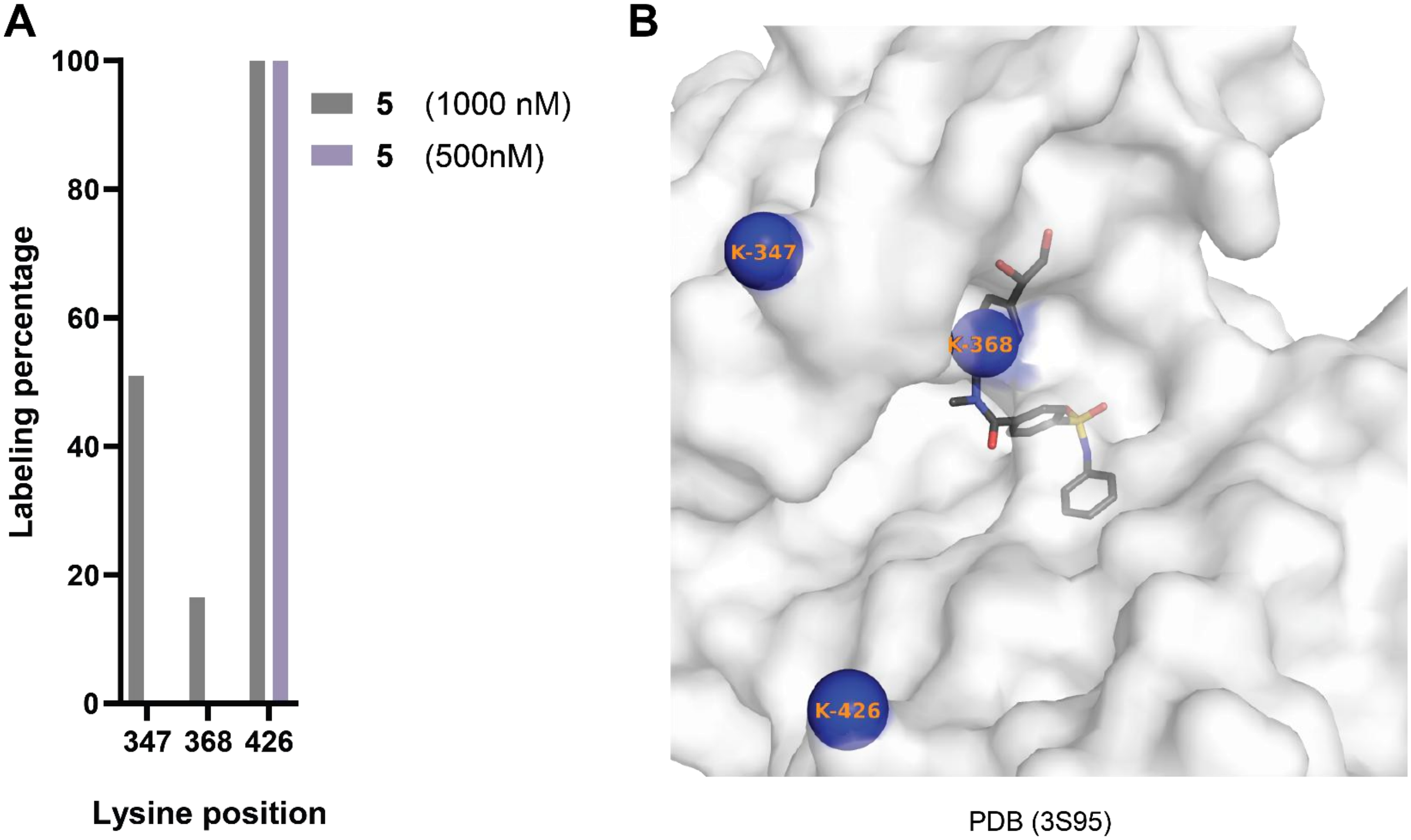

C

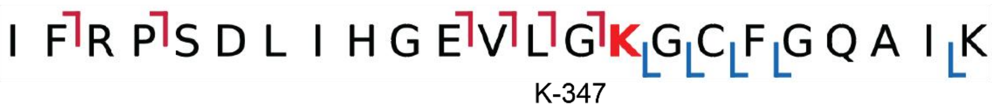

#1	b+	b2+	b3+	b4+	Seq.	y+	y2+	y3+	y4+	#2
1	114.09134	57.54931	38.70196	29.27829	I					23
2	261.15975	131.08352	87.72477	66.04540	F	2509.30202	1255.15465	837.10552	628.08096	22
3	417.26087	209.13407	139.75847	105.07067	R	2362.23361	1181.62044	788.08272	591.31386	21
4	514.31363	257.66045	172.10939	129.33386	P	2206.13250	1103.56989	736.04902	552.28858	20
5	601.34566	301.17647	201.12007	151.09187	S	2109.07973	1055.04350	703.69809	528.02539	19
6	716.37260	358.68994	239.46238	179.84861	D	2022.04770	1011.52749	674.68742	506.26738	18
7	829.45666	415.23197	277.15707	208.11962	L	1907.02076	954.01402	636.34510	477.51065	17
8	942.54073	471.77400	314.85176	236.39064	I	1793.93670	897.47199	598.65042	449.23963	16
9	1079.59964	540.30346	360.53806	270.65537	H	1680.85263	840.92995	560.95573	420.96862	15
10	1136.62110	568.81419	379.54522	284.91073	G	1543.79372	772.40050	515.26942	386.70389	14
11	1265.66370	633.33549	422.55942	317.17138	E	1486.77226	743.88977	496.26227	372.44852	13
12	1364.73211	682.86969	455.58222	341.93849	V	1357.72966	679.36847	453.24807	340.18787	12
13	1477.81617	739.41173	493.27691	370.20950	L	1258.66125	629.83426	420.22527	315.42077	11
14	1534.83764	767.92246	512.28406	384.46487	G	1145.57719	573.29223	382.53058	287.14975	10
15	1742.95881	871.98304	581.65779	436.49516	K-SAS_NA...	1088.55572	544.78150	363.52343	272.89439	9
16	1799.98028	900.49378	600.66494	450.75053	G	880.43455	440.72091	294.14970	220.86409	8
17	1960.01092	980.50910	654.00849	490.75819	C-Carbami...	823.41309	412.21018	275.14255	206.60873	7
18	2107.07934	1054.04331	703.03130	527.52529	F	663.38244	332.19486	221.79900	166.60107	6
19	2164.10080	1082.55404	722.03845	541.78066	G	516.31402	258.66065	172.77619	129.83396	5
20	2292.15938	1146.58333	764.72464	573.79530	Q	459.29256	230.14992	153.76904	115.57860	4
21	2363.19649	1182.10188	788.40368	591.55458	A	331.23398	166.12063	111.08285	83.56395	3
22	2476.28056	1238.64392	826.09837	619.82560	I	260.19687	130.60207	87.40381	65.80467	2
23					K	147.11280	74.06004	49.70912	37.53366	1

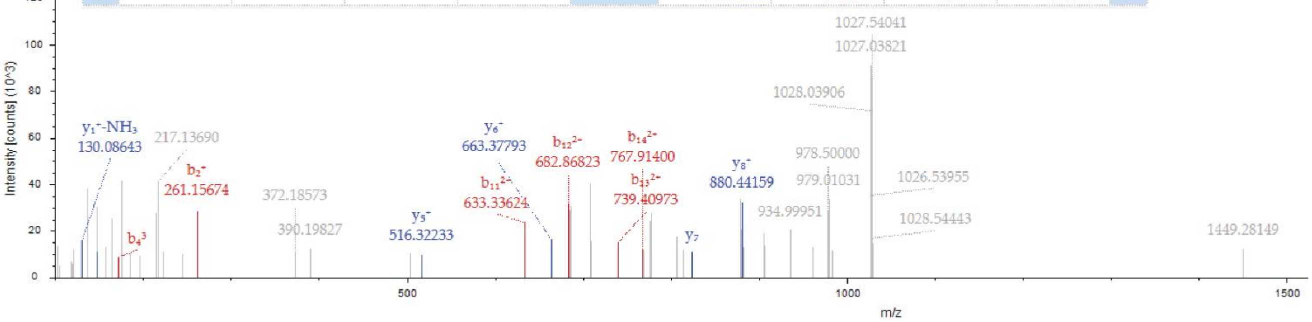

D

E T G E V M V M **K** E L I R

K-368

#1	b^+	b^{2+}	Seq.	y^+	y^{2+}	#2
1	130.04987	65.52857	E			13
2	231.09755	116.05241	T	1485.78038	743.39383	12
3	288.11901	144.56314	G	1384.73270	692.86999	11
4	417.16160	209.08444	E	1327.71124	664.35926	10
5	516.23002	258.61865	V	1198.66864	599.83796	9
6	647.27050	324.13889	M	1099.60023	550.30375	8
7	746.33892	373.67310	V	968.55974	484.78351	7
8	877.37940	439.19334	M	869.49133	435.24930	6
9	1085.50057	543.25393	K-SAS_NA...	738.45085	369.72906	5
10	1214.54317	607.77522	E	530.32967	265.66847	4
11	1327.62723	664.31725	L	401.28708	201.14718	3
12	1440.71130	720.85929	I	288.20302	144.60515	2
13			R	175.11895	88.06311	1

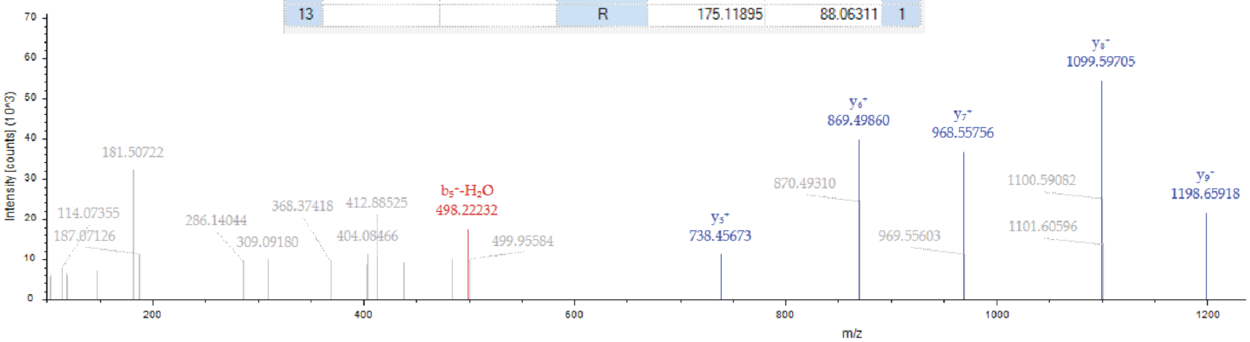

E

G I I **K** S M D S Q Y P W S Q R

K-426

#1	b^+	b^{2+}	Seq.	y^+	y^{2+}	#2
1	58.02874	29.51801	G			15
2	171.11280	86.06004	I	1818.88433	909.94580	14
3	284.19687	142.60207	I	1705.80026	853.40377	13
4	492.31804	246.66266	K-SAS_NA...	1592.71620	796.86174	12
5	579.35007	290.17867	S	1384.59502	692.80115	11
6	710.39055	355.69892	M	1297.56300	649.28514	10
7	825.41750	413.21239	D	1166.52251	583.76489	9
8	912.44953	456.72840	S	1051.49557	526.25142	8
9	1040.50810	520.75769	Q	964.46354	482.73541	7
10	1203.57143	602.28935	Y	836.40496	418.70612	6
11	1300.62420	650.81574	P	673.34163	337.17446	5
12	1486.70351	743.85539	W	576.28887	288.64807	4
13	1573.73554	787.37141	S	390.20956	195.60842	3
14	1701.79411	851.40070	Q	303.17753	152.09240	2
15			R	175.11895	88.06311	1

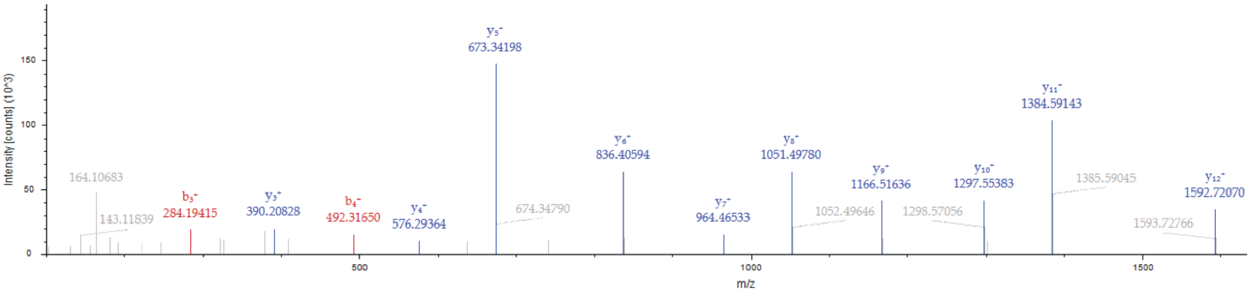

Figure S7. (A) LIMK1-nitrile NASA conjugate **5** labels multiple lysines near the binding pocket. **(B)** Crystal structure of LIMK1 showing multiple lysines (blue sphere) labelled by **5**; LIMK1 (PDB 3S95) aligned with LIMK2 (PDB 4TPT), ligand 35H shown as sticks. **(C-E)** MS/MS spectrum of LIMK1 labeled peptides with **5**.

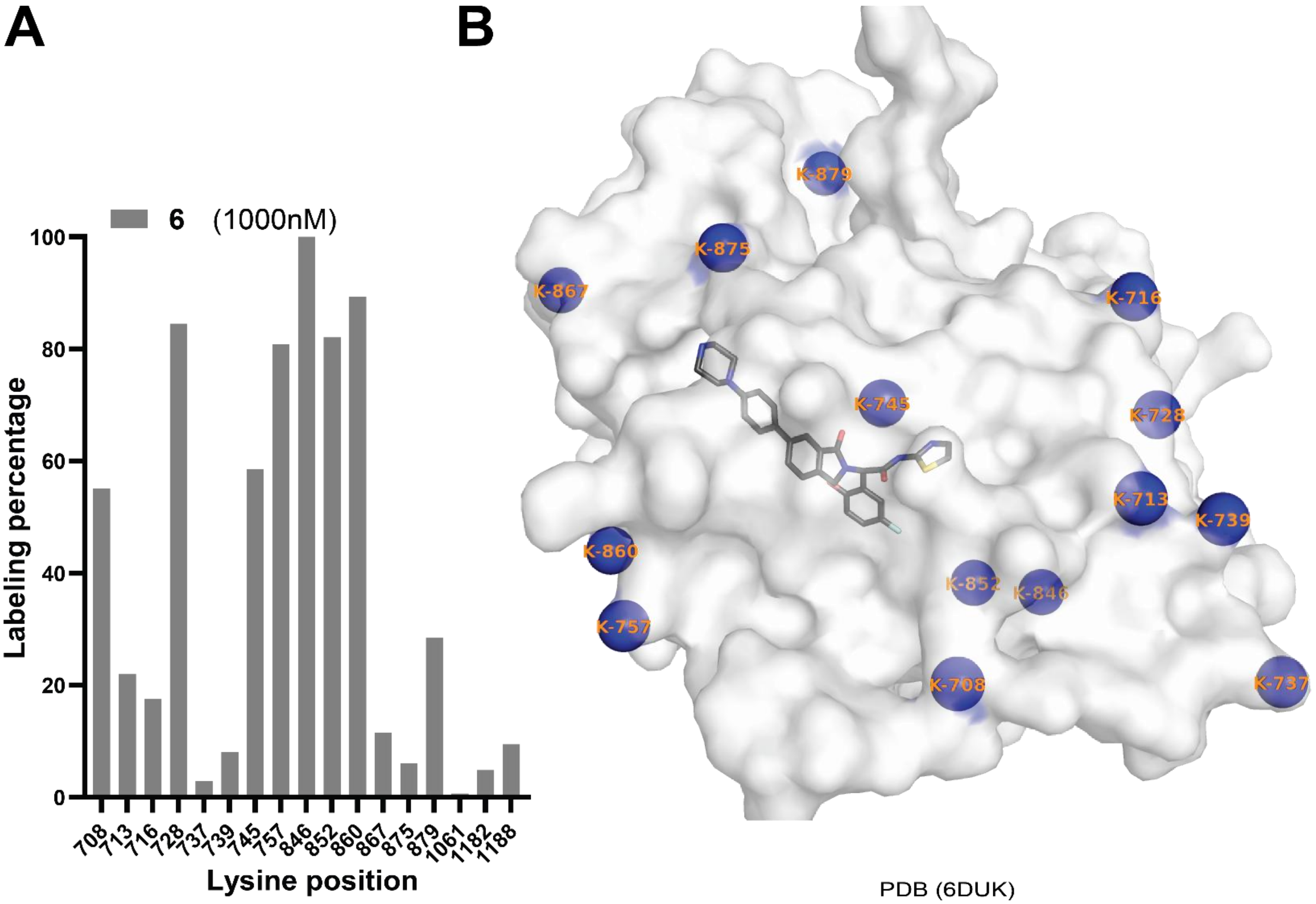

C

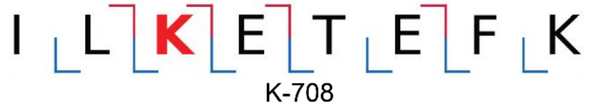

#1	b+	b2+	Seq.	y+	y2+	#2
1	114.09134	57.54931	I			8
2	227.17540	114.09134	L	988.53497	494.77112	7
3	449.31223	225.15975	K-NASA_6...	875.45091	438.22909	6
4	578.35482	289.68105	E	653.31408	327.16068	5
5	679.40250	340.20489	T	524.27149	262.63938	4
6	808.44509	404.72618	E	423.22381	212.11554	3
7	955.51351	478.26039	F	294.18122	147.59425	2
8			K	147.11280	74.06004	1

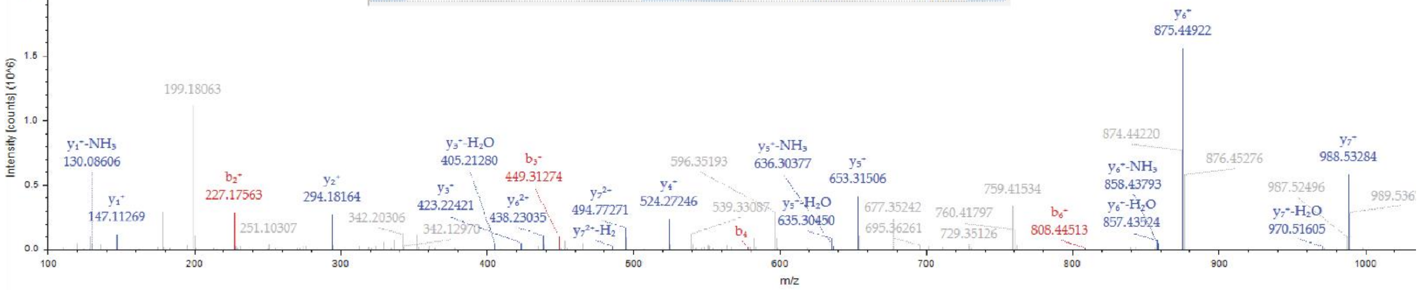

D

E T E F **K** K

K-713

#1	b⁺	b²⁺	Seq.	y⁺	y²⁺	#2
1	130.04987	65.52857	E			6
2	231.09755	116.05241	T	746.40831	373.70779	5
3	360.14014	180.57371	E	645.36063	323.18396	4
4	507.20855	254.10792	F	516.31804	258.66266	3
5	729.34538	365.17633	K-NASA_6...	369.24963	185.12845	2
6			K	147.11280	74.06004	1

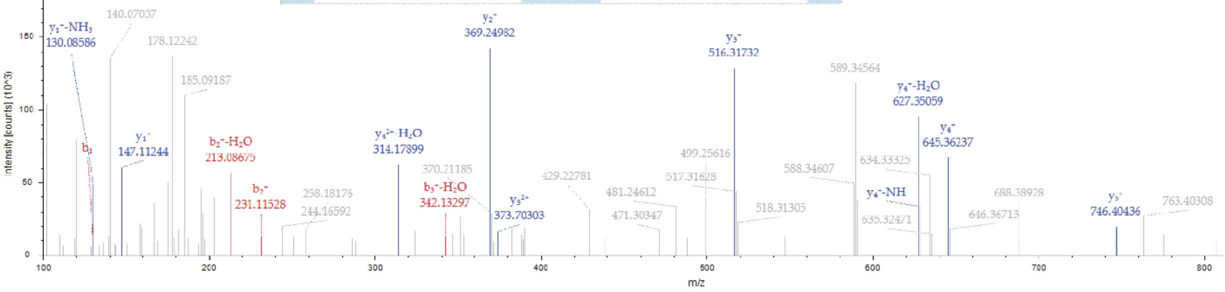

E

K I **K** V L G S G A F G T V Y K

K-716

#1	b⁺	b²⁺	b³⁺	Seq.	y⁺	y²⁺	y³⁺	#2
1	129.10224	65.05476	43.70560	K				15
2	242.18630	121.59679	81.40029	I	1533.86754	767.43741	511.96070	14
3	464.32313	232.66520	155.44589	K-NASA_6...	1420.78347	710.89537	474.26601	13
4	563.39154	282.19941	188.46870	V	1198.64665	599.82696	400.22040	12
5	676.47560	338.74144	226.16339	L	1099.57824	550.29276	367.19760	11
6	733.49707	367.25217	245.17054	G	986.49417	493.75072	329.50291	10
7	820.52910	410.76819	274.18122	S	929.47271	465.23999	310.49575	9
8	877.55056	439.27892	293.18837	G	842.44068	421.72398	281.48508	8
9	948.58767	474.79748	316.86741	A	785.41922	393.21325	262.47792	7
10	1095.65609	548.33168	365.89021	F	714.38210	357.69469	238.79889	6
11	1152.67755	576.84241	384.89737	G	567.31369	284.16048	189.77608	5
12	1253.72523	627.36625	418.57993	T	510.29223	255.64975	170.76893	4
13	1352.79364	676.90046	451.60273	V	409.24455	205.12591	137.08637	3
14	1515.85697	758.43212	505.95718	Y	310.17613	155.59170	104.06356	2
15				K	147.11280	74.06004	49.70912	1

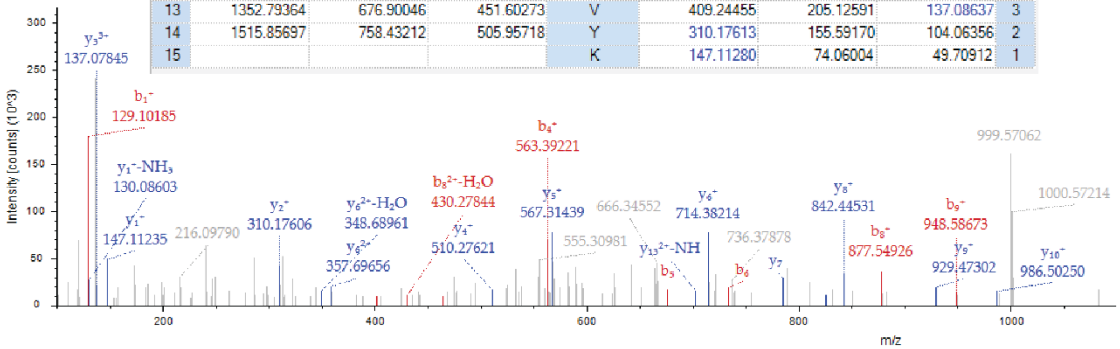

F

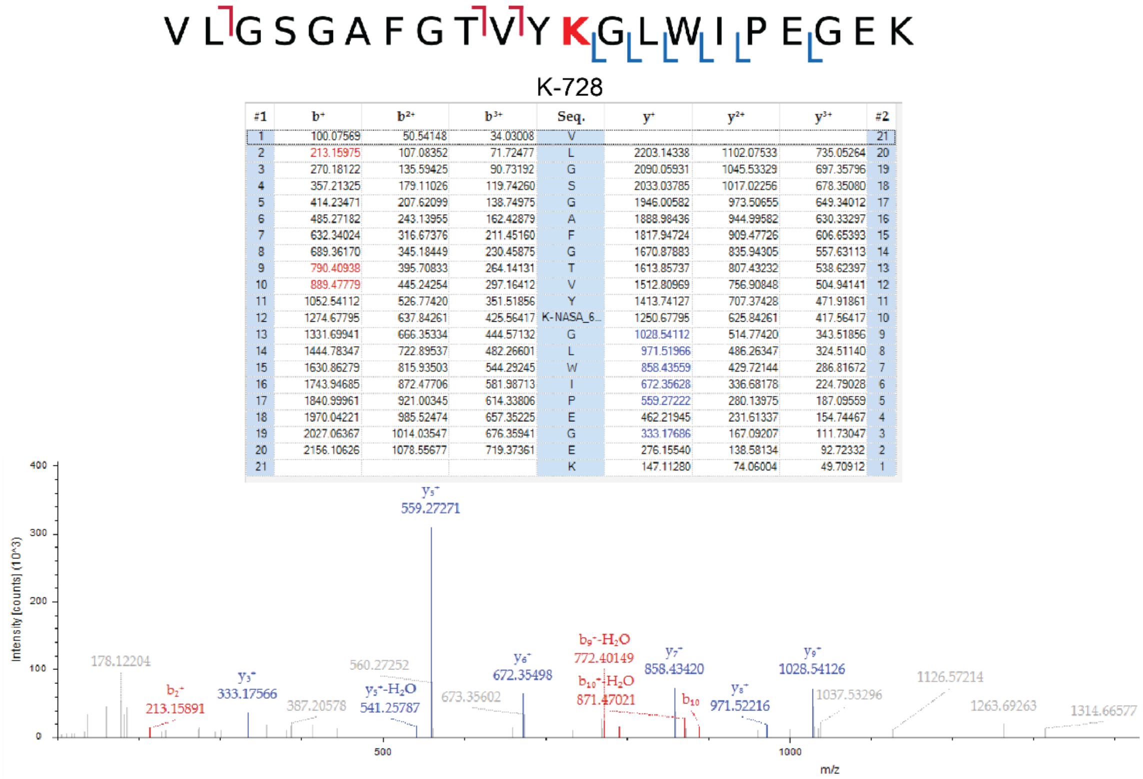

#1	b+	b2+	b3+	Seq.	y+	y2+	y3+	#2
1	100.07569	50.54148	34.03008	V				21
2	213.15975	107.08352	71.72477	L	2203.14338	1102.07533	735.05264	20
3	270.18122	135.59425	90.73192	G	2090.05931	1045.53329	697.35796	19
4	357.21325	179.11026	119.74260	S	2033.03785	1017.02256	678.35080	18
5	414.23471	207.62099	138.74975	G	1946.00582	973.50655	649.34012	17
6	485.27182	243.13955	162.42879	A	1888.98436	944.99582	630.33297	16
7	632.34024	316.67376	211.45160	F	1817.94724	909.47726	606.65393	15
8	689.36170	345.18449	230.45875	G	1670.87883	835.94305	557.63113	14
9	790.40938	395.70833	264.14131	T	1613.85737	807.43232	538.62397	13
10	889.47779	445.24254	297.16412	V	1512.80969	756.90848	504.94141	12
11	1052.54112	526.77420	351.51856	Y	1413.74127	707.37428	471.91861	11
12	1274.67795	637.84261	425.56417	K-NASA_6...	1250.67795	625.84261	417.56417	10
13	1331.69941	666.35334	444.57132	G	1028.54112	514.77420	343.51856	9
14	1444.78347	722.89537	482.26601	L	971.51966	486.26347	324.51140	8
15	1630.86279	815.93503	544.29245	W	858.43559	429.72144	286.81672	7
16	1743.94685	872.47706	581.98713	I	672.35628	336.68178	224.79028	6
17	1840.99961	921.00345	614.33806	P	559.27222	280.13975	187.09559	5
18	1970.04221	985.52474	657.35225	E	462.21945	231.61337	154.74467	4
19	2027.06367	1014.03547	676.35941	G	333.17686	167.09207	111.73047	3
20	2156.10626	1078.55677	719.37361	E	276.15540	138.58134	92.72332	2
21				K	147.11280	74.06004	49.70912	1

G

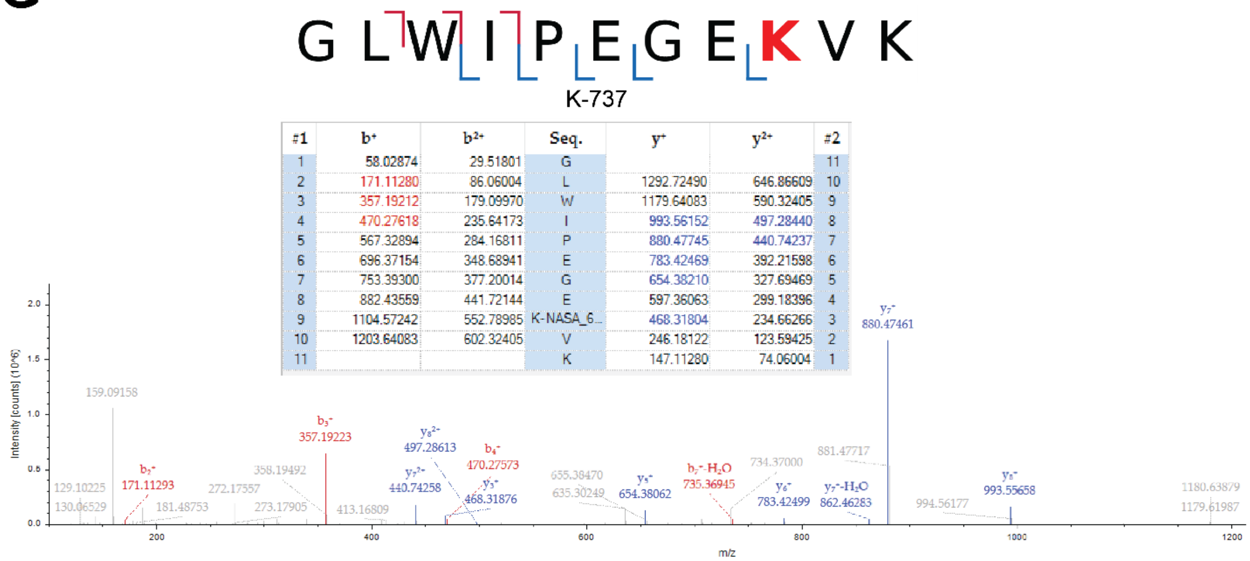

#1	b+	b2+	Seq.	y+	y2+	#2
1	58.02874	29.51801	G			11
2	171.11280	86.06004	L	1292.72490	646.86609	10
3	357.19212	179.09970	W	1179.64083	590.32405	9
4	470.27618	235.64173	I	993.56152	497.28440	8
5	567.32894	284.16811	P	880.47745	440.74237	7
6	696.37154	348.68941	E	783.42469	392.21598	6
7	753.39300	377.20014	G	654.38210	327.69469	5
8	882.43559	441.72144	E	597.36063	299.18396	4
9	1104.57242	552.78985	K-NASA_6...	468.31804	234.66266	3
10	1203.64083	602.32405	V	246.18122	123.59425	2
11			K	147.11280	74.06004	1

H

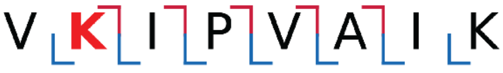

#1	b⁺	b²⁺	Seq.	y⁺	y²⁺	#2
1	100.07569	50.54148	V			8
2	322.21251	161.60990	K-NASA_6...	862.57605	431.79166	7
3	435.29658	218.15193	I	640.43922	320.72325	6
4	532.34934	266.67831	P	527.35516	264.18122	5
5	631.41776	316.21252	V	430.30240	215.65484	4
6	702.45487	351.73107	A	331.23398	166.12063	3
7	815.53893	408.27310	I	260.19687	130.60207	2
8			K	147.11280	74.06004	1

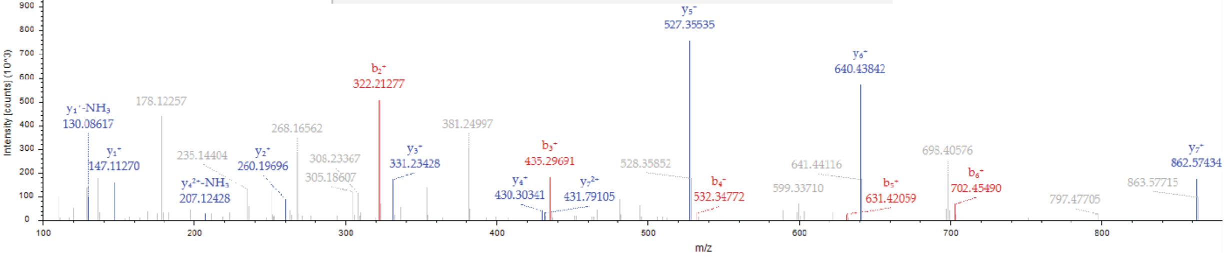

I

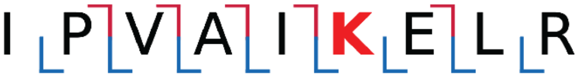

#1	b⁺	b²⁺	Seq.	y⁺	y²⁺	#2
1	114.09134	57.54931	I			9
2	211.14410	106.07569	P	1019.62479	510.31603	8
3	310.21252	155.60990	V	922.57202	461.78965	7
4	381.24963	191.12845	A	823.50361	412.25544	6
5	494.33370	247.67049	I	752.46650	376.73689	5
6	716.47052	358.73890	K-NASA_6...	639.38243	320.19485	4
7	845.51311	423.26019	E	417.24561	209.12644	3
8	958.59718	479.80223	L	288.20302	144.60515	2
9			R	175.11895	88.06311	1

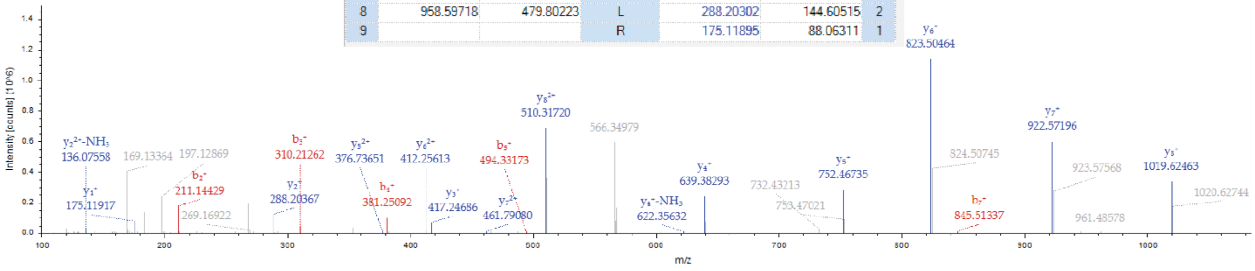

J

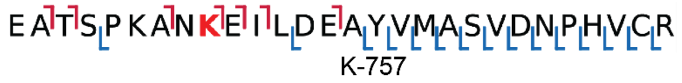

#1	b+	b2+	b3+	b4+	Seq.	y+	y2+	y3+	y4+	#2
1	130.04987	65.52857	44.02147	33.26792	E					28
2	201.08698	101.04713	67.70051	51.02720	A	3109.50813	1555.25770	1037.17423	778.13249	27
3	302.13466	151.57097	101.38307	76.28912	T	3038.47101	1519.73914	1013.49519	760.37321	26
4	389.16669	195.08698	130.39375	98.04713	S	2937.42333	1469.21530	979.81263	735.11129	25
5	486.21945	243.61337	162.74467	122.31032	P	2850.39130	1425.69929	950.80195	713.35328	24
6	614.31442	307.66085	205.44299	154.33406	K	2753.33854	1377.17291	918.45103	689.09009	23
7	685.35153	343.17940	229.12203	172.09334	A	2625.24358	1313.12543	875.75271	657.06635	22
8	799.39446	400.20087	267.13634	200.60407	N	2554.20646	1277.60687	852.07367	639.30707	21
9	1021.53128	511.26928	341.18194	256.13828	K-NASA_6...	2440.16354	1220.58541	814.05936	610.79634	20
10	1150.57387	575.79058	384.19614	288.39893	E	2218.02671	1109.51700	740.01376	555.26214	19
11	1263.65794	632.33261	421.89083	316.66994	I	2088.98412	1044.99570	696.99956	523.00149	18
12	1376.74200	688.87464	459.58552	344.94096	L	1975.90006	988.45367	659.30487	494.73047	17
13	1491.76894	746.38811	497.92783	373.69769	D	1862.81599	931.91163	621.61018	466.45946	16
14	1620.81154	810.90941	540.94203	405.95834	E	1747.78905	874.39816	583.26787	437.70272	15
15	1691.84865	846.42796	564.62107	423.71762	A	1618.74646	809.87687	540.25367	405.44207	14
16	1854.91198	927.95963	618.97551	464.48345	Y	1547.70934	774.35831	516.57463	387.68279	13
17	1953.98039	977.49384	651.99832	489.25056	V	1384.64601	692.82665	462.22019	346.91696	12
18	2085.02088	1043.01408	695.67848	522.01068	M	1285.57760	643.29244	429.19738	322.14986	11
19	2156.05799	1078.53263	719.35752	539.76996	A	1154.53712	577.77220	385.51722	289.38974	10
20	2243.09002	1122.04865	748.36819	561.52796	S	1083.50000	542.25364	361.83819	271.63046	9
21	2342.15843	1171.58286	781.39100	586.29507	V	996.46797	498.73763	332.82751	249.87245	8
22	2457.18538	1229.09633	819.73331	615.05180	D	897.39956	449.20342	299.80470	225.10535	7
23	2571.22830	1286.11779	857.74762	643.56253	N	782.37262	391.68995	261.46239	196.34861	6
24	2668.28107	1334.64417	890.09854	667.82572	P	668.32969	334.66848	223.44808	167.83788	5
25	2805.33998	1403.17363	935.78484	702.09045	H	571.27693	286.14210	191.09716	143.57469	4
26	2904.40839	1452.70784	968.80765	726.85756	V	434.21801	217.61265	145.41086	109.30996	3
27	3064.43904	1532.72316	1022.15120	766.86522	C-Carbami...	335.14960	168.07844	112.38805	84.54286	2
28					R	175.11895	88.06311	59.04450	44.53520	1

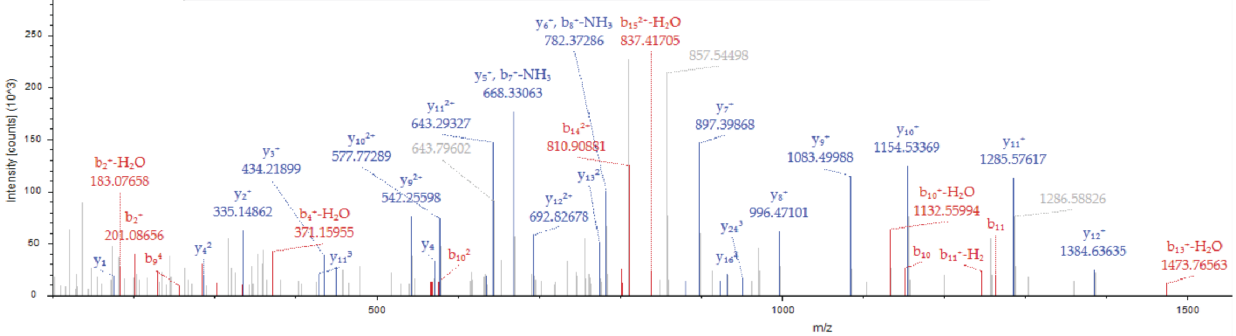

K

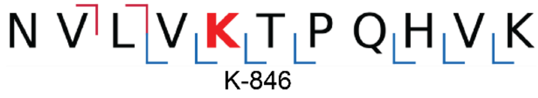

#1	b+	b2+	b3+	Seq.	y+	y2+	y3+	#2
1	115.05020	58.02874	39.02159	N				11
2	214.11862	107.56295	72.04439	V	1242.75686	621.88207	414.92381	10
3	327.20268	164.10498	109.73908	L	1143.68845	572.34786	381.90100	9
4	426.27110	213.63919	142.76188	V	1030.60439	515.80583	344.20631	8
5	648.40792	324.70760	216.80749	K-NASA_6...	931.53597	466.27162	311.18351	7
6	749.45560	375.23144	250.49005	T	709.39915	355.20321	237.13790	6
7	846.50836	423.75782	282.84097	P	608.35147	304.67937	203.45534	5
8	974.56694	487.78711	325.52716	Q	511.29871	256.15299	171.10442	4
9	1111.62585	556.31656	371.21347	H	383.24013	192.12370	128.41823	3
10	1210.69426	605.85077	404.23627	V	246.18122	123.59425	82.73192	2
11				K	147.11280	74.06004	49.70912	1

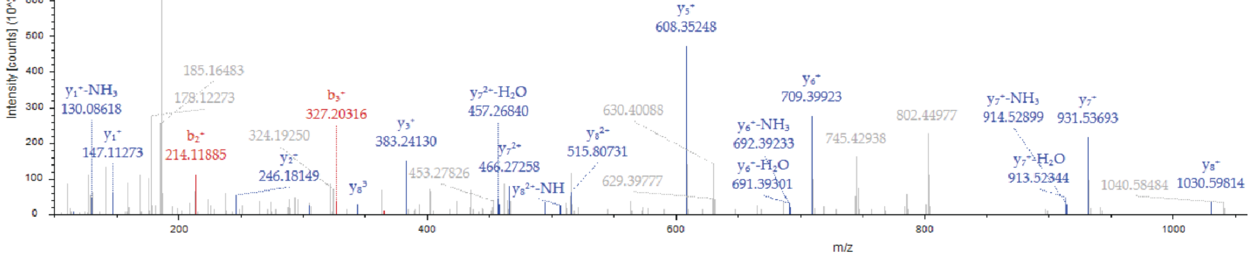

L

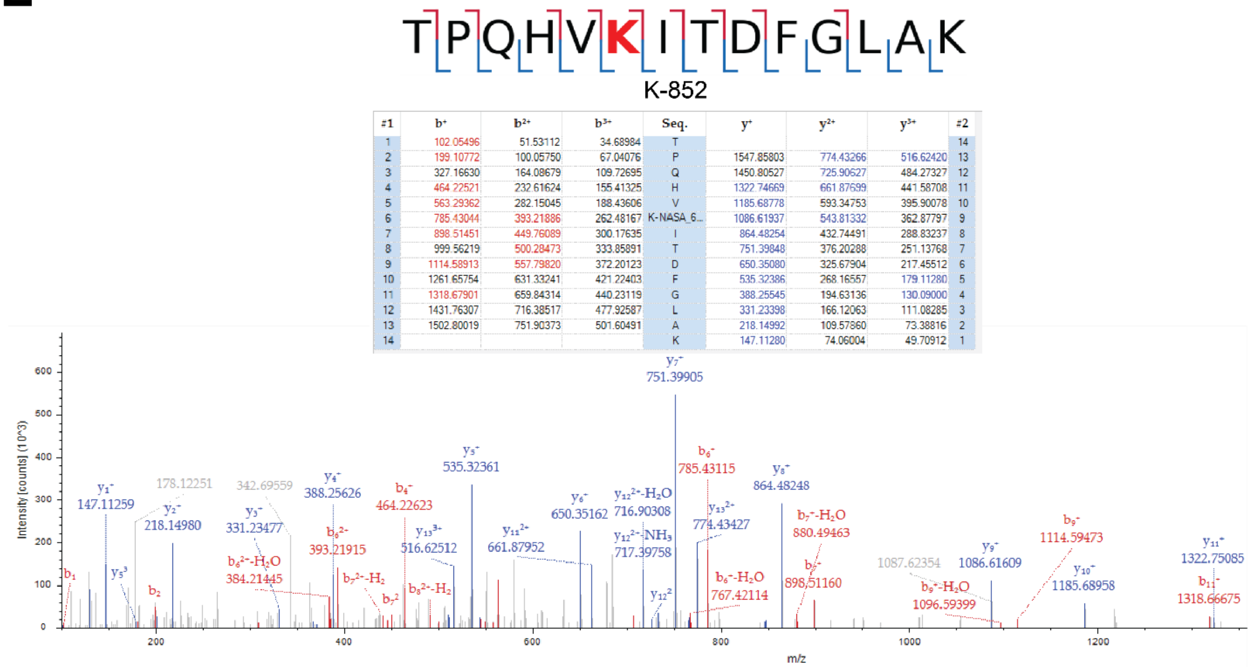

#1	b⁺	b²⁺	b³⁺	Seq.	y⁺	y²⁺	y³⁺	#2
1	102.05496	51.53112	34.68984	T				14
2	199.10772	100.05750	67.04076	P	1547.85803	774.43266	516.62420	13
3	327.16630	164.08679	109.72695	Q	1450.80527	725.90627	484.27327	12
4	464.22521	232.61624	155.41325	H	1322.74669	661.87699	441.58708	11
5	563.29362	282.15045	188.43606	V	1185.68778	593.34753	395.90078	10
6	785.43044	393.21886	262.48167	K-NASA_6...	1086.61937	543.81332	362.87797	9
7	898.51451	449.76089	300.17635	I	864.48254	432.74491	288.83237	8
8	999.56219	500.28473	333.85891	T	751.39848	376.20288	251.13768	7
9	1114.58913	557.79820	372.20123	D	650.35080	325.67904	217.45512	6
10	1261.65754	631.33241	421.22403	F	535.32386	268.16557	179.11280	5
11	1318.67901	659.84314	440.23119	G	388.25545	194.63136	130.09000	4
12	1431.76307	716.38517	477.92587	L	331.23398	166.12063	111.08285	3
13	1502.80019	751.90373	501.60491	A	218.14992	109.57860	73.38816	2
14				K	147.11280	74.06004	49.70912	1

M

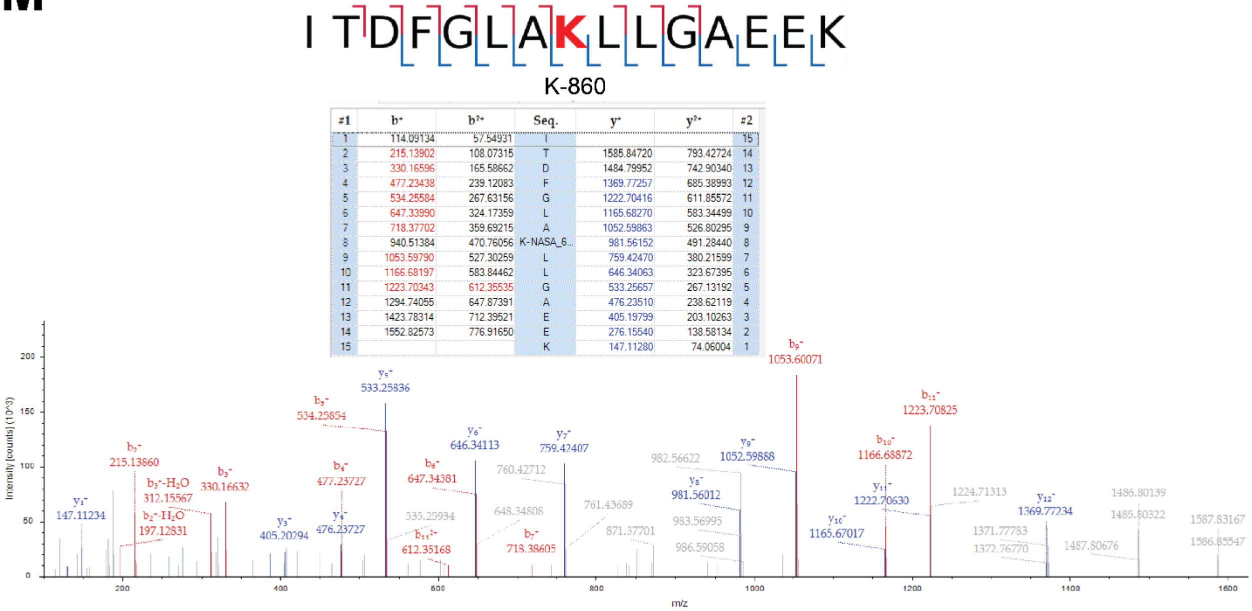

#1	b⁺	b²⁺	Seq.	y⁺	y²⁺	#2
1	114.09134	57.54931	I			15
2	215.13902	108.07315	T	1585.84720	793.42724	14
3	330.16596	165.58662	D	1484.79952	742.90340	13
4	477.23438	239.12083	F	1369.77257	685.38993	12
5	534.25584	267.63156	G	1222.70416	611.85572	11
6	647.33990	324.17359	L	1165.68270	583.34499	10
7	718.37702	359.69215	A	1052.59863	526.80295	9
8	940.51384	470.76056	K-NASA_6...	981.56152	491.28440	8
9	1053.59790	527.30259	L	759.42470	380.21599	7
10	1166.68197	583.84462	L	646.34063	323.67395	6
11	1223.70343	612.35535	G	533.25657	267.13192	5
12	1294.74055	647.87391	A	476.23510	238.62119	4
13	1423.78314	712.39521	E	405.19799	203.10263	3
14	1552.82573	776.91650	E	276.15540	138.58134	2
15			K	147.11280	74.06004	1

N

L L G A E E **K** E Y H A E G G K

K-867

#1	b⁺	b²⁺	b³⁺	Seq.	y⁺	y²⁺	y³⁺	#2
1	114.09134	57.54931	38.70196	L				15
2	227.17540	114.09134	76.39665	L	1611.76492	806.38610	537.92649	14
3	284.19687	142.60207	95.40381	G	1498.68086	749.84407	500.23180	13
4	355.23398	178.12063	119.08285	A	1441.65939	721.33334	481.22465	12
5	484.27657	242.64193	162.09704	E	1370.62228	685.81478	457.54561	11
6	613.31917	307.16322	205.11124	E	1241.57969	621.29348	414.53141	10
7	835.45599	418.23163	279.15685	K-NASA_6...	1112.53709	556.77219	371.51722	9
8	964.49858	482.75293	322.17105	E	890.40027	445.70377	297.47161	8
9	1127.56191	564.28459	376.52549	Y	761.35768	381.18248	254.45741	7
10	1264.62082	632.81405	422.21179	H	598.29435	299.65081	200.10297	6
11	1335.65794	668.33261	445.89083	A	461.23544	231.12136	154.41666	5
12	1464.70053	732.85390	488.90503	E	390.19832	195.60280	130.73763	4
13	1521.72199	761.36464	507.91218	G	261.15573	131.08150	87.72343	3
14	1578.74346	789.87537	526.91934	G	204.13427	102.57077	68.71627	2
15				K	147.11280	74.06004	49.70912	1

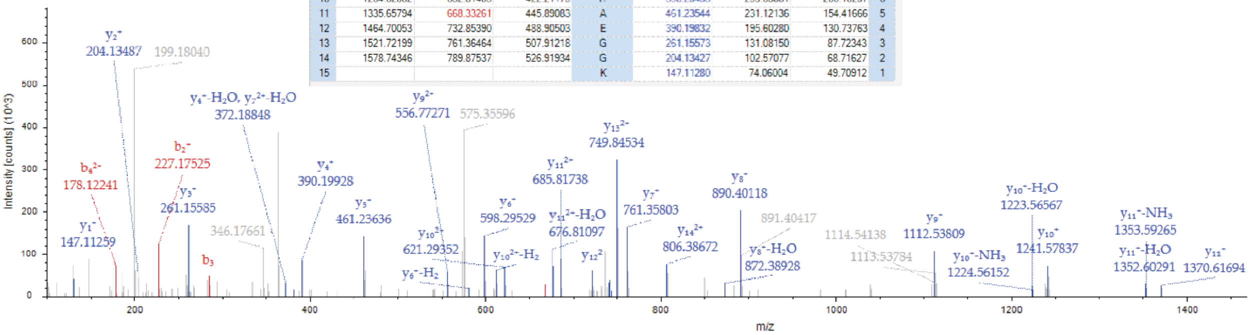

O

E Y H A E G G K V P I K

K-875

#1	b⁺	b²⁺	b³⁺	Seq.	y⁺	y²⁺	y³⁺	#2
1	130.04987	65.52857	44.02147	E				12
2	293.11320	147.06024	98.37592	Y	1292.69974	646.85351	431.57143	11
3	430.17211	215.58969	144.06222	H	1129.63641	565.32185	377.21699	10
4	501.20922	251.10825	167.74126	A	992.57750	496.79239	331.53069	9
5	630.25182	315.62955	210.75546	E	921.54039	461.27383	307.85165	8
6	687.27328	344.14028	229.76261	G	792.49780	396.75254	264.83745	7
7	744.29474	372.65101	248.76977	G	735.47633	368.24180	245.83030	6
8	966.43157	483.71942	322.81537	K-NASA_6...	678.45487	339.73107	226.82314	5
9	1065.49998	533.25363	355.83818	V	456.31805	228.66266	152.77753	4
10	1162.55274	581.78001	388.18910	P	357.24963	179.12845	119.75473	3
11	1275.63681	638.32204	425.88379	I	260.19687	130.60207	87.40381	2
12				K	147.11280	74.06004	49.70912	1

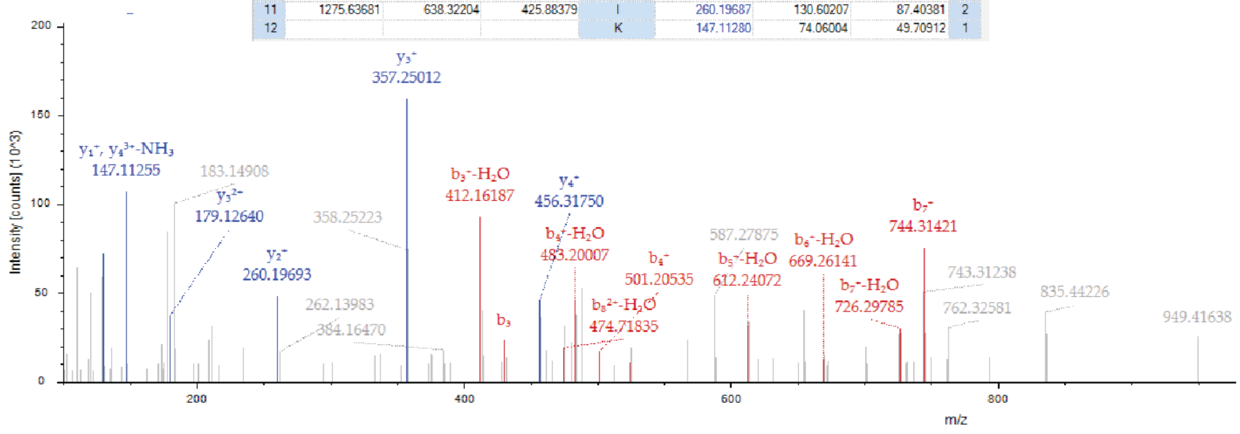

P

V P I **K** W M A L E S I L H R

K-879

#1	b+	b2+	b3+	Seq.	y+	y2+	y3+	#2
1	100.07569	50.54148	34.03008	V				14
2	197.12845	99.06787	66.38100	P	1687.93524	844.47126	563.31660	13
3	310.21252	155.60990	104.07569	I	1590.88248	795.94488	530.96568	12
4	532.34934	266.67831	178.12130	K-NASA_6...	1477.79841	739.40284	493.27099	11
5	718.42865	359.71797	240.14774	W	1255.66159	628.33443	419.22538	10
6	849.46914	425.23821	283.82790	M	1069.58228	535.29478	357.19894	9
7	920.50625	460.75676	307.50694	A	938.54179	469.77453	313.51878	8
8	1033.59032	517.29880	345.20162	L	867.50468	434.25598	289.83974	7
9	1162.63291	581.82009	388.21582	E	754.42061	377.71394	252.14506	6
10	1249.66494	625.33611	417.22650	S	625.37802	313.19265	209.13086	5
11	1362.74900	681.87814	454.92118	I	538.34599	269.67663	180.12018	4
12	1475.83307	738.42017	492.61587	L	425.26193	213.13460	142.42549	3
13	1612.89198	806.94963	538.30218	H	312.17786	156.59257	104.73081	2
14				R	175.11895	88.06311	59.04450	1

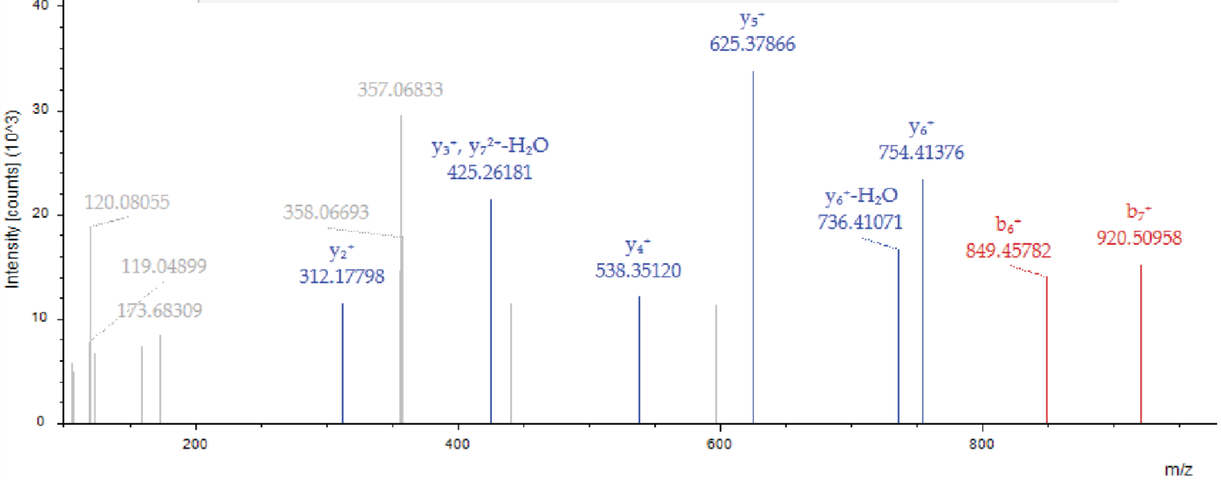

Q

N G L Q S C P I **K** E D S F L Q R

K-1061

#1	b⁺	b²⁺	b³⁺	Seq.	y⁺	y²⁺	y³⁺	#2
1	115.05020	58.02874	39.02159	N				16
2	172.07167	86.53947	58.02874	G	1871.93200	936.46964	624.64885	15
3	285.15573	143.08150	95.72343	L	1814.91054	907.95891	605.64170	14
4	413.21431	207.11079	138.40962	Q	1701.82648	851.41688	567.94701	13
5	500.24634	250.62681	167.42030	S	1573.76790	787.38759	525.26082	12
6	660.27699	330.64213	220.76385	C-Carbami...	1486.73587	743.87157	496.25014	11
7	757.32975	379.16851	253.11477	P	1326.70522	663.85625	442.90659	10
8	870.41381	435.71055	290.80946	I	1229.65246	615.32987	410.55567	9
9	1092.55064	546.77896	364.85506	K-NASA_6...	1116.56839	558.78784	372.86098	8
10	1221.59323	611.30025	407.86926	E	894.43157	447.71942	298.81538	7
11	1336.62017	668.81372	446.21158	D	765.38898	383.19813	255.80118	6
12	1423.65220	712.32974	475.22225	S	650.36204	325.68466	217.45886	5
13	1570.72061	785.86395	524.24506	F	563.33001	282.16864	188.44819	4
14	1683.80468	842.40598	561.93974	L	416.26159	208.63444	139.42538	3
15	1811.86326	906.43527	604.62594	Q	303.17753	152.09240	101.73069	2
16				R	175.11895	88.06311	59.04450	1

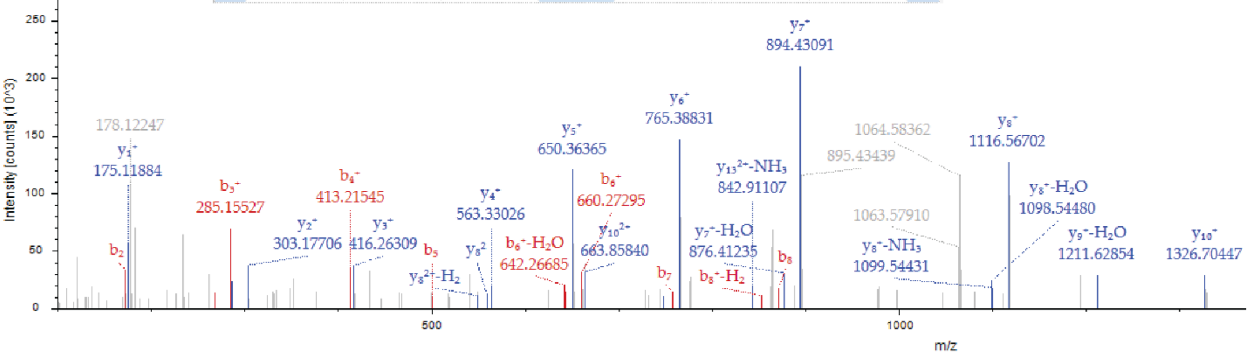

R

E A **K** P N G I F K

K-1182

#1	b⁺	b²⁺	Seq.	y⁺	y²⁺	#2
1	130.04987	65.52857	E			9
2	201.08698	101.04713	A	968.55637	484.78183	8
3	423.22381	212.11554	K-NASA_6...	897.51926	449.26327	7
4	520.27657	260.64192	P	675.38244	338.19486	6
5	634.31950	317.66339	N	578.32967	289.66847	5
6	691.34096	346.17412	G	464.28675	232.64701	4
7	804.42503	402.71615	I	407.26528	204.13628	3
8	951.49344	476.25036	F	294.18122	147.59425	2
9			K	147.11280	74.06004	1

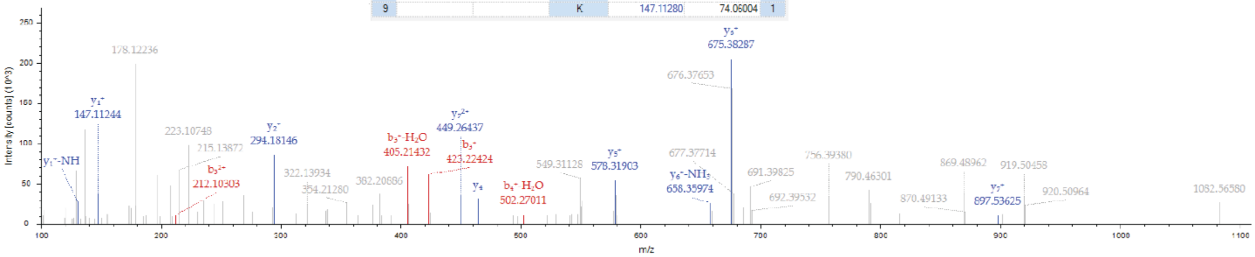

S

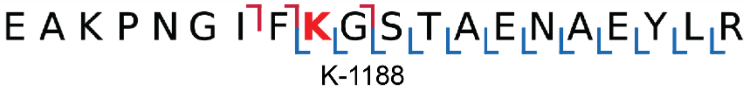

#1	b⁺	b²⁺	b³⁺	Seq.	y⁺	y²⁺	y³⁺	#2
1	130.04987	65.52857	44.02147	E				20
2	201.08698	101.04713	67.70051	A	2160.10839	1080.55783	720.70765	19
3	329.18195	165.09461	110.39883	K	2089.07127	1045.03928	697.02861	18
4	426.23471	213.62099	142.74975	P	1960.97631	980.99179	654.33029	17
5	540.27764	270.64246	180.76406	N	1863.92355	932.46541	621.97937	16
6	597.29910	299.15319	199.77122	G	1749.88062	875.44395	583.96506	15
7	710.38317	355.69522	237.46591	I	1692.85916	846.93322	564.95790	14
8	857.45158	429.22943	286.48871	F	1579.77509	790.39118	527.26322	13
9	1079.58840	540.29784	360.53432	K-NASA_6...	1432.70668	716.85698	478.24041	12
10	1136.60987	568.80857	379.54147	G	1210.56986	605.78857	404.19480	11
11	1223.64189	612.32459	408.55215	S	1153.54839	577.27783	385.18765	10
12	1324.68957	662.84842	442.23471	T	1066.51636	533.76182	356.17697	9
13	1395.72669	698.36698	465.91375	A	965.46869	483.23798	322.49441	8
14	1524.76928	762.88828	508.92794	E	894.43157	447.71942	298.81538	7
15	1638.81221	819.90974	546.94225	N	765.38898	383.19813	255.80118	6
16	1709.84932	855.42830	570.62129	A	651.34605	326.17666	217.78687	5
17	1838.89191	919.94959	613.63549	E	580.30894	290.65811	194.10783	4
18	2001.95524	1001.48126	667.98993	Y	451.26634	226.13681	151.09363	3
19	2115.03931	1058.02329	705.68462	L	288.20302	144.60515	96.73919	2
20				R	175.11895	88.06311	59.04450	1

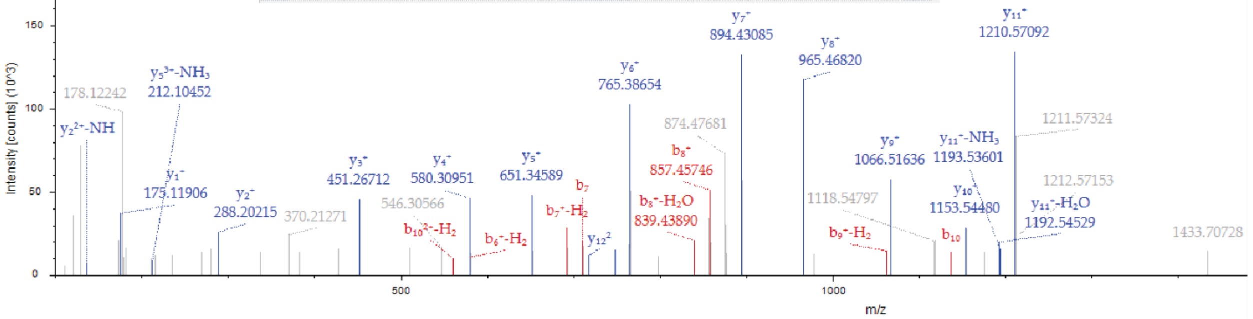

Figure S8. (A) EGFR-nitrile NASA conjugate **6** labels multiple lysines near the binding pocket. **(B)** Crystal structure of EGFR showing multiple lysines (blue sphere) labelled by **6**, EGFR (PDB: 6DUK), ligand shown as sticks. The C-terminal residues after position 1007 were omitted as they were unresolved in the co-crystal structure. **(C-S)** MS/MS spectrum of EGFR labeled peptides with **6**.

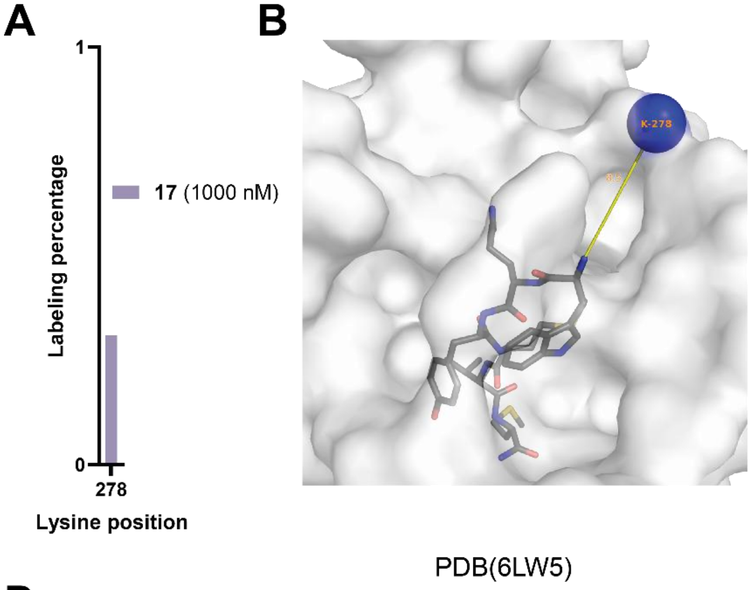

C

#1	b^+	b^{2+}	Seq.	y^+	y^{2+}	#2
1	223.14410	112.07569	K-NASA_6...			13
2	336.22816	168.61772	I	1284.74094	642.87411	12
3	449.31223	225.15975	I	1171.65688	586.33208	11
4	564.33917	282.67322	D	1058.57282	529.79005	10
5	677.42323	339.21526	I	943.54587	472.27657	9
6	790.50730	395.75729	L	830.46181	415.73454	8
7	889.57571	445.29149	V	717.37775	359.19251	7
8	1003.61864	502.31296	N	618.30933	309.65830	6
9	1100.67140	550.83934	P	504.26640	252.63684	5
10	1201.71908	601.36318	T	407.21364	204.11046	4
11	1288.75111	644.87919	S	306.16596	153.58662	3
12	1375.78314	688.39521	S	219.13393	110.07061	2
13			L	132.10191	66.55459	1

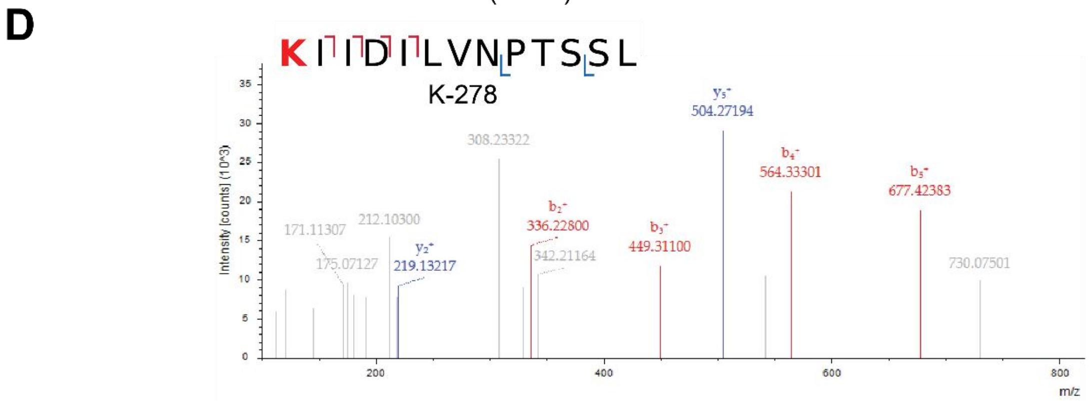

Figure S9. (A) FPR2 targeting SuFA probe **17** labels lysine-278 near the binding pocket. **(B)** Crystal structure of FPR2 showing lysine-178 (blue sphere) labeled by **17**, FPR2 (PDB:6LW5), distances in Å are shown as solid yellow lines. **(C-D)** MS/MS spectrum of FPR2 labeled peptide with **17**.

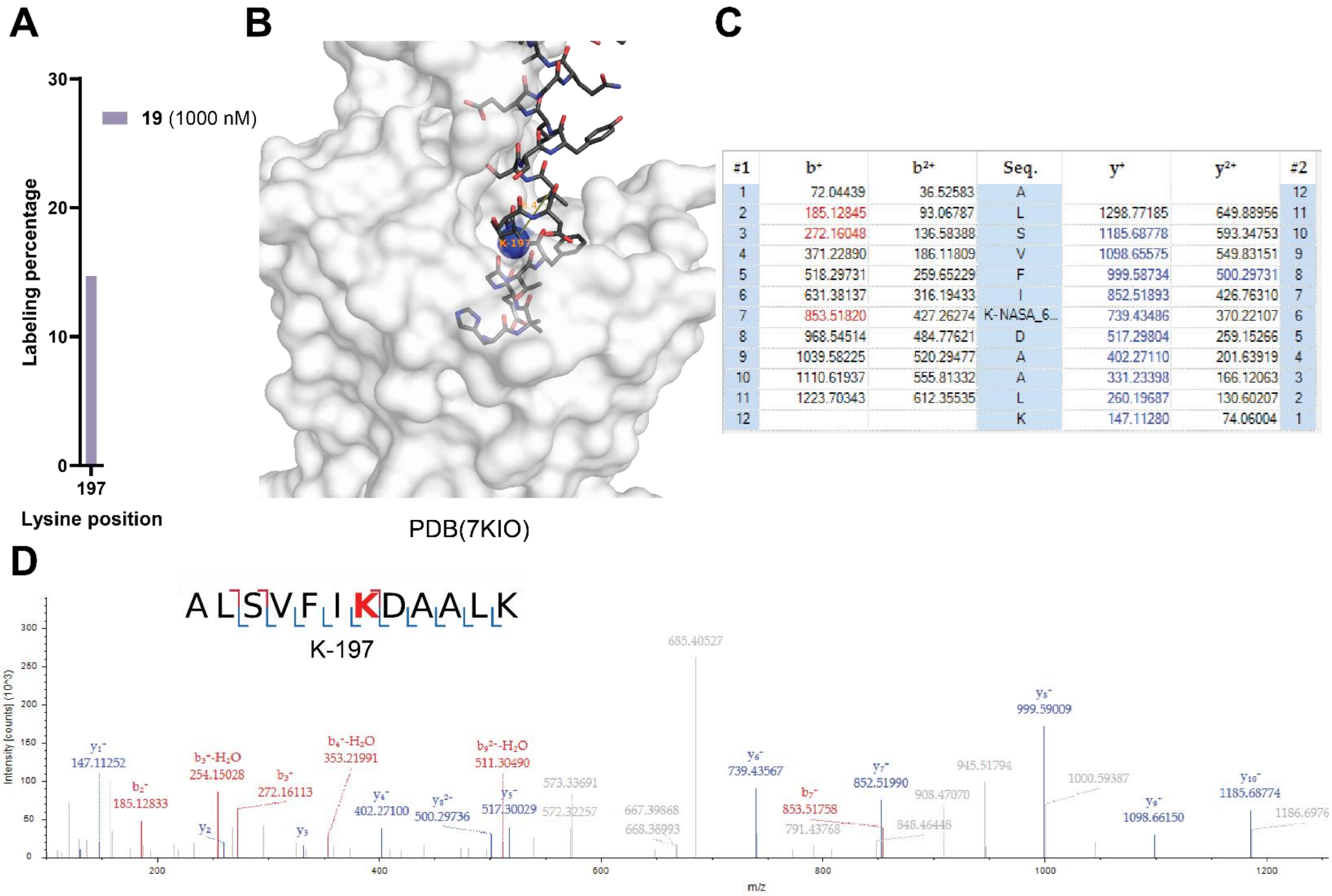

#1	b⁺	b²⁺	Seq.	y⁺	y²⁺	#2
1	72.04439	36.52583	A			12
2	185.12845	93.06787	L	1298.77185	649.88956	11
3	272.16048	136.58388	S	1185.68778	593.34753	10
4	371.22890	186.11809	V	1098.65575	549.83151	9
5	518.29731	259.65229	F	999.58734	500.29731	8
6	631.38137	316.19433	I	852.51893	426.76310	7
7	853.51820	427.26274	K-NASA_6...	739.43486	370.22107	6
8	968.54514	484.77621	D	517.29804	259.15266	5
9	1039.58225	520.29477	A	402.27110	201.63919	4
10	1110.61937	555.81332	A	331.23398	166.12063	3
11	1223.70343	612.35535	L	260.19687	130.60207	2
12			K	147.11280	74.06004	1

Figure S10. (A) GLP1R targeting SuFA probe **19** labels lysine-197 near the binding pocket. **(B)** Crystal structure of GLP1R showing lysine-197 (blue sphere) labeled by **19**, GLP1R (PDB:7KIO), distances in Å are shown as solid yellow lines. **(C-D)** MS/MS spectrum of GLP1R labeled peptide with **19**.

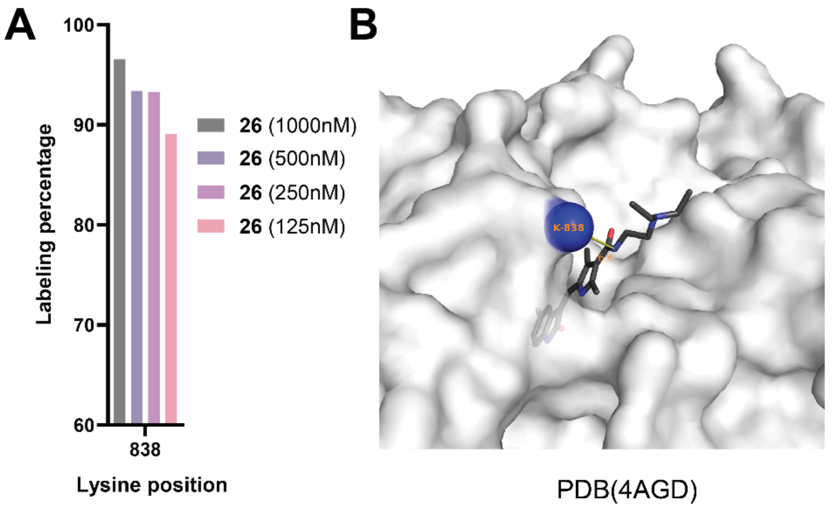

C

#1	b+	b2+	Seq.	y+	y2+	#2
1	114.09134	57.54931	L			7
2	171.11280	86.06004	G	721.43553	361.22140	6
3	393.24963	197.12845	K-NASA_6...	664.41407	332.71067	5
4	490.30239	245.65483	P	442.27724	221.64226	4
5	603.38645	302.19687	L	345.22448	173.11588	3
6	660.40792	330.70760	G	232.14042	116.57385	2
7			R	175.11895	88.06311	1

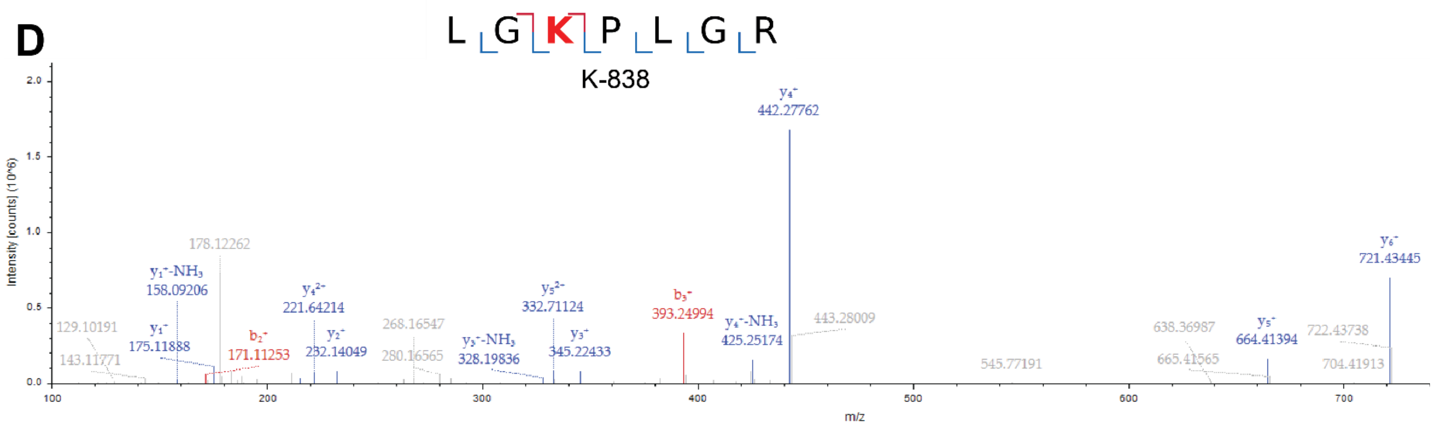

Figure S11. (A) VEGFR2 targeting SuFA probe **26** labels lysine-838 near the binding pocket. **(B)** Crystal structure of VEGFR2 showing lysine-838 (blue sphere) labeled by **26**, VEGFR2 (PDB:4AGD), distances in Å are shown as solid yellow lines. **(C-D)** MS/MS spectrum of VEGFR2 labeled peptide with **26**.

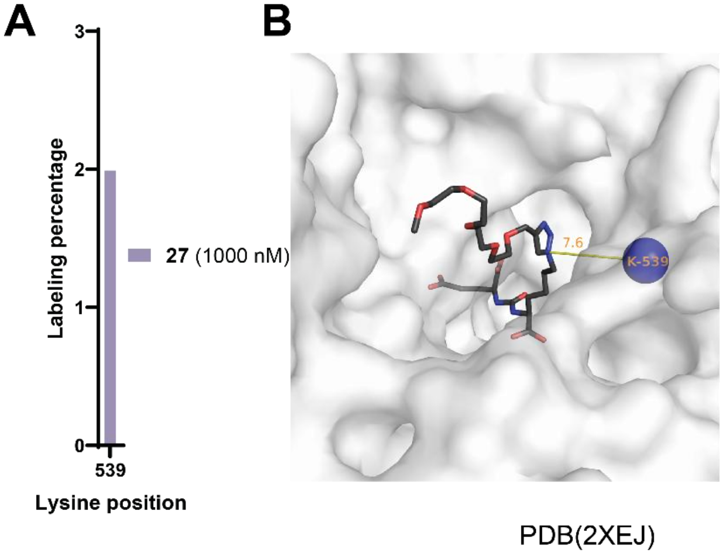

C

#1	b⁺	b²⁺	b³⁺	Seq.	y⁺	y²⁺	y³⁺	#2
1	102.05496	51.53112	34.68984	T				9
2	732.40492	366.70610	244.80649	K-SAS_PS...	1568.78662	784.89695	523.60039	8
3	846.44785	423.72756	282.82080	N	938.43666	469.72197	313.48374	7
4	1032.52716	516.76722	344.84724	W	824.39373	412.70050	275.46943	6
5	1161.56975	581.28851	387.86143	E	638.31442	319.66085	213.44299	5
6	1262.61743	631.81235	421.54399	T	509.27182	255.13955	170.42879	4
7	1376.66036	688.83382	459.55830	N	408.22415	204.61571	136.74623	3
8	1504.75532	752.88130	502.25662	K	294.18122	147.59425	98.73192	2
9				F	166.08626	83.54677	56.03360	1

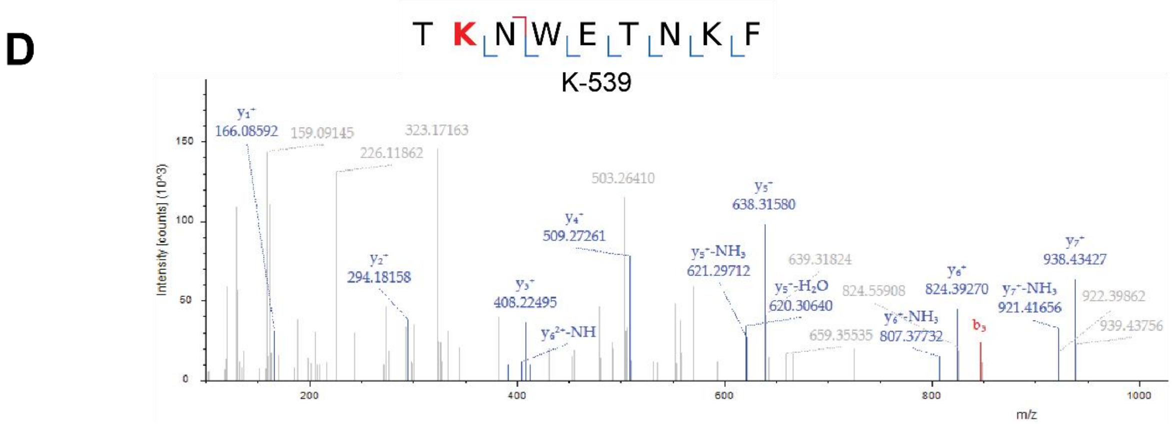

Figure S12. (A) PSMA targeting GRC **27** labels lysine-539 near the binding pocket. **(B)** Crystal structure of PSMA showing lysine-539 (blue sphere) labeled by **27**, PSMA (PDB:2XEJ), distances in Å are shown as solid yellow lines. **(C-D)** MS/MS spectrum of PSMA labeled peptide with **27**.

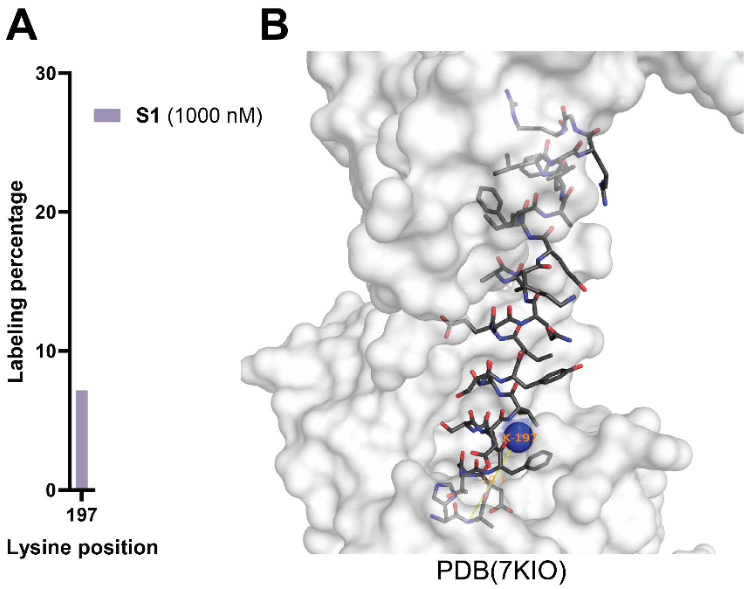

C

#1	b⁺	b²⁺	Seq.	y⁺	y²⁺	#2
1	72.04439	36.52583	A			12
2	185.12845	93.06787	L	1298.77185	649.88956	11
3	272.16048	136.58388	S	1185.68778	593.34753	10
4	371.22890	186.11809	V	1098.65575	549.83151	9
5	518.29731	259.65229	F	999.58734	500.29731	8
6	631.38137	316.19433	I	852.51893	426.76310	7
7	853.51820	427.26274	K-NASA_6...	739.43486	370.22107	6
8	968.54514	484.77621	D	517.29804	259.15266	5
9	1039.58225	520.29477	A	402.27110	201.63919	4
10	1110.61937	555.81332	A	331.23398	166.12063	3
11	1223.70343	612.35535	L	260.19687	130.60207	2
12			K	147.11280	74.06004	1

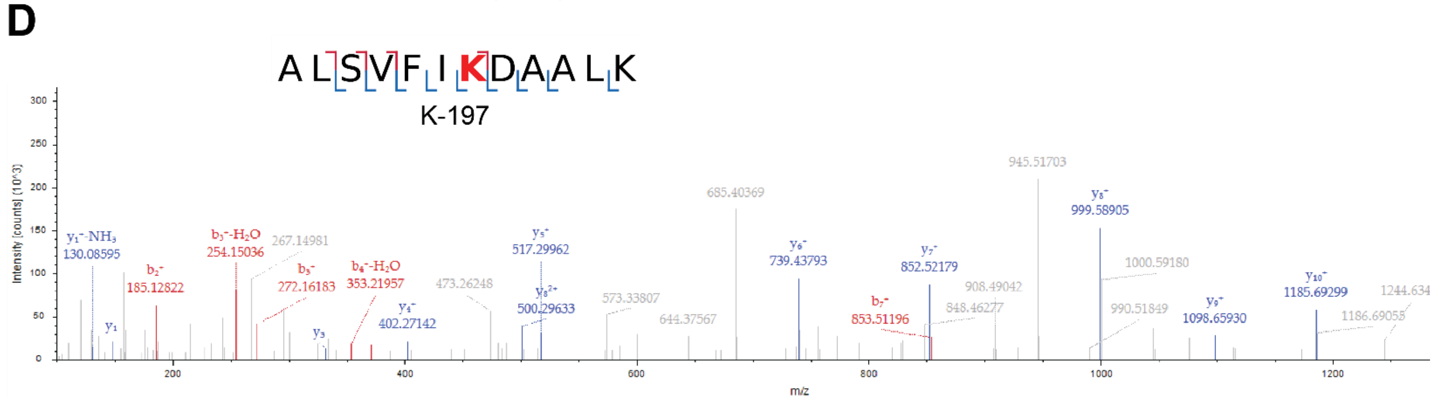

Figure S13. (A) GLP1R targeting SuFA probe **S1** labels lysine-197 near the binding pocket. **(B)** Crystal structure of GLP1R showing lysine-197 (blue sphere) labeled by **S1**, GLP1R (PDB:7KIO), distances in Å are shown as solid yellow lines. **(C-D)** MS/MS spectrum of GLP1R labeled peptide with **S1**.

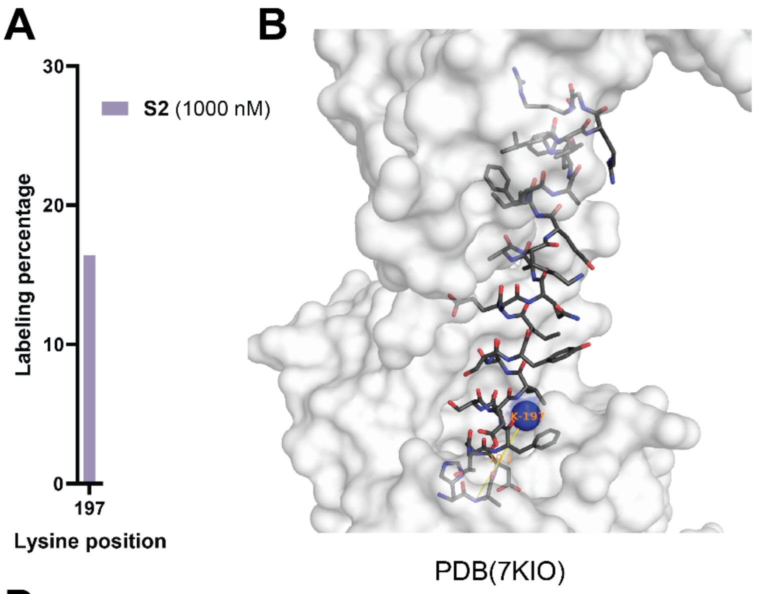

C

#1	b^+	b^{2+}	Seq.	y^+	y^{2+}	#2
1	72.04439	36.52583	A			12
2	185.12845	93.06787	L	1298.77185	649.88956	11
3	272.16048	136.58388	S	1185.68778	593.34753	10
4	371.22890	186.11809	V	1098.65575	549.83151	9
5	518.29731	259.65229	F	999.58734	500.29731	8
6	631.38137	316.19433	I	852.51893	426.76310	7
7	853.51820	427.26274	K-NASA_6...	739.43486	370.22107	6
8	968.54514	484.77621	D	517.29804	259.15266	5
9	1039.58225	520.29477	A	402.27110	201.63919	4
10	1110.61937	555.81332	A	331.23398	166.12063	3
11	1223.70343	612.35535	L	260.19687	130.60207	2
12			K	147.11280	74.06004	1

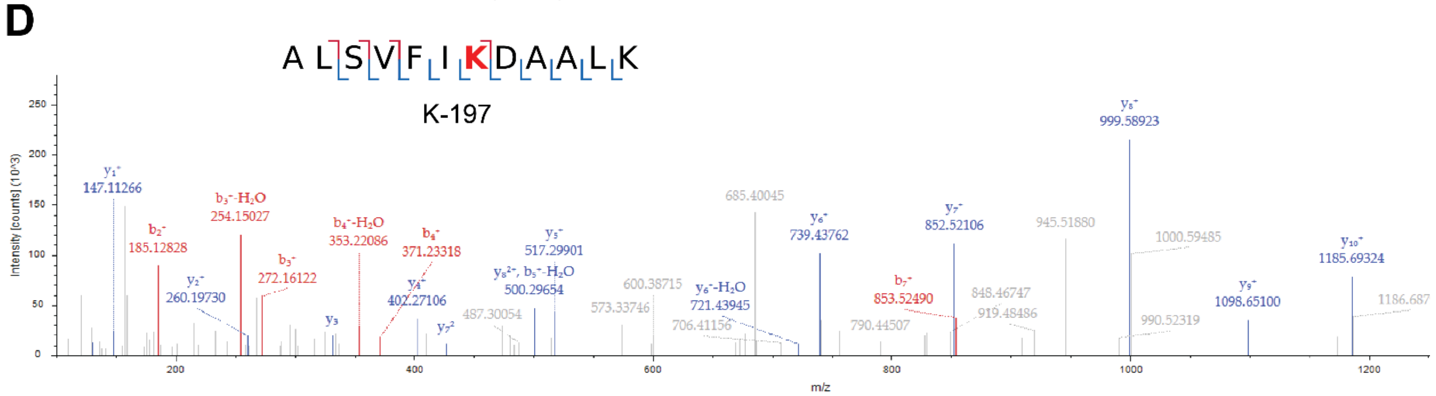

Figure S14. (A) GLP1R targeting SuFA probe **S2** labels lysine-197 near the binding pocket. **(B)** Crystal structure of GLP1R showing lysine-197 (blue sphere) labeled by **S2**, GLP1R (PDB:7KIO), distances in Å are shown as solid yellow lines. **(C-D)** MS/MS spectrum of GLP1R labeled peptide with **S2**.

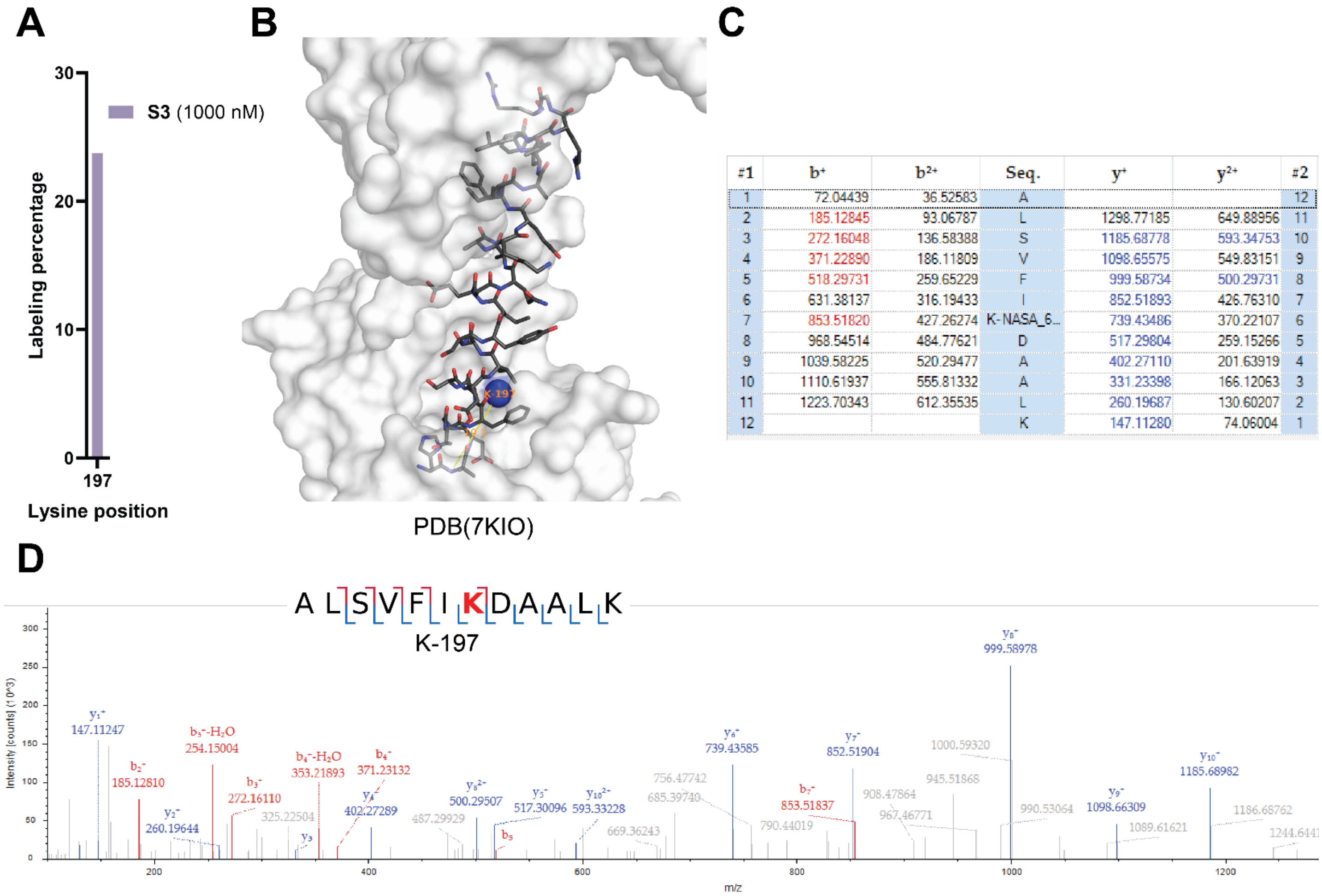

#1	b⁺	b²⁺	Seq.	y⁺	y²⁺	#2
1	72.04439	36.52583	A			12
2	185.12845	93.06787	L	1298.77185	649.88956	11
3	272.16048	136.58388	S	1185.68778	593.34753	10
4	371.22890	186.11809	V	1098.65575	549.83151	9
5	518.29731	259.65229	F	999.58734	500.29731	8
6	631.38137	316.19433	I	852.51893	426.76310	7
7	853.51820	427.26274	K-NASA_6...	739.43486	370.22107	6
8	968.54514	484.77621	D	517.29804	259.15266	5
9	1039.58225	520.29477	A	402.27110	201.63919	4
10	1110.61937	555.81332	A	331.23398	166.12063	3
11	1223.70343	612.35535	L	260.19687	130.60207	2
12			K	147.11280	74.06004	1

Figure S15. (A) GLP1R targeting SuFA probe **S3** labels lysine-197 near the binding pocket. **(B)** Crystal structure of GLP1R showing lysine-197 (blue sphere) labeled by **S3**, GLP1R (PDB:7KIO), distances in Å are shown as solid yellow lines. **(C-D)** MS/MS spectrum of GLP1R labeled peptide with **S3**.

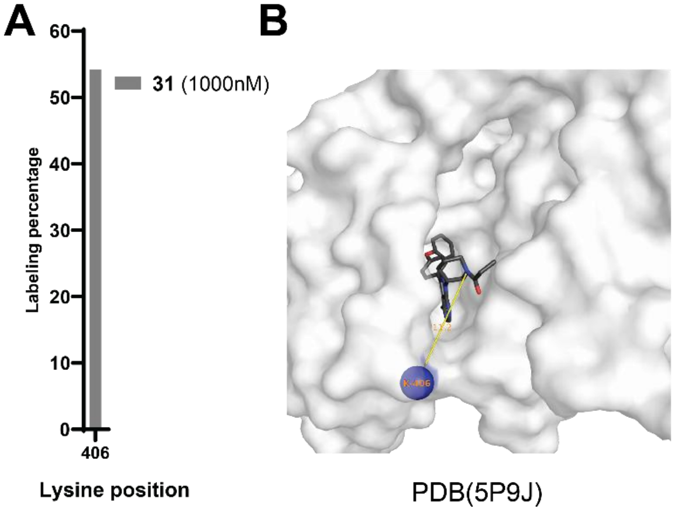

C

1	116.03422	58.52075	39.34959	D				[illegible]
2	229.11828	115.06278	77.04428	L	2080.16083	1040.58405	694.05846	[illegible]
3	330.16596	165.58662	110.72684	T	1967.07677	984.04202	656.36377	[illegible]
4	477.23438	239.12083	159.74964	F	1866.02909	933.51818	622.68121	[illegible]
5	590.31844	295.66286	197.44433	L	1718.96068	859.98398	573.65841	[illegible]
6	1061.57970	531.29349	354.53142	K-SAS_BT...	1605.87661	803.44194	535.96372	[illegible]
7	1190.62230	595.81479	397.54562	E	1134.61535	567.81131	378.87663	[illegible]
8	1303.70636	652.35682	435.24030	L	1005.57276	503.29002	335.86244	[illegible]
9	1360.72782	680.86755	454.24746	G	892.48869	446.74798	298.16775	9
10	1461.77550	731.39139	487.93002	T	835.46723	418.23725	279.16059	8
11	1518.79697	759.90212	506.93717	G	734.41955	367.71341	245.47803	7
12	1646.85554	823.93141	549.62337	Q	677.39809	339.20268	226.47088	6
13	1793.92396	897.46562	598.64617	F	549.33951	275.17339	183.78469	5
14	1850.94542	925.97635	617.65332	G	402.27110	201.63919	134.76188	4
15	1950.01383	975.51056	650.67613	V	345.24963	173.12845	115.75473	3
16	2049.08225	1025.04476	683.69893	V	246.18122	123.59425	82.73192	2
17				K	147.11280	74.06004	49.70912	1

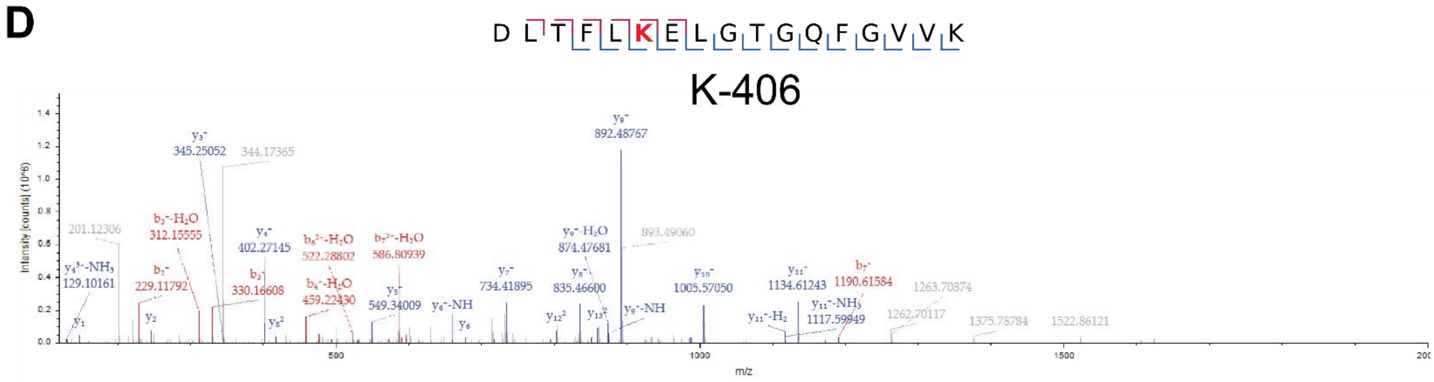

Fig S16. (A) BTK targeting GRC **31** labels lysine-406 near the binding pocket. **(B)** Crystal structure of BTK showing lysine-406 (blue sphere) labeled by **31**, BTK (PDB:5P9J), distances in Å are shown as dashed yellow lines. **(C-D)** MS/MS spectrum of BTK labeled peptide with **31**.

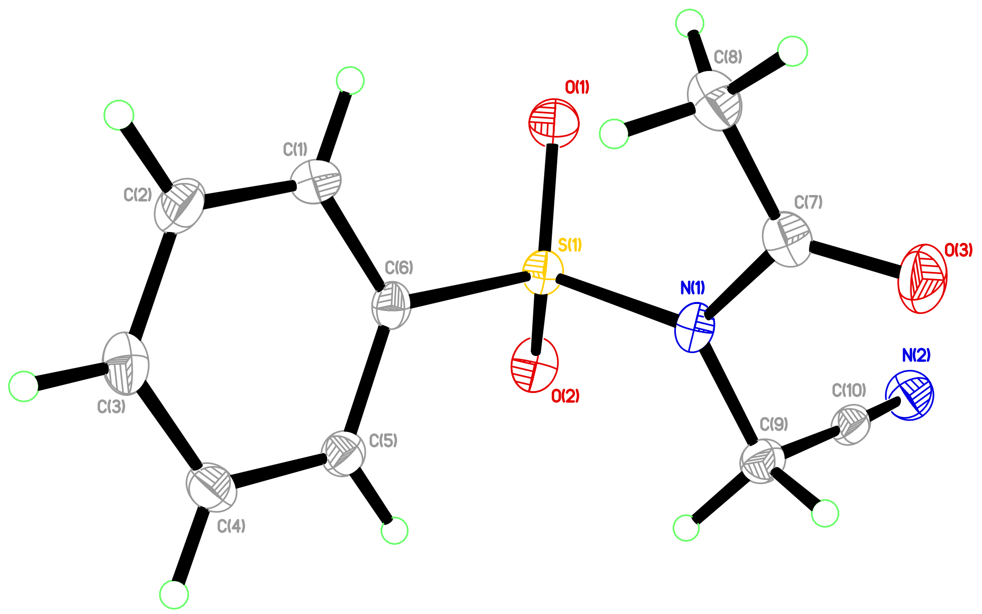

Figure S17*.* Perspective views showing 50% probability of displacement. X-ray structure of compound **9**.

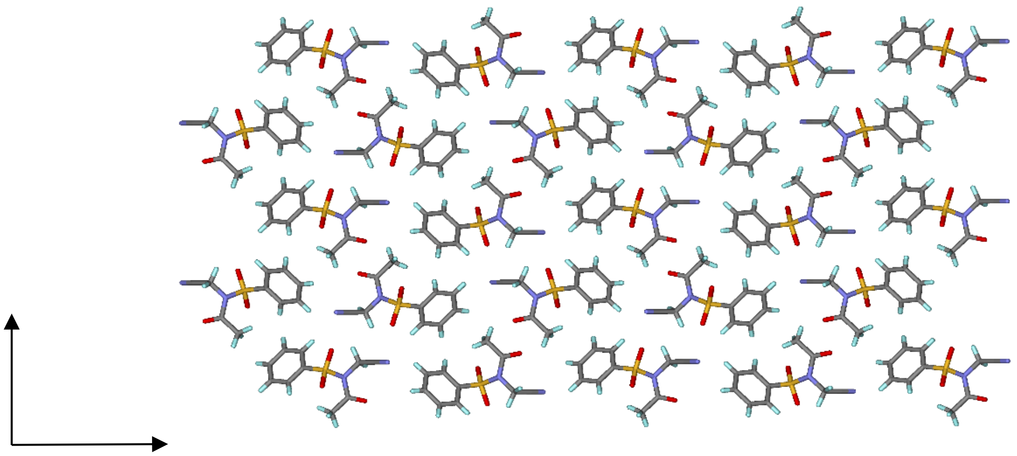

Figure S18. Three-dimensional supramolecular architecture viewed along the *a*-axis direction. X-ray structure of compound **9**.

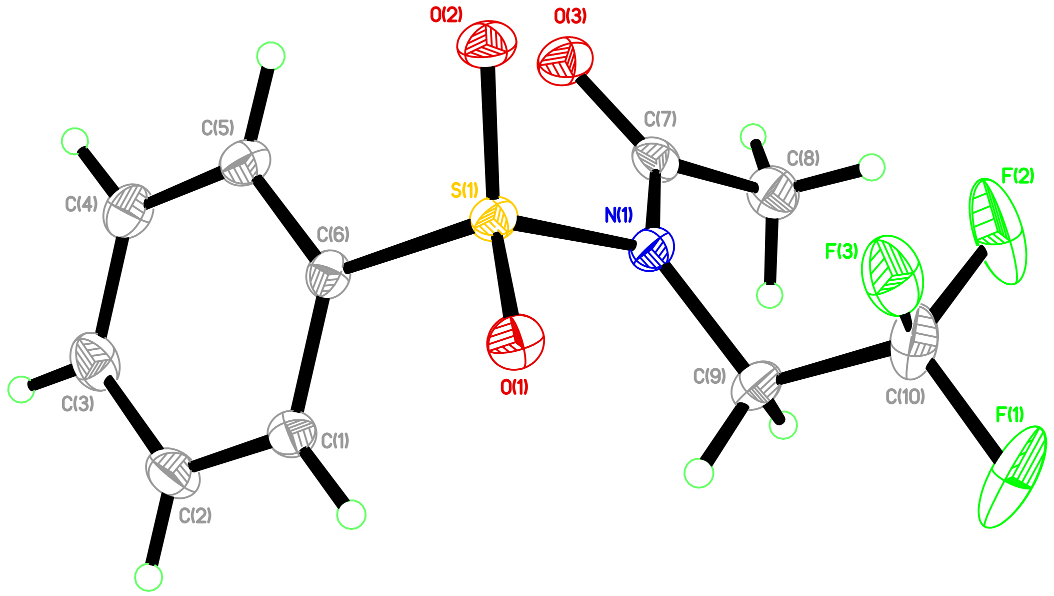

Figure S19. Perspective views showing 50% probability of displacement. X-ray structure of **11**.

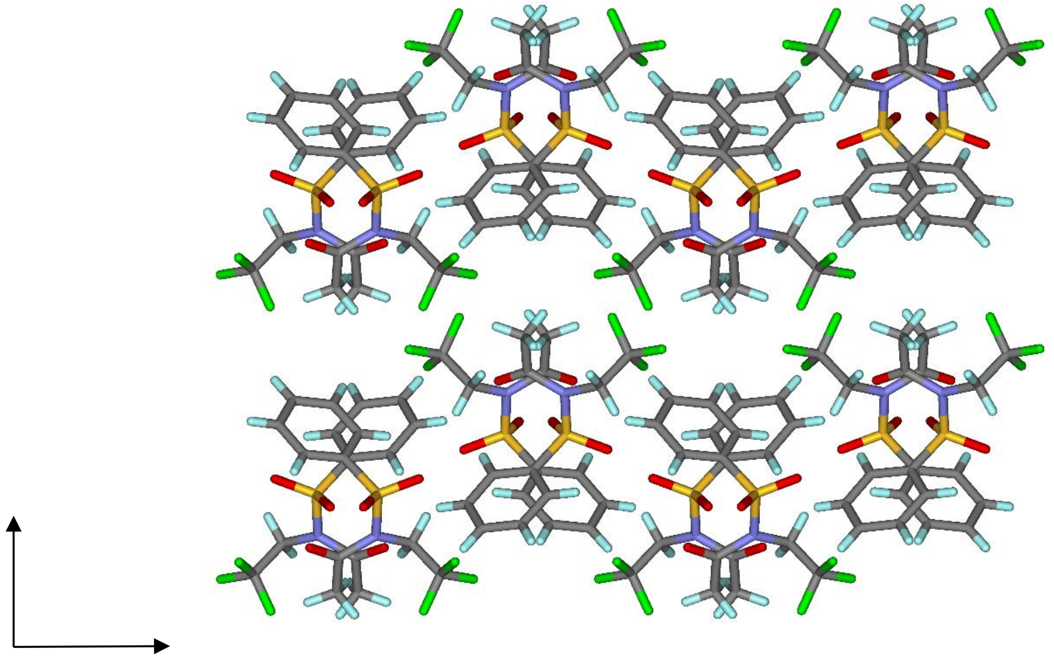

Figure S20. Three-dimensional supramolecular architecture viewed along the *c*-axis direction. X-ray structure of **11**.

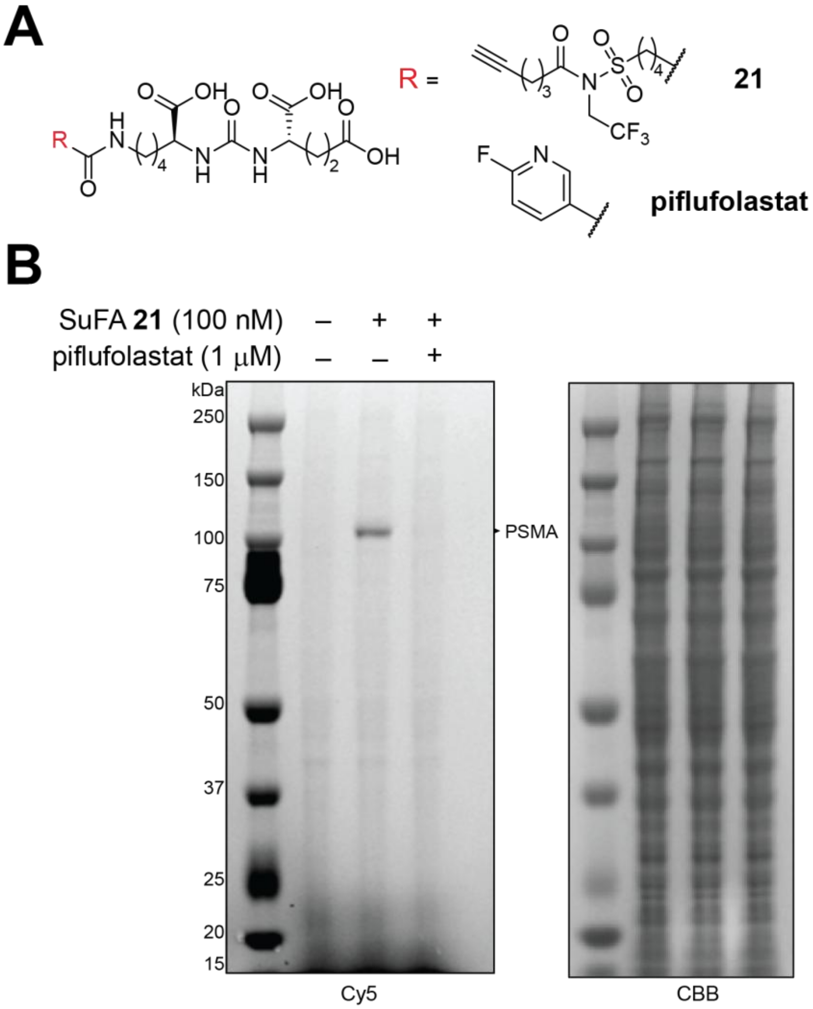

Figure S21. Labeling experiment of PSMA SuFA probe in LNCaP cells. (**A**) Structure of the SuFA probe **21** and the competitor piflufolastat. (**B**) Labeling in LnCAP cells by PSMA SuFA probe **21** (100 nM) with and without co-treatment of piflufolastat (1 μM) as competitor. Full-size blots can be found at Figure S25.

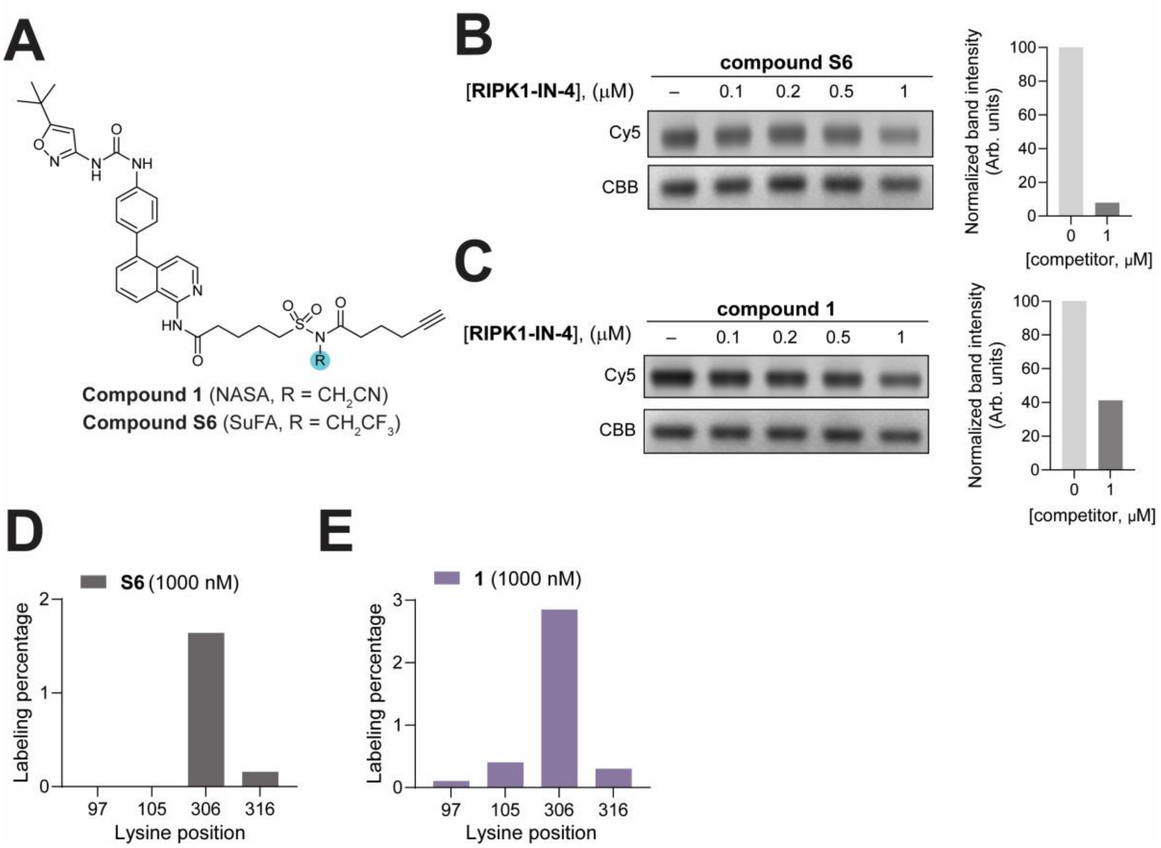

Figure S22. Comparison between NASA and SuFA labeling profiles. (**A**) Structures of RIPK1 SuFA and NASA probes. (**B-C**) Labeling of RIPK1 protein (0.8 µM) by (**B**) 100 nM SuFA probe **S6** and (**C**) 100 nM NASA probe **1** was competed away with varying concentrations of RIPK1-IN-4 as competitor (CAS: 1481641-08-0). (**D-E**) Labeling sites on RIPK1 using (**D**) 1 µM SuFA probe **S6** and (**E**) 1 µM NASA probe **1**. Full size blots of Figure S22B-S22C can be found at Figure S26.

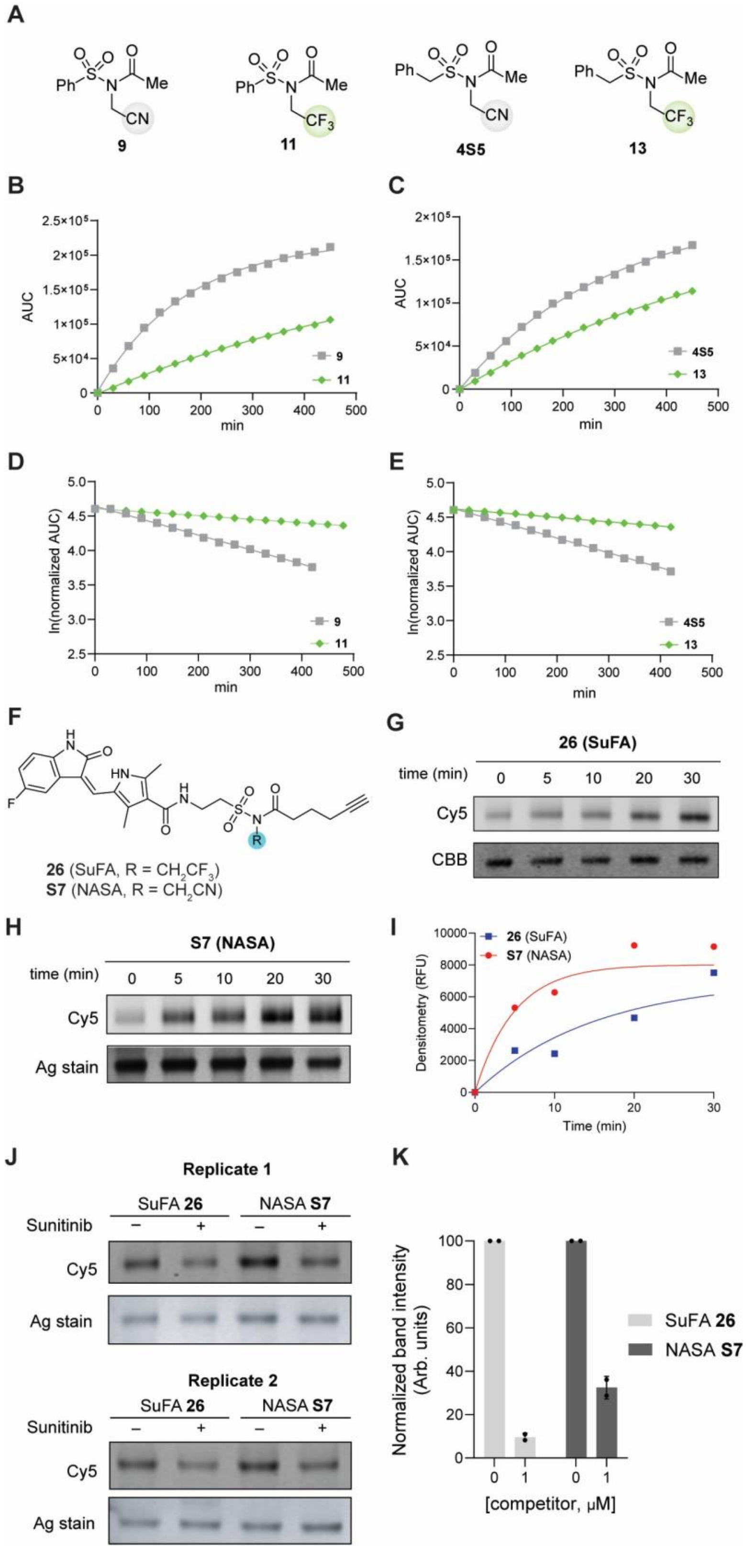

Figure S23. Comparison of reaction kinetics between matched pairs of SuFA and NASA. (**A**) Structures of NASA and SuFA pairs based on arylsulfonamides (**9** and **11**) and benzylsulfonamides (**4S5** and **13**). (**B**) Lysine reactivity of compounds **9** and **11** in MeCN with 1 equivalent *N*-Boc-lysine coumarin. (**C**) Lysine reactivity of compounds **4S5** and **13** in MeCN with 1 equivalent *N*-Boc-lysine coumarin. (**D**) Hydrolysis kinetics measured in PBS pH 7.4 for compounds **9** and **11**. (**E**) Hydrolysis kinetics measured in PBS pH = 7.4 for compounds **4S5** and **13**. (**F**) Structures of VEGFR2 SuFA and NASA probes. (**G-H**) Time-course labeling of VEGFR2 protein (0.5 μM) by 10 μM SuFA

probe **26** (**G**) and 10 µM NASA probe **S7** (**H**). (**I**) Quantification of band intensity for the blots in **G** and **H.** k_{obs} was obtained via first-order association fit. (**J-K**) Labeling of VEGFR2 protein (0.8 µM) by 100 nM SuFA probe **26** and 100 nM NASA probe **S7** was competed away using sunitinib (1 µM, 10x) as pre-incubated competitor. Full size blots of Figure S23G-S22H and Figure S23J can be found at Figure S27 and Figure S28, respectively.

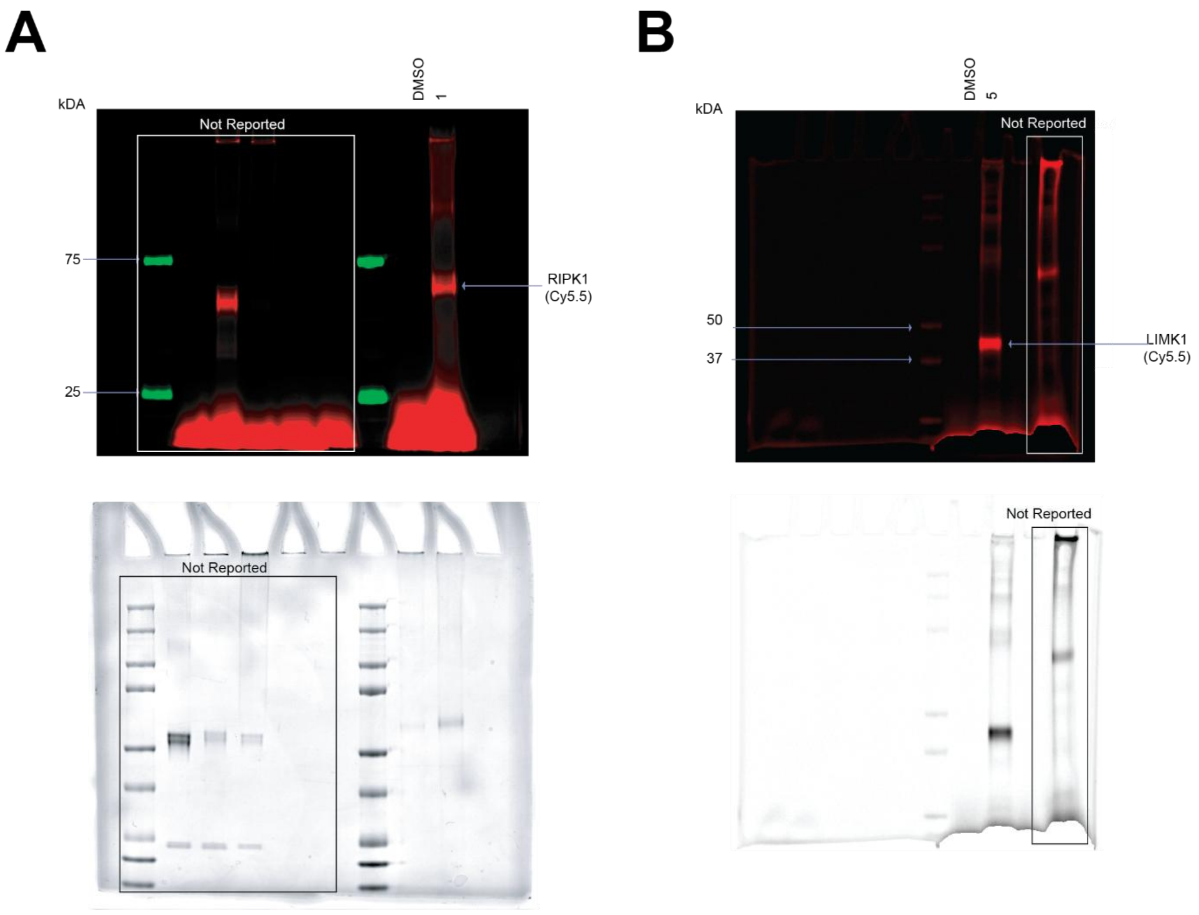

Figure S24. Full-size blots for Figure S2C. Representative blots of in-gel fluorescence studies using NASA probes demonstrating labeling of purified proteins.

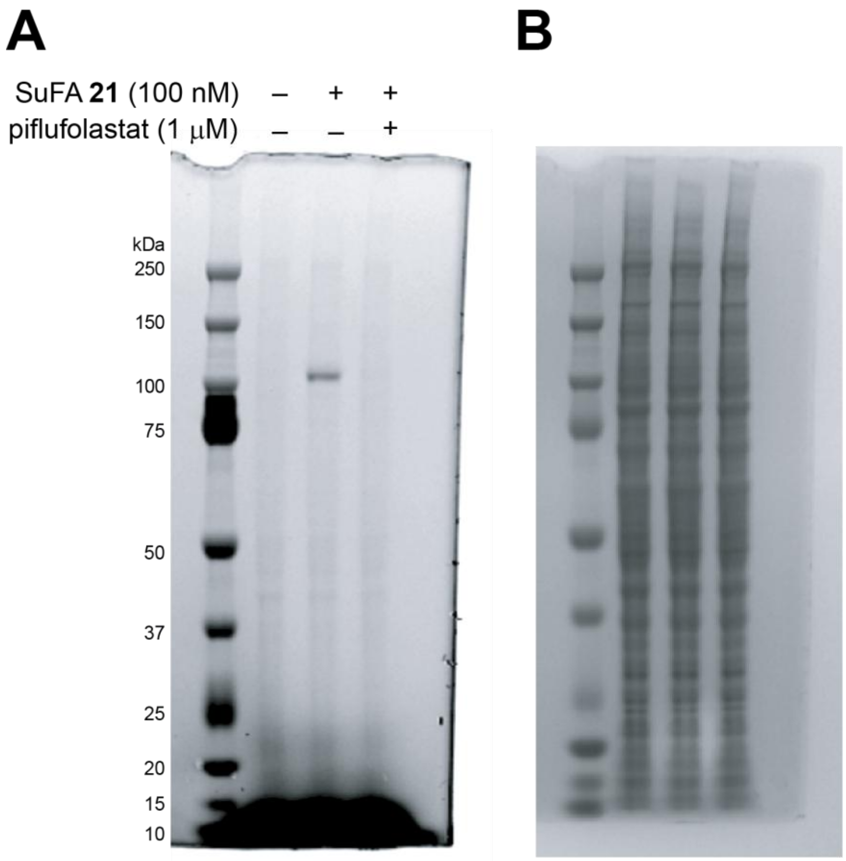

Figure S25. Full-size blots for Figure S21. Labeling experiment of PSMA SuFA probe in LNCaP cells. (**A**) In-gel fluorescence. (**B**) Coomassie stain.

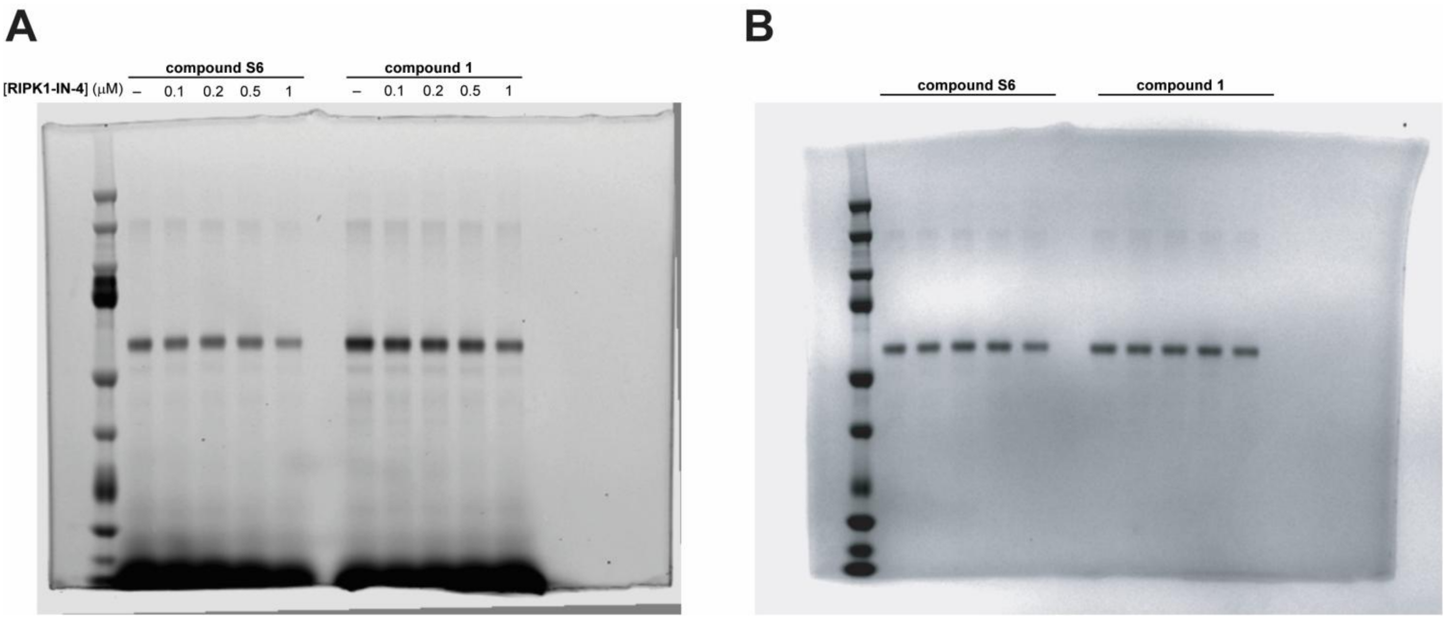

Figure S26. Full size blots of Figure S22B-S22C. Labeling of RIPK1 protein (0.8 μM) by 100 nM SuFA probe **S6** and 100 nM NASA probe **1** was competed away with varying concentrations of RIPK1-IN-4 as competitor (CAS: 1481641-08-0). (**A**) In-gel fluorescence. (**B**) Coomassie stain.

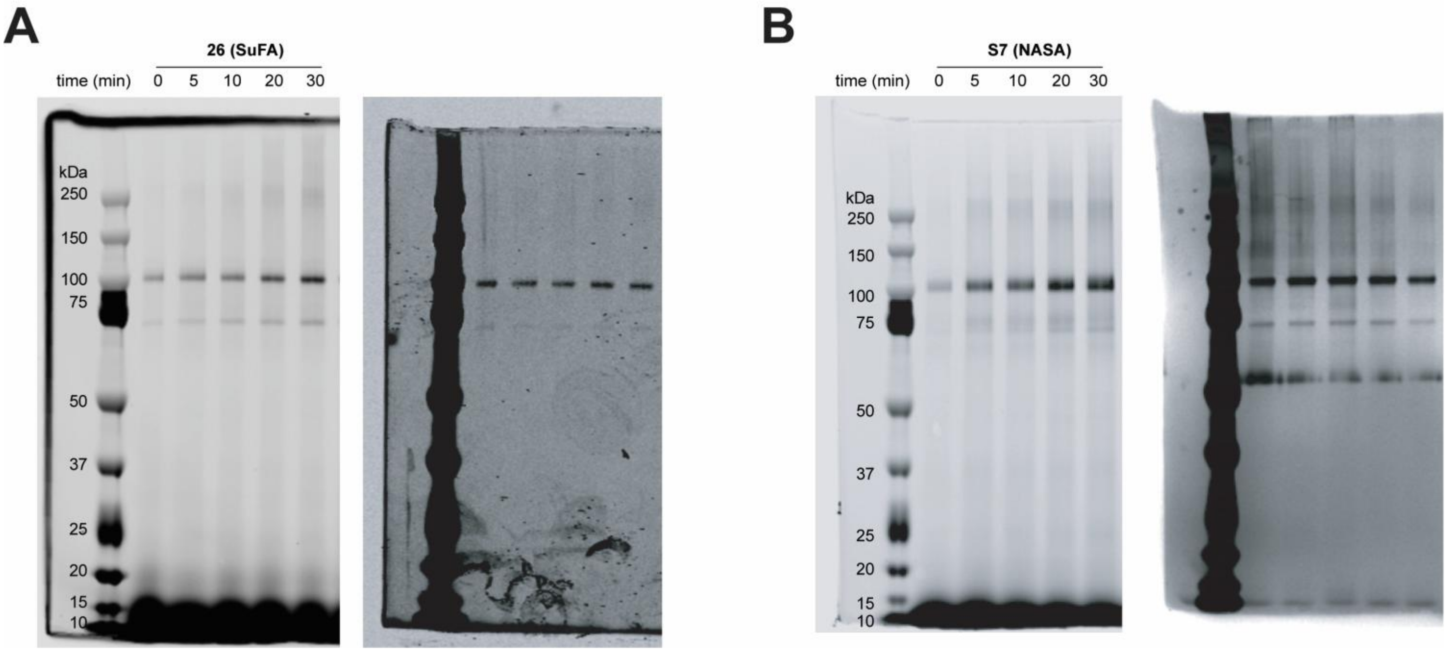

Figure S27. Full size blots of Figure S23G–S23H. Time-course labeling of VEGFR2 protein (0.5 μM) by 10 μM SuFA probe **26** and 10 μM NASA probe **S7**. (**A**) Full size blots of Figure S23G. In-gel fluorescence on the left and Coomassie stain on the right. (**B**) Full size blots of Figure S23H. In-gel fluorescence on the left and silver stain on the right.

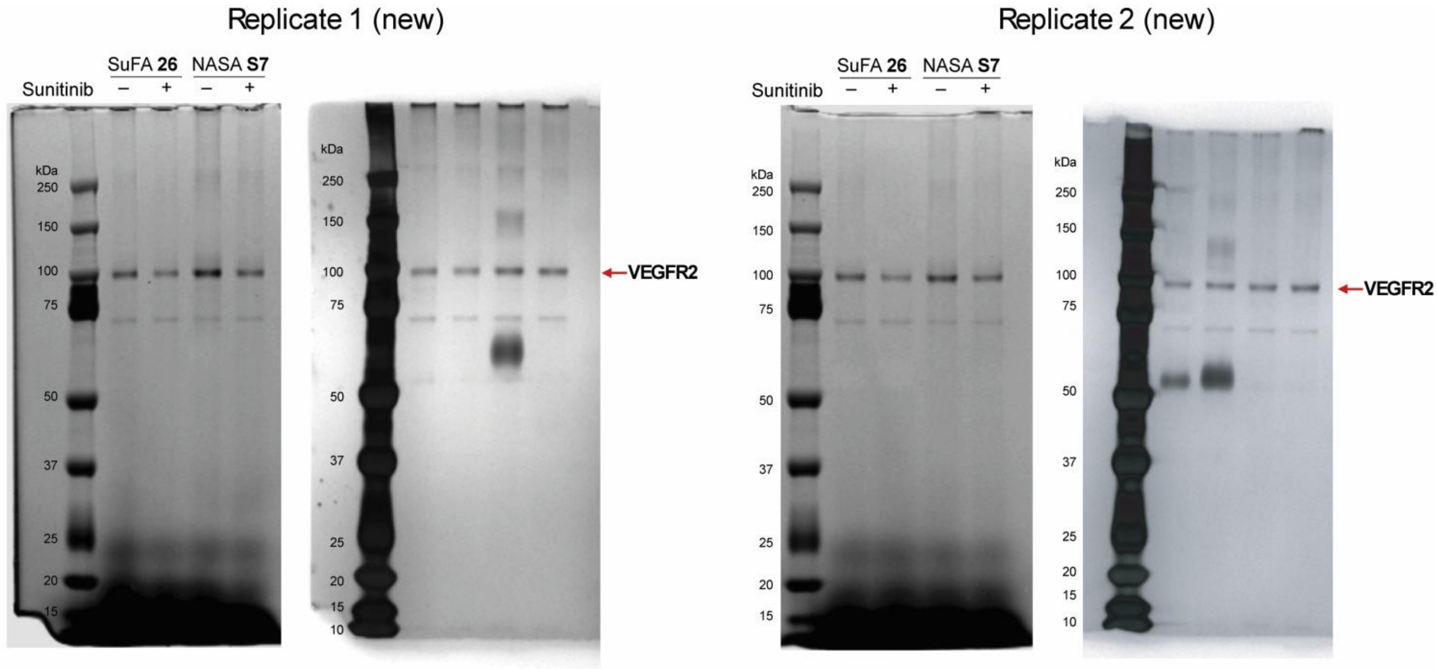

Figure S28. Full size blots of Figure S23J. Labeling of VEGFR2 protein (0.8 μM) by 100 nM SuFA probe **26** and 100 nM NASA probe **S7** was competed away using sunitinib (1 μM, 10x) as pre-incubated competitor. For each replicate, the in-gel fluorescence was shown on the left and the silver stain on the right.

3 Synthesis and Characterization

3.1 Synthesis of the NASA probes

3.1.1 Compound **1** (RIPK1 NASA probe)

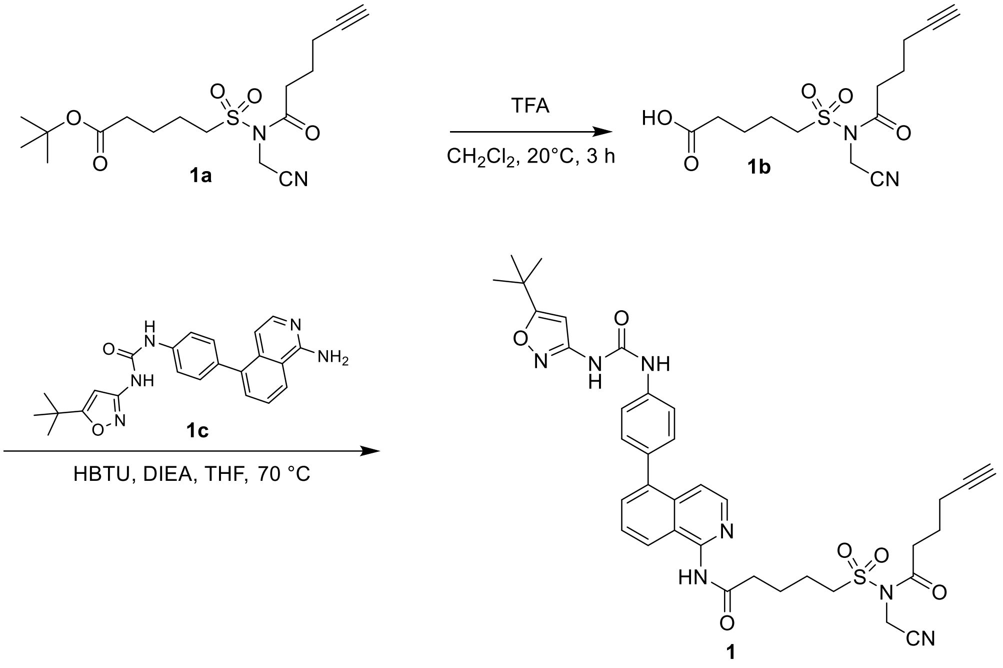

5-(*N*-(cyanomethyl)-*N*-(hex-5-ynoyl)sulfamoyl)pentanoic acid (1b)

To a solution of *tert*-butyl 5-(*N*-(cyanomethyl)-*N*-(hex-5-ynoyl)sulfamoyl)pentanoate (360 mg, 0.97 mmol) in DCM (8.00 mL) was added TFA (1.54 g, 13.50 mmol) at 20 °C for 3 hrs. TLC (PE/EA = 1/1) indicated **1a** was consumed completely and one new spot formed. The reaction was clean according to TLC. The reaction mixture was filtered and concentrated under reduced pressure to give a residue. The residue was purified by column chromatography (SiO_2, PE/EA =3/1 to 1/1) to give 5-(*N*-(cyanomethyl)-*N*-(hex-5-ynoyl)sulfamoyl)pentanoic acid (260 mg, 0.58 mmol, 60% yield, 70% purity) as yellow oil. **^{1}H NMR** (400 MHz, $CDCl_3$) δ 4.67 (s, 2H), 3.57 - 3.40 (m, 2H), 2.93 (t, J = 7.2 Hz, 2H), 2.46 (t, J = 7.2 Hz, 2H), 2.33 (dt, J = 2.8, 6.8 Hz, 2H), 2.02 - 1.92 (m, 4H), 1.91 - 1.78 (m, 3H). **MS** (ESI+): *m/z* 336.9 $[M+Na]^+$.

***N*-((5-((5-(4-(3-(5-(*tert*-butyl)isoxazol-3-yl)ureido)phenyl)isoquinolin-1-yl)amino)-5-oxopentyl)sulfonyl)-*N*-(cyanomethyl)hex-5-ynamide (1)**

To a solution of 1-(4-(1-aminoisoquinolin-5-yl)phenyl)-3-(5-(*tert*-butyl)isoxazol-3-yl)urea (13.0 mg, 0.032 mmol, 1.0 equiv.), HATU (14.8 mg, 0.039 mmol, 1.2 equiv.) and DIPEA (6.3 mg, 0.049 mmol, 1.5 equiv.) in THF (0.5 mL) at 0 °C for 0.5 hr. The mixture was added 5-(*N*-(cyanomethyl)-*N*-(hex-5-ynoyl)sulfamoyl)pentanoic acid (14.5 mg, 0.032 mmol, 70% purity, 1.0 equiv.) at 60 °C for 3 hrs. LC-MS showed no starting material remained. Several new peaks were shown on LC-MS and the desired compound was detected. The reaction mixture was filtered over celite, and the filtrate was purified by prep-HPLC (column: Welch Xtimate C18 150*25mm*5um; mobile phase: [water(TFA)-ACN];B%: 28%-58%,10min) to give *N*-((5-((5-(4-(3-(5-(tert-butyl)isoxazol-3-

yl)ureido)phenyl)isoquinolin-1-yl)amino)-5-oxopentyl)sulfonyl)-*N*-(cyanomethyl)hex-5-ynamide (6.7 mg, 0.009 mmol, 29% yield, 96% purity) as off-white solid. **^{1}H NMR** (400 MHz, CD_3OD) *δ* 8.73 (d, *J* = 8.4 Hz, 1H), 8.09 (d, *J* = 7.2 Hz, 2H), 8.05 - 8.00 (m, 1H), 7.89 - 7.85 (m, 1H), 7.75 - 7.67 (m, 2H), 7.51 - 7.43 (m, 2H), 6.41 (s, 1H), 4.76 (s, 2H), 3.69 (t, *J* = 7.2 Hz, 2H), 2.95 (q, *J* = 6.4 Hz, 4H), 2.34 - 2.25 (m, 3H), 2.03 (dd, *J* = 2.8, 4.4 Hz, 4H), 1.93 - 1.85 (m, 2H), 1.37 (s, 9H). **MS** (ESI+): *m/z* 698.4 $[M+H]^+$.

3.1.2 Compound **2** (MEK2 NASA probe)

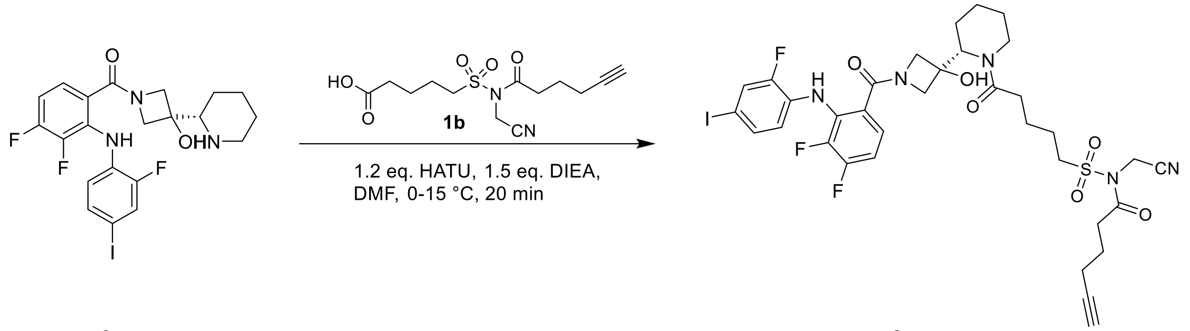

2a 2

***N*-(cyanomethyl)-*N*-[5-[(2*S*)-2-[1-[3,4-difluoro-2-(2-fluoro-4-iodo-anilino)benzoyl]-3-hydroxy-azetidin-3-yl]-1-piperidyl]-5-oxo-pentyl]sulfonyl-hex-5-ynamide (2)**

To a solution of [3,4-difluoro-2-(2-fluoro-4-iodo-anilino)phenyl]-[3-hydroxy-3- [(2S)-2-piperidyl]azetidin-1-yl]methanone (30 mg, 0.056 mmol, 1.0 equiv.) and 5-[cyanomethyl (hex-5-ynoyl)sulfamoyl]pentanoic acid (59.2 mg, 0.11 mmol, 60% purity, 2.0 equiv.) in DMF (0.4 mL) was added DIPEA (10.9 mg, 0.085 mmol, 14.7 µL, 1.5 equiv.) and HATU (25.8 mg, 0.068 mmol, 1.2 equiv.) at 0 °C. The mixture was stirred at 25 °C for 6 h. LCMS showed the desired compound was detected. The reaction solution is filtered, and the filtrate is collected. The residue was purified by prep-HPLC (column: Welch Xtimate C18 150*25mm*5um;mobile phase: [water(TFA)-ACN];B%: 52%-72%,10min) to give *N*-(cyanomethyl)-*N*-[5-[(2S)-2-[1-[3,4-difluoro-2-(2-fluoro-4-iodo-anilino)benzoyl]-3-hydroxy-azetidin-3-yl]-1-piperidyl]-5-oxo-pentyl]sulfonyl-hex-5-ynamide (15.6 mg, 0.015 mmol, 27% yield, 82% purity) as a white solid. **^{1}H NMR** (400 MHz, $CDCl_3$) δ 6.99 - 6.91 (m, 1H), 6.90 - 6.82 (m, 1H), 3.50 - 3.43 (m, 2H), 3.30 - 3.22 (m, 2H), 2.81 (t, J = 2.4 Hz, 1H), 2.38 (t, J = 7.2 Hz, 2H), 2.18 (dt, J = 2.4, 7.2 Hz, 2H), 1.68 (quin, J = 7.2 Hz, 2H), 1.37 (s, 9H). **MS** (ESI+): *m/z* 828.3 $[M+H]^+$.

3.1.3 Compound **3** (IRE1α NASA probe)

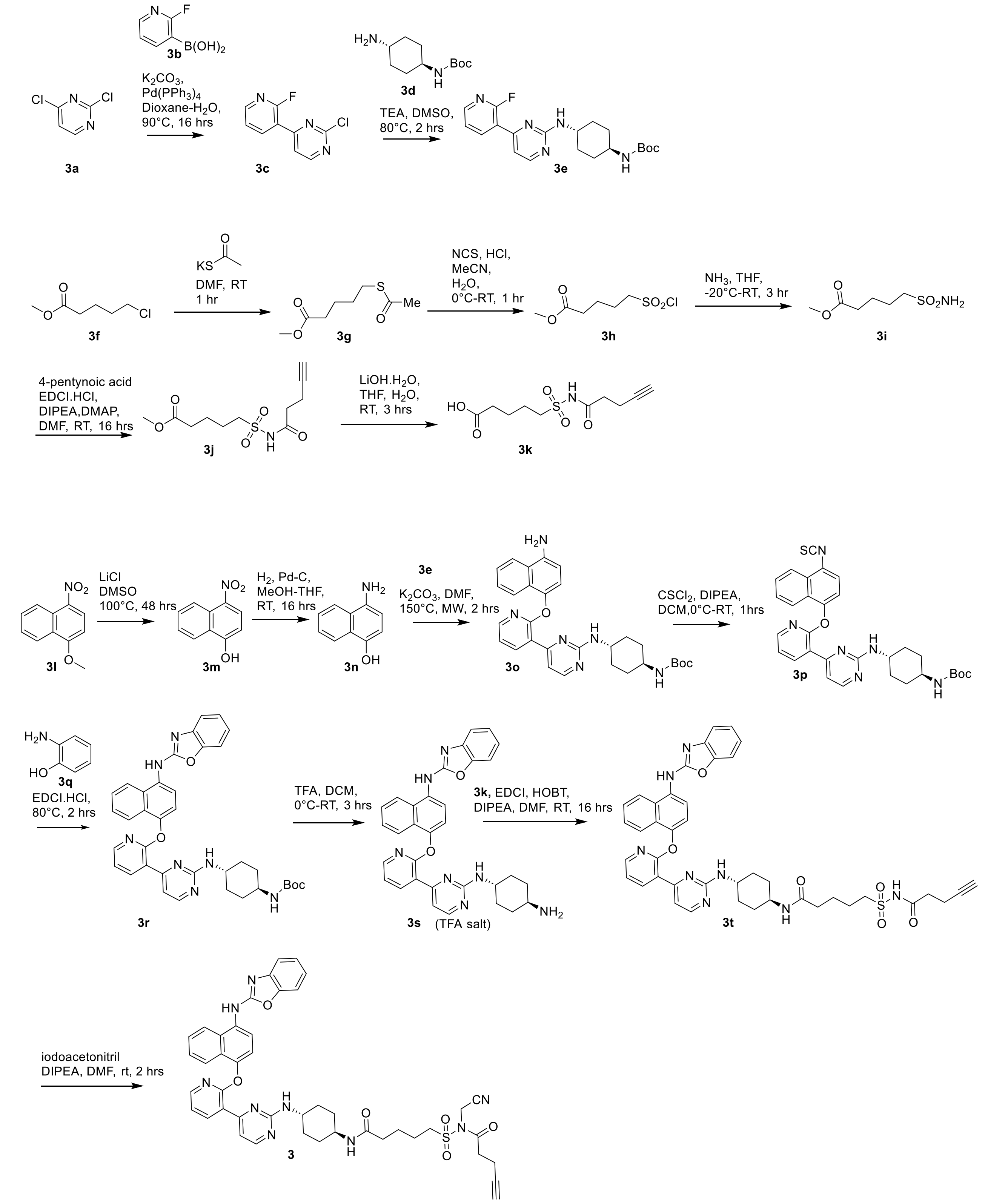

2-chloro-4-(2-fluoropyridin-3-yl)pyrimidine (3c)

To a stirred solution of 2,4-dichloropyrimidine **3a** (5.0 g, 33.56 mmol, 1.0 equiv.) and (2-fluoropyridin-3-yl)boronic acid **3b** (4.7 g, 33.56 mmol, 1.0 equiv.) in 1,4-dioxane (40 mL) and water (10 mL) was added potassium carbonate (9.3 g, 67.11 mmol, 2.0 equiv.) and degassed with argon. $Pd(PPh_3)_4$ (3.9 g, 3.36 mmol, 0.1 equiv.) was added under inert atmosphere. The resulting mixture was heated at 90°C for 16 hrs. After completion, reaction mixture was diluted with ethyl acetate, filtered through a short pad of celite and washed with ethyl acetate. Combined organic layers were washed with water, brine, dried over anhydrous sodium sulphate, filtered and concentrated under reduced pressure. Crude product was purified by column chromatography (silica-gel; 0-10% ethyl acetate - hexane) to afford 2-chloro-4-(2-fluoropyridin-3-yl)pyrimidine **3c** (2.5 g, 11.90 mmol, 35% yield) as light brown solid. **^{1}H NMR** (400 MHz, DMSO-d_6) δ 8.92 (d, *J* = 5.2 Hz, 1H), 8.63 – 8.58 (m, 1H) , 8.46 (d, *J* = 4.64 Hz, 1H), 8.03 – 8.01 (m, 1H), 7.62 – 7.58 (m, 1H). **MS** (ESI+): *m/z* 210.1 $[M+H]^+$.

***tert*-butyl (*1s*,*4r*)-4-(4-(2-fluoropyridin-3-yl)pyrimidin-2-yl)amino) cyclohexyl) carbamate (3e)**

To the stirred solution of 2-chloro-4-(2-fluoropyridin-3-yl)pyrimidine **3c** (2.5 g, 11.96 mmol, 1.0 equiv.) in DMSO (15 mL), TEA (2.5 mL, 17.94 mmol, 1.5 equiv.) and tert-butyl ((1r,4r)-4-aminocyclohexyl)carbamate **3d** (2.6 g, 11.96 mmol, 1.0 equiv.) were added at rt. Resulting mixture was heated at 80°C for 2 hrs. After completion, reaction mixture was diluted with ethyl acetate, washed with water and brine, dried over anhydrous sodium sulphate, filtered and concentrated under reduced pressure. Crude mass was purified by column chromatography (silica-gel; 0-10% EA-DCM) to afford tert-butyl (*1r*,*4r*)-4-(4-(2-fluoropyridin-3-yl)pyrimidin-2-yl)amino) cyclohexyl) carbamate **3e** (1.3 g, 3.36 mmol, 28% yield). **^{1}H NMR** (400 MHz, DMSO-d_6) δ 8.49 (brs, 1H), 8.40 – 8.35 (m, 2H), 7.55 (brs, 1H), 7.25 (d, *J* = 7.88 Hz, 1H), 6.98 (d, *J* = 2.52 Hz, 1H), 6.74 (d, *J* = 7.96 Hz, 1H), 3.70 – 3.68 (m, 1H), 3.22 – 3.20 (m, 1H), 1.99 – 1.78 (m, 4H), 1.38 (s, 9H), 1.34 – 1.23 (m, 4H). **MS** (ESI+): *m/z* 388.1 $[M+H]^+$.

5-(acetylthio)pentanoate (3g)

To a stirred solution of methyl 5-chloropentanoate **3f** (1.0 g, 6.67 mmol, 1.0 equiv.) in DMF (10 mL) was added potassium thioacetate (0.84 g, 7.33 mmol, 1.1 equiv.) at rt under inert atmosphere. The resulting mixture was stirred at rt for 1 hr. After completion, reaction mixture was quenched with ice-cooled water and extracted with ethyl acetate. Combined organic layers were washed with water, brine, dried over anhydrous sodium sulphate, filtered and concentrated under reduced pressure. Crude product was purified by column chromatography (silica-gel; 20-30% ethyl acetate - hexane) to afford methyl 5-(acetylthio)pentanoate **3g** (0.72 g, 3.79 mmol, 57% yield) as colourless liquid. **^{1}H NMR** (400 MHz, DMSO-d_6) δ 3.58 (s, 3H), 2.83 (d, *J* = 6.84 Hz, 2H), 2.32 (s, 3H), 2.30 – 2.29 (m, 2H), 1.56 – 1.51 (m, 4H). **MS** (ESI+): *m/z* 191.2 $[M+H]^+$.

5-(chlorosulfonyl)pentanoate (3h)

To a stirred solution of methyl 5-(acetylthio)pentanoate, **3g** (4.5 g, 23.68 mmol, 1.0 equiv.) in MeCN (40 mL), was drop-wise added 2N aqueous HCl (4.0 mL) at 0-5°C under inert atmosphere. *N*-Chlorosuccinimide (12.6 g, 94.70 mmol, 4.0 equiv.) was then added portion-wise at 0-5°C. Resulting mixture was stirred at rt for 1 hr. After completion, reaction mixture was quenched with ice-cooled water and extracted with diethyl ether. Combined organic layer was washed with saturated aq. $NaHCO_3$, water, brine, dried over anhydrous sodium sulphate, filtered and concentrated under reduced pressure to afford methyl 5-(chlorosulfonyl)pentanoate **3h** (4.0 g, 18.70 mmol, 79% yield) as gummy material. **^{1}H NMR** (400 MHz, DMSO-d_6) δ 3.56 (s, 3H), 2.64 – 2.61 (m, 2H), 2.30 – 2.27 (m, 2H), 1.59 – 1.58 (m, 4H). **MS** (ESI+): *m/z* 214.0 $[M+H]^+$.

Methyl 5-sulfamoylpentanoate (3i)

A solution of methyl 5-(chlorosulfonyl)pentanoate, **3h** (4.0 g, 18.69 mmol, 1.0 equiv.) in THF (20 mL) was taken in a sealed tube, cooled to -20°C and NH_3 gas was purged for 0.5 h. Resulting mixture was stirred at rt for 3 hrs. After completion, reaction mixture was concentrated under reduced pressure, diluted with ethyl acetate. Organic layer was washed with water, brine, dried over anhydrous sodium sulphate, filtered and concentrated under reduced pressure. Crude product was purified by column chromatography (silica-gel; 20-50% ethyl acetate - hexane) to afford methyl 5-sulfamoylpentanoate **3i** (2.5 g, 12.82 mmol, 68% yield) as off white solid. **^{1}H NMR** (400 MHz, DMSO-d_6) δ 6.74 (s, 2H), 3.59 (s, 3H), 2.98 – 2.94 (m, 2H), 2.34 (t, *J* = 7.24 Hz, 2H), 1.68 – 1.60 (m, 4H) ppm. **MS** (ESI+): *m/z* 196.2 $[M+H]^+$.

Methyl 5-(*N*-(pent-4-ynoyl)sulfamoyl)pentanoate (3j)

To a stirred solution of pent-4-ynoic acid (0.75 g, 7.65 mmol, 1.0 equiv.) in DMF (5 mL), were added EDCI.HCl (2.2 g, 11.48 mmol, 1.5 equiv.), DIPEA (3.3 mL, 19.13 mmol, 2.5 equiv.) and DMAP (0.09 g, 0.76 mmol, 0.1 equiv.) at rt under inert atmosphere, stirred for 5 min and methyl 5-sulfamoylpentanoate **3i** (1.5 g, 7.65 mmol, 1.0 equiv.) was added. The resulting solution was stirred at rt for 16 hrs. After completion, reaction mixture was quenched with ice-cooled water and extracted with ethyl acetate. Combined organic layers were washed with water, brine, dried over anhydrous sodium sulphate, filtered and concentrated under reduced pressure. Crude mass was purified by column chromatography (silica-gel; 20-60% ethyl acetate - hexane) to afford methyl 5-(*N*-(pent-4-ynoyl)sulfamoyl)pentanoate **3j** (0.4 g, 1.45 mmol, 19% yield) as white solid. **^{1}H NMR** (400 MHz, DMSO-d_6) δ 11.68 (s, 1H), 3.59 (s, 3H), 3.38 – 3.32 (m, 2H), 2.82 (t, J = 2.6 Hz, 1H), 2.49 – 2.47 (m, 2H), 2.40 – 2.32 (m, 4H), 1.69 – 1.60 (m, 4H). **MS** (ESI+): *m/z* 276.0 $[M+H]^+$.

5-(*N*-(pent-4-ynoyl)sulfamoyl)pentanoic acid (3k)

To a stirred solution of methyl 5-(*N*-(pent-4-ynoyl)sulfamoyl)pentanoate **3j** (1 g, 3.64 mmol, 1.0 equiv.) in THF (10 mL) and H_2O (4 mL), was added $LiOH.H_2O$ (0.46 g, 10.91 mmol, 3.0 equiv.) at rt. Resulting solution was stirred at rt for 3 hrs. After completion, reaction mixture was concentrated under reduced pressure and diluted

with water. The aqueous part was washed with ethyl acetate and organic part was discarded. Aqueous portion was acidified with 1(N) HCl and extracted with ethyl acetate. Combined organic part was dried over anhydrous sodium sulphate, filtered and concentrated under reduced pressure to afford 5-(*N*-(pent-4-ynoyl)sulfamoyl)pentanoic acid **3k** (0.8 g, 3.14 mmol, 86% yield) as white solid. **^{1}H NMR** (400 MHz, DMSO-d_6) δ 12.06 (s, 1H), 11.67 (s, 1H), 3.38 – 3.31 (m, 2H), 2.80 (t, J = 2.48 Hz, 1H), 2.49 – 2.47 (m, 2H), 2.40 – 2.38 (m, 2H), 2.25 – 2.22 (m, 2H), 1.67 – 1.57 (m, 4H). **MS**(ESI+): *m/z* 261.9 $[M+H]^+$.

4-nitronaphthalen-1-ol (3m)

To a stirred solution of 1-methoxy-4-nitronaphthalene **3l** (0.25 g, 1.232 mmol, 1.00 equiv.) in DMSO (2 mL) was added LiCl (0.26 g, 6.158 mmol, 5.00 equiv.). The resulting mixture was heated at 100°C for 48 h. After completion, reaction mixture was diluted with ethyl acetate and washed with water and brine. Organic part was separated, dried over anhydrous sodium sulphate, filtered, and concentrated under reduced pressure. Crude mass was purified by column chromatography (silica-gel; 70% ethyl acetate - hexane) to afford 4-nitronaphthalen-1-ol **3m** (0.1 g, 0.529 mmol, 43% yield) as light-yellow solid. **^{1}H NMR** (400 MHz, DMSO-d_6) δ 11.93 (s, 1H), 8.67 (d, *J* = 8.76 Hz, 1H), 8.41 (d, *J* = 8.6 Hz, 1H), 8.33 (d, *J* = 8.4 Hz, 1H), 7.81 (t, *J* = 8.28 Hz, 1H), 7.65 (t, *J* = 8.04 Hz, 1H), 6.99 (d, *J* = 8.68 Hz, 1H). **MS** (ESI+): *m/z* 190.1 $[M+H]^+$.

4-aminonaphthalen-1-ol (3n)

To a degassed solution of 4-nitronaphthalen-1-ol **3m** (0.5 g, 2.64 mmol, 1.0 equiv.) in MeOH (3 mL) and THF (3 mL) was added 10% Pd-C (0.2 g). The resulting mixture was stirred under hydrogen balloon pressure at rt for 16 hrs. After completion, reaction mixture was filtered through a short pad of celite, washed with ethyl acetate and concentrated under reduced pressure. Crude mass was purified by flash chromatography (silica-gel; 5% MeOH-DCM) to afford 4-aminonaphthalen-1-ol **3n** (0.4 g, 2.51 mmol, 95% yield) as light brown solid. **MS** (ESI+): *m/z* 160.1 $[M+H]^+$.

***Tert*-butyl ((1*r*,4*r*)-4-((4-(2-((4-aminonaphthalen-1-yl)oxy)pyridin-3-yl)pyrimidin-2-yl)amino)cyclohexyl) carbamate (3o)**

To a stirred solution of tert-butyl (*1r*,*4r*)-4-(4-(2-fluoropyridin-3-yl)pyrimidin-2-yl)amino) cyclohexyl) carbamate **3e** (0.10 g, 0.26 mmol, 1.0 equiv.) in DMF (1 mL), was added potassium carbonate (0.054 g, 0.39 mmol, 1.5 equiv.) at rt and degassed with argon. 4-aminonaphthalen-1-ol **3n** (0.041 g, 0.26 mmol, 1.0 equiv.) was added under inert atmosphere. The resulting mixture was heated under microwave irradiation at 150°C for 2 hrs. After completion, reaction mixture was diluted with ethyl acetate and washed with ice cold water. Organic part was separated, dried over anhydrous sodium sulphate, filtered, and concentrated under reduced pressure. Crude product was purified by combiflash chromatography (silica-gel; 40-60% ethyl acetate - hexane) to afford *tert*-butyl ((1*r*,4*r*)-4-((4-(2-((4-aminonaphthalen-1-yl)oxy)pyridin-3-yl)pyrimidin-2-yl)amino)cyclohexyl) carbamate **3o** (0.035 g, 0.067 mmol, 26% yield) as sticky brown solid. **^{1}H NMR** (400 MHz, DMSO-d_6) δ 8.38 – 8.36 (m, 2H),

8.11 (d, *J* = 9 Hz, 1H), 8.04 (brs, 1H), 7.58 – 7.56 (m, 2H), 7.39 – 7.37 (m, 2H), 7.22 – 7.21 (m, 1H), 7.13 – 7.12 (m, 1H), 7.02 – 7.00 (m, 1H), 6.73 – 6.71 (m, 1H), 6.67 (d, *J* = 7.56 Hz, 1H), 5.67 (s, 2H), 3.9 – 3.8 (m, 1H), 3.2 – 3.1 (m, 1H), 1.98 – 1.96 (m, 2H), 1.79 – 1.77 (m, 2H), 1.39 (s, 9H), 1.31 – 1.26 (m, 4H). **MS** (ESI+): *m/z* 527.2 $[M+H]^+$.

***Tert*-butyl ((*1r,4r*)-4-((4-(2-((4-isothiocyanatonaphthalen-1-yl)oxy)pyridin-3-yl)pyrimidin-2-yl)amino) cyclohexyl)carbamate (3p)**

To a stirred solution of *tert*-butyl ((1*r*,4*r*)-4-((4-(2-((4-aminonaphthalen-1-yl)oxy)pyridin-3-yl)pyrimidin-2-yl)amino)cyclohexyl)carbamate **3o** (0.18 g, 0.34 mmol, 1.0 equiv.) in DCM (5 mL) was added DIPEA (0.18 mL, 1.03 mmol, 3.0 equiv.) under inert atmosphere. The reaction mixture was cooled to 0°C and thiophosgene (0.029 ml, 0.38 mmol, 1.1 equiv.) was added dropwise. The resulting mixture was stirred at rt for 1 hr. After completion, reaction mixture was poured into ice-cooled water and extracted by DCM. Combined organic part was washed with water, brine, dried over anhydrous sodium sulphate, filtered and concentrated under reduced pressure. Crude product was purified by flash chromatography (silica-gel; 20-30% ethyl acetate - hexane) to afford *tert*-butyl ((1*r*,4*r*)-4-((4-(2-((4-isothiocyanatonaphthalen-1-yl)oxy)pyridin-3-yl)pyrimidin-2-yl)amino) cyclohexyl) carbamate **3p** (0.13 g, 0.23 mmol, 66% yield) as brown solid. **^{1}H NMR** (400 MHz, DMSO-d_6) δ 8.42 (d, *J* = 7.12 Hz, 1H), 8.36 (d, *J* = 5 Hz, 1H), 8.13 – 8.08 (m, 2H) , 7.96 (d, *J* = 8.24 Hz, 1H), 7.81 – 7.73 (m, 2H), 7.68 – 7.66 (m, 1H), 7.36 – 7.29 (m, 3H), 7.15 (d, *J* = 7.64 Hz, 1H), 6.71 (d, *J* = 6.88 Hz, 1H), 3.9 – 3.7 (m, 1H), 3.3 – 3.1 (m, 1H), 1.99 – 1.93 (m, 2H), 1.81 – 1.79 (m, 2H), 1.39 (s, 9H), 1.35 – 1.18 (m, 4H). **MS** (ESI+): *m/z* 569.2 $[M+H]^+$.

***Tert*-butyl ((*1r,4r*)-4-((4-(2-((4-(benzo[d]oxazol-2-ylamino)naphthalen-1-yl)oxy)pyridin-3-yl)pyrimidin-2-yl)amino)cyclohexyl)carbamate (3r)**

To a stirred solution of *tert*-butyl ((1*r*,4*r*)-4-((4-(2-((4-isothiocyanatonaphthalen-1-yl)oxy)pyridin-3-yl)pyrimidin-2-yl)amino)cyclohexyl)carbamate **3p** (0.13 g, 0.23 mmol, 1.0 equiv.) in MeCN (5 mL), were added 2-aminophenol **3q** (0.027 g, 0.25 mmol, 1.1 equiv.) and EDCI.HCl (0.065 g, 0.34 mmol, 1.5 equiv.) at rt under inert atmosphere. The resulting mixture was stirred at 80°C for 2 hrs. After completion, reaction mixture was diluted with water and extracted with ethyl acetate. Combined organic part was washed with water, brine, dried over anhydrous sodium sulphate, filtered and concentrated under reduced pressure. Crude mass was purified by flash chromatography (silica-gel; 50-60% ethyl acetate - hexane) to afford *tert*-butyl ((1*r*,4*r*)-4-((4-(2-((4-(benzo[d]oxazol-2-ylamino) naphthalen-1-yl)oxy) pyridin-3-yl) pyrimidin-2-yl)amino) cyclohexyl)carbamate **3r** (0.06 g, 0.093 mmol, 41% yield) as brown solid. **^{1}H NMR** (400 MHz, DMSO-d_6) δ 10.45 (s, 1H), 8.44 (d, *J* = 7.96 Hz, 1H), 8.39 (d, *J* = 5.1 Hz, 1H), 8.29 (d, *J* = 8.44 Hz, 1H), 8.12 – 8.11 (m, 1H) , 8.06 (d, *J* = 8.2 Hz, 1H), 7.85 (d, *J* = 7.72 Hz, 1H), 7.63 – 7.49 (m, 3H), 7.39 – 7.37 (m, 4H), 7.2 – 7.0 (m, 3H), 6.73 (d, *J* = 7.92 Hz, 1H), 3.75 (brs, 1H), 3.22 – 3.21 (m, 1H), 1.99 – 1.97 (m, 2H), 1.84 – 1.82 (m, 2H), 1.38 (s, 9H), 1.33 – 1.25 (m, 4H). **MS** (ESI+): *m/z* 644.3 $[M+H]^+$.

(1*r*,4*r*)-*N*[1]-(4-(2-((4-(benzo[d]oxazol-2-ylamino)naphthalen-1-yl)oxy)pyridin-3-yl)pyrimidin-2-yl) cyclohexane-1,4-diamine, TFA salt (3s)

To a stirred solution of tert-butyl ((1*r*,4*r*)-4-((4-(2-((4-(benzo[d]oxazol-2-ylamino)naphthalen-1-yl)oxy)pyridin-3-yl)pyrimidin-2-yl)amino)cyclohexyl)carbamate **3r** (0.06 g, 0.093 mmol, 1.0 equiv.) in DCM (1.5 mL), was added trifluoroacetic acid (0.6 mL, excess) dropwise at 0°C under argon atmosphere. The resulting mixture was stirred at rt for 3 h. After completion, reaction mixture was concentrated under reduced pressure to afford (1*r*,4*r)*-*N*[1]-(4-(2-((4-(benzo[d]oxazol-2-ylamino)naphthalen-1-yl)oxy)pyridin-3-yl)pyrimidin-2-yl)cyclohexane-1,4-diamine **3s** (TFA salt, 0.045 g, 0.083 mmol, 88% yield) as brown sticky liquid. **MS** (ESI+): *m/z* 542.6 $[M+H]^+$.

***N*-((5-(((1*r*,4*r*)-4-((4-(2-((4-(benzo[d]oxazol-2-ylamino)naphthalen-1-yl)oxy)pyridin-3-yl) pyrimidin-2-yl)amino)cyclohexyl)amino)-5-oxopentyl)sulfonyl)pent-4-ynamide (3t)**

To a stirred solution of 5-(*N*-(pent-4-ynoyl)sulfamoyl)pentanoic acid **3k** (0.021 g, 0.08 mmol, 1.0 equiv.) in DMF (1.5 mL), were added EDCI.HCl (0.023 g, 0.12 mmol, 1.5 equiv.), HOBT (0.015 g, 0.11 mmol, 1.4 equiv.) and DIPEA (0.028 mL, 0.16 mmol, 2.0 equiv.) at rt under inert atmosphere, stirred for 5 min and (1*r*,4*r*)-*N*[1]-(4-(2-((4-(benzo[*d*]oxazol-2-ylamino)naphthalen-1-yl)oxy)pyridin-3-yl)pyrimidin-2-yl)cyclohexane-1,4-diamine **3s** (TFA salt , 0.044 g, 0.08 mmol, 1.0 equiv.) was added. The resulting solution was stirred at rt for 16 hrs. After completion, reaction mixture concentrated under reduced pressure. Crude mass was purified by prep-HPLC to afford *N*-((5-(((1*r*,4*r*)-4-((4-(2-((4-(benzo[d]oxazol-2-ylamino)naphthalen-1-yl)oxy)pyridin-3-yl)pyrimidin-2-yl)amino)cyclohexyl) amino)-5-oxopentyl)sulfonyl)pent-4-ynamide **3** (0.025 g, 0.032 mmol, 40% yield) as white solid. **^{1}H NMR** (400 MHz, DMSO-d_6) δ 11.67 (s, 1H), 10.44(s, 1H), 8.47 – 8.45 (m, 1H), 8.40 (d, *J* = 5.04 Hz, 1H), 8.29 (d, *J* = 8.32 Hz, 1H), 8.12 (d, *J* = 3.28 Hz, 1H), 8.06 (d, *J* = 7.96 Hz, 1H), 7.86 (d, *J* = 8.16 Hz, 1H), 7.71 (d, *J* = 8.32 Hz, 1H), 7.70 – 7.49 (m, 3H), 7.41 – 7.12 (m, 7H), 3.85 – 3.79 (m, 1H), 3.53 – 3.51 (m, 1H), 3.42 – 3.41 (m, 2H), 2.82 (s, 1H), 2.41 – 2.39 (m, 2H), 2.08 – 1.99 (m, 4H), 1.84 – 1.81 (m, 2H), 1.61 – 1.59 (m, 4H), 1.43 – 1.26 (m, 4H). **MS** (ESI+): *m/z* 787.5 $[M+H]^+$

***N*-((5-(((*1r*,*4r*)-4-((4-(2-((4-(benzo[d]oxazol-2-ylamino)naphthalen-1-yl)oxy)pyridin-3-yl)pyrimidin-2-yl) amino)cyclohexyl)amino)-5-oxopentyl)sulfonyl)-N-(cyanomethyl)pent-4-ynamide (3)**

The **3t** (11.4 mg, 0.014 mmol, 1.0 equiv.) was dissolved in 500 µL dry DMF and treated with iodoacetonitrile (21 µL, 0.29 mmol, 20.0 equiv.) and DIPEA (50 µL, 0.29 mmol, 20.0 equiv.). The mixture was stirred overnight at rt. After overnight stirring, four peaks are observed. The desired product was isolated by preparative HPLC (10-100 % acetonitrile in water + 0.1% formic acid). **MS** (ESI+): *m/z* 865.57 $[M+H]^+$.

3.1.4 Compound **4** (MEK2 NASA probe)

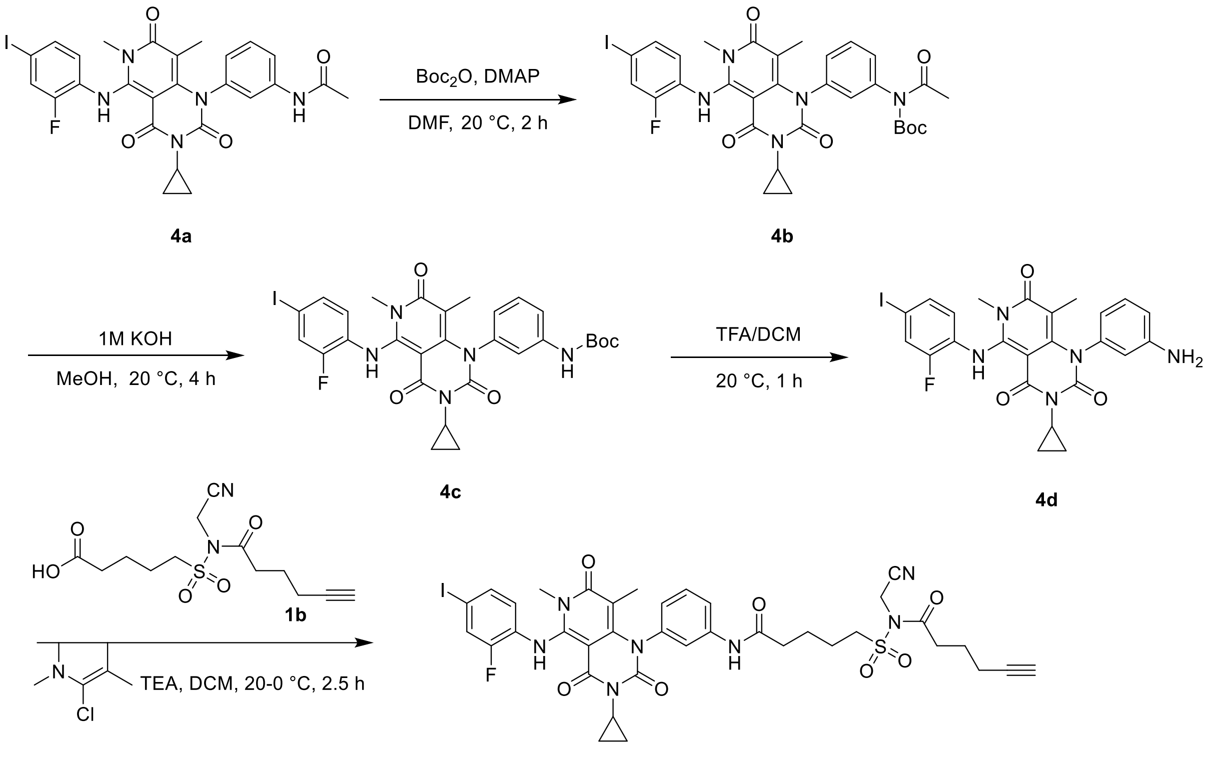

4

***Tert*-butyl acetyl(3-(3-cyclopropyl-5-((2-fluoro-4-iodophenyl)amino)-6,8-dimethyl-2,4,7-trioxo-3,4,6,7-tetrahydropyrido[4,3-d]pyrimidin-1(2*H*)-yl)phenyl)carbamate (4b)**

To a solution of *N*-(3-(3-cyclopropyl-5-((2-fluoro-4-iodophenyl)amino)-6,8-dimethyl-2,4,7-trioxo-3,4,6,7-tetrahydropyrido[4,3-d]pyrimidin-1(2*H*)-yl)phenyl)acetamide (1.75 g, 2.84 mmol, 1.0 equiv.) and DMAP (0.69 g, 5.69 mmol, 2 equiv.) in DMF (10 mL) was added a solution of Boc$_2$O (1.86 g, 8.53 mmol, 1.96 mL) and DMF (5 mL) dropwise over 1 min. Then the mixture was degassed and purged with N_2 for 3 times, and then the mixture was stirred at 20 °C for 2 hrs under N_2 atmosphere. LC-MS showed starting material was consumed completely. Several new peaks were shown on LC-MS and one peak with the desired mass was detected. The reaction mixture was diluted with ethyl acetate (EA) 60 mL and extracted with EA (80 mL × 3). The combined organic layers were washed with brine (100 mL × 3), dried over Na_2SO_4, filtered and concentrated under reduced pressure to give crude product *tert*-butyl acetyl(3-(3-cyclopropyl-5-((2-fluoro-4-iodophenyl)amino)-6,8-dimethyl-2,4,7-trioxo-3,4,6,7-tetrahydropyrido[4,3-d]pyrimidin-1(2*H*)-yl)phenyl)carbamate (2.68 g, 2.62 mmol, 92% yield, 70% purity) as yellow gum. **^{1}H NMR** (400 MHz, DMSO-d_6) δ 11.12 - 10.93 (m, 1H), 7.77 (dd, J = 2.0, 10.4 Hz, 1H), 7.56 - 7.51 (m, 1H), 7.50 - 7.45 (m, 1H), 7.38 (td, J = 1.6, 8.4 Hz, 1H), 7.21 - 7.17 (m, 2H), 6.92 (t, J = 8.8 Hz, 1H), 3.07 (s, 3H), 2.73 (s, 3H), 2.63 - 2.60 (m, 1H), 2.47 (s, 3H), 1.35 (s, 9H), 0.95 (d, J = 7.2 Hz, 2H), 0.66 (s, 2H). **MS** (ESI+)**:** *m/z* 716.7 $[M+H]^+$.

***Tert*-butyl (3-(3-cyclopropyl-5-((2-fluoro-4-iodophenyl)amino)-6,8-dimethyl-2,4,7-trioxo-3,4,6,7-tetrahydropyrido[4,3-d]pyrimidin-1(2H)-yl)phenyl)carbamate (4c)**

To a solution of *tert*-butyl acetyl(3-(3-cyclopropyl-5-((2-fluoro-4-iodophenyl)amino)-6,8-dimethyl-2,4,7-trioxo-3,4,6,7-tetrahydropyrido[4,3-d]pyrimidin-1(2*H*)-yl)phenyl) carbamate (2.68 g, 2.62 mmol, 70% purity) in MeOH (20 mL) was added KOH (1 M, 13 mL, excess) at 20 °C for 4 hrs. LC-MS showed **4b** was consumed completely. Several new peaks were shown on LC-MS and one peak with the desired mass was detected. The reaction mixture was into 200 mL water and solids are separated out, filtered, and the solid was the products. The solids are pulled dry by an oil pump to remove excess water. The residue was purified by flash silica gel chromatography (ISCO®; 40 g SepaFlash® Silica Flash Column, Eluent of 0~45% EA/PE @ 80 mL/min) to give *tert*-butyl (3-(3-cyclopropyl-5-((2-fluoro-4-iodophenyl)amino)-6,8-dimethyl-2,4,7-trioxo-3,4,6,7-tetrahydropyrido [4,3-d]pyrimidin-1(2*H*)-yl)phenyl)carbamate (1.51 g, 2.24 mmol, 85% yield) as off-yellow solid. **^{1}H NMR** (400 MHz, DMSO-d_6) δ 11.09 (s, 1H), 9.50 (s, 1H), 7.78 (dd, *J* = 2.0, 10.4 Hz, 1H), 7.61 - 7.47 (m, 2H), 7.43 - 7.35 (m, 1H), 7.35 - 7.26 (m, 1H), 6.99 - 6.88 (m, 2H), 3.33 - 3.32 (m, 3H), 3.07 (s, 3H), 2.65 - 2.58 (m, 1H), 1.46 (s, 9H), 0.95 (q, *J* = 7.2 Hz, 2H), 0.72 - 0.61 (m, 2H). **MS** (ESI+)**:** *m/z* 674.3 $[M+H]^+$.

1-(3-aminophenyl)-3-cyclopropyl-5-((2-fluoro-4-iodophenyl)amino)-6,8-dimethylpyrido[4,3-d]pyrimidine-2,4,7(1*H*,3*H*,6*H*)-trione (4d)

To a solution of *tert*-butyl (3-(3-cyclopropyl-5-((2-fluoro-4-iodophenyl)amino)-6,8-dimethyl-2,4,7-trioxo-3,4,6,7-tetrahydropyrido[4,3-d]pyrimidin-1(2*H*)-yl)phenyl)carbamate (1.51 g, 2.24 mmol) in DCM (5 mL) was added TFA (3.08 g, 27.01 mmol, 2 mL) at 20 °C for 1 hr. LC-MS showed **4c** was consumed completely. Several new peaks were shown on LC-MS and one peak with the desired mass was detected. The pH was basified to 7~8 with saturated $NaHCO_3$ (50 mL). The mixture was extracted with DCM (3 × 60 mL). The organic layer was dried over Na_2SO_4, filtered and concentrated under vacuum to give 1-(3-aminophenyl)-3-cyclopropyl-5-((2-fluoro-4-iodophenyl)amino)-6,8-dimethylpyrido[4,3-d]pyrimidine-2,4,7(1*H*,3*H*,6*H*)-trione (1.2 g, 2.09 mmol, 93% yield) as off-yellow solid. The crude product was used in the next step without further purification. **^{1}H NMR** (400 MHz, DMSO-d_6) δ 11.06 (s, 1H), 7.79 (dd, *J* = 2.0, 10.4 Hz, 1H), 7.55 (dd, *J* = 1.2, 8.4 Hz, 1H), 7.23 (t, *J* = 8.4 Hz, 1H), 6.91 (t, *J* = 8.8 Hz, 1H), 6.85 - 6.75 (m, 3H), 3.07 (s, 3H), 2.65 - 2.58 (m, 1H), 1.30 (s, 3H), 1.00 - 0.90 (m, 2H), 0.69 - 0.61 (m, 2H). **MS** (ESI+)**:** *m/z* 574.2 $[M+H]^+$.

***N*-(cyanomethyl)-*N*-((5-((3-(3-cyclopropyl-5-((2-fluoro-4-iodophenyl)amino)-6,8-dimethyl-2,4,7-trioxo-3,4,6,7-tetrahydropyrido[4,3-d]pyrimidin-1(2*H*)-yl)phenyl)amino)-5-oxopentyl) sulfonyl)hex-5-ynamide (compound 4)**

To a solution of 1-chloro-*N*,*N*,2-trimethyl-prop-1-en-1-amine (111.9 mg, 0.84 mmol, 110.8 µL) in DCM (0.5 mL) under nitrogen was added 5-[cyanomethyl(hex-5-ynoyl)sulfamoyl]pentanoic acid (263.17 mg, 0.42 mmol, 50%

purity) at 20 °C for 0.5 hr. Then added to a solution of 1-(3-aminophenyl)-3-cyclopropyl-5-((2-fluoro-4-iodophenyl)amino)-6,8-dimethylpyrido[4,3-d]pyrimidine-2,4,7(1*H*,3*H*,6*H*)-trione (160.0 mg, 0.28 mmol) in DCM (0.5 mL) at 0 °C. TEA (84.7 mg, 0.84 mmol, 116.5 µL) was then added, and the mixture was stirred at 0 °C for 2 hrs. LC-MS showed no **4d** remained. Several new peaks were shown on LC-MS and the desired compound was detected. The reaction mixture was filtered over Celite, and the filtrate was blow-dried with nitrogen to obtain the crude product. The crude product was purified by prep-HPLC (column: Welch Xtimate C18 150*25mm*5um; mobile phase: [water (TFA)-ACN]; B%: 50%-70%, 10min). to give *N*-(cyanomethyl)-*N*-((5-((3-(3-cyclopropyl-5-((2-fluoro-4-iodophenyl)amino)-6,8-dimethyl-2,4,7-trioxo-3,4,6,7-tetrahydropyrido[4,3-d]pyrimidin-1(2*H*)-yl)phenyl)amino)-5-oxopentyl) sulfonyl)hex-5-ynamide (77.2 mg, 0.087 mmol, 31.1% yield, 97.7% purity) as a white solid. **^{1}H NMR** (400 MHz, DMSO-d_6) δ 11.07 (s, 1H), 10.08 (s, 1H), 7.78 (dd, *J* = 1.6, 10.4 Hz, 1H), 7.68 - 7.45 (m, 3H), 7.36 (t, *J* = 8.0 Hz, 1H), 7.03 (d, *J* = 8.0 Hz, 1H), 6.92 (t, *J* = 8.8 Hz, 1H), 4.74 (s, 2H), 3.69 (t, *J* = 7.2 Hz, 2H), 3.08 (s, 3H), 2.86 (t, *J* = 7.2 Hz, 2H), 2.81 (t, *J* = 2.8 Hz, 1H), 2.62 (tt, *J* = 3.6, 6.8 Hz, 1H), 2.37 (t, *J* = 6.8 Hz, 2H), 2.23 (dt, *J* = 2.8, 7.2 Hz, 2H), 1.75 (td, *J* = 6.8, 14.0 Hz, 6H), 1.25 (s, 3H), 0.95 (q, *J* = 6.4 Hz, 2H), 0.67 (s, 2H). **MS** (ESI+): *m/z* 870.4 $[M+H]^+$.

3.1.5 Compound **5** (LIMK1 NASA probe)

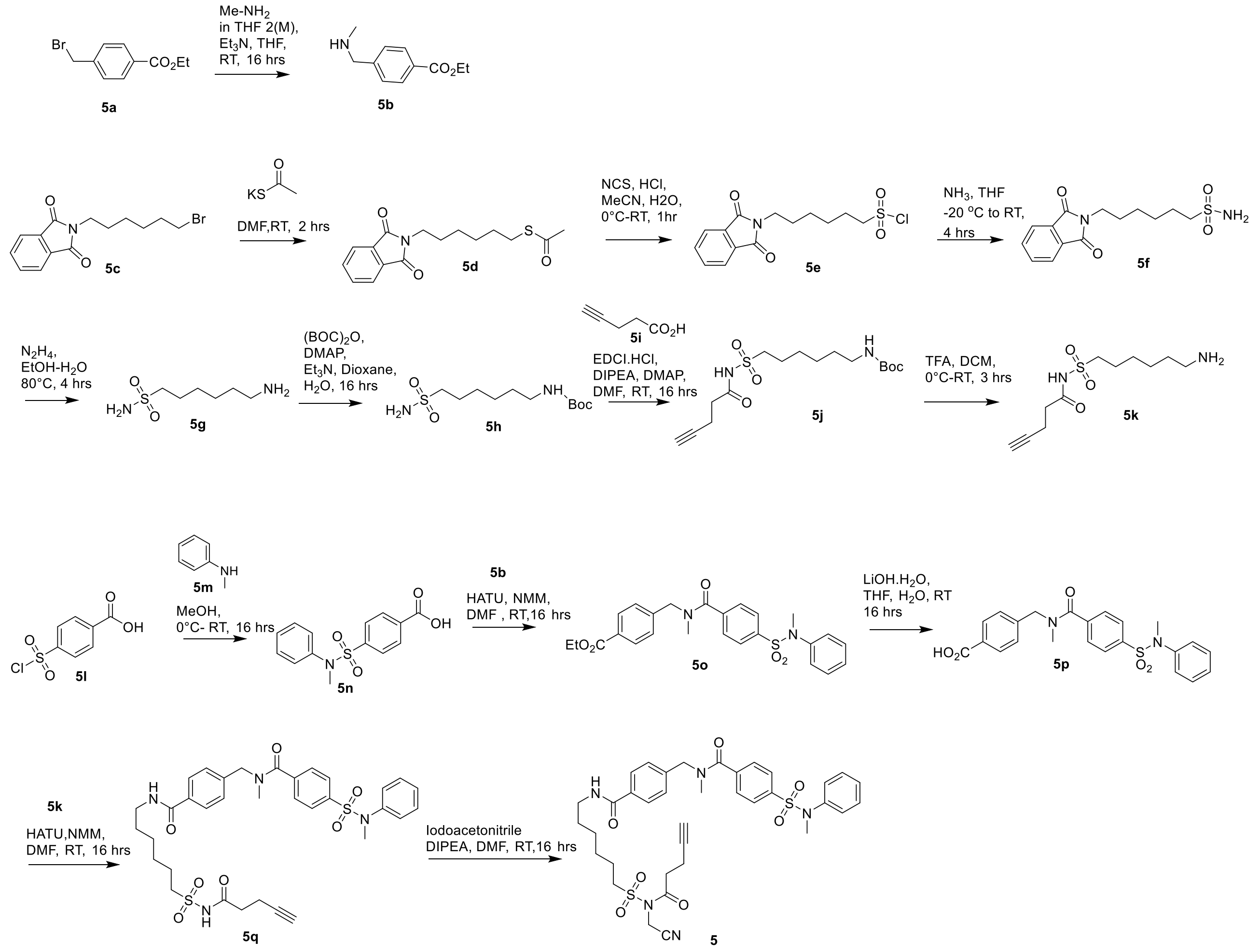

Ethyl 4-((methylamino)methyl)benzoate (5b)

To a stirred solution of ethyl 4-(bromomethyl)benzoate **5a** (5.0 g, 20.58 mmol, 1.0 equiv.) in THF (100 mL) was added TEA (5.7 mL, 41.15 mmol, 2.0 equiv.) followed by the addition of methyl amine (2M THF, 103 mL, 10.0 equiv.). The resulting mixture was stirred at rt for 16 hrs. After completion, reaction mixture was concentrated under reduced pressure and diluted with 10% MeOH in DCM. Organic portion was washed with saturated aq. $NaHCO_3$ solution, water, brine, dried over anhydrous sodium sulphate, filtered and concentrated under reduced pressure. Crude product was purified by column chromatography (silica-gel; 10-30% ethyl acetate - hexane) to afford ethyl 4-((methylamino)methyl)benzoate **5b** (2.8 g, 14.50 mmol, 70% yield) as light brown solid. **^{1}H NMR** (400 MHz, $CDCl_3$) δ 7.89 (d, J = 8.16 Hz, 2H), 7.45 (d, J = 8.08 Hz, 2H), 4.32 – 4.27 (m, 2H), 3.69 (s, 2H), 2.25 (s, 2H), 1.31 (t, J = 7.12 Hz, 3H). **MS** (ESI+): *m/z* 194.1 $[M+H]^+$.

***S*-(6-(1,3-dioxoisoindolin-2-yl)hexyl) ethanethioate (5d)**

To a stirred solution of 2-(6-bromohexyl)isoindoline-1,3-dione **5c** (1.0 g, 3.23 mmol, 1.0 equiv.) in DMF (10 mL), was added potassium thioacetate (0.40 g, 3.55 mmol, 1.1 equiv.) at rt under inert atmosphere. The resulting mixture was stirred at rt for 2 hrs. After completion, ice-cold water was added to reaction mixture and stirred for

1 h. Precipitate thus formed was filtered and dried under reduced pressure to afford *S*-(6-(1,3-dioxoisoindolin-2-yl)hexyl) ethanethioate **5d** (0.73 g, 2.39 mmol, 74% yield) as white solid. **^{1}H NMR** (400 MHz, $CDCl_3$) δ 7.89 (d, J = 8.16 Hz, 2H), 7.45 (d, J = 8.08 Hz, 2H), 3.66 (t, J = 7.12 Hz, 2H), 2.84 (t, J = 7.12 Hz, 2H), 2.29 (s, 3H), 1.70 – 1.22 (m, 8H). **MS** (ESI+): *m/z* 306.2 $[M+H]^+$.

6-(1,3-dioxoisoindolin-2-yl)hexane-1-sulfonyl chloride (5e)

To a stirred solution of *S*-(6-(1,3-dioxoisoindolin-2-yl)hexyl)ethanethioate **5d** (1.5 g, 4.92 mmol, 1.0 equiv.) in MeCN (30 mL), was drop-wise added 2M aqueous HCl (3.0 mL) at 0-5°C under inert atmosphere. *N*-chlorosuccinimide (2.8 g, 21.70 mmol, 4.4 equiv.) was then added portion-wise at 0-5°C. Resulting mixture was stirred at rt for 1 hr. After completion, reaction mixture was quenched with ice-cooled water and extracted with diethyl ether. Combined organic layer was washed with saturated aq. $NaHCO_3$, water, brine, dried over anhydrous sodium sulphate, filtered and concentrated under reduced pressure to afford 6-(1,3-dioxoisoindolin-2-yl)hexane-1-sulfonyl chloride **5e** (1.5 g, 4.56 mmol, 92% yield) as white solid. **^{1}H NMR** (400 MHz, DMSO-d_6) δ 7.87 – 7.81 (m, 4H), 3.55 (t, J = 7 Hz, 2H), 2.56 – 2.52 (m, 2H), 1.60 – 1.25 (m, 8H). **MS** (ESI+): *m/z* 330.2 $[M+H]^+$.

6-(1,3-dioxoisoindolin-2-yl)hexane-1-sulfonamide (5f)

A solution of 6-(1,3-dioxoisoindolin-2-yl)hexane-1-sulfonyl chloride **5e** (10 g, 30.39 mmol, 1.0 equiv.) in THF (50 mL) was taken in a sealed tube, cooled to -20°C and NH_3 gas was purged for 0.5 hr. Resulting mixture was stirred at rt for 4 hrs. After completion, reaction mixture concentrated under reduced pressure and diluted with ethyl acetate. Organic layer was washed with water, brine, dried over anhydrous sodium sulphate, filtered and concentrated under reduced pressure. Crude product was purified by column chromatography (silica-gel; 50-70% ethyl acetate - hexane) to afford 6-(1,3-dioxoisoindolin-2-yl)hexane-1-sulfonamide **5f** (6 g, 19.35 mmol, 63% yield) as white solid. **^{1}H NMR** (400 MHz, CD_3OD) δ 7.85 – 7.77 (m, 4H), 3.67 (t, J = 7.04 Hz, 2H), 3.07 – 3.03 (m, 2H), 1.84 – 1.42 (m, 8H). **MS** (ESI+): *m/z* 311 $[M+H]^+$.

6-aminohexane-1-sulfonamide (5g)

To a stirred solution of 6-(1,3-dioxoisoindolin-2-yl)hexane-1-sulfonamide **5f** (6.0 g, 19.35 mmol, 1.0 equiv.) in ethanol (80 mL) and water (3 mL), was added hydrazine monohydrate (1.0 mL, 21.29 mmol, 1.0 equiv.). The resulting mixture was stirred at 80°C for 4 hrs. After completion, the precipitation was filtered and dried under reduced pressure. Filtrate was acidified to pH-4 with 1N aq. HCl, concentrated under reduced pressure and diluted with water. Precipitate formed was filtered. Combined residue was triturated with diethyl ether and pentane to afford 6-aminohexane-1-sulfonamide **5g** (HCl salt**,** 3.4 g, 97% yield) as brown sticky solid. **MS** (ESI+): *m/z* 181.1 $[M+H]^+$.

***Tert*-butyl (6-sulfamoylhexyl)carbamate (5h)**

To a stirred solution of 6-aminohexane-1-sulfonamide **5g** (HCl salt**,** 4.0 g, 22.22 mmol, 1.0 equiv.) in 1,4-dioxane (40 mL) and water (16 mL), were added Et_3N (9.3 mL, 66.66 mmol, 3.0 equiv.) and DMAP (1.3 g, 11.11 mmol, 0.5 equiv.) under inert atmosphere. Boc anhydride (5.61mL, 24.44 mmol, 1.1 equiv.) was then added dropwise. Resulting mixture was stirred at rt for 16 hrs. After completion, reaction mixture was quenched with water and extracted with ethyl acetate. Organic portion was dried over anhydrous sodium sulphate, filtered and concentrated under reduced pressure. Crude mass was purified by combiflash chromatography (silica-gel; 20-50% ethyl acetate - hexane) to afford tert-butyl (6-sulfamoylhexyl)carbamate **5h** (2.6 g, 9.20 mmol, 41% yield) as white solid. **^{1}H NMR** (400 MHz, DMSO-d_6) δ 6.77 – 6.76 (m, 1H), 6.71 (s, 2H), 2.95 – 2.87 (m, 4H), 1.70 – 1.62 (m, 2H), 1.37 – 1.34 (m, 13H), 1.28 – 1.24 (m, 2H). **MS** (ESI+): *m/z* 281.0 [M+H]$^+$.

***Tert*-butyl (6-(*N*-(pent-4-ynoyl)sulfamoyl)hexyl)carbamate (5j)**

To a stirred solution of pent-4-ynoic acid **5i** (0.9 g, 9.18 mmol, 1.0 equiv.) in DMF (15 mL), were added EDCI.HCl (2.6 g, 13.77 mmol, 1.5 equiv.), DIPEA (3.2 mL, 18.36 mmol, 2.0 equiv.) and DMAP (0.12 g, 0.92 mmol, 0.1 equiv.) at rt under inert atmosphere, stirred for 5 min and *tert*-butyl (6-sulfamoylhexyl)carbamate **5h** (2.5 g, 9.18 mmol, 1.0 equiv.) was added. The resulting solution was stirred at rt for 16 hrs. After completion, reaction mixture was quenched with ice-cooled water and extracted with ethyl acetate. The combined organic layer was washed with water, brine, dried over anhydrous sodium sulphate, filtered and concentrated under reduced pressure. Crude product was purified by column chromatography (silica-gel; 50-80% ethyl acetate - hexane) to afford tert-butyl (6-(*N*-(pent-4-ynoyl)sulfamoyl)hexyl)carbamate **5j** (1.8 g, 5.10 mmol, 54% yield) as white solid. **^{1}H NMR** (400 MHz, DMSO-d_6) δ 11.66 (s, 1H), 6.76 (s, 1H), 2.89 – 2.80 (m, 3H), 2.49 – 2.31 (m, 4H), 1.65 – 1.63 (m, 2H), 1.37 -1.36 (m, 15H), 1.25 – 1.23 (m, 2H). **MS** (ESI+): *m/z* 359.0 [M-H]$^+$.

***N*-((6-aminohexyl)sulfonyl)pent-4-ynamide (5k)**

To a stirred solution of tert-butyl (6-(*N*-(pent-4-ynoyl)sulfamoyl)hexyl)carbamate **5j** (1.8 g, 4.98 mmol, 1.0 equiv.) in DCM (20 mL), was added TFA (15 mL, excess) dropwise at 0 °C under argon atmosphere. The resulting mixture was stirred at rt for 3 h. After completion, reaction mixture was concentrated under reduced pressure. Crude mass was triturated with diethyl ether and dried under reduced pressure to afford *N*-((6-aminohexyl)sulfonyl)pent-4-ynamide **5k** (TFA salt, 1.1 g, 4.42 mmol, 89 % yield) as light-yellow gummy liquid. **^{1}H NMR** (400 MHz, DMSO-d_6) δ 11.70 (s, 1H), 7.61 (br s, 2H), 3.38 – 3.34 (m, 2H), 2.83 – 2.82 (m, 1H), 2.79 – 2.73 (m, 2H), 2.47 – 2.45 (m, 2H), 2.40 – 2.37 (m, 2H), 1.67 – 1.28 (m, 8H). **MS** (ESI+): *m/z* 260.8 [M+H]$^+$.

4-(*N*-methyl-*N*-phenylsulfamoyl)benzoic acid (5m)

To a stirred solution of 4-(chlorosulfonyl)benzoic acid **5l** (5.0 g, 22.73 mmol, 1.0 equiv.) in MeOH (100 mL) at 0°C, *N*-methylaniline **5m** (7.3 g, 68.18 mmol, 3.0 equiv.) was added dropwise under inert atmosphere. Resulting mixture was stirred at rt for 16 hrs. After completion, reaction mixture was filtered, solid residue thus obtained

was washed with 10% diethyl ether in pentane and dried under reduced pressure to afford 4-(*N*-methyl-*N*-phenylsulfamoyl) benzoic acid **5n** (3.1 g, 10.65 mmol, 47% yield) as off-white solid. **^{1}H NMR** (400 MHz, DMSO-d_6): δ 13.51 (s, 1H), 8.09 (d, J = 8 Hz, 2H), 7.62 (d, J = 8.4 Hz, 2H), 7.37 – 7.29 (m, 3H), 7.10 (d, J = 8 Hz, 2H), 3.17 (s, 3H). **MS** (ESI+): *m/z* 290.1 $[M+H]^+$

Ethyl 4-((*N*-methyl-4-(*N*-methyl-*N*-phenylsulfamoyl) benzamido) methyl)benzoate (5o)

To a stirred solution of 4-(*N*-methyl-*N*-phenylsulfamoyl) benzoic acid, **5n** (1.0 g, 5.18 mmol, 1.0 equiv.) and ethyl 4-((methylamino)methyl)benzoate **5b** (1.5 g, 5.18 mmol, 1.0 equiv.) in DMF (3 mL) was added NMM (1.4 mL, 12.95 mmol, 2.5 equiv.) followed by the addition of HATU (2.4 g, 6.22 mmol, 1.2 equiv.). The resulting mixture was stirred at rt for 16 hrs. After completion, reaction was quenched with water and extracted with ethyl acetate. Combined organic part was washed with water, brine, dried over anhydrous sodium sulphate, filtered and concentrated under reduced pressure. Crude mass was purified by combiflash chromatography (silica-gel; 30-50% ethyl acetate - hexane) to afford 4-((*N*-methyl-4-(*N*-methyl-*N*-phenylsulfamoyl) benzamido) methyl)benzoate **5o** (1.1 g, 2.36 mmol, 45% yield). **^{1}H NMR** (400 MHz, DMSO-d_6) (VT-NMR) δ 7.96 (d, J = 8 Hz, 2H), 7.61 (s, 4H), 7.42 (t, J = 6.8 Hz, 2H), 7.35 – 7.28 (m, 3H), 7.13 (d, J = 7.2 Hz, 2H), 4.67 (brs, 2H), 4.37 – 4.32 (m, 2H), 3.20 (s, 3H), 2.90 (s, 3H), 1.35 (t, J = 7.2 Hz, 3H). **MS** (ESI+): *m/z* 467.2 $[M+H]^+$.

4-(*N*-methyl-4-(*N*-methyl-*N*-phenylsulfamoyl)benzamido)methyl)benzoic acid (5p)

To a stirred solution of 4-(*N*-methyl-4-(*N*-methyl-*N*-phenylsulfamoyl) benzamido) methyl)benzoate **5p** (0.2 g, 0.43 mmol, 1.0 equiv.) in THF (6 mL) and water (2 mL), was added LiOH (0.09 g, 2.15 mmol, 5.0 equiv.) at rt. The resulting mixture was stirred at rt for 16 hrs. After completion, reaction mixture was concentrated under reduced pressure, diluted with water and washed with ethyl acetate. The aqueous portion part was acidified with 1N HCl and extracted with ethyl acetate. Combined organic portion was dried over anhydrous sodium sulphate, filtered and concentrated under reduced pressure to afford 4-(*N*-methyl-4-(*N*-methyl-*N*-phenylsulfamoyl)benzamido)methyl)benzoic acid **5p** (0.12 g, 0.27 mmol, 86% yield) as off white solid. **^{1}H NMR** (400 MHz, DMSO-d_6) δ 12.94 (s, 1H), 7.96 – 7.94 (m, 2H), 7.68 – 7.08 (m, 11H), 4.76 (s, 1H), 4.49 (s, 1H), 3.17-3.13 (m, 3H), 2.96 (s, 1H), 2.81 (s, 2H). **MS** (ESI+): *m/z* 439.1 $[M+H]^+$.

***N*-methyl-4-(*N*-methyl-*N*-phenylsulfamoyl)-*N*-(4-((6-(*N*-(pent-4-ynoyl) sulfamoyl) hexyl) carbamoyl) benzyl)benzamide (5q)**

To the stirred solution of 4-(*N*-methyl-4-(*N*-methyl-*N*-phenylsulfamoyl)benzamido)methyl)benzoic acid **5p** (0.10 g, 0.23 mmol, 1.20 equiv.) in DMF (1 mL), HATU (0.09 g, 0.23 mmol, 1.2 equiv.) and NMM (0.06 mL, 0.57 mmol, 3.00 equiv.) were added, stirred at rt for 15 min and a solution of N-((6-aminohexyl)sulfonyl)pent-4-ynamide **5k** (0.05 g, 0.92 mmol, 1 equiv.) in DMF (1 mL) was added. The resulting mixture was stirred at rt for 16 hrs. After completion, reaction was quenched with water, extracted with ethyl acetate. Combined organic part was washed with water, brine, dried over anhydrous sodium sulphate, filtered and concentrated under reduced

pressure. Crude product was purified by combiflash chromatography (silica-gel; 50-80% ethyl acetate - hexane) to afford *N*-methyl-4-(*N*-methyl-*N*-phenylsulfamoyl)-*N*-(4-((6-(*N*-(pent-4-ynoyl) sulfamoyl) hexyl) carbamoyl) benzyl)benzamide **5q** (30 mg, 0.044 mmol, 23% yield) as white solid. **^{1}H NMR** (400 MHz, DMSO-d_6) (VT-NMR) δ 11.30 (s, 1H), 8.10 (s, 1H), 7.83 (d, J = 7.6 Hz, 2H), 7.61 (s, 4H), 7.33 – 7.30 (m, 5H), 7.14 (d, J = 7.2 Hz, 2H), 4.64 (br s, 2H), 3.36 – 3.27 (m, 4H), 3.20 (s, 3H), 2.89 (s, 3H), 2.61 (s, 1H), 2.43 – 2.42 (m, 3H), 1.74 – 1.27 (m, 9H). **MS** (ESI+): *m/z* 681.4 $[M+H]^+$.

***N*-(4-((6-(*N*-(cyanomethyl)-*N*-(pent-4-ynoyl)sulfamoyl)hexyl)carbamoyl)benzyl)-*N*-methyl-4-(*N*-methyl-*N*-phenylsulfamoyl)benzamide (5)**

The **5q** (12.0 mg, 0.018 mmol, 1.0 equiv.) was dissolved in 1 mL dry DMF and treated with iodoacetonitrile (26 µL, 0.35 mmol, 20.0 equiv.) and DIPEA (61 µL, 0.35 mmol, 20 equiv.). The mixture was stirred overnight at rt. The desired product was isolated by preparative HPLC (10-100 % acetonitrile in water + 0.1% formic acid).

3.1.6 Compound **6** (EGFR NASA probe)

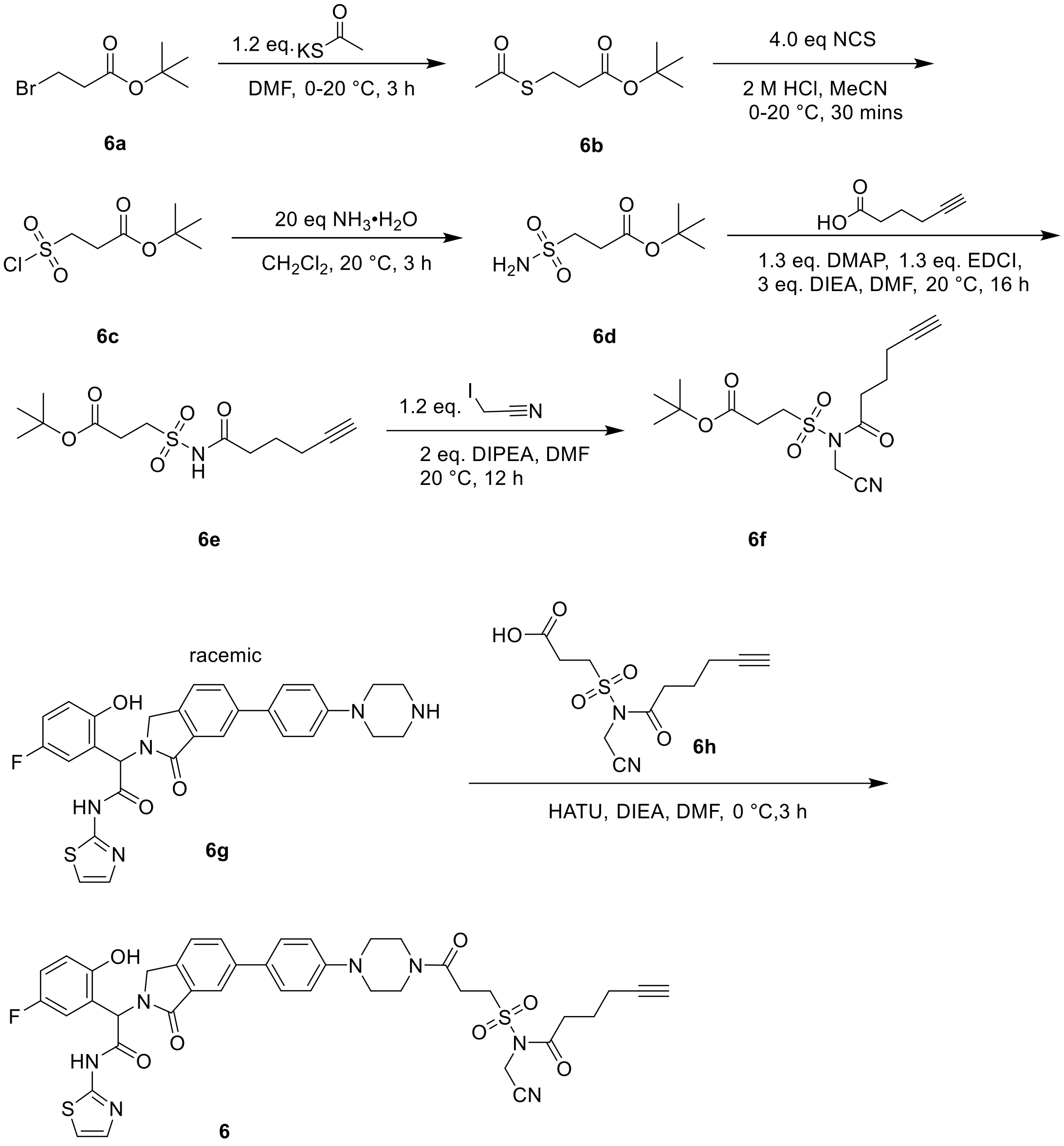

Tert-butyl 3-acetylsulfanylpropanoate **(6b)**

A mixture of tert-butyl 3-bromopropanoate (20.0 mL, 119.57 mmol, 1.0 equiv.), K_2CO_3 (33.0 g, 239.14 mmol, 2.0 equiv.) in DMF (250 mL), then acetylsulfanylpotassium (16.4 g, 143.49 mmol, 1.2 equiv.) was added in portion at 0 °C. The mixture was degassed and purged with N_2 for 3 times, and then the mixture was stirred at 25 °C for 3 h under N_2 atmosphere. TLC (Petroleum ether/Ethyl acetate=10/1) indicated **6a** was consumed completely and one new spot formed. The mixture was diluted with 200 mL of H2O and extracted with ethyl acetate (250 mL × 3). The combined organic layers were washed with brine (200 mL × 6), the combined organic layers were dried over anhydrous sodium sulfate, filtered and the filtrate was concentrated to obtain the crude product *tert*-butyl 3-

acetylsulfanylpropanoate (23.9 g, 117.09 mmol, 98% yield) as a yellow oil. ^{1}**H NMR** (400 MHz, $CDCl_3$) δ 3.08 (t, J = 6.8 Hz, 2H), 2.53 (t, J = 7.2 Hz, 2H), 2.33 (s, 3H), 1.45 (s, 9H).

***Tert*-butyl 3-(chlorosulfonyl)propanoate (6c)**

To a solution of *tert*-butyl 3-acetylsulfanylpropanoate (5.0 g, 24.48 mmol, 1.0 equiv.) in MeCN (50 mL) was added HCl (2 M, 17.1 mL, 1.4 equiv.), NCS (13.1 g, 97.90 mmol, 4.0 equiv.) at 0 °C. The mixture was stirred at 20 °C for 0.5 hr. The TLC (Petroleum ether/Ethyl acetate=1/1) indicated **6b** was consumed completely and one new spot formed. The mixture was diluted with 30 mL of H_2O and extracted with ethyl acetate (150 mL × 3). The combined organic layers were dried over anhydrous sodium sulfate, filtered and the filtrate was concentrated to obtain the crude product *tert*-butyl 3-chlorosulfonylpropanoate (5.0 g, 21.86 mmol, 89% yield) as a white solid. ^{1}**H NMR** (400 MHz, DMSO-d_6) δ 2.71 - 2.68 (m, 2H), 2.47 - 2.42 (m, 2H), 1.38 (s, 9H).

***Tert*-butyl 3-sulfamoylpropanoate (6d)**

To a solution of *tert*-butyl 3-chlorosulfonylpropanoate (5.0 g, 21.86 mmol, 1.0 equiv.) in DCM (20 mL) was added $NH_3.H_2O$ (67.4 mL, 437.27 mmol, 25% purity, 20.0 equiv.). The mixture was stirred at 20 °C for 3 hrs. The TLC (Petroleum ether/Ethyl acetate =1/1) showed no **6c** remained. The reaction mixture was quenched by addition diluted saturated $NaHCO_3$ (50 mL) at 25 °C and then extracted with ethyl acetate (100 mL × 1). The combined organic layers were washed with saturated $NaHCO_3$ (50 mL × 5), the combined organic layers were dried over anhydrous sodium sulfate, filtered and the filtrate was concentrated to dryness to give the crude product *tert*-butyl 3-sulfamoylpropanoate (2.0 g, 9.56 mmol, 44% yield) as a yellow solid. ^{1}**H NMR** (400 MHz, DMSO-d_6) δ 6.85 (s, 2H), 3.18 (t, J = 7.2 Hz, 2H), 2.63 (t, J = 7.2 Hz, 2H), 1.41 (s, 9H).

***Tert*-butyl 3-(hex-5-ynoylsulfamoyl)propanoate (6e)**

To a solution of *tert*-butyl 3-sulfamoylpropanoate (2.0 g, 9.56 mmol, 1.0 equiv.) in DMF (20 mL) were added hex-5-ynoic acid (1.3 mL, 11.47 mmol, 1.2 equiv.), DIPEA (5.0 mL, 28.67 mmol, 3.0 equiv.), DMAP (1.5 g, 12.42 mmol, 1.3 equiv.) and EDCI (2.4 g, 12.42 mmol, 1.3 equiv.). The mixture was stirred at 20 °C for 16 hrs. The TLC (Petroleum ether/Ethyl acetate=1/1) indicated **6d** was consumed completely and one new spot formed. The pH was adjusted to 4 with citric acid (40 mL). The mixture was diluted with 10 mL of H_2O and extracted with ethyl acetate (50 mL × 3). The combined organic layers were washed with brine (30 mL × 3), the combined organic layers were dried over anhydrous sodium sulfate, filtered and the filtrate was concentrated. The residue was purified by column chromatography (SiO_2, Petroleum ether/Ethyl acetate=10/1 to 4/1) to give *tert*-butyl 3-(hex-5-ynoylsulfamoyl)propanoate (2.1 g, 6.92 mmol, 72% yield) as a colorless oil. ^{1}**H NMR** (400 MHz, DMSO-d_6) δ 11.69 (s, 1H), 3.57 (t, J = 6.8 Hz, 2H), 2.80 (t, J = 2.8 Hz, 1H), 2.64 (t, J = 7.0 Hz, 2H), 2.37 (t, J = 7.2 Hz, 2H), 2.17 (dt, J = 2.8, 7.2 Hz, 2H), 1.67 (quin, J = 7.2 Hz, 2H), 1.41 (s, 9H).

***Tert*-butyl 3-[cyanomethyl(hex-5-ynoyl)sulfamoyl]propanoate (6f)**

A mixture of *tert*-butyl 3-(hex-5-ynoylsulfamoyl)propanoate (2.1 g, 6.92 mmol, 1.0 equiv.), 2-iodoacetonitrile (2.3 g, 13.84 mmol, 2.0 equiv.), DIPEA (2.4 mL, 13.84 mmol, 2.0 equiv.) in DMF (20 mL) was degassed and purged with N_2 for 3 times, and then the mixture was stirred at 20 °C for 12 hrs under N_2 atmosphere. LC-MS showed the desired compound was detected. The mixture was diluted with 30 mL of H_2O and extracted with ethyl acetate (50 mL × 3). The combined organic layers were washed with brine (20 mL × 3), the combined organic layers were dried over anhydrous sodium sulfate, filtered and the filtrate was concentrated. The residue was purified by column chromatography (SiO_2, Petroleum ether/Ethyl acetate=8/1 to 4/1) to give *tert*-butyl 3-[cyanomethyl(hex-5-ynoyl)sulfamoyl]propanoate (1.1 g, 2.92 mmol, 42% yield, 99% purity) as a yellow solid. **^{1}H NMR** (400 MHz, DMSO-*d*$_6$) δ 4.73 (s, 2H), 3.86 (t, J = 7.2 Hz, 2H), 2.84 (t, J = 7.2 Hz, 2H), 2.81 (t, J = 2.8 Hz, 1H), 2.75 (t, J = 7.2 Hz, 2H), 2.24 (dt, J = 2.8, 7.2 Hz, 2H), 1.75 (quin, J = 7.2 Hz, 2H), 1.41 (s, 9H). **MS** (ESI+): *m/z* 360 $[M+18]^+$.

***N*-(cyanomethyl)-*N*-((3-(4-(4-(2-(1-(5-fluoro-2-hydroxyphenyl)-2-oxo-2-(thiazol-2-ylamino) ethyl)-3-oxoisoindolin-5-yl) phenyl)piperazin-1-yl)-3-oxopropyl)sulfonyl)hex-5-ynamide (6)**

To a solution of 2-(5-fluoro-2-hydroxy-phenyl)-2-[1-oxo-6-(4-piperazin-1-ylphenyl)isoindolin-2-yl]-*N*-thiazol-2-yl-acetamide (27.0 mg, 0.049 mmol, 1.0 equiv.) and 3-[cyanomethyl(hex-5-ynoyl)sulfamoyl] propanoic acid (21.3 mg, 0.074 mmol, 1.5 equiv.) in DMF (0.5 mL) were added DIPEA (12.8 mg, 0.099 mmol, 2.0 equiv.) and HATU (22.7 mg, 0.060 mmol, 1.3 equiv.) at 0 °C for 3 hrs. LC-MS showed no starting material remained. Several new peaks were shown on LC-MS and the desired compound was detected. The reaction mixture was filtered over celite, and the filtrate was purified by perp-HPLC (column: Phenomenex luna C18 150*25mm* 10um;mobile phase: [water(FA)-ACN];B%: 44%-74%,10min) to give *N*-(cyanomethyl)-*N*-((3-(4-(4-(2-(1-(5-fluoro-2-hydroxyphenyl)-2-oxo-2-(thiazol-2-ylamino) ethyl)-3-oxoisoindolin-5-yl)phenyl)piperazin-1-yl)-3-oxopropyl) sulfonyl) hex-5-ynamide (10.2 mg, 0.012 mmol, 24% yield, 96% purity) as white solid. **^{1}H NMR** (400 MHz, CD_3OD) *δ* 7.99 (d, *J* = 1.2 Hz, 1H), 7.84 (dd, *J* = 2.0, 8.0 Hz, 1H), 7.60 (d, *J* = 8.8 Hz, 2H), 7.55 (d, *J* = 8.0 Hz, 1H), 7.43 (d, *J* = 3.6 Hz, 1H), 7.15 (d, *J* = 3.6 Hz, 1H), 7.09 (d, *J* = 8.8 Hz, 2H), 7.04 (dt, *J* = 3.2, 8.4 Hz, 1H), 6.99 (dd, *J* = 3.2, 8.8 Hz, 1H), 6.90 (dd, *J* = 4.8, 8.8 Hz, 1H), 6.50 (s, 1H), 4.80 (d, *J* = 18.0 Hz, 1H), 4.74 (s, 2H), 4.07 (d, *J* = 17.6 Hz, 1H), 3.92 (t, *J* = 6.8 Hz, 2H), 3.74 (td, *J* = 5.2, 13.2 Hz, 4H), 3.29 - 3.21 (m, 4H), 3.06 (t, *J* = 6.8 Hz, 2H), 2.97 (t, *J* = 7.2 Hz, 2H), 2.35 - 2.26 (m, 3H), 1.91 (t, *J* = 7.2 Hz, 2H). **MS**(ESI+): *m/z* 812.2 $[M+H]^+$.

3.2 Synthesis of the *N*-substituted *N*-acyl sulfonamide library

3.2.1 *N*-(2-oxopropyl)-*N*-(phenylsulfonyl)acetamide (compound **7**)

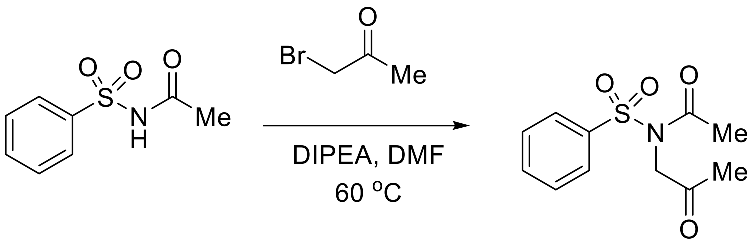

To a solution of *N*-(phenylsulfonyl)acetamide (200 mg, 1.0 mmol, 1.0 equiv.) in dry DMF was added DIPEA (0.52 mL, 3.0 mmol, 3.0 equiv.) at rt and stirred for 1 hr. Then 1-bromopropan-2-one (3.0 mmol, 3.0 equiv.) was added, and the mixture was allowed to stir at 60 °C for 14 hrs. After removal of the solvent, the residue was purified by HPLC to give compound **7** (50% yield). **^{1}H NMR** (400 MHz, $CDCl_3$) δ 8.11 – 7.99 (m, 1H), 7.80 – 7.65 (m, 0H), 7.65 – 7.55 (m, 1H), 4.70 (s, 1H), 2.33 (s, 1H), 2.25 (s, 1H). **^{13}C NMR** (101 MHz, $CDCl_3$) δ 200.6, 169.6, 139.2, 134.1, 129.4, 128.0, 54.5, 26.9, 24.2.

3.2.2 *N*-(1-cyanoethyl)-*N*-(phenylsulfonyl)acetamide (compound **8**)

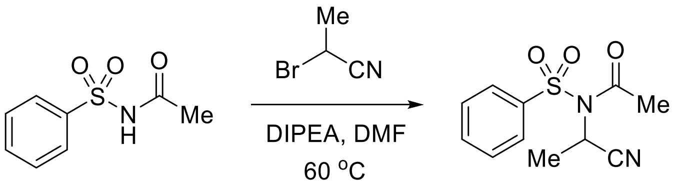

To a solution of *N*-(phenylsulfonyl)acetamide (200 mg, 1.0 mmol, 1.0 equiv.) in dry DMF was added DIPEA (0.52 mL, 3.0 mmol, 3.0 equiv.) at rt and stirred for 1 hr. Then 2-bromopropanenitrile (3.0 mmol, 3.0 equiv.) was added and the mixture was allowed to stir at 60 °C for 14 hrs. After removal of the solvent, the residue was purified by HPLC to give compound **8** (42% yield). **^{1}H NMR** (400 MHz, $CDCl_3$) δ 8.07 – 7.91 (m, 2H), 7.86 – 7.72 (m, 1H), 7.72 – 7.62 (m, 2H), 5.50 (q, *J* = 6.9 Hz, 1H), 2.41 (s, 3H), 1.83 (d, *J* = 7.0 Hz, 3H). **^{13}C NMR** (101 MHz, $CDCl_3$) δ 168.9, 138.7, 134.7, 130.0, 127.2, 117.0, 43.1, 25.3, 19.2.

3.2.3 *N*-(Cyanomethyl)-*N*-(phenylsulfonyl)acetamide (compound **9**)

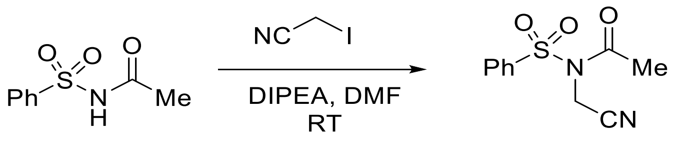

To a solution of *N*-(phenylsulfonyl)acetamide (200 mg, 1.0 mmol, 1.0 equiv.) in dry DMF was added DIPEA (0.52 mL, 3.0 mmol, 3.0 equiv.) at rt and stirred for 1 hr. Then 2-iodoacetonitrile (3.0 mmol, 3.0 equiv.) was added and the mixture was allowed to stir at room temperature for 14 hrs. After removal of the solvent, the residue was purified by HPLC to give compound **9** (32%). **^{1}H NMR** (400 MHz, $CDCl_3$) δ 8.11 – 7.89 (m, 2H), 7.86 – 7.71 (m, 1H), 7.71 – 7.60 (m, 2H), 4.80 (s, 2H), 2.43 (s, 3H). **^{13}C NMR** (101 MHz, $CDCl_3$) δ 169.0, 138.1, 135.0, 130.0, 127.6, 114.6, 32.9, 24.3.

3.2.4 *N*-(2-amino-2-oxoethyl)-*N*-(phenylsulfonyl)acetamide (compound **10**)

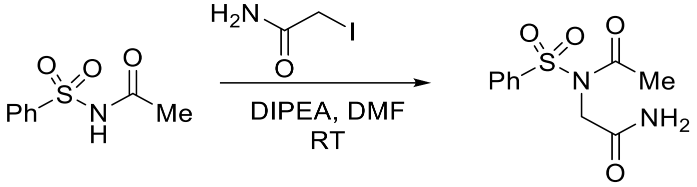

To a solution of *N*-(phenylsulfonyl)acetamide (20 mg, 0.1 mmol, 1.0 equiv.) in dry DMF was added DIPEA (0.07 mL, 0.4 mmol, 4 equiv.) at rt and stirred for 1 hr. Then 2-iodoacetamide (37 mg, 0.2 mmol, 2.0 equiv.) was added and the mixture was allowed to stir at room temperature for 14 hrs. After removal of the solvent, the residue was purified by flash chromatography (0-20% MeOH in DCM), followed by another purification by HPLC to give compound **10**. **MS**(ESI+): *m/z* 279.2 $[M+Na]^+$. (ESI-): *m/z* 255.2 $[M-H]^-$.

3.2.5 *N*-(Phenylsulfonyl)-*N*-(2,2,2-trifluoroethyl)acetamide (compound **11**)

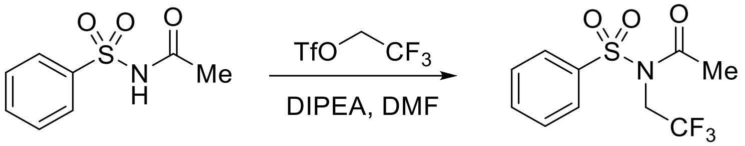

To a solution of *N*-(phenylsulfonyl)acetamide (200 mg, 1.0 mmol, 1.0 equiv.) in dry DMF was added DIPEA (0.52 mL, 3.0 mmol, 3.0 equiv.) at rt and stirred for 1 hr. Then 2,2,2-trifluoroethyl trifluoromethanesulfonate (3.0 mmol, 3.0 equiv.) was added and the mixture was allowed to stir at room temperature for 14 hrs. After removal of the solvent, the residue was purified by HPLC to give compound **11** (30% yield). ^{1}**H NMR** (400 MHz, $CDCl_3$) δ 8.09 – 7.84 (m, 2H), 7.78 – 7.65 (m, 1H), 7.64 – 7.53 (m, 2H), 4.62 (q, *J* = 8.3 Hz, 2H), 2.34 (s, 3H). ^{13}**C NMR** (101 MHz, CDCl3) δ 169.5, 139.0, 134.4, 129.5, 127.9, 123.2 (q, *J* = 280 Hz), 45.4 (q, *J* = 36.3 Hz), 24.4. ^{19}**F NMR** (376 MHz, $CDCl_3$) δ -69.26.

3.2.6 *N*-(2,2,3,3,3-pentafluoropropyl)-*N*-(phenylsulfonyl)acetamide (compound **12**)

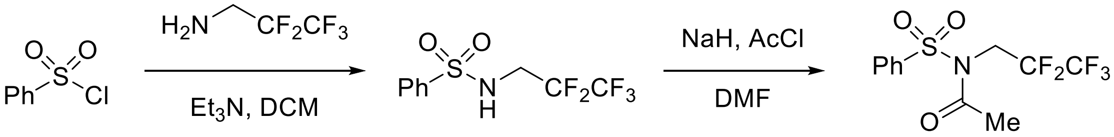

To a solution of 2,2,3,3,3-pentafluoropropan-1-amine (200 mg, 1.0 mmol, 1.0 equiv) in CH_2Cl_2 at 0 °C was added dropwise Et_3N (0.28 mL, 2.5 mmol, 2.5 equiv.) and benzenesulfonyl chloride (212 mg, 1.2 mmol, 1.2 equiv.). The mixture was stirred at rt overnight. The reaction mixture was concentrated under reduced pressure and purified by flash column chromatography to yield intermediate sulfonamide in 75% yield. To a solution of sulfonamide (0.5 mmol, 1.0 equiv.) in dry DMF was added NaH (2.0 equiv.) at 0 °C and stirred for 1 hr. Then acetyl chloride (0.071 mL, 1.0 mmol, 2 equiv.) was added, and the mixture was allowed to stir at room temperature for 14 hrs. After removal of the solvent, the residue was purified by HPLC to give compound **12** in 66% yield. ^{1}**H NMR** (400 MHz, $CDCl_3$) δ 7.97 – 7.90 (m, 2H), 7.75 – 7.66 (m, 1H), 7.60 (dd, *J* = 8.5, 7.2 Hz, 2H), 4.66 (t, *J* = 14.8 Hz, 2H),

2.34 (s, 3H). **^{13}C NMR** (101 MHz, $CDCl_3$) δ 169.8, 139.2, 134.6, 129.7, 128.0, 120.1, 114.9, 112.7, 112.3, 43.8, 43.5, 43.3, 24.6. **^{19}F NMR** (376 MHz, $CDCl_3$) δ -84.49, -118.77.

3.2.7 *N*-(benzylsulfonyl)-*N*-(2,2,2-trifluoroethyl)acetamide (compound **13**)

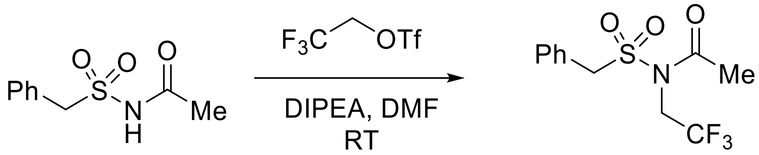

To a solution of *N*-(benzylsulfonyl)acetamide (252 mg, 1.0 mmol, 1.0 equiv.) in dry DMF was added DIPEA (0.52 mL, 3.0 mmol, 3.0 equiv.) at rt and stirred for 1 hr. Then 2,2,2-trifluoroethyl trifluoromethanesulfonate (3.0 mmol, 3.0 equiv.) was added and the mixture was allowed to stir at room temperature for 14 hrs. After removal of the solvent, the residue was purified by HPLC to give compound **13** (40% yield). **^{1}H NMR** (400 MHz, $CDCl_3$) δ 7.57 – 7.33 (m, 5H), 4.66 (s, 2H), 4.25 (q, *J* = 8.5 Hz, 2H), 2.16 (s, 3H). **^{13}C NMR** (101 MHz, $CDCl_3$) δ 170.4, 131.0, 129.9, 129.3, 126.7, 123.4 (q, *J* = 285.8 Hz), 60.9, 46.0 (q, *J* = 35.8 Hz), 23.87. **^{19}F NMR** (376 MHz, CDCl3) δ -69.47.

3.2.8 *N*-(phenethylsulfonyl)-*N*-(2,2,2-trifluoroethyl)acetamide (compound **14**)

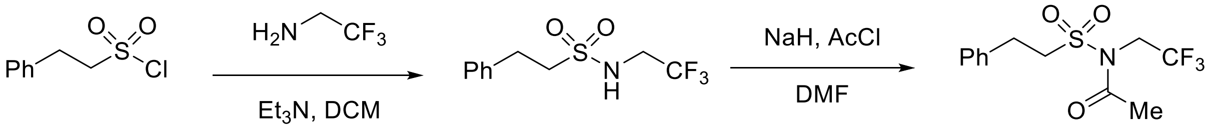

To a solution of 2,2,2-trifluoroethan-1-amine (0.078 mL, 1.0 mmol, 1.0 equiv) in CH_2Cl_2 at 0 °C was added dropwise Et_3N (0.28 mL, 2.5 mmol, 2.5 equiv) and 2-phenylethane-1-sulfonyl chloride (1.2 equiv.). The mixture was stirred at rt overnight. The reaction mixture was concentrated under reduced pressure and purified by flash column chromatography to yield 2-phenyl-*N*-(2,2,2-trifluoroethyl)ethane-1-sulfonamide in 70% yield.

To a solution of sulfonamide (134 mg, 0.5 mmol, 1.0 equiv.) in dry DMF was added NaH (2.0 equiv.) at 0 °C and stirred for 1 hr. Then acetyl chloride (0.071 mL, 1.0 mmol, 2.0 equiv.) was added, and the mixture was allowed to stir at room temperature for 14 hrs. After removal of the solvent, the residue was purified by HPLC to give compound **14** (30% yield). **^{1}H NMR** (400 MHz, $CDCl_3$) δ 7.42 – 7.18 (m, 5H), 4.39 (q, *J* = 8.4 Hz, 2H), 3.85 – 3.66 (m, 2H), 3.32 – 3.10 (m, 2H), 2.40 (s, 3H). **^{13}C NMR** (101 MHz, $CDCl_3$) δ 170.1, 136.6, 129.0, 128.4, 127.4, 123.2 (q, *J* = 276.2 Hz), 56.4, 45.6 (q, *J* = 35.6 Hz), 29.2, 24.1. **^{19}F NMR** (376 MHz, $CDCl_3$) δ -69.80.

3.2.9 Methyl *N*-acetyl-*N*-(phenylsulfonyl)glycinate (**4S1**)

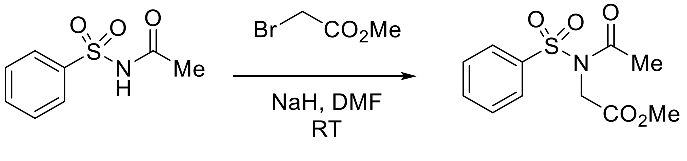

To a solution of *N*-(phenylsulfonyl)acetamide (200 mg, 1.0 mmol, 1.0 equiv.) in dry DMF was added NaH (2 equiv.) at 0 °C and stirred for 1 hr. Then methyl 2-bromoacetate (0.19 mL, 2.0 mmol, 2.0 equiv.) was added and the mixture was allowed to stir at room temperature for 14 hrs. After removal of the solvent, the residue was purified by HPLC to give compound **4S1** (35% yield). **^{1}H NMR** (400 MHz, $CDCl_3$) δ 8.13 – 7.98 (m, 2H), 7.75 – 7.66 (m, 1H), 7.61 (ddd, *J* = 8.7, 5.2, 1.8 Hz, 2H), 4.64 (d, *J* = 2.3 Hz, 2H), 3.78 (d, *J* = 3.3 Hz, 3H), 2.34 (d, *J* = 3.0 Hz, 3H). **^{13}C NMR** (101 MHz, $CDCl_3$) δ 169.7, 168.5, 139.1, 134.2, 129.4, 128.0, 52.6, 46.7, 24.4.

3.2.10 *N*-(2,2,3,3,3-pentafluoropropyl)-*N*-(phenethylsulfonyl)acetamide (**4S3**)

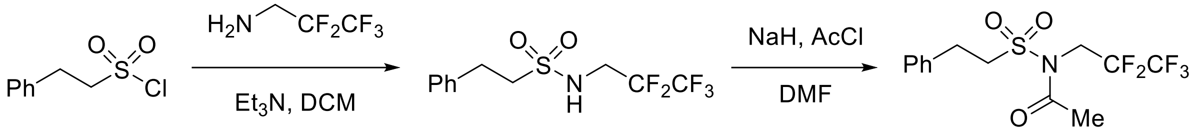

To a solution of 2,2,3,3,3-pentafluoropropan-1-amine (149 mg, 1.0 mmol, 1.0 equiv.) in CH_2Cl_2 at 0 °C was added dropwise Et_3N (0.28 mL, 2.5 mmol, 2.5 equiv) and 2-phenylethane-1-sulfonyl chloride (246 mg, 1.2 mmol, 1.2 equiv.). The mixture was stirred at rt overnight. The reaction mixture was concentrated under reduced pressure and purified by flash column chromatography to yield intermediate sulfonamide in 60% yield. To a solution of sulfonamide (159 mg, 0.5 mmol, 1.0 equiv.) in dry DMF was added NaH (2.0 equiv.) at 0 °C and stirred for 1 hr. Then acetyl chloride (0.071 mL, 1.0 mmol, 2.0 equiv.) was added, and the mixture was allowed to stir at room temperature for 14 hrs. After removal of the solvent, the residue was purified by HPLC to give compound **4S3** (50% yield). **^{1}H NMR** (400 MHz, $CDCl_3$) δ 7.48 – 7.13 (m, 5H), 4.45 (t, *J* = 15.2 Hz, 2H), 3.91 – 3.65 (m, 2H), 3.36 – 3.14 (m, 2H), 2.43 (s, 3H). **^{13}C NMR** (101 MHz, $CDCl_3$) δ 170.1, 136.6, 129.1, 128.4, 127.4, 118.2 (tq, *J* = 273.0, 38.0 Hz), 112.2 (tq, *J* = 265.2, 35.0 Hz), 56.5, 43.5 (t, *J* = 22.0 Hz) 29.2, 24.1. **^{19}F NMR** (376 MHz, $CDCl_3$) δ -84.42, -118.94.

3.3 Synthesis of the SuFA linker S15

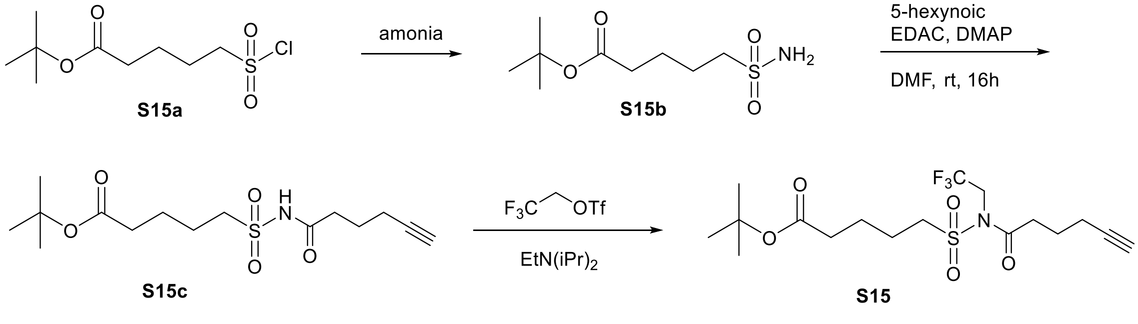

***Tert*-butyl 5-sulfamoylpentanoate (S15b)**

The sulfonyl chloride **S15a** was synthesized using a reported method.[9]

To make the intermediate **S15b**, a solution of **S15a** (200 mg, 0.78 mmol, 1.0 equiv.) in dry dioxane was added dropwise to a solution of ammonia 0.5 M in dioxane (7.8 mL, 3.90 mmol, 5.0 equiv.) at room temperature. The mixture was stirred for 2 hrs at room temperature. Solvent was removed under vacuum and the crude reaction was used for the next step.

***Tert*-butyl 5-(*N*-(hex-5-ynoyl)sulfamoyl)pentanoate (S15c)**

To a solution of sulfonamide **S15b** (100 mg, 0.41 mmol, 1.0 equiv.), DMAP (67 mg, 0.55 mmol, 1.3 equiv.), DIPEA (0.22 mL, 1.26 mmol, 3,0 equiv.) and 5-hexynoic acid in dry DMF, was added EDAC·HCl (121 mg, 0.63 mmol, 1.5 equiv.). The mixture was stirred for 2 hrs art room temperature, and the product was isolated by flash chromatography using C18 column, gradient 10-100% ACN in water (+0.1% formic). Obtained 42 mg (30% yield). **^{1}H NMR** (400 MHz, DMSO) δ 11.59 (s, 1H), 3.33 (d, *J* = 8.0 Hz, 2H), 2.80 (t, *J* = 2.6 Hz, 1H), 2.36 (t, *J* = 7.4 Hz, 2H), 2.28 – 2.13 (m, 4H), 1.73 – 1.52 (m, 6H), 1.39 (s, 9H). **^{13}C NMR** (101 MHz, DMSO) δ 172.2, 171.8, 83.6, 79.6, 71.8, 51.6, 34.2, 34.1, 27.7, 23.0, 22.9, 22.3, 17.1. ESI- 330.2 $(M-H)^{-}$.

***Tert*-butyl 5-(N-(hex-5-ynoyl)-N-(2,2,2-trifluoroethyl)sulfamoyl)pentanoate (S15, SuFA linker precursor)**

To a solution of **int S15c** (80 mg, 0.24 mmol, 1.0 equiv.) in 2 mL dry DMF, were added $TfOCH_2CF_3$ (0.17 mL, 1.2 mmol, 5.0 equiv.) and DIPEA (0.25 mL, 1.45 mmol, 6.0 equiv.). The mixture was stirred for 12 hrs at room temperature and monitored by LC-MS. The reaction was stopped by adding trifluoracetic acid at 0 °C and the product was purified by prep HPLC (10-100% ACN in water + 0.1% formic). Obtained 62 mg of a pale-yellow oil (62% yield). **^{1}H NMR** (400 MHz, $CDCl_3$) δ 4.50 (q, *J* = 8.5 Hz, 2H), 3.54 – 3.46 (m, 2H), 2.86 (t, *J* = 7.0 Hz, 2H), 2.35 – 2.24 (m, 4H), 2.02 (t, *J* = 2.6 Hz, 1H), 1.94 (q, *J* = 7.0 Hz, 4H), 1.75 (p, *J* = 7.4 Hz, 2H), 1.44 (s, 9H). **^{13}C NMR** (101 MHz, $CDCl_3$) δ 172.7, 172.1, 127.8, 125.0, 122.2, 119.4, 83.1, 80.9, 70.0, 55.5, 45.7, 45.4, 45.0, 44.7, 34.7, 34.5, 28.2, 23.6, 23.3, 22.5, 17.5. **^{19}F NMR** (376 MHz, $CDCl_3$) δ -69.76. **MS** (ESI+): *m/z* 436.2 $(M+Na)^{+}$.

3.4 Synthesis of compound 17 (FPR SuFA probe)

Synthesis of precursor 16 (FPR2 peptide) on solid phase

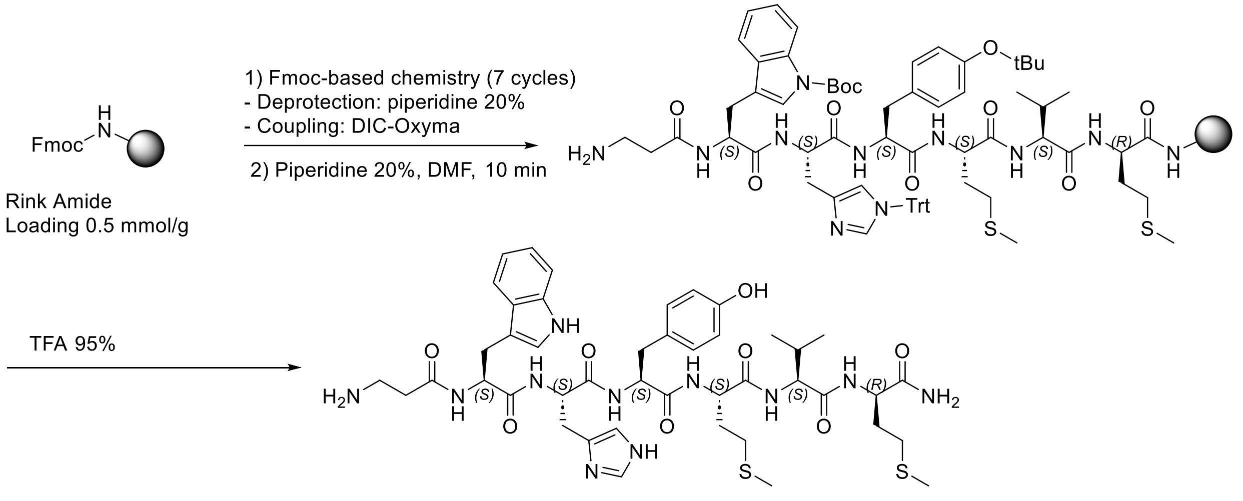

Compound **16**

The precursor peptide was synthesized at 100 µmol scale on Rink Amide (200 mg, loading 0.5 mmol/g) using microwave peptide synthesizer Liberty Blue 2.0 (CEM, Matthews, NC). In short, the Fmoc protecting group was removed with piperidine 20% in DMF (75 °C, 15s; followed by 90 °C, 50s) while peptide coupling was carried out with amino acid (5 equiv.), DIC (5 equiv.), oxyma (5 equiv.) (75°C, 15s; followed by 90°C, 110s) in DMF. Resin was washed with DMF between coupling and deprotection cycle or after every other reaction. Seven deprotection-coupling cycles were employed to build the precursor peptide Fmoc-(β-Ala)-Trp(Boc)-His(Trt)-Tyr(tBu)-Met-Val-(*D*-Met)-Rink Amide. When the last amino acid was coupled, the Fmoc was deprotected by piperidine 20% and washed thoroughly with DMF, followed by DCM. The peptide was cleaved with TFA-water-TIPS (95:2.5:2.5) for 2 h (4 mL for 200 mg resin). The liquid was collected by filtration and concentrated under nitrogen flow until < 1 mL. The peptide was precipitated in 8 mL cold ether. The solid was isolated by centrifugation (2000 rpm, 10 min at 4°C), dried for 5 min under nitrogen flow before redissolved in DMSO for purification. The product was purified by preparative HPLC using gradient 10-35% ACN in water (+0.1% formic) for 30 min on XBridge Prep C18 5µm 19 × 250mm OBD column (Waters, Milford, MA) with AccQPrep HP150 system (Teledyne, Thousand Oaks, CA). Pure fractions were combined, following by adding 0.1 mL trifluoroacetic acid before lyophilization. Obtained 14 mg of final product. **^{1}H NMR** (400 MHz, DMSO-d_6) δ 4.23 (s, 1H), 14.12 (s, 1H), 10.80 (s, 1H), 8.96 (s, 1H), 8.50 – 8.38 (m, 2H), 8.31 (d, *J* = 7.6 Hz, 1H), 8.23 (d, *J* = 8.2 Hz, 1H), 8.00 (d, *J* = 7.4 Hz, 1H), 7.94 (d, *J* = 7.9 Hz, 1H), 7.68 (s, 3H), 7.59 (d, *J* = 7.9 Hz, 1H), 7.38 – 7.26 (m, 3H), 7.11 (t, *J* = 3.1 Hz, 2H), 7.07 – 6.98 (m, 3H), 6.92 (t, *J* = 7.5 Hz, 1H), 6.63 (d, *J* = 8.1 Hz, 2H), 4.61 (q, *J* = 7.4 Hz, 1H), 4.54 – 4.38 (m, 3H), 4.29 (td, *J* = 8.9, 4.2 Hz, 1H), 4.13 (t, *J* = 7.5 Hz, 1H), 3.13 – 3.02 (m, 2H), 3.00 – 2.83 (m, 5H), 2.73 (dd, *J* = 14.0, 8.7 Hz, 1H), 2.47 – 2.33 (m, 6H), 2.02 (s, 6H), 1.95 (t, *J* = 6.8 Hz, 3H), 1.85 – 1.71 (m, 2H), 0.86 (d, *J* = 6.6 Hz, 6H). **^{13}C NMR** (101 MHz, DMSO-d_6) δ 173.2, 171.7, 171.2, 171.1, 170.9, 169.7, 169.5, 161.8, 155.9, 136.0, 133.7, 130.2, 129.2, 127.3, 127.2, 123.7, 120.9, 118.5, 118.2, 116.9, 114.9, 111.3, 109.9,

58.4, 55.9, 54.2, 53.6, 51.9, 51.4, 36.5, 35.2, 32.0, 31.9, 31.3, 30.2, 29.7, 29.4, 27.6, 19.1, 18.3, 14.7, 14.5. **^{19}F NMR** (376 MHz, DMSO-d_6) δ -73.65 (TFA salt). **MS** (ESI+): *m/z* 936.26 (M+H)$^+$.

Synthesis of compound 17 (FPR SuFA probe)

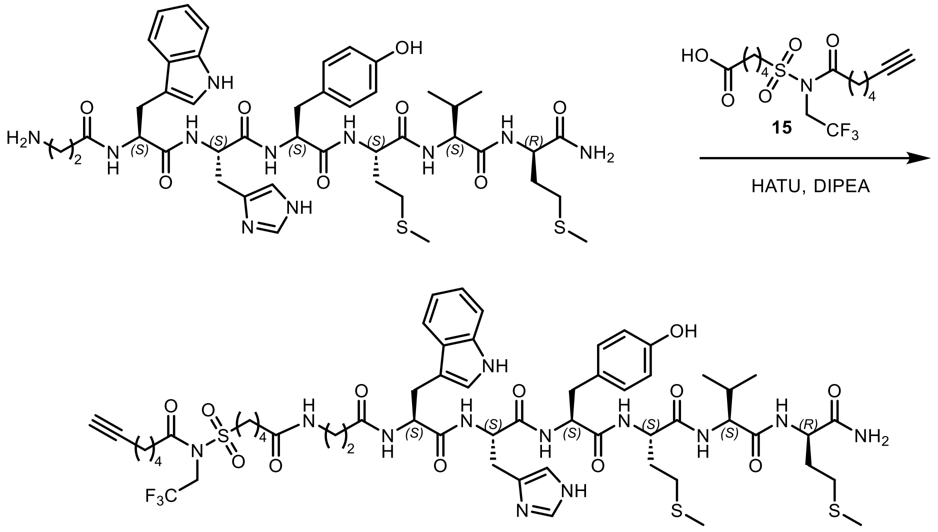

Tert-butyl ester of SuFA linker (**S15**, 6.56 mg, 0.016 mmol, 1.5 equiv.) was weighted to a 4 mL vial and diluted with 1 mL of DCM before adding 1 mL TFA. The mixture was stirred at rt for 30 min before transferred to a 50 mL RBF. Solvent was removed under vacuum and the crude was co-evaporated 3 times with toluene (40 mbar) to yield linker **15**. To activate the carboxylic acid of SuFA linker **15**, HATU (6.0 mg, 0.016 mmol, 1.5 equiv.) was added, followed by 1.5 mL of dried DMF. The DIPEA (18 µL, 0.11 mmol, 10 equiv.) was added to the mixture and stirred for 10 min. To this mixture, a solution of FPR2 ligand **16** (9.9 mg, 0.011 mmol, 1.0 equiv.) in 0.5 mL DMF was added slowly. The reaction was run for 1 hr (crude LC-MS at 45 min below) before stopping by adding 20 µL TFA at 0 °C.

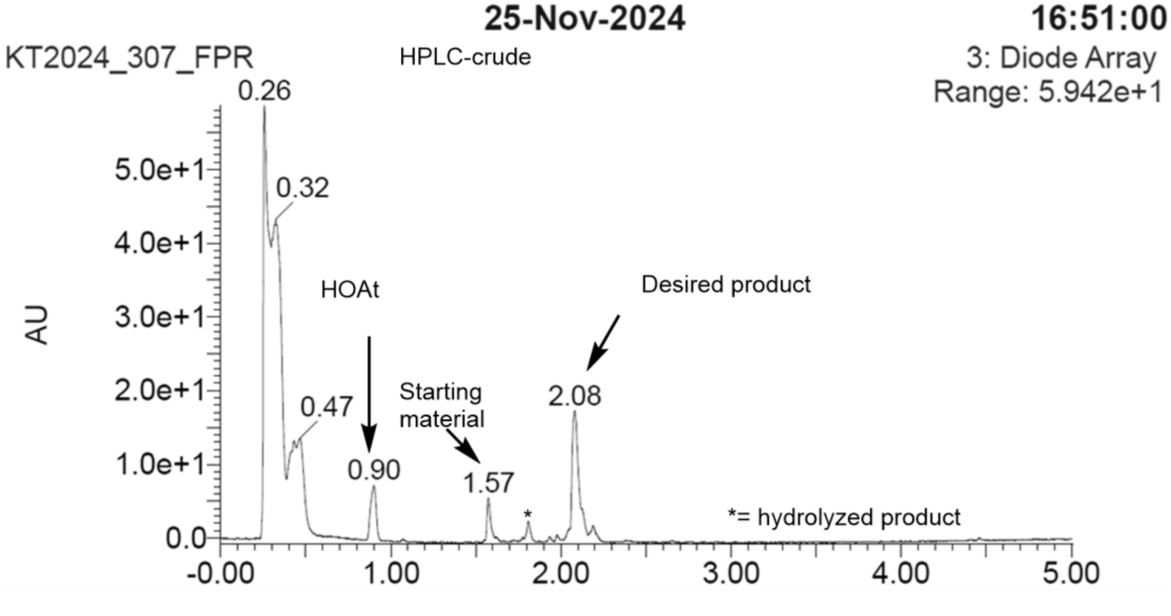

The crude mixture was filtered, and the product was isolated by preparative HPLC using gradient 10-35% ACN in water (+0.1% formic) for 30 min on XBridge Prep C18 5μm 19x250mm OBD column (Waters, Milford, MA) with AccQPrep HP150 system (Teledyne, Thousand Oaks, CA). Obtained 7.1 mg (53% yield). The peptide purity was assessed using Acquity UPLC-MS system (Waters, Milford, MA), equipped with BEH C18 1.7μm 2.1x50mm column. The gradient used ACN and water with 0.1% HCOOH (0 → 0.25 min, 5% ACN; 0.25 → 4.25 min, 5% → 95%; 4.25 → 4.75 min, 95%; 4.75 → 5.0 min, 95% → 5%). Peptides display purity 95% before used for testing. **^{1}H NMR** (400 MHz, DMSO-d_6) δ 14.09 (s, 1H), 14.01 (s, 1H), 10.76 (s, 1H), 9.17 (s, 1H), 8.96 (s, 1H), 8.38 (d, *J* = 7.9 Hz, 1H), 8.35 (d, *J* = 8.0 Hz, 1H), 8.23 (d, *J* = 8.3 Hz, 1H), 8.11 (d, *J* = 7.5 Hz, 1H), 7.99 (d, *J* = 7.5 Hz, 1H), 7.94 (d, *J* = 7.9 Hz, 1H), 7.74 (t, *J* = 5.8 Hz, 1H), 7.58 (d, *J* = 7.9 Hz, 1H), 7.31 (d, *J* = 7.9 Hz, 3H), 7.10 (d, *J* = 2.7 Hz, 2H), 7.03 (d, *J* = 7.8 Hz, 3H), 6.92 (t, *J* = 7.5 Hz, 1H), 6.63 (d, *J* = 8.0 Hz, 2H), 4.58 (dd, *J* = 18.0, 8.4 Hz, 3H), 4.47 (dt, *J* = 20.8, 7.0 Hz, 3H), 4.28 (td, *J* = 9.0, 4.4 Hz, 1H), 4.12 (t, *J* = 7.5 Hz, 1H), 3.61 (t, *J* = 7.6 Hz, 2H), 3.20 – 3.02 (m, 4H), 2.99 – 2.86 (m, 3H), 2.87 – 2.79 (m, 3H), 2.73 (dd, *J* = 14.1, 8.9 Hz, 1H), 2.47 – 2.34 (m, 4H), 2.21 (dq, *J* = 10.6, 5.1 Hz, 4H), 2.05 (d, *J* = 7.2 Hz, 2H), 2.02 (s, 6H), 1.95 (p, *J* = 6.4 Hz, 3H), 1.77 (dq, *J* = 24.8, 6.3 Hz, 4H), 1.66 (q, *J* = 7.9 Hz, 2H), 1.57 (q, *J* = 7.3 Hz, 2H), 0.86 (dd, *J* = 6.7, 2.4 Hz, 6H). **^{13}C NMR** (101 MHz, DMSO-d_6) δ 173.2, 173.0, 172.0, 171.5, 171.2, 171.1, 170.9, 170.7, 169.7, 155.9, 136.0, 133.6, 130.2, 129.1, 128.9, 127.3, 127.2, 125.2, 123.6, 120.7, 118.5, 118.2, 114.9, 111.2, 110.1, 83.6, 71.9, 58.4, 53.7, 51.4, 40.1, 39.9, 39.7, 39.5, 39.3, 39.1, 38.9, 35.2, 34.4, 33.7, 31.3, 30.2, 29.7, 29.4, 23.5, 23.2, 22.1, 19.1, 18.3, 16.8, 14.7, 14.5. **^{19}F NMR** (376 MHz, DMSO-d_6) δ -67.93, -74.16 (TFA salt). The integrity of the SuFA linker was indicated by HMBC signal between the C$\underline{H_2}$$CF_3$ and the carbonyl carbon ($\underline{C}$=O) on the alkyne handle (please see NMR spectra section). ESI+ 1274.13 $(M+H)^+$. **HRMS** (ESI+): *m/z* calcd for $C_{57}H_{78}F_3N_{12}O_{12}S_3$: 1275.4971 $[M+H]^+$; found: 1275.4971.

3.5 Synthesis of compound 19 (GLP1R SuFA probe)

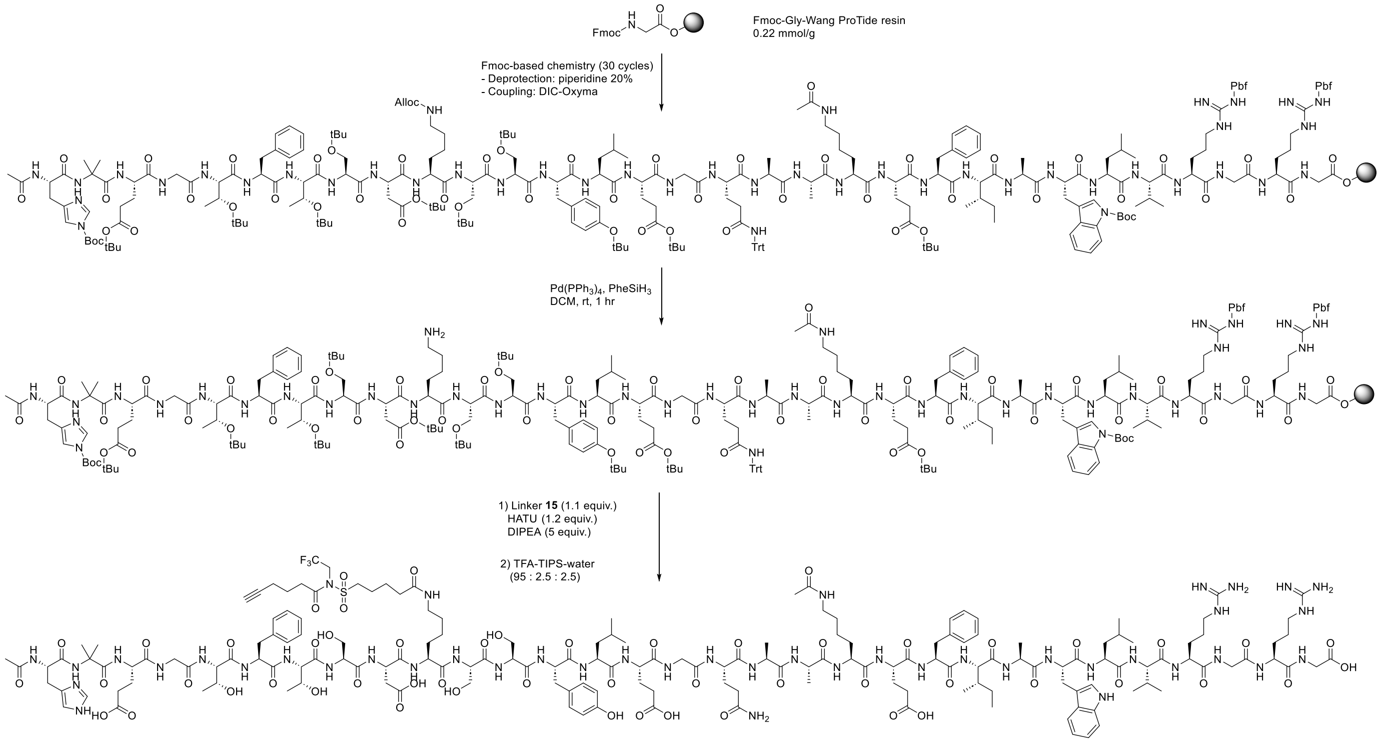

The precursor peptide was synthesized at 100 µmol scale on Fmoc-Gly-Wang ProTide resin (300 mg, loading 0.3 mmol/g) using microwave peptide synthesizer Liberty Blue 2.0 (CEM, Matthews, NC). In short, the Fmoc protecting group was removed with piperidine 20% in DMF (90 °C, 15 s; followed by 110 °C, 80 s) while peptide coupling was carried out with amino acid (5.0 equiv.), DIC (5.0 equiv.), oxyma (5.0 equiv.) (90 °C, 15 s; followed by 110 °C, 60 s) in DMF. Resin was washed with DMF between coupling and deprotection cycle or after every other reaction. Thirty cycles of deprotection/coupling were used to build the precursor peptide. When the last amino acid was coupled, the Fmoc was deprotected by piperidine 20% and the N-terminus was capped with acetic anhydride (47 µL, 0.5 mmol, 5.0 equiv.).

Alloc deprotection: In a round bottom flash, the resin was suspended in 5 mL DCM and nitrogen was bubbled to the mixture for 5 min. The phenyl silane (370 µL, 3.0 mmol, 30 equiv.) was added, followed by tetrakis triphenylphosphine (29 mg, 0.035 mmol, 0.25 equiv.). The mixture was stirred for 1hr. Excess of reagent was removed by filtration and resin was washed twice with DMF, twice with 1% sodium diethyl dithiocarbamate in DMF.

SuFA linker coupling: Resin was washed 3 times with NMP, twice with solution of 2.0 equiv. of HOBt in NMP (5 min each, to remove piperidine traces), followed by 3 times NMP to clean all the reagent before SuFA linker coupling. In a round bottom flash, the linker **15** (prepared from its tert-butyl ester as previously described in SI section 3.4) was activated by treating with two equivalents of HATU (47 mg, 0.12 mmol), followed by 3 mL of dried DMF, and DIPEA (0.052 mL, 0.31 mmol, 5.0 equiv.). Resin was added in small portion into the activated SuFA linker solution and stirred at rt for 40 min. The excess reagent was removed by filtration and the resin was washed thrice with DCM.

The peptide was cleaved with TFA-water-TIPS (95:2.5:2.5) for 2 hrs (5 mL for 300 mg resin). The liquid was collected by filtration and concentrated under nitrogen flow until < 1 mL. The peptide was precipitated in 8 mL cold ether. The solid was isolated by centrifugation (2000 rpm, 10 min at 4°C), dried for 5 min under nitrogen flow before redissolved in DMSO for purification. The product was purified by preparative HPLC using gradient 10-35% ACN in water (+0.1% formic) for 30 min on XBridge Prep C18 5µm 19x250mm OBD column (Waters, Milford, MA) with AccQPrep HP150 system (Teledyne, Thousand Oaks, CA). Pure fraction was combined and lyophilized to afford 16.9 mg product as white powder. The peptide purity was assessed using Acquity UPLC-MS system (Waters, Milford, MA), equipped with BEH C18 1.7µm 2.1x50mm column. The gradient used ACN and water with 0.1% HCOOH (0 → 0.25 min, 5% ACN; 0.25 → 4.25 min, 5% → 95%; 4.25 → 4.75 min, 95%; 4.75 → 5.0 min, 95% → 5%). Peptides display purity 95% before used for testing. **HRMS** (ESI+): *m/z* calcd for $C_{170}H_{254}F_3N_{44}O_{53}S$: 1283.6115 $[M+3H]^{3+}$; found: 1283.6110.

LC-MS crude of semaglutide analogs was provided after the following steps:

- Synthesis of linear peptide (upper pannel)
- Alloc deprotection (middle pannel)
- SuFA linker coupling (bottom pannel)

As the figure below shows that majority of impurities appeared during the Alloc deprotection steps.

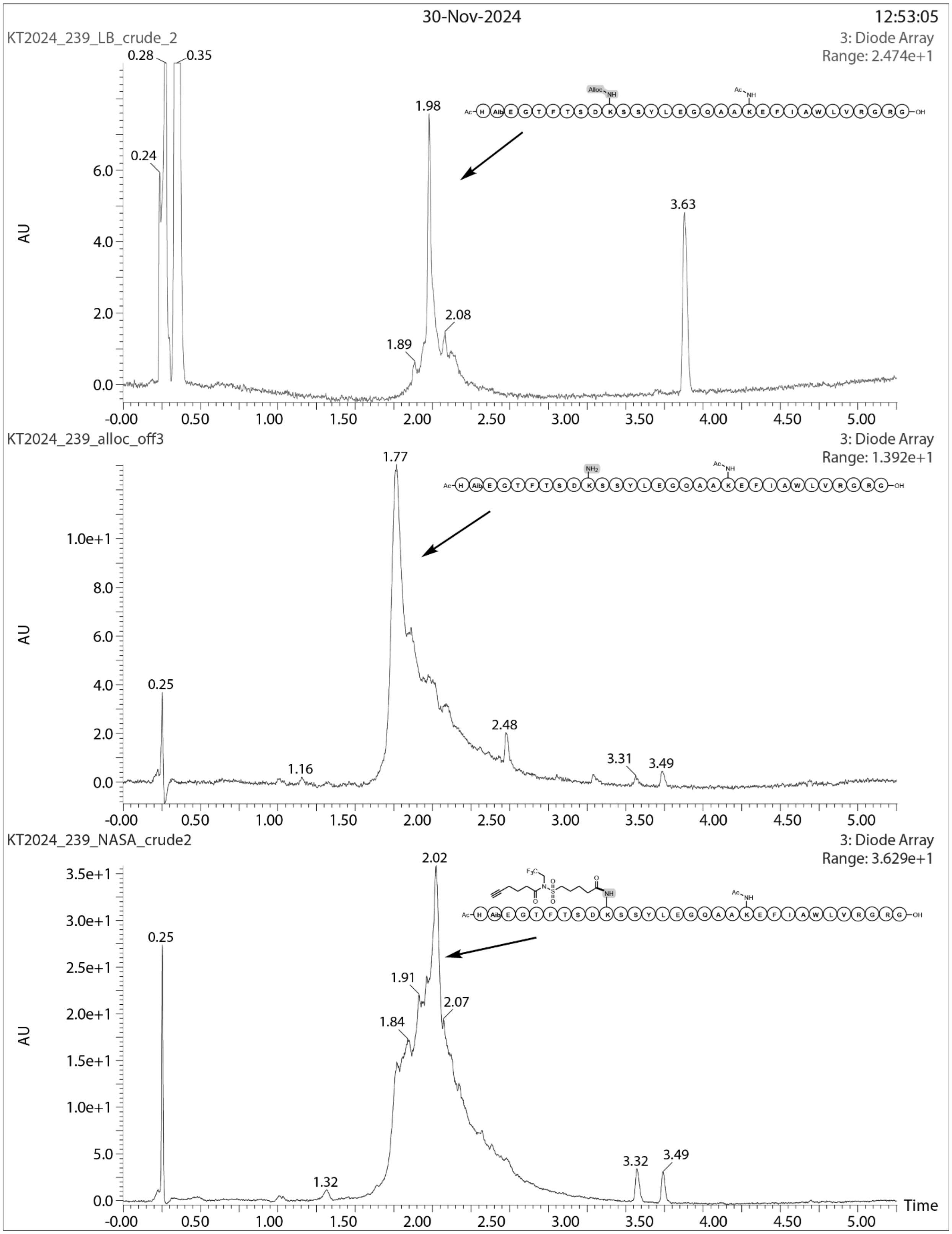

Synthesis of **S4** and **S5** were similar to the synthesis of GLP1R probe **19**.

3.6 Synthesis of compound 21 (PSMA SuFA probe)

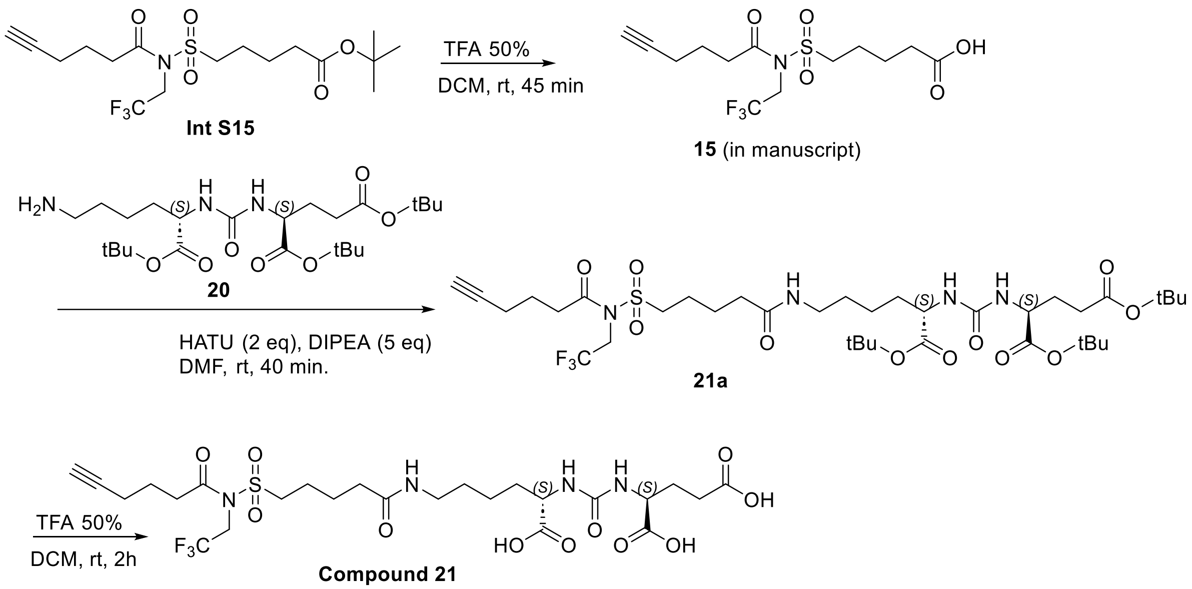

Di-*tert*-butyl (((*S*)-1-(tert-butoxy)-6-(5-(*N*-(hex-5-ynoyl)-*N*-(2,2,2-trifluoroethyl) sulfamoyl) pentanamido)-1-oxohexan-2-yl) carbamoyl)-*L*-glutamate (21a)

To a 4 mL vial, the precursor **S15** (30 mg, 0.068 mmol, 1.1 equiv.) was weighed and dissolved in 1 mL of DCM. The mixture was cooled down by dried ice and one mL TFA was added to the mixture. The mixture was stirred at rt for 45 min before transferred to a 50 mL round bottom flash. Solvent was removed under vacuum and the crude was co-evaporated 3 times to remove all TFA. The vacuum should be set to 40 mbar. The intermediate **15** was used directly for the next step without further purification.

To activate the carboxylic acid on the SuFA linker, two equivalents of HATU (47 mg, 0.12 mmol, 2.0 equiv.) were added, followed by 1 mL of dried DMF. The base DIPEA (0.052 mL, 0.31 mmol, 5.0 equiv.) was added to the mixture and stirred for 10 min. A solution of PMSA ligand **20** (30 mg, 0.061 mmol, 1.0 equiv., CAS: 1025796-31-9, Combi-Blocks) in 1 mL DMF was added in small portion into the activated SuFA linker solution and stirred at rt for 40 min. The mixture was filtered and purified on prep HPLC, gradient 20-100% ACN. The product has small UV peak (absorption of the carbonyl group) and it was eluted around 80-100% ACN in water. Fractions containing **21a** were combined, lyophilized and used directly for the next step. **MS** (ESI+): *m/z* 827.71 (M+H)$^+$.

(((*S*)-1-carboxy-5-(5-(N-(hex-5-ynoyl)-*N*-(2,2,2-trifluoroethyl) sulfamoyl) pentanamido) pentyl) carbamoyl) -*L*-glutamic acid (compound 21)

The starting material **21a** (30 mg, 0.061 mmol, 1.0 equiv.) was dissolved in 4 mL DCM and cooled to 0 °C. Three mL TFA was added slowly, and the mixture was stirred for 2 hrs. Solvent was removed under nitrogen flow and the crude was dissolved in DMSO, filtered before purified by prep HPLC 20-100% ACN in water (+0.1% formic). Obtained 15 mg of desired product (37% yield, 2 steps). **^{1}H NMR** (400 MHz, DMSO-d_6) δ 12.40 (s, 3H), 7.78 (t,

J = 5.6 Hz, 1H), 6.30 (dd, *J* = 12.4, 8.2 Hz, 2H), 4.55 (q, *J* = 8.9 Hz, 2H), 4.06 (dtd, *J* = 21.3, 8.1, 5.1 Hz, 2H), 3.67 – 3.59 (m, 2H), 3.00 (q, *J* = 6.6 Hz, 2H), 2.89 – 2.77 (m, 3H), 2.33 – 2.15 (m, 4H), 2.08 (t, *J* = 7.1 Hz, 2H), 1.98 – 1.84 (m, 1H), 1.80 – 1.57 (m, 8H), 1.55 – 1.44 (m, 1H), 1.43 – 1.32 (m, 2H), 1.31 – 1.23 (m, 2H). **^{13}C NMR** (101 MHz, DMSO-d_6) δ 174.5, 174.2, 173.7, 172.9, 171.3, 157.3, 125.2, 122.4, 83.6, 71.9, 53.6, 52.2, 51.6, 45.6, 45.2, 38.3, 34.5, 33.8, 31.8, 29.9, 28.8, 27.5, 23.6, 23.2, 22.6, 22.2, 16.8. **HRMS** (ESI+): *m/z* calcd for $C_{25}H_{38}F_3N_4O_{11}S$: 659.2204 [*M*+H]$^+$; found: 659.2190.

3.7 Synthesis of compound 23 (BTK SuFA probe)

(*R*)-*N*-((5-(3-(4-amino-3-(4-phenoxyphenyl)-1*H*-pyrazolo[3,4-d]pyrimidin-1-yl)piperidin-1-yl)-5-oxopentyl)sulfonyl)-*N*-(2,2,2-trifluoroethyl)hex-5-ynamide (compound 30a)

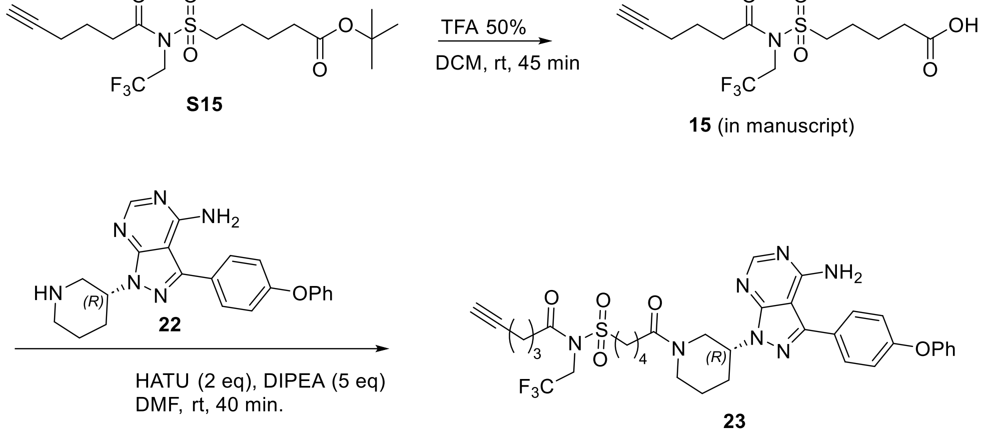

To a 4 mL vial, the precursor **S15** (35 mg, 0.085 mmol, 1.1 equiv.) was weighted and dissolved in 1 mL of DCM. The mixture was cooled down by dried ice and one mL TFA was added to the mixture. The mixture was stirred at rt for 45 min before transferring to a 50 mL round bottom flash. Solvent was removed under vacuum and the crude was co-evaporated 3 times to remove all TFA. Vacuum should be set up to 40 mbar. The intermediate **15** was used directly for the next step without further purification.

To activate the carboxylic acid on the SuFA linker, two equivalents of HATU (59 mg, 0.155 mmol) were added, followed by 1 mL of dried DMF. The base DIPEA (0.068 mL, 0.388 mmol, 5 equiv.) was added to the mixture and stirred for 10 min. A solution of ibrutinib analog **22** (30 mg, 0.077 mmol, 1 equiv., CAS: 1022150-12-4, Combi-Blocks) in 1 mL DMF was added in small portion into the activated SuFA linker solution and stirred at rt for 40 min. The mixture was filtered and purified on prep HPLC, gradient 20-100% ACN. Obtained 20 mg of desired product (35% yield). **^{1}H NMR** (400 MHz, $CDCl_3$) δ 8.36 (d, *J* = 11.8 Hz, 1H), 7.64 (dd, *J* = 8.7, 2.5 Hz, 2H), 7.43 – 7.34 (m, 2H), 7.17 (tt, *J* = 8.5, 2.3 Hz, 3H), 7.08 (d, *J* = 8.0 Hz, 2H), 5.67 (s, 2H), 4.82 (ddd, *J* = 11.7, 6.9, 4.2 Hz, 1H), 4.51 (d, *J* = 9.0 Hz, 2H), 4.13 – 3.79 (m, 1H), 3.73 – 3.25 (m, 3H), 3.18 – 2.65 (m, 3H), 2.51 – 2.17 (m, 7H), 2.07 – 1.88 (m, 6H), 1.82 (q, *J* = 7.1 Hz, 2H), 1.76 – 1.55 (m, 1H). **^{13}C NMR** (101 MHz, $CDCl_3$) δ 172.7, 172.7, 170.4, 170.3, 158.8, 158.7, 158.0, 157.9, 156.5, 156.4, 156.0, 155.7, 154.4, 154.3, 144.2, 144.0, 130.1, 130.1, 130.1, 127.9, 127.7, 125.0, 124.3, 124.2, 122.2, 119.7, 119.7, 119.3, 98.8, 98.7, 83.1, 70.0, 55.6, 53.5, 52.7, 50.0, 45.9, 45.6, 45.4, 45.1, 42.0, 34.5, 32.5, 30.4, 30.0, 25.3, 24.2, 23.7, 23.6, 23.3, 22.9, 22.9, 17.5. **HRMS** (ESI+): *m/z* calcd for $C_{35}H_{39}F_3N_7O_5S$: 726.2680 $[M+H]^+$; found: 726.26640.

3.8 Synthesis of compound 26 (VEGFR2 SuFA probe)

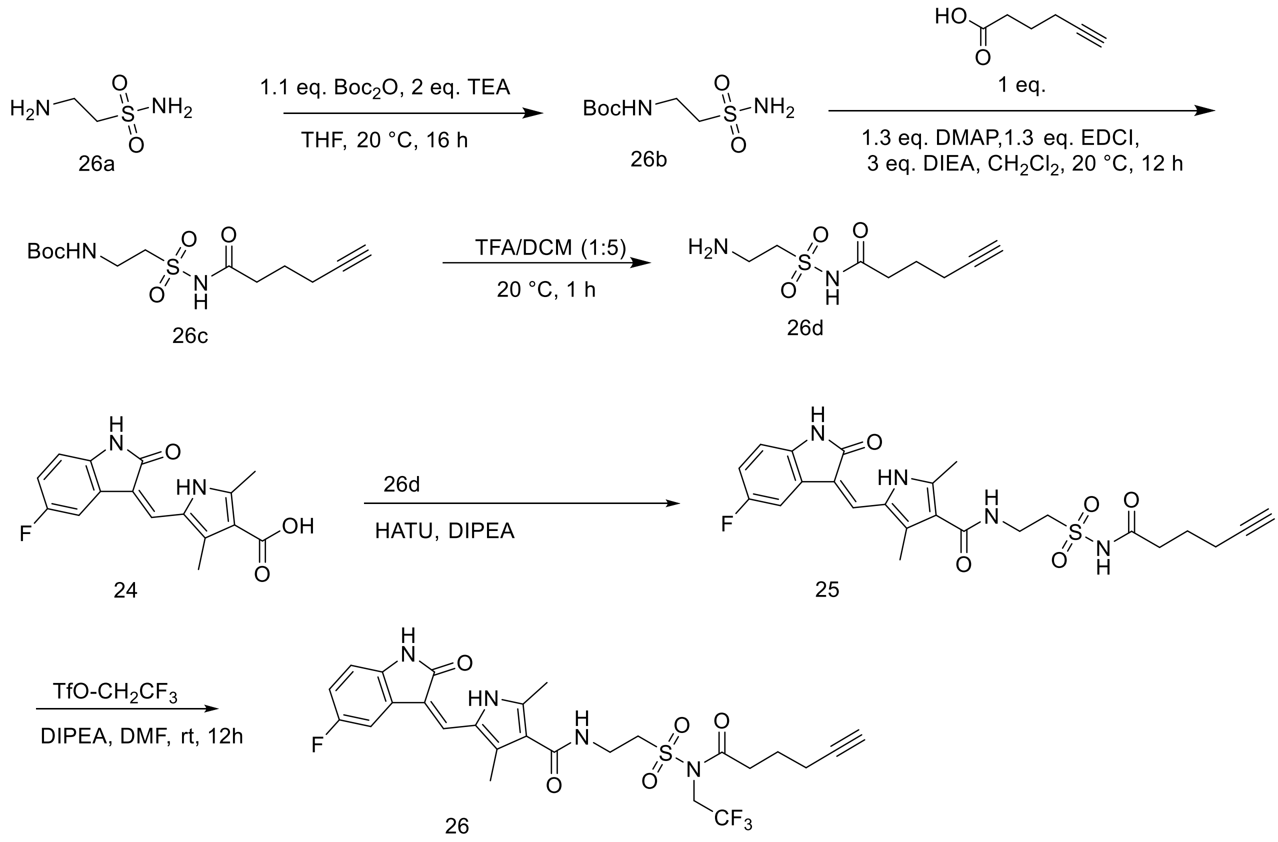

Tert-butyl *N*-(2-sulfamoylethyl) carbamate (26b)

To a solution of 2-aminoethanesulfonamide (5 g, 31.13 mmol, 1.0 equiv.) in THF (30 mL) was added Boc_2O (7.47 g, 34.24 mmol, 7.87 mL, 1.1 equiv.) and TEA (6.30 g, 62.26 mmol, 8.67 mL, 2.0 equiv.). The mixture was stirred at 20 °C for 16 h. TLC (Petroleum ether/Ethyl acetate=1/1) indicated starting material was consumed completely and one new spot formed. The mixture was concentrated to dryness. The mixture was diluted with 30 mL of H_2O and extracted with ethyl acetate (50 mL × 3). The combined organic layers were washed with brine (20 mL × 3), the combined organic layers were dried over anhydrous sodium sulfate, filtered and the filtrate was concentrated to the crude product *tert*-butyl *N*-(2-sulfamoylethyl)carbamate (6.3 g, 28.09 mmol, 90% yield) as a white solid. **^{1}H NMR** (400 MHz, DMSO-*d$_6$*) δ 7.03 - 6.64 (m, 3H), 3.31 - 3.26 (m, 2H), 3.08 (dd, J = 6.0, 8.8 Hz, 2H), 1.38 (s, 9H).

tert-butyl *N*-[2-(hex-5- ynoylsulfamoyl)ethyl]carbamate (26c)

To a solution of hex-5-ynoic acid (2.5 g, 22.30 mmol, 2.43 mL, 1.0 equiv.) in DMF (60 mL) was added EDCI (5.56 g, 28.99 mmol, 1.3 equiv.), DMAP (3.54 g, 28.99 mmol, 1.3 equiv.), DIPEA (11.6 mL, 66.89 mmol, 3.0 equiv.) and *t*ert-butyl *N*-(2-sulfamoylethyl)carbamate (6.00 g, 26.76 mmol, 1.2 equiv.). The mixture was stirred at 20 °C for 12 h. TLC(Petroleum ether/Ethyl acetate =1/1) showed no starting material remained. The mixture was diluted with 80 mL of H_2O and extracted with ethyl acetate (150 mL × 3). The combined organic layers were

washed with brine (100 mL × 3), the combined organic layers were dried over anhydrous sodium sulfate, filtered and the filtrate was concentrated. The crude product was triturated with 50 mL(Petroleum ether : Ethyl acetate =10/1) at 20 °C for 30 min. Then the crude product was triturated with 50 mL(Petroleum ether : Ethyl acetate =5/1) at 20 °C for 30 min to give *t*ert-butyl *N*-[2-(hex-5-ynoylsulfamoyl)ethyl]carbamate (5.73 g, 16.45 mmol, 73.78% yield, 91.41% purity) as a white solid. **^{1}H NMR** (400 MHz, DMSO-d_6) δ 6.99 - 6.91 (m, 1H), 6.90 - 6.82 (m, 1H), 3.50 - 3.43 (m, 2H), 3.30 - 3.22 (m, 2H), 2.81 (t, J = 2.4 Hz, 1H), 2.38 (t, J = 7.2 Hz, 2H), 2.18 (dt, J = 2.4, 7.2 Hz, 2H), 1.68 (quin, J = 7.2 Hz, 2H), 1.37 (s, 9H).

***N*-((2-aminoethyl)sulfonyl) hex-5-ynamide (26d)**

To a solution of *tert*-butyl *N*-[2-(hex-5-ynoylsulfamoyl)ethyl]carbamate (800 mg, 2.51 mmol, 1 equiv.) in DCM (8 mL) was added TFA (2.46 g, 21.61 mmol, 1.6 mL, 8.60 equiv.). The mixture was stirred at 20 °C for 1 hour. TLC(Petroleum ether/Ethyl acetate =1/1) showed no starting material remained. The mixture was concentrated to dryness to give the crude product *N*-(2-aminoethylsulfonyl)hex-5-ynamide (800 mg, 2.41 mmol, 95.81% yield, TFA) as a colorless oil. **^{1}H NMR** (400 MHz, DMSO-d_6) δ 8.02 (s, 3H), 3.72 - 3.62 (m, 2H), 3.18 (s, 2H), 2.82 (t, J = 2.4 Hz, 1H), 2.42 (t, J = 7.2 Hz, 2H), 2.19 (dt, J = 2.4, 7.2 Hz, 2H), 1.69 (quin, J = 7.2 Hz, 2H).

***(Z)*-5-((5-fluoro-2-oxoindolin-3-ylidene)methyl)-*N*-(2-(*N*-(hex-5-ynoyl)sulfamoyl)ethyl)-2,4-dimethyl-1*H*-pyrrole-3-carboxamide (25)**

To a solution of 5-[(*Z*)-(5-fluoro-2-oxo-indolin-3-ylidene)methyl]-2,4-dimethyl- 1H-pyrrole-3-carboxylic acid (600 mg, 2.00 mmol, 1 equiv.) in DMF (10 mL) was added HOBt (350.99 mg, 2.60 mmol, 1.3 equiv.), DIPEA (1.03 g, 7.99 mmol, 1.39 mL, 4 equiv.), *N*-(2-aminoethylsulfonyl)hex-5-ynamide (796.76 mg, 2.40 mmol, 1.2 *eq*, TFA) and EDCI (574.56 mg, 3.00 mmol, 1.5 equiv.). The mixture was stirred at 20 °C for 16 h. LCMS showed the desired compound was detected. The mixture was diluted with 30 mL of H_2O and extracted with ethyl acetate (50 mL × 3). The water phase was sent to lyophilization. The residue was purified by prep-HPLC (column: Waters xbridge 150*25mm 10um;mobile phase: [water(NH_4HCO_3)-ACN];B%: 12%-42%,8min) to give 5-[(*Z*)-(5-fluoro-2-oxo-indolin-3- ylidene)methyl]-*N*-[2-(hex-5-ynoylsulfamoyl)ethyl]-2,4-dimethyl-1*H*-pyrrole-3-carboxamide (21.86 mg, 0.040 mmol, 2.00% yield, 91.3% purity) as an orange solid. **^{1}H NMR** (400 MHz, DMSO-d_6) δ 13.71 (s, 1H), 10.90 (s, 1H), 7.77 (dd, J = 2.4, 9.4 Hz, 1H), 7.72 (s, 1H), 7.65 (t, J = 5.2 Hz, 1H), 6.93 (dt, J = 2.4, 8.8 Hz, 1H), 6.87 - 6.80 (m, 1H), 3.55 (q, J = 6.0 Hz, 2H), 3.46 - 3.37 (m, 2H), 2.76 (t, J = 2.4 Hz, 1H), 2.44 (d, J = 8.8 Hz, 6H), 2.26 - 2.19 (m, 2H), 2.15 (dt, J = 2.4, 7.1 Hz, 2H), 1.71 - 1.58 (m, 2H). **MS** (ESI+): *m/z* 501.3 $[M+H]^+$.

(*Z*)-5-((5-fluoro-2-oxoindolin-3-ylidene)methyl)-*N*-(2-(*N*-(hex-5-ynoyl)-*N*-(2,2,2-trifluoroethyl)sulfamoyl) ethyl)-2,4-dimethyl-1*H*-pyrrole-3-carboxamide (26)

To a solution of **25** (12 mg, 0.024 mmol) in 2 mL dry DMF, were added $TfOCH_2CF_3$ (1.5 equiv.) and DIPEA (3 equiv.). The mixture was stirred for 16 hrs at room temperature and monitored by LC-MS. The reaction was stopped by adding trifluoracetic acid at 0 °C and the crude was purified by prep HPLC (10-100% ACN in water

+ 0.1% formic) to afford the final product **26** (54% yield). **^{1}H NMR** (400 MHz, DMSO) δ 13.71 (s, 1H), 10.90 (s, 1H), 7.85 – 7.74 (m, 2H), 7.72 (s, 1H), 6.96 – 6.90 (m, 1H), 6.84 (dd, *J* = 8.5, 4.5 Hz, 1H), 4.97 – 4.55 (m, 2H), 3.90 (t, *J* = 6.5 Hz, 1H), 3.68 (dq, *J* = 12.6, 6.5 Hz, 2H), 3.53 – 3.24 (m, 1H), 2.97 – 2.76 (m, 3H), 2.45 (s, 3H), 2.43 (s, 3H), 2.25 (dtd, *J* = 14.2, 6.9, 2.5 Hz, 2H), 1.79 (dp, *J* = 28.1, 7.2 Hz, 2H). **^{13}C NMR** (101 MHz, DMSO-d_6) δ 173.7, 173.0, 169.6, 164.8, 159.4, 157.1, 136.9, 134.6, 130.4, 127.1, 127.0, 125.8, 124.9, 119.7, 114.9, 112.6, 112.4, 110.1, 106.1, 105.9, 83.6, 83.1, 72.1, 71.9, 53.4, 45.7, 45.1, 33.9, 33.7, 33.3, 32.0, 30.7, 24.3, 23.2, 17.2, 16.9, 13.4, 10.6, 10.6. **^{19}F NMR** (376 MHz, DMSO-d_6) δ -68.28, -72.06, -122.52, -122.53. Signal was report for both rotamers. **HRMS** (ESI+): *m/z* calcd for $C_{26}H_{27}F_4N_4O_5S$: 583.1633 $[M+H]^+$; found: 583.1625.

3.9 Synthesis of compound 27 (Halo-PSMA-GRC).

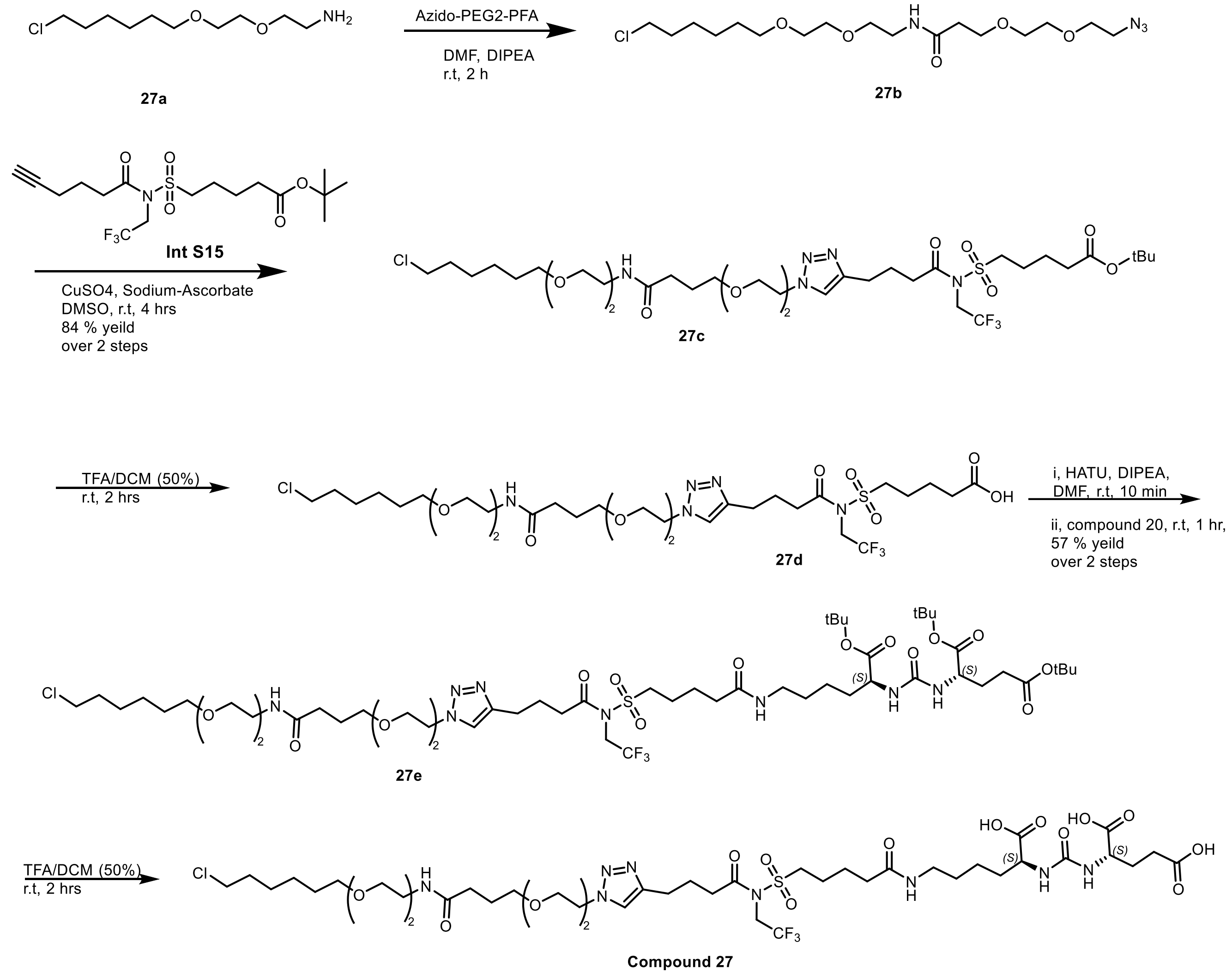

3-(2-(2-azidoethoxy)ethoxy)-N-(2-(2-((6-chlorohexyl)oxy)ethoxy)ethyl)propenamide (27b)

To a stirred solution of 2-(2-((6-chlorohexyl)oxy)ethoxy)ethan-1-amine (int **27a**, 112 mg, 0.5 mmol, 1.0 equiv.) in DMF (2 mL), where added Azido-PEG2-PFP ester (185 mg, 0.5 mmol, 1.0 equiv.) and DIPEA (261 µL, 1.5 mmol, 3 equiv.). The mixture was stirred at r.t for 2 hrs and diluted with brine and EtOAc. The organic layer was collected, concentrated under reduced pressure, and used for the next step without further purification.

***Tert*-butyl 5-(N-(4-(1-(22-chloro-9-oxo-3,6,13,16-tetraoxa-10-azadocosyl)-1*H*-1,2,3-triazol-4-yl)butanoyl)-N-(2,2,2-trifluoroethyl)sulfamoyl)pentanoate (27c)**

To a stirred solution of the resulting compound (**27d**) in DMSO (2 mL), where added tert-butyl 5-(*N*-(hex-5-ynoyl)-*N*-(2,2,2-trifluoroethyl)sulfamoyl)pentanoate (compound **S15**, 206 mg, 0.5 mmol, 1.0 equiv.), $CuSO_4$ (20 mol%) and sodium ascorbate (40 mol%). The reaction was monitored via LC-MS, and upon completion (4 hrs), it was purified on a reverse phase HPLC with acetonitrile/water (+0.1% formic acid) as eluent (isolation monitored by LCMS), providing 347 mg of **6c** (84% yield over two steps) of the product which was used for the next step.

Di-*tert*-butyl (((*S*)-1-(tert-butoxy)-6-(5-(*N*-(4-(1-(22-chloro-9-oxo-3,6,13,16-tetraoxa-10-azadocosyl)-1H-1,2,3-triazol-4-yl)butanoyl)-*N*-(2,2,2-trifluoroethyl)sulfamoyl)pentanamido) -1-oxohexan-2-yl)carbamoyl)-*L*-glutamate (27e)

Compound **27c** (82 mg, 0.1 mmol) was dissolved in 50% DCM/TFA (4 mL) and stirred at r.t for 2 hrs, after which volatiles were removed under reduced pressure. The resulting carboxylate (**27d**) is then dissolved in DMF (2 mL). Then HATU (57 mg, 0.15 mmol, 1.5 equiv.) and DIPEA (0.09 µL, 0.5 mmol, 5 equiv.) were added. The resulting solution is stirred at r.t for 10 minutes, after which di-*tert*-butyl (((*S*)-6-amino-1-(tert-butoxy)-1-oxohexan-2-yl)carbamoyl)-*L*-glutamate (compound **20**, 82 mg, 0.1 mmol, 1.0 equiv.) was added. The resulting mixture is stirred for an additional 1 hr. After this, it is purified on a reverse phase HPLC with acetonitrile/water (+0.1% formic acid) as eluent and product isolation was monitored by LCMS, providing 71 mg of **27e** (57 % yield over two steps).

(((*S*)-1-carboxy-5-(5-(*N*-(4-(1-(22-chloro-9-oxo-3,6,13,16-tetraoxa-10-azadocosyl)-1*H*-1,2,3-triazol-4-yl) butanoyl)-*N*-(2,2,2-trifluoroethyl)sulfamoyl)pentanamido)pentyl)carbamoyl)-*L*-glutamic acid (compound 25)

To access the final desired product, compound **27e** (71 mg, 0.057 mmol) is dissolved in 50% DCM/TFA (0.5 mL) and stirred at r.t for 2 h, after which volatiles are removed under reduced pressure. The crude mixture is then purified on a reverse-phase HPLC with acetonitrile/water (+0.1% formic acid) as eluent, providing 57 mg, 94% yield of pure product **27**. **^{1}H NMR** (400 MHz, DMSO) δ 12.43 (s, 3H), 7.88 (t, J = 5.6 Hz, 1H), 7.84 (s, 1H), 7.79 (t, J = 5.6 Hz, 1H), 6.36 – 6.27 (m, 2H), 4.56 (q, J = 8.8 Hz, 2H), 4.46 (t, J = 5.3 Hz, 2H), 4.08 (dtd, J = 21.7, 8.2, 5.2 Hz, 2H), 3.79 (t, J = 5.3 Hz, 2H), 3.68 – 3.33 (m, 17H), 3.19 (q, J = 5.8 Hz, 2H), 3.01 (q, J = 6.6 Hz, 2H), 2.82

(t, J = 7.1 Hz, 2H), 2.66 (t, J = 7.6 Hz, 2H), 2.34 – 2.16 (m, 4H), 2.10 (t, J = 7.1 Hz, 2H), 1.91 (h, J = 7.8 Hz, 19H), 1.71 – 1.24 (m, 4H). ^{13}**C NMR** (101 MHz, DMSO) δ 175.0, 174.7, 174.2, 173.6, 171.8, 170.6, 157.8, 146.5, 128.4, 125.6, 122.8, 122.8, 120.1, 70.6, 70.0, 69.9, 69.9, 69.9, 69.6, 69.2, 67.2, 54.2, 52.7, 52.1, 49.7, 46.3, 46.0, 45.8, 45.7, 45.3, 39.0, 38.8, 36.5, 35.0, 34.8, 32.5, 32.3, 30.4, 29.5, 29.3, 28.0, 26.6, 25.4, 24.6, 24.4, 24.1, 23.1, 22.7.^{19}**F NMR** (376 MHz, DMSO) δ -68.3. **HRMS** (ESI+): *m/z* calcd for $C_{42}H_{71}ClF_3N_8O_{16}S$: 1067.4344 $[M+H]^+$; found: 1067.4331.

3.10 Synthesis of compound 28 (Halo-PSMA-iGRC)

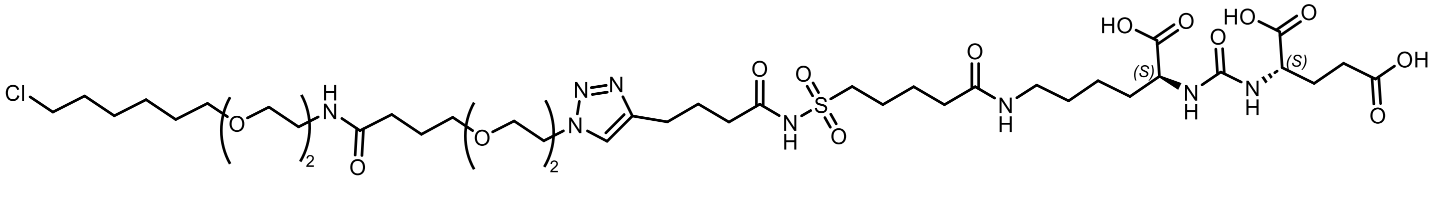

Compound 28

This compound was synthesized following the same reaction steps as the synthesis of compound **27**, but in instead of compound **S15** in the second step (click chemistry reaction), tert-butyl 5-(N-(hex-5-ynoyl)sulfamoyl)pentanoate is used.^{1}**H NMR** (400 MHz, CD_3OD) δ 7.83 (s, 1H), 4.56 (t, *J* = 5.1 Hz, 2H), 4.31 (ddd, *J* = 18.3, 8.5, 4.9 Hz, 2H), 3.90 (t, *J* = 5.1 Hz, 2H), 3.72 (t, *J* = 6.2 Hz, 2H), 3.66 – 3.34 (m, 25H), 3.27 – 3.14 (m, 2H), 2.77 (t, *J* = 7.5 Hz, 2H), 2.52 – 2.35 (m, 6H), 2.28 – 2.12 (m, 3H), 2.08 – 1.35 (m, 22H). ^{13}**C NMR** (101 MHz, CD_3OD) δ 175.1, 175.0, 174.5, 173.8, 172.9, 172.6, 122.9, 70.8, 70.0, 70.0, 69.9, 69.8, 69.2, 69.0, 66.9, 52.6, 52.2, 52.0, 50.0, 44.3, 39.0, 38.7, 36.2, 34.8, 34.8, 32.4, 31.8, 29.7, 29.1, 28.5, 27.5, 26.3, 25.1, 24.1, 24.0, 23.9, 22.6, 22.5. **HRMS** (ESI+): *m/z* calcd for $C_{40}H_{70}Cl_1N_8O_{16}S_1$: 985.4314 $[M+H]^+$; found: 985.4303.

3.11 Synthesis of compound 29 (FKBP-PSMA-GRC).

(((*S*)-1-carboxy-5-(5-(*N*-(4-(1-(1-(2-((*R*)-3-(3,4-dimethoxyphenyl)-1-(((*S*)-1-((*S*)-2-(3,4,5-trimethoxyphenyl) butanoyl)piperidine-2-carbonyl)oxy)propyl)phenoxy)-2-oxo-6,9,12-trioxa-3-azatetradecan-14-yl)-1*H*-1,2,3-triazol-4-yl)butanoyl)-*N*-(2,2,2-trifluoroethyl) sulfamoyl)pentanamido)pentyl)carbamoyl)-*L*-glutamic acid.

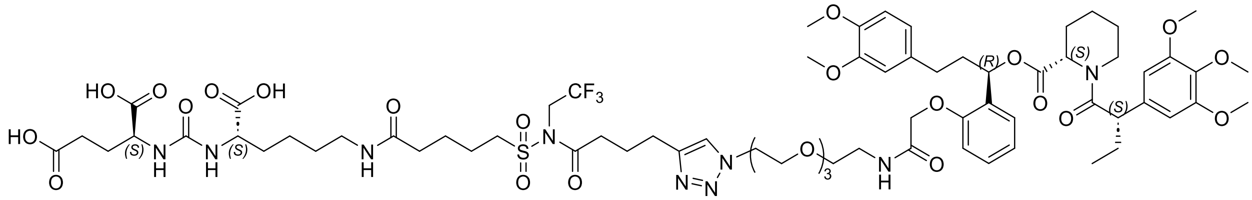

To a solution of compound **21** (7.5 mg, 0.011 mmol, 1.0 equiv.) and AP1867-peg3-azide (11.2 mg, 0.012 mmol, 1.1 equiv.) in 2 mL DMSO, was added sodium ascorbate 0.5 M (44 μL, 2.0 equiv.), followed by Cu (II) sulfate 0.5

M (22 µL, 1.0 equiv.) through a needle. The mixture was stirred under nitrogen for 1 hr. When the reaction was completed (monitored by LC-MS), the solution was filtered and purified by prep HPLC 10-100% ACN (+0.1% formic acid). Obtained 7 mg (36% yield). Major rotamer ^{1}**H NMR** (400 MHz, CD_3CN-*d_3*) δ 7.65 (s, 1H), 7.21 (t, *J* = 7.7 Hz, 1H), 7.12 (t, *J* = 5.8 Hz, 1H), 6.93 – 6.89 (m, 1H), 6.89 – 6.84 (m, 2H), 6.84 – 6.81 (m, 1H), 6.81 – 6.75 (m, 1H), 6.72 (s, 1H), 6.65 (d, *J* = 8.2 Hz, 1H), 6.58 – 6.51 (m, 2H), 6.06 (t, *J* = 6.4 Hz, 2H), 5.98 (d, *J* = 8.0 Hz, 1H), 5.39 (d, *J* = 5.4 Hz, 1H), 4.55 – 4.47 (m, 2H), 4.47 – 4.39 (m, 4H), 4.30 – 4.24 (m, 1H), 4.19 (q, *J* = 7.1 Hz, 1H), 4.01 (d, *J* = 13.7 Hz, 1H), 3.80 – 3.76 (m, 4H), 3.75 – 3.72 (m, 6H), 3.64 – 3.61 (m, 6H), 3.52 – 3.45 (m, 8H), 3.44 – 3.39 (m, 5H), 3.33 (q, *J* = 5.6 Hz, 2H), 3.16 – 3.09 (m, 2H), 2.78 – 2.70 (m, 4H), 2.66 – 2.59 (m, 1H), 2.56 – 2.49 (m, 1H), 2.45 – 2.35 (m, 3H), 2.23 – 2.15 (m, 3H), 2.13 – 2.04 (m, 2H), 2.03 – 1.96 (m, 3H), 1.89 – 1.84 (m, 1H), 1.82 – 1.72 (m, 4H), 1.70 – 1.56 (m, 6H), 1.56 – 1.51 (m, 1H), 1.43 (p, *J* = 5.8 Hz, 3H), 1.34 (q, *J* = 7.1 Hz, 3H), 1.23 – 1.12 (m, 1H), 0.83 (t, *J* = 7.3 Hz, 3H). ^{13}**C NMR** (101 MHz, CD_3CN-*d_3*) δ 175.2, 175.1, 174.9, 174.3, 173.5, 172.1, 169.4, 159.5, 155.1, 154.5, 154.2, 150.1, 150.0, 148.5, 148.4, 147.6, 137.6, 136.9, 134.8, 134.7, 130.1, 130.0, 128.1, 127.9, 126.3, 123.7, 123.5, 122.8, 121.3, 121.2, 118.3, 113.3, 113.2, 113.1, 112.8, 106.3, 105.8, 71.0, 71.0, 70.9, 70.8, 69.9, 68.3, 60.9, 60.8, 56.9, 56.8, 56.5, 56.4, 56.3, 55.3, 54.0, 53.5, 52.9, 51.0, 50.4, 46.8, 46.4, 44.5, 39.6, 37.5, 37.2, 35.9, 35.6, 32.1, 31.8, 30.8, 29.6, 29.1, 29.0, 28.2, 27.3, 26.1, 25.2, 24.8, 24.8, 23.4, 21.7, 12.9, 12.6. ^{19}**F NMR** (376 MHz, CD_3CN) δ -69.94. **HRMS** (ESI+): *m/z* calcd for $C_{71}H_{101}F_3N_9O_{24}S$: 1552.6627 $[M+H]^+$; found: 1552.6605.

3.12 Synthesis of compound 30 (FKBP-PSMA-iGRC)

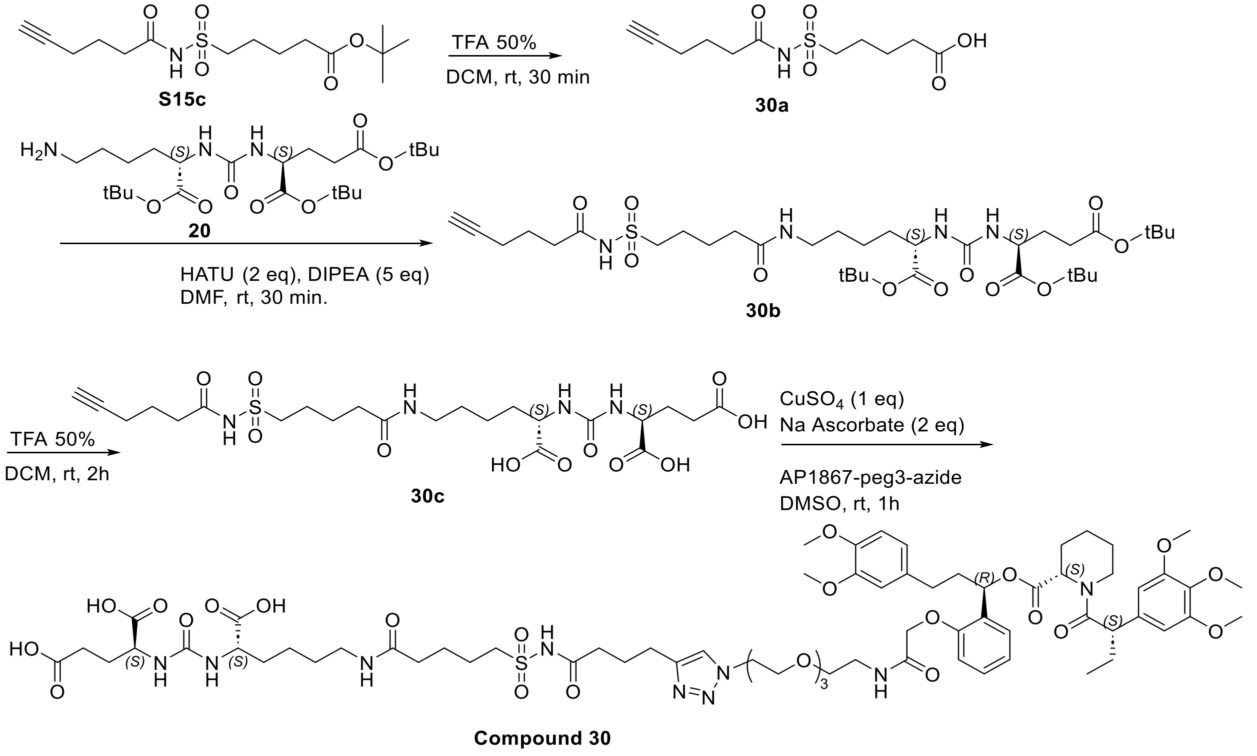

Di-*tert*-butyl (((*S*)-1-(tert-butoxy)-6-(5-(*N*-(hex-5-ynoyl)sulfamoyl)pentanamido)-1-oxohexan-2-yl)carbamoyl)-*L*-glutamate (30b)

To a 4 mL vial, **Int S15c** (31 mg, 0.092 mmol, 1.5 equiv.) was weighted and dissolved in 1 mL of DCM. The mixture was cooled down by dried ice and one mL TFA was added to the mixture. The mixture was stirred at rt for 45 min before transferred to a 50 mL round bottom flash. Solvent was removed under vacuum and the crude was co-evaporated 3 times to remove all TFA. Vacuum should be set to 40 mbar. The intermediate **30a** was used directly for the next step without further purification.

To activate the carboxylate on the linker, two equivalents of HATU (47 mg, 0.123 mmol) were added, followed by 1 mL of dried DMF. The base DIPEA (0.052 mL, 0.308 mmol, 5 equiv.) was added to the mixture and stirred for 10 min. A solution of PMSA ligand (30 mg, 0.061 mmol, 1 equiv.) in 1 mL DMF was added in small portion into the activated SuFA linker solution and stirred at rt for 40 min. The mixture was filtered and purified on prep HPLC, gradient 20-100% ACN. The product has small UV peak (absorption of the carbonyl group) and it was eluted around 80-100% ACN in water. Obtained 30 mg (59% yield) of **30b** which was used directly for the next step.

(((*S*)-1-carboxy-5-(5-(*N*-(hex-5-ynoyl)sulfamoyl)pentanamido)pentyl)carbamoyl)-*L*-glutamic acid (30c)

The starting material **30b** (22 mg, 0.032 mmol) was dissolved in 2 mL DCM and cooled to 0°C. Two mL TFA was added slowly, and the mixture was stirred for 2 hrs. Solvent was removed under nitrogen flow and the crude was dissolved in DMSO, filtered before purified by prep HPLC 10-90% ACN in water (+0.1% formic). Obtained 7.5 mg of desired product **30c** (41% yield). **^{1}H NMR** (400 MHz, DMSO-d_6) δ (ppm) 7.81 (t, *J* = 5.6 Hz, 1H), 6.31 (dd, *J* = 10.8, 8.2 Hz, 2H), 4.05 (dtd, *J* = 20.8, 8.1, 5.1 Hz, 2H), 3.33 (t, *J* = 7.3 Hz, 2H), 2.99 (q, *J* = 6.6 Hz, 2H), 2.82 (t, *J* = 2.7 Hz, 1H), 2.54 (s, 1H), 2.36 (t, *J* = 7.4 Hz, 2H), 2.23 (dt, *J* = 8.7, 6.7 Hz, 2H), 2.16 (td, *J* = 7.1, 2.6 Hz, 2H), 2.06 (t, *J* = 6.6 Hz, 2H), 1.97 – 1.83 (m, 1H), 1.77 – 1.55 (m, 8H), 1.54 – 1.45 (m, 1H), 1.39 – 1.32 (m, 2H), 1.30 – 1.17 (m, 2H). **^{13}C NMR** (101 MHz, DMSO-d_6) δ (ppm) 174.7, 174.3, 173.9, 172.3, 171.4, 157.4, 83.8, 71.9, 52.3, 51.7, 40.4, 38.4, 34.7, 34.3, 31.8, 30.0, 28.9, 27.6, 23.7, 23.0, 22.7, 22.6, 17.1. **MS** (ESI-): *m/z* 575.37 $(M-H)^-$.

(((*S*)-1-carboxy-5-(5-(*N*-(4-(1-(1-(2-((*R*)-3-(3,4-dimethoxyphenyl)-1-(((*S*)-1-((*S*)-2-(3,4,5-trimethoxyphenyl) butanoyl)piperidine-2-carbonyl)oxy)propyl)phenoxy)-2-oxo-6,9,12-trioxa-3-azatetradecan-14-yl)-1*H*-1,2,3 -triazol-4-yl)butanoyl)sulfamoyl)pentanamido) pentyl)carbamoyl)-*L*-glutamic acid (Compound 30)

To a solution of PSMA-iGRC **30c** (7.5 mg, 0.013 mmol, 1 equiv.) and FKBP-peg3-azide (11.63 mg, 0.013 mmol, 1 equiv.) in 2 mL DMSO under nitrogen, was added sodium ascorbate 0.5M (46 µL, 2 equiv.), followed by Cu (II) sulfate 0.5M (26 µL, 1 equiv.) through a needle. The mixture was stirred under nitrogen for 1h. When the reaction was completed (monitored by LC-MS), the solution was filtered and purified by prep HPLC 10-100% ACN (+0.1% formic acid). Obtained 6.3 mg of compound **30** (36% yield). **^{1}H NMR** (400 MHz, CD_3OD) δ (ppm) 7.81 (s, 1H), 7.25 (td, *J* = 7.8, 1.7 Hz, 1H), 6.95 – 6.84 (m, 3H), 6.81 – 6.74 (m, 2H), 6.70 (dd, *J* = 8.2, 2.0 Hz, 1H), 6.65 (s,

2H), 6.15 (dd, *J* = 8.0, 5.7 Hz, 1H), 5.45 (d, *J* = 5.3 Hz, 1H), 4.62 – 4.40 (m, 4H), 4.31 (ddd, *J* = 18.6, 8.6, 4.8 Hz, 2H), 4.16 (d, *J* = 13.7 Hz, 1H), 3.91 (t, *J* = 7.3 Hz, 1H), 3.85 – 3.75 (m, 10H), 3.70 (d, *J* = 4.8 Hz, 8H), 3.58 – 3.47 (m, 10H), 3.42 (q, *J* = 6.9 Hz, 4H), 3.17 (hept, *J* = 6.7 Hz, 2H), 2.74 (t, *J* = 7.6 Hz, 2H), 2.67 – 2.54 (m, 2H), 2.51 – 2.36 (m, 5H), 2.31 – 2.12 (m, 4H), 2.07 – 1.89 (m, 6H), 1.86 – 1.62 (m, 9H), 1.60 – 1.50 (m, 3H), 1.44 (q, *J* = 7.5 Hz, 2H), 1.30 – 1.15 (m, 1H), 0.91 (t, *J* = 7.3 Hz, 3H). **^{13}C NMR** (101 MHz, CD_3OD) δ 176.5, 175.9, 175.2, 174.8, 174.2, 172.3, 170.8, 160.1, 155.5, 154.8, 154.6, 150.3, 148.7, 147.9, 137.9, 136.9, 135.1, 130.4, 130.2, 128.2, 124.3, 123.2, 121.8, 113.5, 113.2, 113.0, 106.5, 105.8, 71.5, 71.4, 71.3, 71.0, 70.4, 70.2, 68.4, 61.1, 56.5, 56.5, 56.4, 54.0, 53.5, 53.4, 53.3, 51.3, 51.0, 45.0, 40.1, 39.9, 37.8, 36.2, 36.1, 33.2, 32.2, 31.1, 29.9, 29.3, 28.9, 27.6, 26.4, 25.4, 25.4, 25.3, 24.0, 23.9, 21.9, 12.6. **HRMS** (ESI+): *m/z* calcd for $C_{69}H_{99}N_9O_{24}S_1$: 1470.6596 $[M+H]^+$; found: 1470.6586.

3.13 Synthesis of compound 31 (Halo-BTK-GRC)

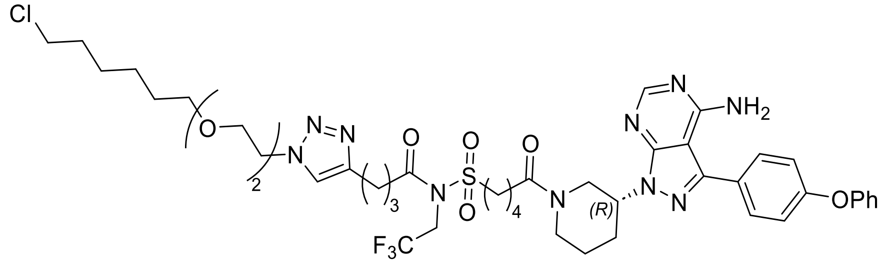

To a solution of compound **23** (8 mg, 0.011 mmol, 1.0 equiv.) and 1-(2-(2-azidoethoxy)ethoxy)-6-chlorohexane (11.2 mg, 0.012 mmol, 1.1 equiv., CAS: 2568146-55-2, MedChemExpress) in 2 mL DMSO, was added sodium ascorbate 0.5 M (44 µL, 2.0 equiv.), followed by Cu (II) sulfate 0.5 M (22 µL, 1.0 equiv.) through a needle. The mixture was stirred under nitrogen for 1 hr. When the reaction was completed (monitored by LC-MS), the solution was filtered and purified by prep HPLC 10-100% ACN (+0.1% formic acid). Obtained 9.1 mg (85% yield**). ^{1}H NM**R (400 MHz, CD_3CN-*d$_3$*) δ 8.27 (d, *J* = 7.8 Hz, 1H), 7.65 (d, *J* = 8.6 Hz, 2H), 7.59 (d, *J* = 6.5 Hz, 1H), 7.42 (dd, *J* = 8.6, 7.3 Hz, 2H), 7.23 – 7.07 (m, 5H), 5.89 (s, 2H), 4.85 – 4.69 (m, 1H), 4.68 – 4.57 (m, 2H), 4.53 – 4.46 (m, 2H), 4.45 – 4.40 (m, 2H), 4.27 (d, *J* = 13.6 Hz, 1H), 4.07 – 3.97 (m, 1H), 3.81 – 3.77 (m, 2H), 3.67 – 3.60 (m, 1H), 3.59 – 3.54 (m, 3H), 3.53 – 3.50 (m, 3H), 3.47 – 3.44 (m, 2H), 3.38 – 3.32 (m, 2H), 3.19 – 3.06 (m, 1H), 2.78 (dt, *J* = 14.4, 7.1 Hz, 2H), 2.69 (dt, *J* = 13.7, 7.4 Hz, 2H), 2.47 – 2.38 (m, 1H), 2.38 – 2.21 (m, 2H), 2.20 – 2.12 (m, 2H), 1.89 – 1.78 (m, 2H), 1.77 – 1.65 (m, 5H), 1.50 (p, *J* = 6.8 Hz, 2H), 1.45 – 1.35 (m, 2H), 1.34 – 1.25 (m, 2H). **^{13}C NMR** (101 MHz, CD_3CN) δ 174.3, 171.5, 171.4, 170.8, 170.4, 159.2, 158.9, 157.8, 156.7, 156.7, 155.5, 155.3, 147.6, 144.6, 131.1, 129.4, 129.3, 126.3, 124.9, 124.8, 123.5, 123.1, 120.2, 120.1, 101.1, 99.0, 73.4, 71.6, 71.1, 70.6, 70.0, 55.5, 54.0, 53.3, 50.7, 50.4, 46.7, 46.4, 46.2, 42.3, 35.6, 33.3, 32.9, 32.8, 30.7, 30.5, 30.3, 27.4, 26.1, 25.7, 25.2, 25.1, 25.0, 24.5, 24.3, 23.6, 21.1, 20.9. **^{19}F NMR** (376 MHz, CD_3CN-*d$_3$*) δ -70.10, -70.11. **HRMS** (ESI+): *m/z* calcd for $C_{45}H_{59}Cl_1F_3N_{10}O_7S$: 975.3924 $[M+H]^+$; found: 975.3902.

3.14 Synthesis of compound 32 (Halo-BTK-iGRC)

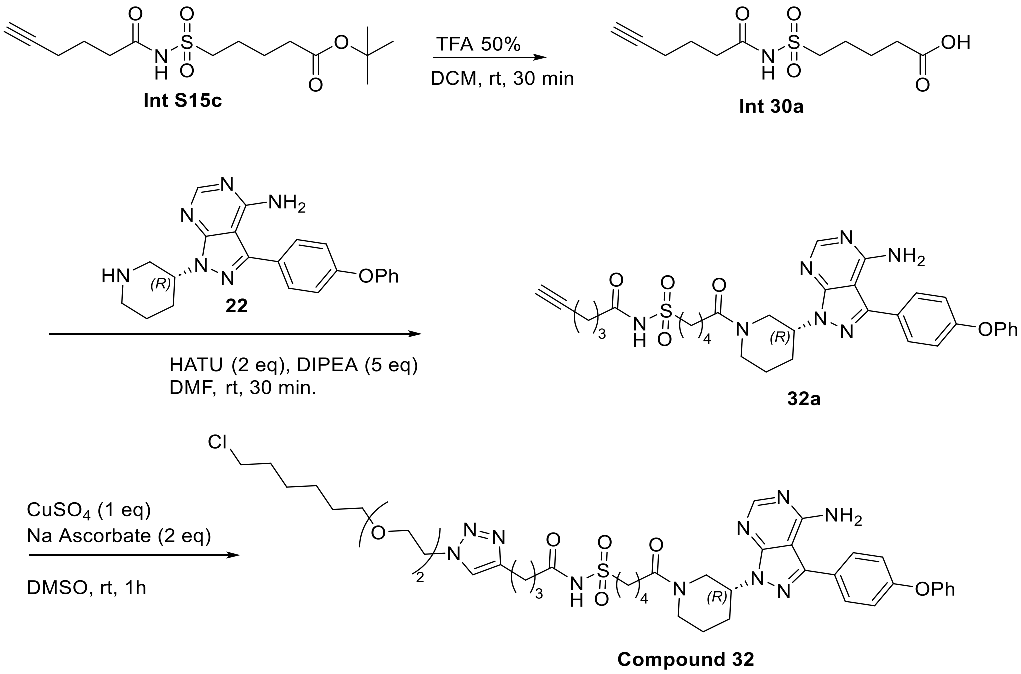

(*R*)-*N*-((5-(3-(4-amino-3-(4-phenoxyphenyl)-1H-pyrazolo[3,4-d]pyrimidin-1-yl)piperidin-1-yl)-5-oxopentyl) sulfonyl)hex-5-ynamide (32a)

To a 4 mL vial, **Int S15c** (16.5 mg, 0.060 mmol, 1.5 equiv.) was weighted and dissolved in 1 mL of DCM. The mixture was cooled down by dried ice and one mL TFA was added to the mixture. The mixture was stirred at rt for 45 min before transferred to a 50 mL round bottom flash. Solvent was removed under vacuum and the crude was co-evaporated 3 times to remove all TFA. Vacuum should be set up to 40 mbar. The intermediate **30a** was used directly for the next step without further purification.

To activate the carboxylate on the linker **30a**, two equivalents of HATU (30 mg, 0.079 mmol) were added, followed by 1 mL of dried DMF. The base DIPEA (0.034 mL, 0.197 mmol, 5 equiv.) was added to the mixture and stirred for 10 min. A solution of ibrutinib analogs (15.4 mg, 0.040 mmol, 1 equiv.) in 1 mL DMF was added in small portion into the activated linker **30a** solution and stirred at rt for 40 min. The mixture was filtered and purified on prep HPLC, gradient 20-100% ACN. The product has small UV peak (absorption of the carbonyl group) and it was eluted around 80-100% ACN in water. Obtained 6 mg of **32a** (59% yield) which was used directly for the next step.

(*R*)-*N*-((5-(3-(4-amino-3-(4-phenoxyphenyl)-1*H*-pyrazolo[3,4-d]pyrimidin-1-yl)piperidin-1-yl)-5-oxopentyl) sulfonyl)-4-(1-(2-(2-((6-chlorohexyl)oxy)ethoxy)ethyl)-1*H*-1,2,3-triazol-4-yl)butanamide (compound 32)

To a solution of **32a** (5.0 mg, 0.007 mmol, 1.0 equiv.) and 1-(2-(2-azidoethoxy)ethoxy)-6-chlorohexane (1.9 mg, 0.008 mmol, 1.1 equiv., CAS: 2568146-55-2, MedChemExpress) in 2 mL DMSO under nitrogen, was added sodium ascorbate 0.5M (28 µL, 2 equiv.), followed by Cu (II) sulfate 0.5M (14 µL, 1.0 equiv.) through a needle. The mixture was stirred under nitrogen for 1h. When the reaction was completed (monitored by LC-MS), the

solution was filtered and purified by prep HPLC 10-100% ACN (+0.1% formic acid). Obtained 5.0 mg of compound **32** (74% yield). **^{1}H NMR** (400 MHz, CD_3CN-d_3) δ 8.27 (d, *J* = 14.0 Hz, 1H), 7.65 (d, *J* = 8.3 Hz, 2H), 7.59 (d, *J* = 5.0 Hz, 1H), 7.42 (t, *J* = 7.7 Hz, 2H), 7.20 (d, *J* = 7.5 Hz, 1H), 7.16 (dd, *J* = 8.7, 2.2 Hz, 2H), 7.11 (d, *J* = 8.0 Hz, 2H), 5.86 (s, 2H), 4.86 – 4.22 (m, 4H), 4.10 – 3.85 (m, 1H), 3.79 (t, *J* = 5.3 Hz, 2H), 3.64 – 3.54 (m, 2H), 3.53 – 3.49 (m, 2H), 3.47 – 3.44 (m, 2H), 3.42 – 3.30 (m, 4H), 3.11 (q, *J* = 10.2 Hz, 1H), 2.87 (t, *J* = 12.1 Hz, 0H), 2.68 (dt, *J* = 10.3, 7.5 Hz, 2H), 2.54 – 1.97 (m, 8H), 1.92 – 1.85 (m, 3H), 1.84 – 1.54 (m, 7H), 1.56 – 1.45 (m, 2H), 1.44 – 1.35 (m, 2H), 1.34 – 1.24 (m, 2H). **^{13}C NMR** (101 MHz, CD_3CN) δ 173.2, 171.7, 171.5, 159.3, 158.9, 157.8, 156.8, 147.4, 144.6, 131.1, 129.3, 124.8, 123.2, 120.2, 120.1, 71.6, 71.1, 70.6, 70.0, 54.0, 53.3, 53.3, 50.8, 46.7, 46.2, 42.3, 36.0, 33.3, 33.1, 32.9, 30.7, 30.3, 27.4, 26.1, 25.7, 25.1, 24.6, 24.4, 24.3, 23.8. **HRMS** (ESI+): *m/z* calcd for $C_{43}H_{58}Cl_1N_{10}O_7S_1$: 893.3894 $[M+H]^+$; found: 893.3873.

3.15 Synthesis of compound S1, S2 and S3 (GLP1R SuFA probe)

Compound S1

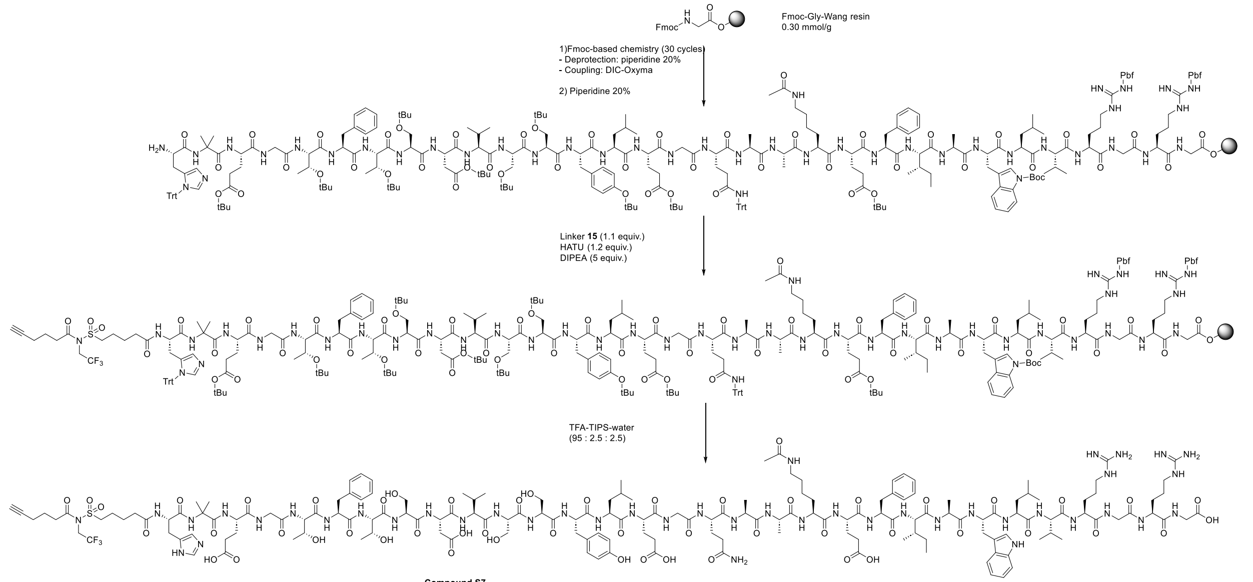

Compound S7

The precursor peptide was synthesized at 100 µmol scale on Fmoc-Gly-Wang ProTide resin (300 mg, loading 0.3 mmol/g) using microwave peptide synthesizer Liberty Blue 2.0 (CEM, Matthews, NC). In short, the Fmoc protecting group was removed with piperidine 20% in DMF (90 °C, 15 s; followed by 110 °C, 80 s) while peptide coupling was carried out with amino acid (5 equiv.), DIC (5 equiv.), oxyma (5 equiv.) (90 °C, 15 s; followed by 110 °C, 60 s) in DMF. Resin was washed with DMF between coupling and deprotection cycle or after every other reaction. Thirty cycles of deprotection/coupling were used to build the precursor peptide. When the last amino acid was coupled, the Fmoc was deprotected by piperdine 20% and washed thoroughly with DMF. Resin was washed 3 times with NMP, twice with solution of 2 equiv HOBt in NMP (5 min each, to remove piperidine traces), followed by 3 times NMP to clean all the reagent before SuFA linker coupling. In a round bottom flash, the SuFA linker (prepared from its tert-butyl ester as previously described) was activated by treating with two equivalents

of HATU (47 mg, 0.123 mmol), followed by 3 mL of dried DMF, and DIPEA (0.052 mL, 0.308 mmol, 5 equiv.). Resin was added in small portion into the activated SuFA linker solution and stirred at rt for 40 min. The excess reagent was removed by filtration and the resin was washed with thrice with DCM. The peptide was cleaved with TFA-water-TIPS (95:2.5:2.5) for 2 h (5 mL for 300 mg resin). The liquid was collected by filtration and concentrated under nitrogen flow until < 1mL. The peptide was precipitated in 8 mL cold ether. The solid was isolated by centrifugation (2000 rpm, 10 min at 4°C), dried for 5 min under nitrogen flow before redissolved in DMSO for purification. The product was purified by preparative HPLC using gradient 10-35% ACN in water (+0.1% formic) for 30 min on XBridge Prep C18 5μm 19x250mm OBD column (Waters, Milford, MA) with AccQPrep HP150 system (Teledyne, Thousand Oaks, CA). Obtained 8.7 mg. The peptide purity was assessed using Acquity UPLC-MS system (Waters, Milford, MA), equipped with BEH C18 1.7μm 2.1x50mm column. The gradient used ACN and water with 0.1% HCOOH (0 → 0.25 min, 5% ACN; 0.25 → 4.25 min, 5% → 95%; 4.25 → 4.75 min, 95%; 4.75 → 5.0 min, 95% → 5%). Peptides display purity 95% before used for testing. **MS** (ESI+): *m/z* 1260.44 $(M+3)^{3+}$.

Compound S2

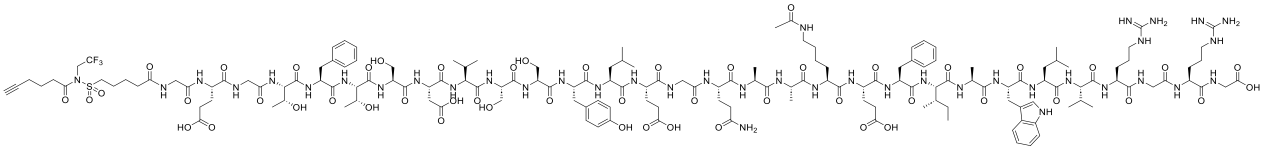

Used similar synthesis route as compound S7. The crude after cleavage made gel after dissolved in DMSO. A small amount of trifluoroacetic (40 μL for 4 mL DMSO) was added to keep the mixture in solution. Obtained 1.55 mg. **MS** (ESI+): *m/z* 1206.12 $(M+3H)^{3+}$.

Compound S3

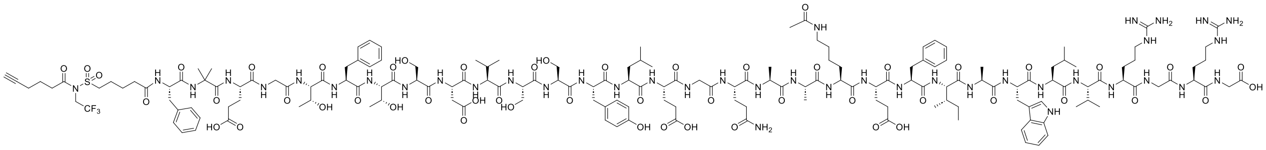

Used similar synthesis route as compound S7. The crude after cleavage made gel after dissolved in DMSO. A small amount of trifluoroacetic (40 μL for 4 mL DMSO) was added to keep the mixture in solution. Obtained 8.9 mg. **MS** (ESI+): *m/z* 1264.55 $(M+3H)^{3+}$.

3.16 Synthesis of compound S4 and S5 (GLP1R SuFA probe)

Compound S4

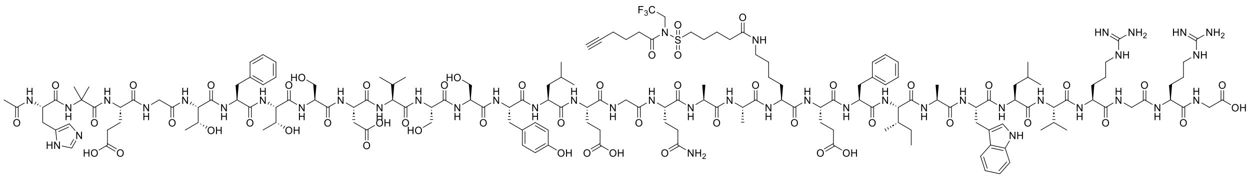

Used similar synthesis route as compound S**4**. Obtained 2.3 mg. **MS** (ESI+): *m/z* 1260.95 $(M+3H)^{3+}$.

Compound S5

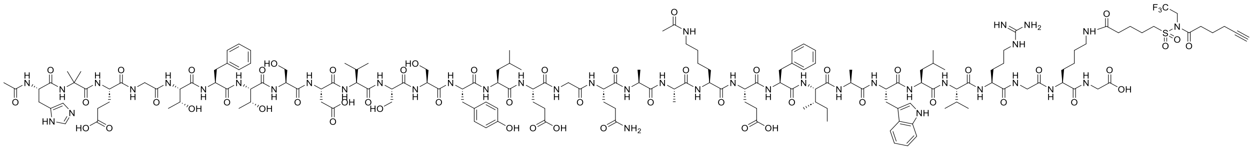

Used similar synthesis route as compound **S5**. Obtained 5.3 mg. **MS** (ESI+): *m/z* 1265.65 $(M+3H)^{3+}$.

3.17 Synthesis of compound S6 (RIPK1 SuFA probe)

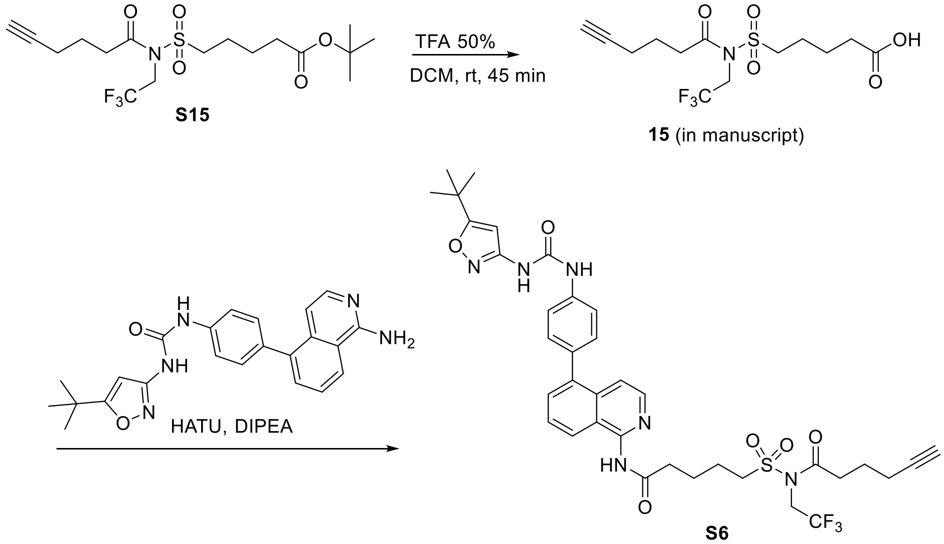

Compound **S6** was synthesized from **S15** and 1-(4-(1-aminoisoquinolin-5-yl)phenyl)-3-(5-(tert-butyl)isoxazol-3-yl)urea (RIPK1-IN-4, CAS: 1481641-08-0, 1Pluschem), following the procedure described for the synthesis of **23**, section 3.7. ^{1}H NMR (400 MHz, CD_3CN) δ 8.82 (s, 1H), 8.55 (s, 1H), 8.22 (s, 1H), 8.03 (s, 2H), 7.67 (q, *J* = 3.6 Hz, 2H), 7.64 – 7.56 (m, 3H), 7.45 – 7.38 (m, 2H), 6.33 (s, 1H), 4.50 (q, *J* = 8.7 Hz, 2H), 3.65 – 3.57 (m, 2H), 2.84 (t, *J* = 7.2 Hz, 2H), 2.63 (t, *J* = 6.9 Hz, 2H), 2.29 – 2.19 (m, 4H), 1.93 – 1.77 (m, 5H), 1.34 (s, 9H). ^{13}C NMR (101 MHz, CD_3CN) δ 182.08, 173.98, 159.59, 152.35, 141.94, 139.82, 139.51, 136.84, 134.80, 132.15, 131.35, 128.98, 127.76, 126.21, 125.88, 124.97, 123.43, 93.07, 84.30, 70.58, 55.46, 46.56 (q, *J* = 35.5 Hz), 36.18, 35.14, 33.54, 28.77, 24.45, 24.21, 23.46, 20.73, 17.85. ^{19}F NMR (376 MHz, CD_3CN) δ -70.06. **HRMS** (ESI+): *m/z* calcd for $C_{36}H_{40}F_3N_6O_6S_1$: 741.2677 $[M+H]^+$; found: 741.2646.

3.18 Synthesis of compound S7 (VEGFR2 NASA probe)

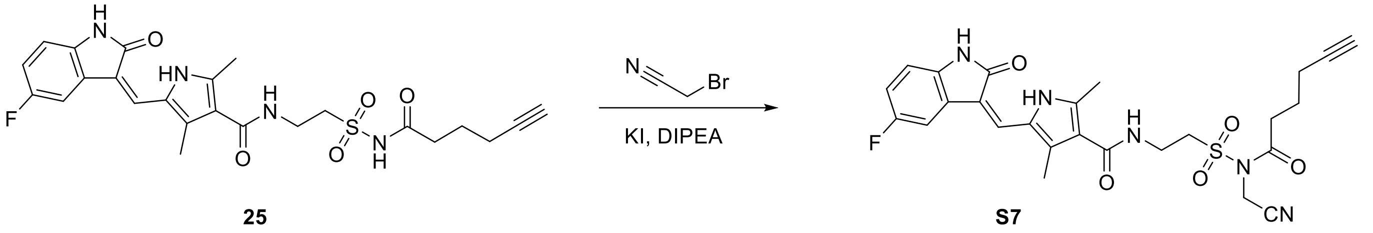

Compound **S7** was synthesized from **25** and bromoacetonitrile, following the procedure described for the synthesis of **26**, section 3.8. ^{1}H NMR (400 MHz, DMSO) δ 13.72 (s, 1H), 10.90 (s, 1H), 7.84 (t, *J* = 5.7 Hz, 1H), 7.77 (dd, *J* = 9.4, 2.6 Hz, 1H), 7.72 (s, 1H), 6.98 – 6.89 (m, 1H), 6.85 (dd, *J* = 8.5, 4.6 Hz, 1H), 4.81 (s, 2H), 3.90 (t, *J* = 6.5 Hz, 2H), 3.68 (q, *J* = 6.3 Hz, 2H), 2.90 – 2.79 (m, 3H), 2.44 (d, *J* = 9.8 Hz, 6H), 2.24 (td, *J* = 7.2, 2.7 Hz, 2H), 1.77 (p, *J* = 7.2 Hz, 2H). ^{13}C NMR (101 MHz, DMSO) δ 172.4, 169.6, 165.0, 159.4, 136.9, 134.6, 130.4, 127.0, 125.8, 124.9, 119.8, 116.5, 114.9, 114.9, 112.6, 112.4, 110.1, 110.0, 105.9, 83.7, 71.9, 53.3, 34.2, 33.7, 33.2, 23.2, 16.9, 13.4, 10.6, 1.2. **HRMS** (ESI+): *m/z* calcd for $C_{26}H_{27}FN_5O_5S_1$: 540.1711 $[M+H]^+$; found: 540.1695.

3.19 X-ray data for compound 9 and 11

Table S1. Experimental details of X-ray crystallography of compound 9

	Compound 9
Crystal data	
Chemical formula	$C_{10}H_{10}N_2O_3S$
M_r	238.26
Crystal system, space group	Orthorhombic, $P2_12_12_1$
Temperature (K)	100
a, *b*, *c* (Å)	4.9264 (1), 10.5515 (2), 20.5162 (4)
V (Å^3)	1066.45 (4)
Z	4
Radiation type	Mo *K*α
μ (mm^{-1})	0.30
Crystal size (mm)	0.18 × 0.12 × 0.10
Data collection	
Diffractometer	Bruker D8 goniometer with Photon area detector
Absorption correction	Multi-scan<br>*SADABS*

T_{min}, T_{max}	0.772, 0.801
No. of measured, independent and observed [$I > 2\sigma(I)$] reflections	33431, 2458, 2385
R_{int}	0.033
$(\sin \theta/\lambda)_{max}$ (Å^{-1})	0.649
Refinement	
$R[F^2 > 2\sigma(F^2)]$, $wR(F^2)$, S	0.022, 0.060, 1.07
No. of reflections	2458
No. of parameters	146
H-atom treatment	H-atom parameters constrained
$\Delta\rho_{max}$, $\Delta\rho_{min}$ (e Å^{-3})	0.32, -0.28
Absolute structure	Flack x determined using 954 quotients [(I+)-(I-)]/[(I+)+(I-)] (Parsons, Flack and Wagner, Acta Cryst. B69 (2013) 249-259).
Absolute structure parameter	-0.003 (16)

Computer programs: *SAINT* 8.40A (Bruker-AXS, 2019), *SHELXT2018* (Sheldrick, 2015), *SHELXL2018* (Sheldrick, 2015), Bruker *SHELXTL* (Sheldrick, 2015).

Table S2. Selected geometric parameters (Å, º) of the crystal structure of 9

S1—O1	1.4274 (13)	C3—C4	1.393 (2)
S1—O2	1.4328 (13)	C3—H3	0.9500
S1—N1	1.6749 (15)	C4—C5	1.386 (2)
S1—C6	1.7586 (16)	C4—H4	0.9500
O3—C7	1.201 (2)	C5—C6	1.388 (2)
N1—C7	1.423 (2)	C5—H5	0.9500
N1—C9	1.467 (2)	C7—C8	1.493 (2)
N2—C10	1.141 (3)	C8—H8A	0.9800
C1—C6	1.388 (2)	C8—H8B	0.9800
C1—C2	1.388 (3)	C8—H8C	0.9800
C1—H1	0.9500	C9—C10	1.471 (3)
C2—C3	1.387 (3)	C9—H9A	0.9900
C2—H2	0.9500	C9—H9B	0.9900
O1—S1—O2	119.98 (8)	C4—C5—H5	120.6
O1—S1—N1	108.08 (7)	C6—C5—H5	120.6

O2—S1—N1	104.18 (8)	C1—C6—C5	121.77 (16)
O1—S1—C6	108.60 (8)	C1—C6—S1	119.08 (13)
O2—S1—C6	109.36 (8)	C5—C6—S1	119.15 (13)
N1—S1—C6	105.70 (8)	O3—C7—N1	117.13 (16)
C7—N1—C9	115.18 (14)	O3—C7—C8	124.07 (17)
C7—N1—S1	126.01 (12)	N1—C7—C8	118.76 (15)
C9—N1—S1	117.72 (12)	C7—C8—H8A	109.5
C6—C1—C2	118.93 (17)	C7—C8—H8B	109.5
C6—C1—H1	120.5	H8A—C8—H8B	109.5
C2—C1—H1	120.5	C7—C8—H8C	109.5
C3—C2—C1	120.00 (17)	H8A—C8—H8C	109.5
C3—C2—H2	120.0	H8B—C8—H8C	109.5
C1—C2—H2	120.0	N1—C9—C10	110.80 (15)
C2—C3—C4	120.37 (17)	N1—C9—H9A	109.5
C2—C3—H3	119.8	C10—C9—H9A	109.5
C4—C3—H3	119.8	N1—C9—H9B	109.5
C5—C4—C3	120.15 (17)	C10—C9—H9B	109.5
C5—C4—H4	119.9	H9A—C9—H9B	108.1
C3—C4—H4	119.9	N2—C10—C9	177.25 (19)
C4—C5—C6	118.76 (16)		
O1—S1—N1—C7	31.39 (17)	C4—C5—C6—S1	178.73 (13)
O2—S1—N1—C7	160.04 (15)	O1—S1—C6—C1	-8.79 (16)
C6—S1—N1—C7	-84.73 (16)	O2—S1—C6—C1	-141.40 (14)
O1—S1—N1—C9	-136.05 (13)	N1—S1—C6—C1	106.98 (14)
O2—S1—N1—C9	-7.41 (15)	O1—S1—C6—C5	171.33 (13)
C6—S1—N1—C9	107.82 (14)	O2—S1—C6—C5	38.71 (15)
C6—C1—C2—C3	-0.2 (3)	N1—S1—C6—C5	-72.91 (15)
C1—C2—C3—C4	-0.7 (3)	C9—N1—C7—O3	8.0 (3)
C2—C3—C4—C5	0.7 (3)	S1—N1—C7—O3	-159.75 (15)
C3—C4—C5—C6	0.2 (3)	C9—N1—C7—C8	-169.86 (17)
C2—C1—C6—C5	1.2 (3)	S1—N1—C7—C8	22.4 (3)
C2—C1—C6—S1	-178.72 (14)	C7—N1—C9—C10	-83.29 (19)
C4—C5—C6—C1	-1.1 (2)	S1—N1—C9—C10	85.51 (16)

Table S3. Experimental details of X-ray crystallography for compound 11

	Compound **11**
Crystal data	
Chemical formula	$C_{10}H_{10}F_3NO_3S$
M_r	281.25
Crystal system, space group	Monoclinic, $P2_1/c$
Temperature (K)	100
a, *b*, *c* (Å)	10.3165 (3), 12.3031 (3), 9.6211 (3)
β (°)	106.8565 (9)
V (Å^3)	1168.69 (6)
Z	4
Radiation type	Mo *K*α
μ (mm^{-1})	0.32
Crystal size (mm)	0.24 × 0.18 × 0.16
Data collection	
Diffractometer	Bruker D8 goniometer with Photon area detector
Absorption correction	Multi-scan<br>*SADABS*
T_{min}, T_{max}	0.718, 0.746
No. of measured, independent and observed [$I > 2\sigma(I)$] reflections	35891, 2685, 2440
R_{int}	0.033
$(\sin \theta/\lambda)_{max}$ (Å^{-1})	0.649
Refinement	
$R[F^2 > 2\sigma(F^2)]$, $wR(F^2)$, S	0.028, 0.075, 1.03
No. of reflections	2685
No. of parameters	164
H-atom treatment	H-atom parameters constrained
$\Delta\rho_{max}$, $\Delta\rho_{min}$ (e Å^{-3})	0.35, -0.41

Computer programs: *SAINT* 8.40A (Bruker-AXS, 2019), *SHELXT2018* (Sheldrick, 2015), *SHELXL2018* (Sheldrick, 2015), Bruker *SHELXTL* (Sheldrick, 2015).

Table S4. Geometric parameters (Å, º) of crystal structure of 11

S1—O2	1.4264 (9)	C2—H2	0.9500
S1—O1	1.4324 (9)	C3—C4	1.388 (2)
S1—N1	1.7026 (11)	C3—H3	0.9500
S1—C6	1.7543 (13)	C4—C5	1.3885 (19)
F1—C10	1.3389 (18)	C4—H4	0.9500
F2—C10	1.3388 (18)	C5—C6	1.3905 (17)
F3—C10	1.3366 (18)	C5—H5	0.9500
O3—C7	1.2122 (16)	C7—C8	1.5061 (18)
N1—C7	1.4061 (16)	C8—H8A	0.9800
N1—C9	1.4618 (16)	C8—H8B	0.9800
C1—C2	1.3863 (18)	C8—H8C	0.9800
C1—C6	1.3910 (18)	C9—C10	1.510 (2)
C1—H1	0.9500	C9—H9A	0.9900
C2—C3	1.390 (2)	C9—H9B	0.9900
O2—S1—O1	119.28 (6)	C5—C6—C1	121.96 (12)
O2—S1—N1	108.15 (6)	C5—C6—S1	120.57 (10)
O1—S1—N1	104.29 (5)	C1—C6—S1	117.48 (10)
O2—S1—C6	110.58 (6)	O3—C7—N1	119.74 (12)
O1—S1—C6	108.22 (6)	O3—C7—C8	122.02 (12)
N1—S1—C6	105.30 (5)	N1—C7—C8	118.24 (11)
C7—N1—C9	123.46 (11)	C7—C8—H8A	109.5
C7—N1—S1	118.06 (9)	C7—C8—H8B	109.5
C9—N1—S1	117.88 (9)	H8A—C8—H8B	109.5
C2—C1—C6	118.73 (12)	C7—C8—H8C	109.5
C2—C1—H1	120.6	H8A—C8—H8C	109.5
C6—C1—H1	120.6	H8B—C8—H8C	109.5
C1—C2—C3	120.06 (13)	N1—C9—C10	111.46 (11)
C1—C2—H2	120.0	N1—C9—H9A	109.3
C3—C2—H2	120.0	C10—C9—H9A	109.3
C4—C3—C2	120.49 (13)	N1—C9—H9B	109.3
C4—C3—H3	119.8	C10—C9—H9B	109.3
C2—C3—H3	119.8	H9A—C9—H9B	108.0
C3—C4—C5	120.27 (12)	F3—C10—F2	106.34 (13)
C3—C4—H4	119.9	F3—C10—F1	107.24 (12)

C5—C4—H4	119.9	F2—C10—F1	107.23 (12)
C4—C5—C6	118.47 (12)	F3—C10—C9	113.00 (11)
C4—C5—H5	120.8	F2—C10—C9	112.66 (12)
C6—C5—H5	120.8	F1—C10—C9	110.04 (13)
O2—S1—N1—C7	-53.52 (10)	O1—S1—C6—C5	150.88 (10)
O1—S1—N1—C7	178.56 (9)	N1—S1—C6—C5	-98.05 (11)
C6—S1—N1—C7	64.71 (10)	O2—S1—C6—C1	-160.92 (10)
O2—S1—N1—C9	135.07 (9)	O1—S1—C6—C1	-28.58 (12)
O1—S1—N1—C9	7.15 (10)	N1—S1—C6—C1	82.49 (11)
C6—S1—N1—C9	-106.69 (10)	C9—N1—C7—O3	175.76 (12)
C6—C1—C2—C3	-0.8 (2)	S1—N1—C7—O3	4.87 (16)
C1—C2—C3—C4	-0.1 (2)	C9—N1—C7—C8	-5.23 (18)
C2—C3—C4—C5	1.0 (2)	S1—N1—C7—C8	-176.12 (9)
C3—C4—C5—C6	-1.1 (2)	C7—N1—C9—C10	93.52 (14)
C4—C5—C6—C1	0.16 (19)	S1—N1—C9—C10	-95.58 (12)
C4—C5—C6—S1	-179.27 (10)	N1—C9—C10—F3	58.38 (16)
C2—C1—C6—C5	0.8 (2)	N1—C9—C10—F2	-62.18 (16)
C2—C1—C6—S1	-179.79 (10)	N1—C9—C10—F1	178.22 (11)
O2—S1—C6—C5	18.54 (12)		

3.20 LC-MS traces of ligands in manuscript

3.20.1 Compound **1** (RIPK1 NASA probe)

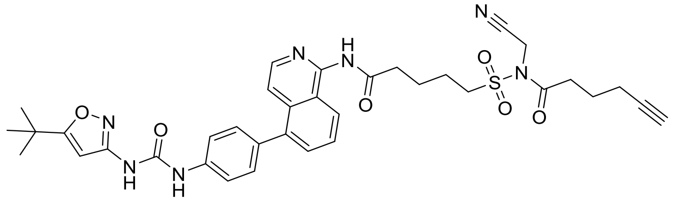

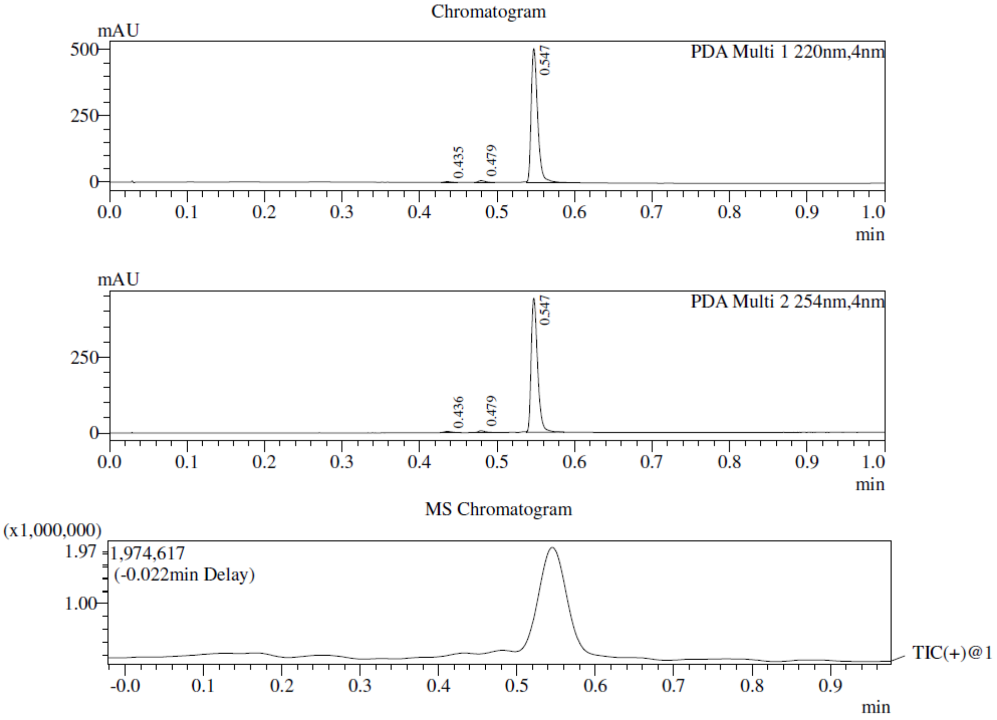

Mass Spectrum

RetTime: 0.480   DateFile: D:\Data\2022\2203\220315\EC6659-61-P1C2.lcd

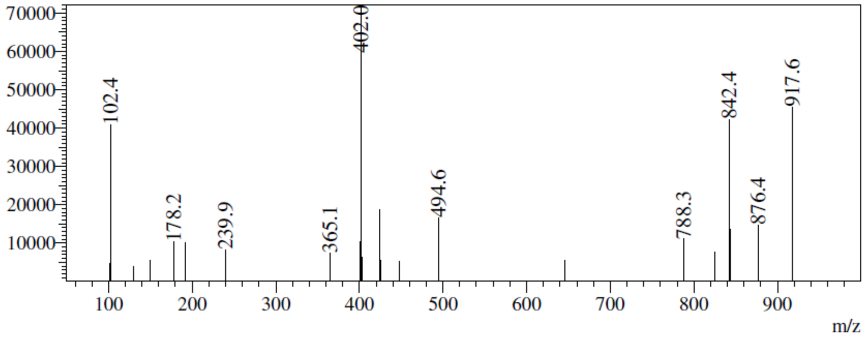

RetTime: 0.548   DateFile: D:\Data\2022\2203\220315\EC6659-61-P1C2.lcd

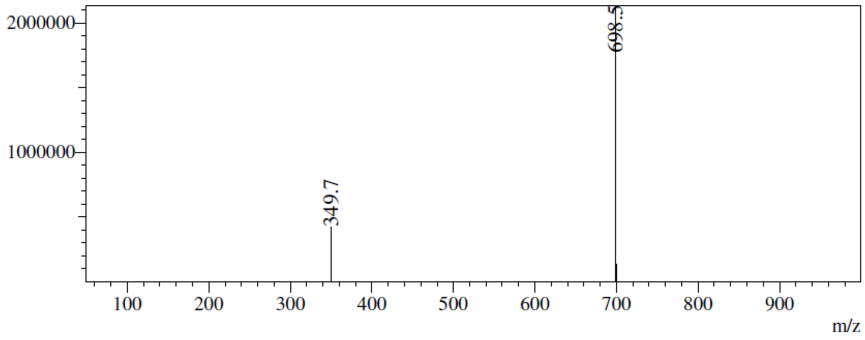

Analytical HPLC

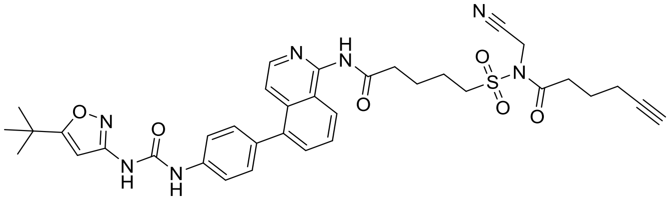

Chromatogram

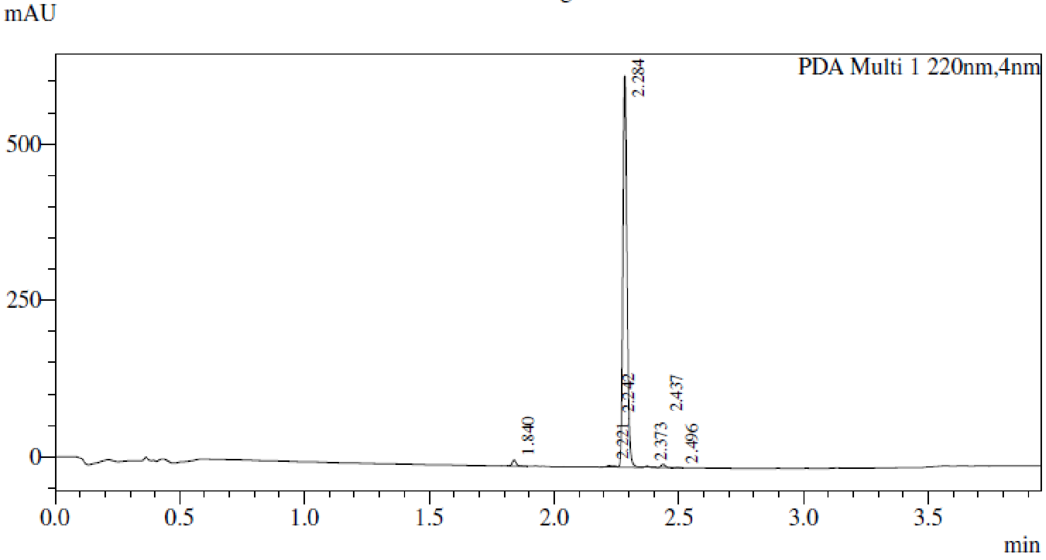

Integration Result

PDA Ch1 220nm

Peak#	Ret. Time	USP Width	Height	Height%	Area	Area%
1	1.840	0.029	9304	1.431	10617	1.426
2	2.221	0.035	2865	0.441	3181	0.427
3	2.242	0.042	2568	0.395	2814	0.378
4	2.284	0.030	626517	96.343	717062	96.309
5	2.373	0.030	2361	0.363	2855	0.383
6	2.437	0.033	5429	0.835	6710	0.901
7	2.496	0.028	1252	0.193	1304	0.175
Total			650296	100.000	744542	100.000

3.20.2 <u>Compound **2** (MEK2 NASA probe)</u>

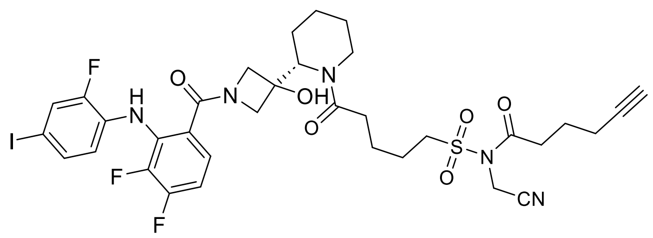

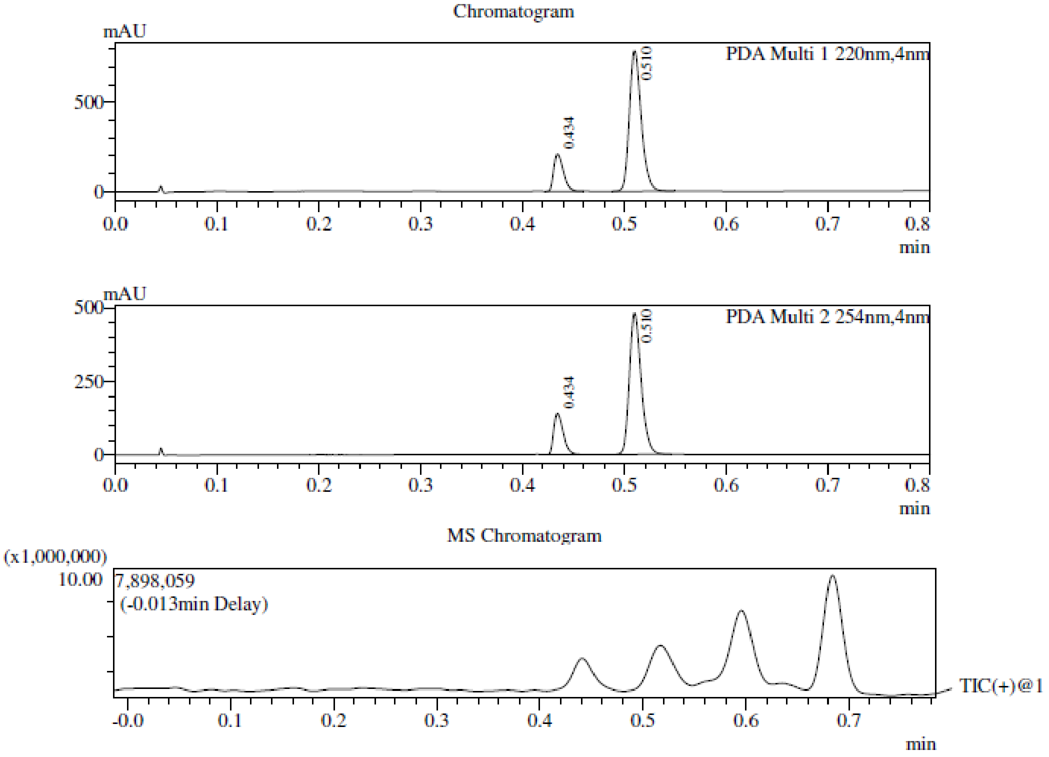

Mass Spectrum

RetTime: 0.434   DateFile: D:\Data\2022\2203\220302\EC6436-66-P1E.lcd

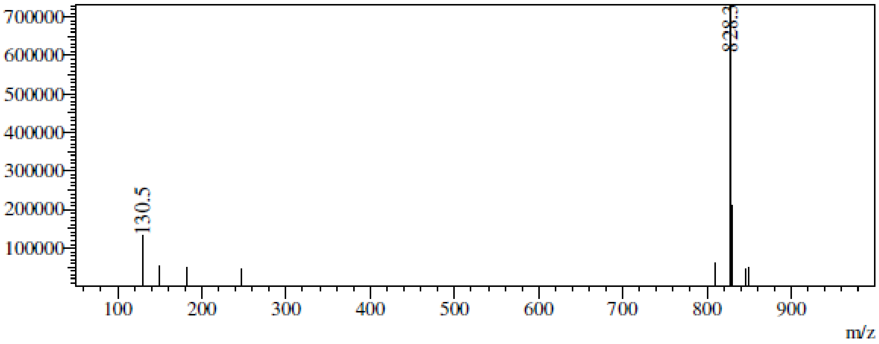

RetTime: 0.509   DateFile: D:\Data\2022\2203\220302\EC6436-66-P1E.lcd

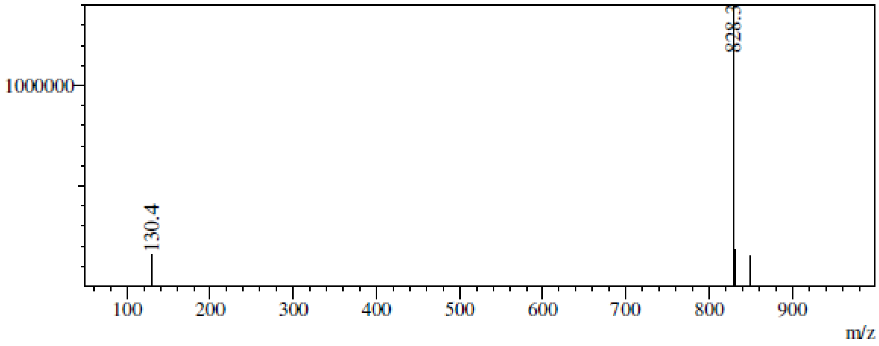

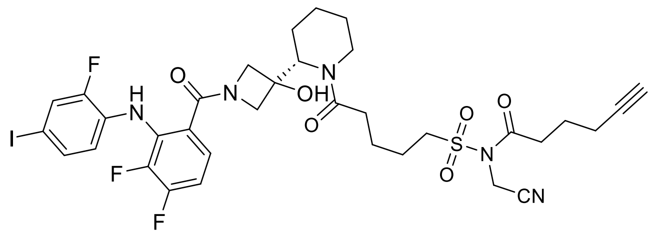

Analytical HPLC

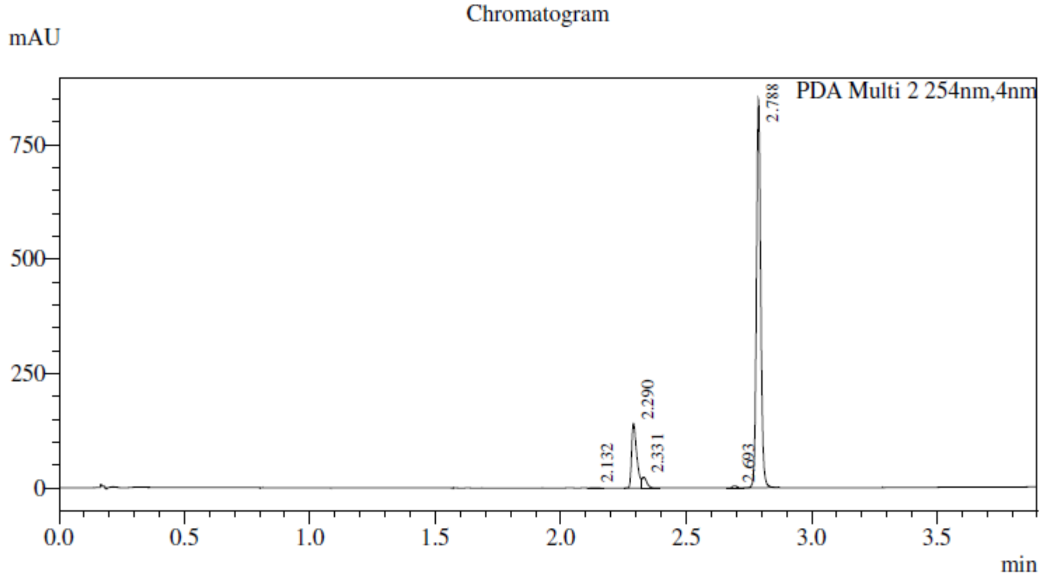

Integration Result

PDA Ch2 254nm

Peak#	Ret. Time	USP Width	Height	Height%	Area	Area%
1	2.132	0.034	1011	0.100	1325	0.106
2	2.290	0.038	140624	13.885	201795	16.102
3	2.331	0.059	23813	2.351	32684	2.608
4	2.693	0.031	4740	0.468	5646	0.451
5	2.788	0.031	842592	83.196	1011781	80.734
Total			1012780	100.000	1253232	100.000

3.20.3 <u>Compound **3** (IRE1α NASA probe)</u>

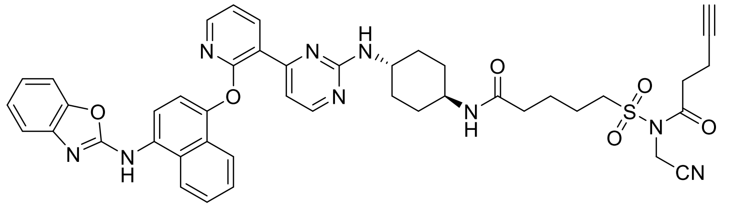

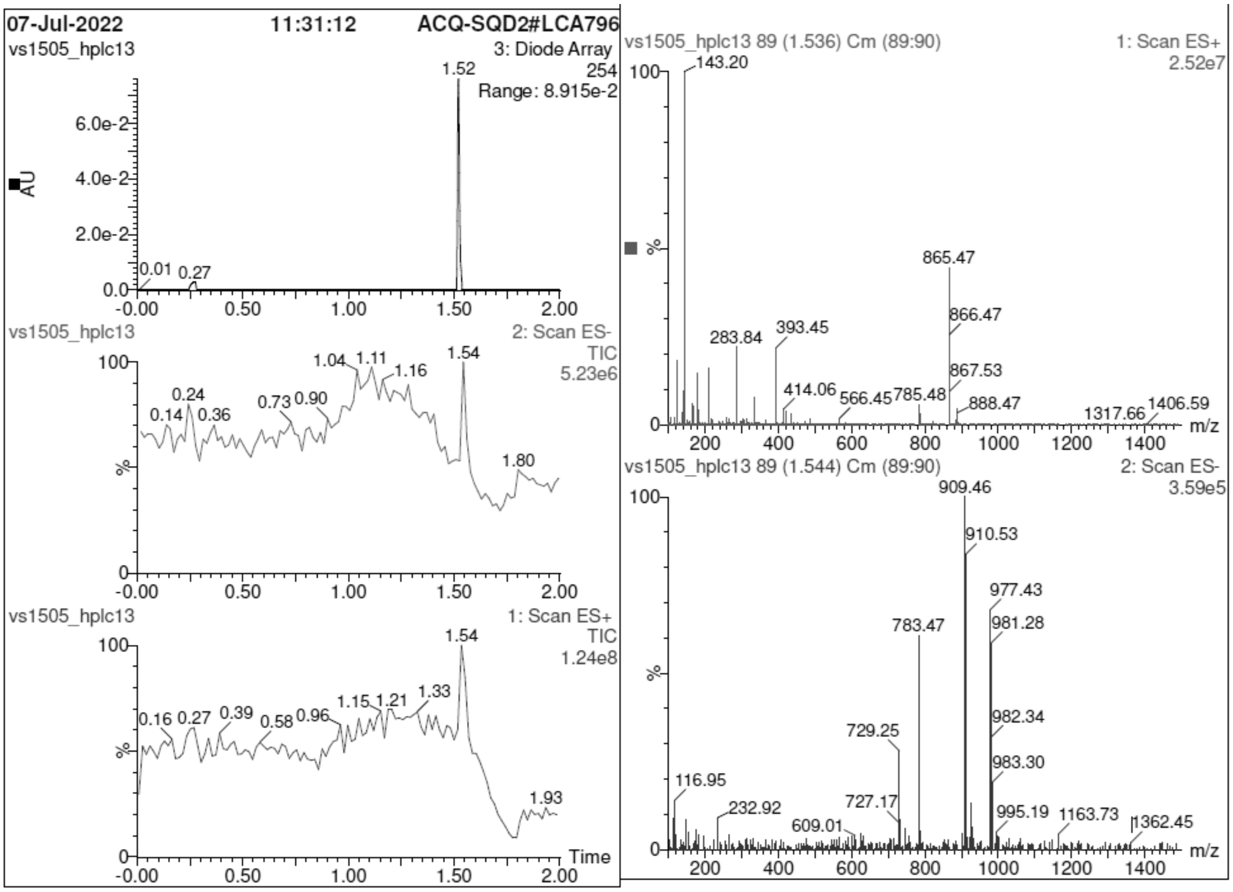

3.20.4 Compound **4** (MEK2 NASA probe)

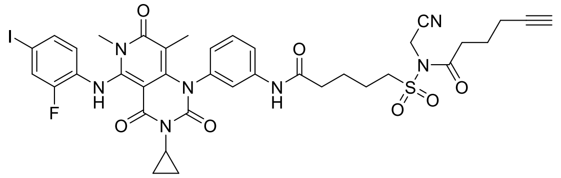

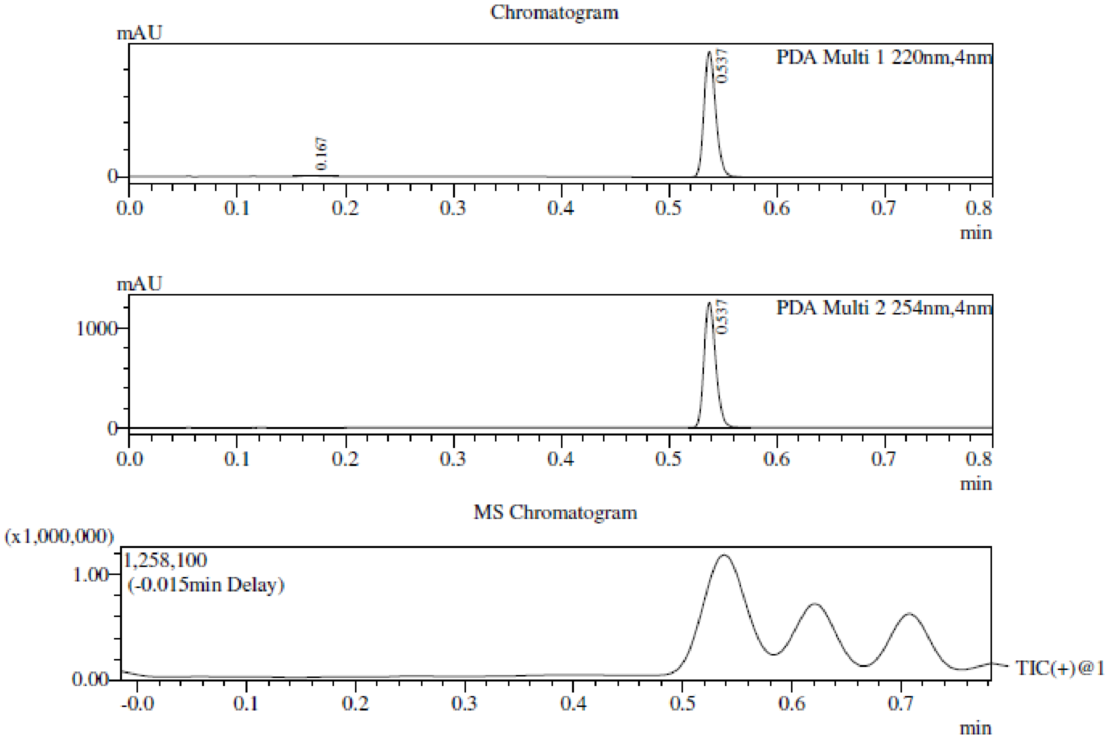

Mass Spectrum

RetTime: 0.167   DateFile: D:\Data\2022\2203\220302\EC6659-36-P1B.lcd

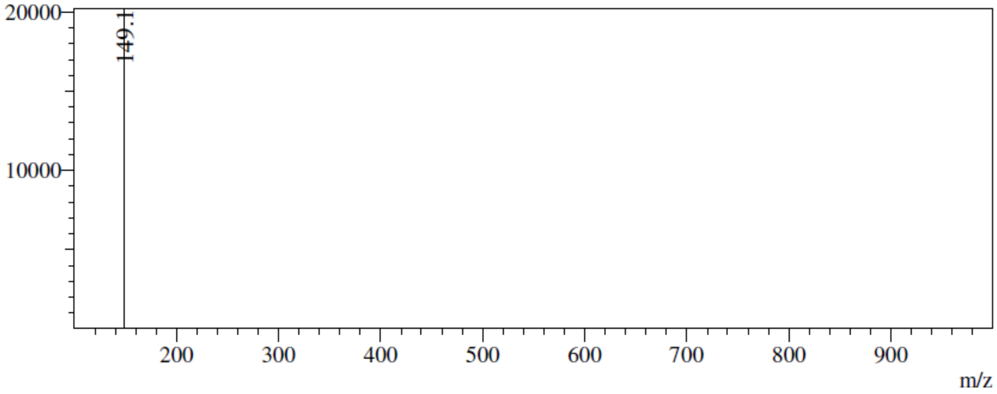

RetTime: 0.537   DateFile: D:\Data\2022\2203\220302\EC6659-36-P1B.lcd

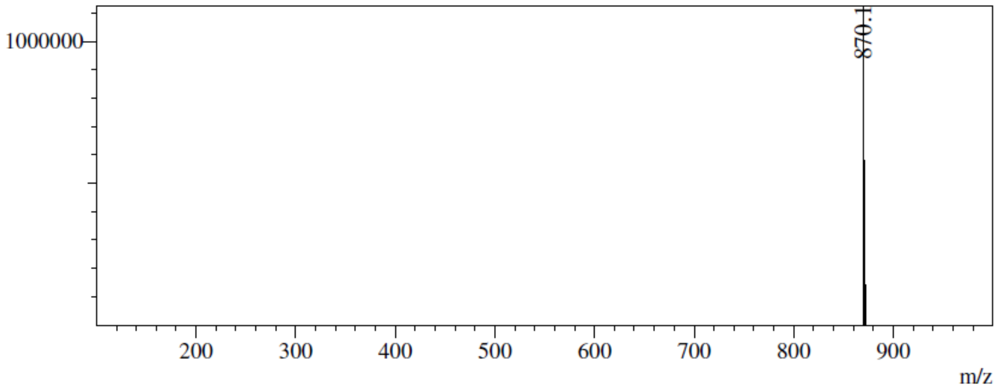

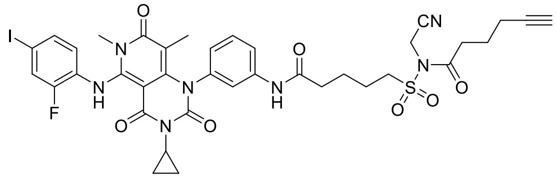

Analytical HPLC

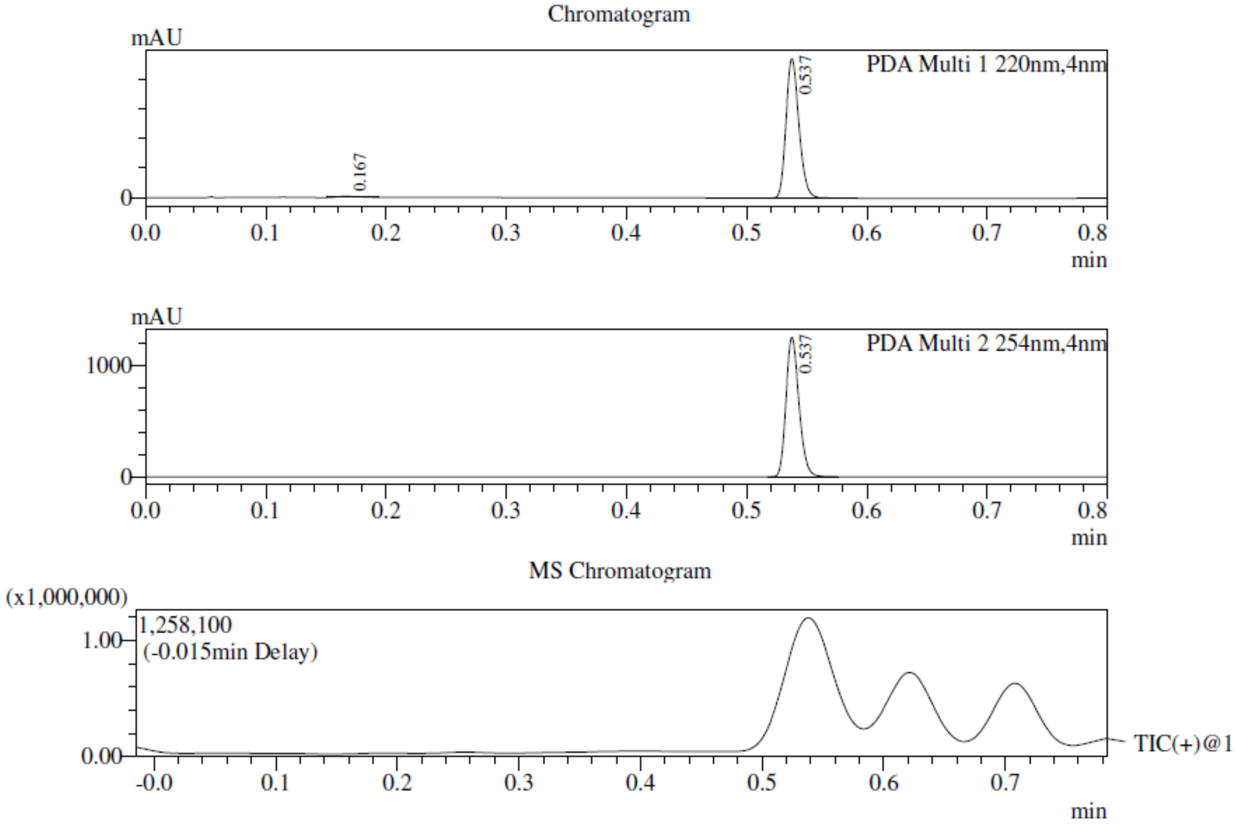

3.20.5 <u>Compound **6** (EGFR NASA probe)</u>

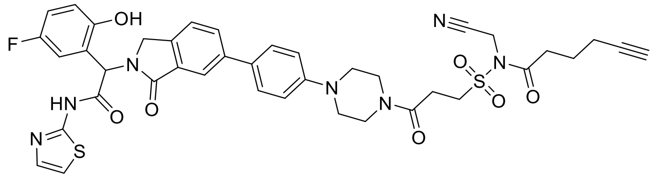

Chromatogram

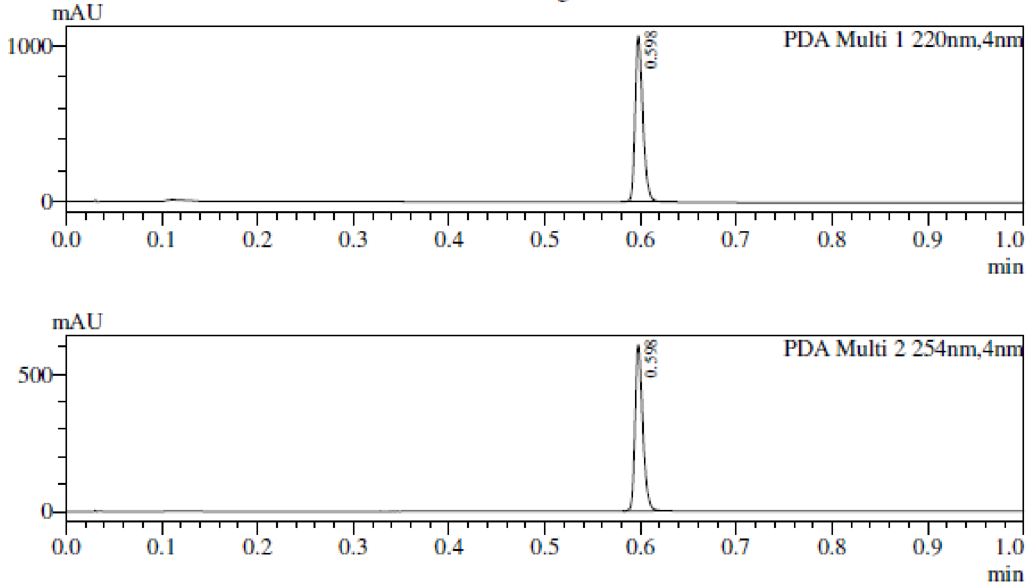

MS Chromatogram

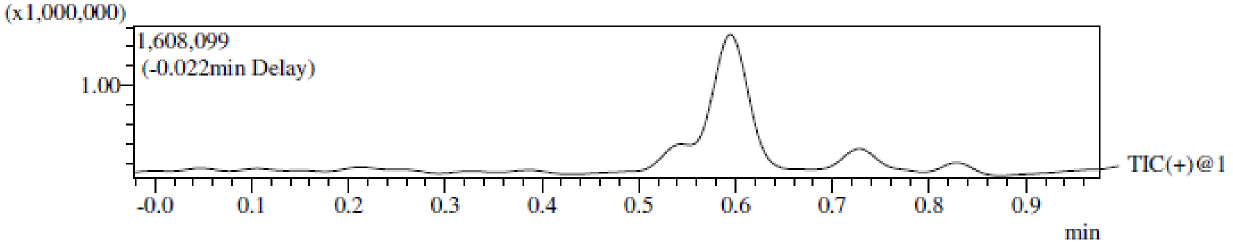

Mass Spectrum

RetTime: 0.597 DateFile: D:\Data\2022\2203\220304\EC6659-45-P1C.lcd

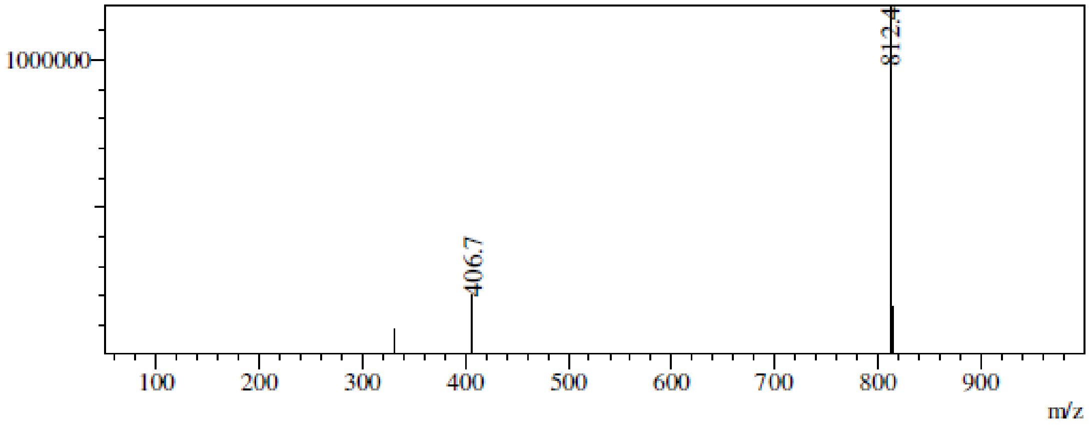

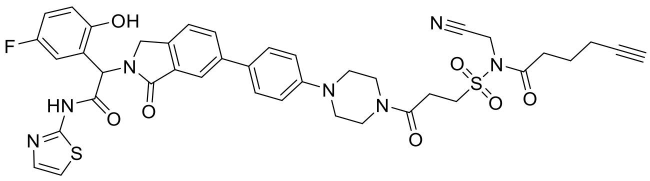

Chromatogram

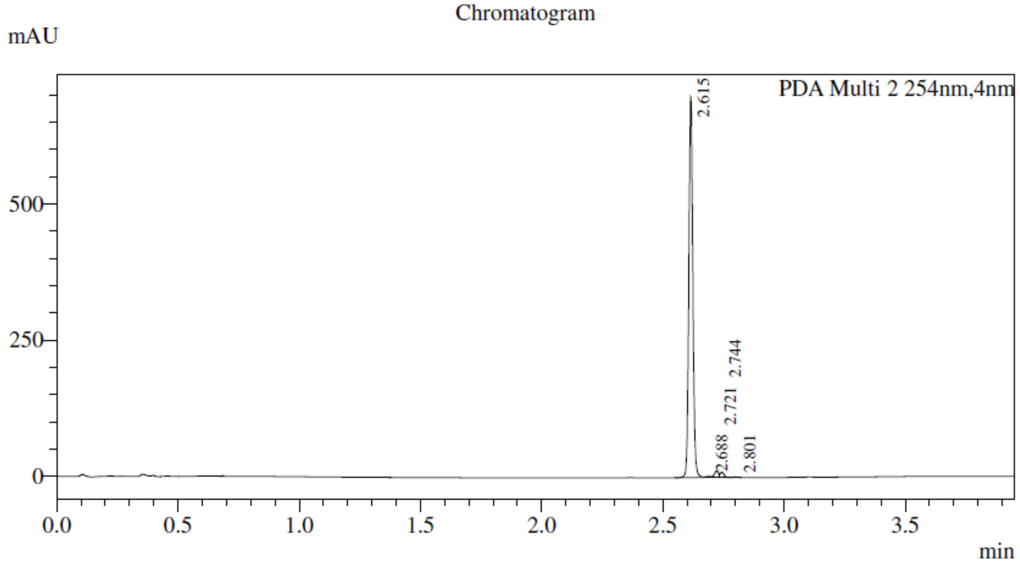

Integration Result

PDA Ch2 254nm

Peak#	Ret. Time	USP Width	Height	Height%	Area	Area%
1	2.615	0.030	700596	96.524	800951	96.451
2	2.688	0.038	2627	0.362	3330	0.401
3	2.721	0.036	11922	1.643	14189	1.709
4	2.744	0.039	9644	1.329	10789	1.299
5	2.801	0.030	1040	0.143	1165	0.140
Total			725829	100.000	830424	100.000

3.20.6 Compound **17** (FPR SuFA probe)

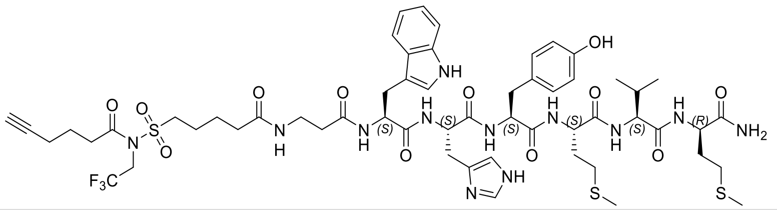

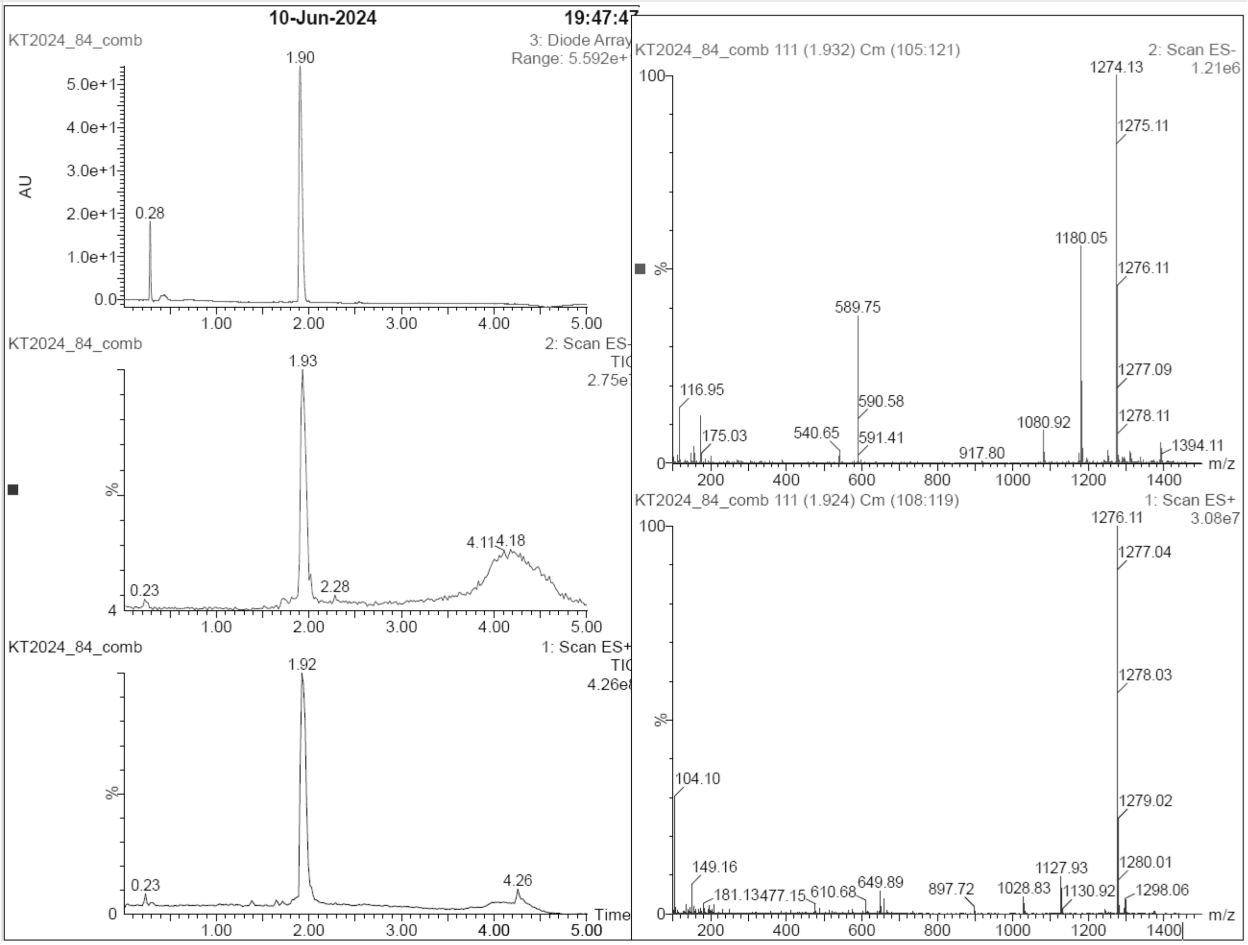

3.20.7 Compound **19** (GLP1R SuFA probe)

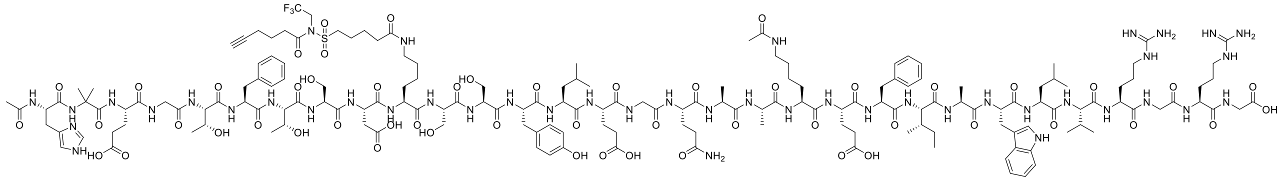

UPLC-MS

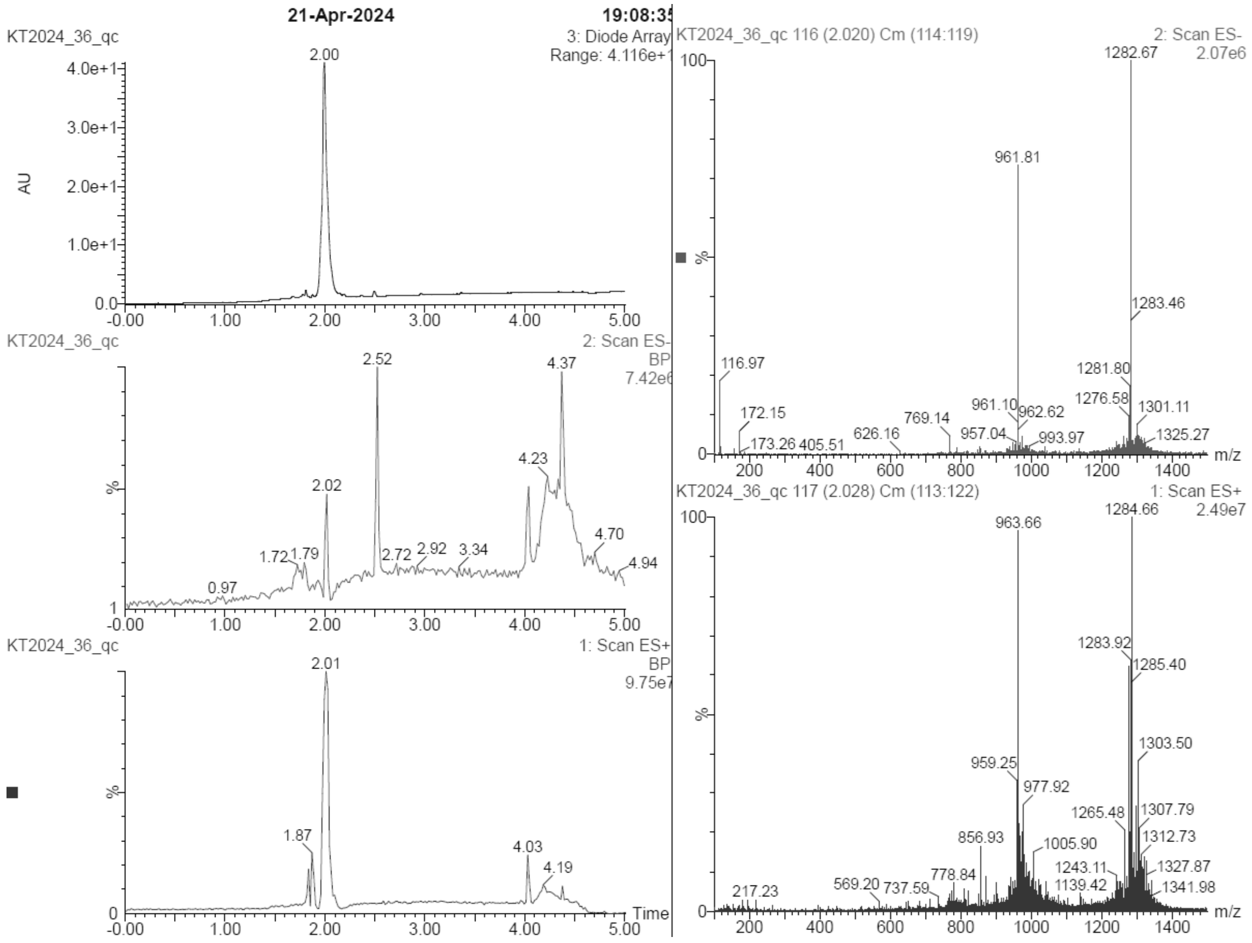

3.20.8 Compound **23** (BTK SuFA probe)

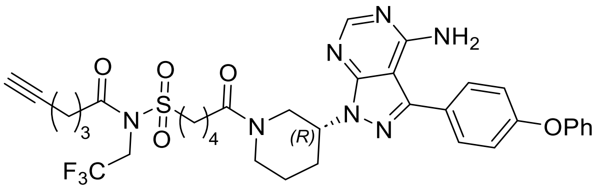

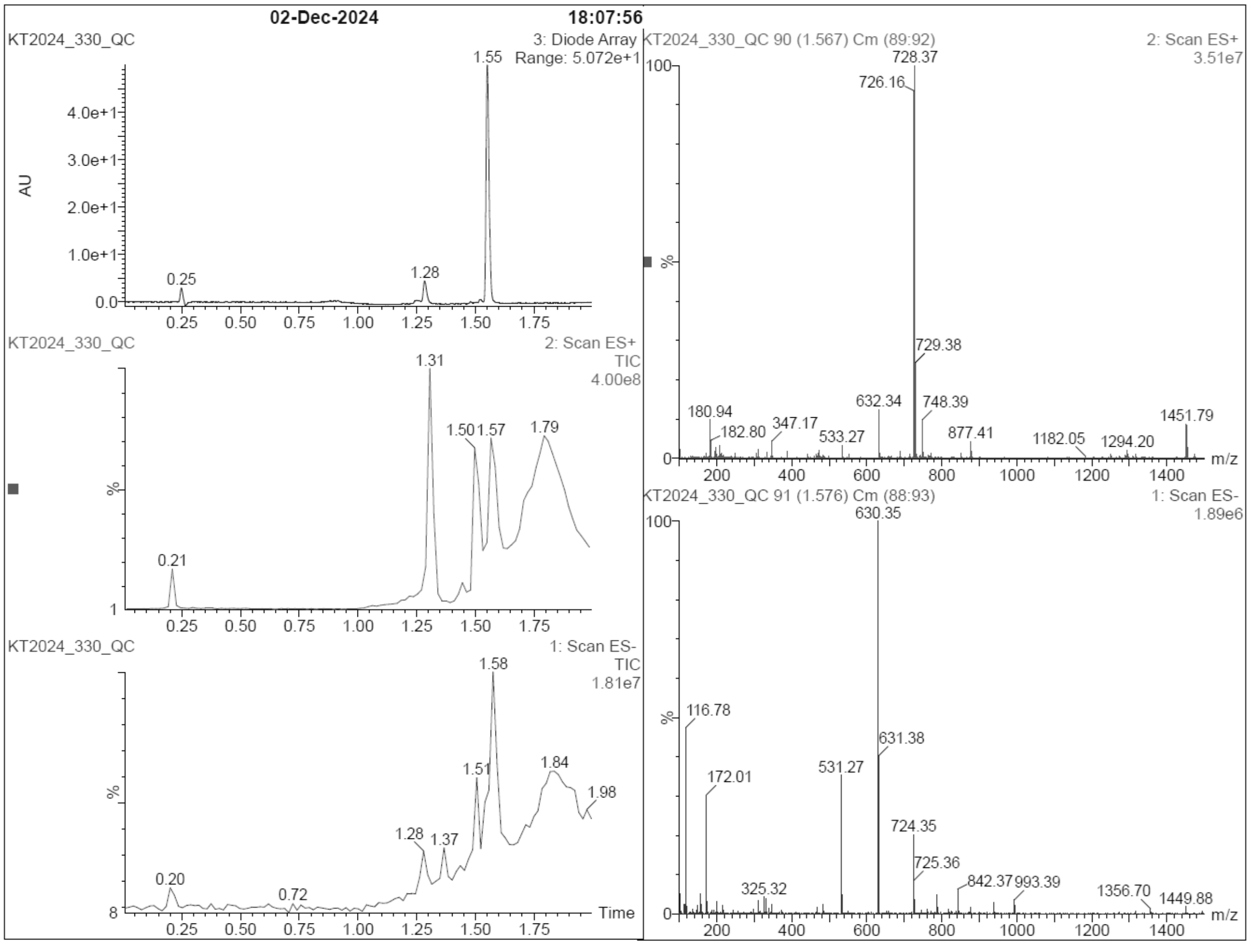

3.20.9 <u>Compound **26** (VEGFR2 SuFA probe)</u>

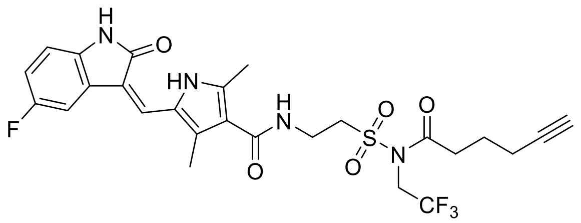

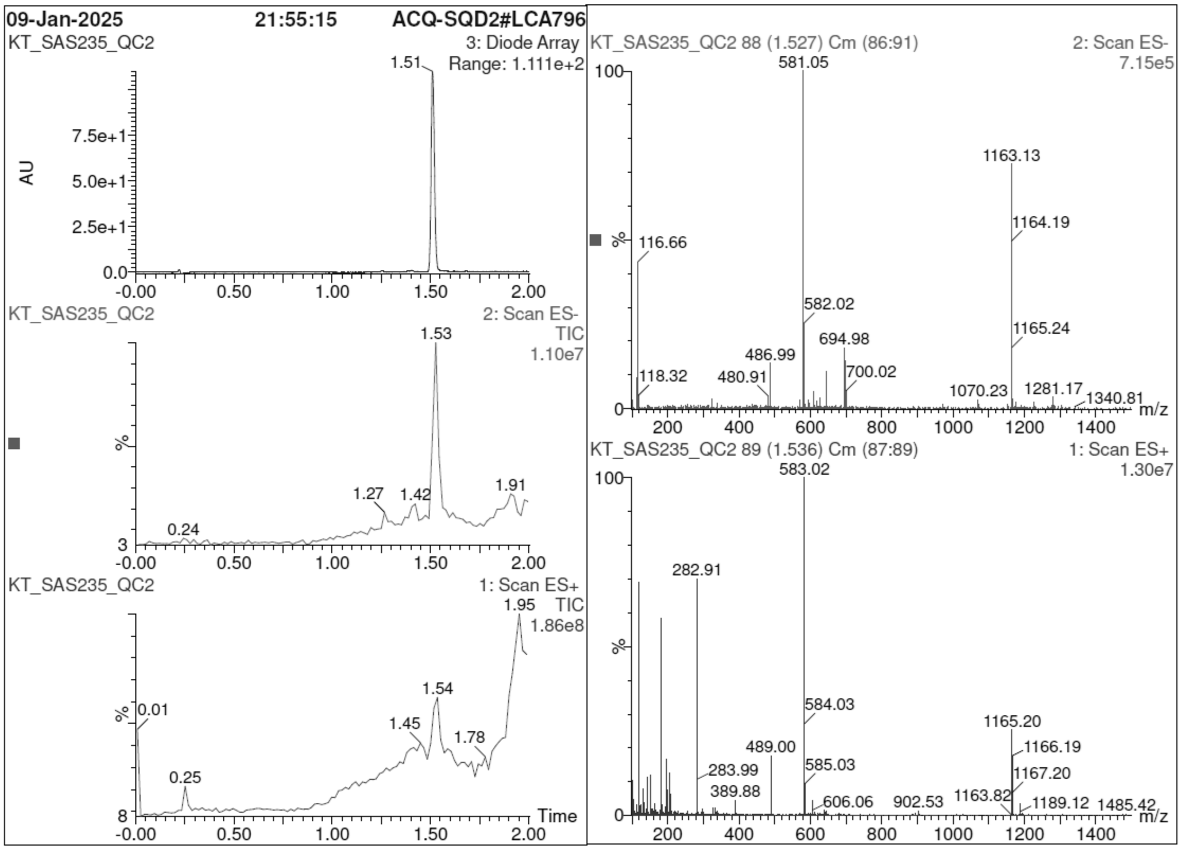

3.20.10 Compound **27** (Halo-PSMA-GRC)

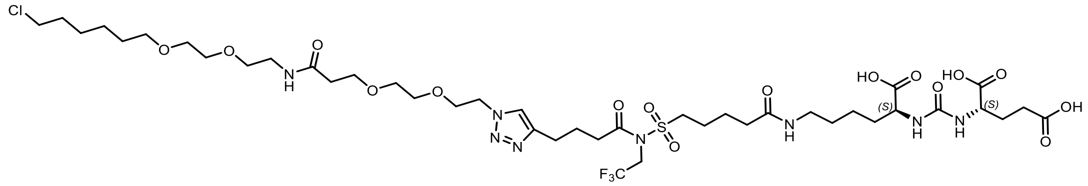

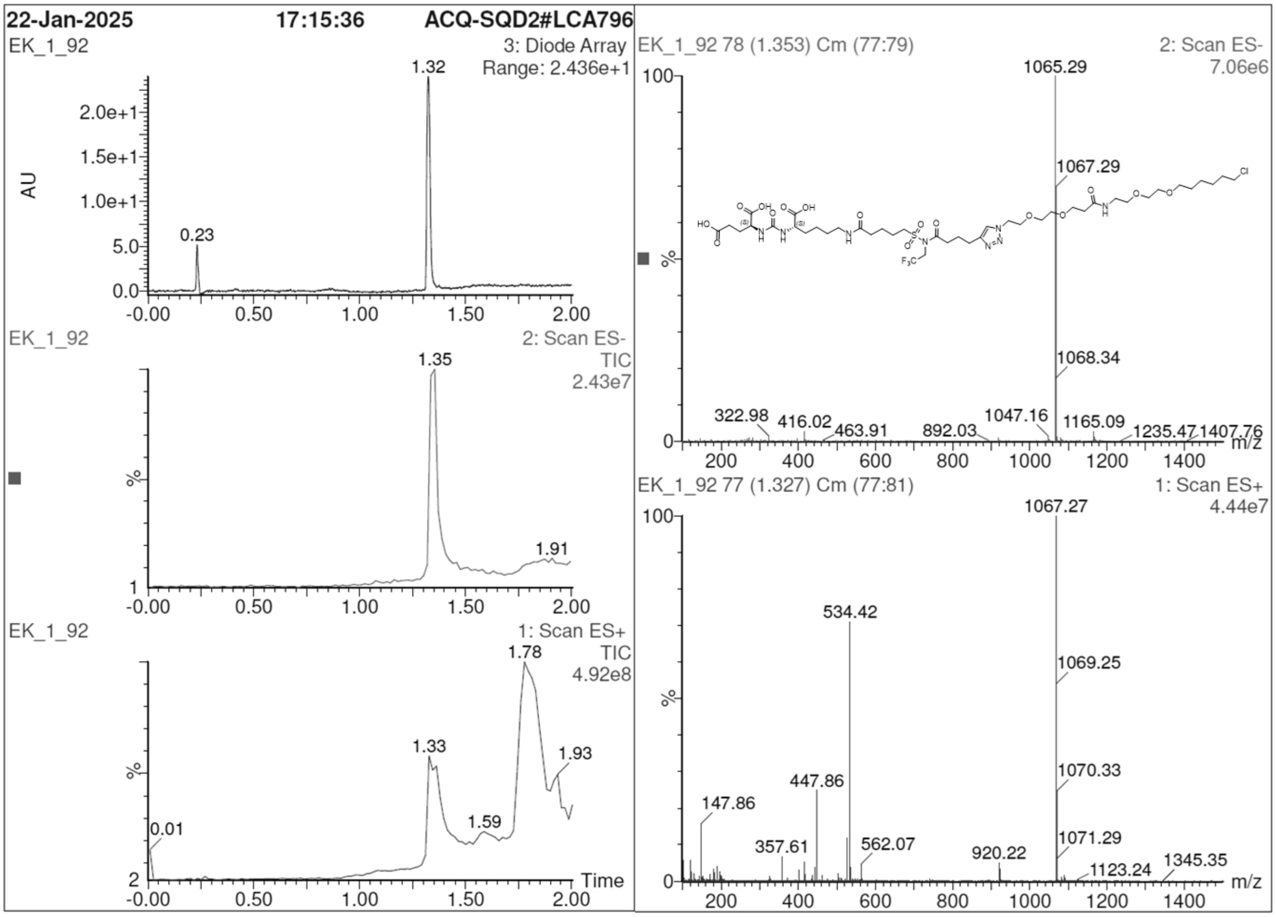

3.20.11 <u>Compound **28** (Halo-PSMA-iGRC)</u>

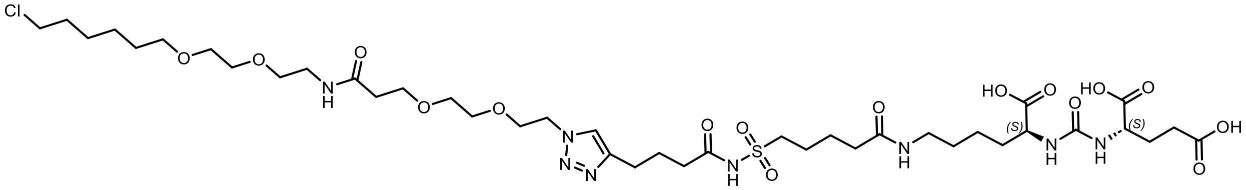

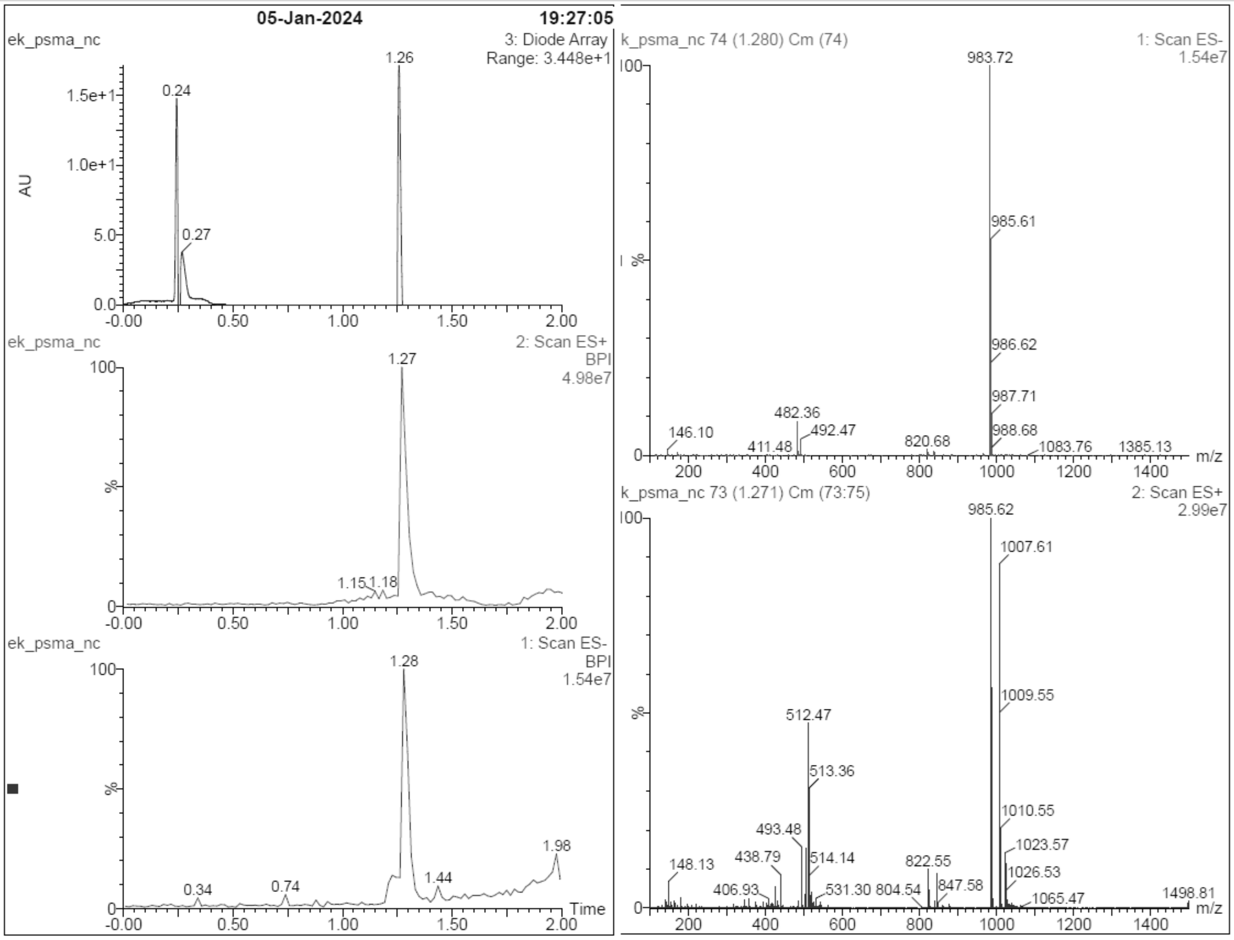

3.20.12 Compound **29** (FKBP-PSMA-GRC)

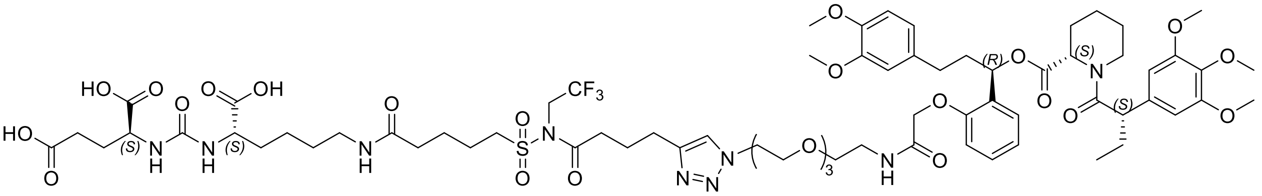

UPLC-MS

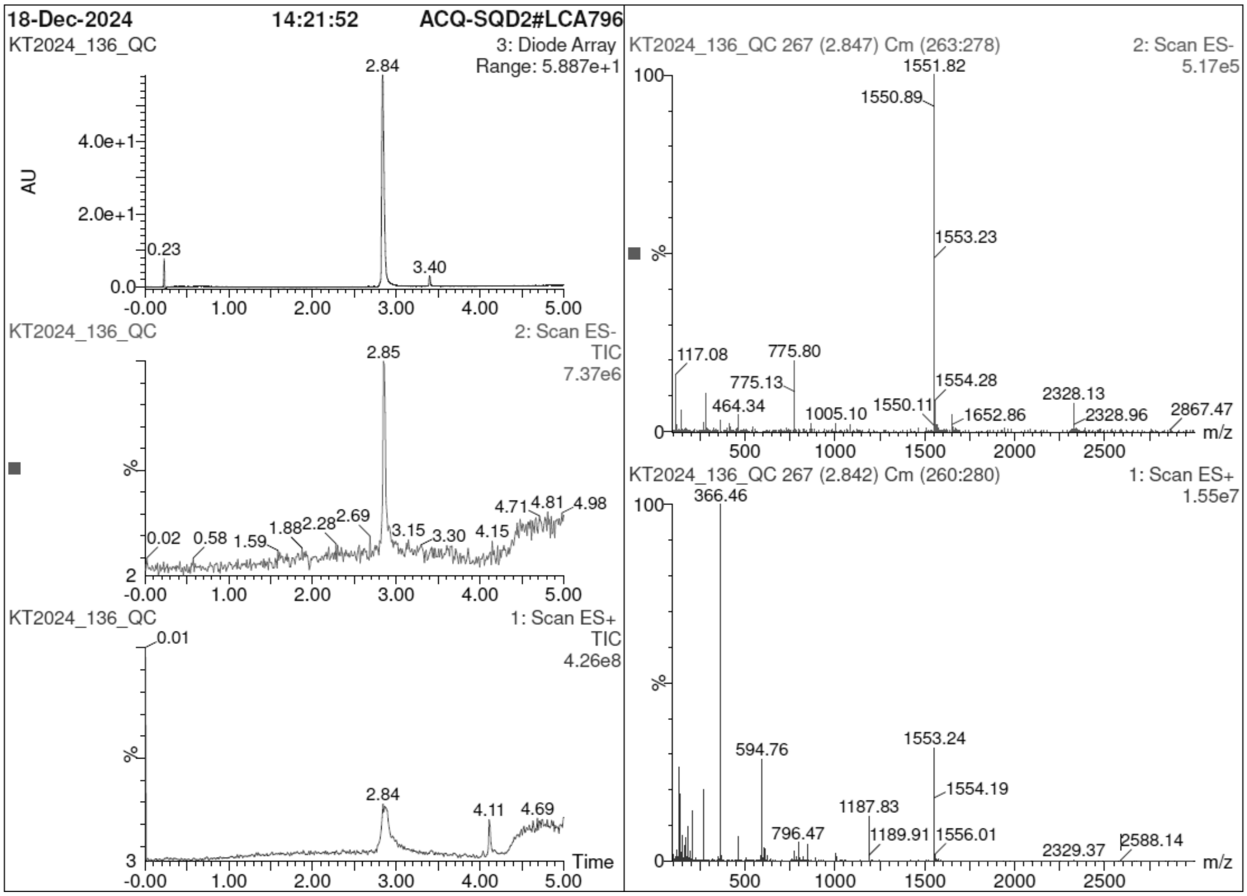

3.20.13 Compound **30** (FKBP-PSMA-iGRC)

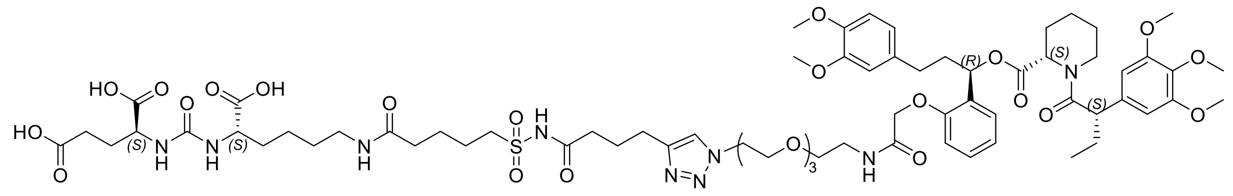

UPLC-MS

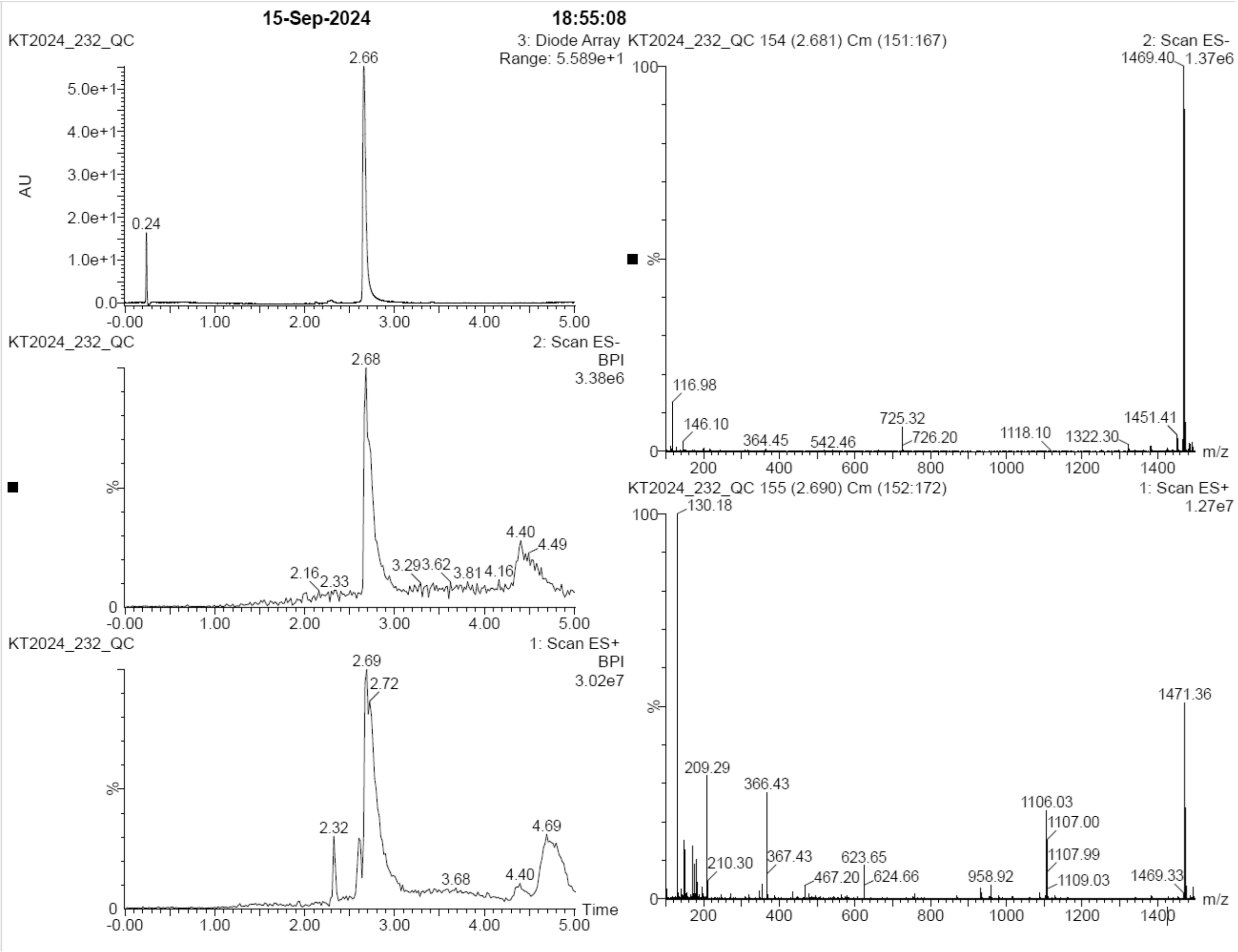

3.20.14 Compound **31** (Halo-BTK-GRC)

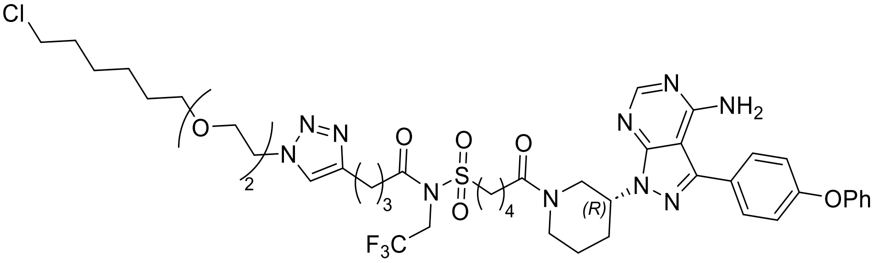

UPLC-MS

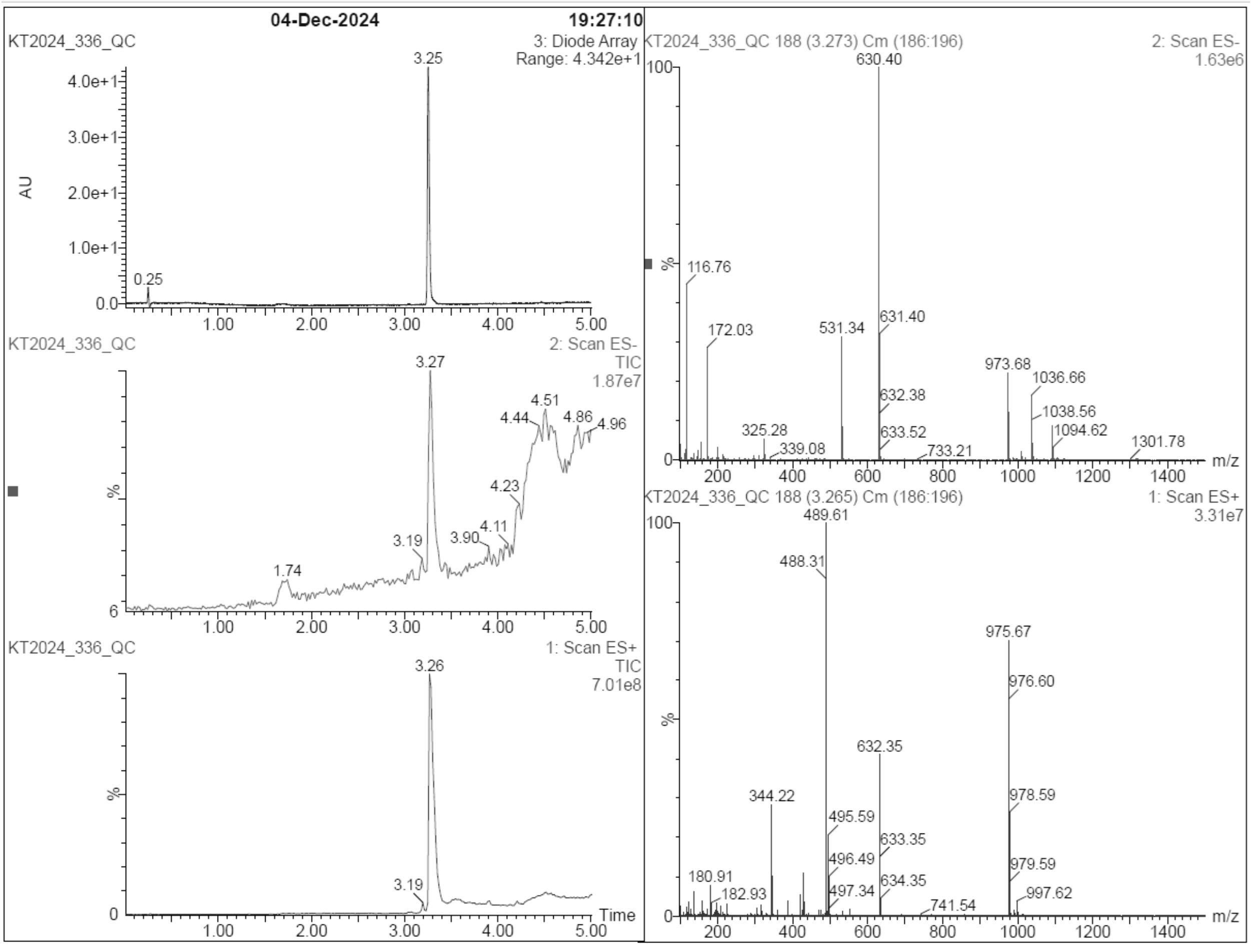

3.20.15 Compound **32** (Halo-BTK-iGRC)

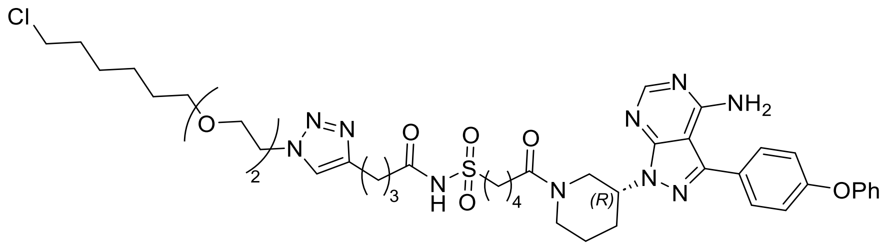

UPLC-MS

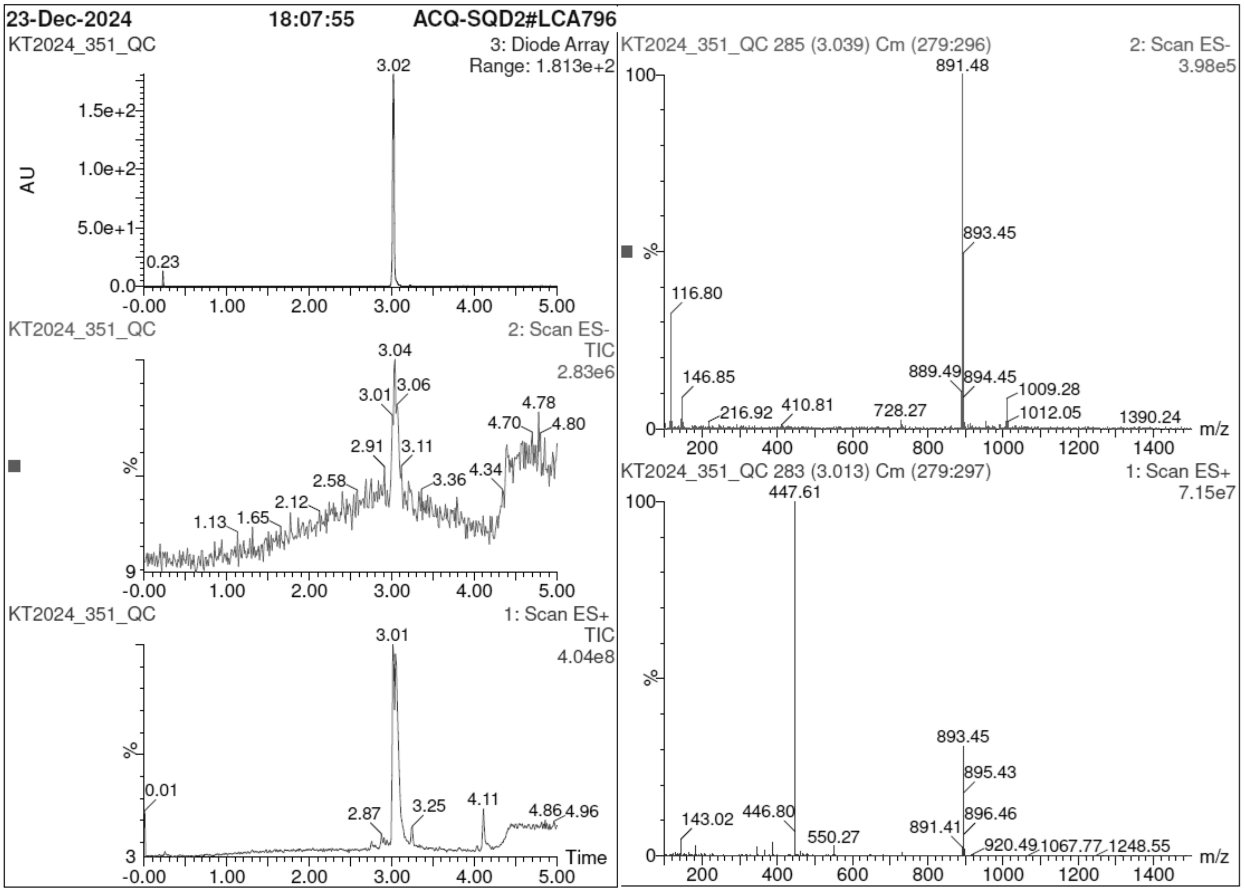

3.20.16 Compound **S1** (GLP1R SuFA probe)

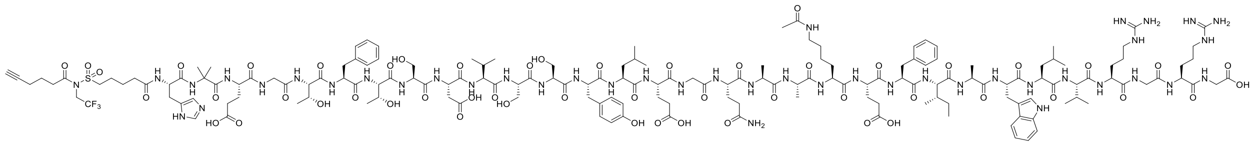

UPLC-MS

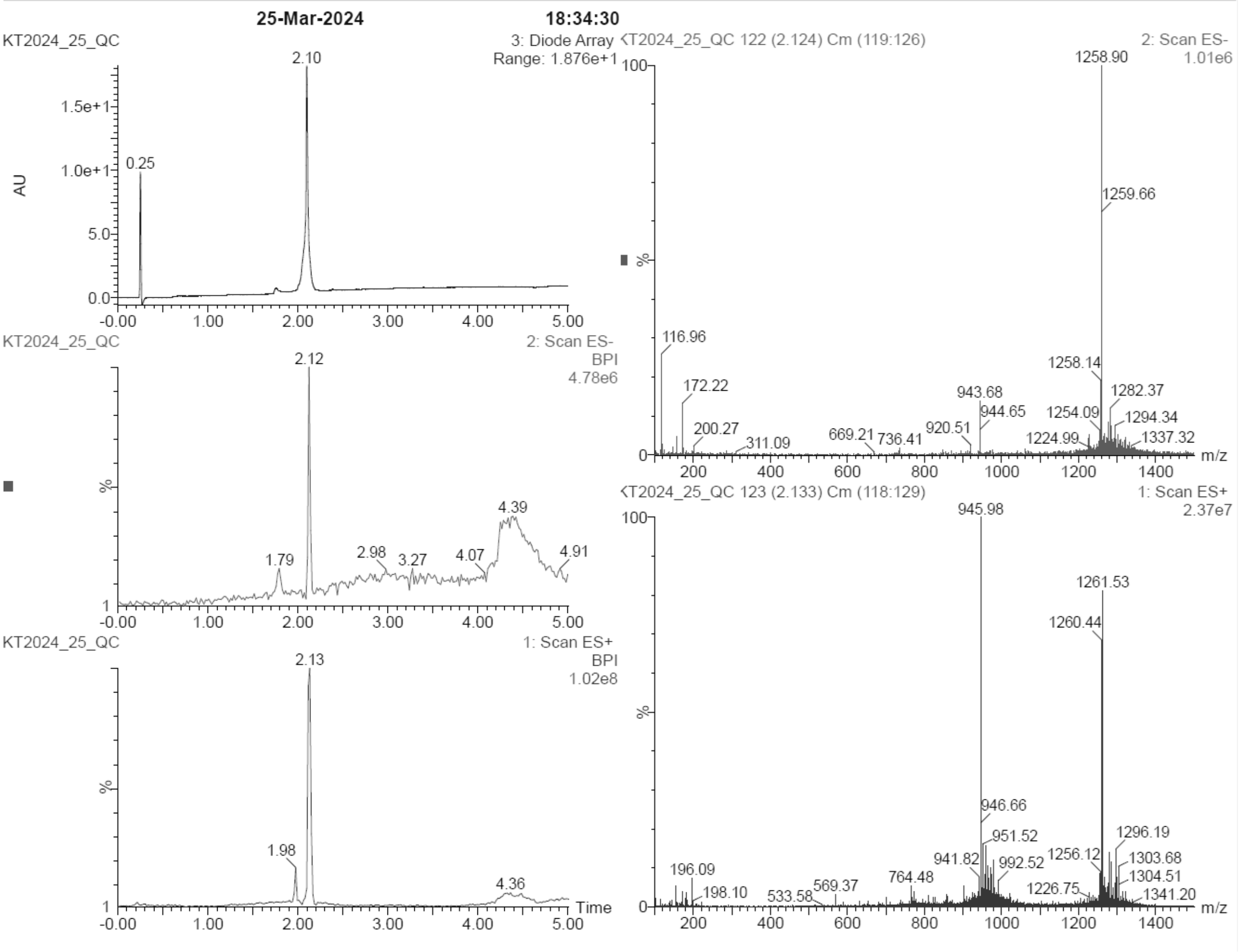

3.20.17 Compound **S2** (GLP1R SuFA probe)

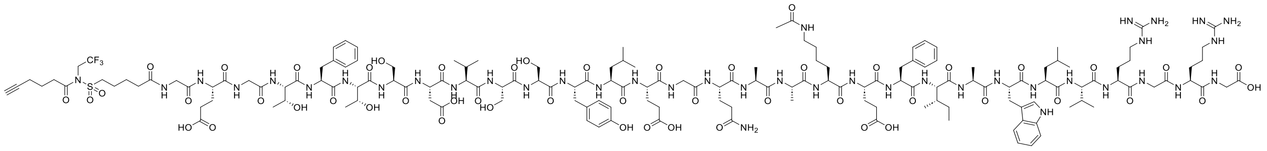

UPLC-MS

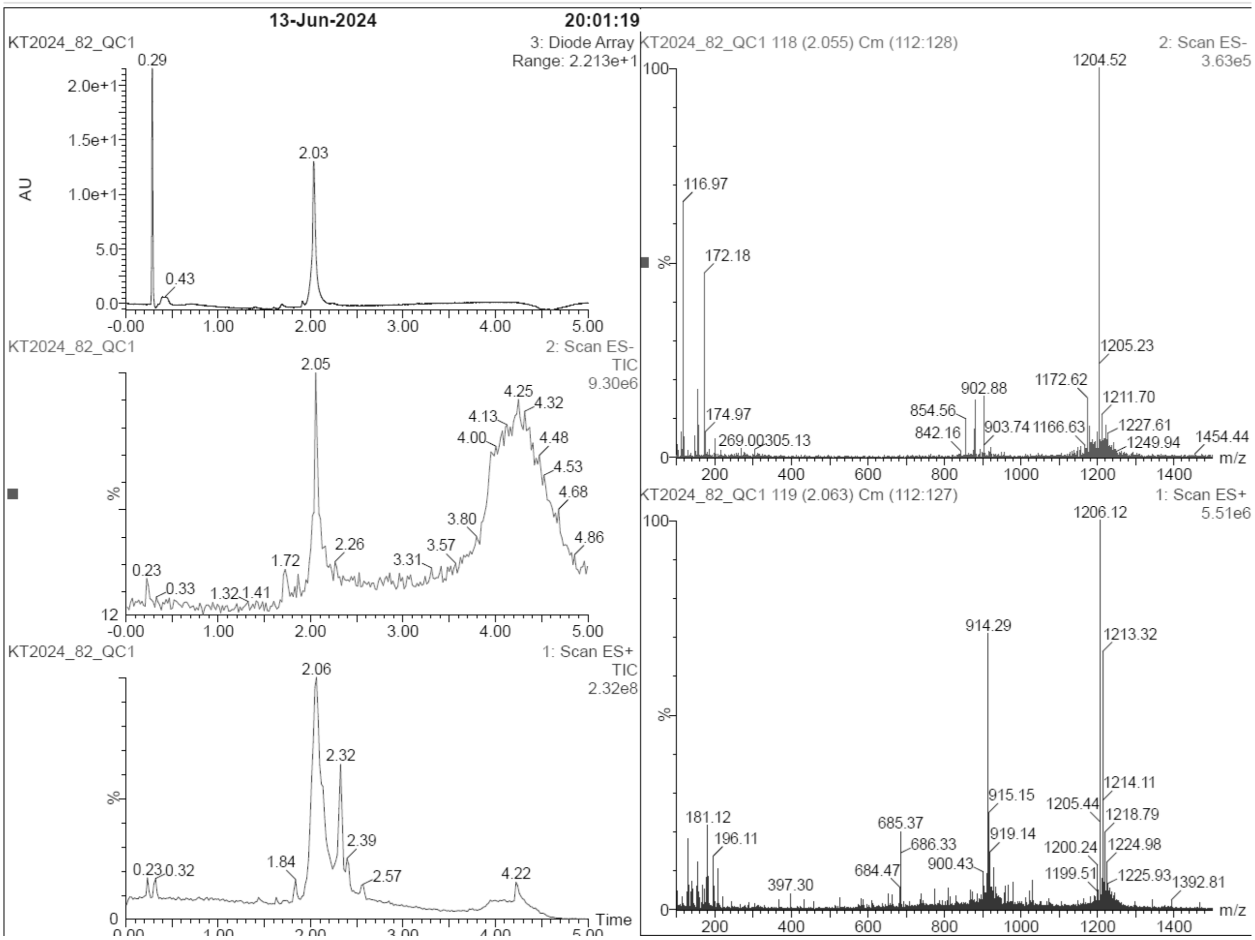

3.20.18 Compound **S3** (GLP1R SuFA probe)

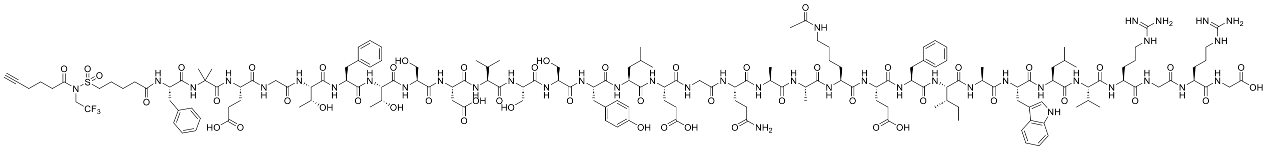

UPLC-MS

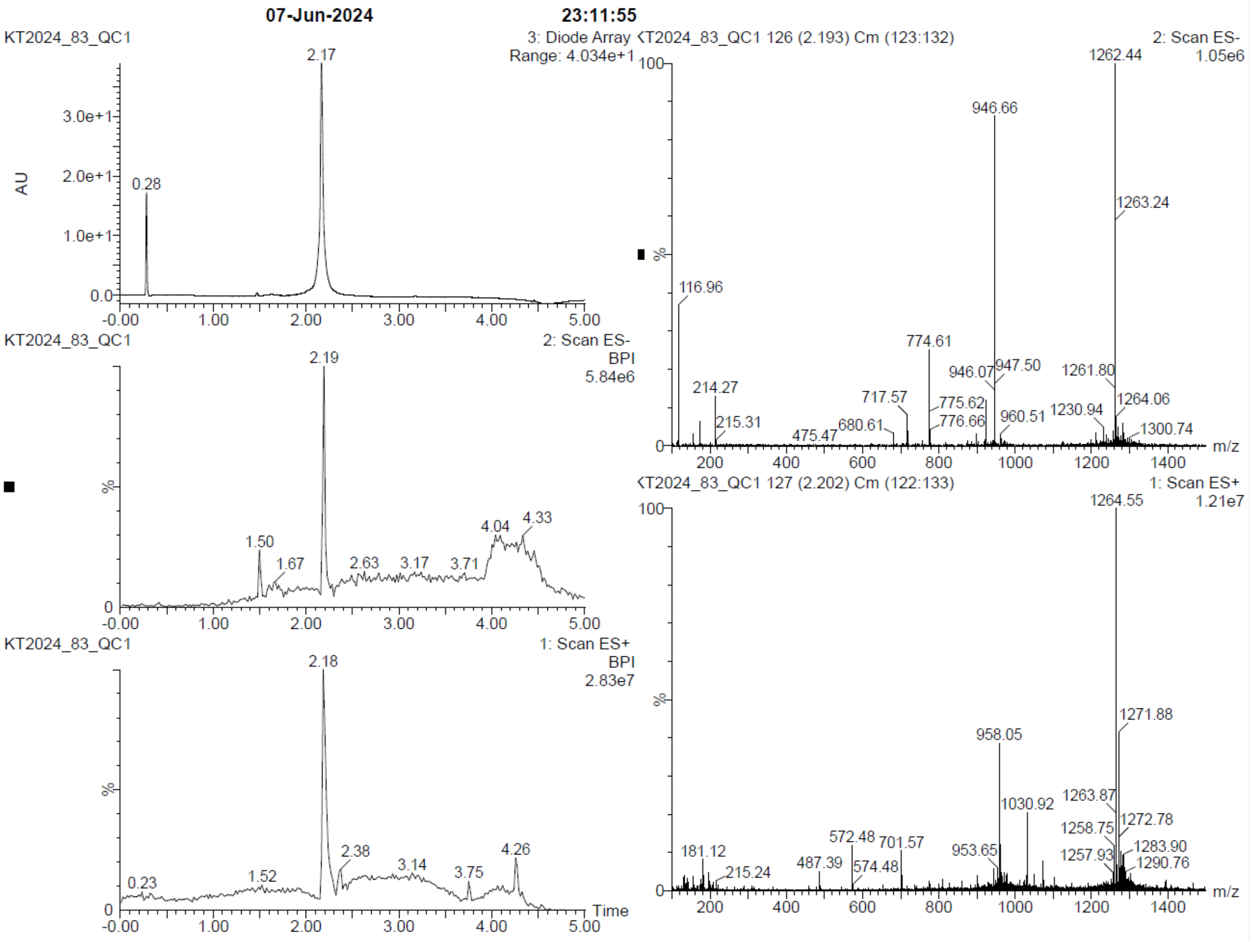

3.20.19 Compound **S4** (GLP1R SuFA probe)

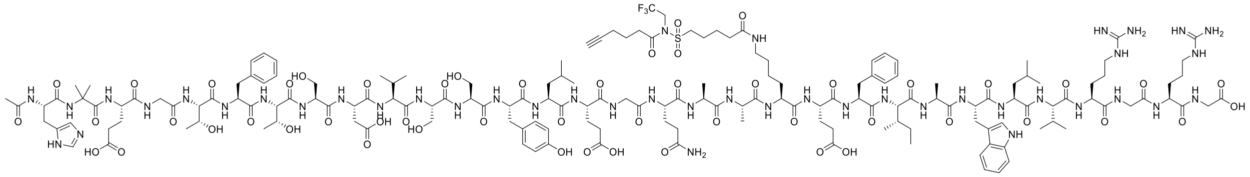

UPLC-MS

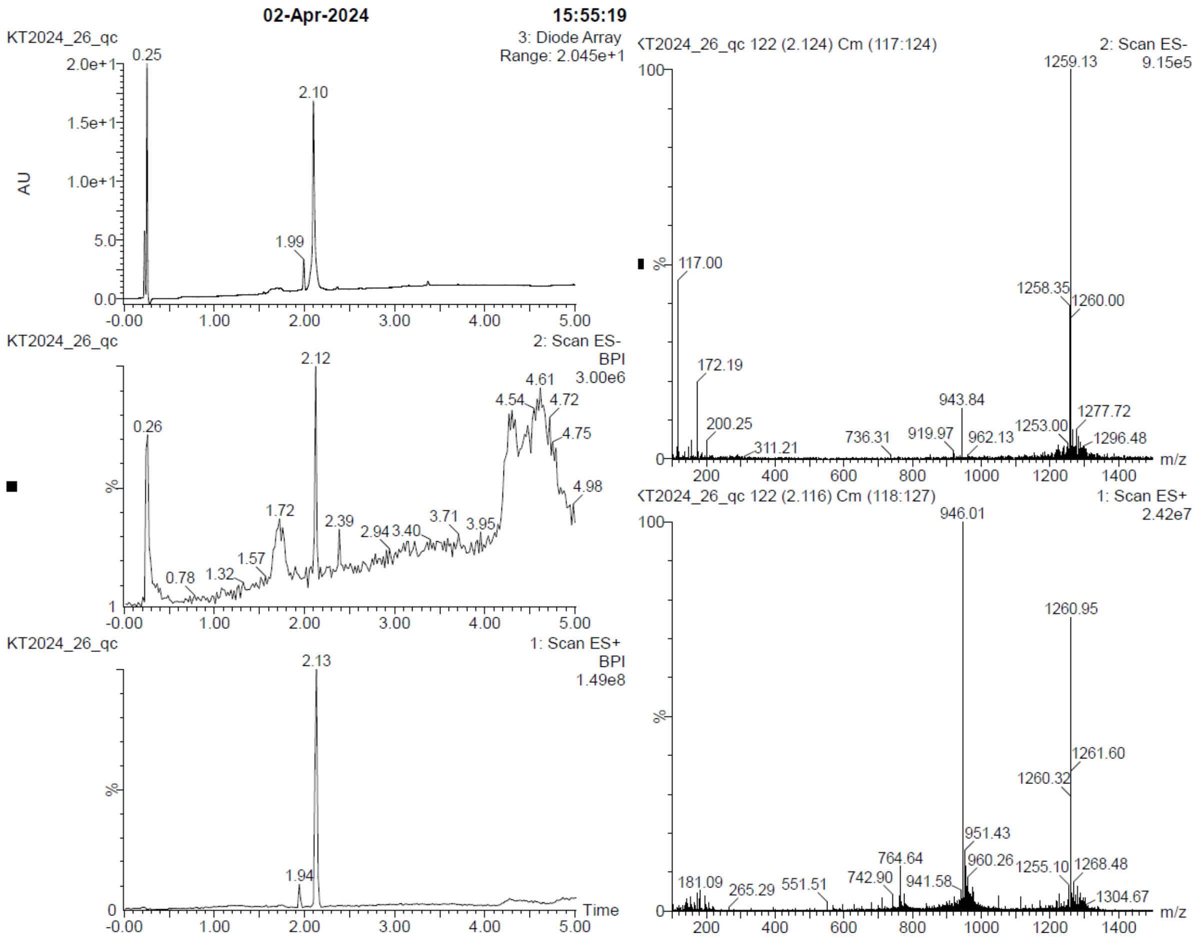

3.20.20 Compound **S5** (GLP1R SuFA probe)

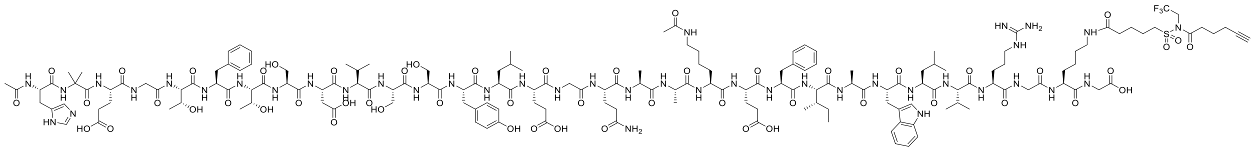

UPLC-MS

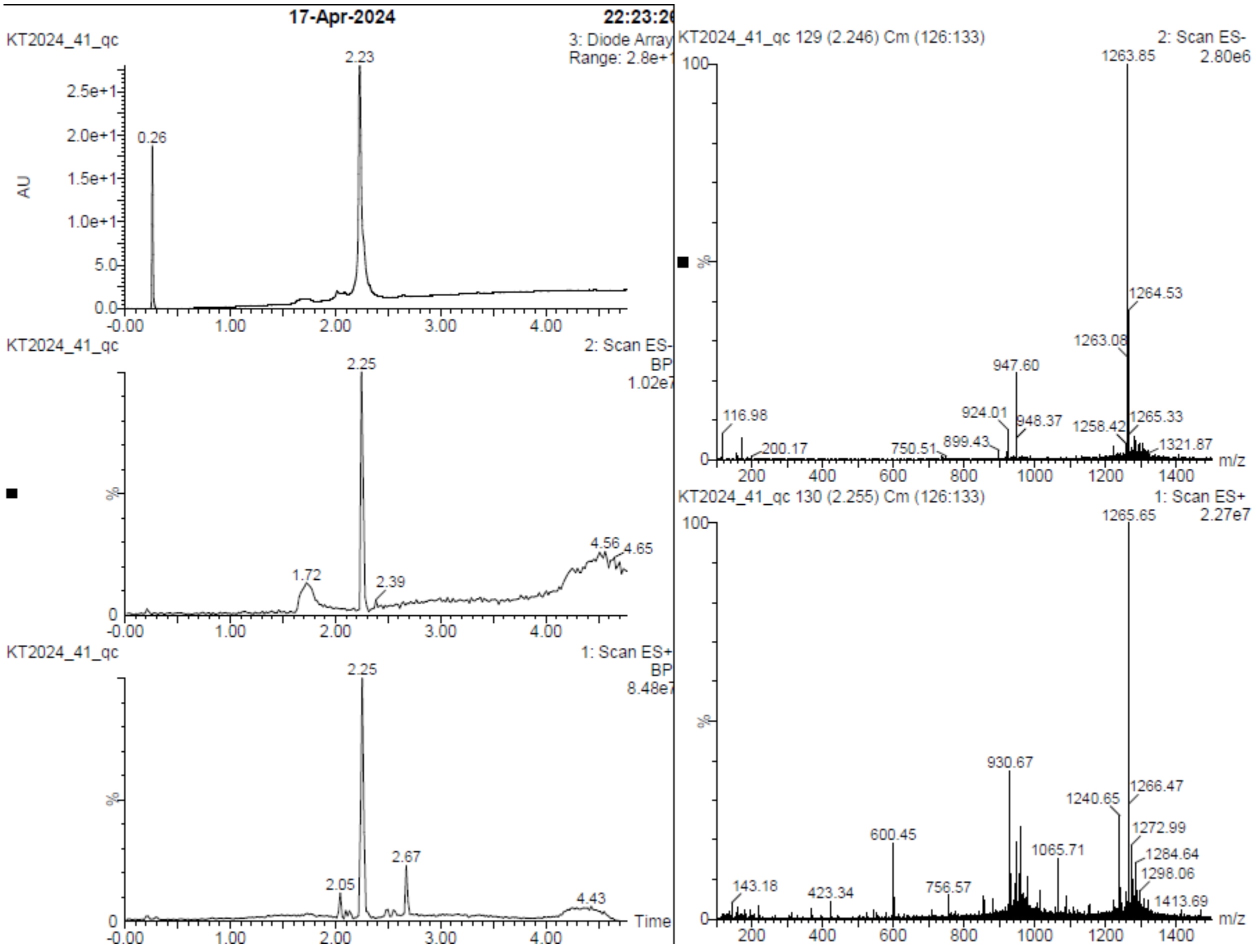

3.20.21 Compound **S6** (RIPK1 SuFA probe)

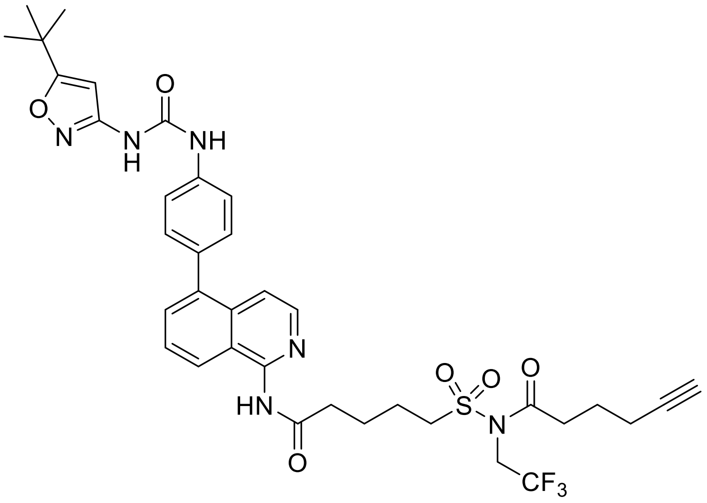

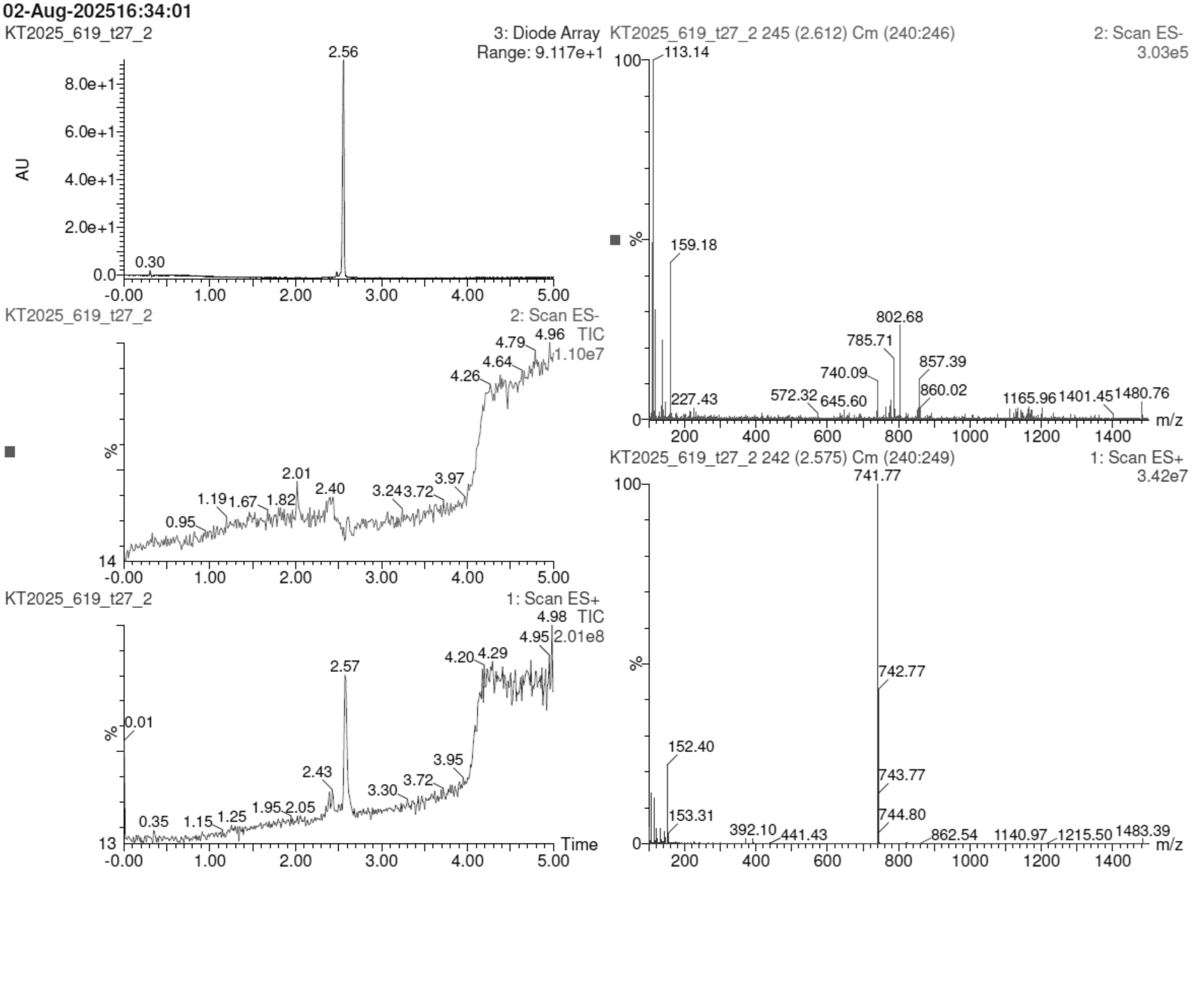

3.20.22 Compound **S7** (VEGFR2 NASA probe)

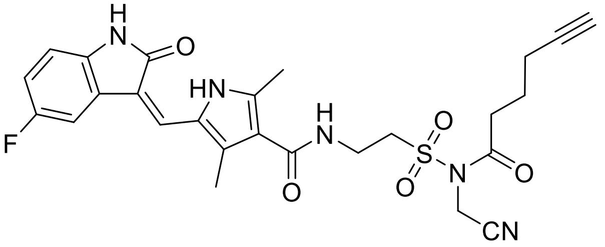

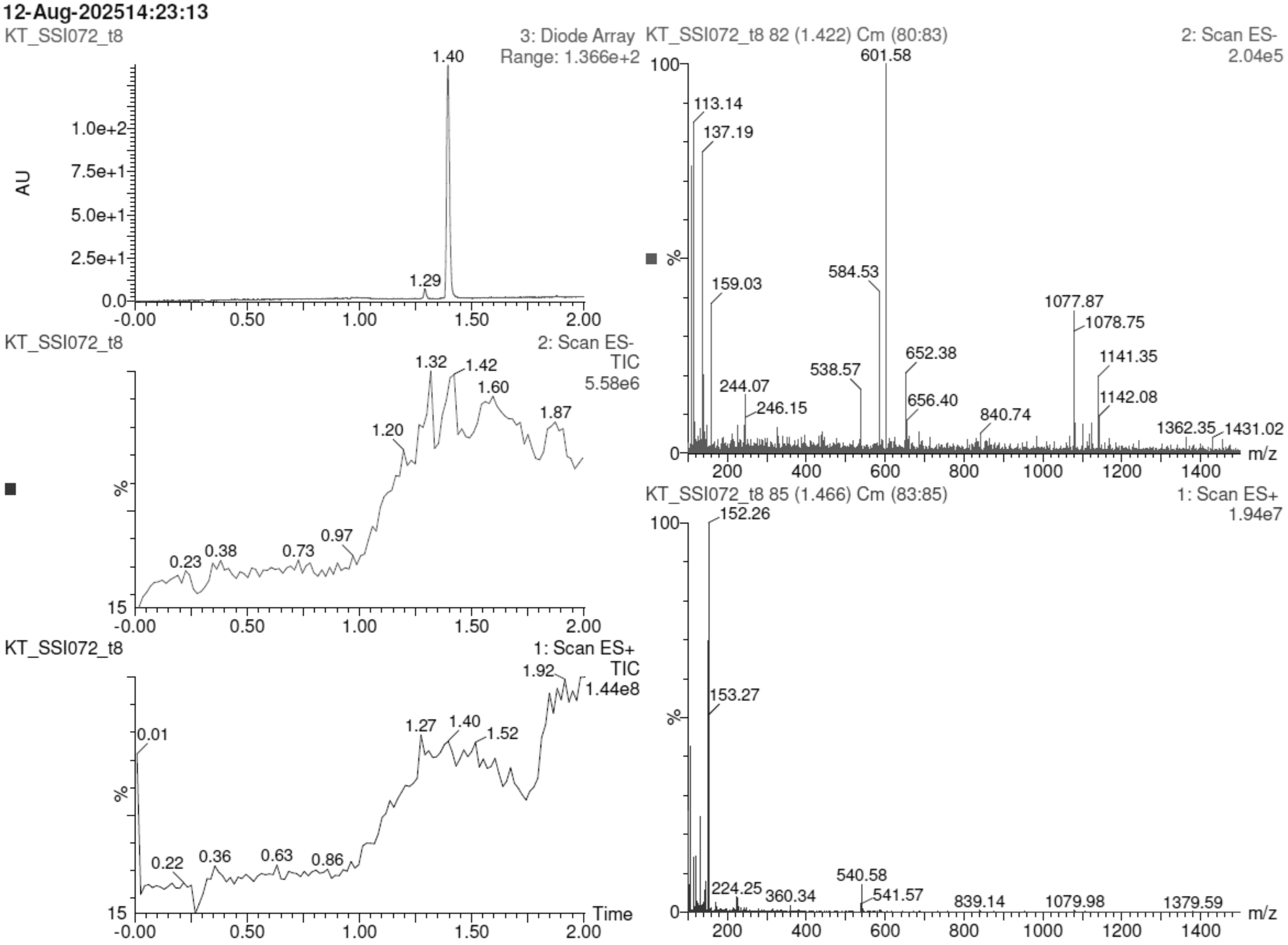

3.21 HRMS spectra of ligands in manuscript

3.21.1 <u>Compound **17** (FPR SuFA probe)</u>

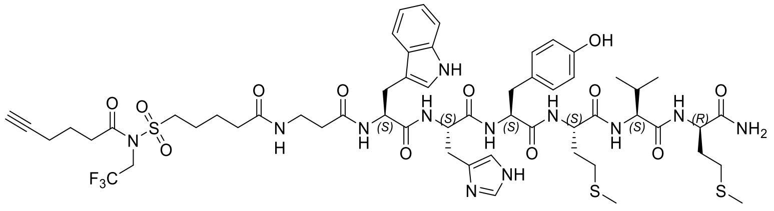

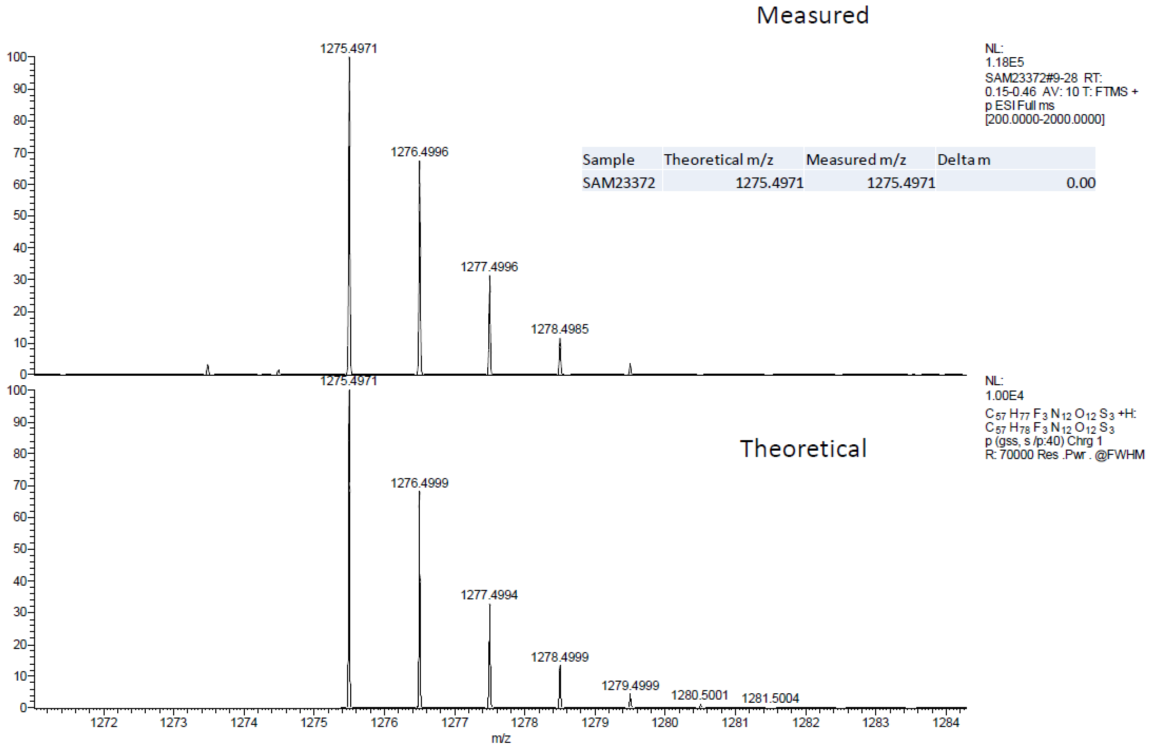

Sample	Theoretical m/z	Measured m/z	Delta m
SAM23372	1275.4971	1275.4971	0.00

3.21.2 Compound **19** (GLP1R SuFA probe)

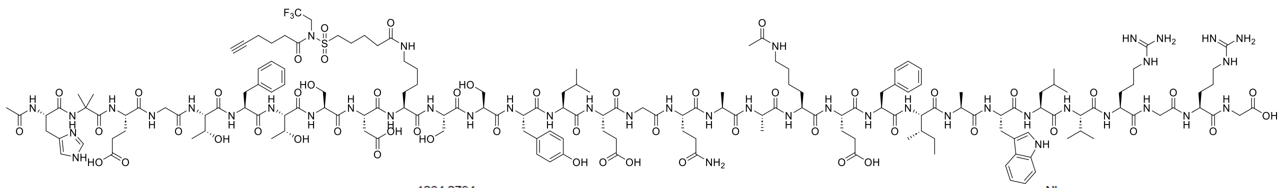

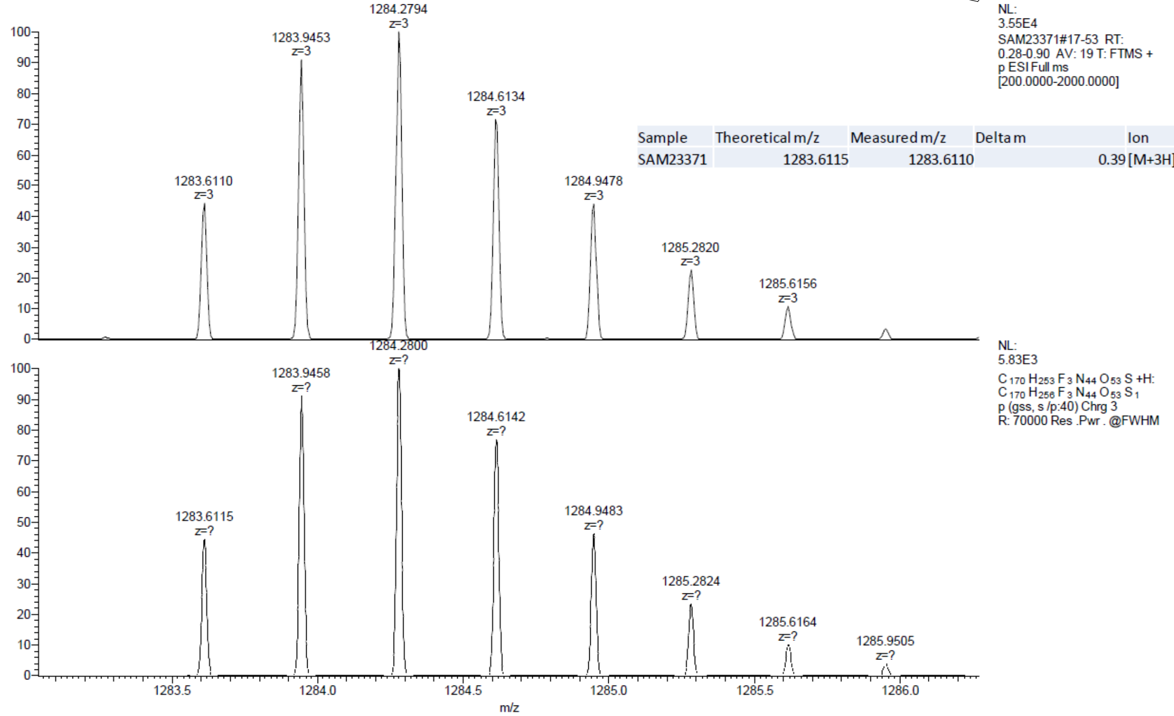

Sample	Theoretical m/z	Measured m/z	Delta m	Ion
SAM23371	1283.6115	1283.6110	0.39	[M+3H]3+

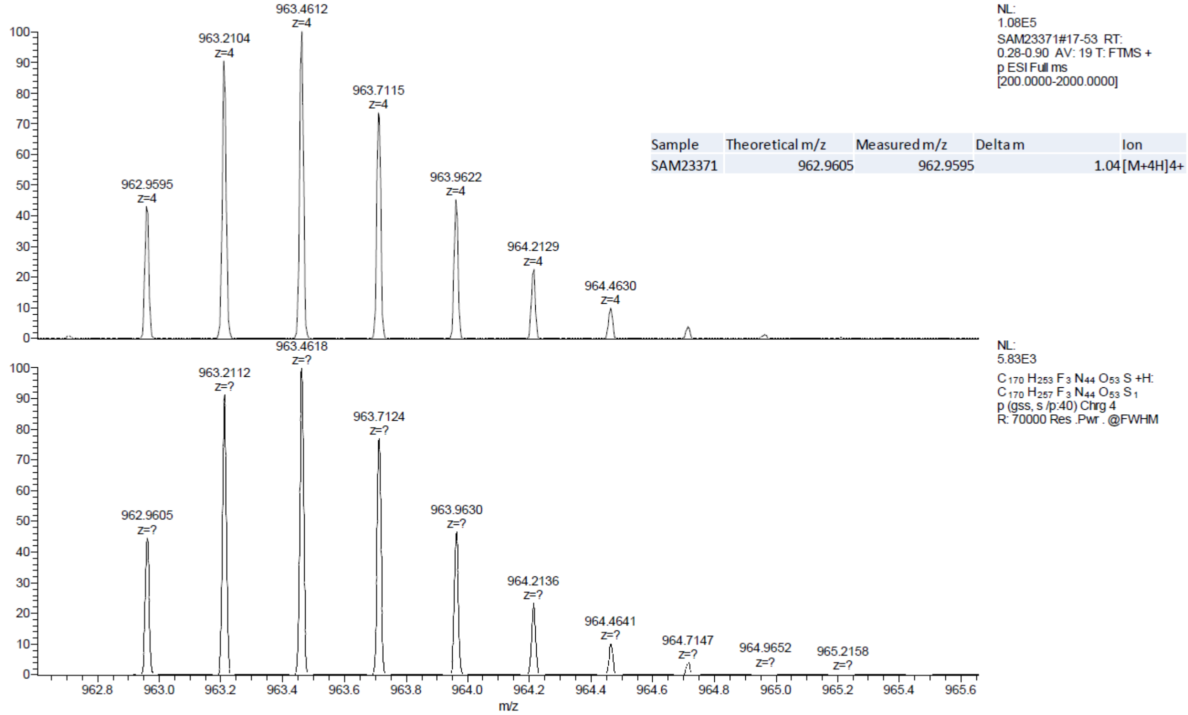

Sample	Theoretical m/z	Measured m/z	Delta m	Ion
SAM23371	962.9605	962.9595	1.04	[M+4H]4+

3.21.3 Compound **21** (PSMA SuFA probe)

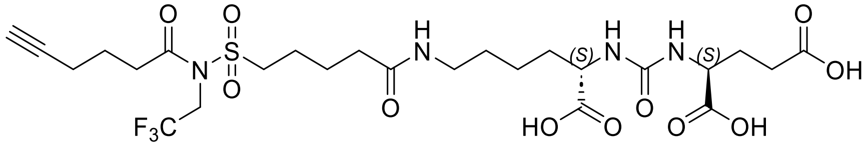

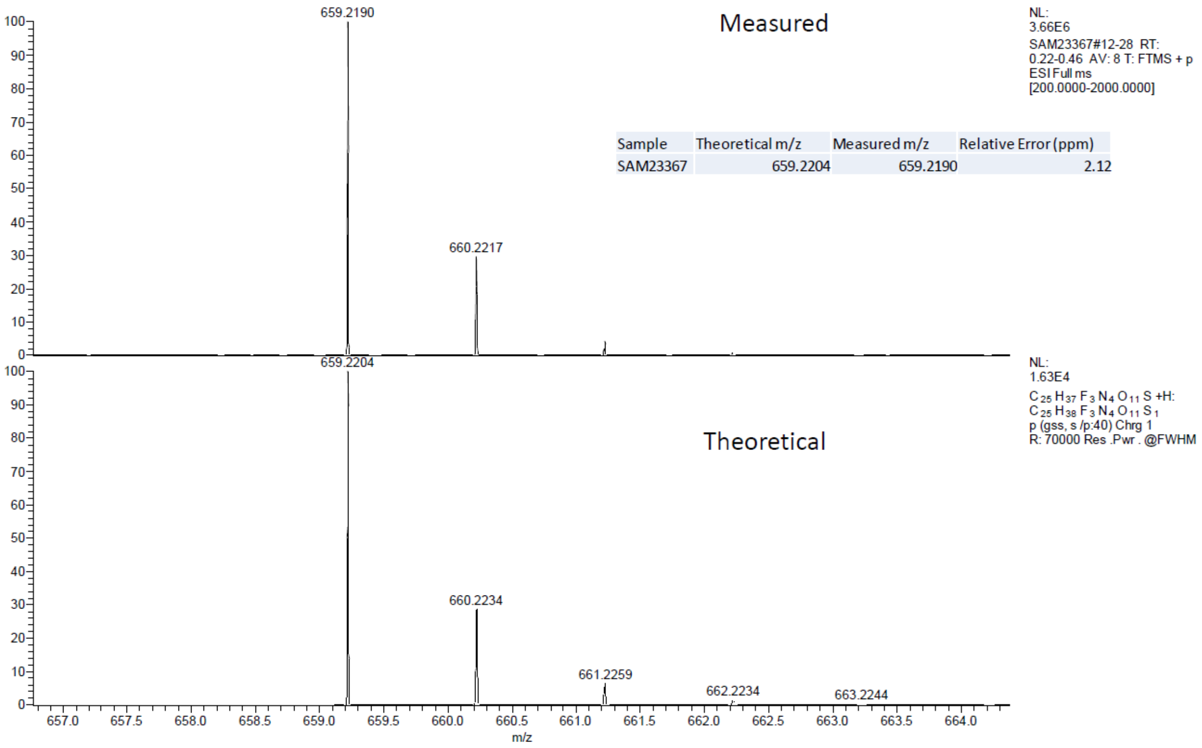

3.21.4 Compound **23** (BTK SuFA probe)

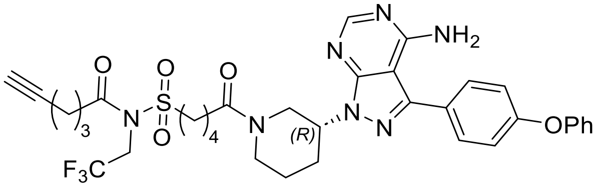

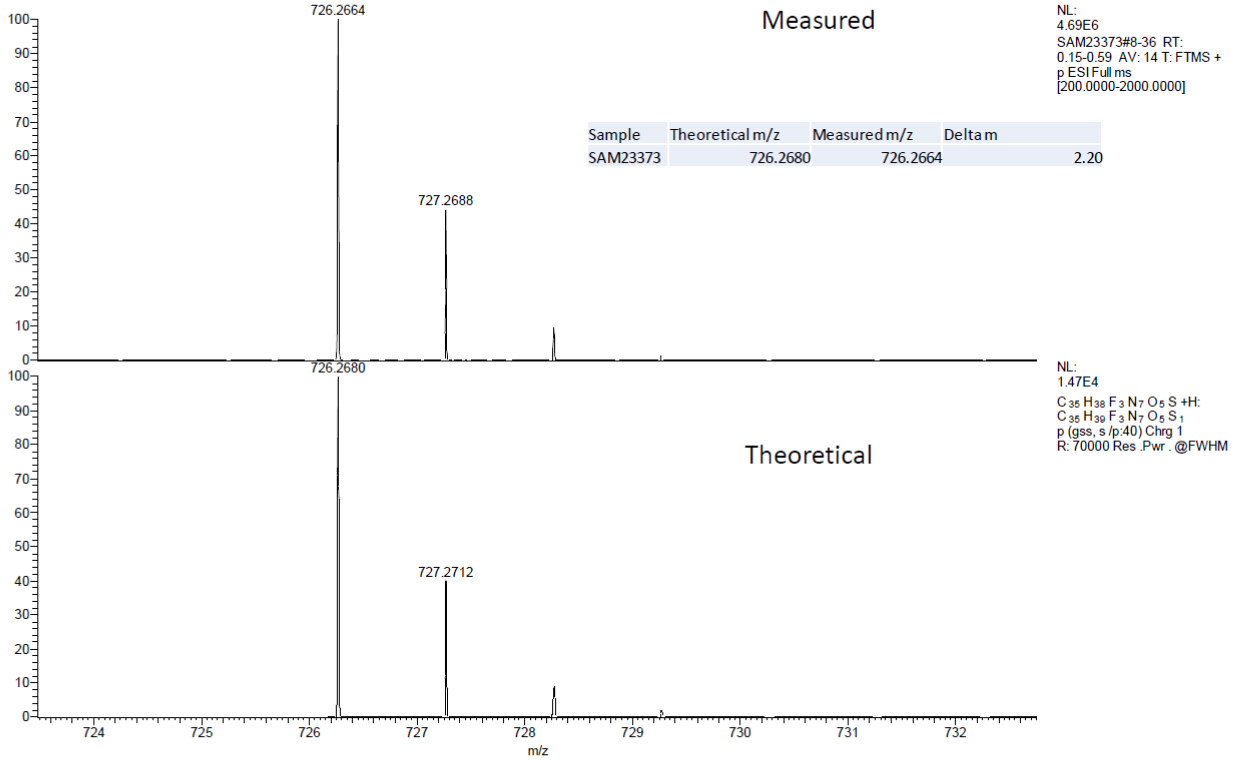

Sample	Theoretical m/z	Measured m/z	Delta m
SAM23373	726.2680	726.2664	2.20

3.21.5 Compound **26** (VEGFR2 SuFA probe)

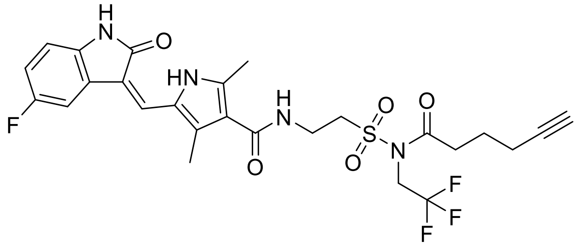

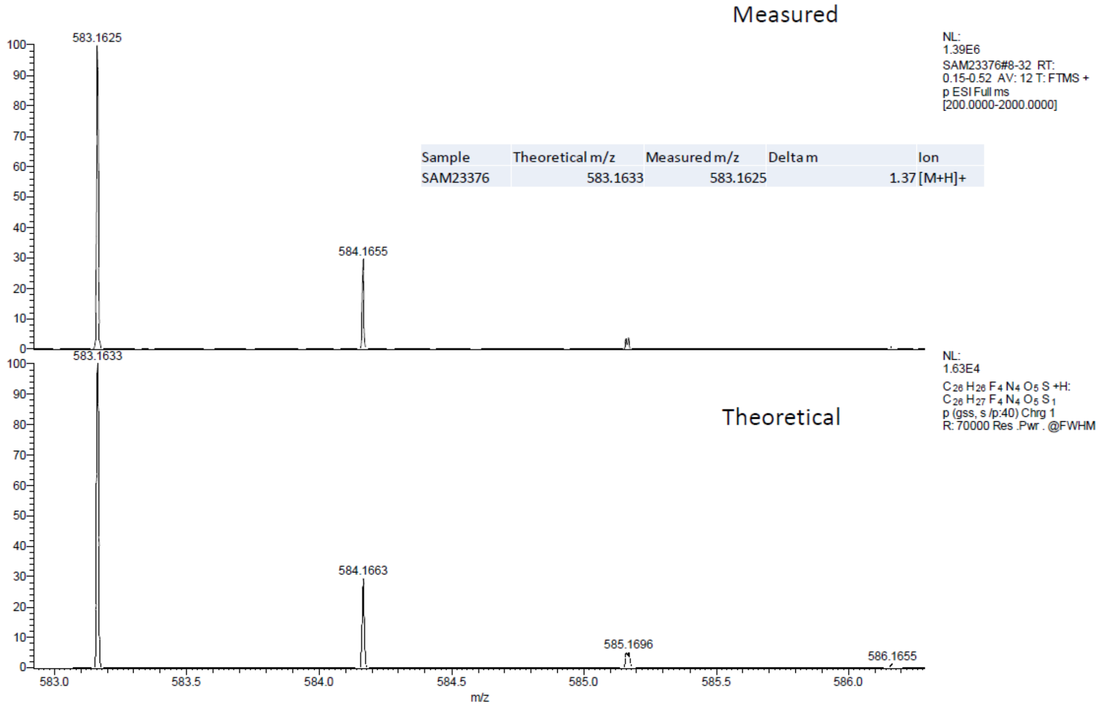

Sample	Theoretical m/z	Measured m/z	Delta m	Ion
SAM23376	583.1633	583.1625	1.37	[M+H]+

3.21.6 Compound **27** (Halo-PSMA-GRC)

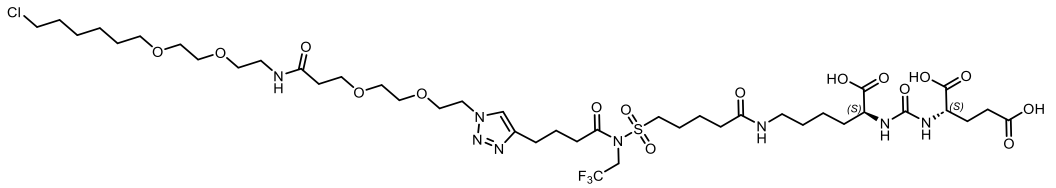

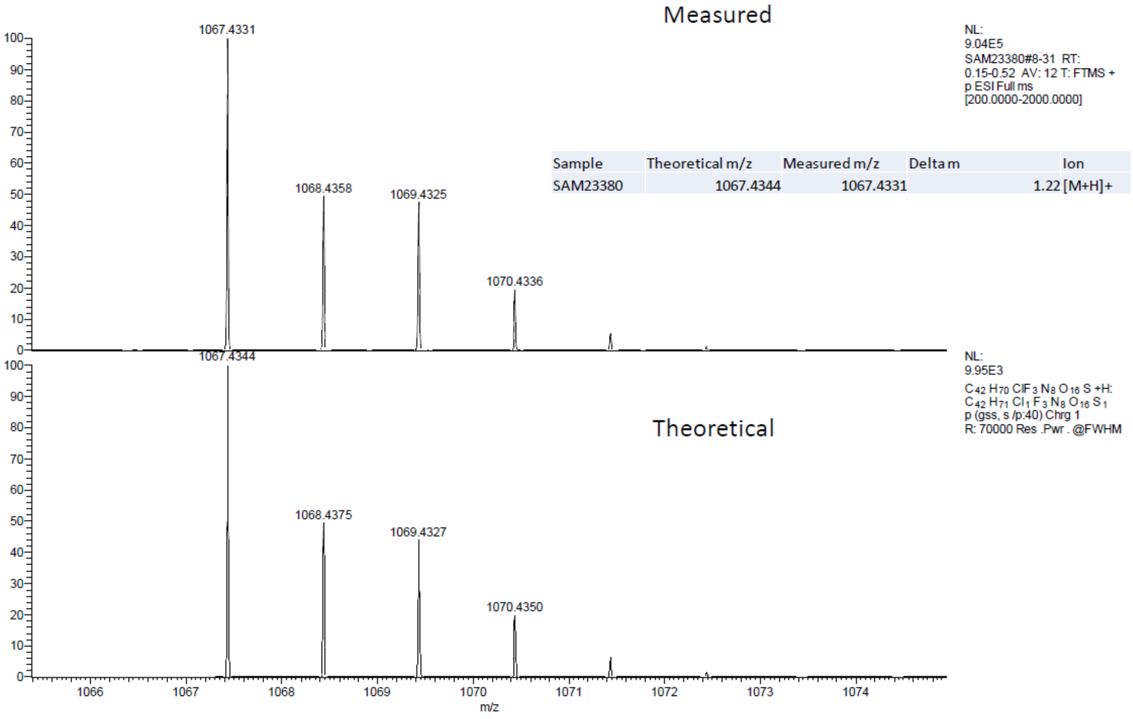

Sample	Theoretical m/z	Measured m/z	Delta m	Ion
SAM23380	1067.4344	1067.4331	1.22	[M+H]+

3.21.7 Compound **28** (Halo-PSMA-iGRC)

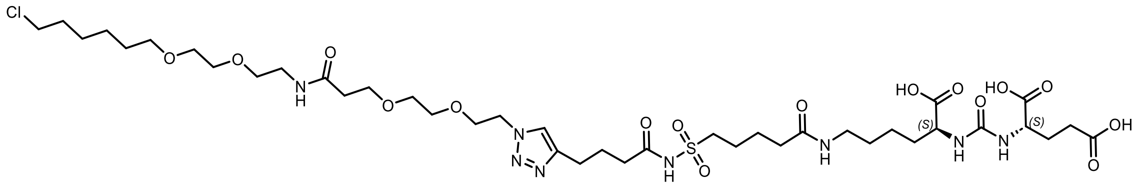

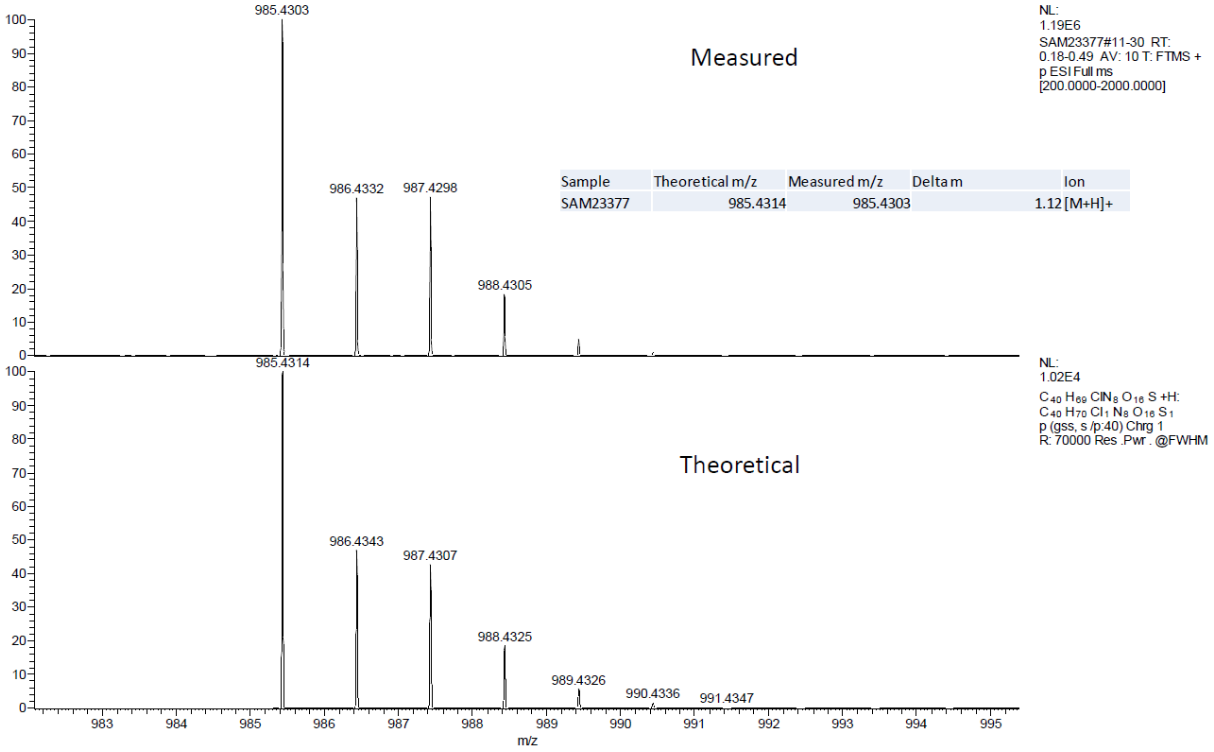

Sample	Theoretical m/z	Measured m/z	Delta m	Ion
SAM23377	985.4314	985.4303	1.12	[M+H]+

3.21.8 Compound **29** (FKBP-PSMA-GRC)

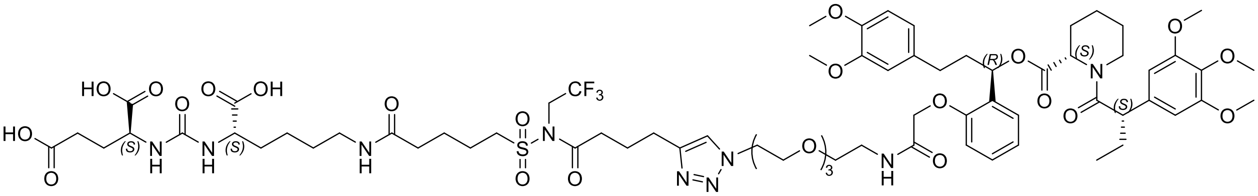

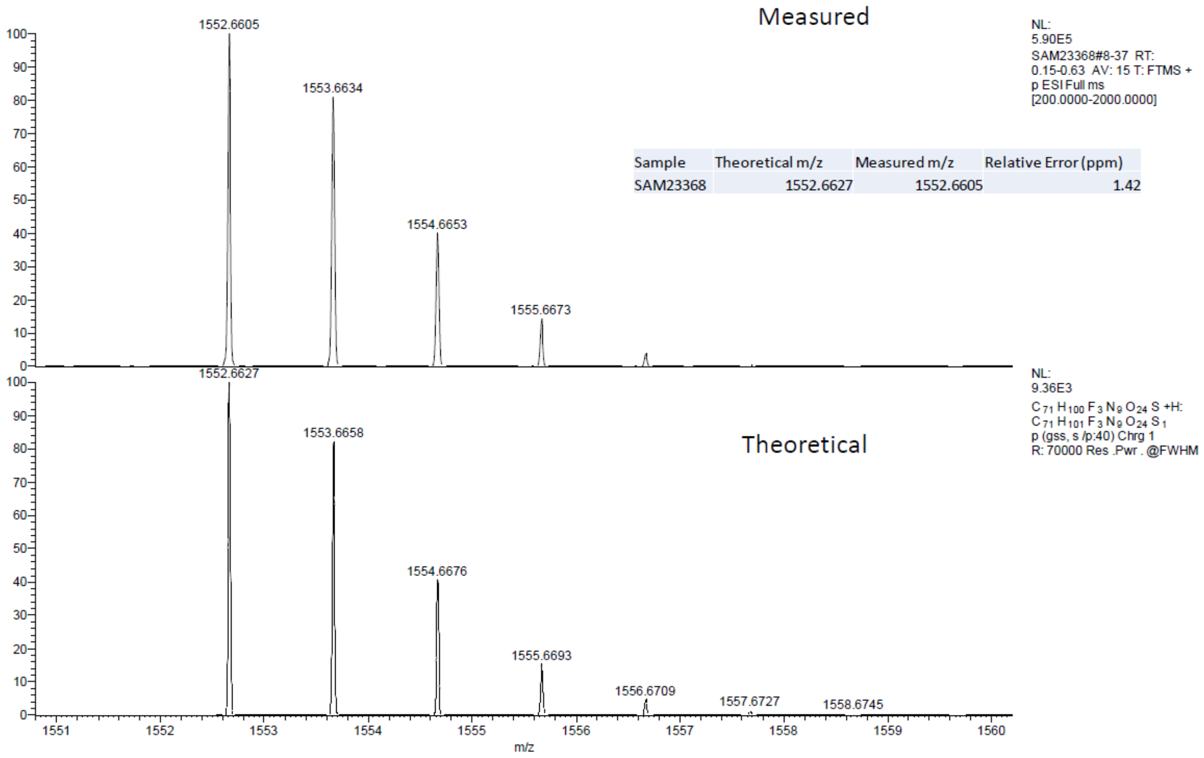

Sample	Theoretical m/z	Measured m/z	Relative Error (ppm)
SAM23368	1552.6627	1552.6605	1.42

3.21.9 Compound **30** (FKBP-PSMA-iGRC)

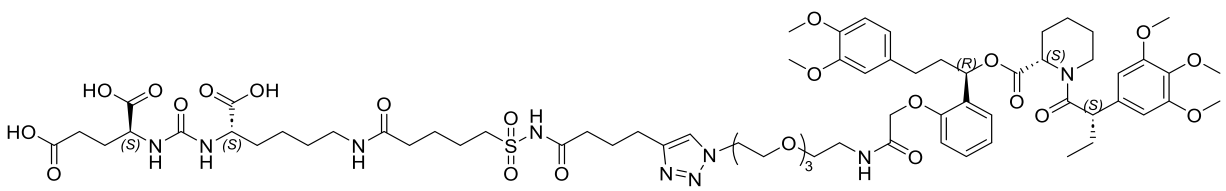

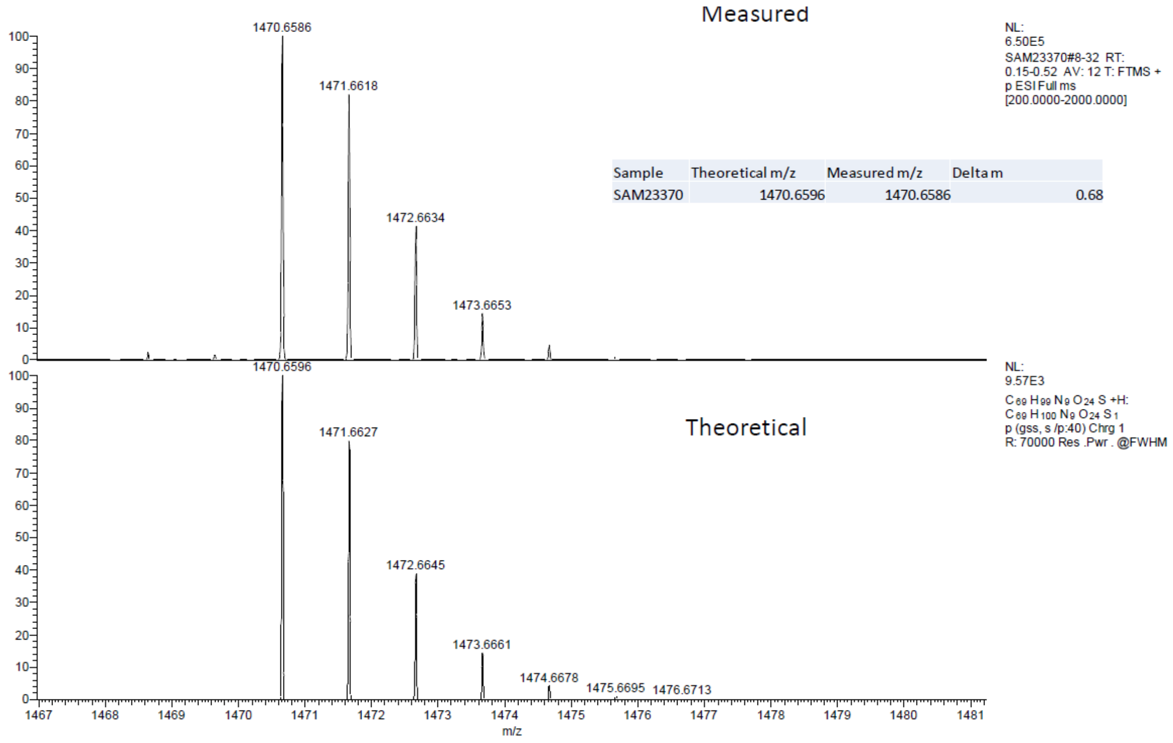

3.21.10 Compound **31** (Halo-BTK-GRC)

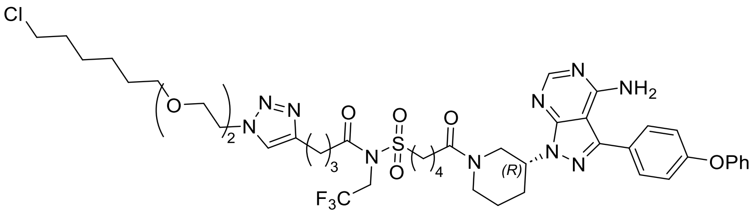

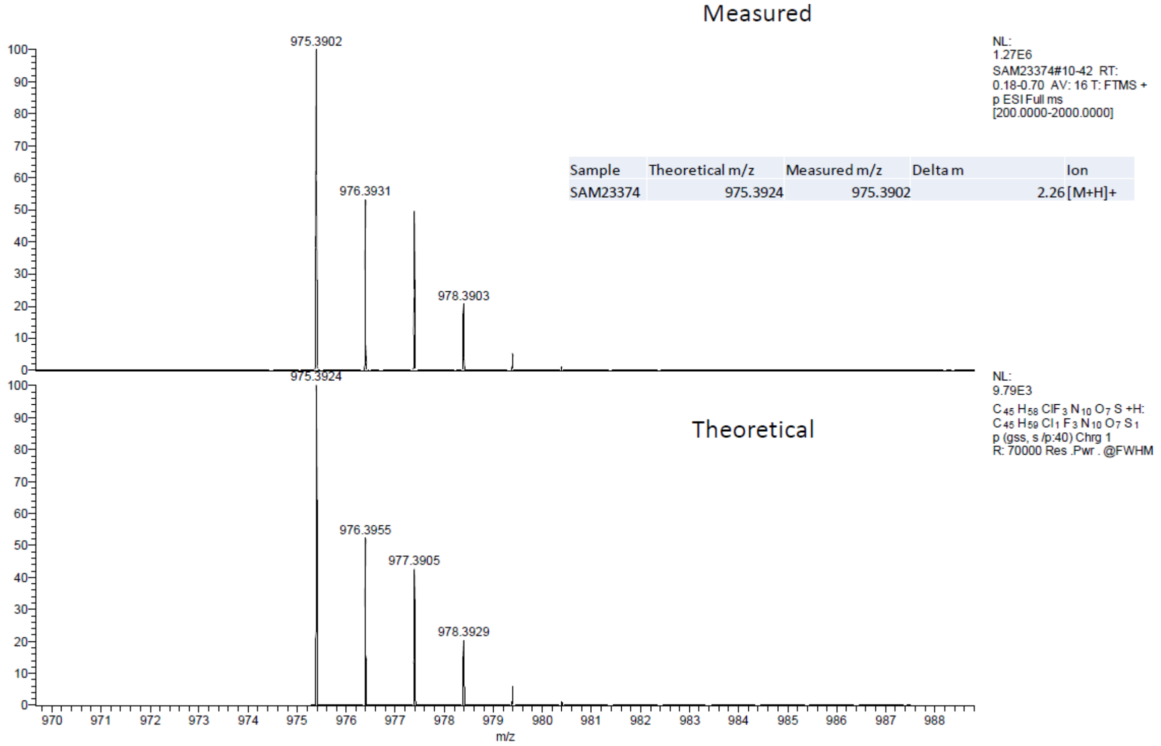

Sample	Theoretical m/z	Measured m/z	Delta m	Ion
SAM23374	975.3924	975.3902	2.26	[M+H]+

3.21.11 Compound **32** (Halo-BTK-iGRC)

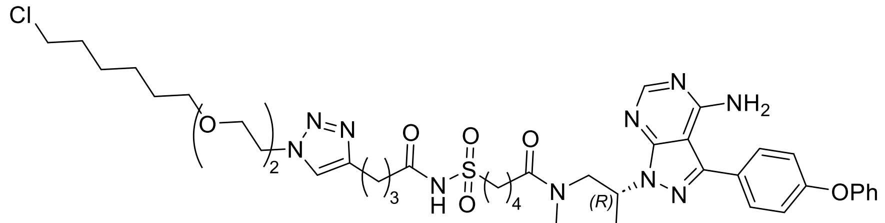

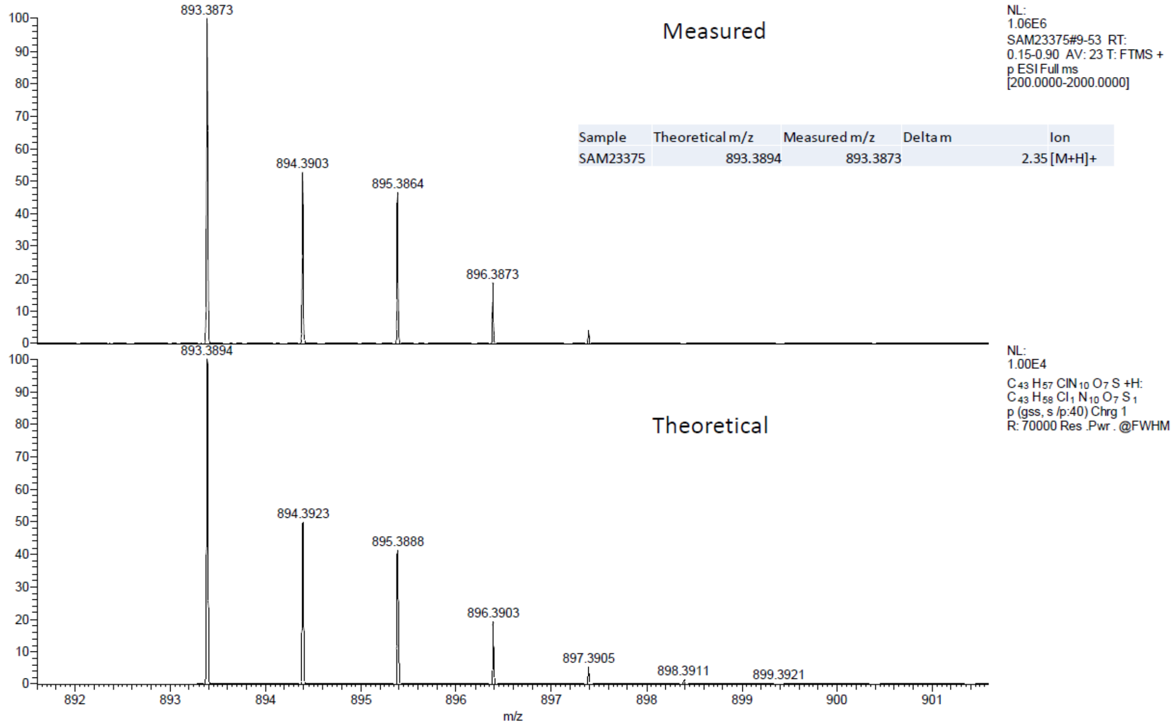

3.21.12 Compound **S6** (RIPK1 SuFA probe)

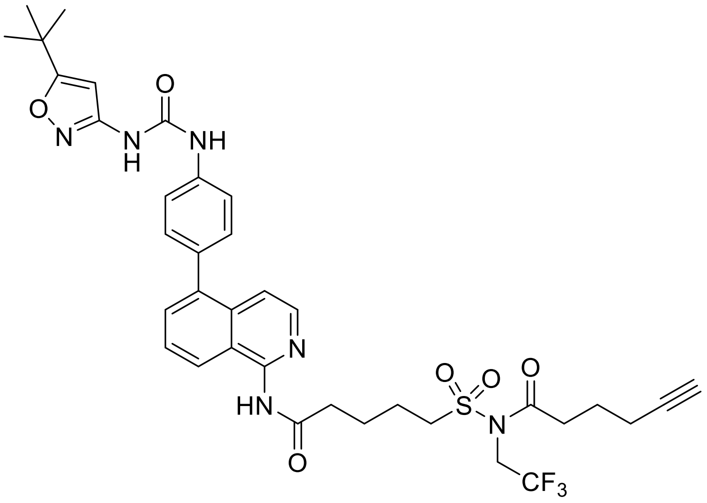

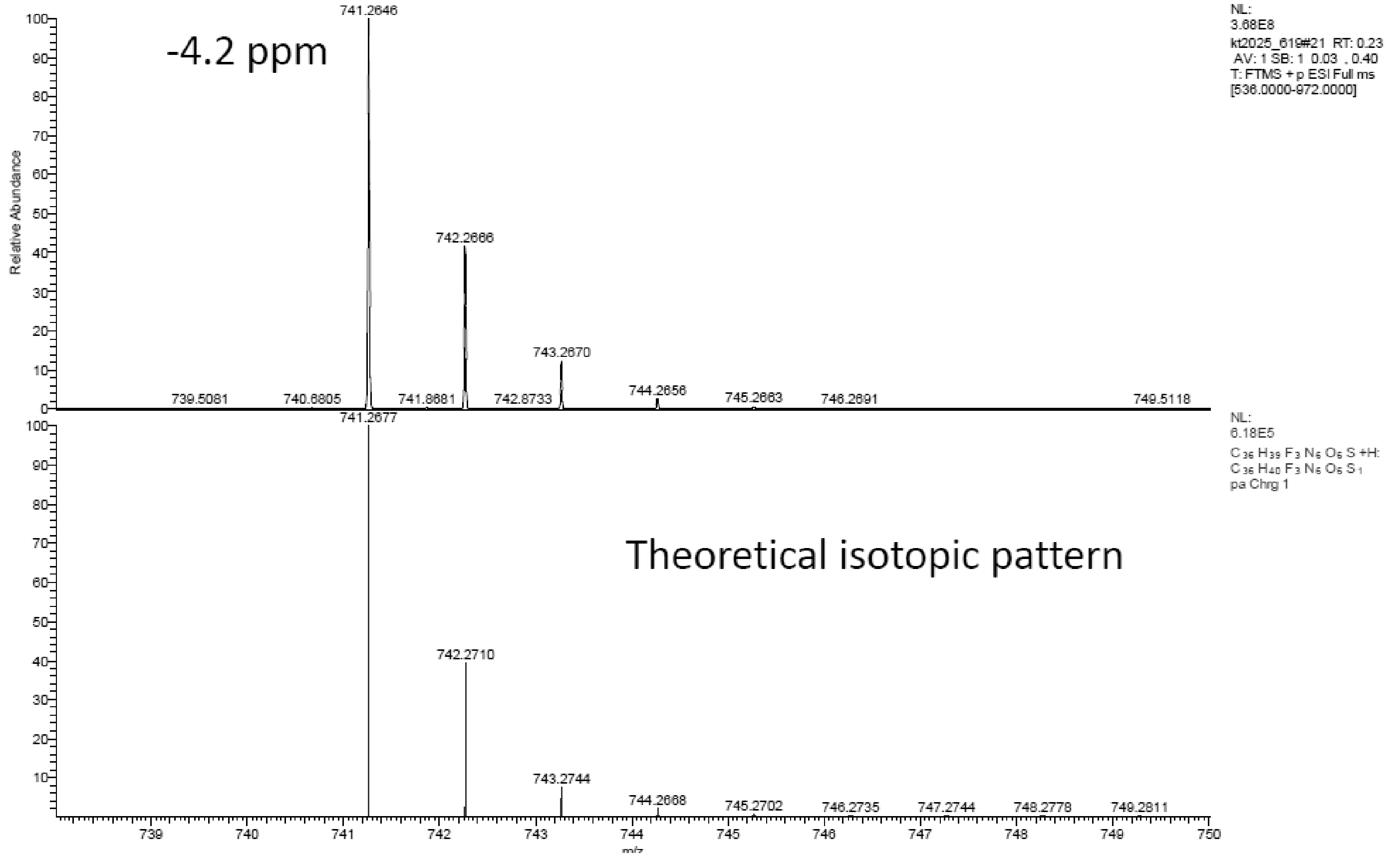

3.21.13 Compound **S7** (VEGFR2 NASA probe)

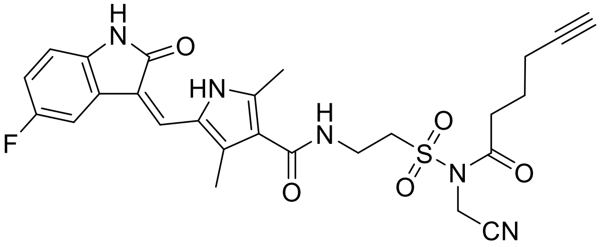

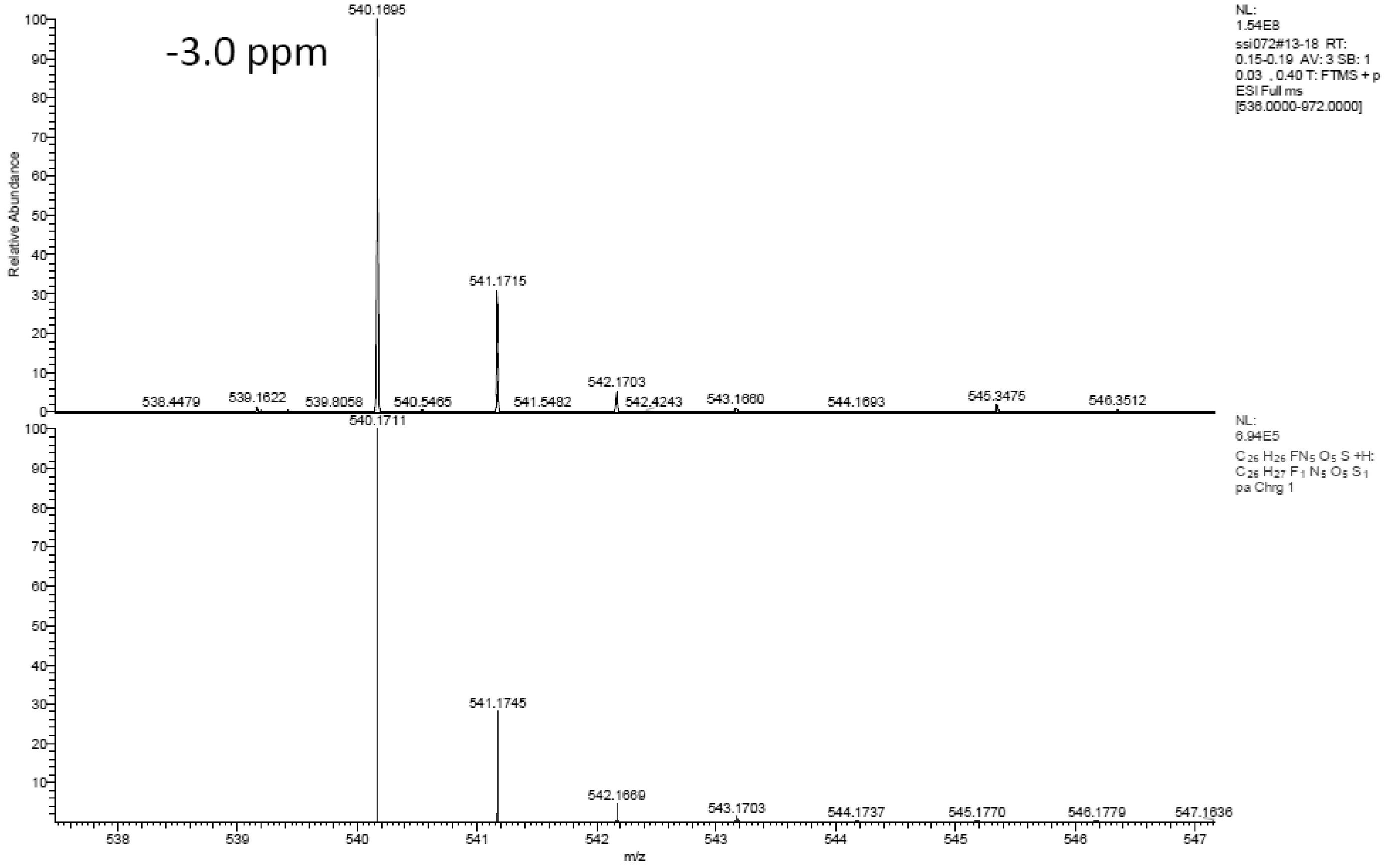

3.22 NMR spectra

3.22.1 <u>Compound **1** (RIPK1 NASA probe)</u>

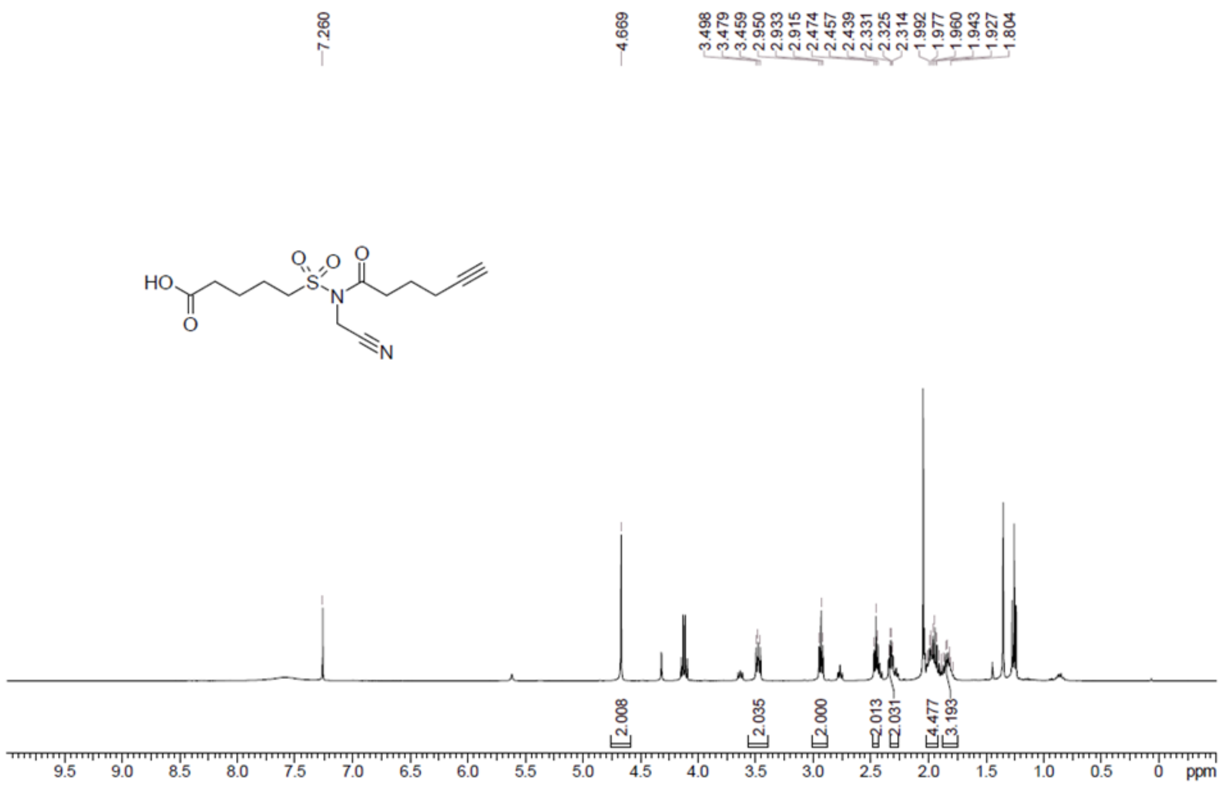

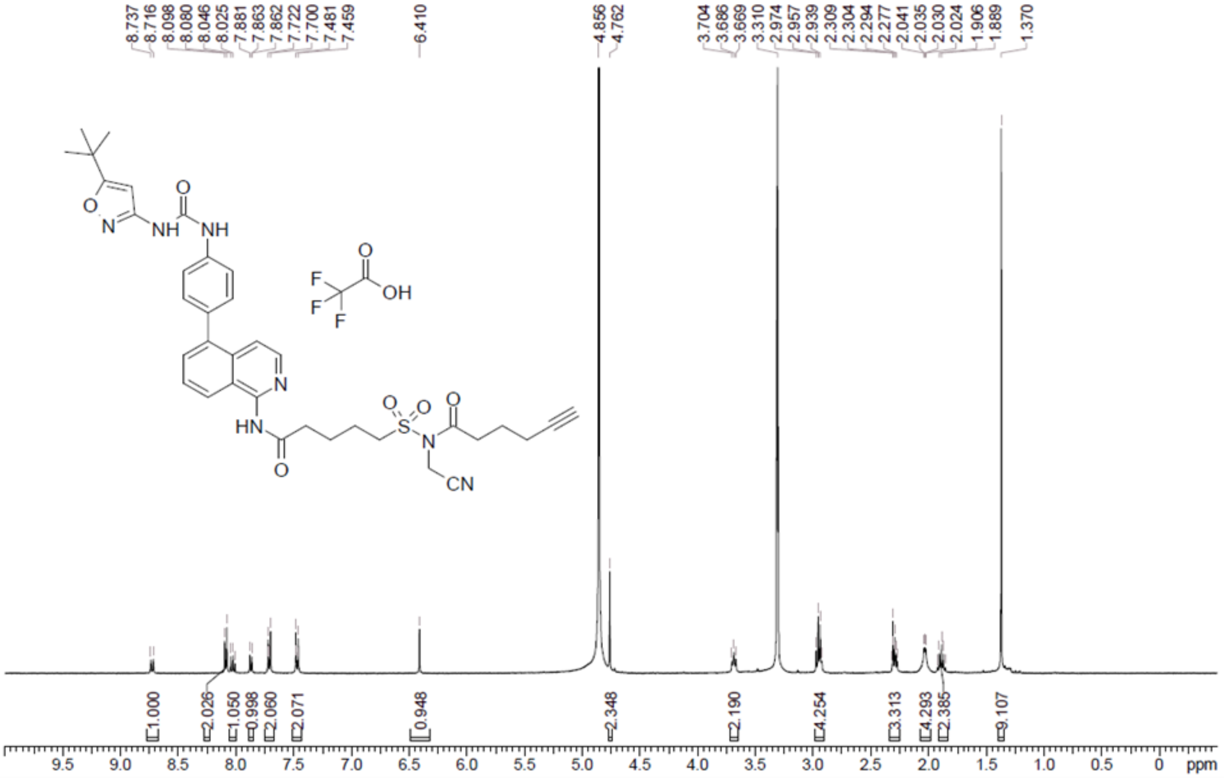

3.22.2 <u>Compound **2** (MEK2 NASA probe)</u>

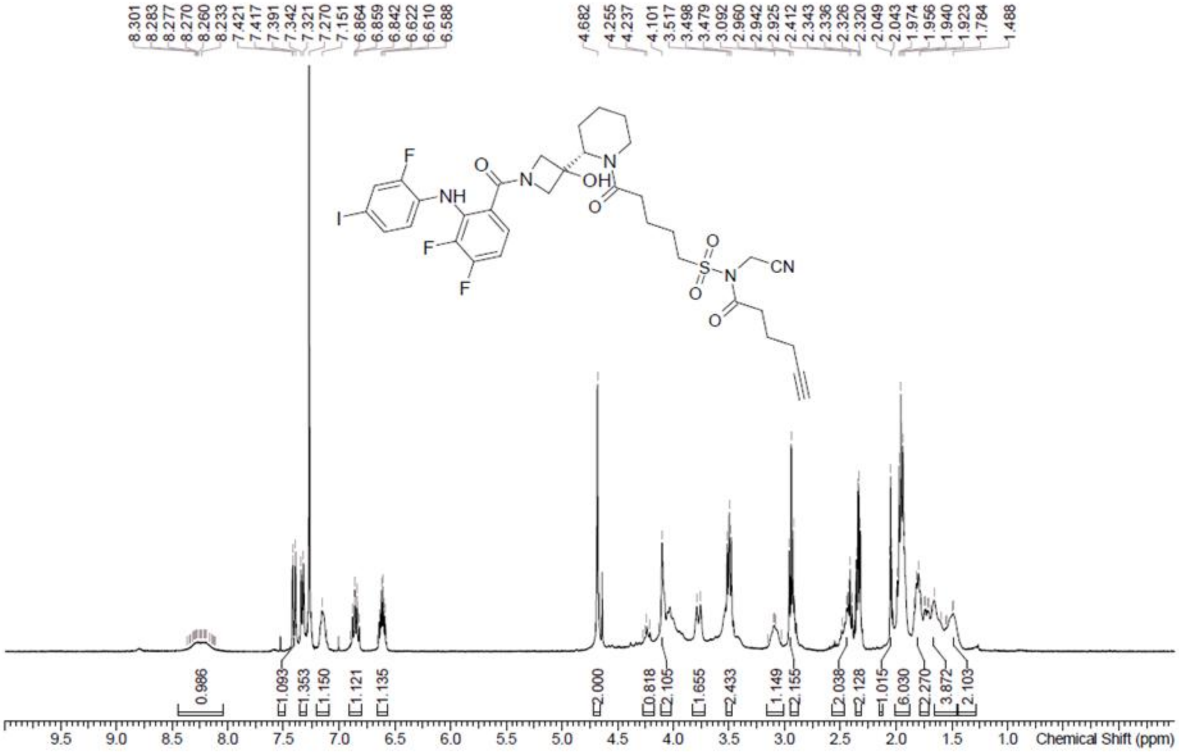

3.22.3 Compound **3** (IRE1α NASA probe)

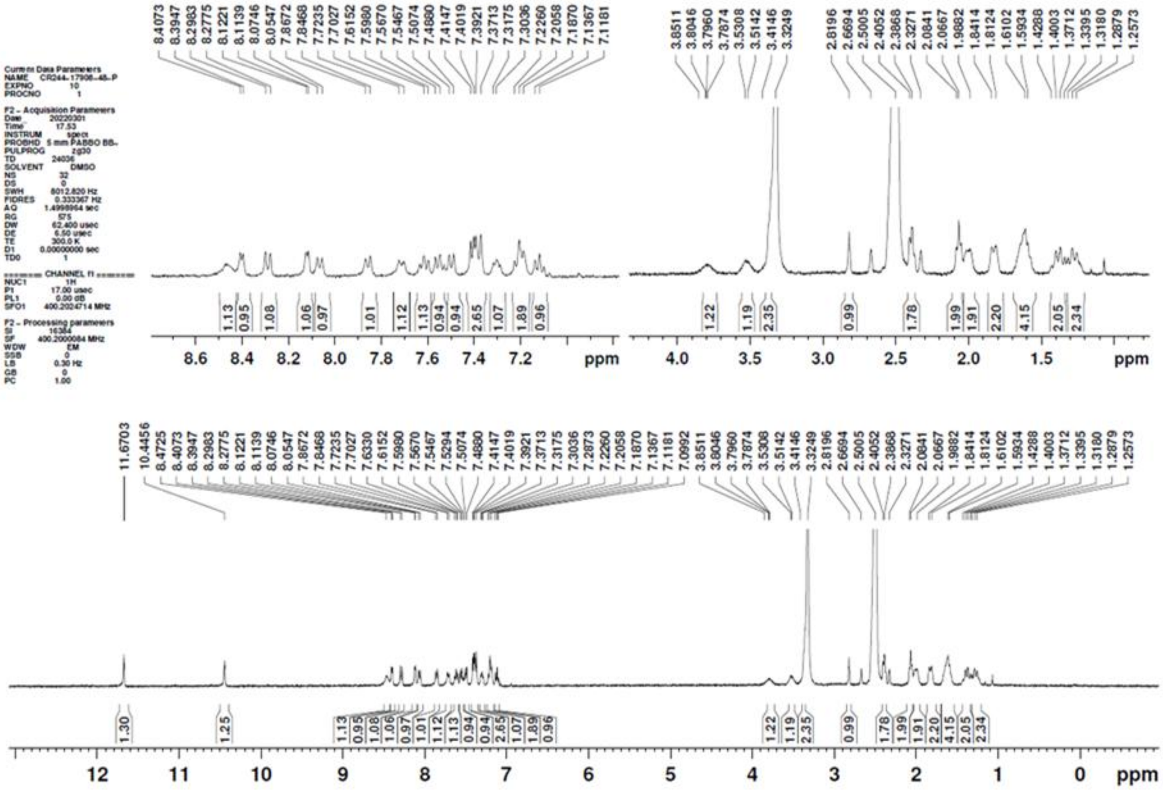

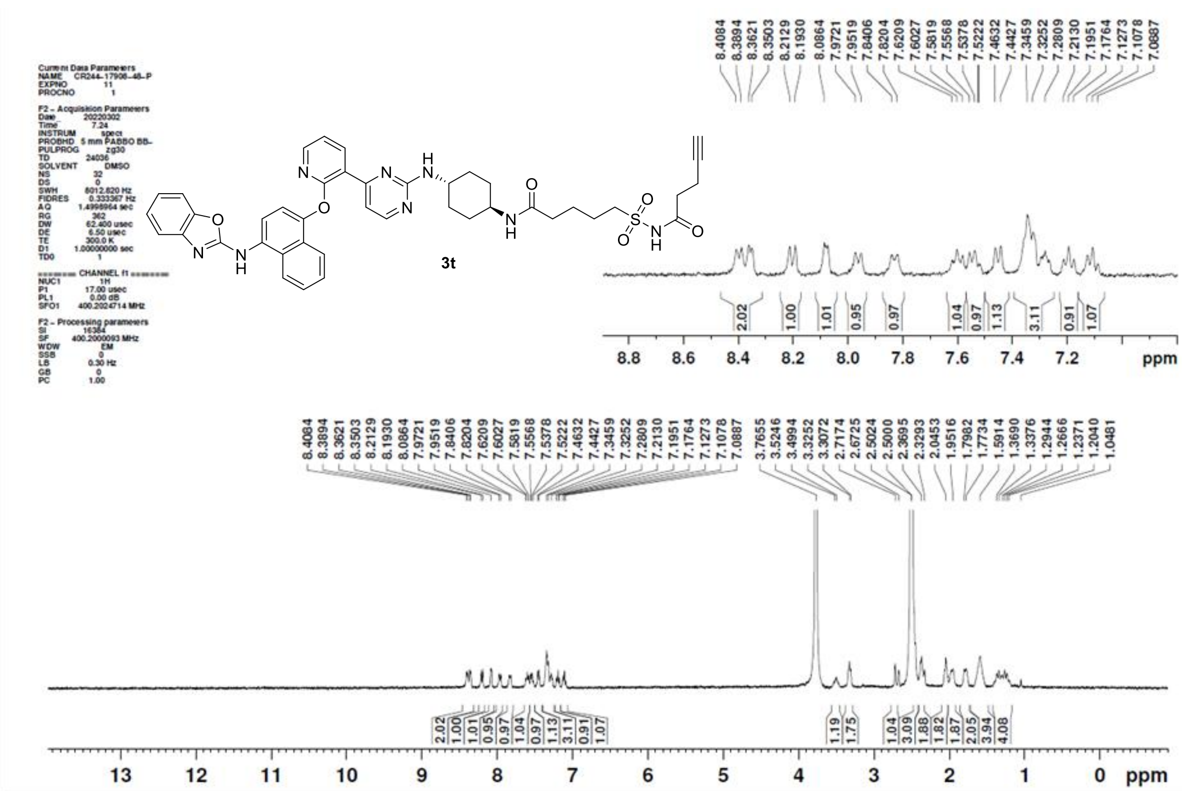

3.22.4 Compound **4** (MEK2 NASA probe)

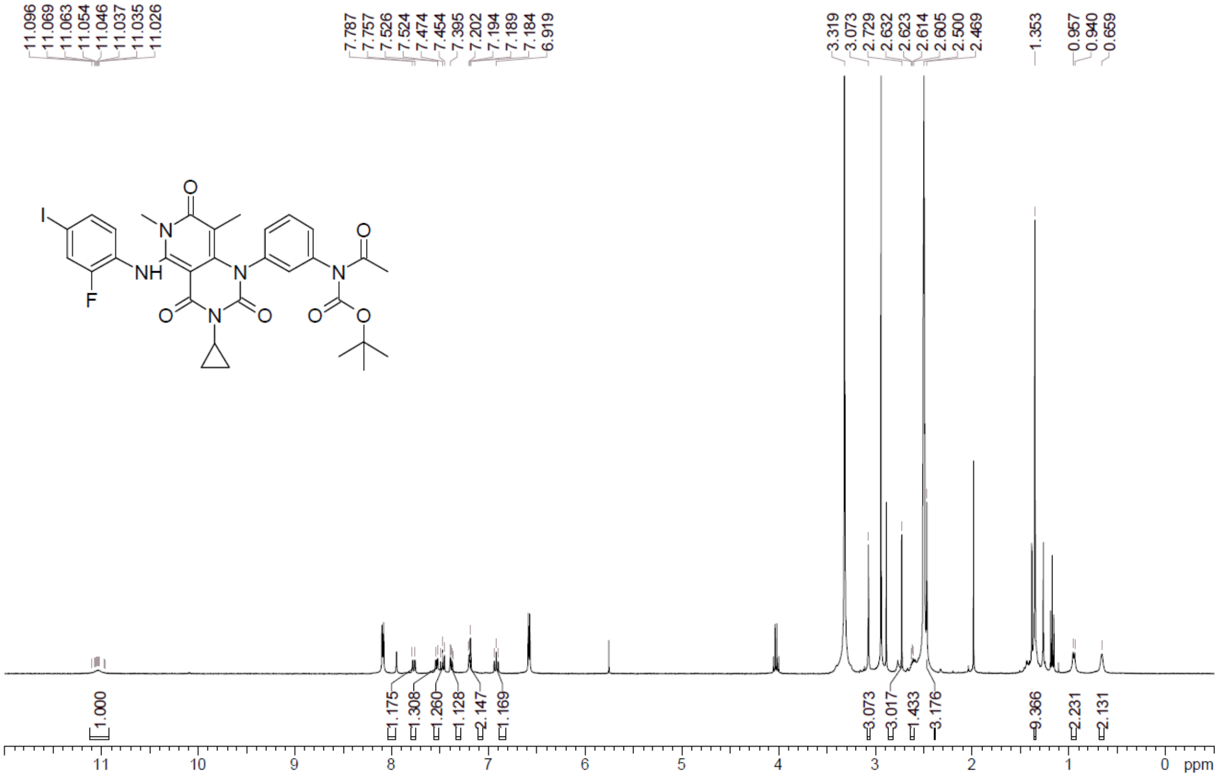

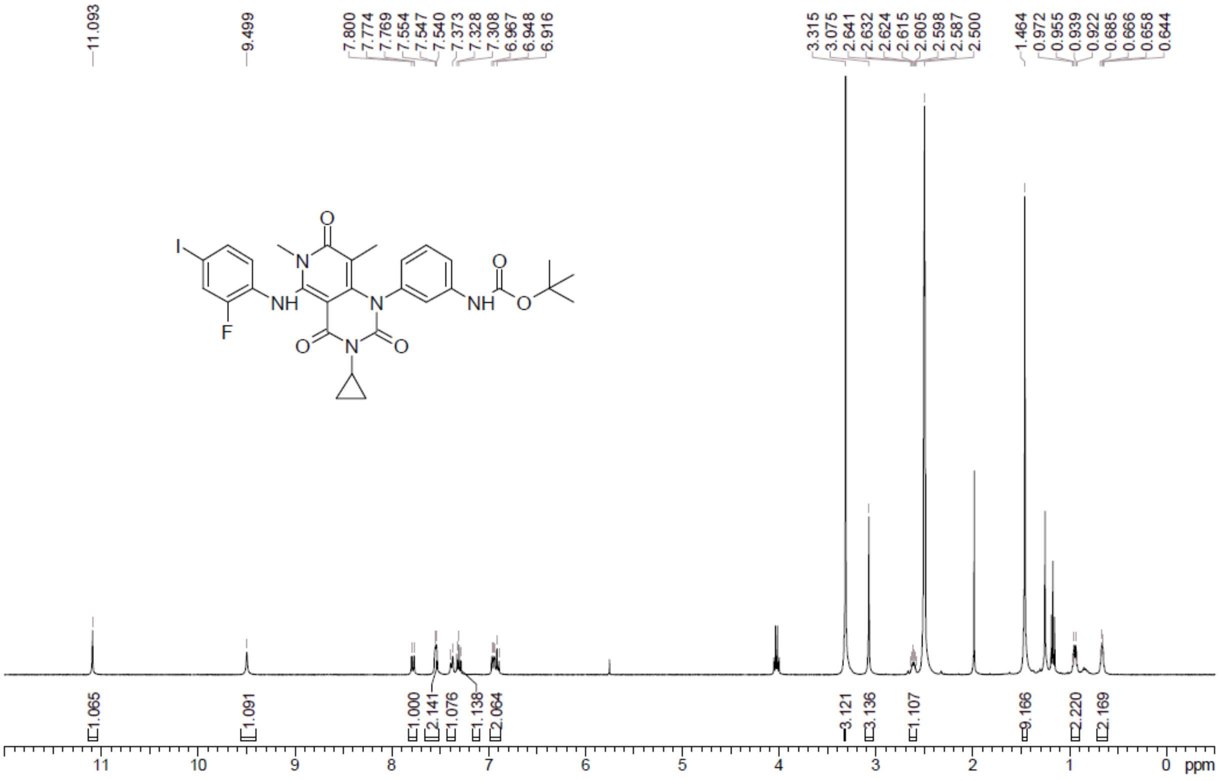

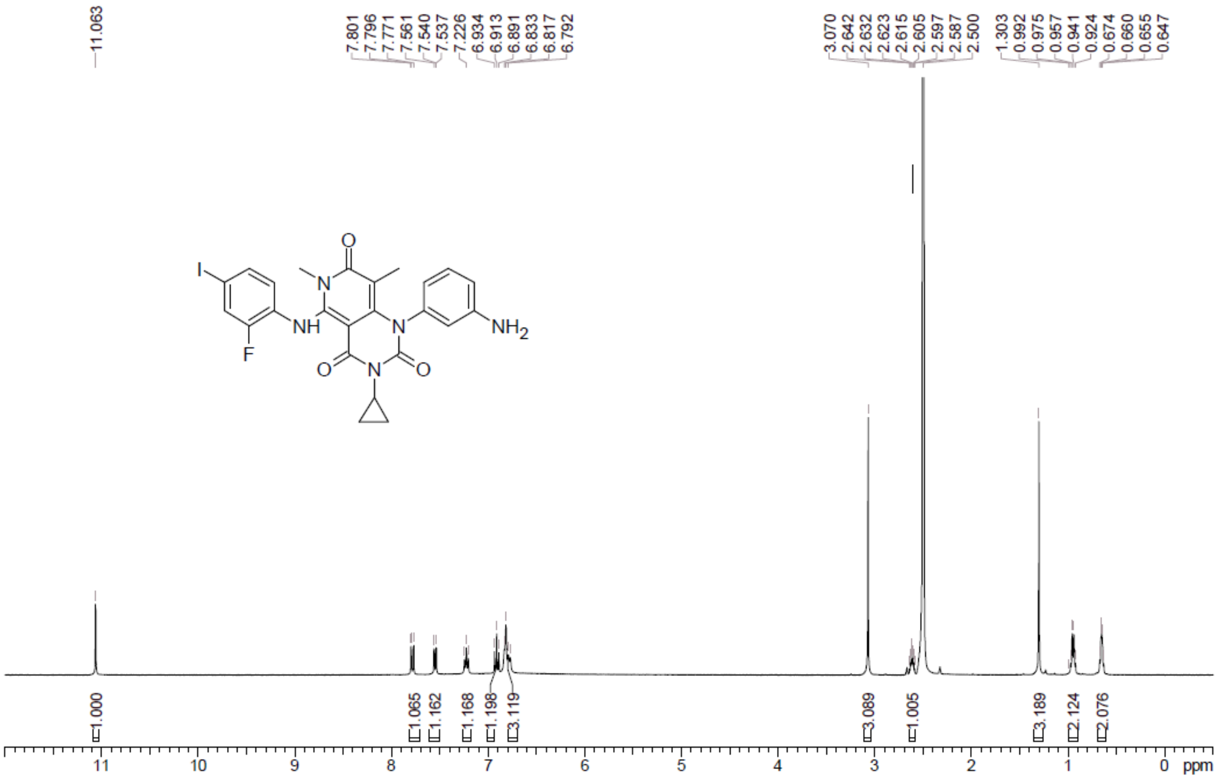
I
N
O
NH
F
NH2
ppm

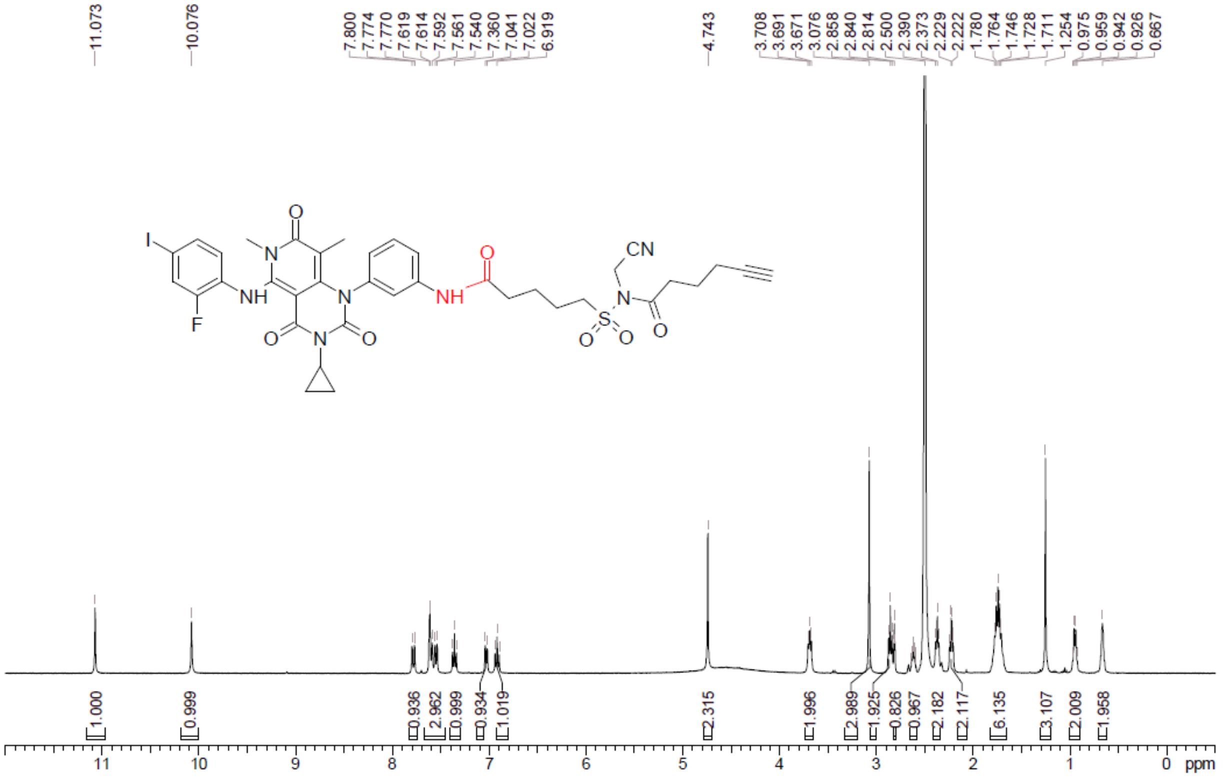
I
N
O
NH
F
CN
S
ppm

3.22.5 Compound **5** (LIMK1 NASA probe)

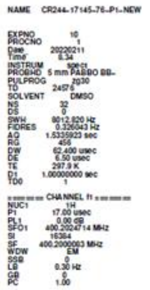

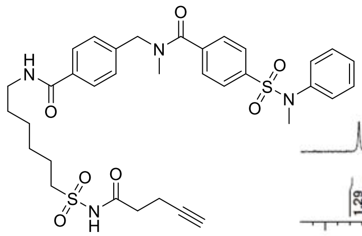

5q

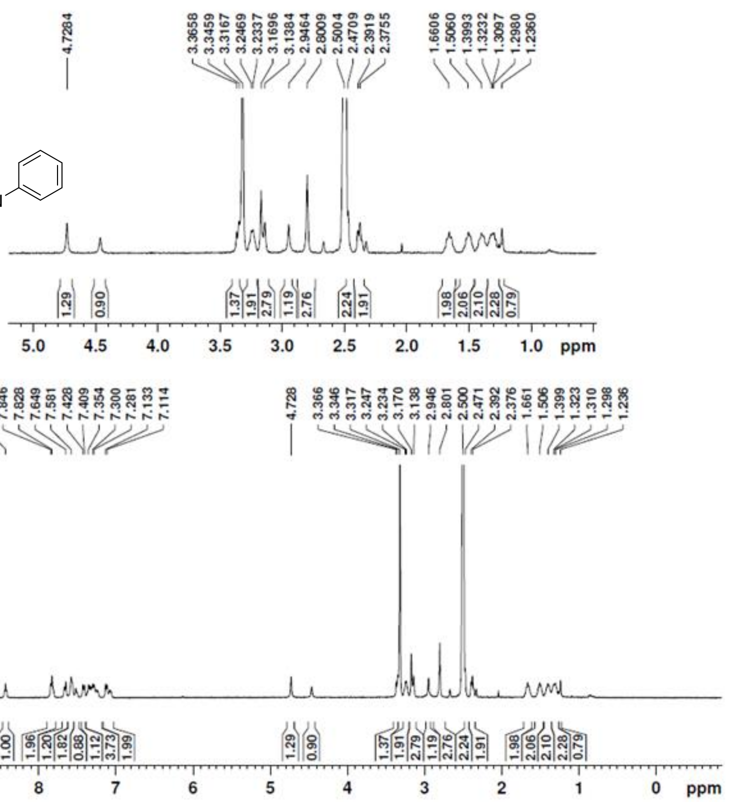

CR244–17145–76–P1–NEW IN DMSO AT 100 DEG C

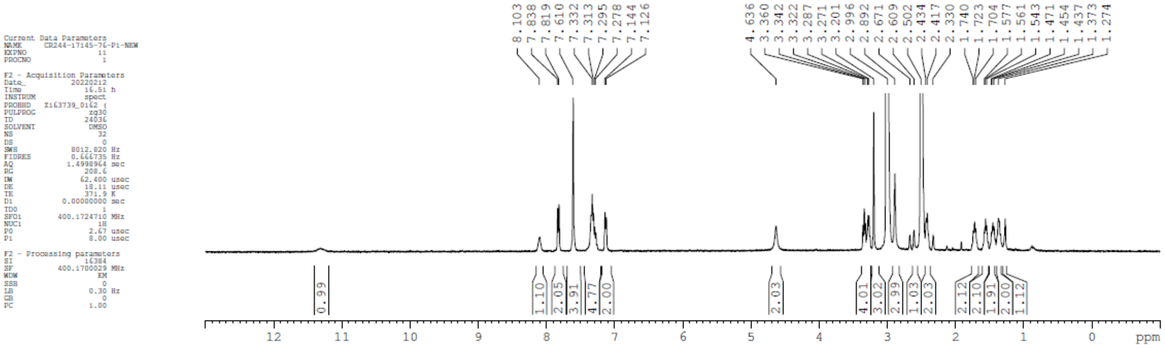

CR244–17145–76–P1–NEW IN DMSO AT 20 DEG C

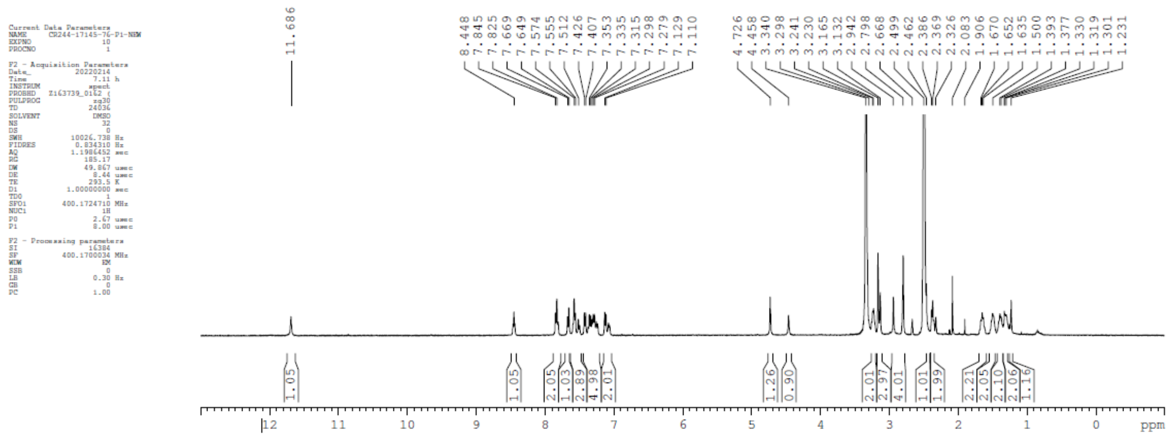

CR244-17145-76-P1-MeOD IN MeOD

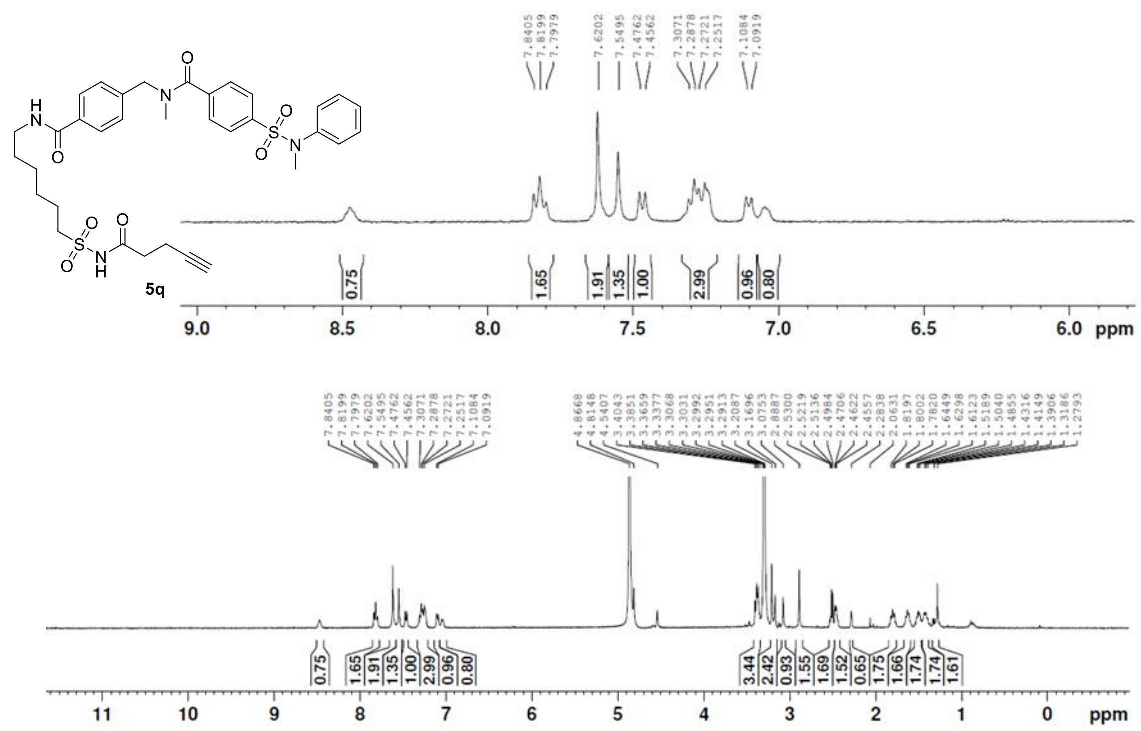

3.22.6 Compound **6** (EGFR NASA probe)

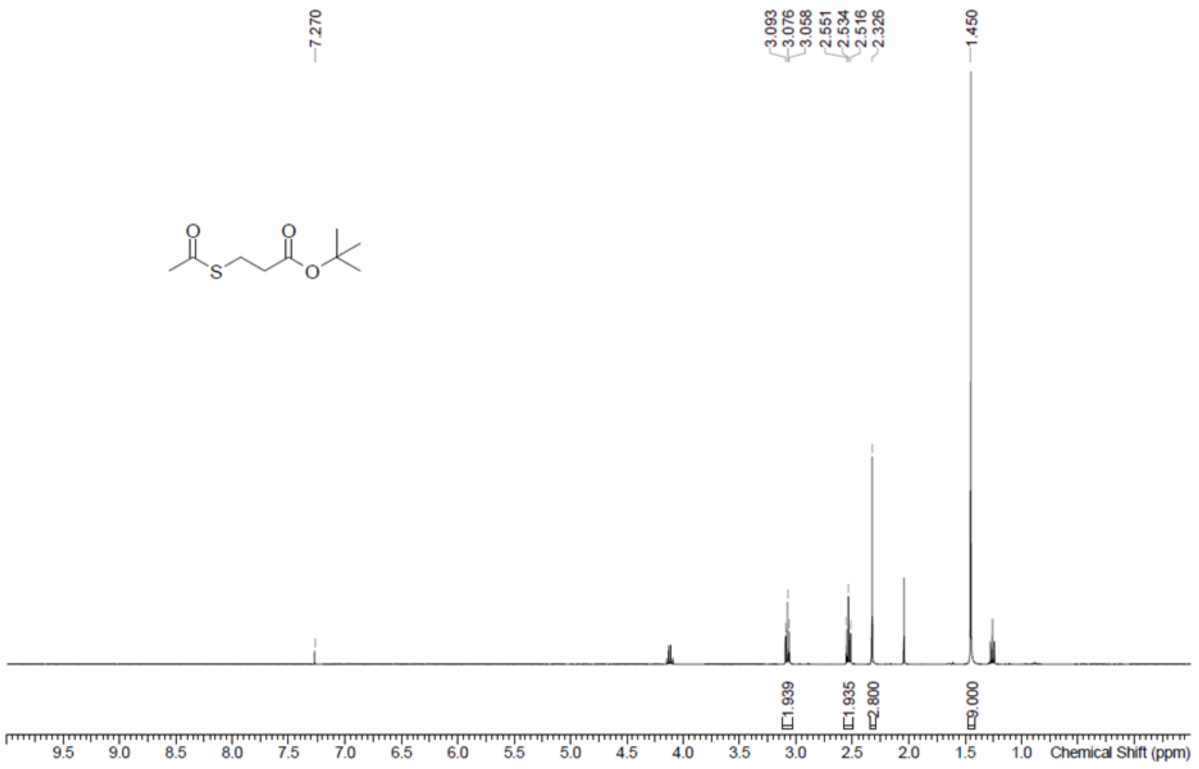

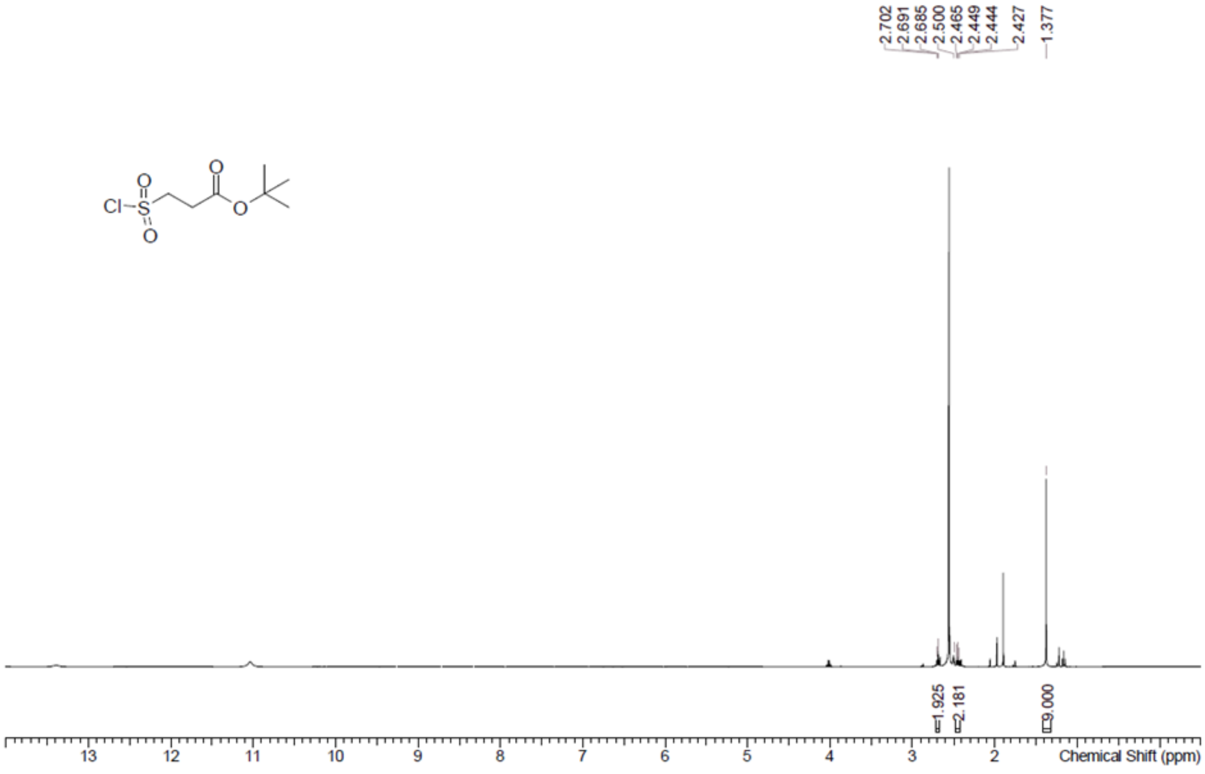

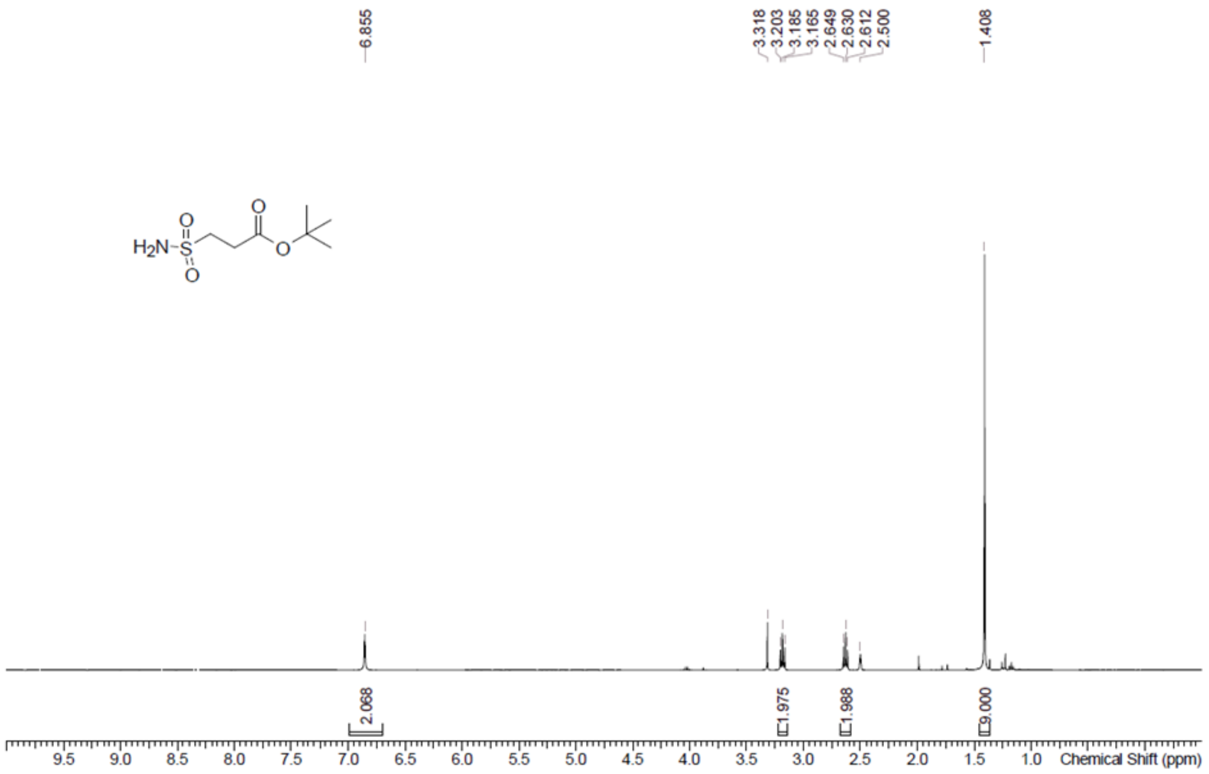
6.855
3.318
3.203
3.185
3.165
2.649
2.630
2.612
2.500
1.408
H_2N
O
S
O
O
O
2.068
1.975
1.988
9.000
Chemical Shift (ppm)

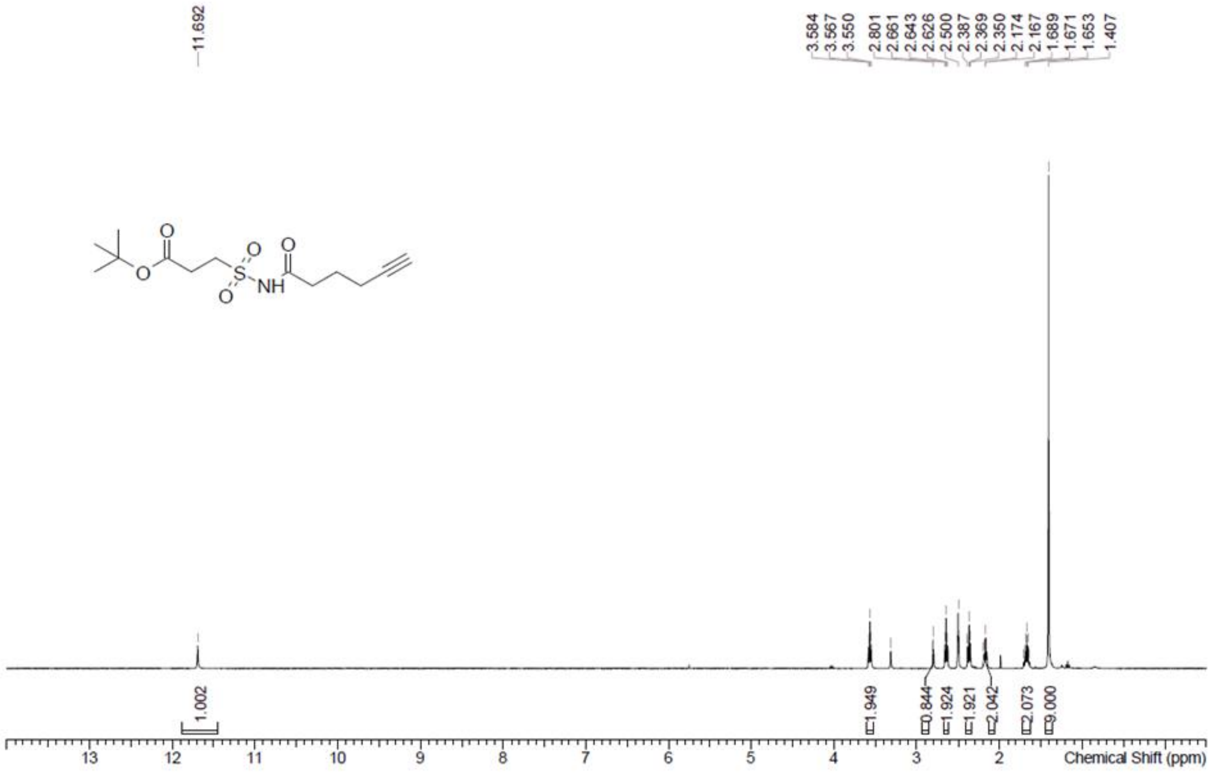
11.692
3.584
3.567
3.550
2.801
2.661
2.643
2.626
2.500
2.387
2.369
2.350
2.174
2.167
1.689
1.671
1.653
1.407
O
O
S
O
O
NH
O
1.002
1.949
0.844
1.924
1.921
2.042
2.073
9.000
Chemical Shift (ppm)

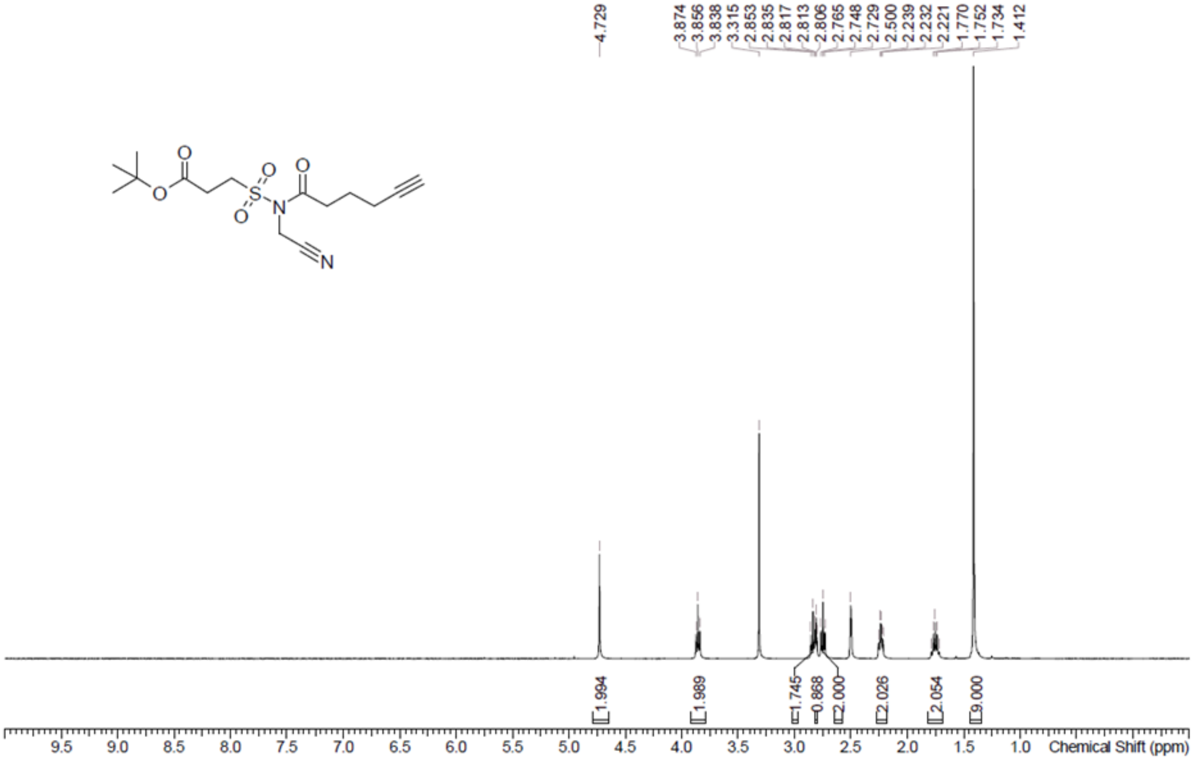

4.729
3.874 3.856 3.838 3.315 2.853 2.835 2.817 2.813 2.806 2.765 2.748 2.729 2.500 2.239 2.232 2.221 1.770 1.752 1.734 1.412
1.994 1.989 1.745 0.868 2.000 2.026 2.054 9.000
9.5 9.0 8.5 8.0 7.5 7.0 6.5 6.0 5.5 5.0 4.5 4.0 3.5 3.0 2.5 2.0 1.5 1.0 Chemical Shift (ppm)

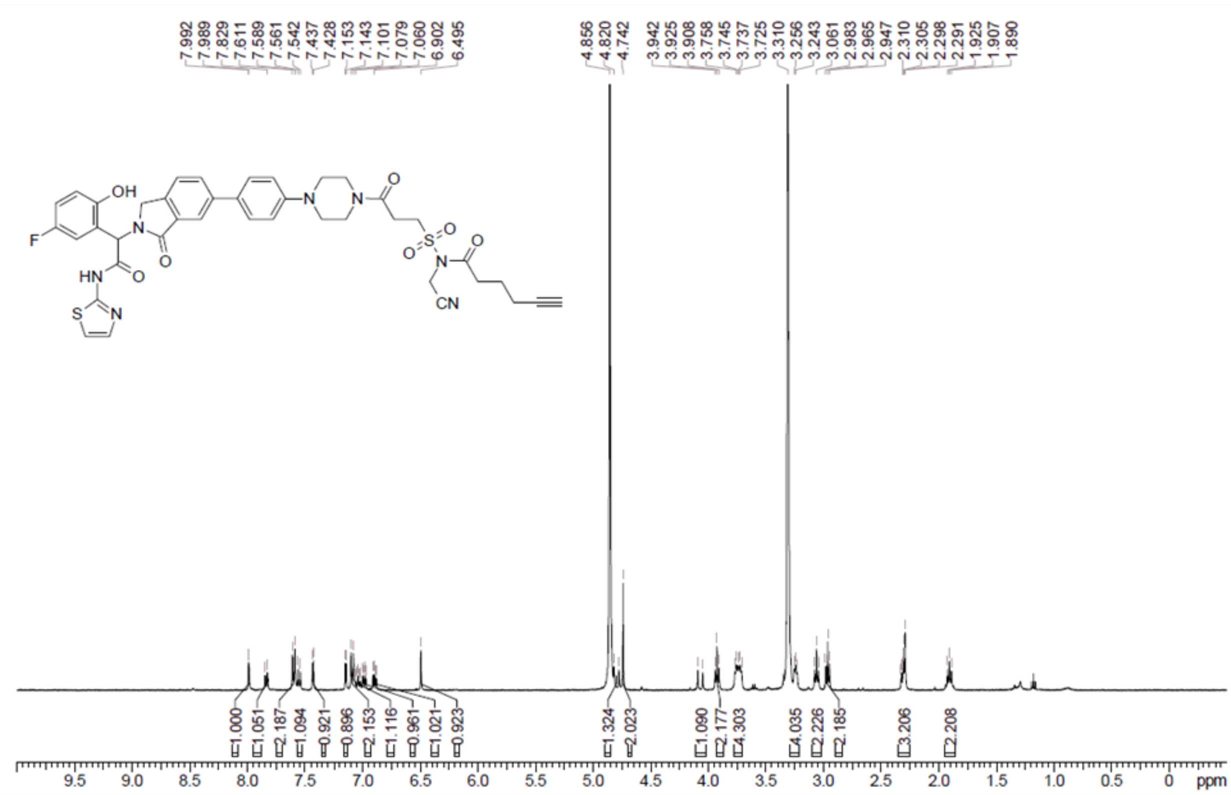

7.992 7.989 7.829 7.611 7.589 7.561 7.542 7.437 7.428 7.153 7.143 7.101 7.079 7.060 6.902 6.495
4.856 4.820 4.742
3.942 3.925 3.908 3.758 3.745 3.737 3.725 3.310 3.256 3.243 3.061 2.983 2.965 2.947 2.310 2.305 2.298 2.291 1.925 1.907 1.890
1.000 1.051 2.187 1.094 0.921 0.896 2.153 1.116 0.961 1.021 0.923
1.324 2.023
1.090 2.177 4.303 4.035 2.226 2.185 3.206 2.208
9.5 9.0 8.5 8.0 7.5 7.0 6.5 6.0 5.5 5.0 4.5 4.0 3.5 3.0 2.5 2.0 1.5 1.0 0.5 0 ppm

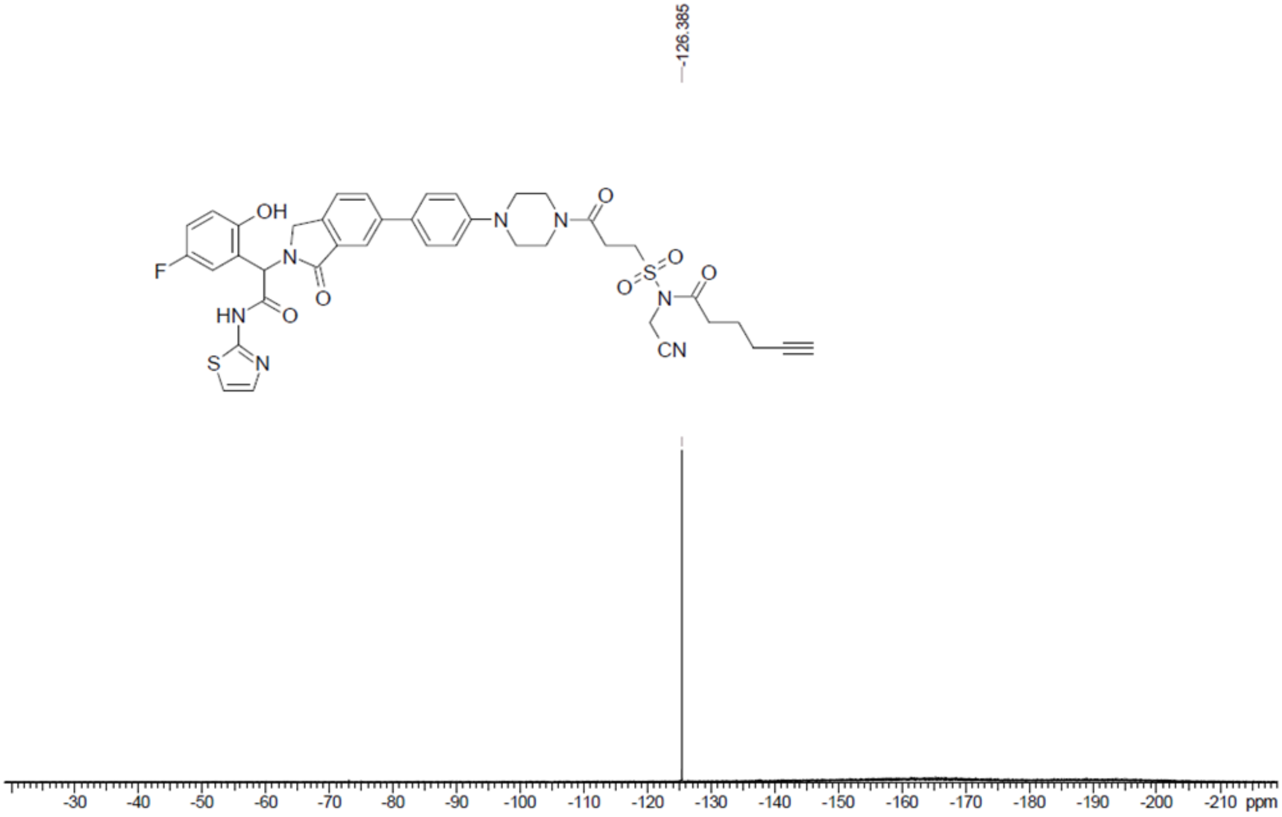
-126.385
OH
F
N
O
HN
O
S
N
N
N
O
S
O
O
N
O
CN
-30
-40
-50
-60
-70
-80
-90
-100
-110
-120
-130
-140
-150
-160
-170
-180
-190
-200
-210
ppm

3.22.7 <u>*N*-substituted *N*-acyl sulfonamide library **7-14**</u>

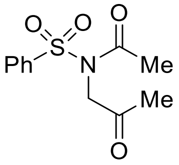

Compound 7

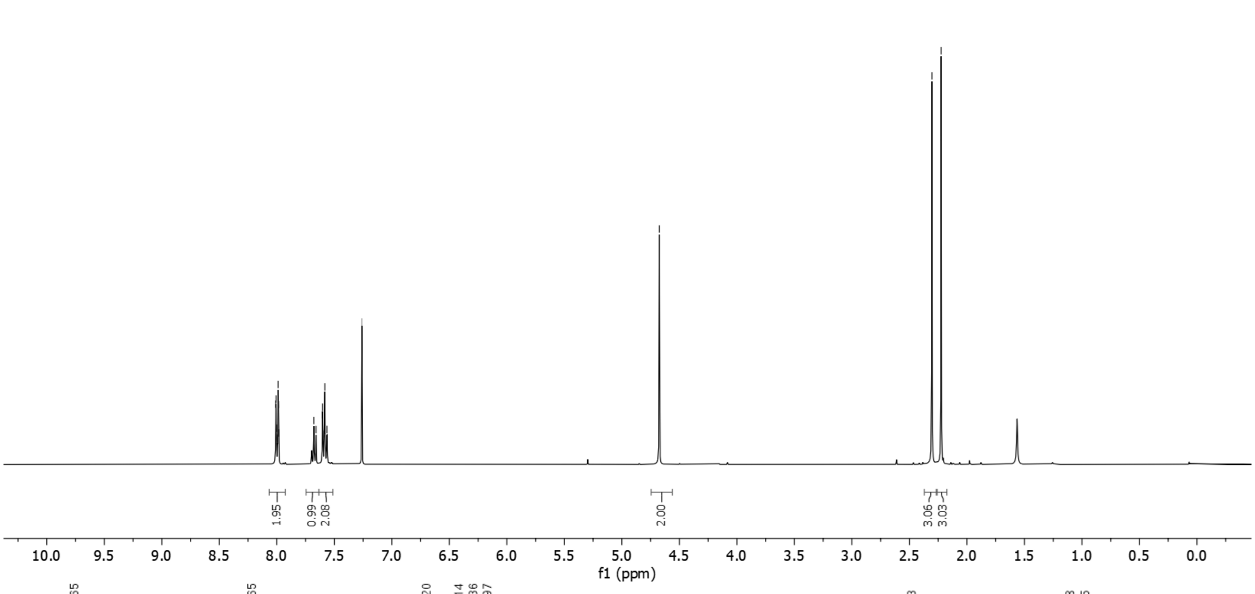

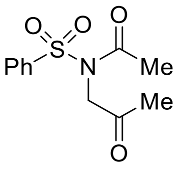

Compound 7

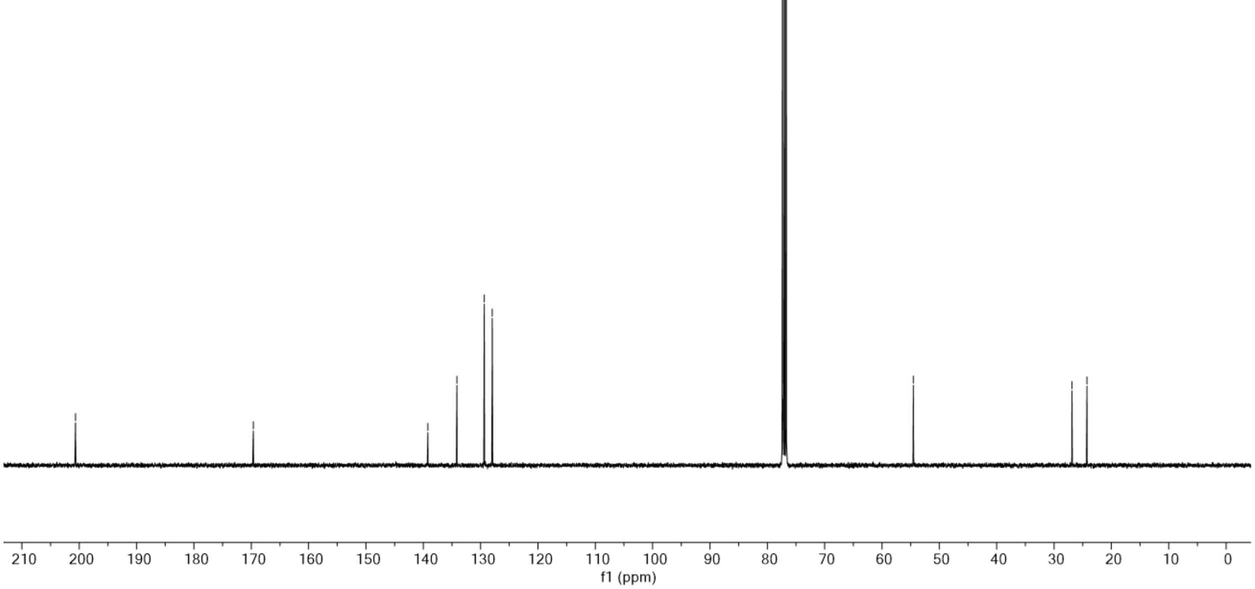

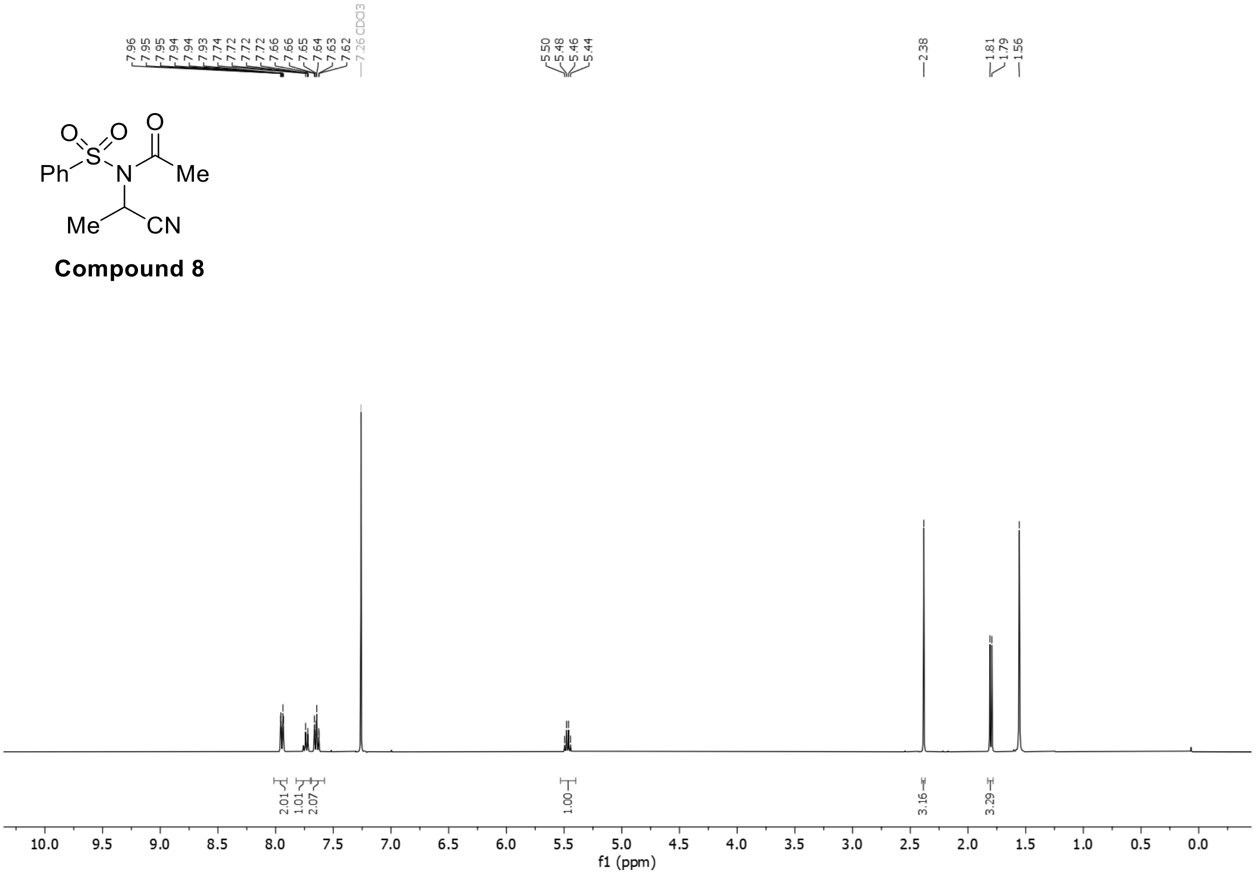

Compound 8

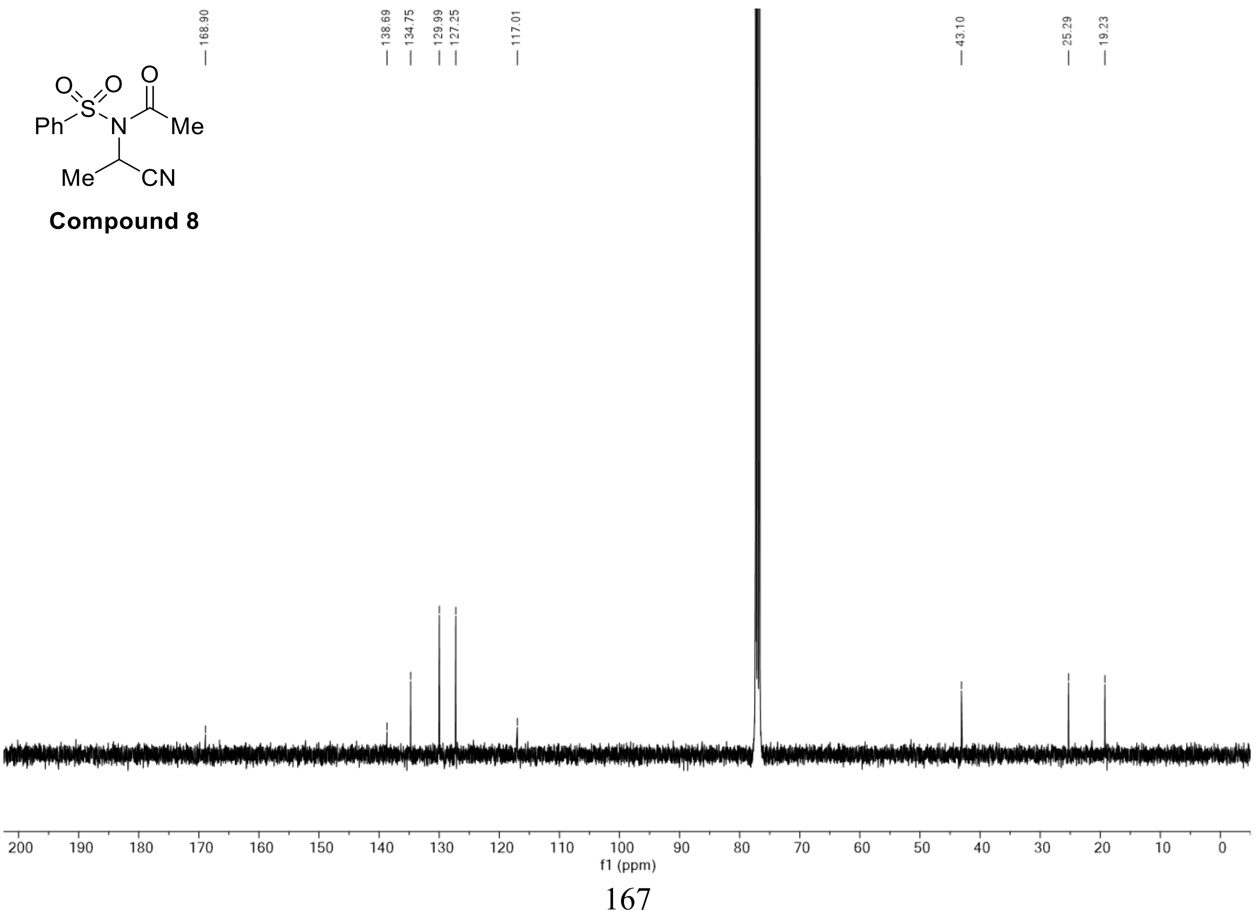

Compound 8

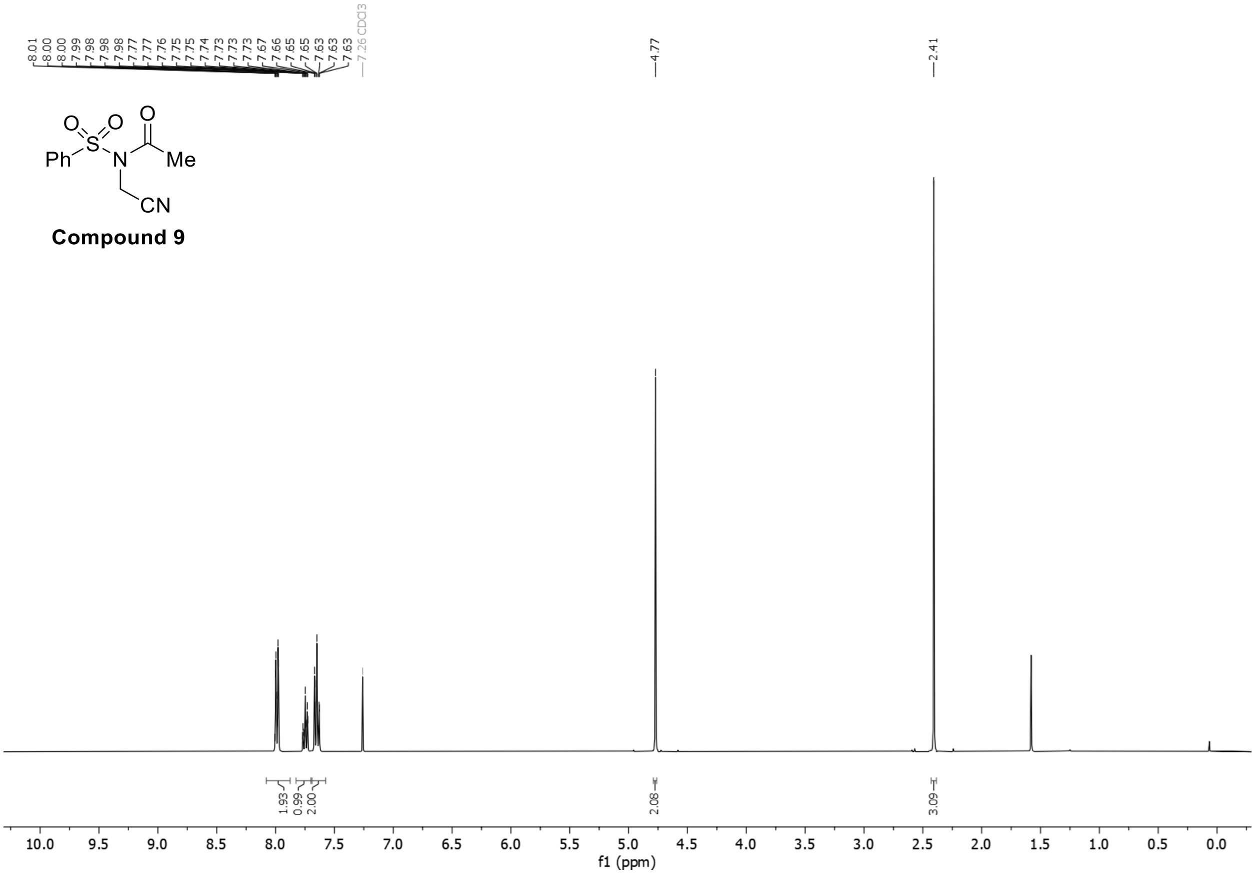
8.01 8.00 8.00 7.99 7.98 7.98 7.98 7.77 7.77 7.76 7.75 7.75 7.74 7.73 7.73 7.73 7.73 7.67 7.66 7.65 7.65 7.63 7.63 7.63
7.26 CDCl3
4.77
2.41
Ph
Me
CN
Compound 9
1.93
0.99
2.00
2.08
3.09
f1 (ppm)

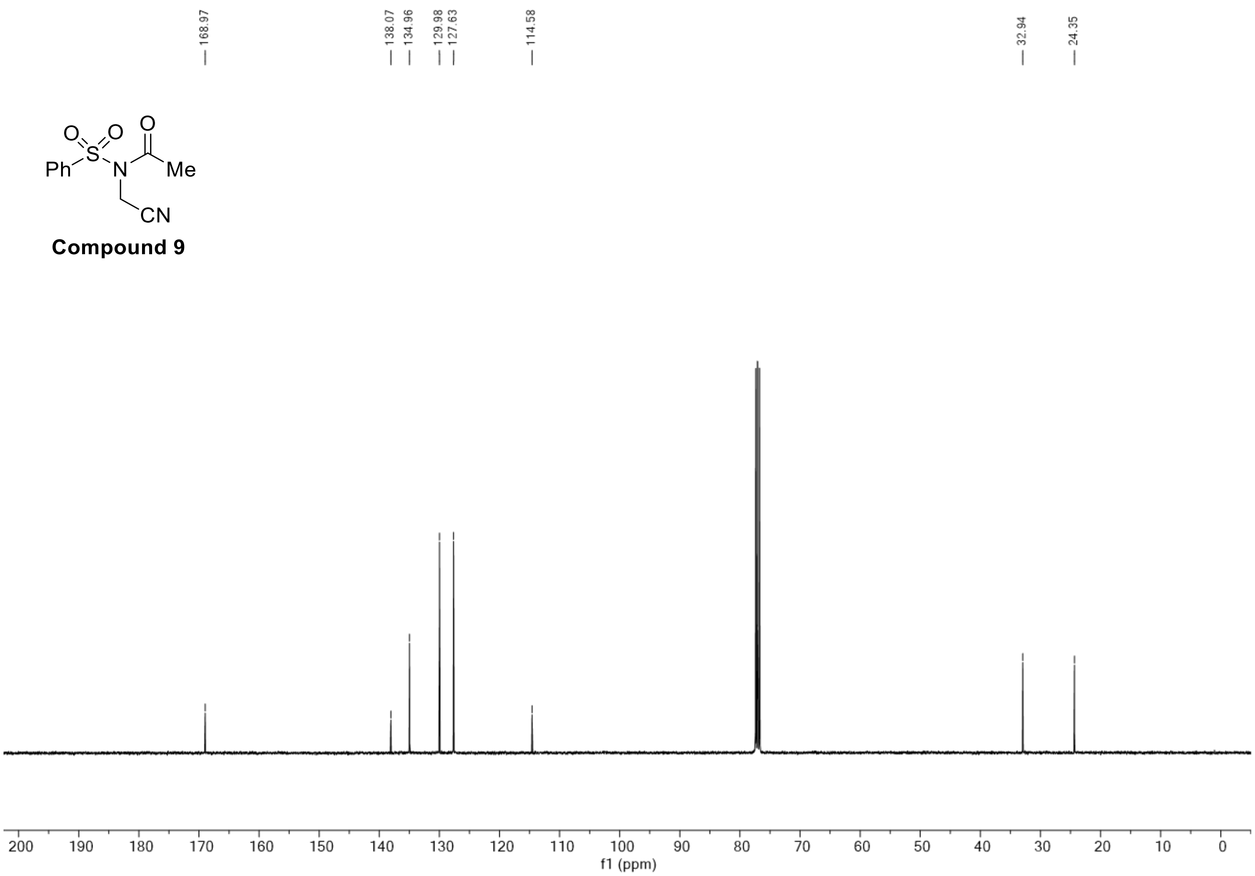
168.97
138.07
134.96
129.98
127.63
114.58
32.94
24.35
Ph
Me
CN
Compound 9
f1 (ppm)

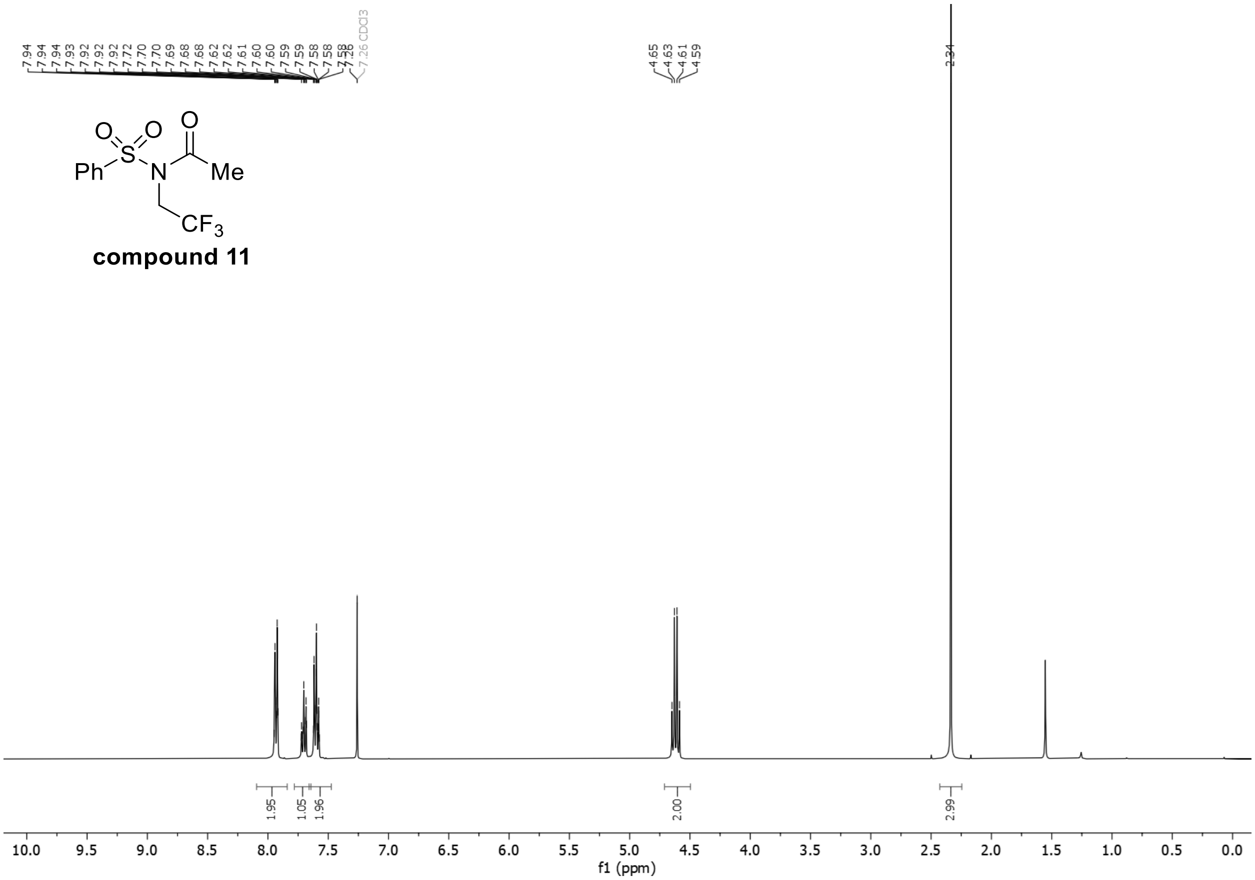
compound 11
7.26 CDCl3
f1 (ppm)

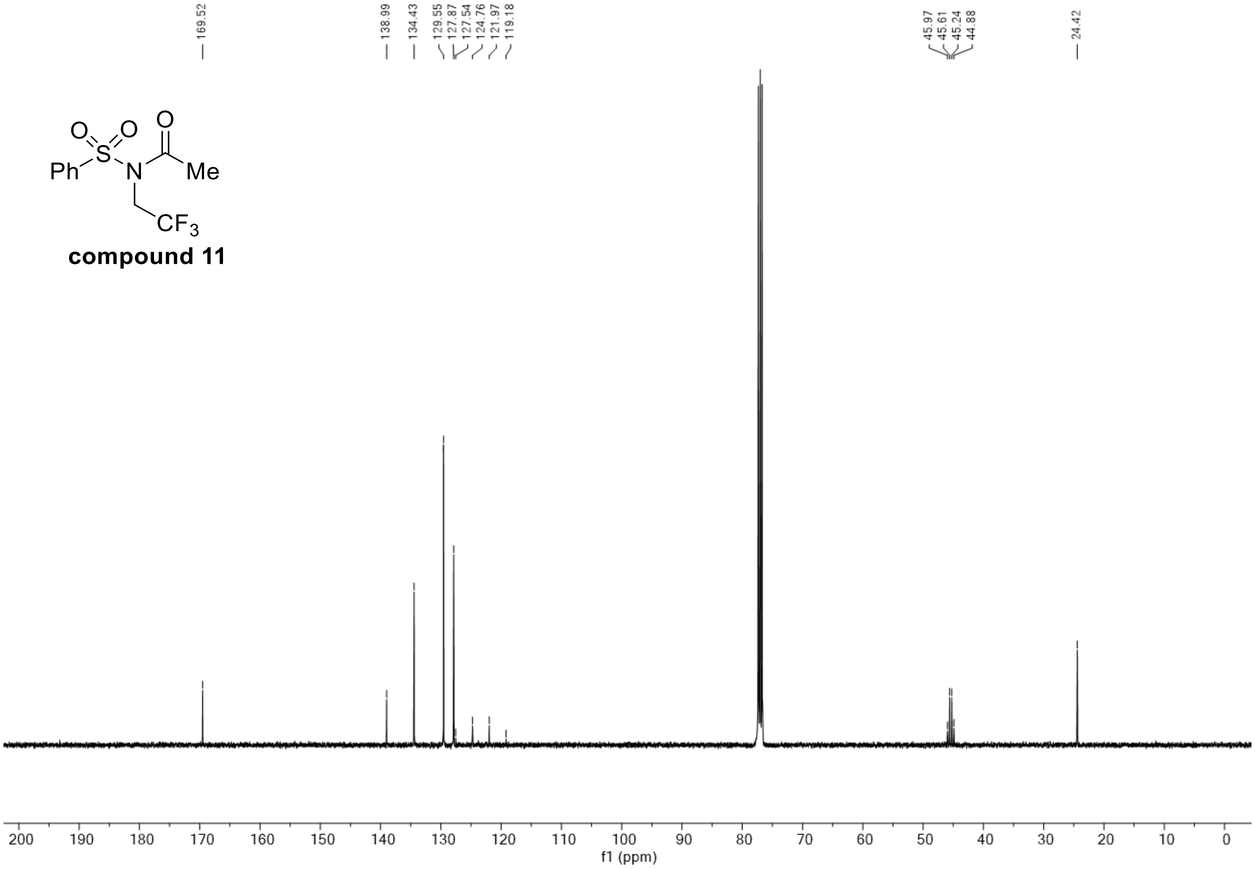
169.52
138.99
134.43
129.55
127.87
127.54
124.76
121.97
119.18
45.97
45.61
45.24
44.88
24.42
Ph
S
N
Me
CF3
compound 11
f1 (ppm)

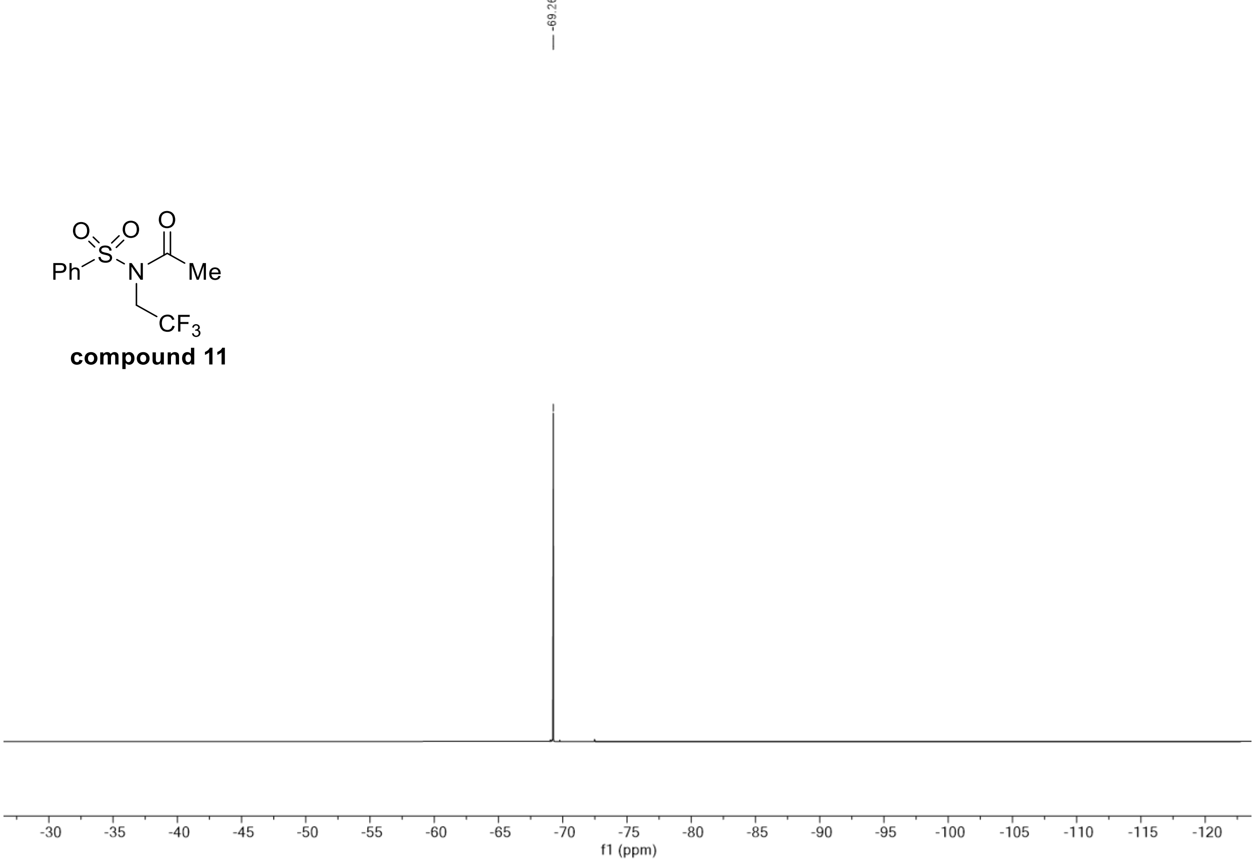
-69.26
Ph
S
N
Me
CF3
compound 11
f1 (ppm)

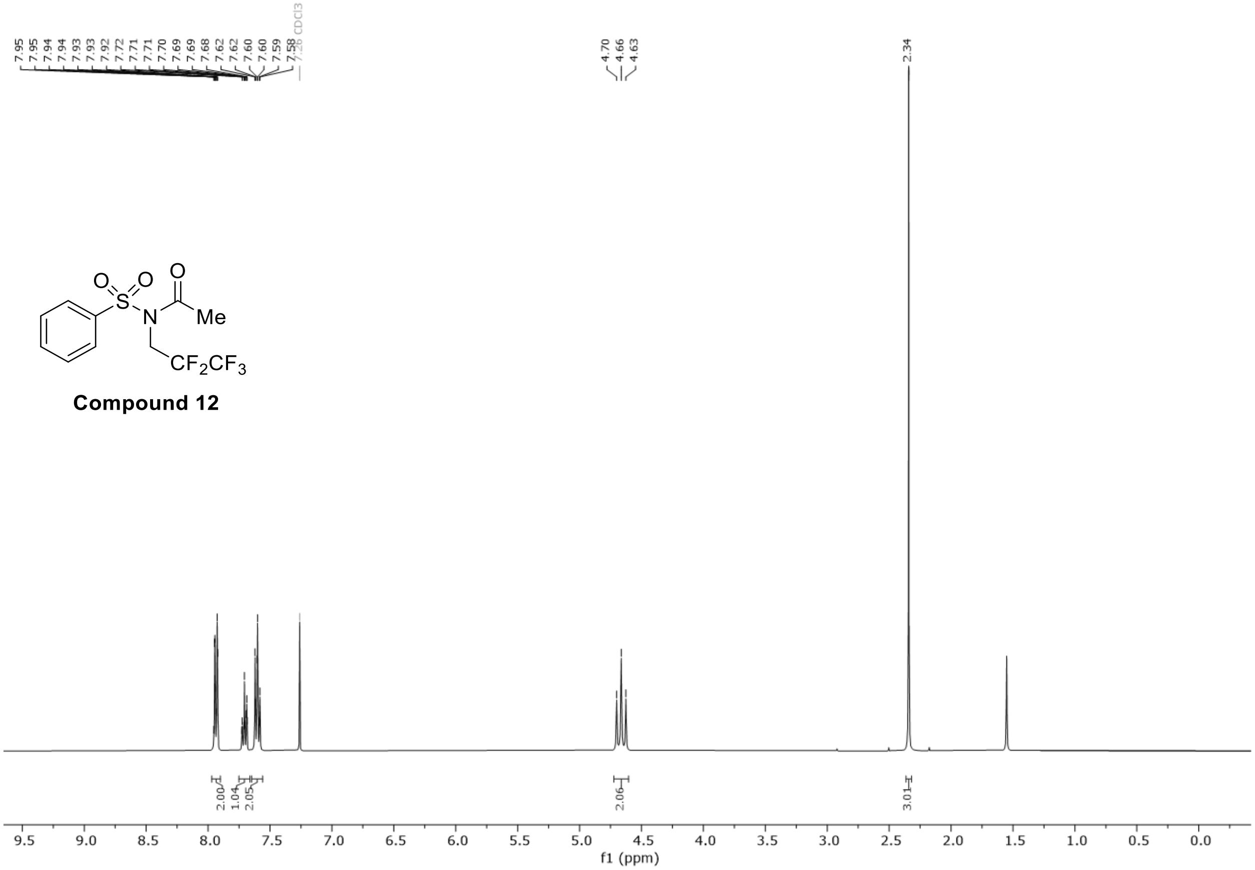

Compound 12

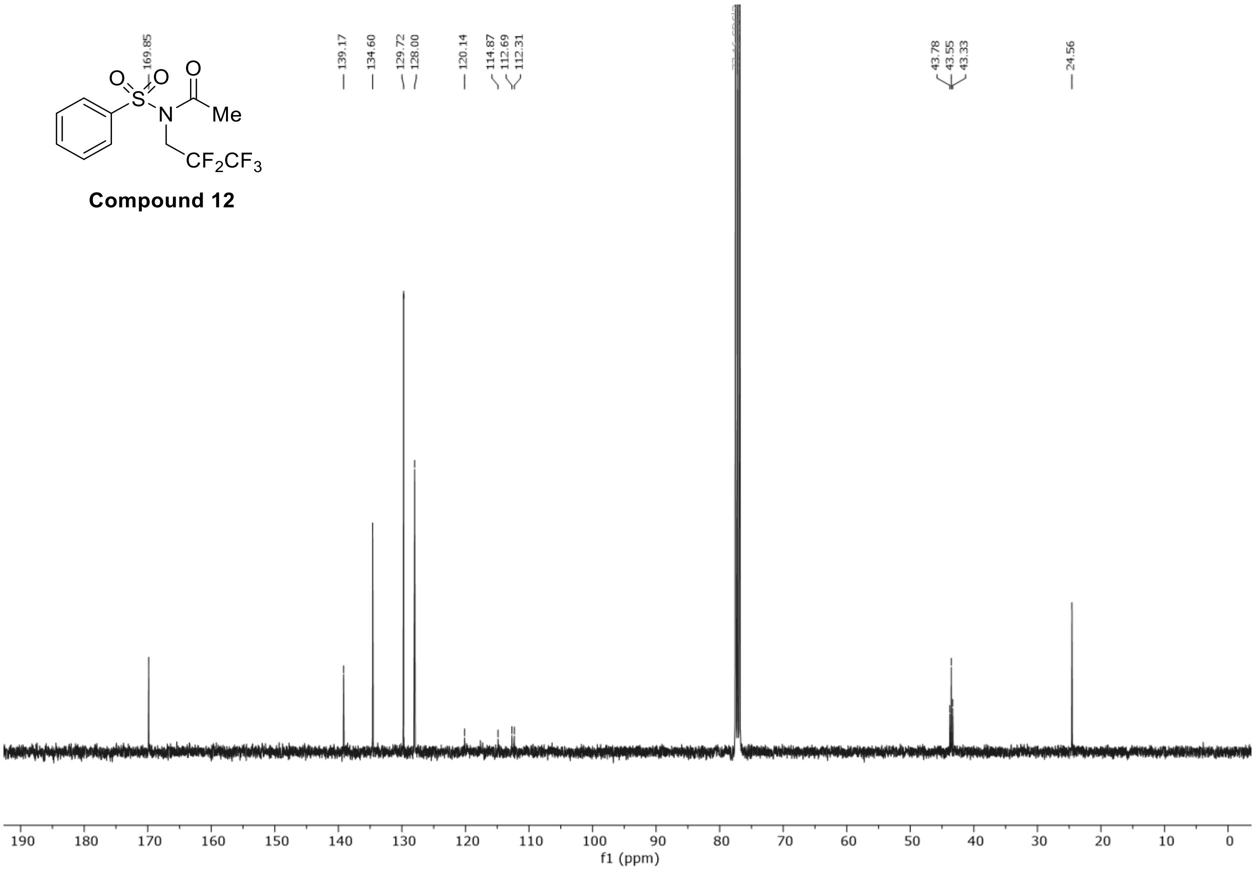

Compound 12

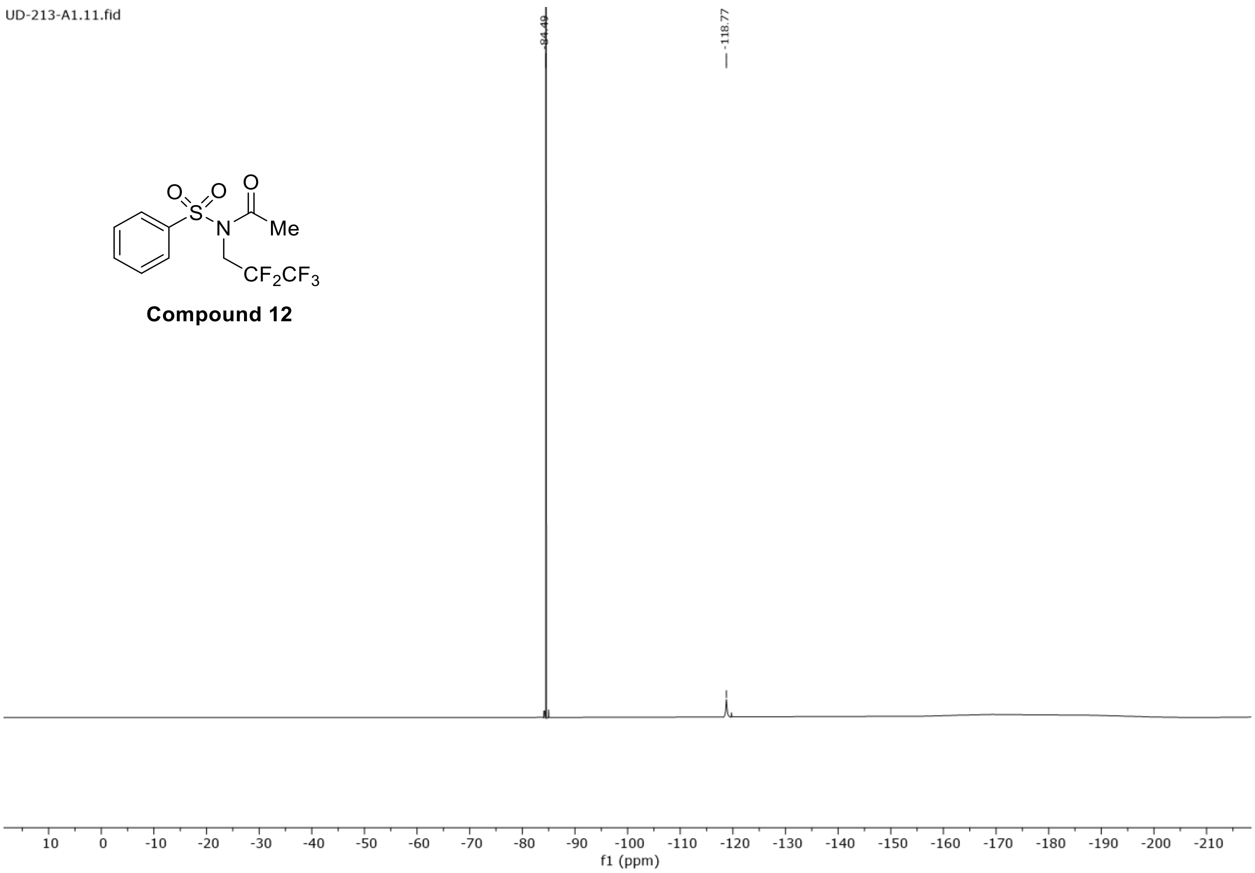
UD-213-A1.11.fid
-84.49
-118.77
O
O
O
S
N
Me
CF_2CF_3
10 0 -10 -20 -30 -40 -50 -60 -70 -80 -90 -100 -110 -120 -130 -140 -150 -160 -170 -180 -190 -200 -210
f1 (ppm)

Compound 12

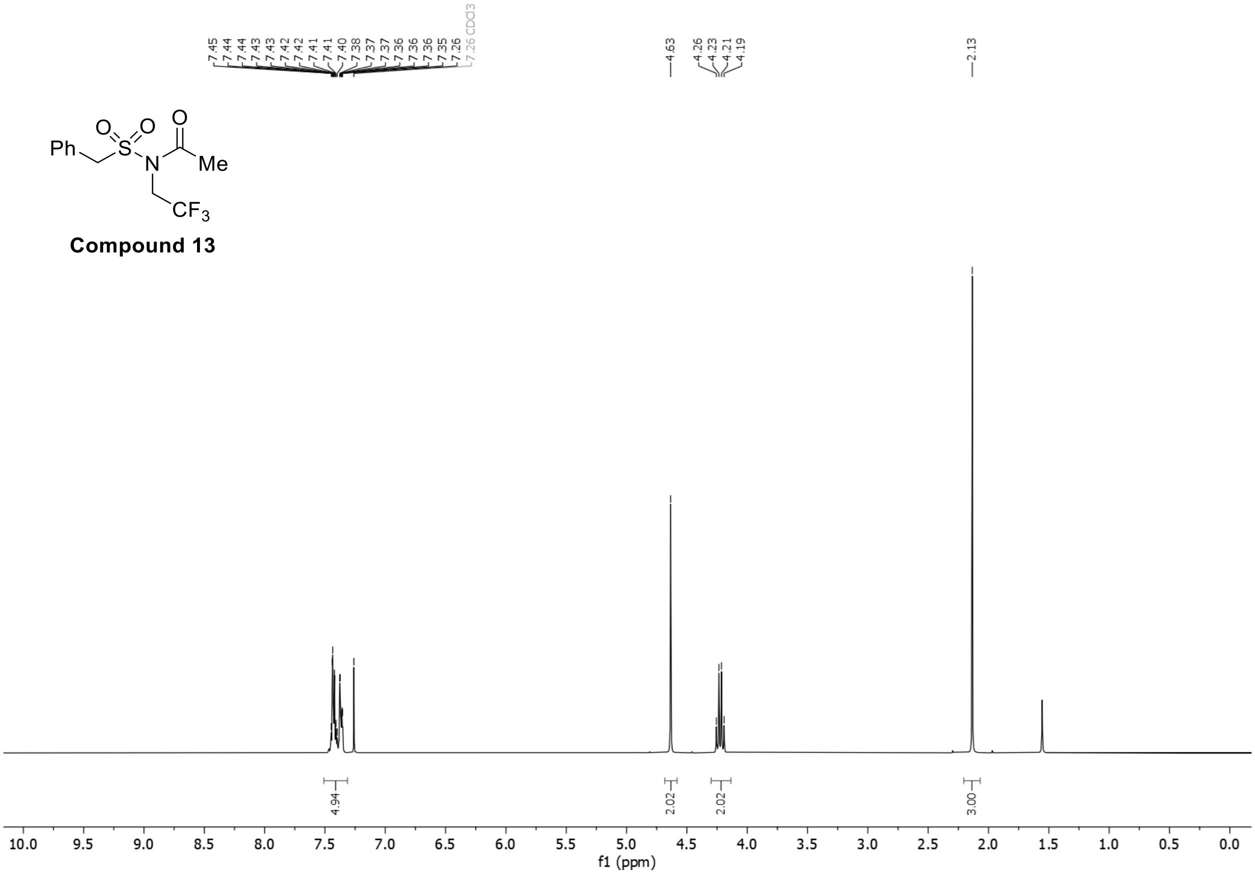
Compound 13
Ph
O
S
N
Me
CF3
7.45 7.44 7.44 7.43 7.43 7.42 7.42 7.42 7.41 7.41 7.40 7.38 7.37 7.37 7.36 7.36 7.36 7.36 7.35 7.26 7.26 CDCl3
4.63
4.26 4.23 4.21 4.19
2.13
4.94
2.02
2.02
3.00
f1 (ppm)

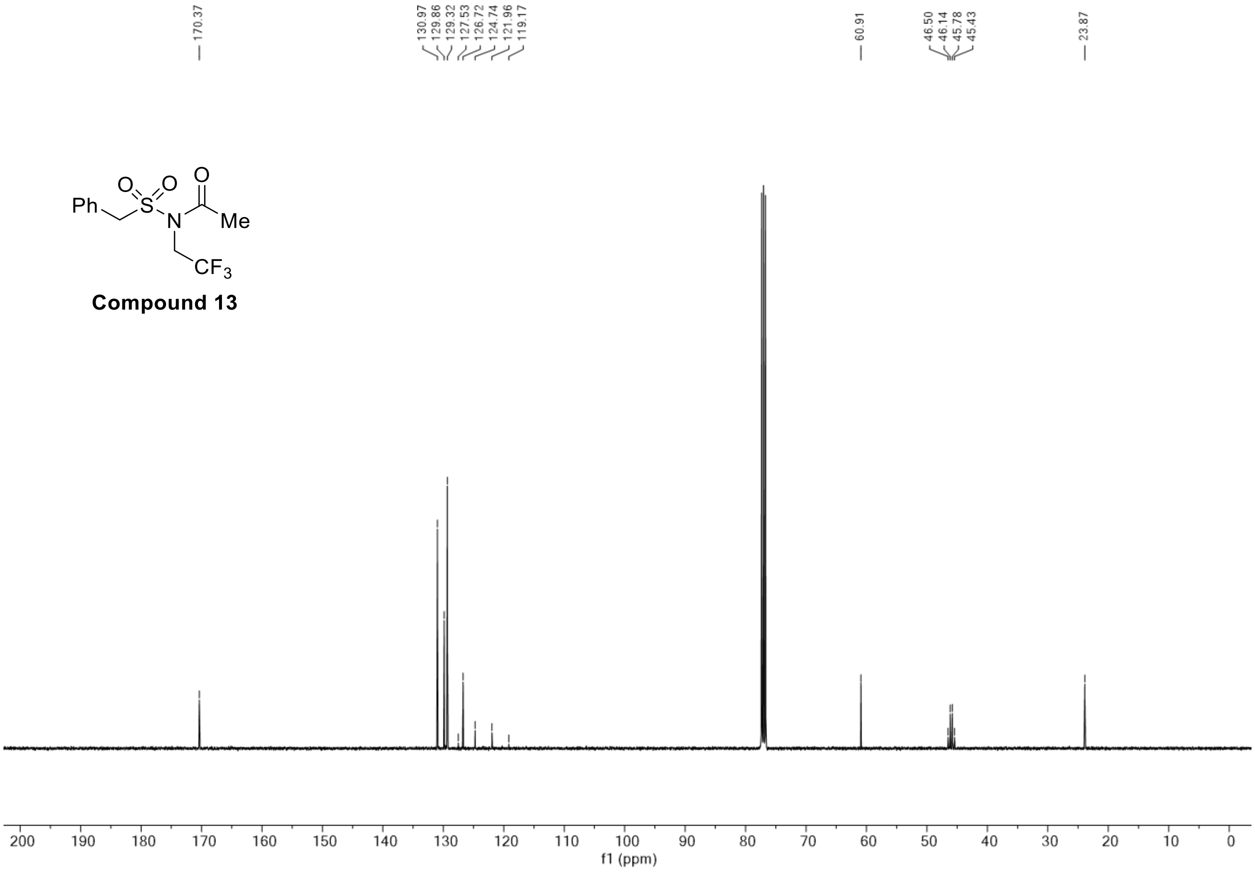
Compound 13
Ph
O
S
N
Me
CF3
170.37
130.97 129.86 129.32 127.53 126.72 124.74 121.96 119.17
60.91
46.50 46.14 45.78 45.43
23.87
f1 (ppm)

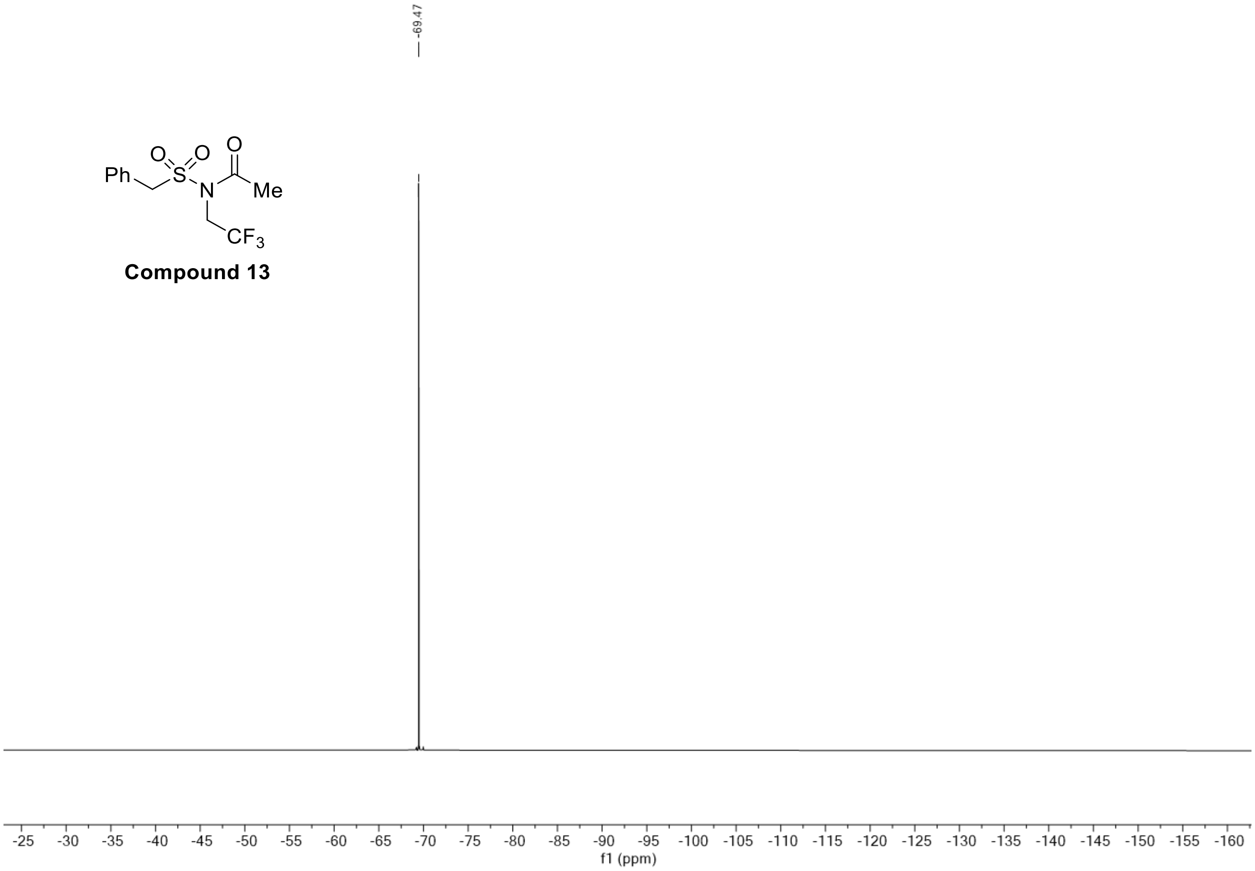

-69.47
Ph
O
S
O
N
O
Me
CF3
Compound 13
-25 -30 -35 -40 -45 -50 -55 -60 -65 -70 -75 -80 -85 -90 -95 -100 -105 -110 -115 -120 -125 -130 -135 -140 -145 -150 -155 -160
f1 (ppm)

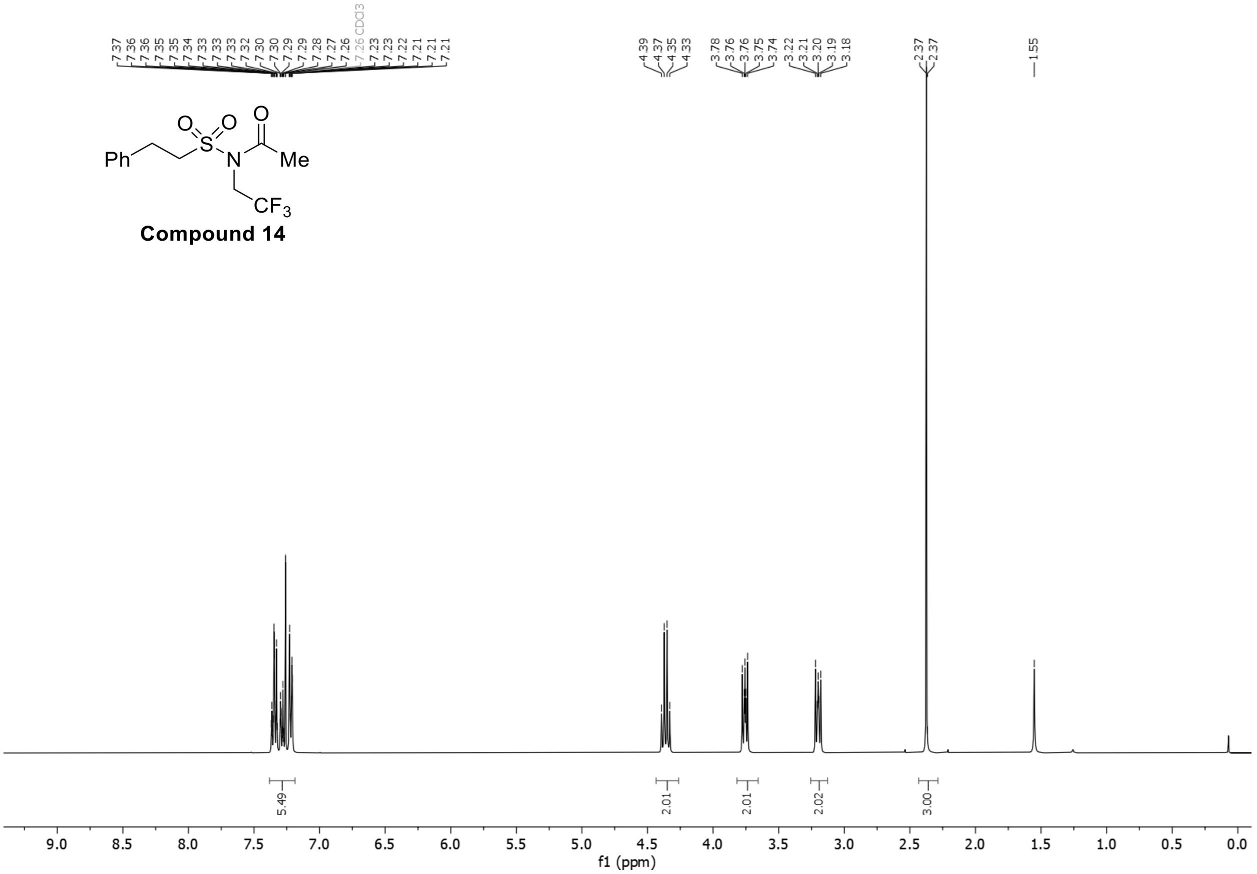

Compound 14

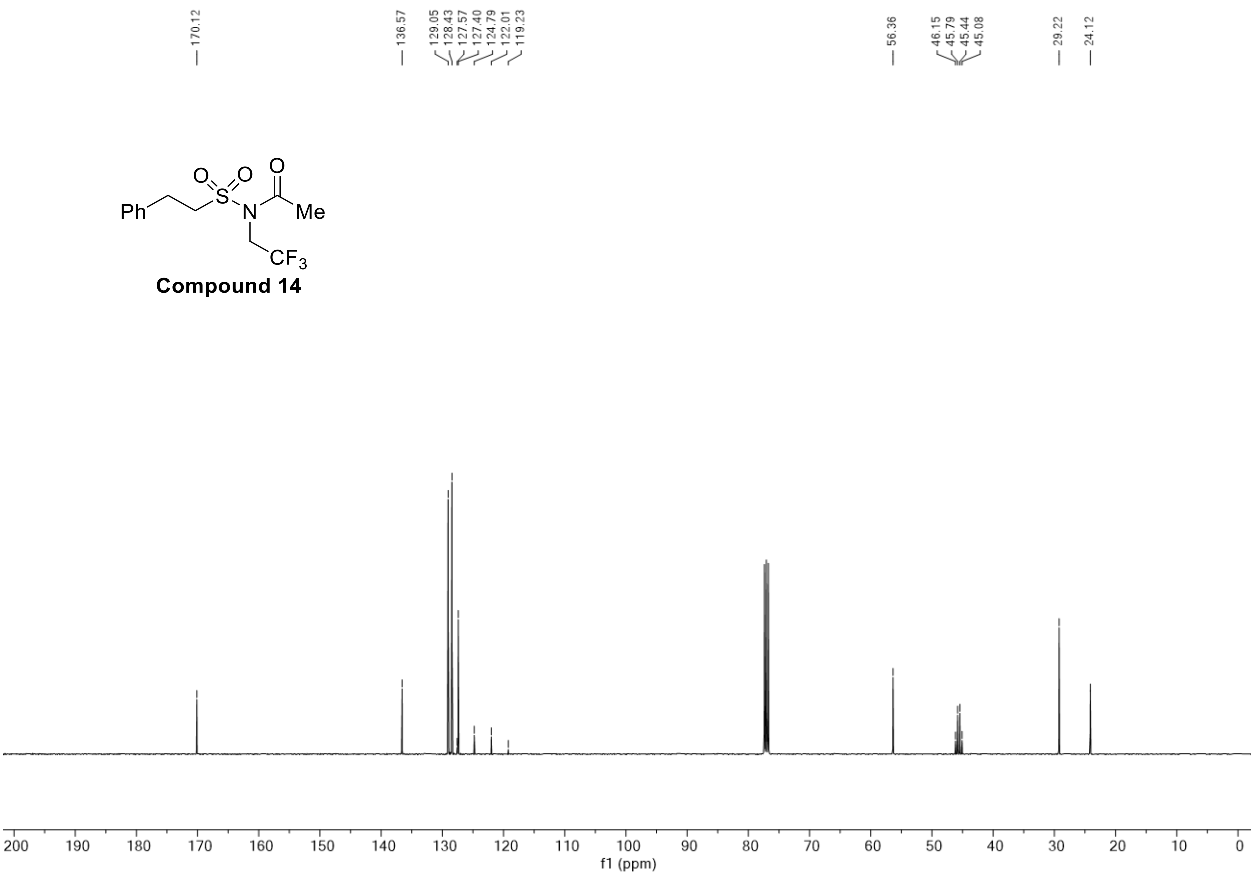

Compound 14

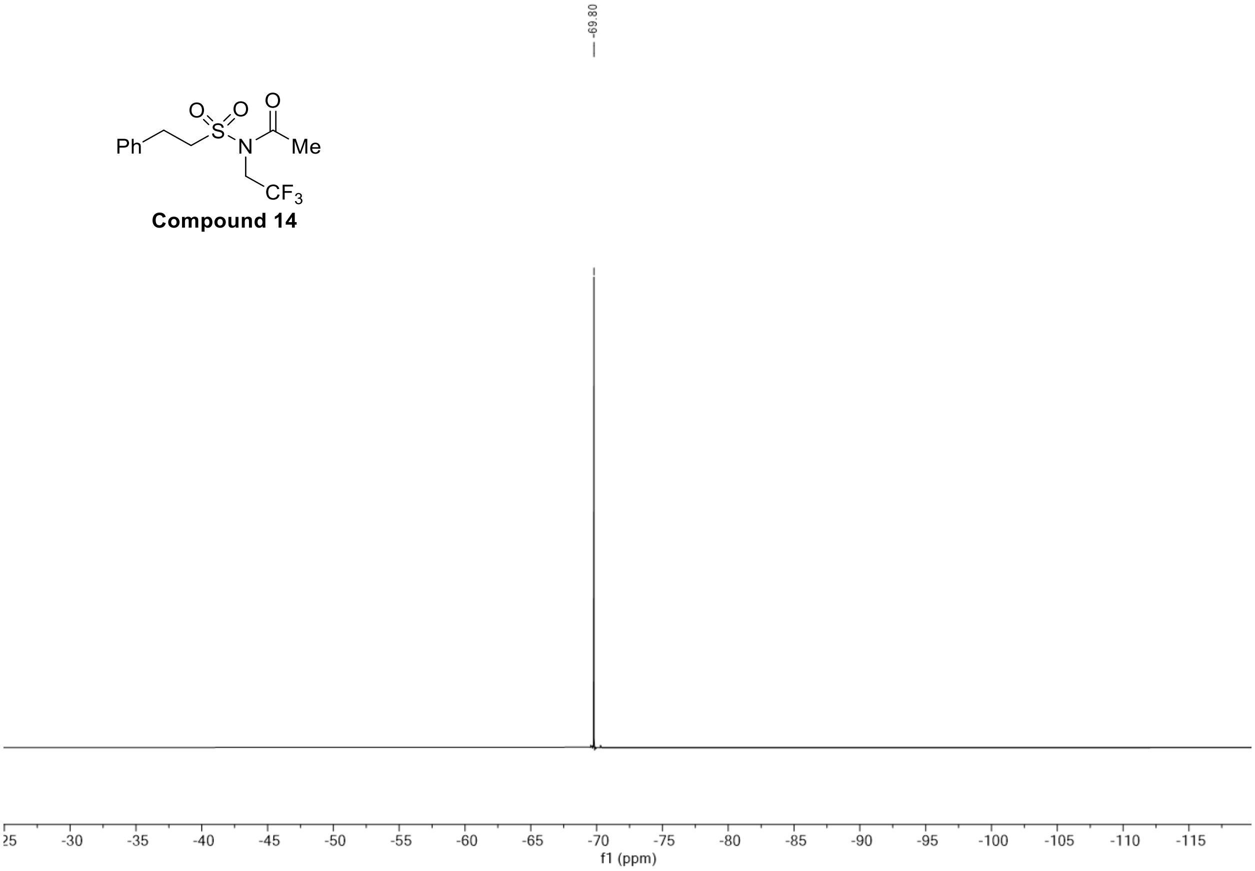

Compound 14

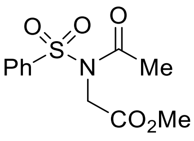

4S1

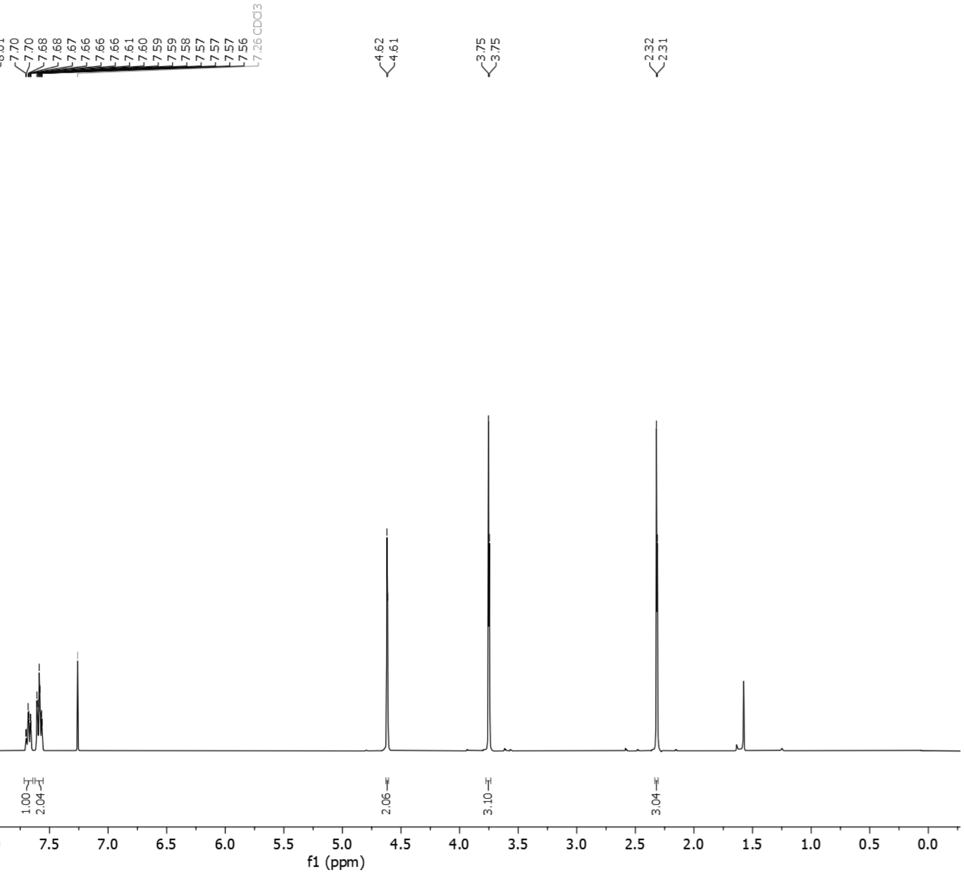

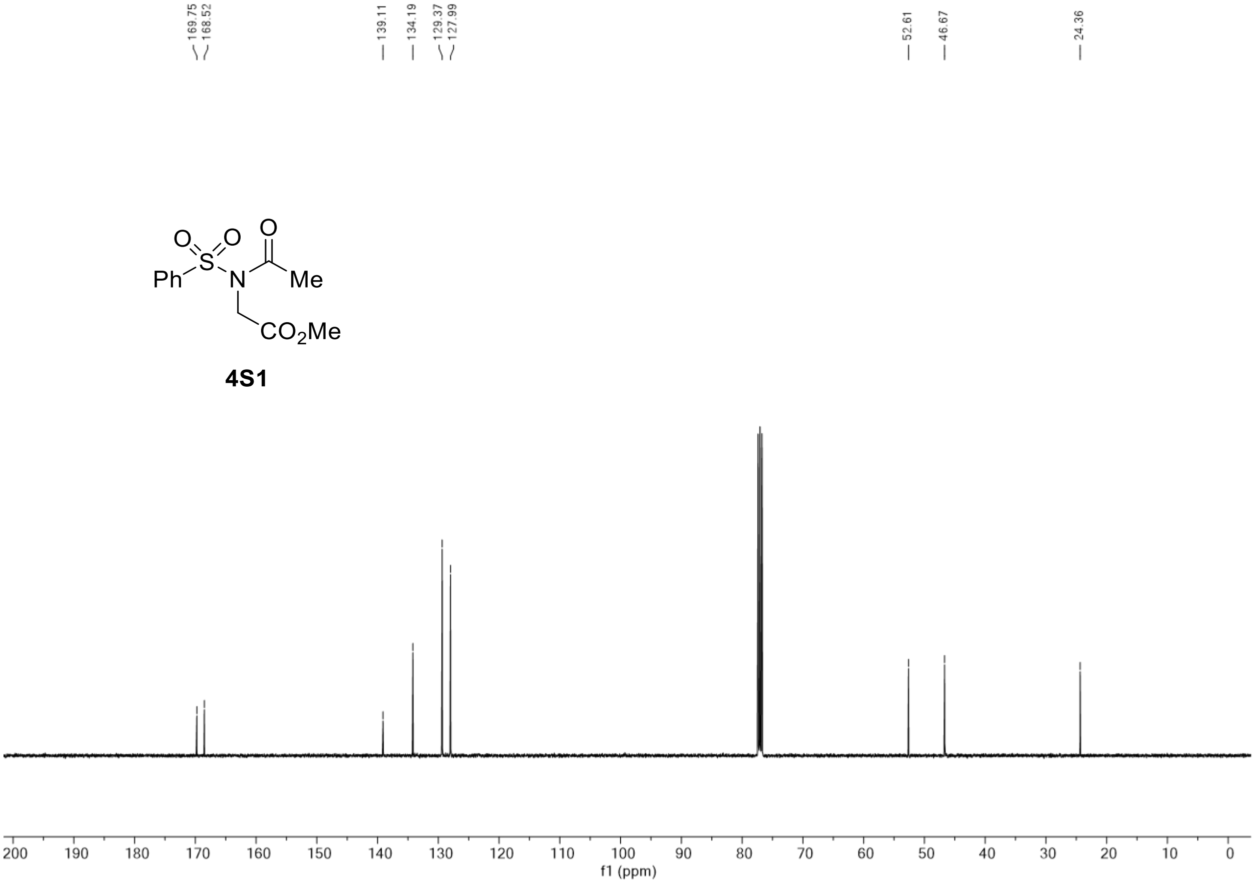
169.75
168.52
139.11
134.19
129.37
127.99
52.61
46.67
24.36
O
O
S
Ph
N
O
Me
CO2Me
4S1
200 190 180 170 160 150 140 130 120 110 100 90 80 70 60 50 40 30 20 10 0
f1 (ppm)

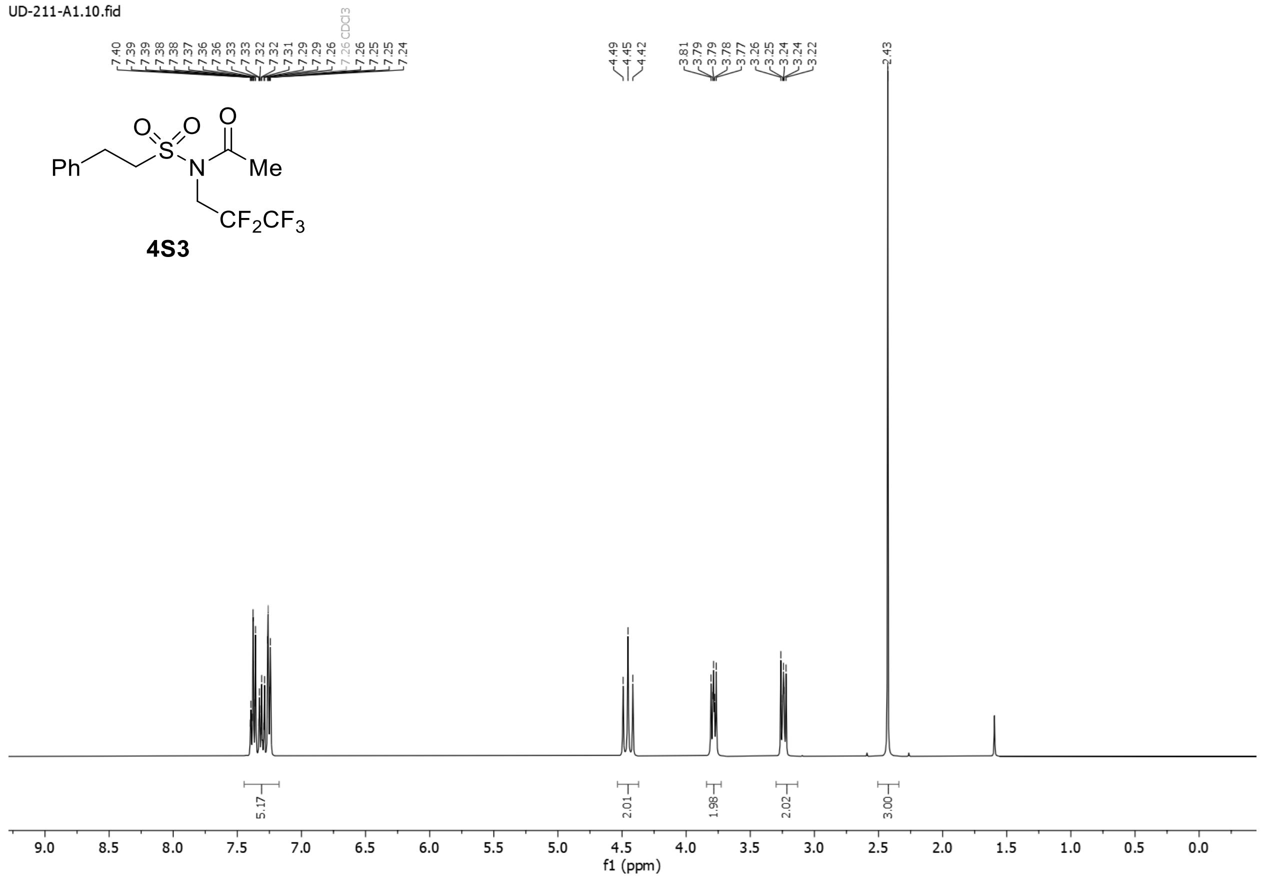
UD-211-A1.10.fid
4S3
f1 (ppm)

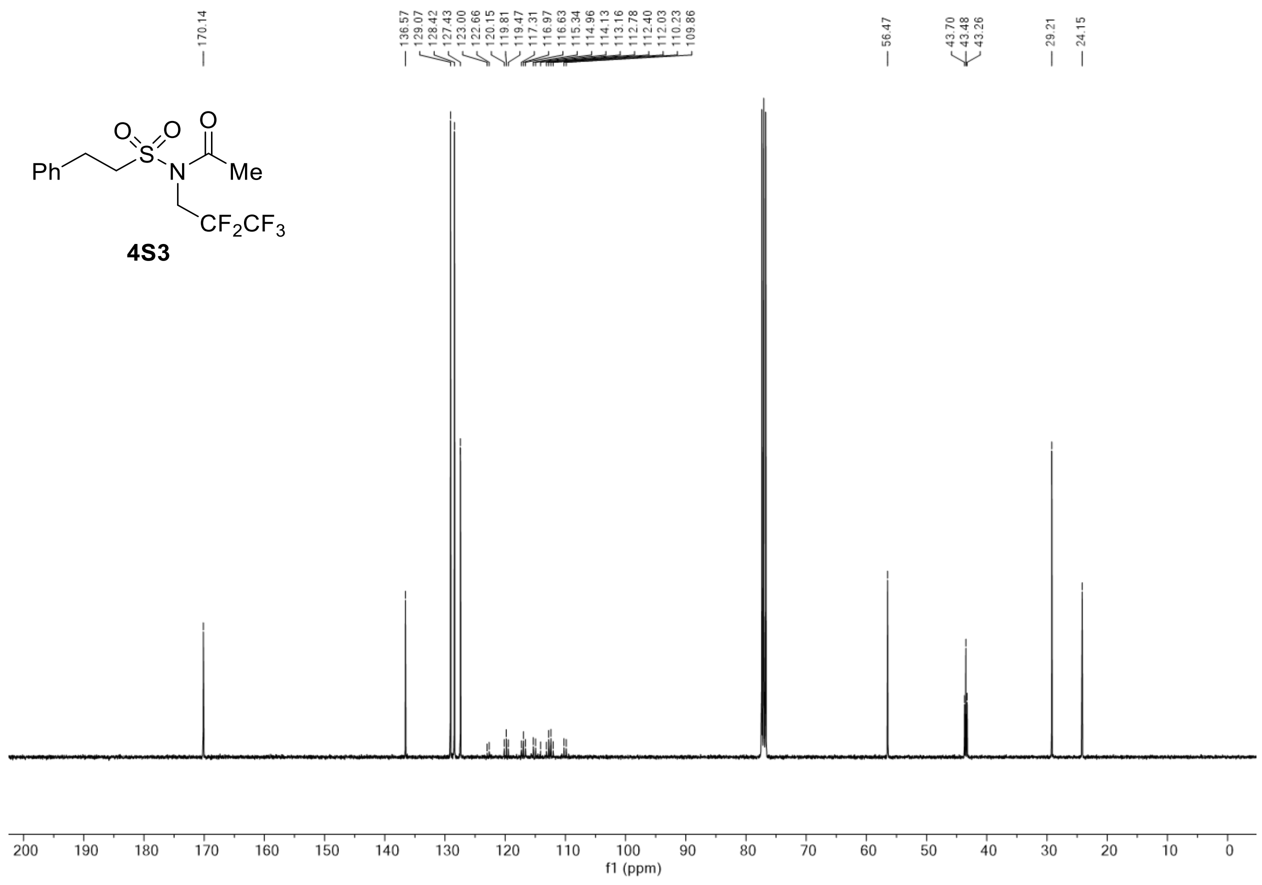
4S3
f1 (ppm)

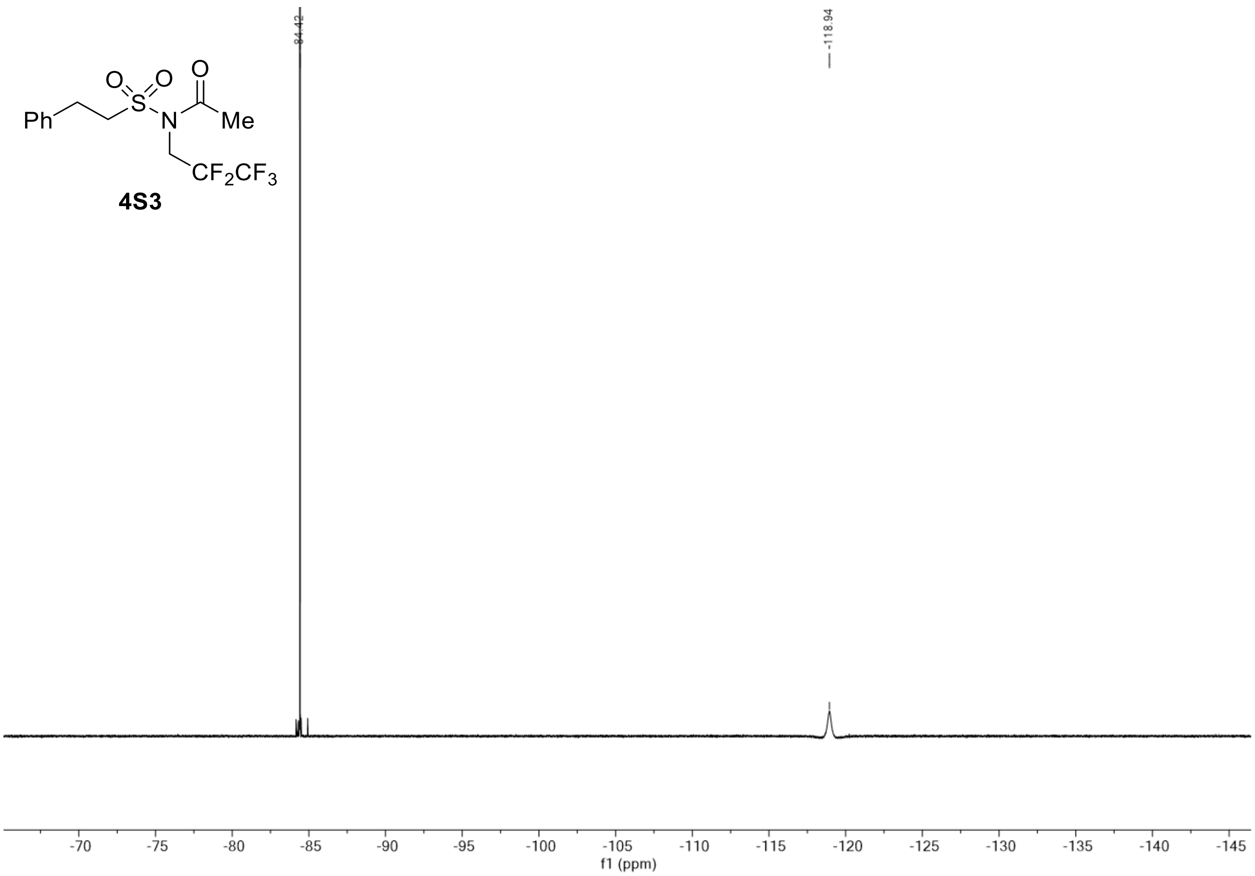

Ph
O
S
O
N
O
Me
CF2CF3
-84.42
-118.94
-70
-75
-80
-85
-90
-95
-100
-105
-110
-115
-120
-125
-130
-135
-140
-145
f1 (ppm)

4S3

3.22.8 <u>SuFA linker **S15**</u>

<u>Intermediate **S15c**</u>

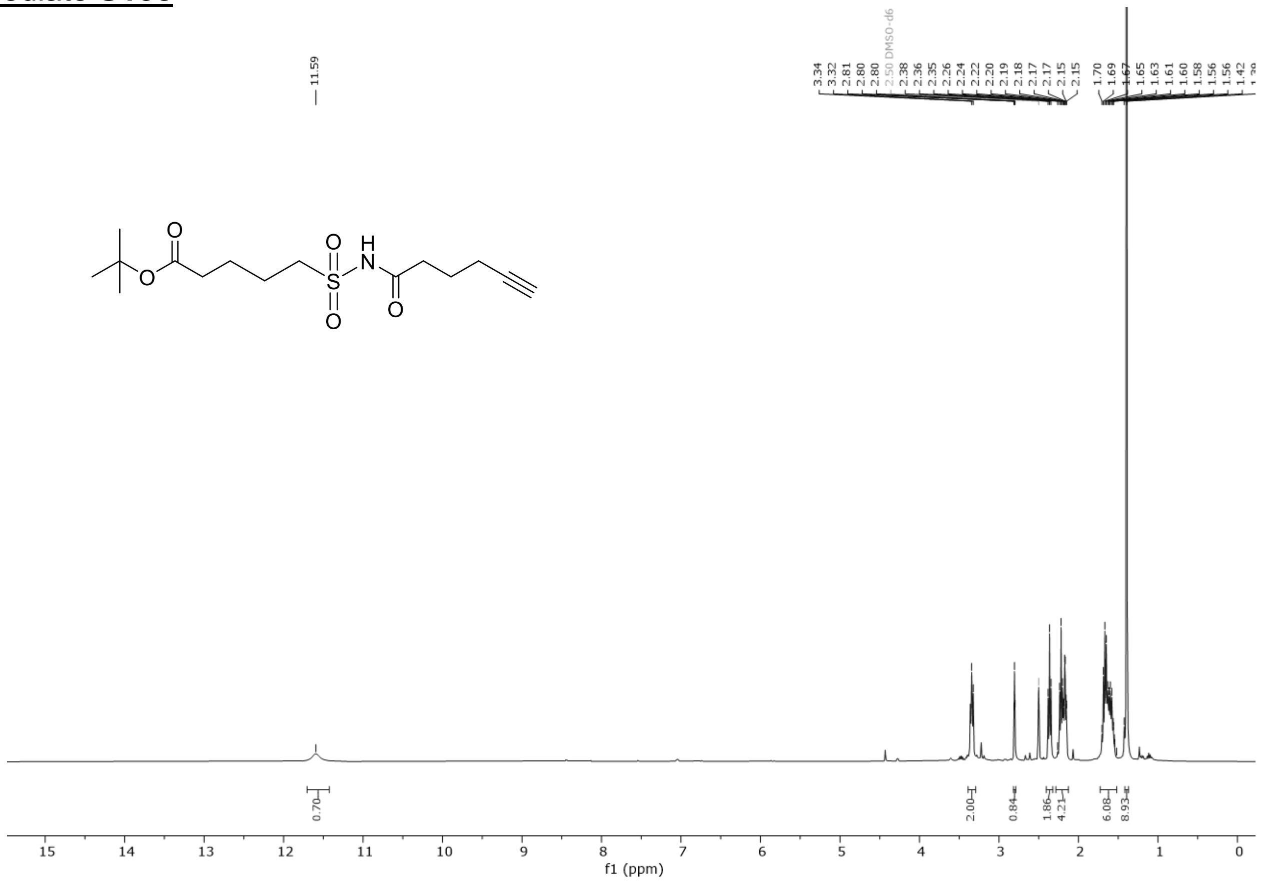

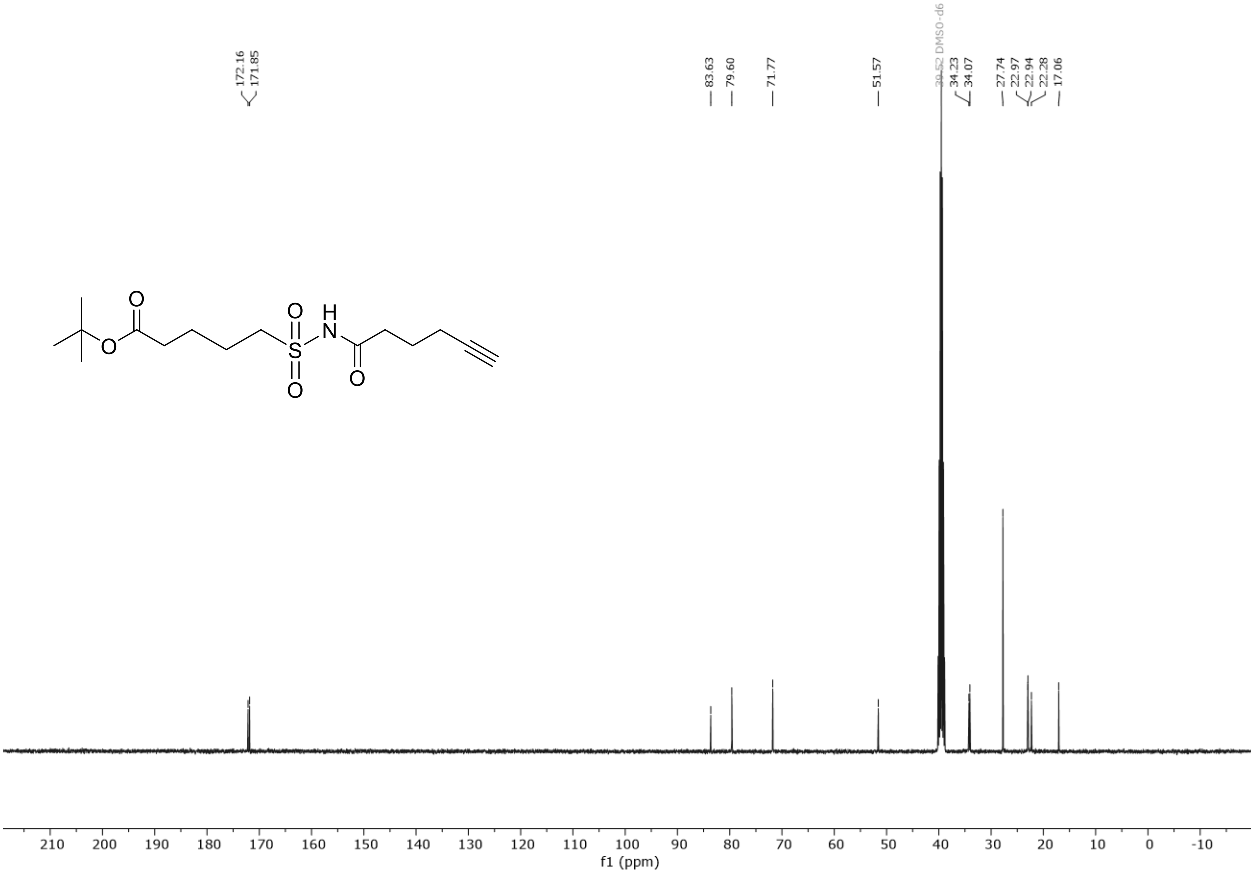
172.16
171.85
83.63
79.60
71.77
51.57
39.52 DMSO-d6
34.23
34.07
27.74
22.97
22.94
22.28
17.06
210 200 190 180 170 160 150 140 130 120 110 100 90 80 70 60 50 40 30 20 10 0 -10
f1 (ppm)

SuFA linker **S15**

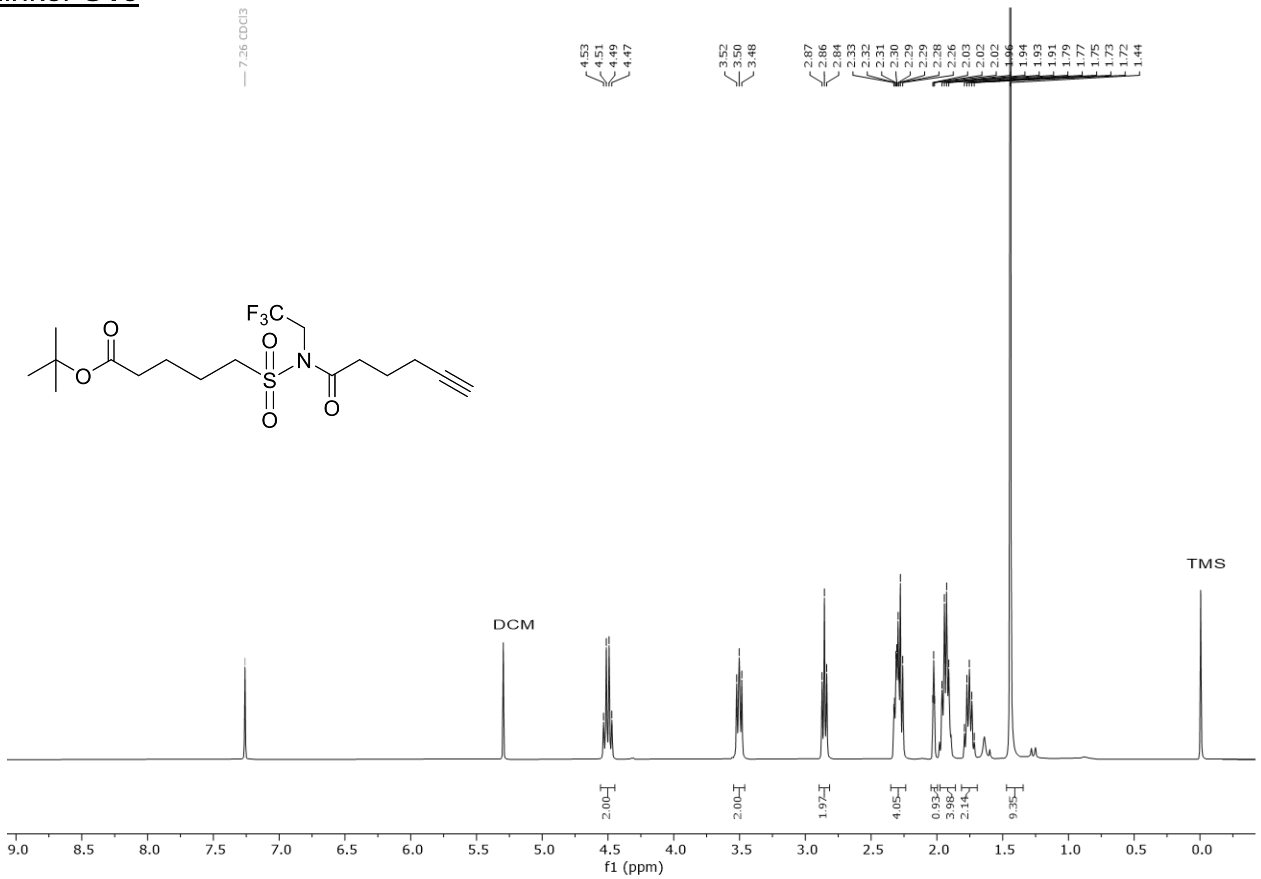

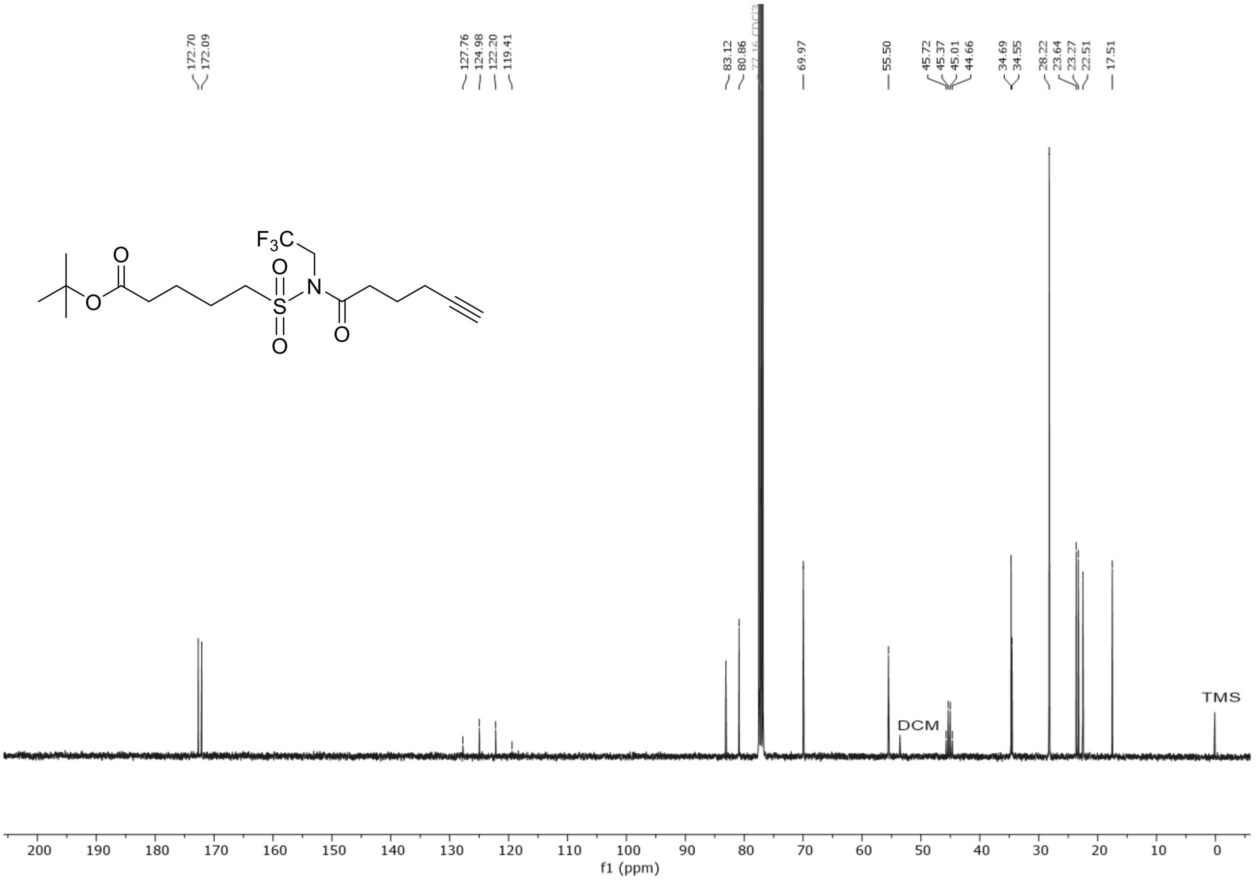

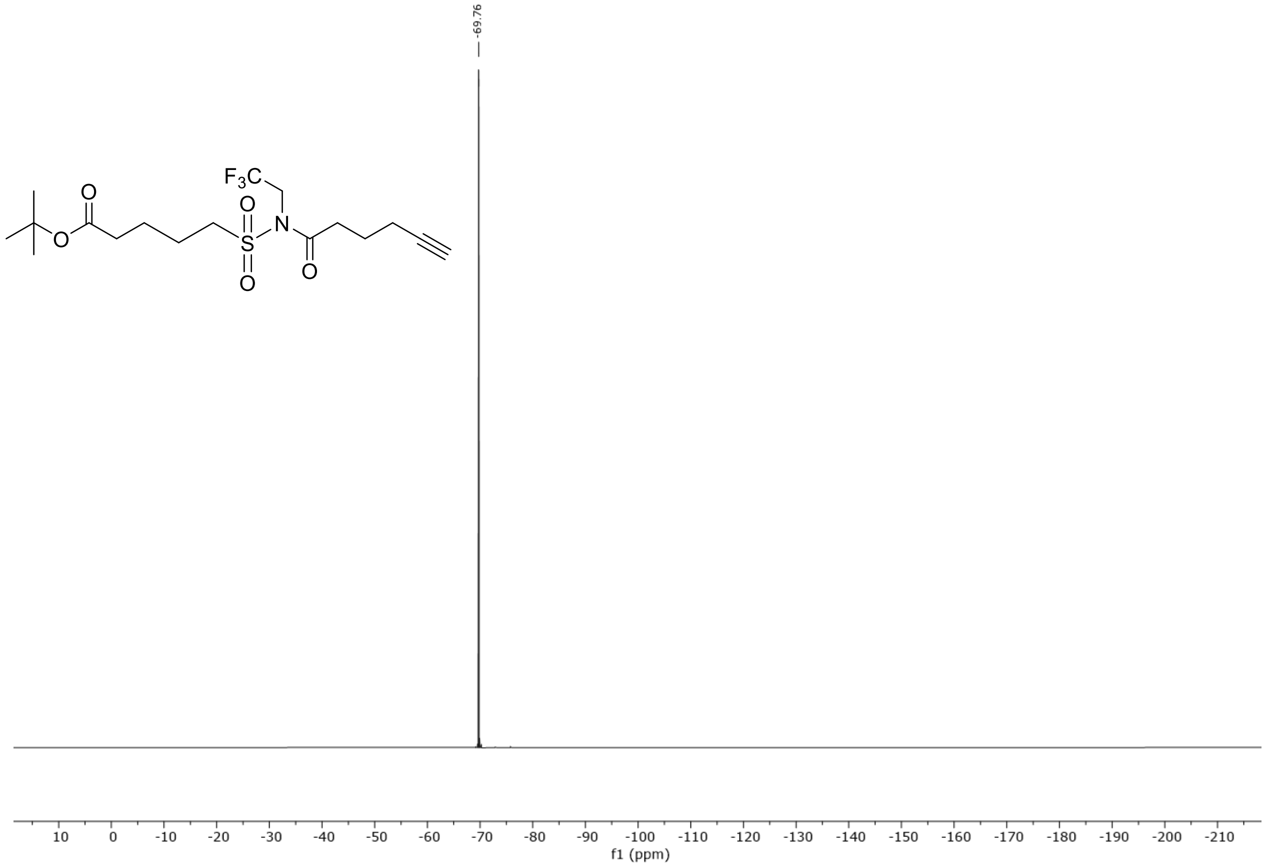
-69.76
f1 (ppm)

3.22.9 Compound **17** (FPR2 SuFA probe)

Intermediate **16** (FPR2 peptide precursor)

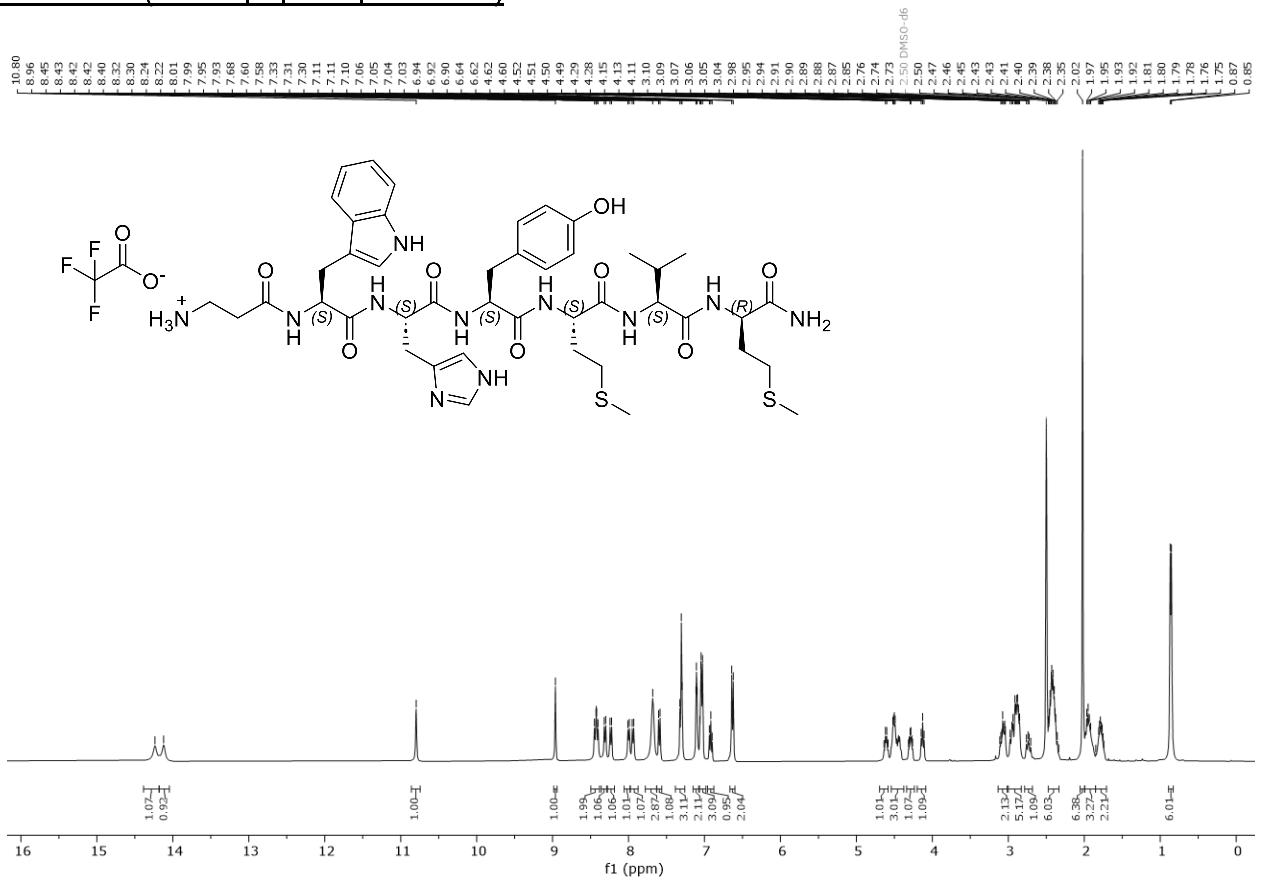

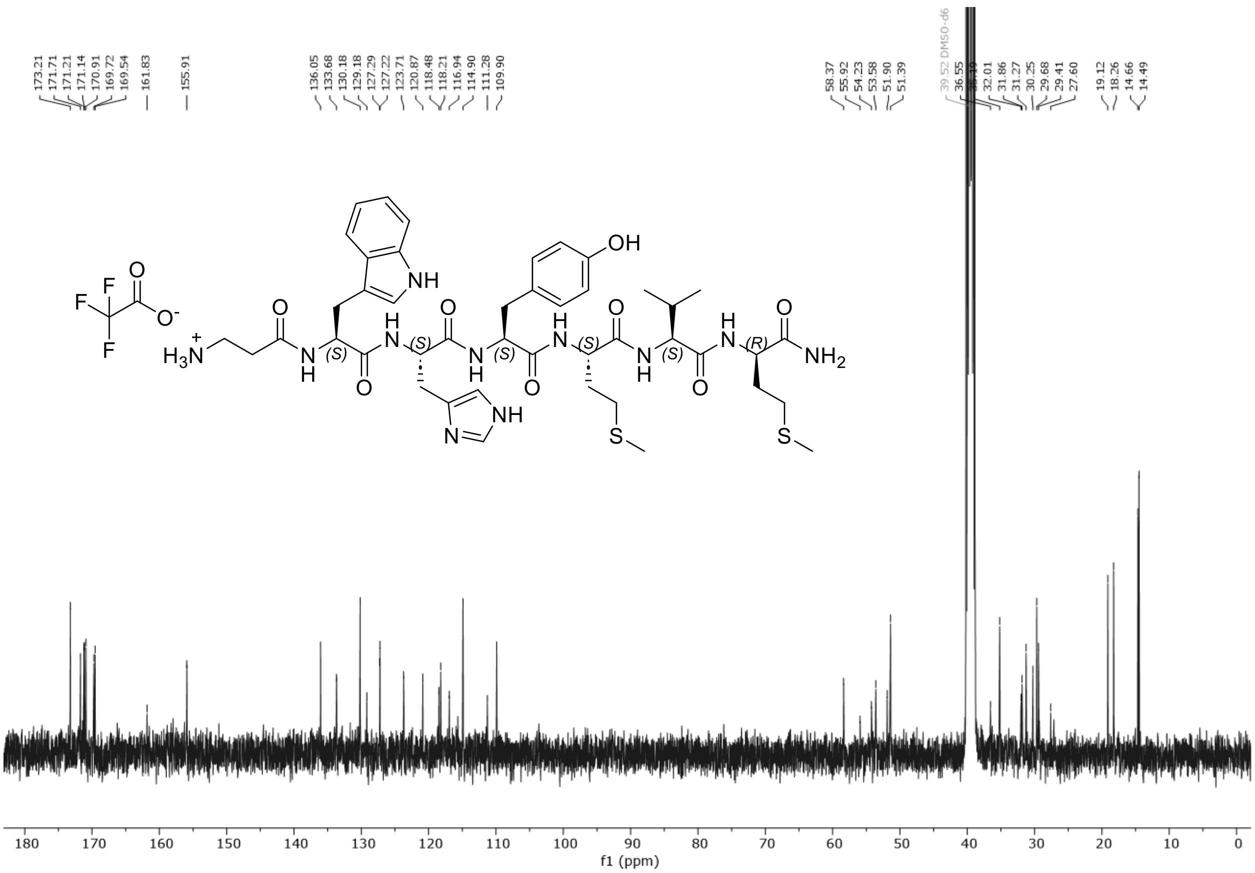

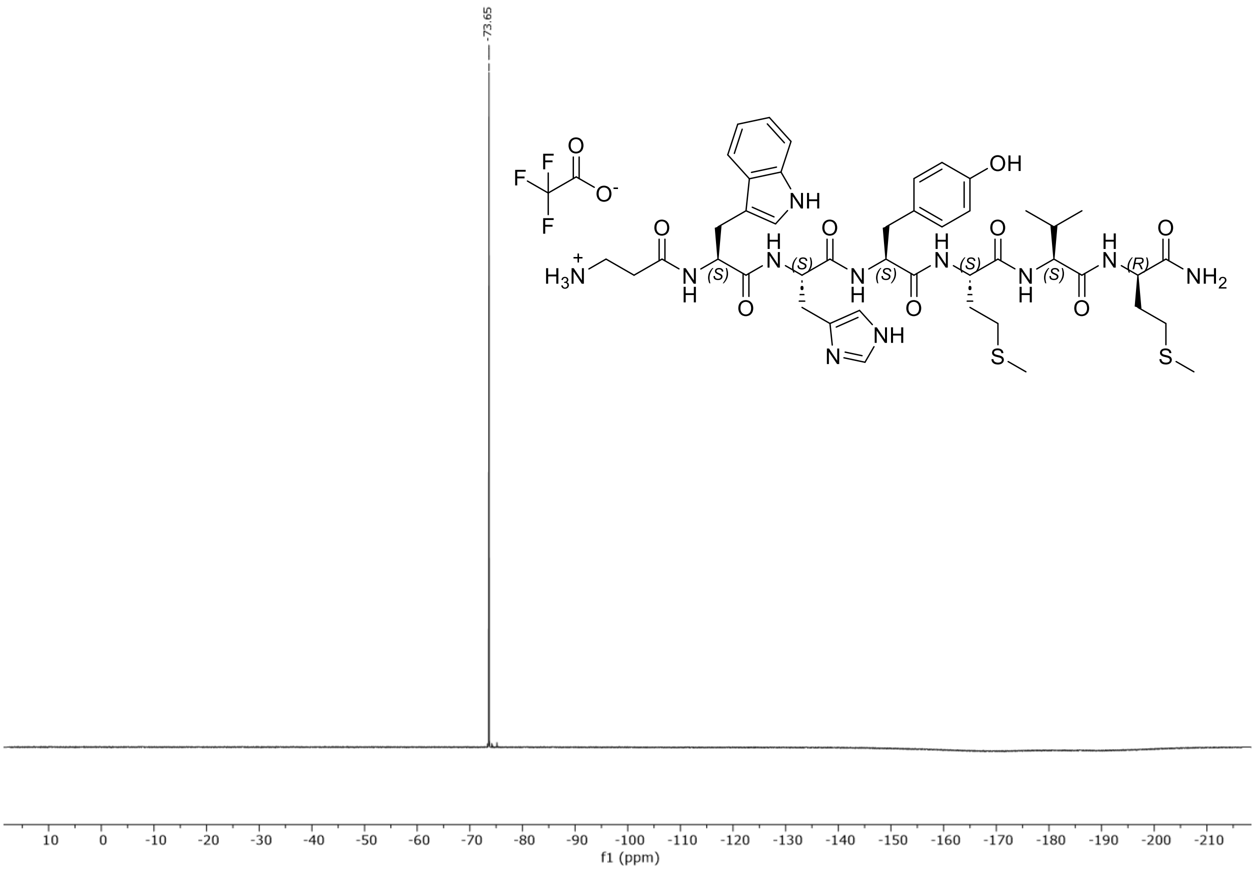

Compound **17** (FPR2 SuFA probe)

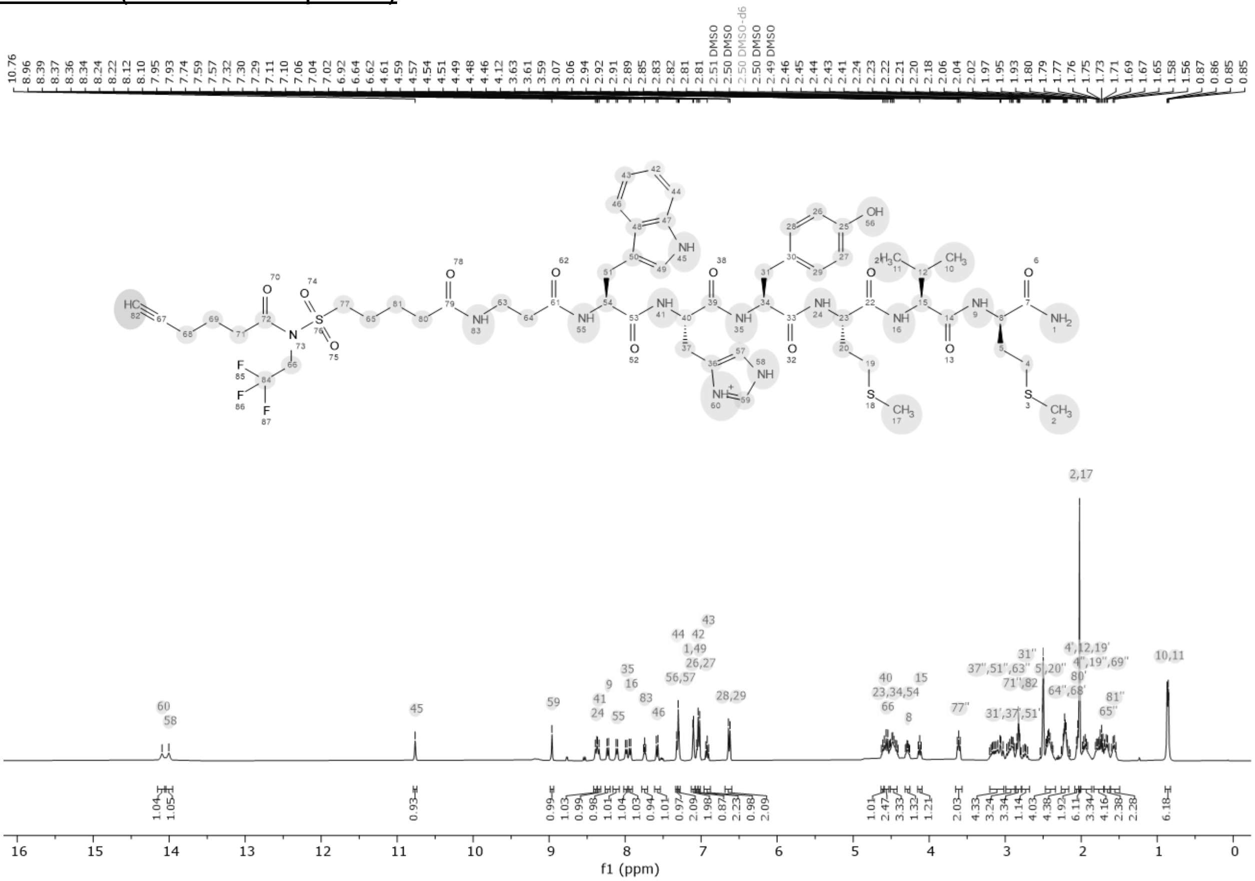

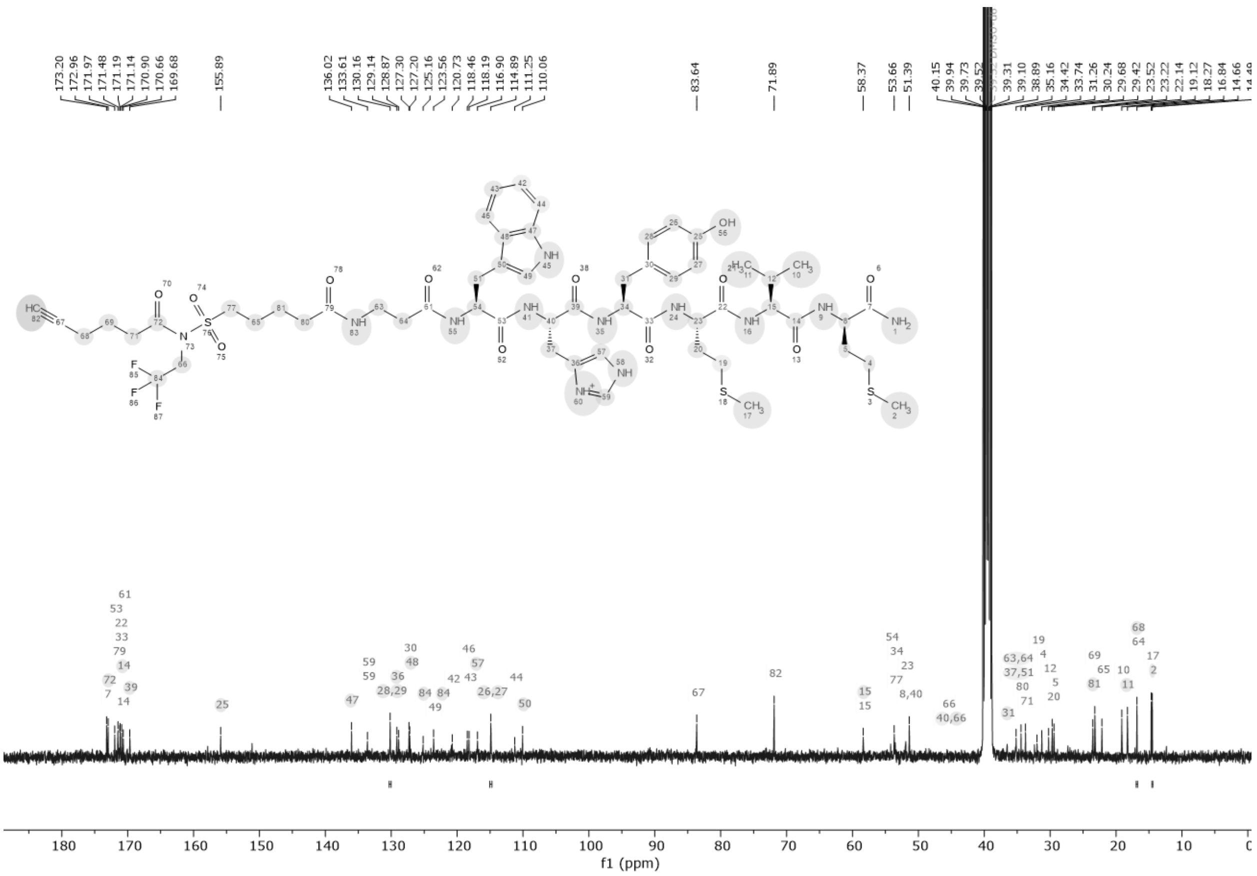

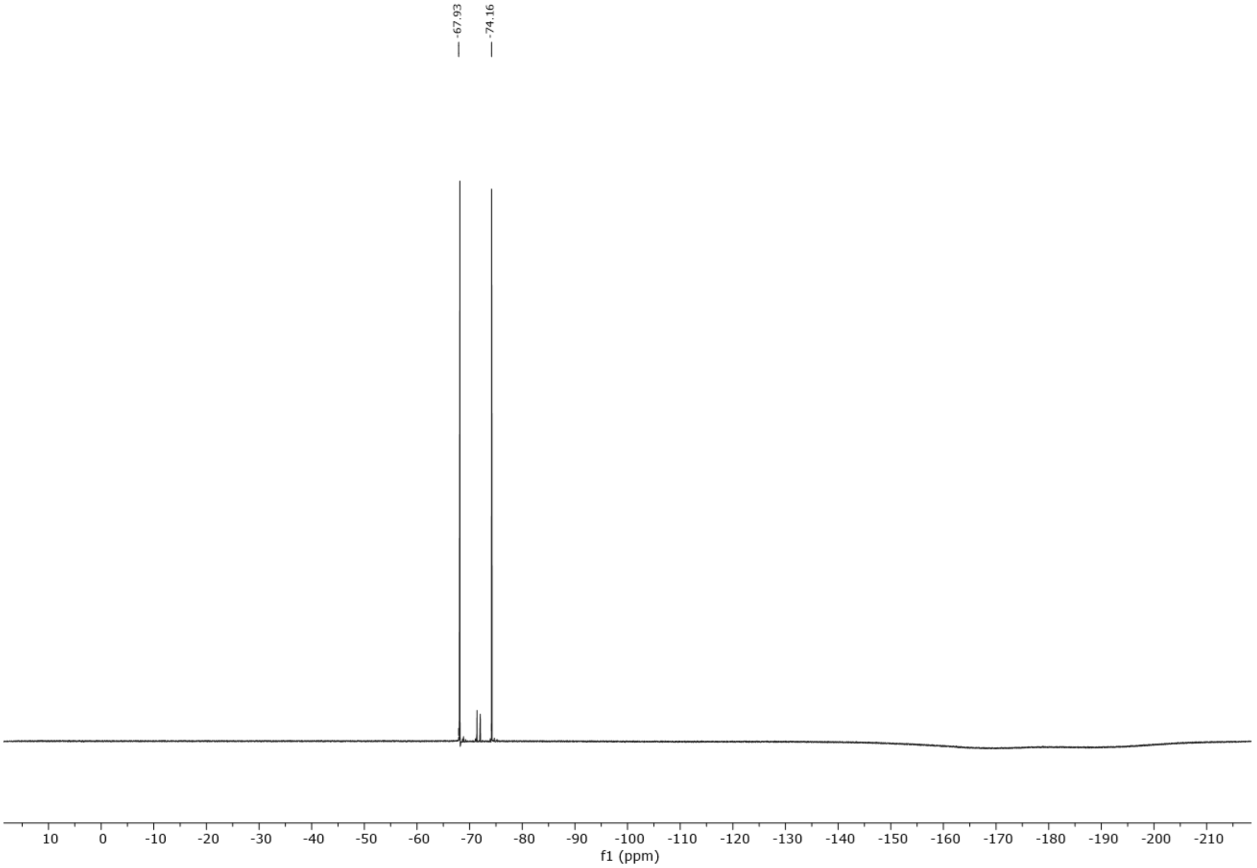

HSQC

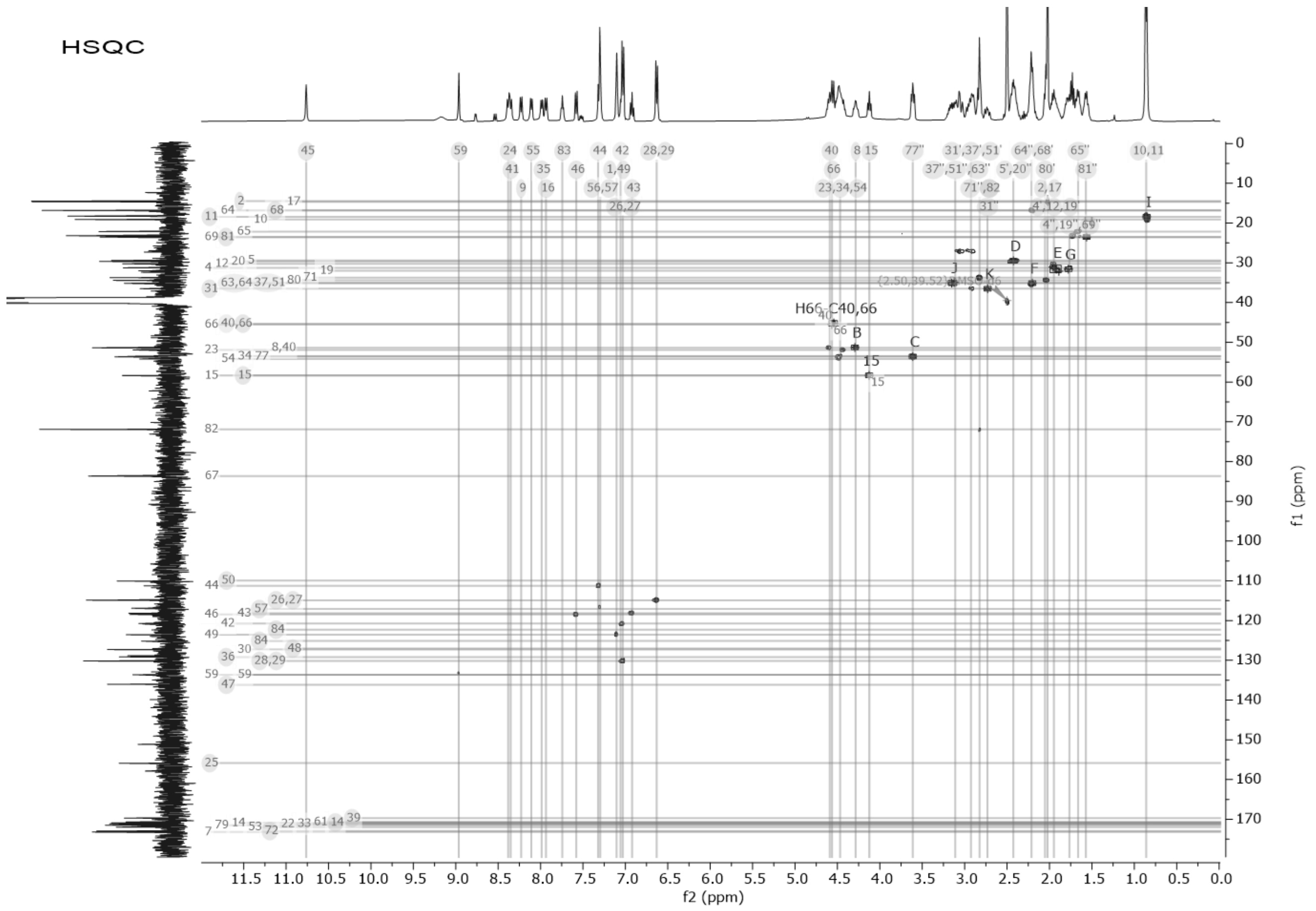

TOCSY

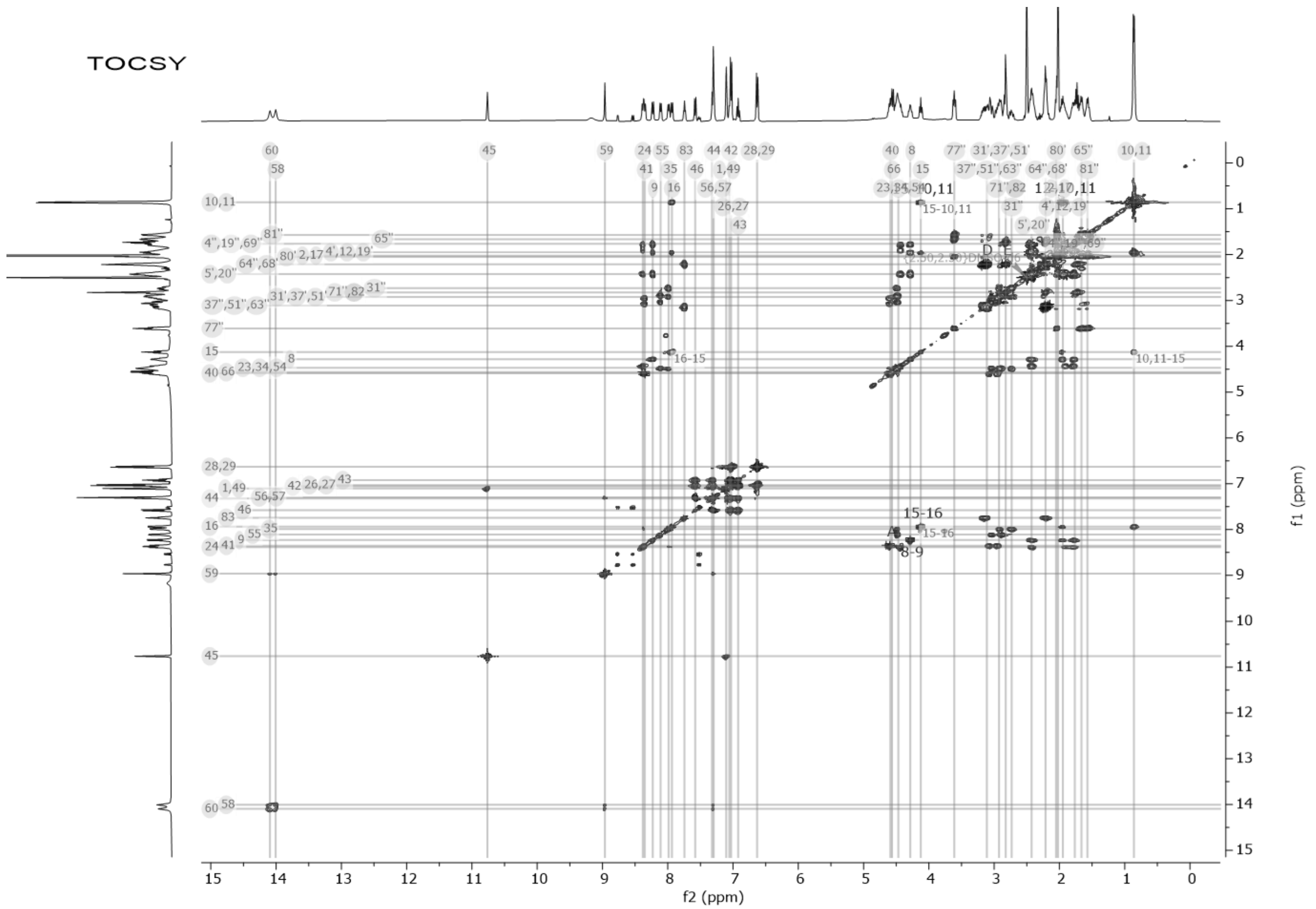

COSY

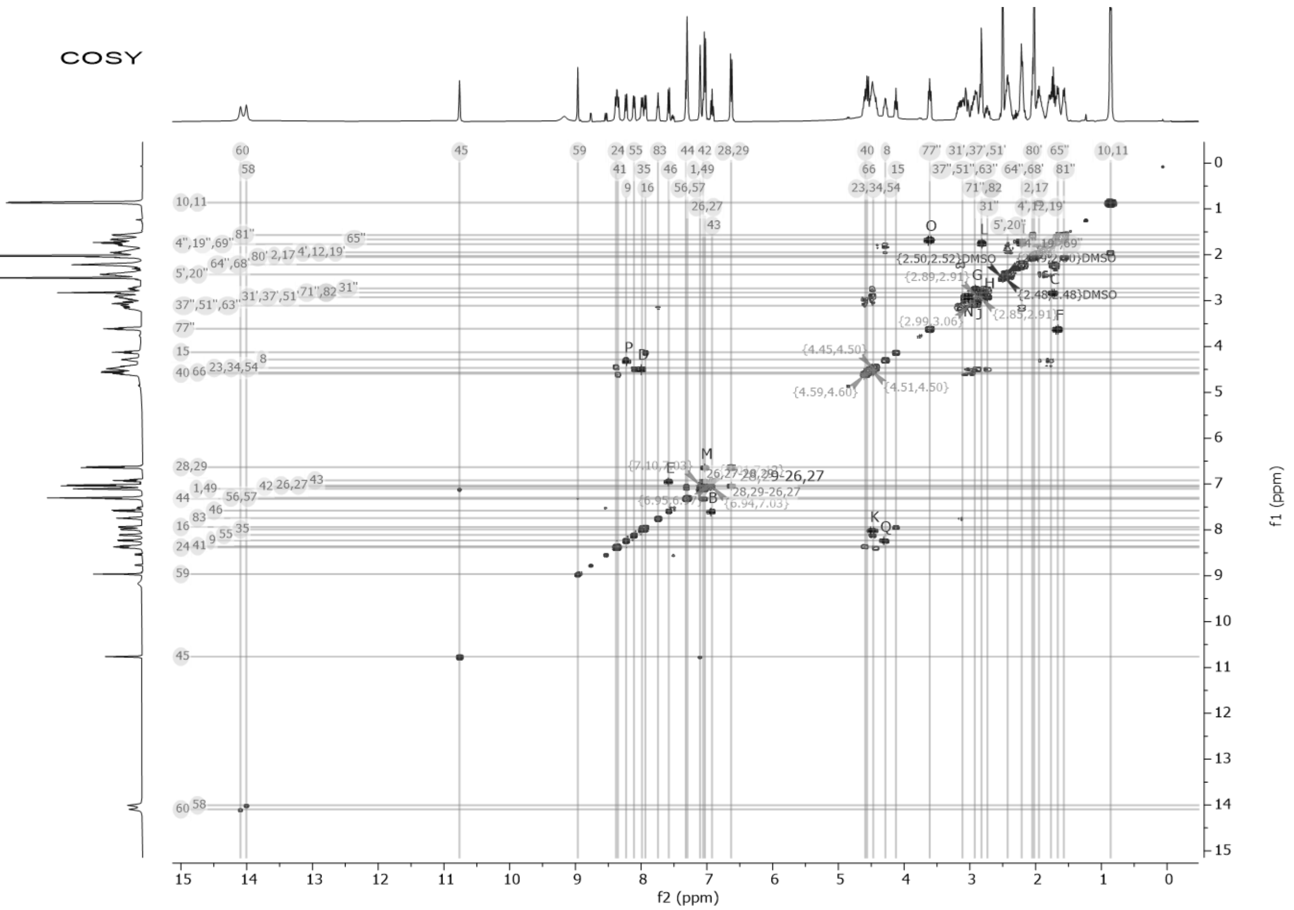

NOESY

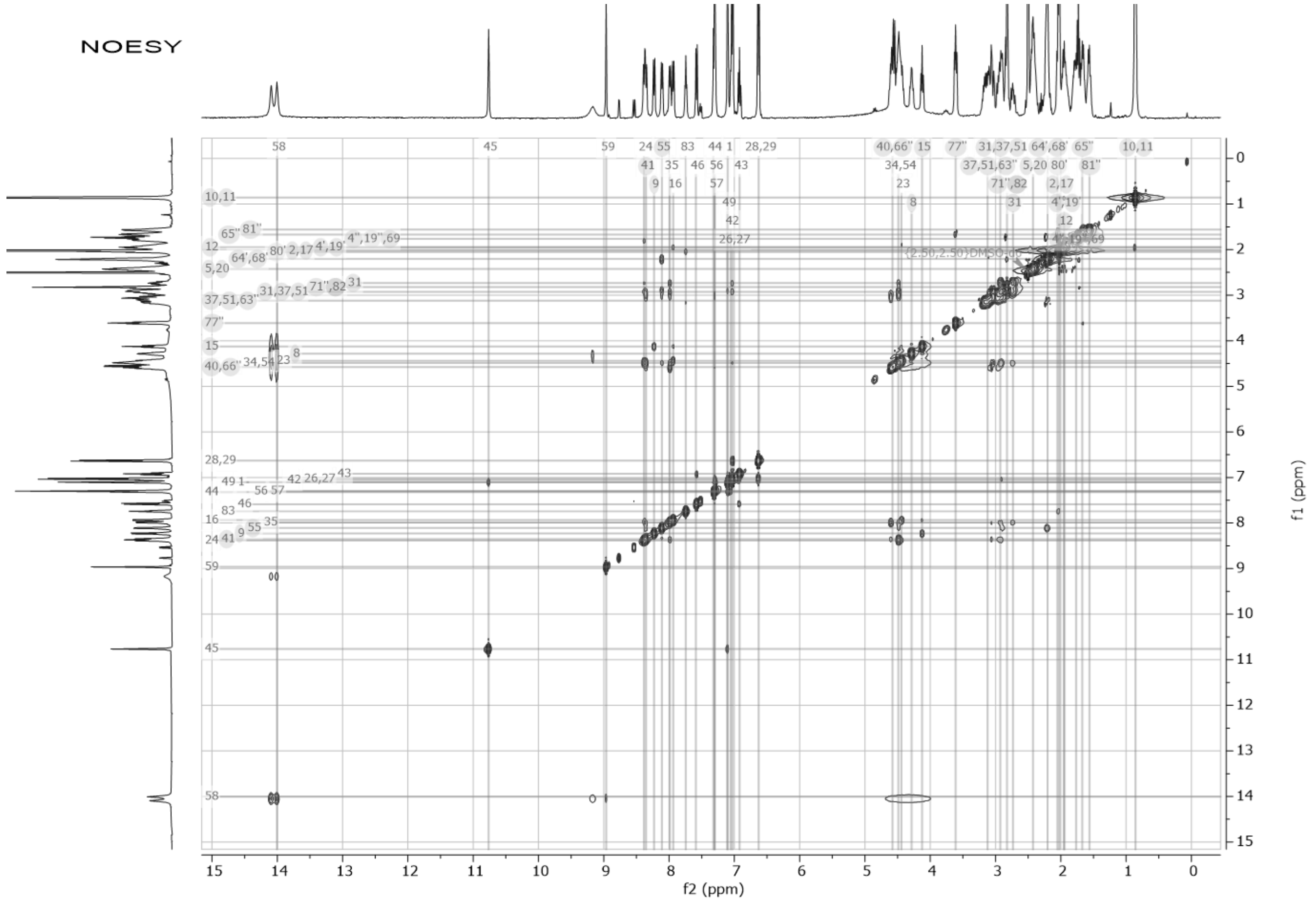

HMBC

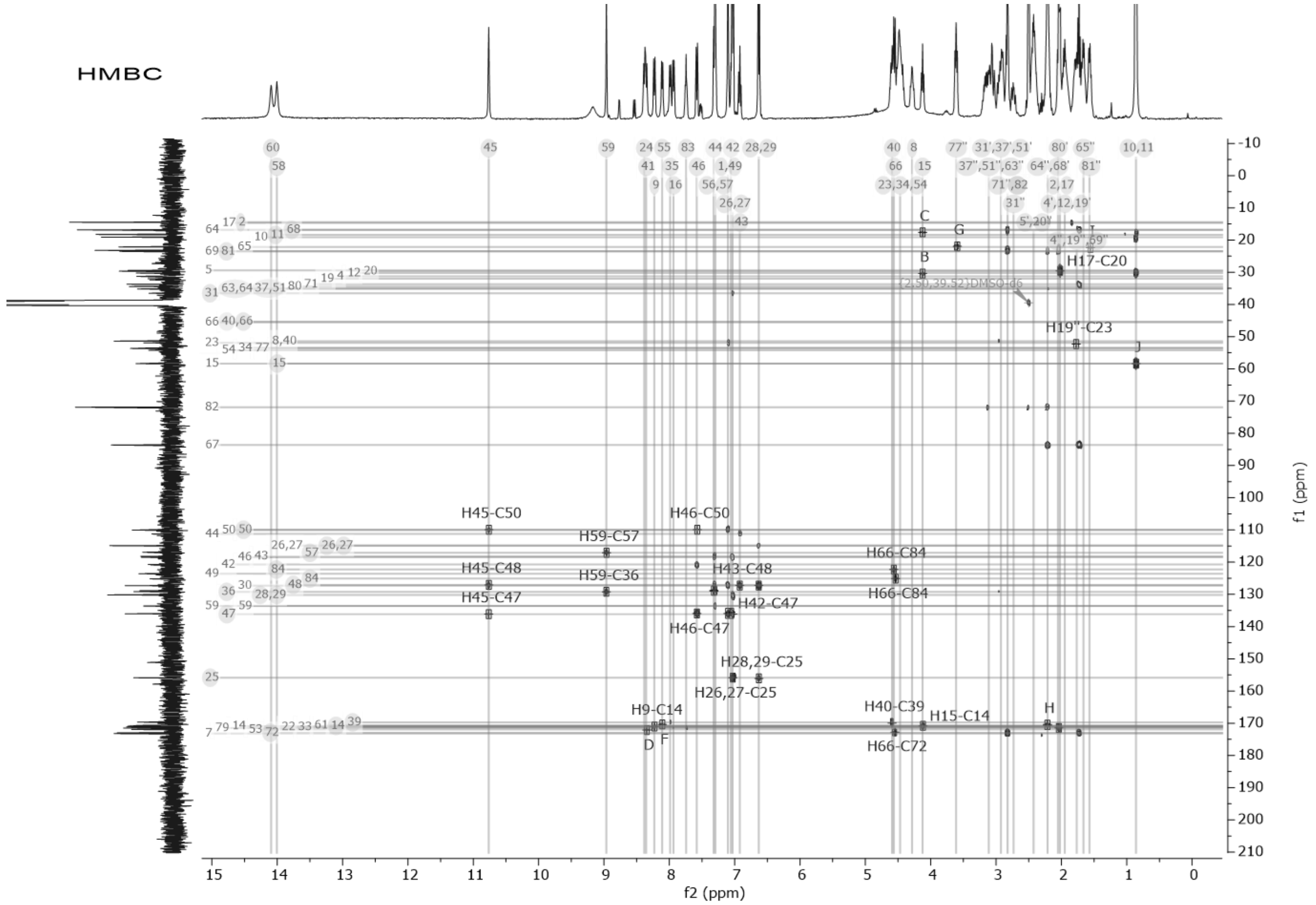

f2 (ppm)
f1 (ppm)
H45-C50
H46-C50
H59-C57
H45-C48
H59-C36
H43-C48
H45-C47
H42-C47
H46-C47
H66-C84
H66-C84
H28,29-C25
H26,27-C25
H9-C14
H40-C39
H15-C14
H66-C72
H17-C20
H19''-C23

HMBC shows a cross peak between H66 and C72, which indicates that the SuFA warhead is intact.

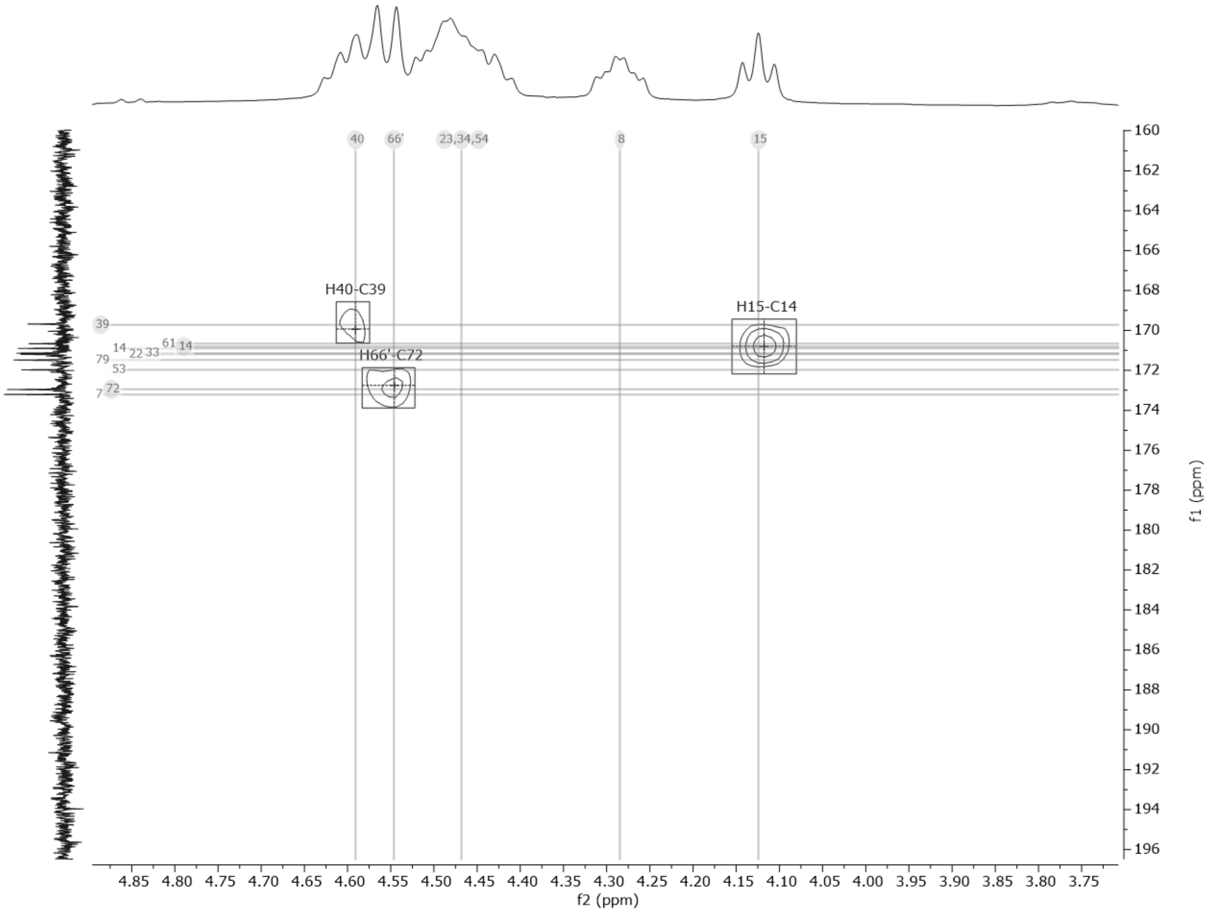

3.22.10 Compound **21** (PSMA SuFA probe)

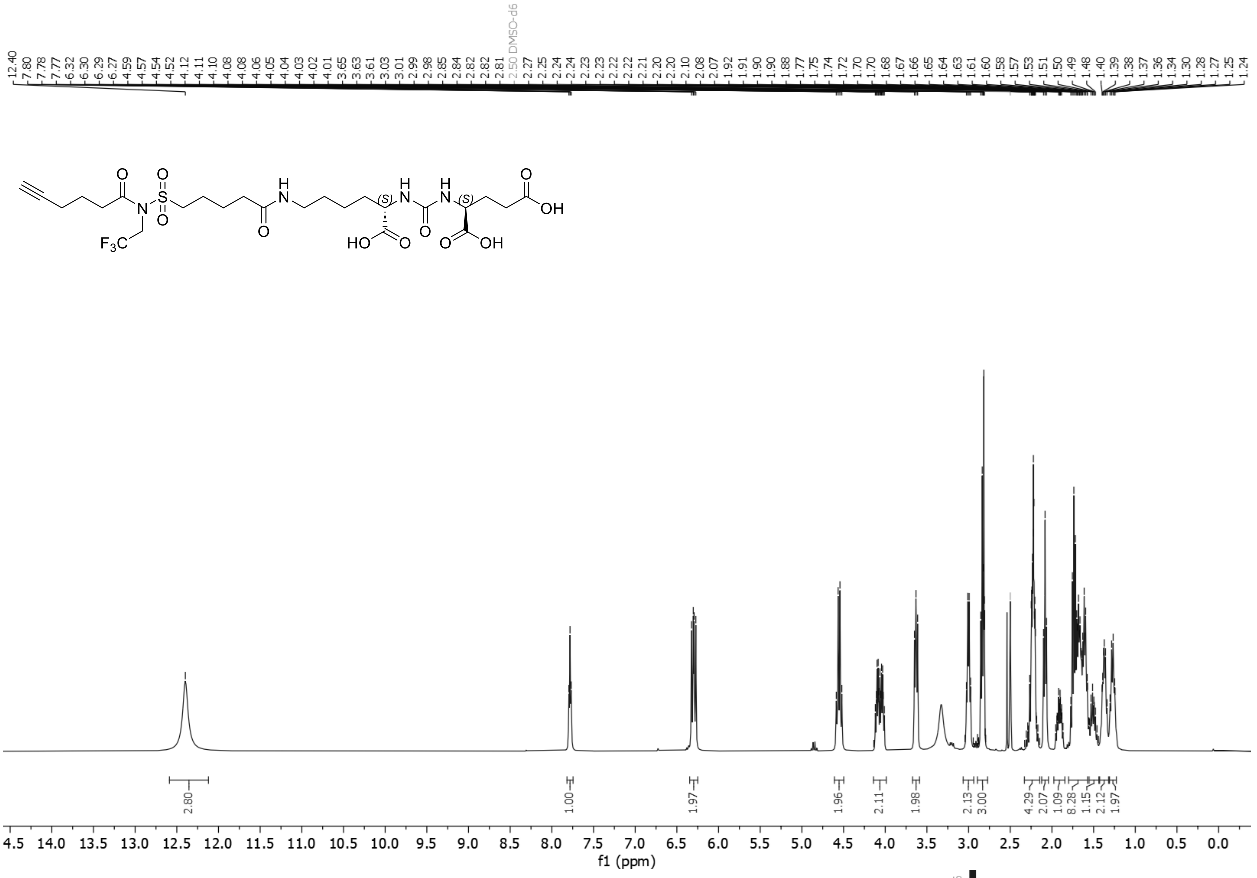

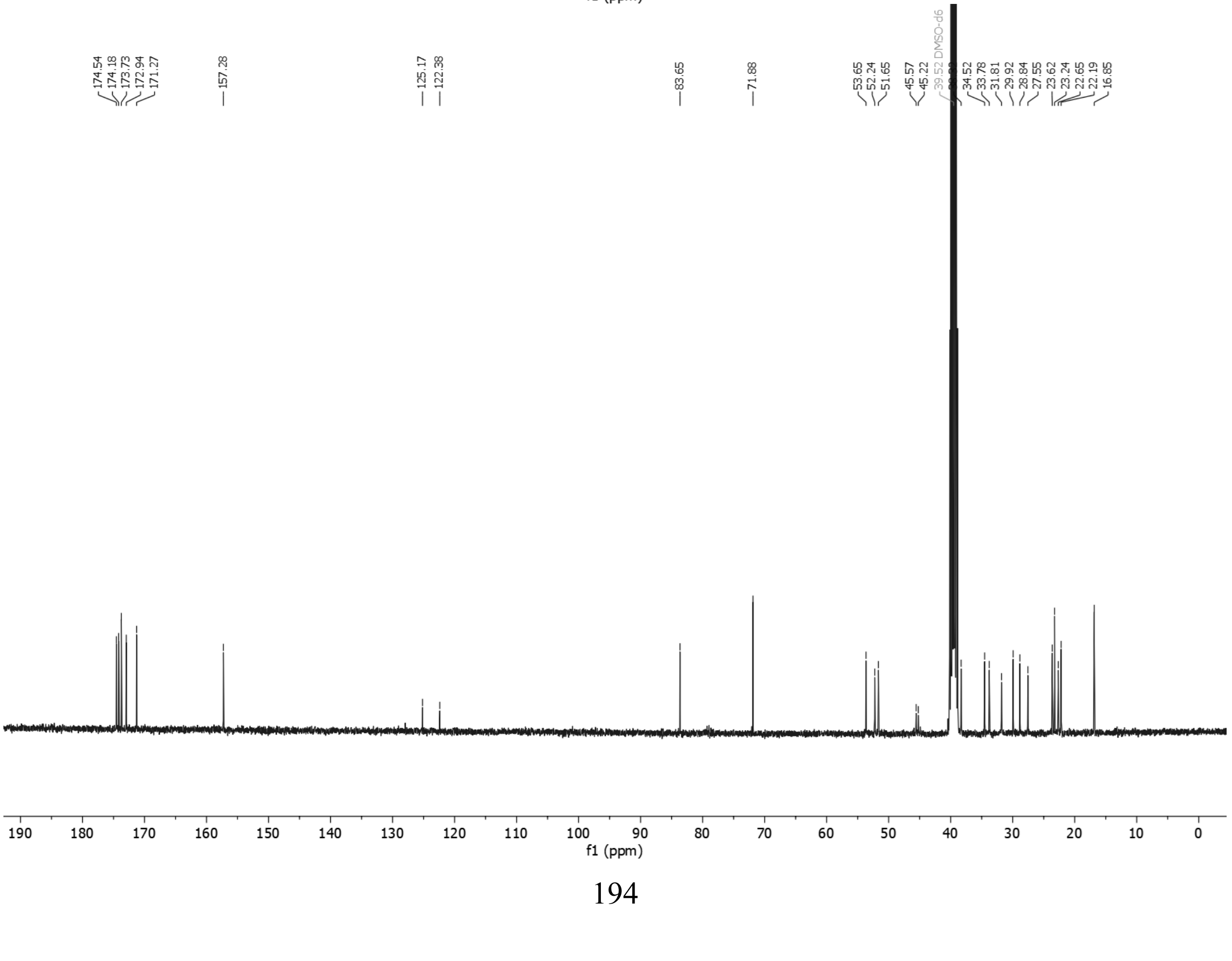

3.22.11 <u>Compound **23** (BTK SuFA probe)</u>

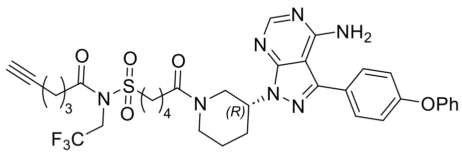

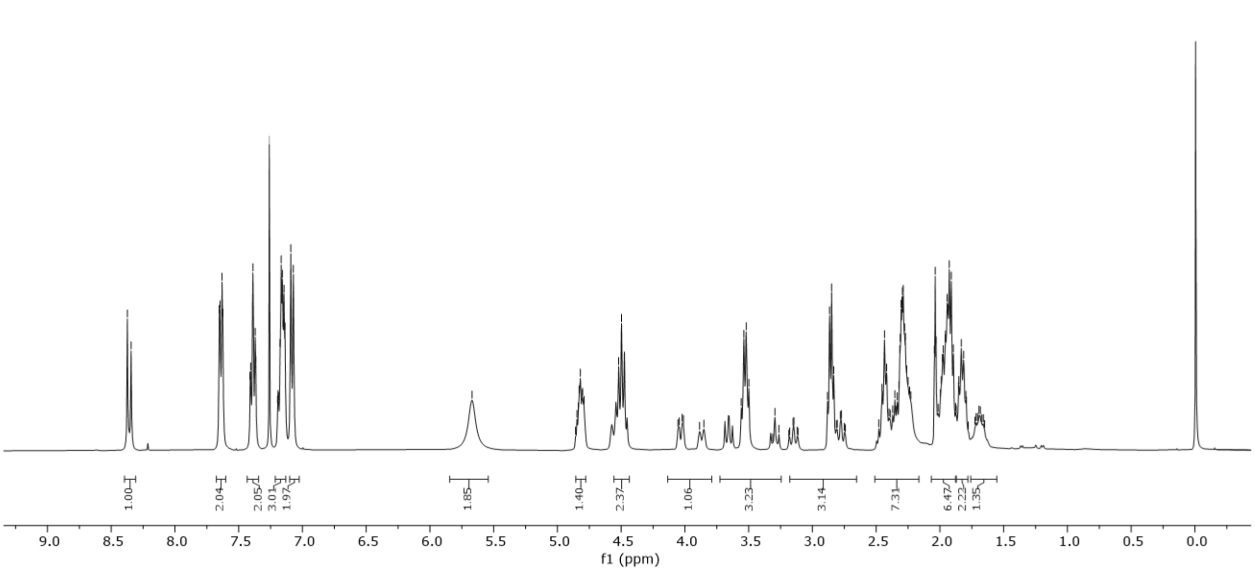

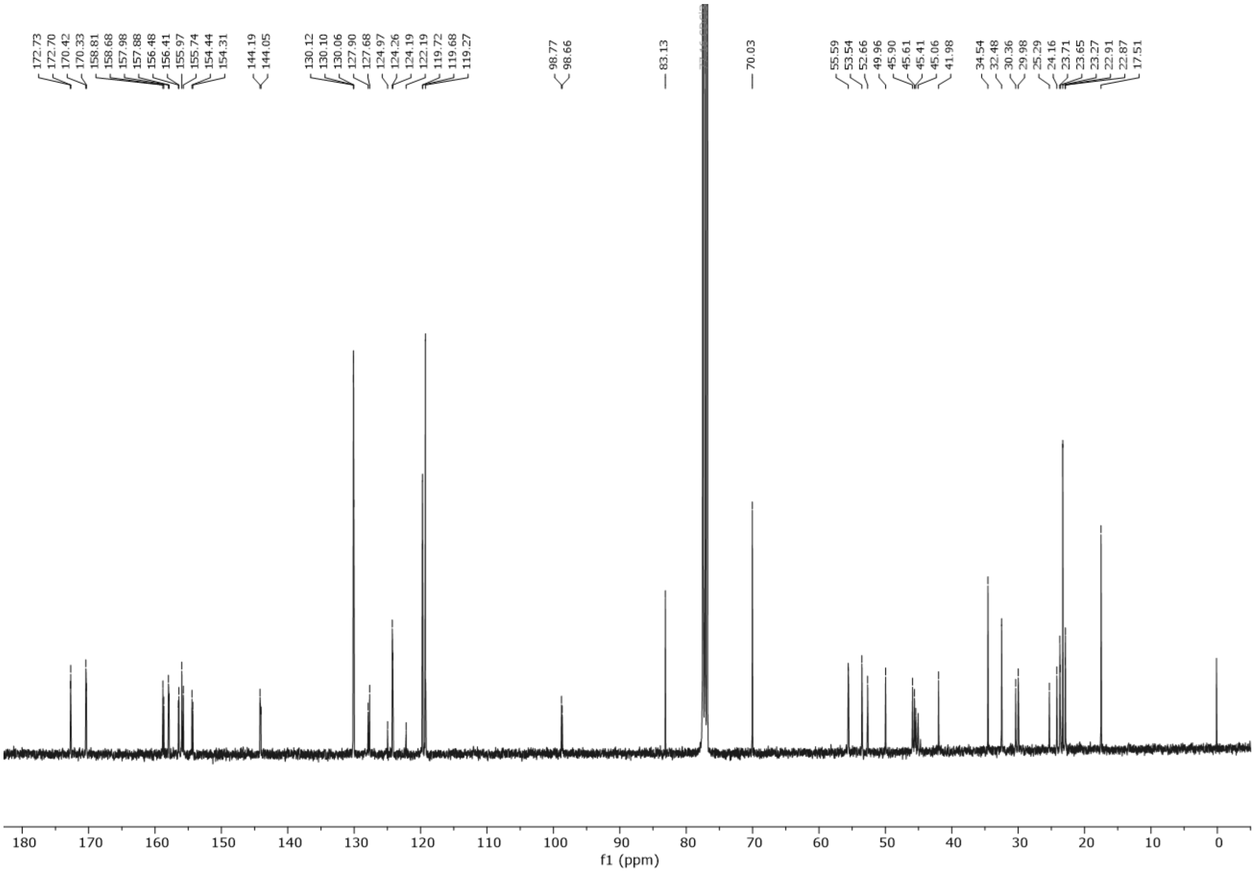

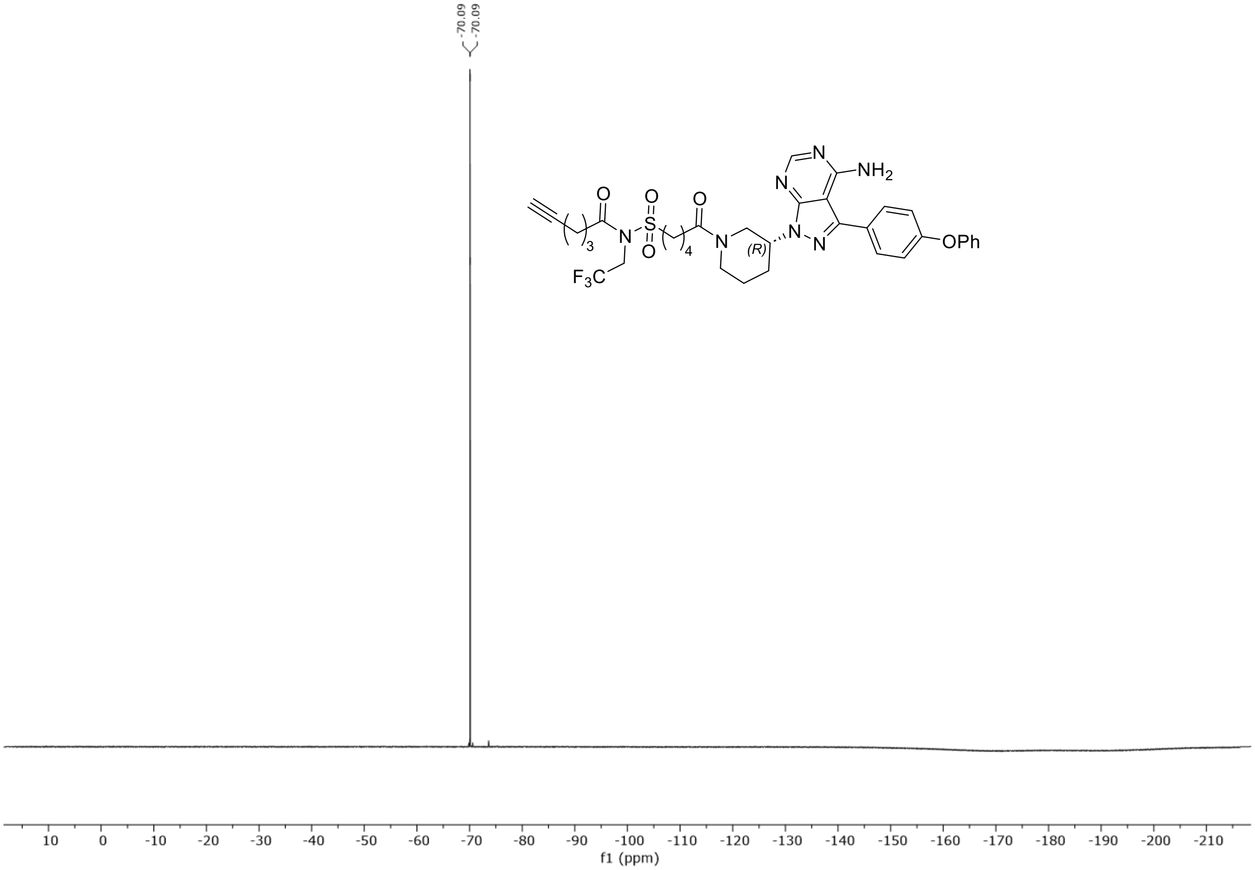
-70.09
-70.09
O
O
O
O
N
NH2
N
N
S
N
N
N
N
3
4
(R)
F3C
OPh
10 0 -10 -20 -30 -40 -50 -60 -70 -80 -90 -100 -110 -120 -130 -140 -150 -160 -170 -180 -190 -200 -210
f1 (ppm)

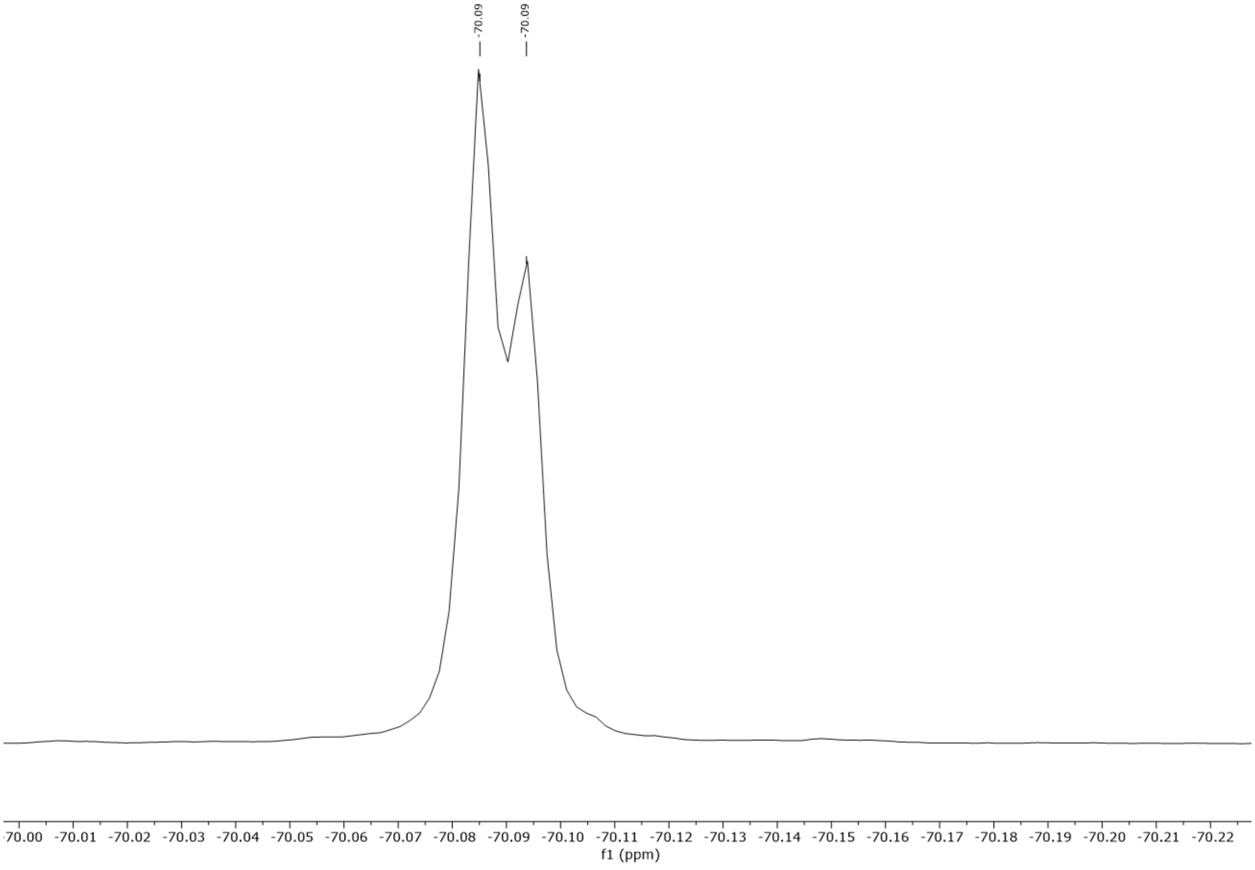
-70.09
-70.09
-70.00 -70.01 -70.02 -70.03 -70.04 -70.05 -70.06 -70.07 -70.08 -70.09 -70.10 -70.11 -70.12 -70.13 -70.14 -70.15 -70.16 -70.17 -70.18 -70.19 -70.20 -70.21 -70.22
f1 (ppm)

3.22.12 Compound **26** (VEGFR2 SuFA probe)

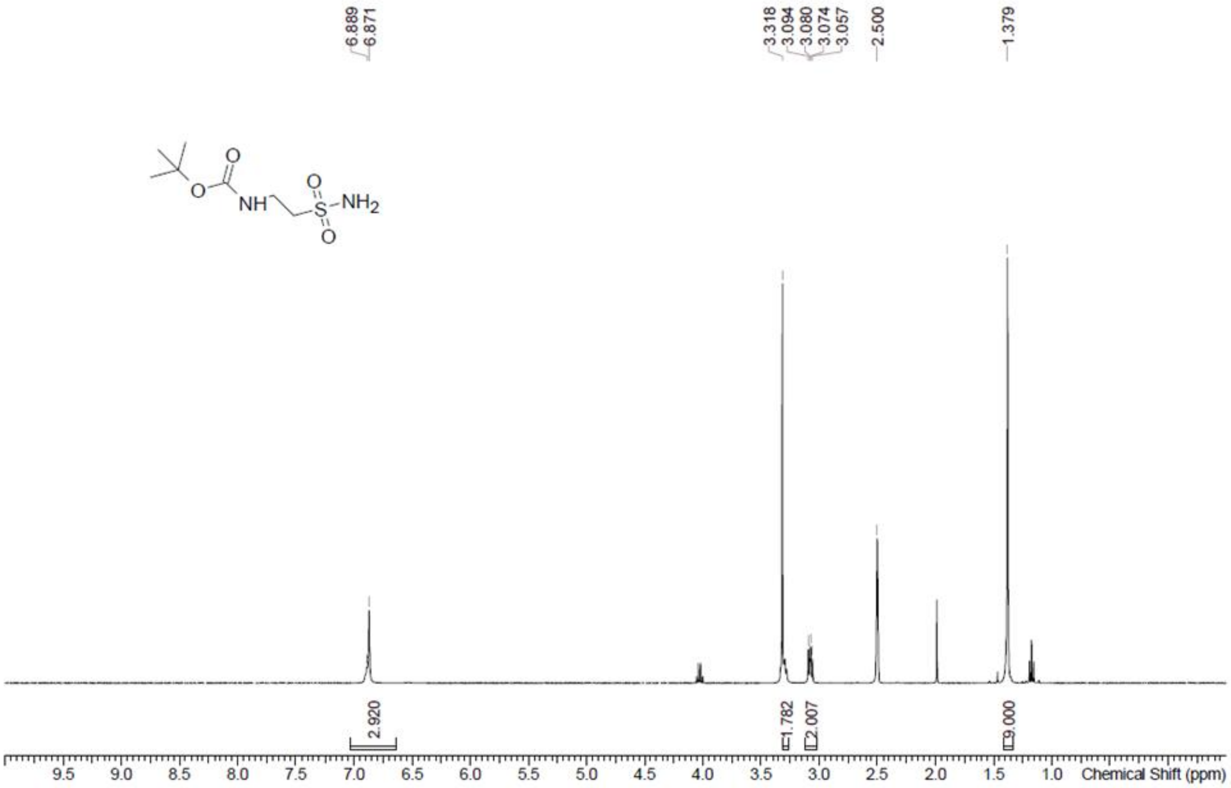

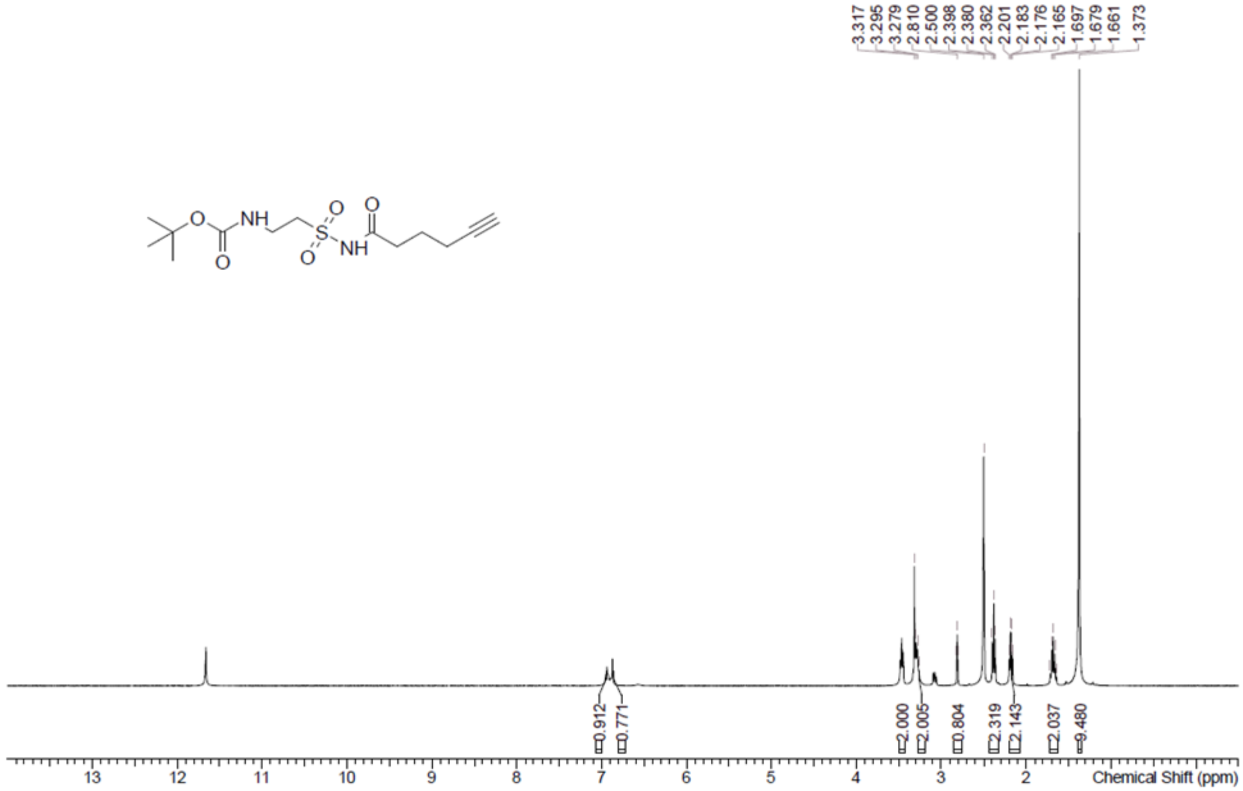

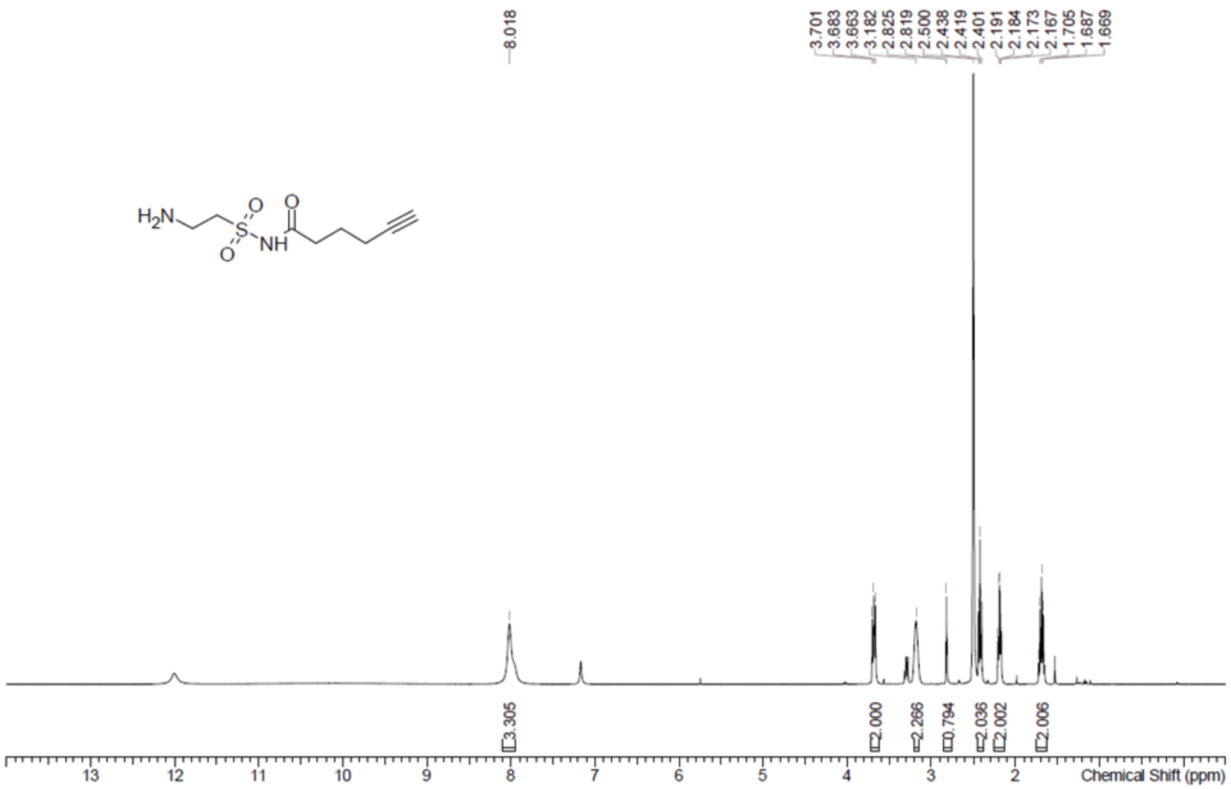

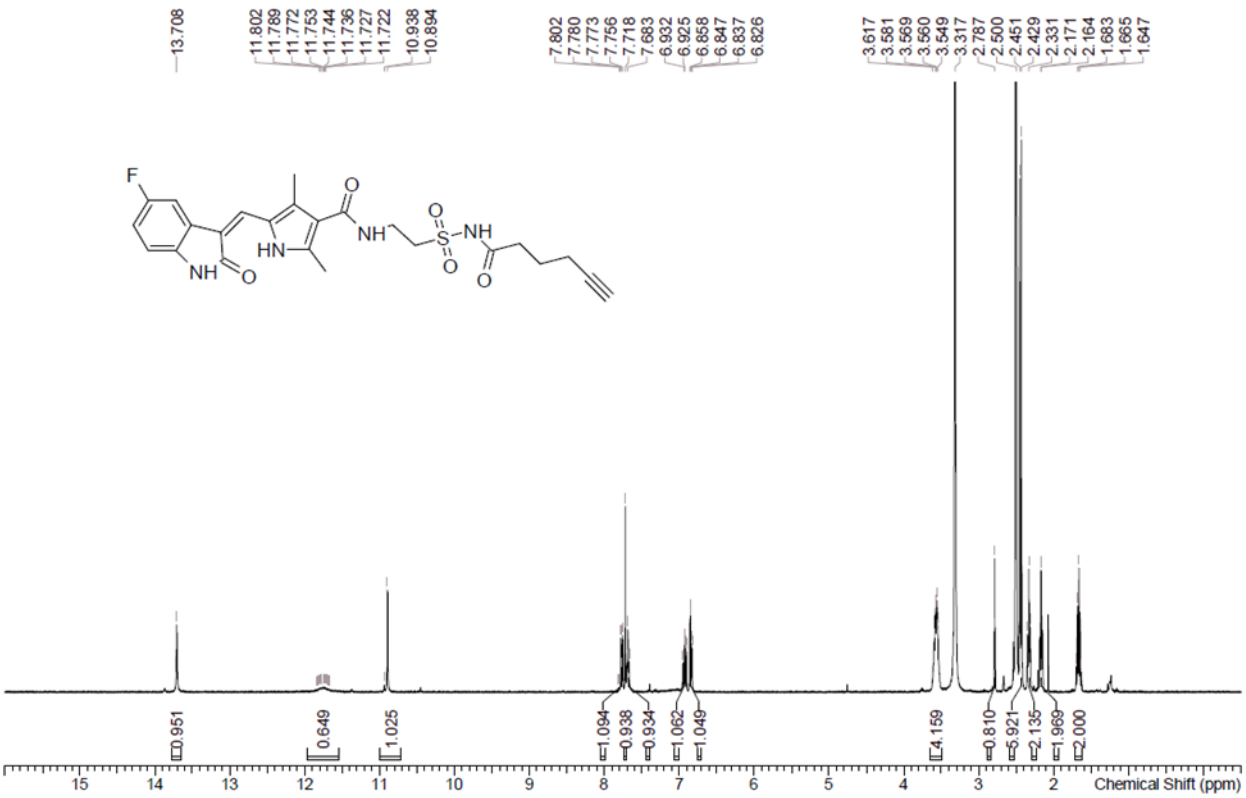

Compound 26

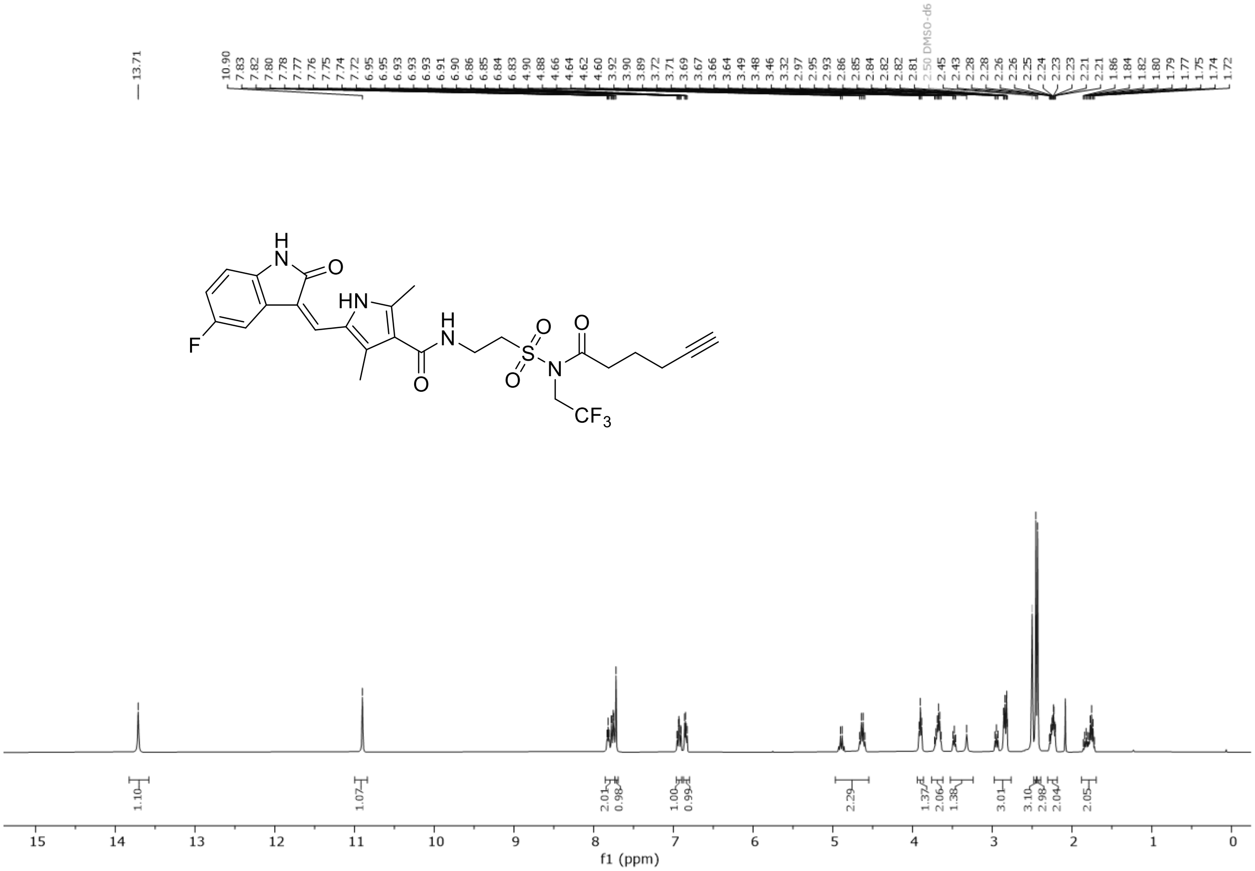
13.71
10.90 7.83 7.82 7.80 7.78 7.77 7.76 7.75 7.74 7.72 6.95 6.95 6.93 6.93 6.93 6.91 6.90 6.86 6.85 6.84 6.83 4.90 4.88 4.66 4.64 4.62 4.60 3.92 3.90 3.89 3.72 3.71 3.69 3.67 3.66 3.64 3.49 3.48 3.46 3.32 2.97 2.95 2.93 2.86 2.85 2.84 2.82 2.82 2.81 2.50 DMSO-d6 2.45 2.43 2.28 2.28 2.26 2.26 2.25 2.24 2.23 2.23 2.21 2.21 1.86 1.84 1.82 1.80 1.79 1.77 1.75 1.74 1.72
H
N
O
HN
H
N
O
O
S
O
N
F
O
CF3
1.10 1.07 2.01 0.98 1.00 0.99 2.29 1.37 2.06 1.38 3.01 3.10 2.98 2.04 2.05
15 14 13 12 11 10 9 8 7 6 5 4 3 2 1 0
f1 (ppm)

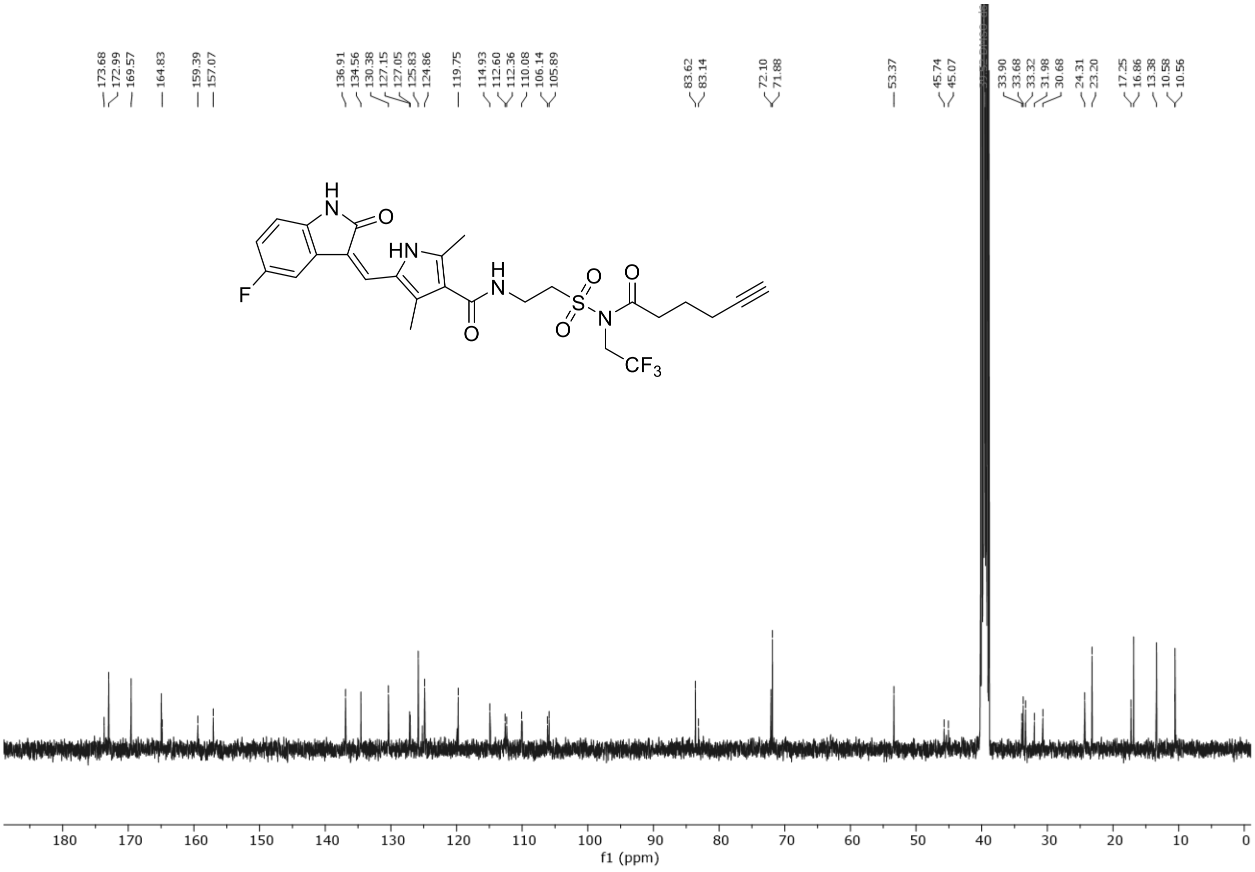
173.68 172.99 169.57 164.83 159.39 157.07 136.91 134.56 130.38 127.15 127.05 125.83 124.86 119.75 114.93 112.60 112.36 110.08 106.14 105.89 83.62 83.14 72.10 71.88 53.37 45.74 45.07 33.90 33.68 33.32 31.98 30.68 24.31 23.20 17.25 16.86 13.38 10.58 10.56
H
N
O
HN
H
N
O
O
S
O
N
F
O
CF3
180 170 160 150 140 130 120 110 100 90 80 70 60 50 40 30 20 10 0
f1 (ppm)

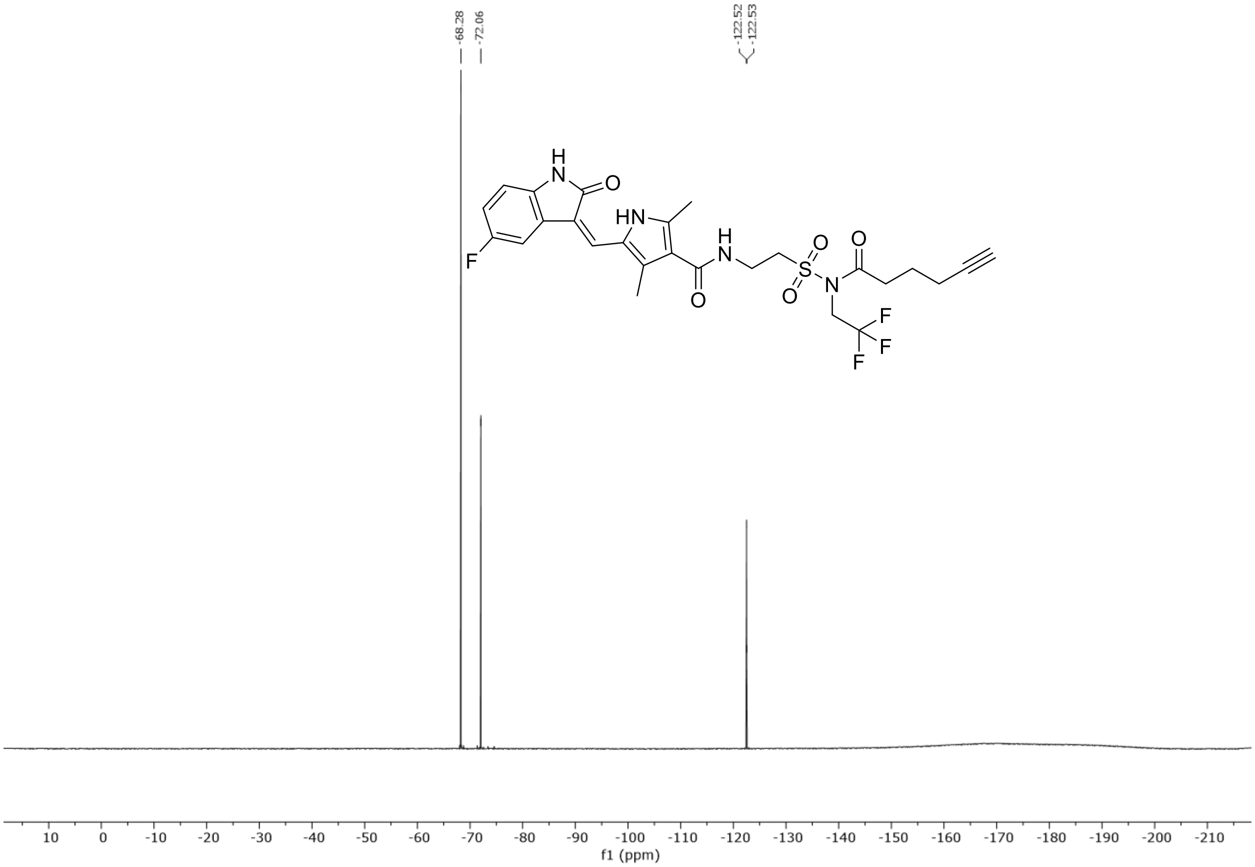
-68.28
-72.06
-122.52
-122.53
H
N
O
HN
H
N
O
S
O
O
N
O
F
F
F
F
10 0 -10 -20 -30 -40 -50 -60 -70 -80 -90 -100 -110 -120 -130 -140 -150 -160 -170 -180 -190 -200 -210
f1 (ppm)

3.22.13 Compound **27** (Halo-PSMA-GRC)

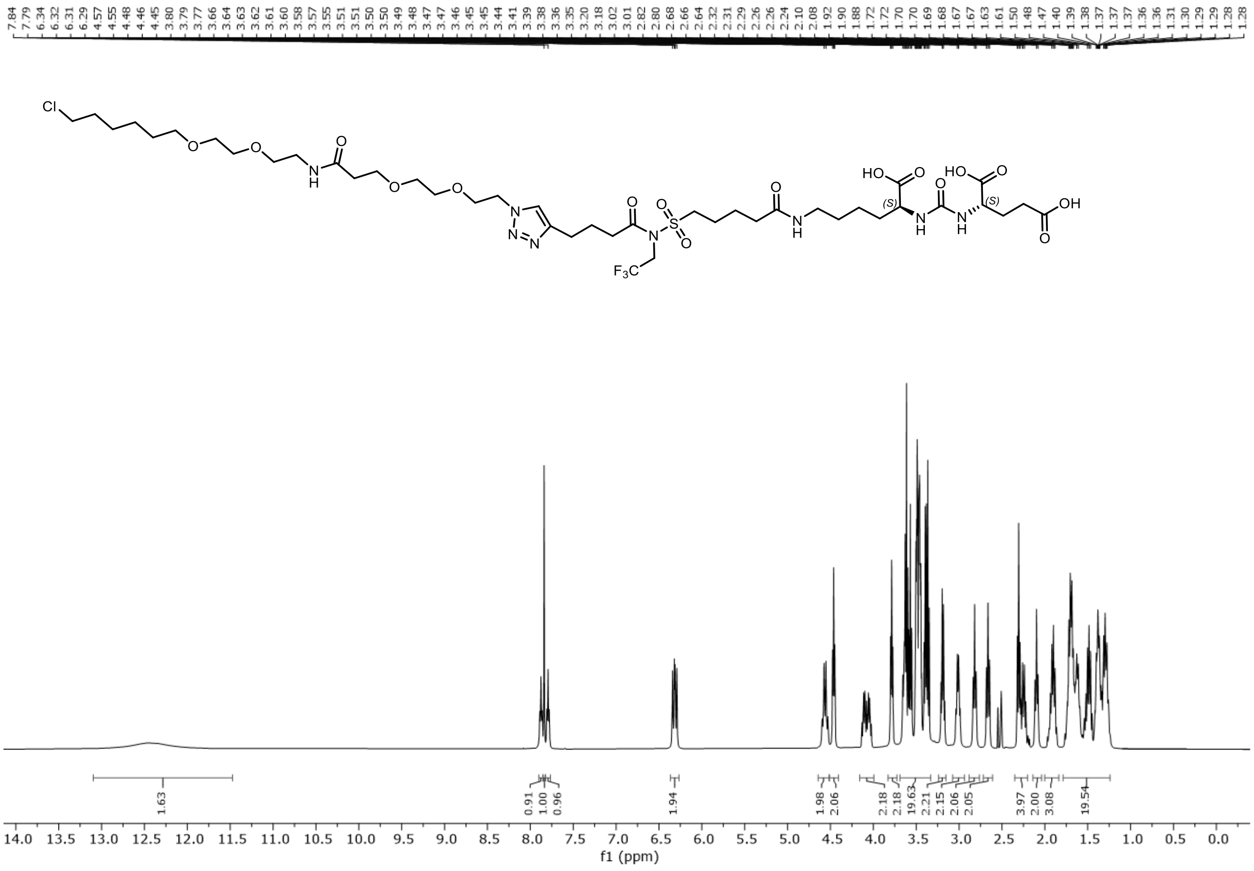

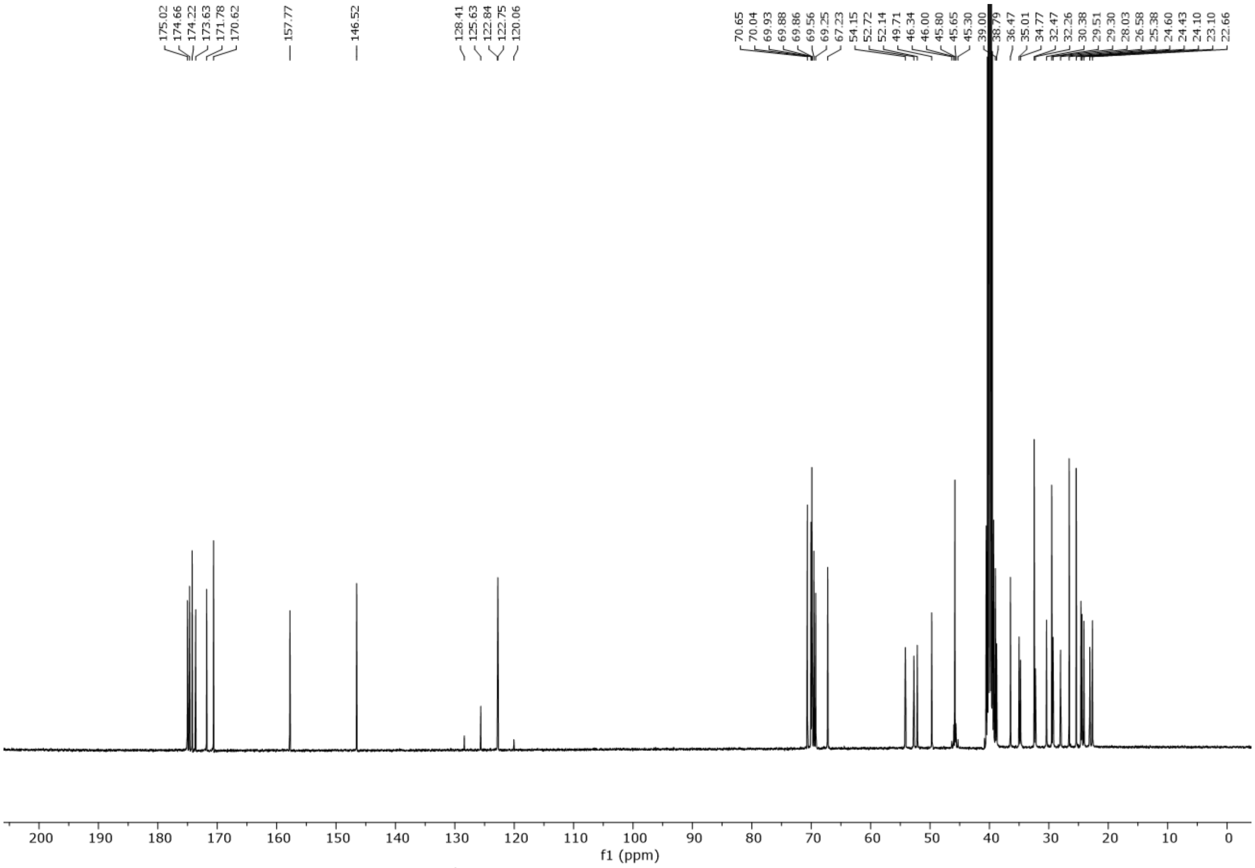
175.02
174.66
174.22
173.63
171.78
170.62
157.77
146.52
128.41
125.63
122.84
122.75
120.06
70.65
70.04
69.93
69.88
69.86
69.56
69.25
67.23
54.15
52.72
52.14
49.71
46.34
46.00
45.80
45.65
45.30
39.00
38.79
36.47
35.01
34.77
32.47
32.26
30.38
29.51
29.30
28.03
26.58
25.38
24.60
24.43
24.10
23.10
22.66
f1 (ppm)

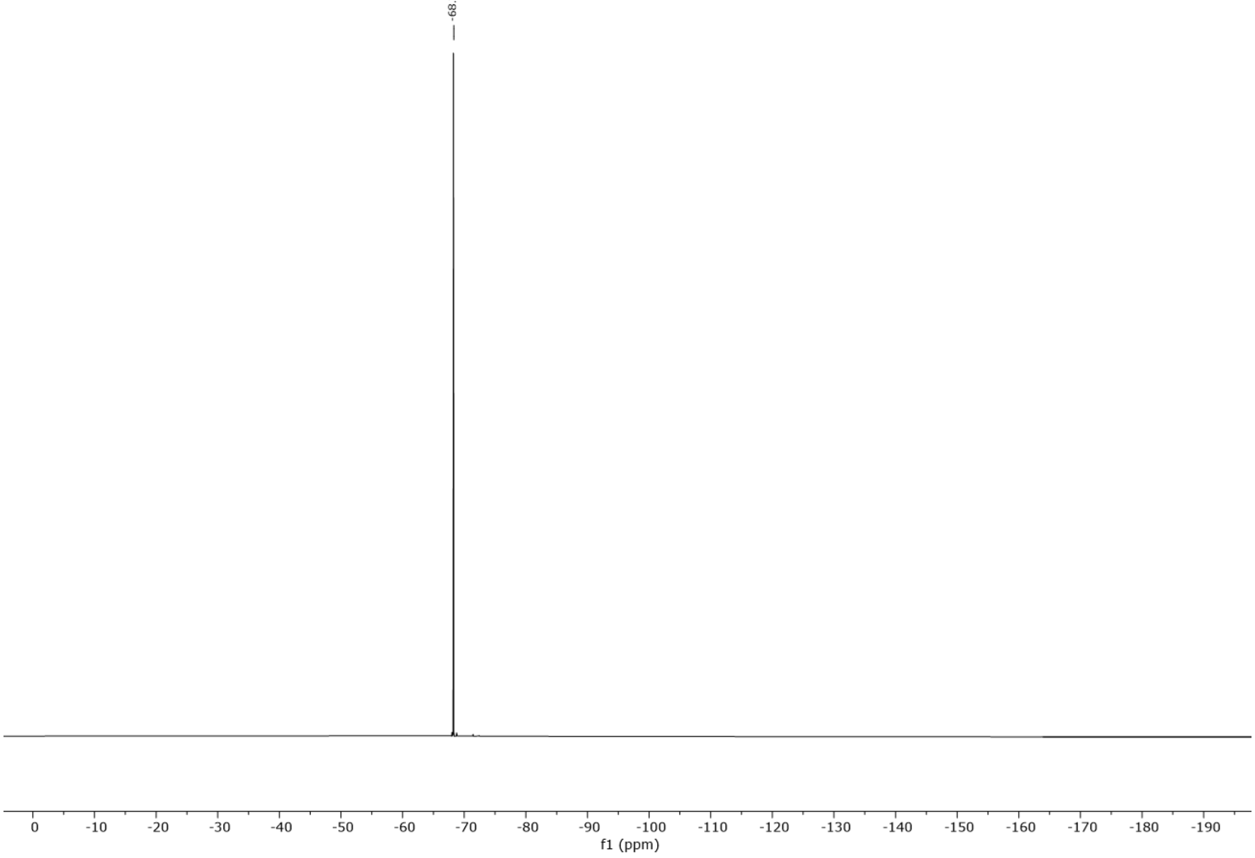
-68.31
f1 (ppm)

3.22.14 Compound **28** (Halo-PSMA-iGRC)

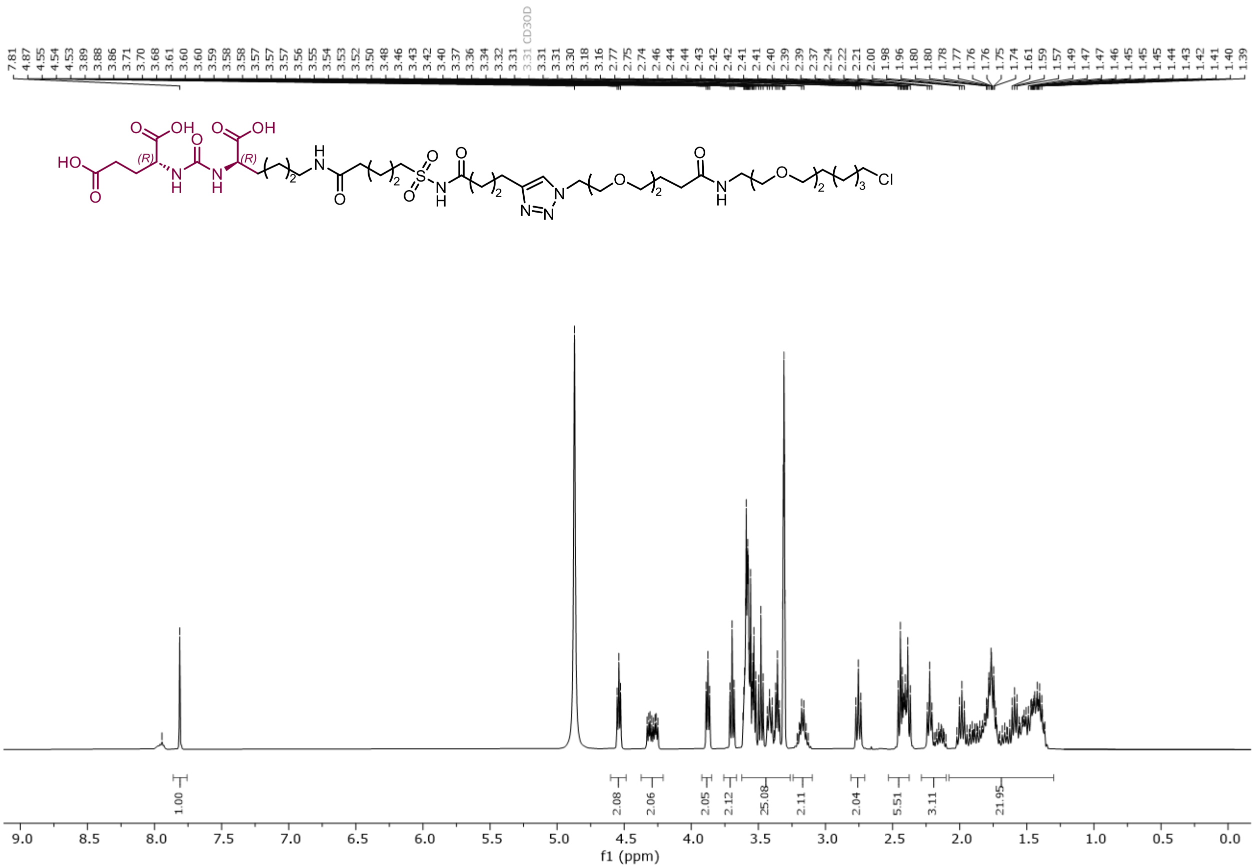

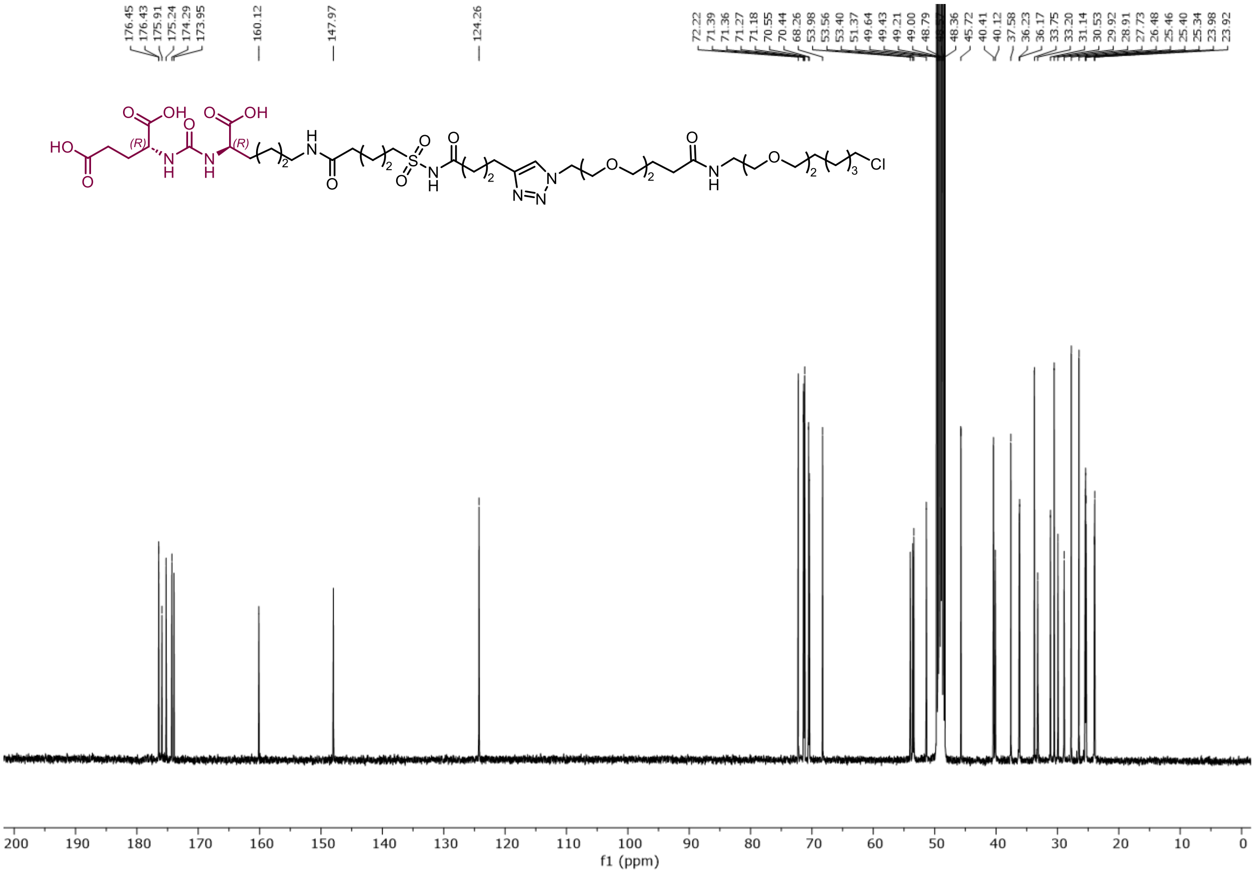

3.22.15 Compound **29** (FKBP-PSMA-GRC)

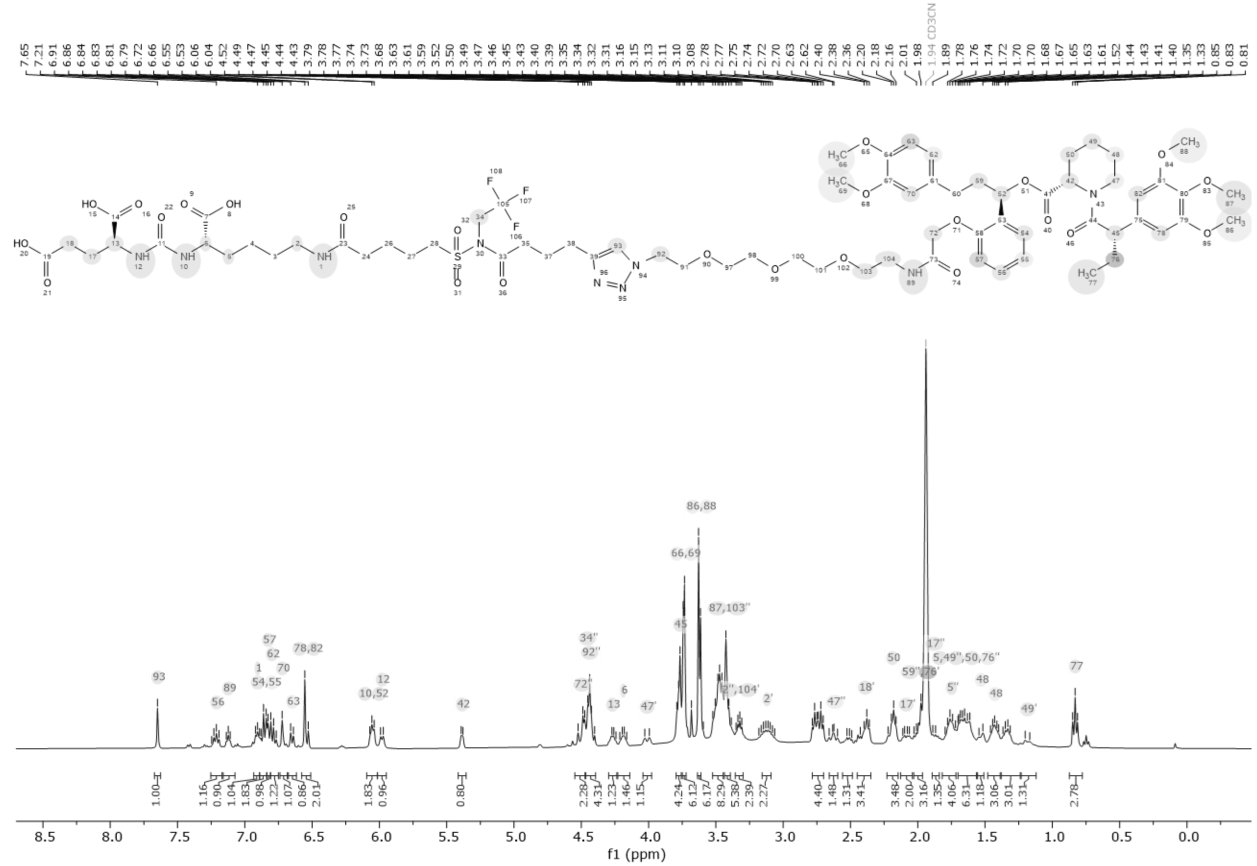

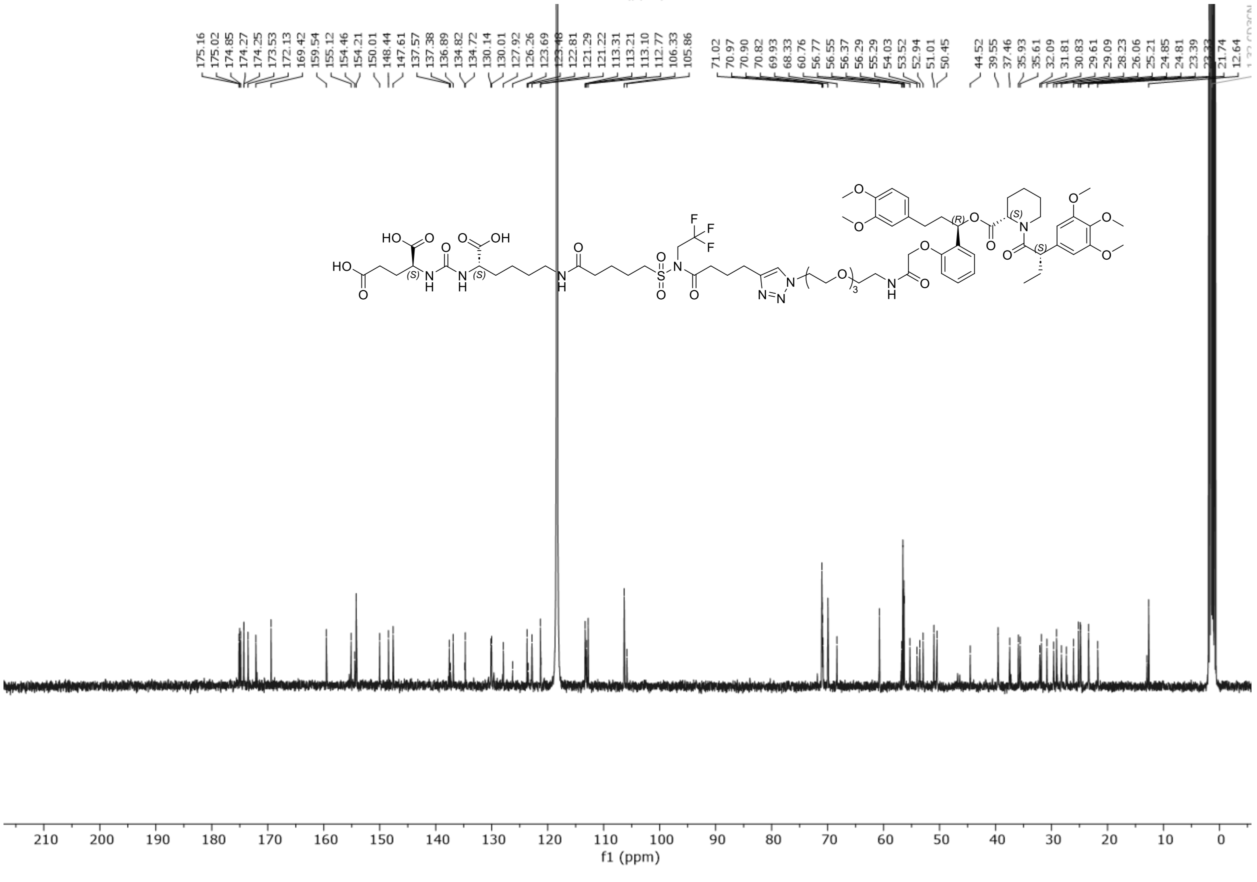

19F NMR

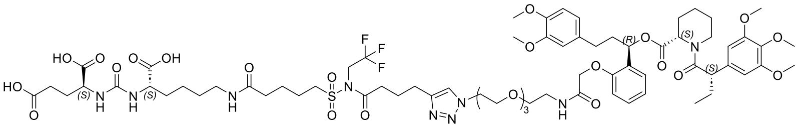

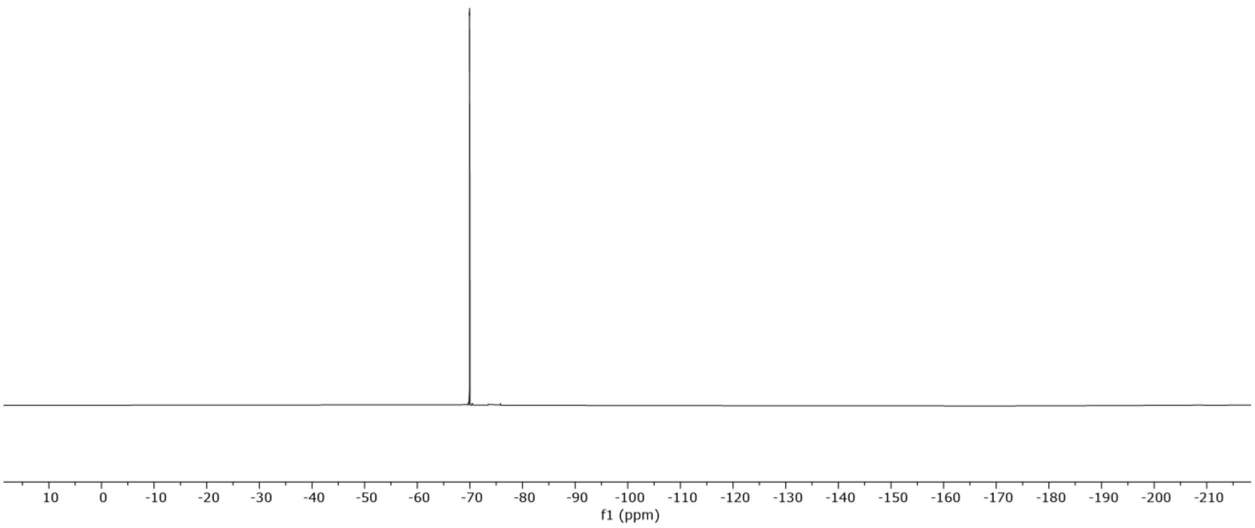

-69.94
10
0
-10
-20
-30
-40
-50
-60
-70
-80
-90
-100
-110
-120
-130
-140
-150
-160
-170
-180
-190
-200
-210
f1 (ppm)

COSY

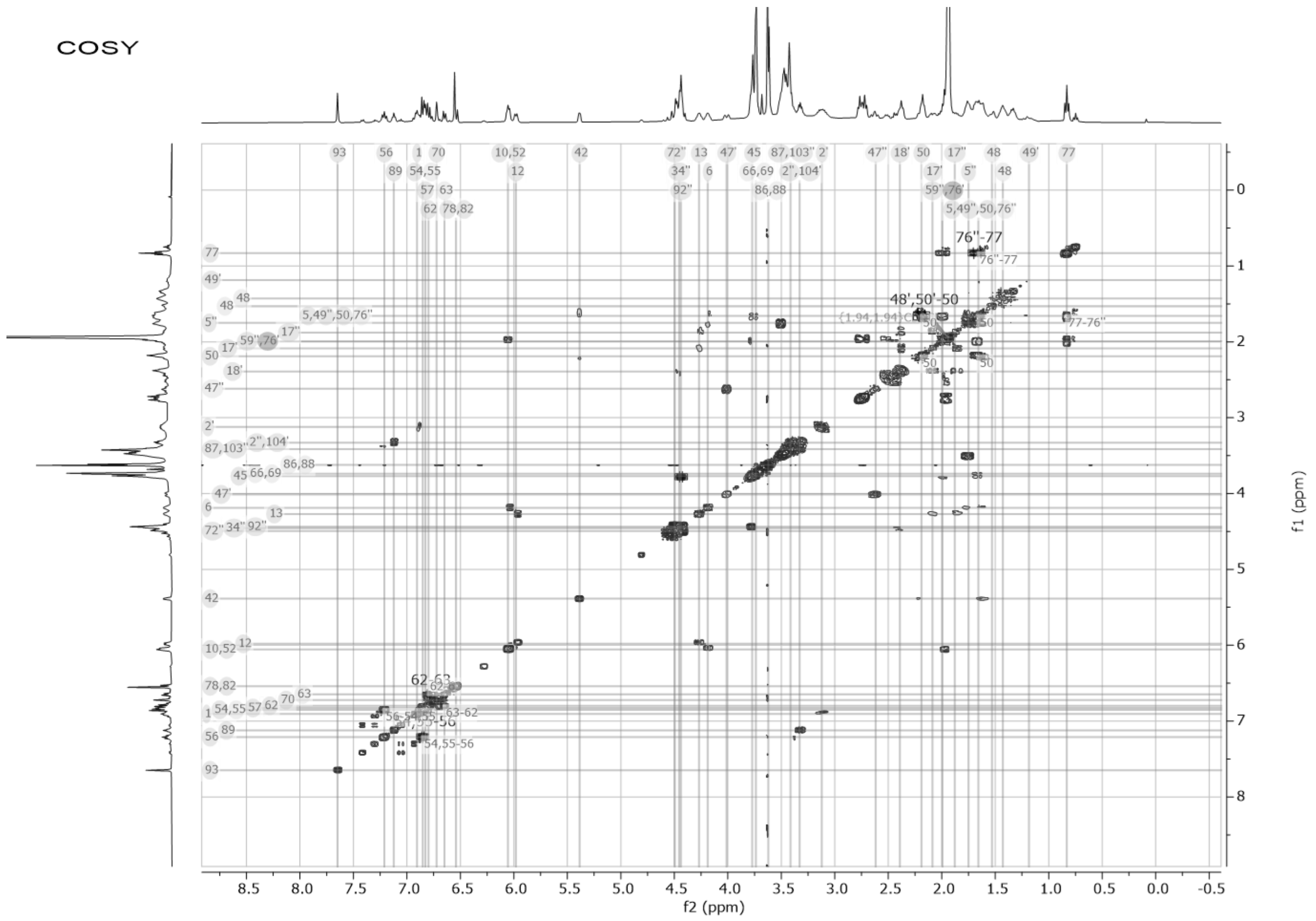

76''-77
48',50'-50
62-63
f2 (ppm)
f1 (ppm)
8.5 8.0 7.5 7.0 6.5 6.0 5.5 5.0 4.5 4.0 3.5 3.0 2.5 2.0 1.5 1.0 0.5 0.0 -0.5
0 1 2 3 4 5 6 7 8

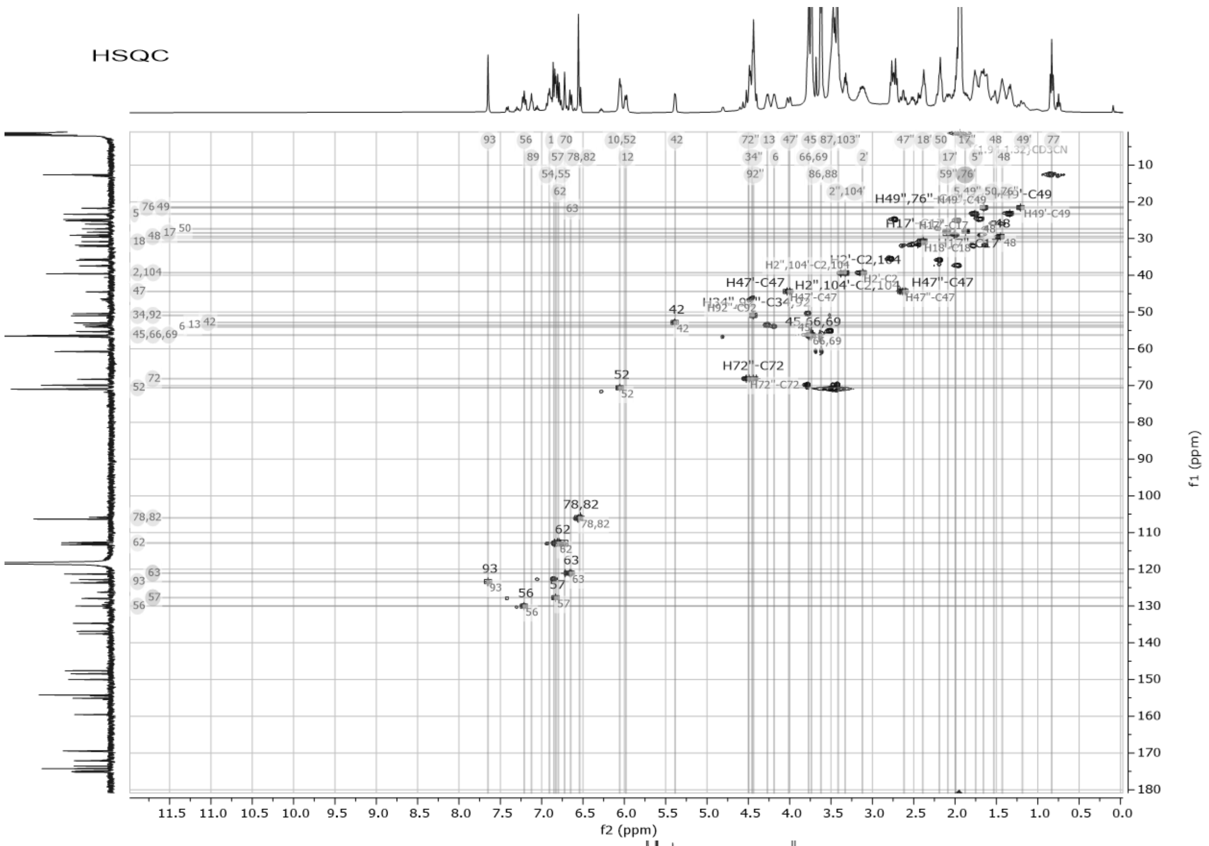
HSQC
H49",76"-C49
H17'-C17
H2'-C2,104
H47'-C47
H2",104'-C2,104
H47"-C47
H34",92"-C34,92
45,66,69
H72"-C72
52
42
78,82
62
63
93
57
56
f2 (ppm)
f1 (ppm)

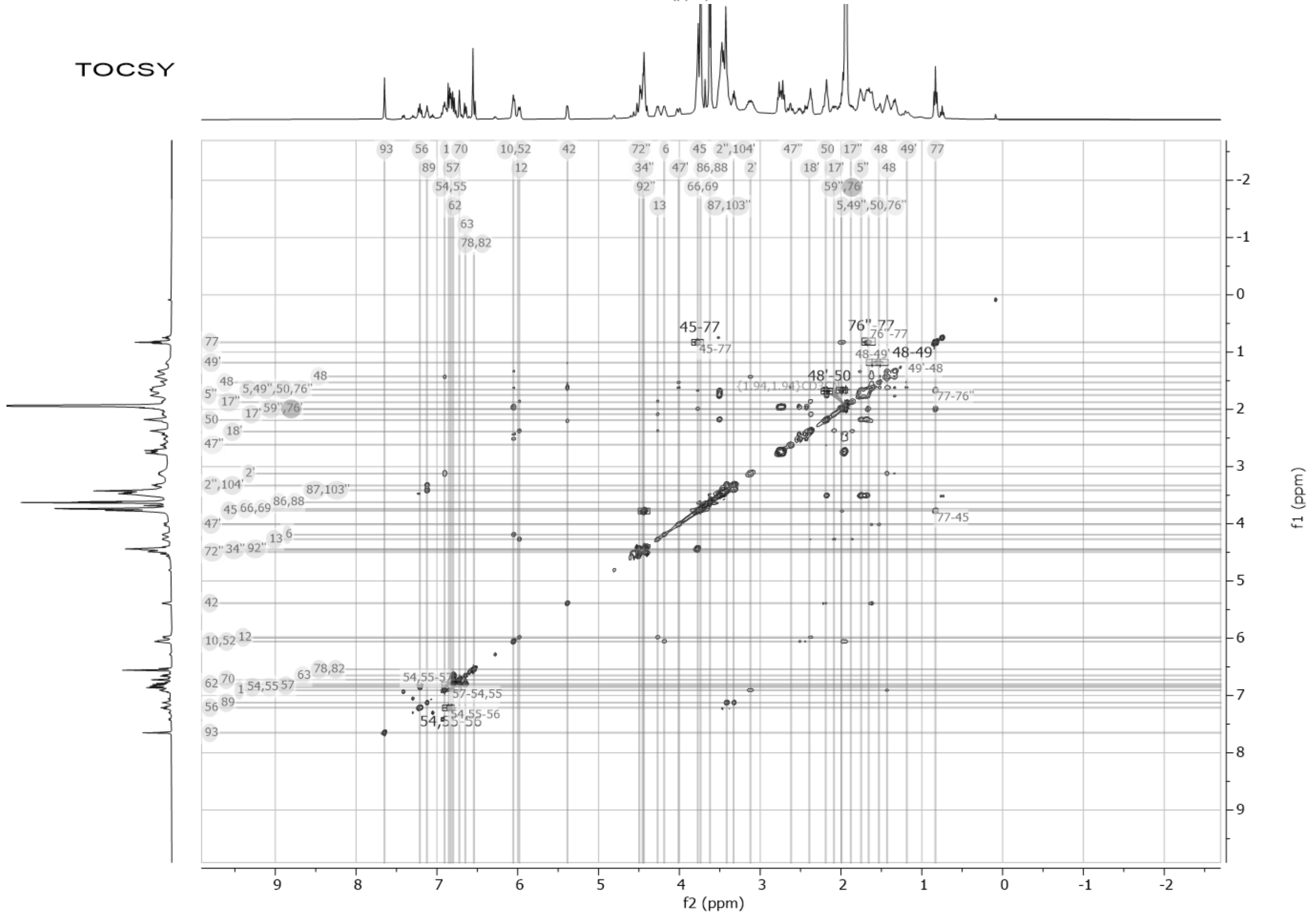
TOCSY
45-77
76"-77
48-49
48'-50
49'-48
77-76"
77-45
54,55-57
57-54,55
54,55-56
f2 (ppm)
f1 (ppm)

NOESY

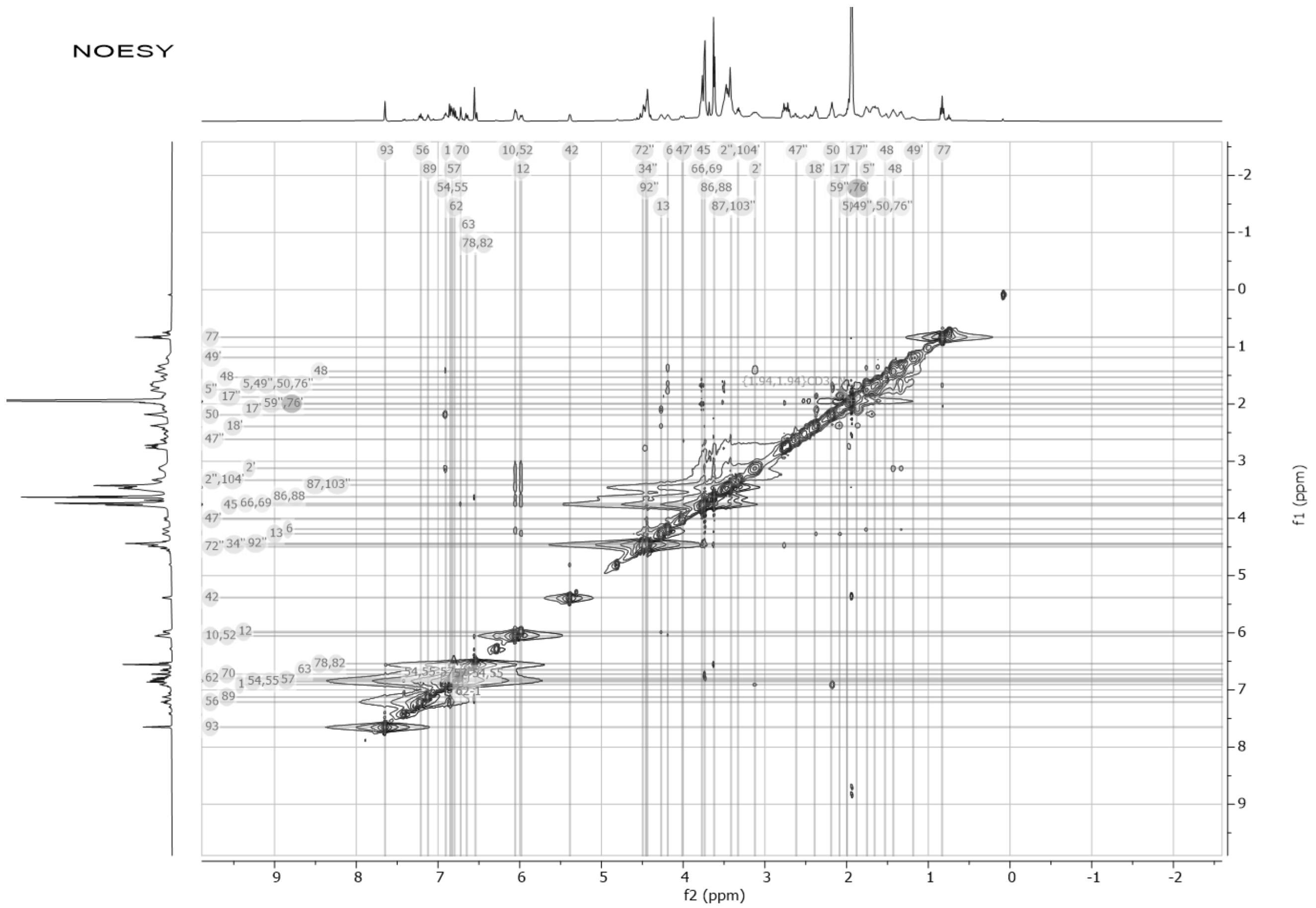

HMBC

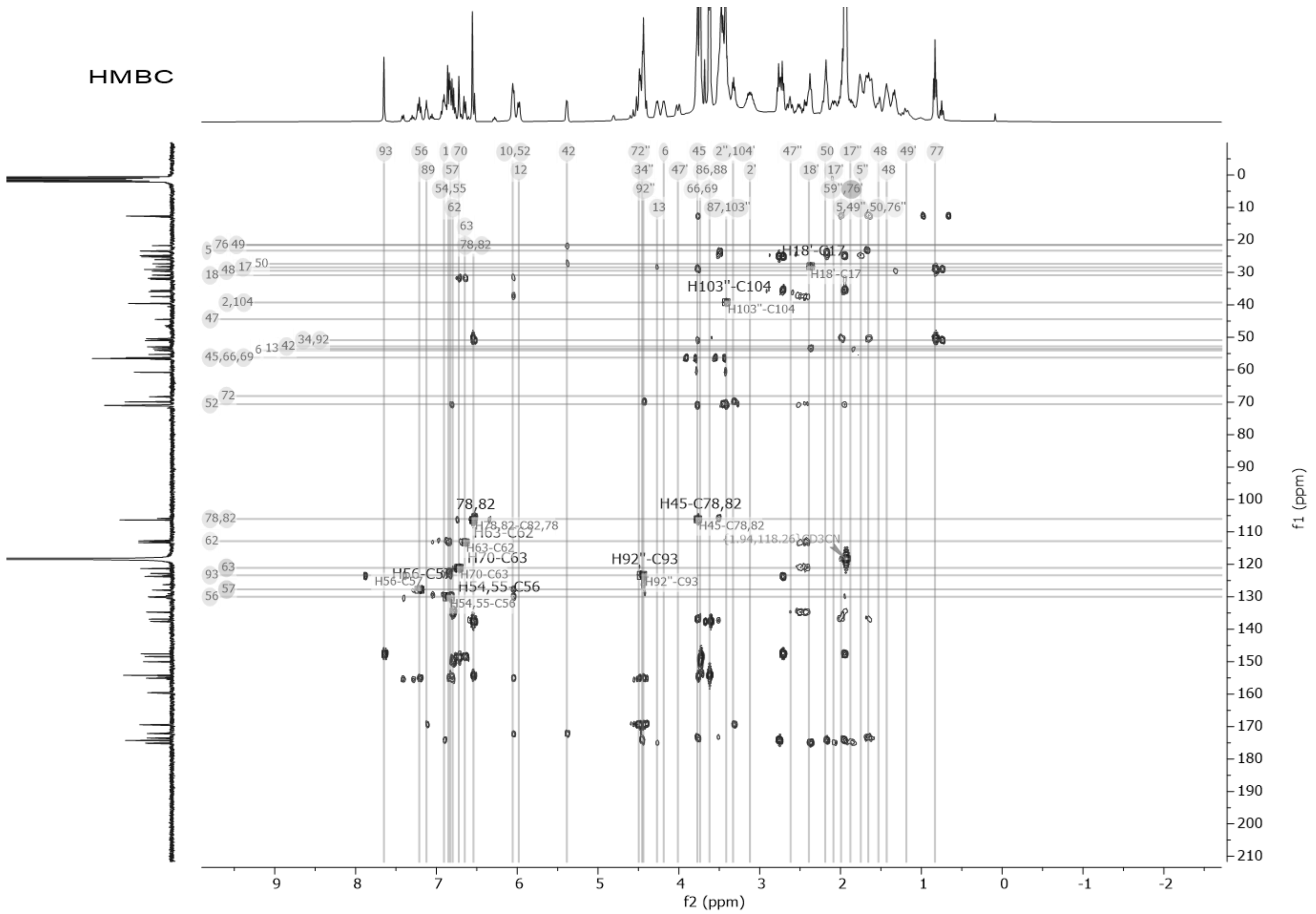

f2 (ppm)
f1 (ppm)
H18'-C17
H103''-C104
H45-C78,82
78,82
H63-C62
H70-C63
H92''-C93
H56-C57
H54,55-C56

3.22.16 Compound **30** (FKBP-PSMA-iGRC)

Intermediate **30c**

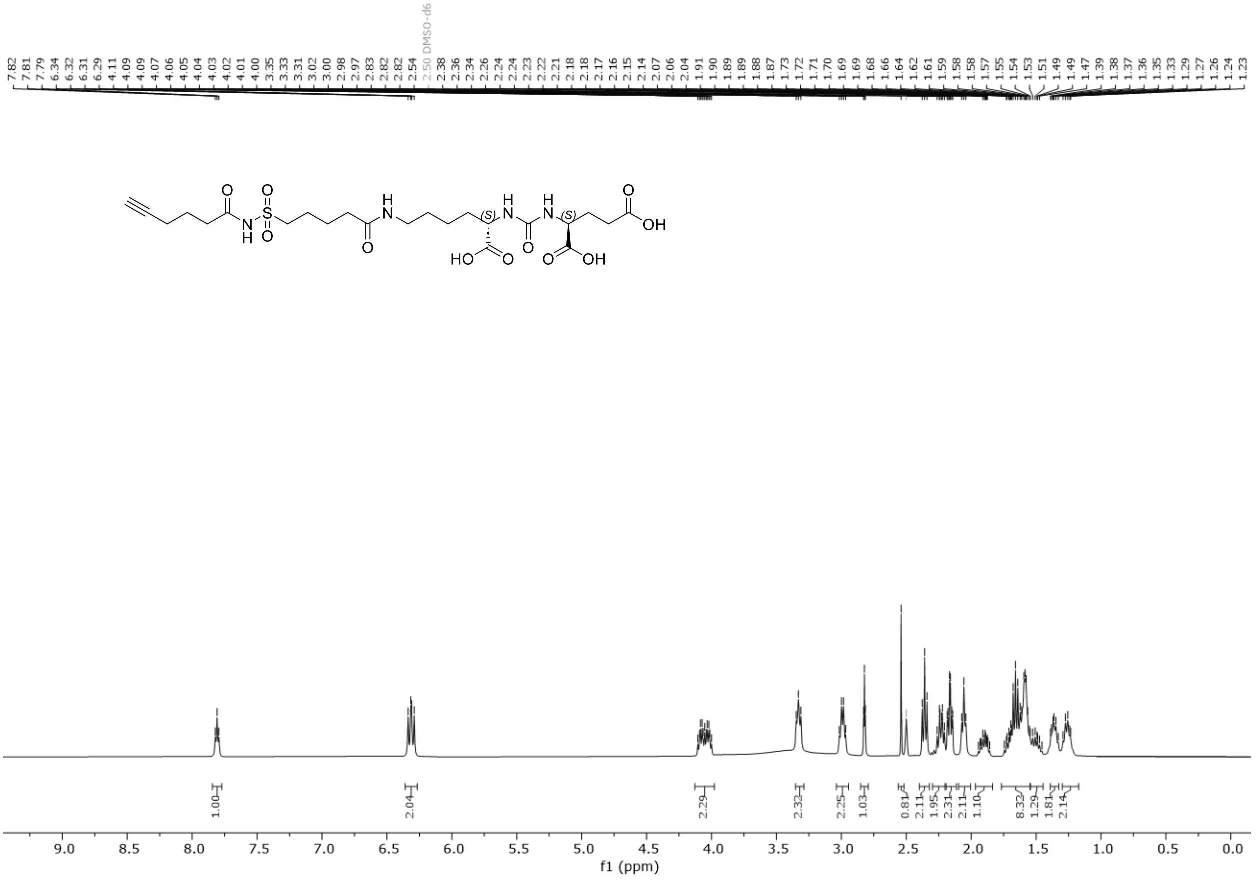

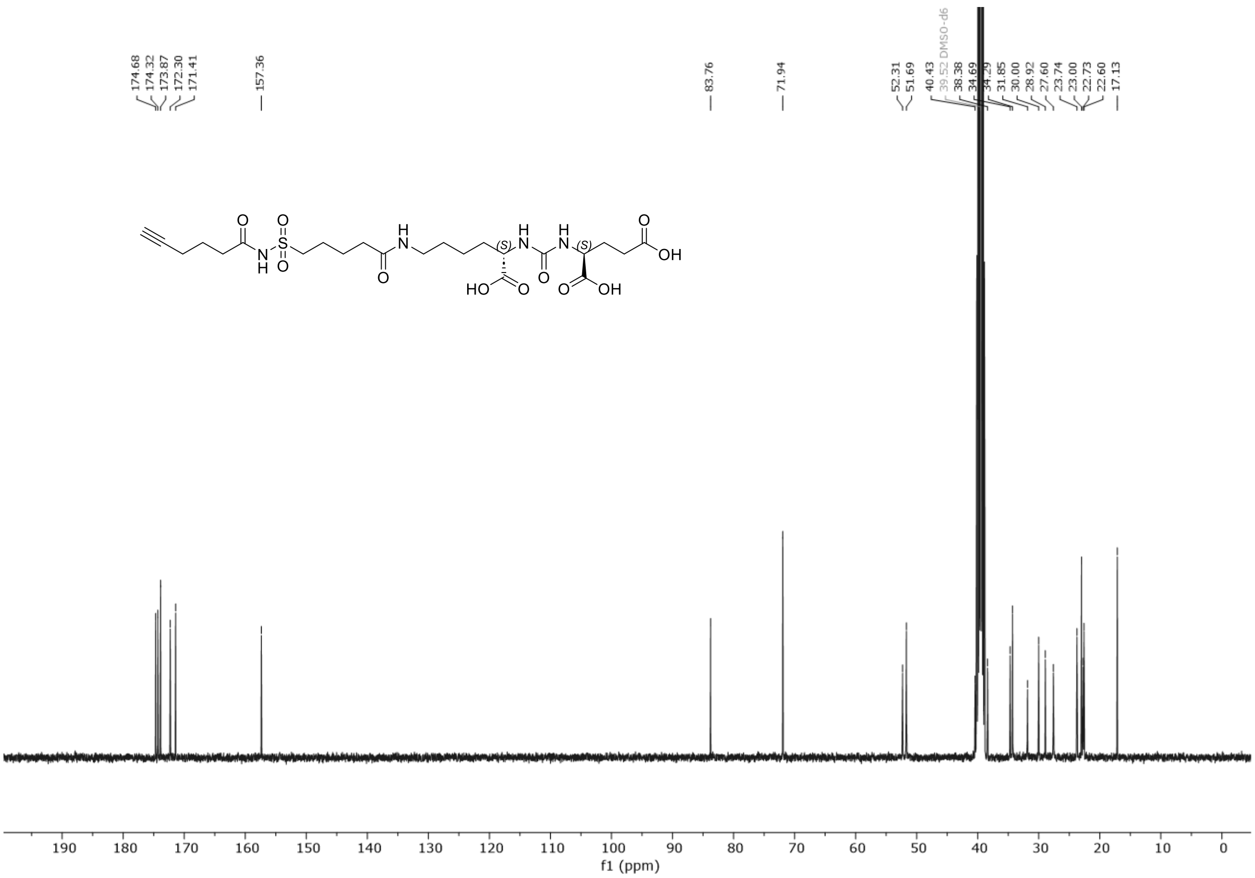
174.68
174.32
173.87
172.30
171.41
157.36
83.76
71.94
52.31
51.69
40.43
39.52 DMSO-d6
38.38
34.69
34.29
31.85
30.00
28.92
27.60
23.74
23.00
22.73
22.60
17.13
f1 (ppm)

Compound **30** (PSMA-iGRC-FKBP)

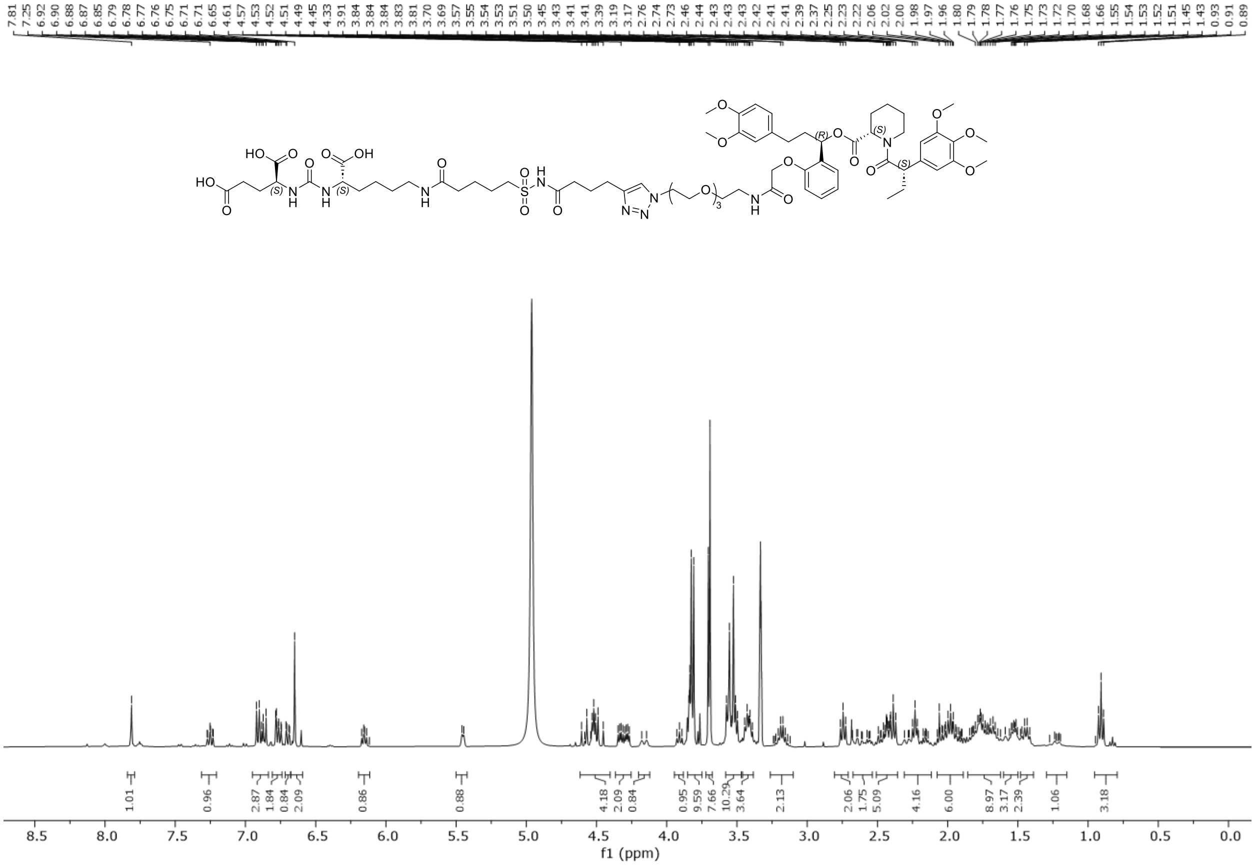

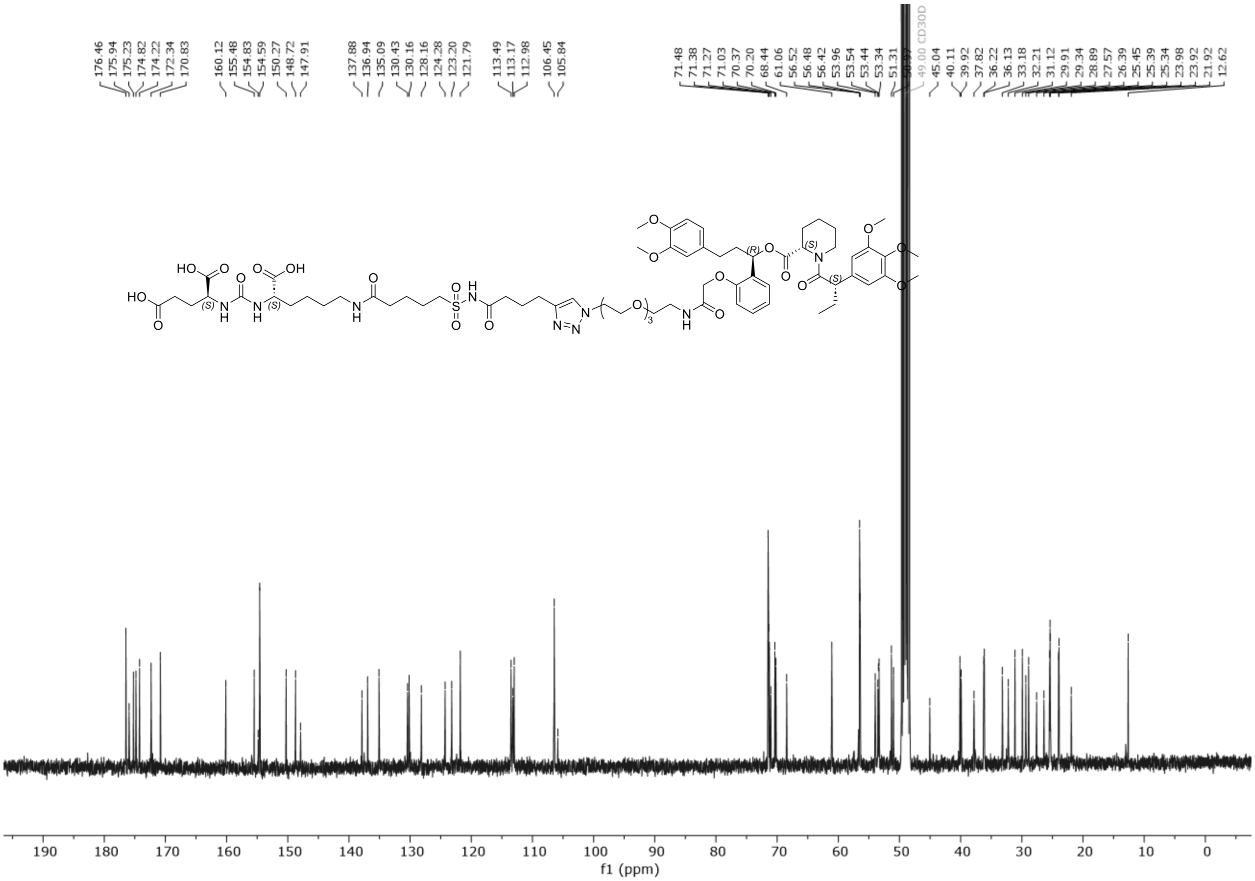

3.22.17 Compound **31** (Halo-BTK-GRC)

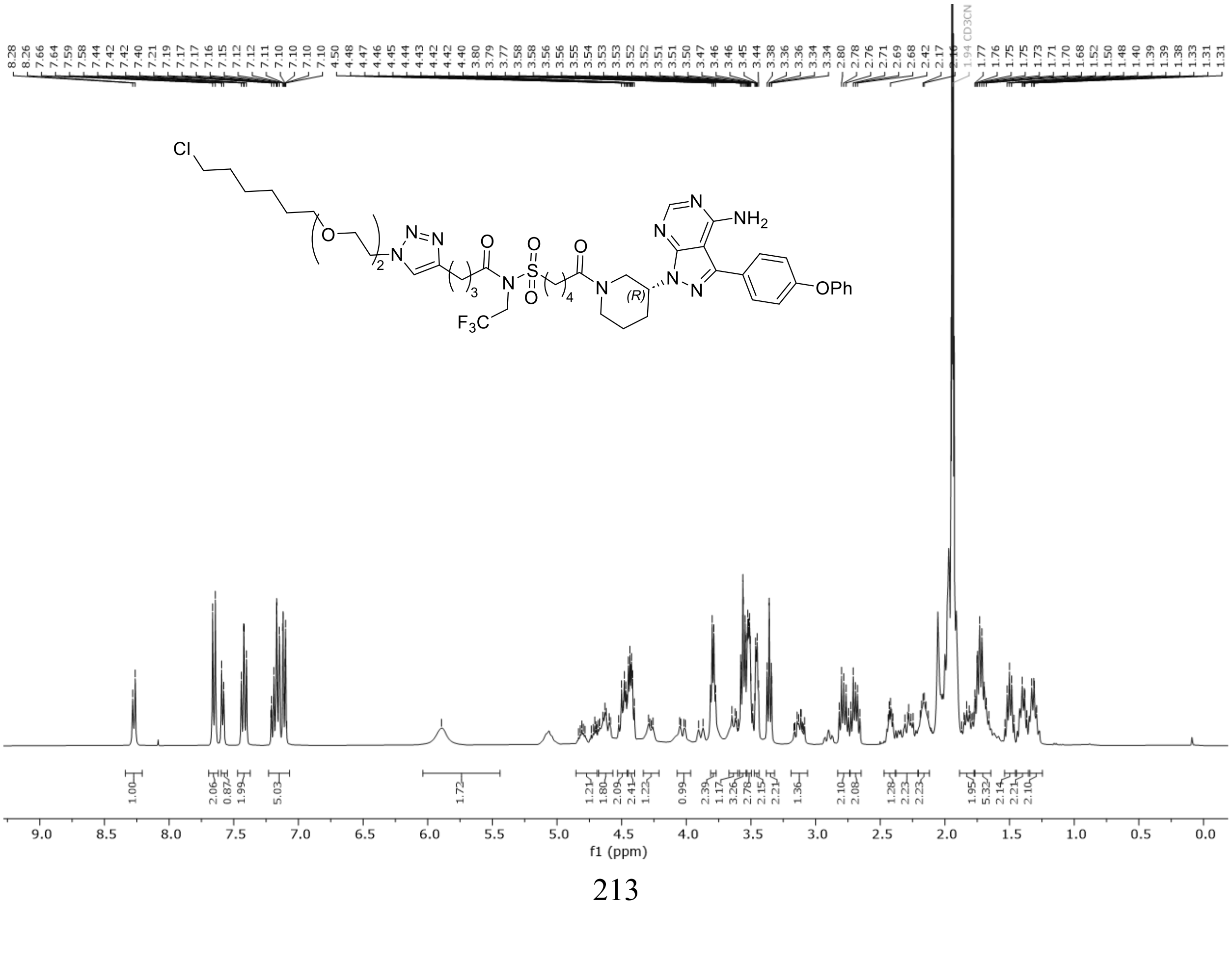

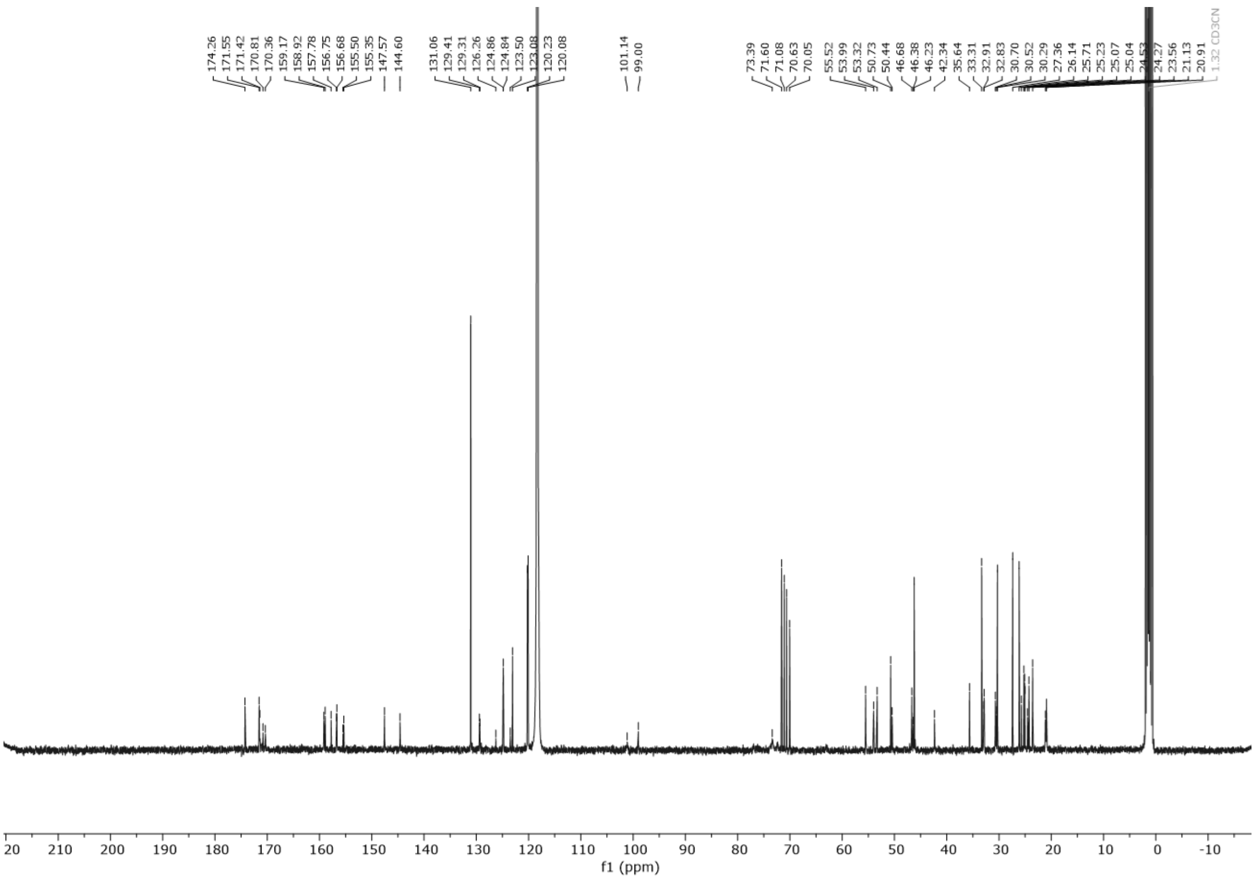
174.26
171.55
171.42
170.81
170.36
159.17
158.92
157.78
156.75
156.68
155.50
155.35
147.57
144.60
131.06
129.41
129.31
126.26
124.86
124.84
123.50
123.08
120.23
120.08
101.14
99.00
73.39
71.60
71.08
70.63
70.05
55.52
53.99
53.32
50.73
50.44
46.68
46.38
46.23
42.34
35.64
33.31
32.91
32.83
30.70
30.52
30.29
27.36
26.14
25.71
25.23
25.07
25.04
24.53
24.27
23.56
21.13
20.91
1.32 CD3CN
f1 (ppm)

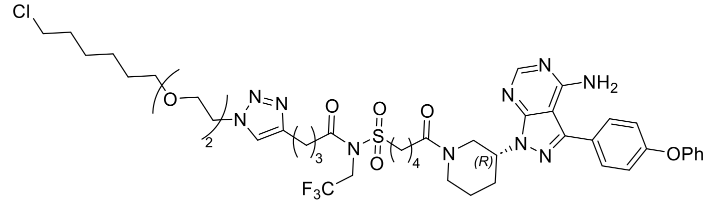
Cl
O
N
N
N
NH2
OPh
F3C
(R)

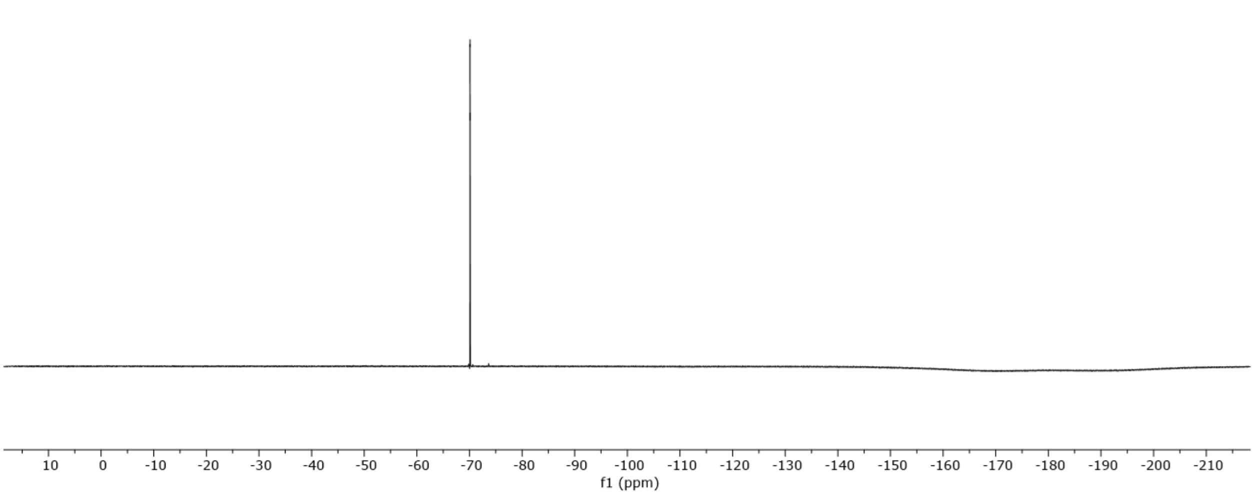
-70.10
-70.11
f1 (ppm)

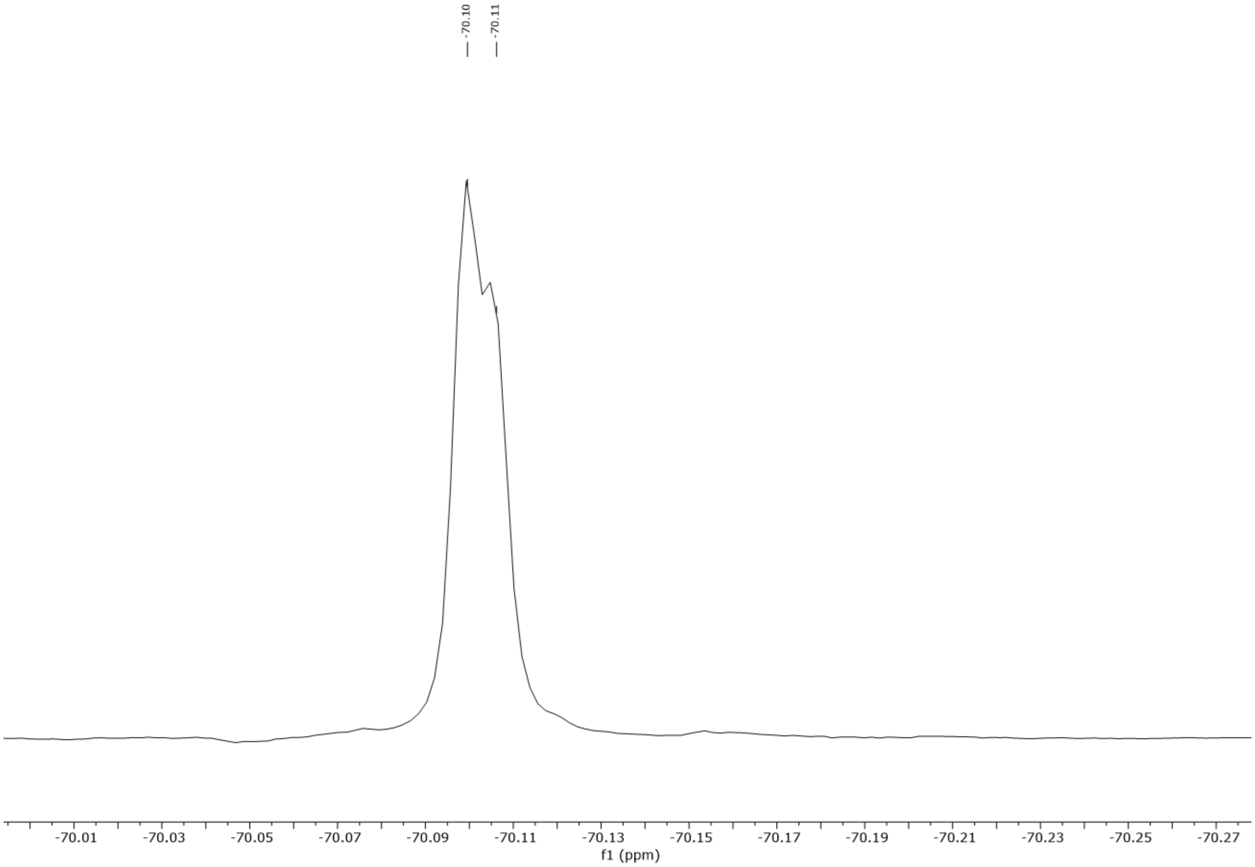
-70.10
-70.11
-70.01
-70.03
-70.05
-70.07
-70.09
-70.11
-70.13
-70.15
-70.17
-70.19
-70.21
-70.23
-70.25
-70.27
f1 (ppm)

3.22.18 <u>Compound **32** (Halo-BTK-iGRC)</u>

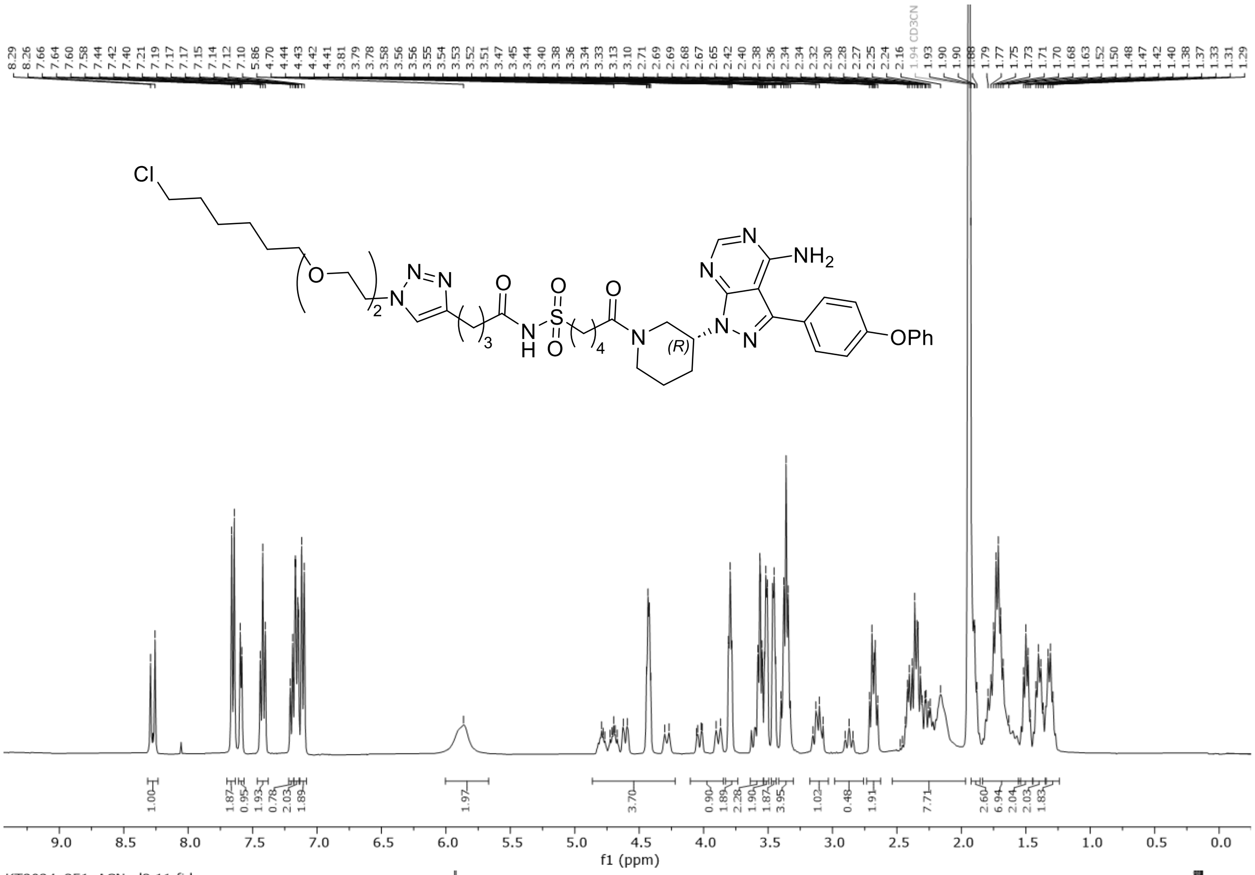

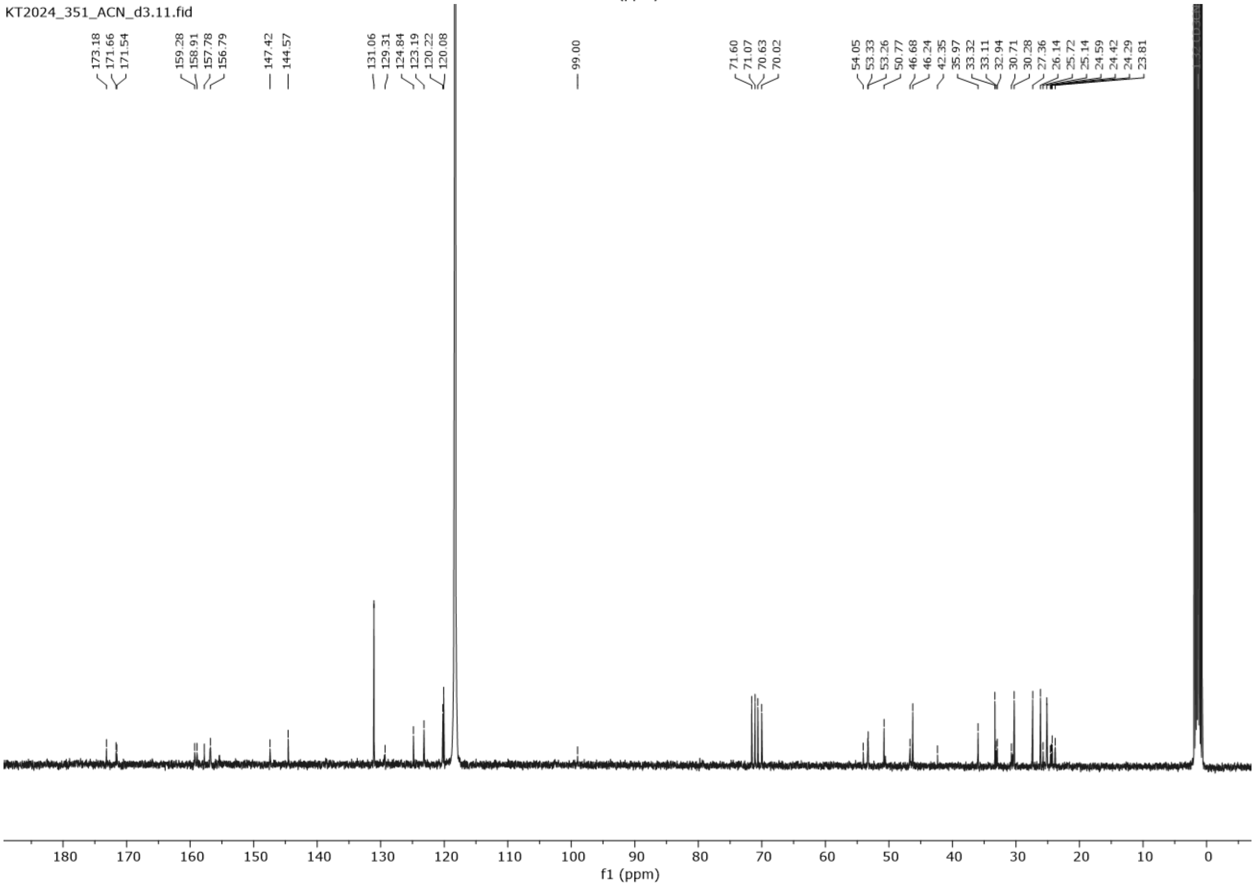

3.22.19 Compound **S6** (RIPK1 SuFA probe)

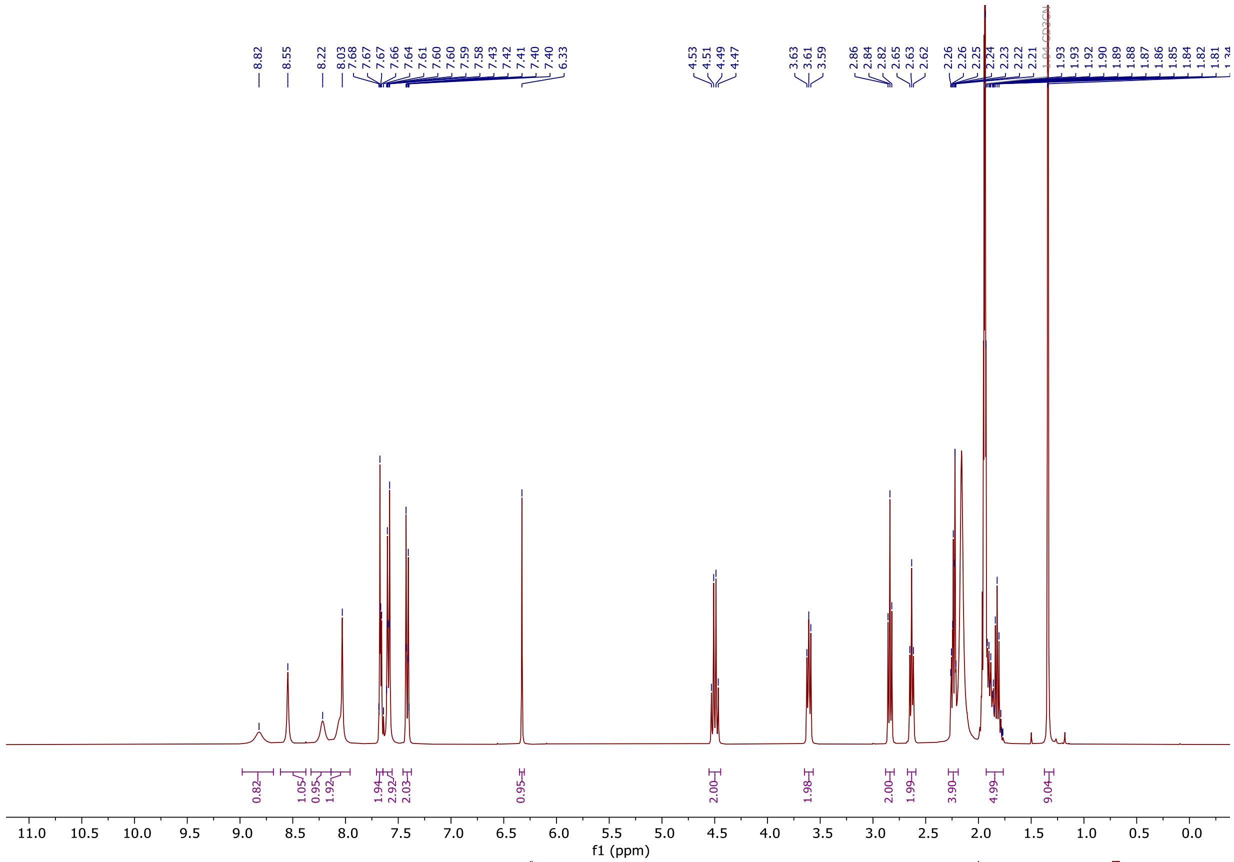

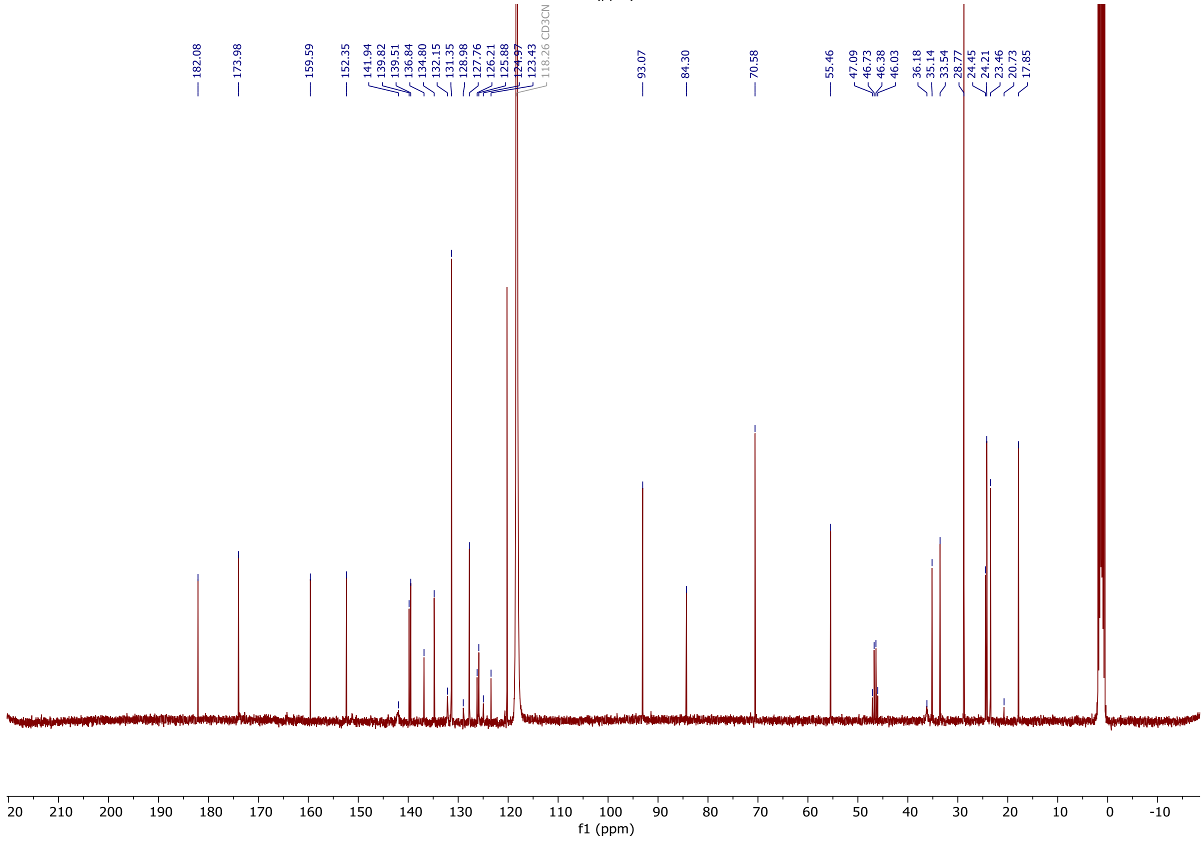

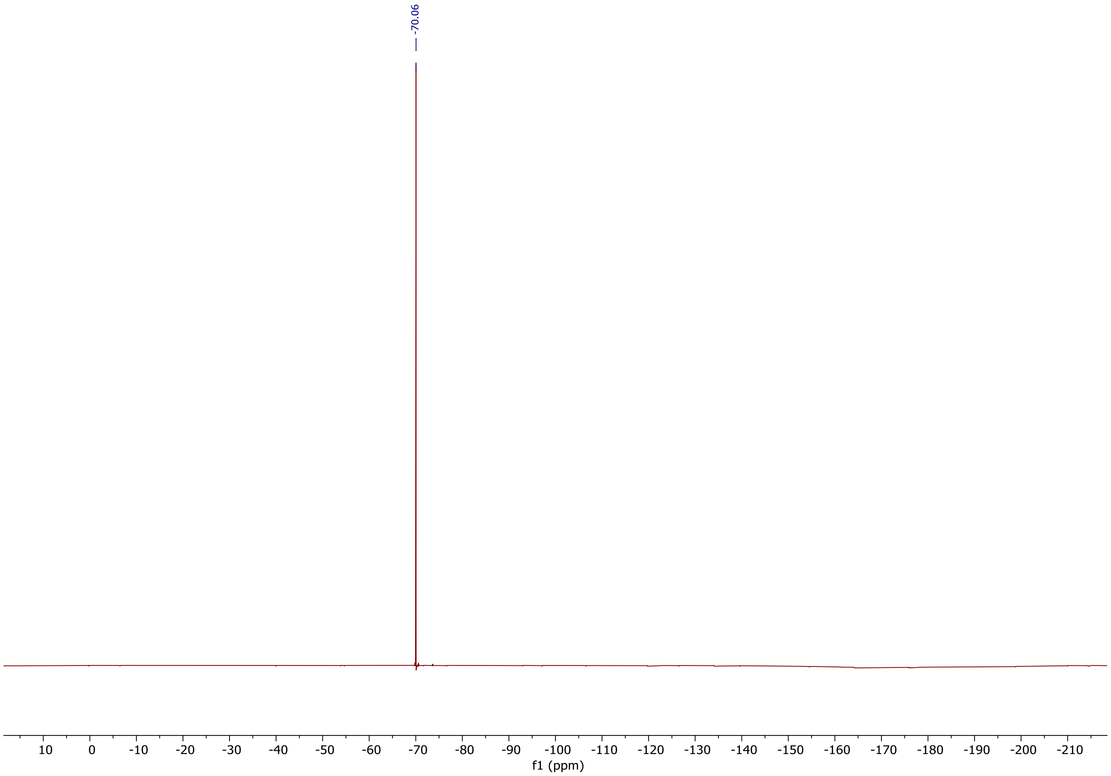
-70.06
10 0 -10 -20 -30 -40 -50 -60 -70 -80 -90 -100 -110 -120 -130 -140 -150 -160 -170 -180 -190 -200 -210
f1 (ppm)

3.22.20 Compound **S7** (VEGFR2 NASA probe)

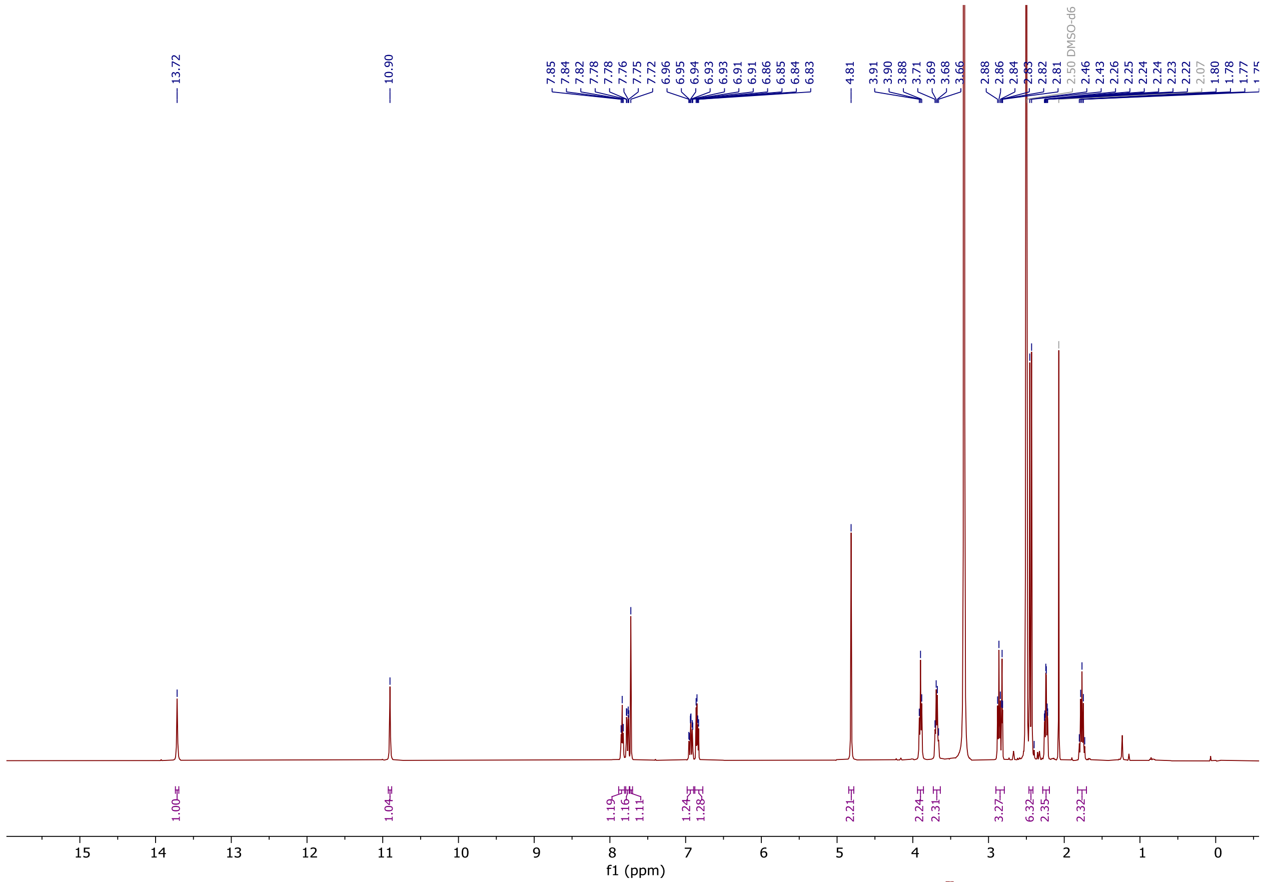

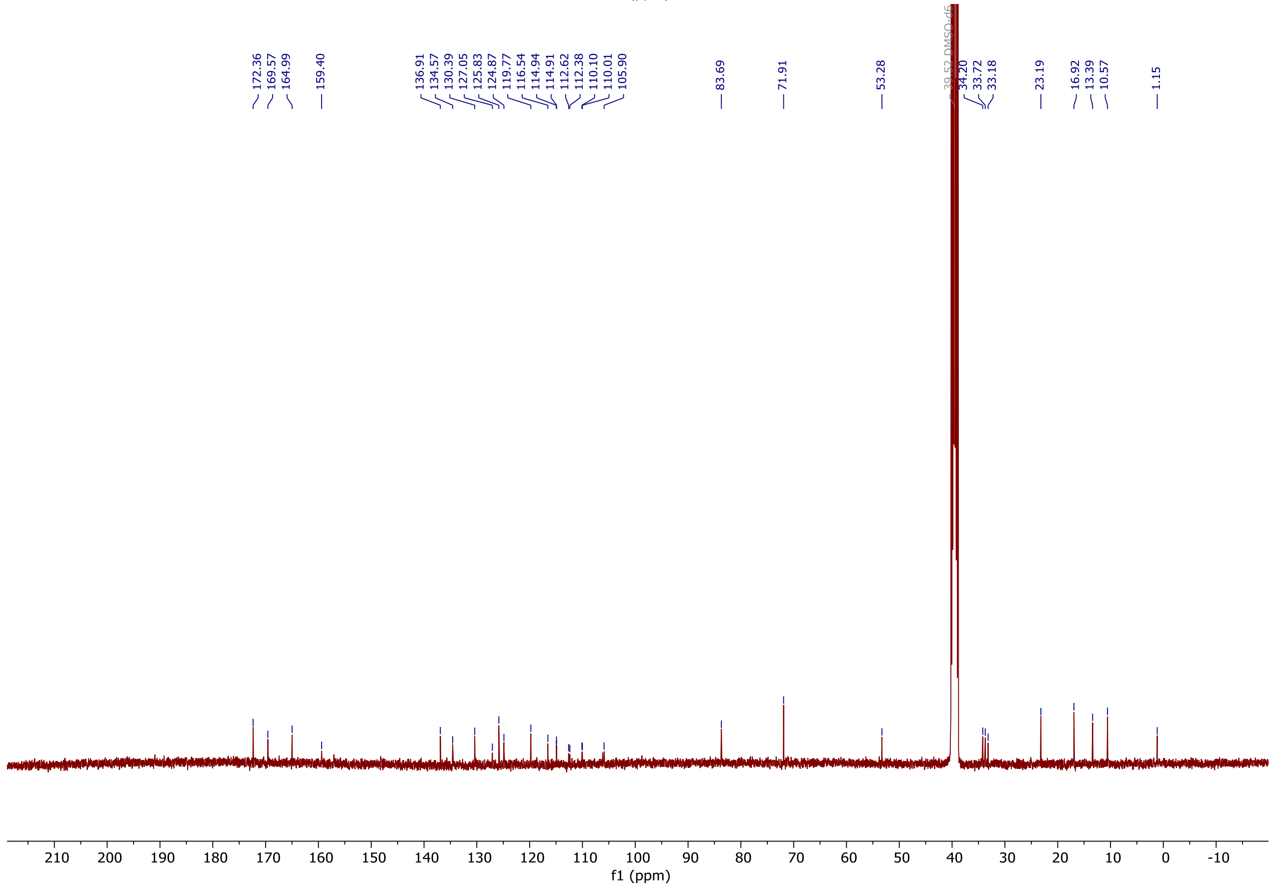

4 References

[1] R. Wang, X. Fang, Y. Lu, S. Wang, *J Med Chem* **2004**, *47*, 2977-2980.
[2] M. Attene, *The visual computer* **2010**, *26*, 1393-1406.
[3] https://github.com/nmwsharp/potpourri3d.
[4] aL. Krause, R. Herbst-Irmer, G. M. Sheldrick, D. Stalke, *Appl Crystallogr* **2015**, *48*, 3-10; b.
[5] G. M. Sheldrick, *Acta Crystallogr. A* **2015**, *71*, 3-8.
[6] G. M. Sheldrick, *Acta Crystallogr. C* **2015**, *71*, 3-8.
[7] O. V. Dolomanov, L. J. Bourhis, R. J. Gildea, J. A. Howard, H. Puschmann, *Appl Crystallogr* **2009**, *42*, 339-341.
[8] A. S. I. Accelrys DS Visualizer v2.0.1, **2007**.
[9] P. S. Pedersen, D. C. Blakemore, G. M. Chinigo, T. Knauber, D. W. C. MacMillan, *J Am Chem Soc* **2023**, *145*, 21189-21196.
